# Intramolecular Tricarbonyl‐Ene Reactions and α‐Hydroxy‐β‐Diketone Rearrangements Inspired by the Biosynthesis of Polycyclic Polyprenylated Acylphloroglucinols

**DOI:** 10.1002/anie.202203311

**Published:** 2022-07-14

**Authors:** Andreas B. zur Bonsen, Ricardo A. Peralta, Thomas Fallon, David M. Huang, Jonathan H. George

**Affiliations:** ^1^ Department of Chemistry The University of Adelaide Adelaide SA 5005 Australia

**Keywords:** Biomimetic Synthesis, Carbonyl-Ene Reaction, Cascade Reactions, Natural Products, Rearrangement

## Abstract

Structurally unique natural products pose biosynthetic puzzles whose solution can inspire new chemical reactions. Herein, we propose a unified biosynthetic pathway towards some complex meroterpenoids—the hyperireflexolides, biyoulactones, hybeanones and hypermonones. This hypothesis led to the discovery of uncatalyzed, intramolecular carbonyl‐ene reactions that are spontaneous at room temperature. We also developed an anionic cascade reaction featuring an α‐hydroxy‐β‐diketone rearrangement and an intramolecular aldol reaction to access four distinct natural product scaffolds from a common intermediate.

## Graphical abstract

Chemical speculation on the biosynthesis of some complex meroterpenoids led to the discovery of intricate cascades that feature spontaneous intramolecular carbonyl‐ene reactions, α‐hydroxy‐β‐diketone rearrangements, and intramolecular aldol reactions. The combination of these transformations allowed divergent access to four distinct natural product scaffolds from a common intermediate, mimicking their proposed biosynthetic pathways.

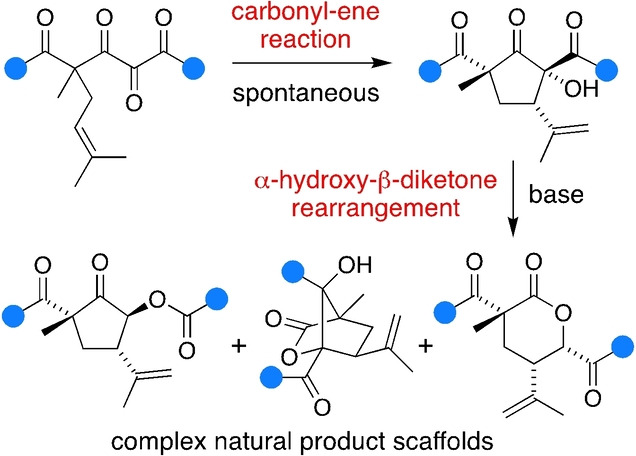

## Introduction

**Figure 1:**
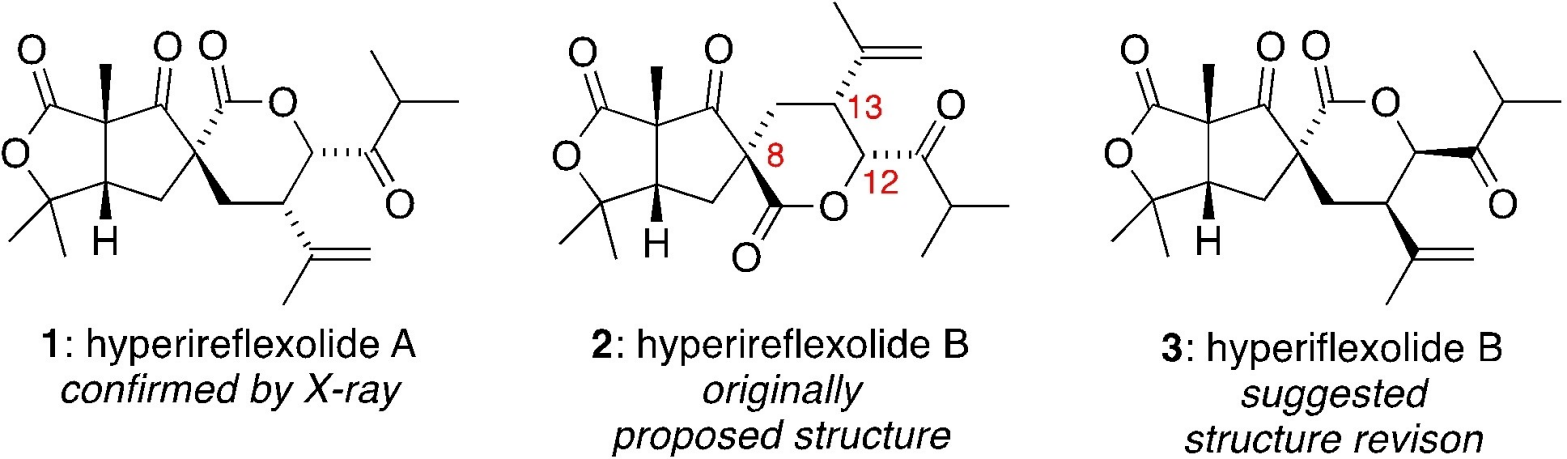
Hyperireflexolides A and B.

Polycyclic polyprenylated acylphloroglucinols (PPAPs) are a large family of highly oxygenated meroterpenoids produced almost exclusively by plants of the *Hypericum* and *Garcinia* genera.^[1]^ Although most PPAPs contain a common bicyclo[3.3.1]nonane‐2,4,9‐trione core that can be linked to a mixed mevalonate‐polyketide biosynthetic pathway, there are several non‐canonical PPAP sub‐families whose biogenetic origins are more obscure. For example, hyperireflexolide A (**1**) and hyperireflexolide B (**2**, initially proposed structure)^[2]^ are spirocyclic bislactone natural products^[3]^ isolated as racemates^[4]^ from *Hypericum reflexum* (Figure 1). Although **1** and **2** were originally considered to be oxidized derivatives of abietane terpenes, herein we propose a cryptic PPAP biosynthetic pathway that also explains their racemic nature. The structure of hyperireflexolide A (**1**) was determined by X‐ray crystallography, while hyperireflexolide B was assigned as **2** by NMR studies. Compared to **1**, structure **2** has the opposite relative configuration at the C8, C12 and C13 stereocentres. However, our biosynthetic hypothesis predicts that the structure of hyperireflexolide B should be reassigned to **3**, with the same relative configuration at the spirocyclic C8 stereocentre as in **1**.

**Figure 2:**
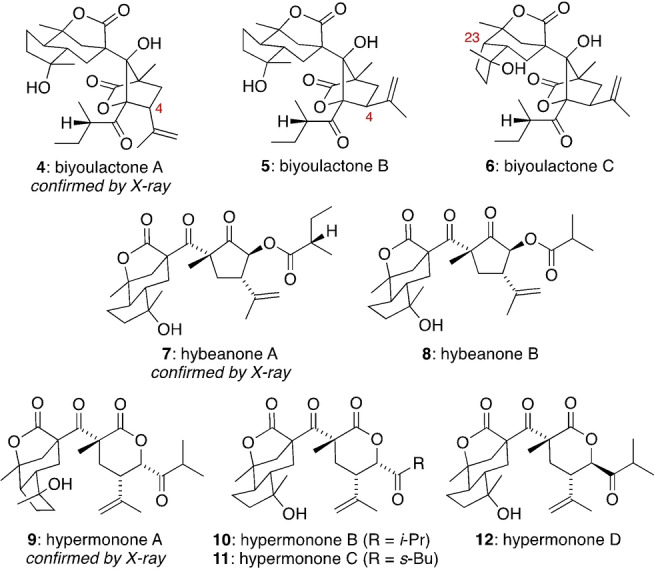
The biyoulactone, hybeanone and hypermonone family.

Biyoulactones A, B and C (**4**, **5** and **6**) are pentacyclic bislactone meroterpenoids^[5]^ isolated from *Hypericum chinense* (Figure 2). The structure of **4** was determined by X‐ray crystallography, while **5** and **6** were assigned as the C4‐ and C23‐epimers of **4** via NMR spectroscopy. Although the biyoulactones were recognized as rearranged PPAPs on their isolation, we suggest a revised biosynthetic pathway that closely mirrors the origin of the hyperireflexolides. The distinctive tricyclic γ‐lactone ring system of the biyoulactones is shared by the biosynthetically related hybeanones A and B (**7** and **8**), isolated from *Hypericum beanii*,^[6]^ and also hypermonones A, B, C and D (**9**, **10**, **11** and **12**) which were found in *Hypericum monogynum*.^[7]^

**Figure 3:**
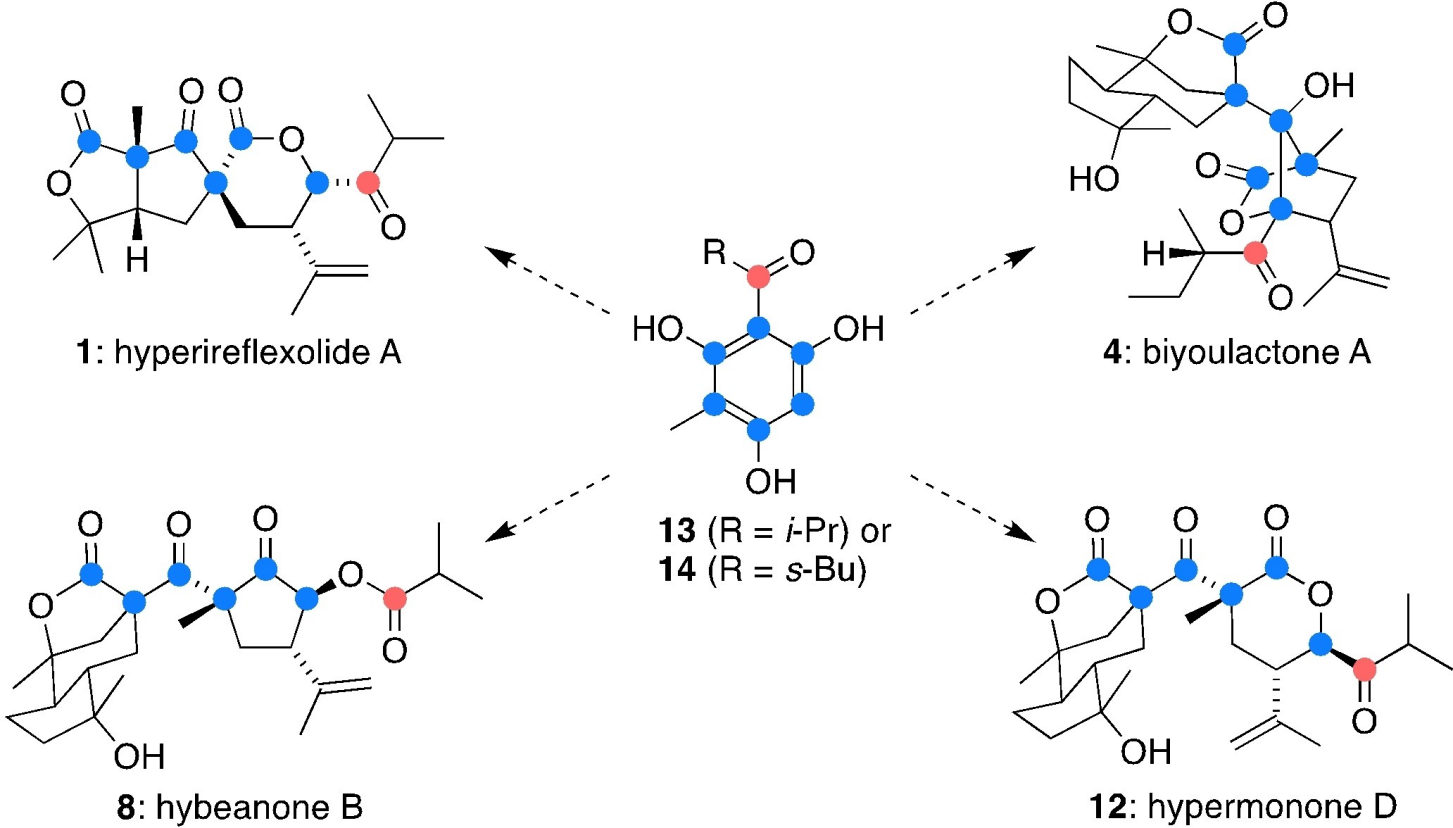
Comprehensive dearomatization of acylphloroglucinols in the biosynthesis of rearranged, non‐canonical PPAPs.

Herein, we propose that the hyperireflexolide, biyoulactone, hybeanone and hypermonone polycyclic ring systems are all constructed in nature via sequences of intramolecular carbonyl‐ene reactions and α‐hydroxy‐β‐diketone rearrangements. Both these transformations are rare in biosynthetic pathways, but here they rationalise how simple acylphloroglucinol building blocks such as **13** and **14** undergo prenylation, dearomatization and fragmentation reactions to give complex natural products such as **1**, **4**, **8** and **12** (Figure 3). The diverse fate of the highlighted phloroglucinol carbon atoms exemplifies the power of dearomatization in natural product biosynthesis.^[8]^

## Results and Discussion

**Scheme 1:**
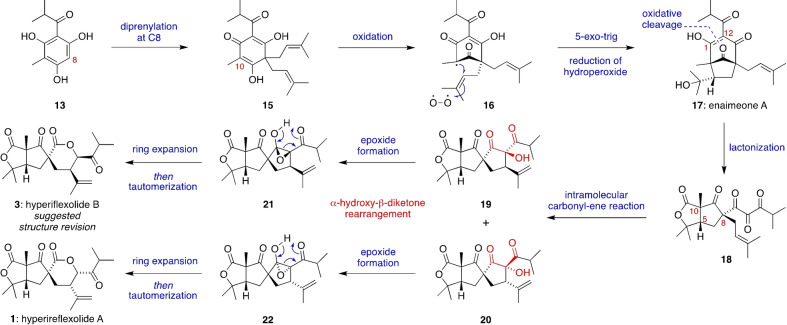
Proposed biosynthesis of hyperireflexolides A and B via an intramolecular carbonyl‐ene reaction and an α‐hydroxy‐β‐diketone rearrangement.

Our biosynthetic proposal for hyperireflexolides A and B (**1** and **3**), which asserts their meroterpenoid and PPAP origin, is outlined in Scheme 1. Acylphloroglucinol **13** is an aromatic polyketide commonly invoked in the biosynthesis of PPAPs, and it is the aglycone component of a recently isolated glycoside.^[9]^ Di‐prenylation of **13** at C8 (hyperireflexolide numbering) gives the dearomatized β‐acid **15**, which could undergo single electron oxidation at C10 to form the stabilized β‐diketo radical **16**. Cyclization of **16** via a 5‐exo‐trig addition to one of the two equivalent prenyl side chains, followed by trapping of a tertiary radical intermediate with triplet oxygen and reduction of the resultant hydroperoxide, would give enaimeone A (**17**).^[10]^ This bicyclo[3.2.1]octane PPAP natural product has been isolated from *Hypericum papuanum*.^[11]^ Next, oxidative cleavage of the C1–C12 double bond, perhaps mediated by singlet oxygen,^[12]^ would release a 1,2,3‐triketone motif and a carboxylic acid group, which could combine with the nearby tertiary alcohol to give the bicyclic lactone **18**. Compound **18** is a key intermediate in this biosynthetic pathway, containing the fused γ‐lactone ring system of the hyperireflexolides and with the relative configurations at C5, C8 and C10 fixed. It is also at the same overall level of oxidation as the hyperireflexolides. We then propose a type I intramolecular carbonyl‐ene reaction^[13]^ between the 1,2,3‐triketone^[14]^ and the prenyl side chain of **18** to give cyclic α‐hydroxy‐β‐diketones **19** and **20** via two diastereomeric pericyclic transition states. Although carbonyl‐ene reactions typically require Lewis acid catalysis^[15]^ or thermal activation, 1,2,3‐triketones are known to be highly reactive enophiles in intermolecular reactions.^[16]^ We therefore proposed that an intramolecular carbonyl‐ene reaction such as **18**→**19**/**20** should be favourable, and indeed DFT calculations predict that it will occur spontaneously at room temperature (see later). The final step in the proposed biosynthetic pathway is the ring expansion of **19** and **20** to construct the spirocyclic δ‐lactones of hyperireflexolides A and B via an unusual α‐hydroxy‐β‐diketone rearrangement. First reported by Blatt and Hawkins in 1936,^[17]^ the α‐hydroxy‐β‐diketone rearrangement to give ester or lactone products has only rarely been applied in total synthesis^[18]^ or invoked in biosynthetic pathways.^[19]^ As proposed by House,^[20]^ the mechanism of the α‐hydroxy‐β‐diketone rearrangement can proceed via an anionic or a neutral, thermal pathway (as shown below) involving epoxide intermediates such as **21** and **22**. Cleavage of the epoxide C−C bond of **21**/**22** followed by diastereoselective tautomerization of the resultant enols then gives **1** and **3**. In addition to justifying the relative configuration of hyperireflexolides A and B, this biosynthetic proposal also rationalizes their racemic nature via cyclization of the achiral radical intermediate **16**.

**Scheme 2:**
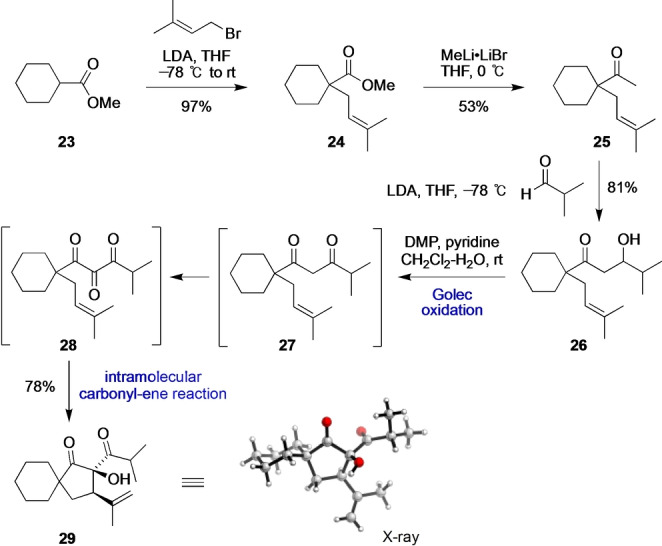
Hyperireflexolide‐inspired oxidative intramolecular carbonyl‐ene reaction of an intermediate 1,2,3‐triketone. LDA=lithium diisopropylamide, THF=tetrahydrofuran, DMP=Dess–Martin periodinane.

To gain insight into this unusual biosynthesis, we mimicked the intramolecular carbonyl‐ene reaction of the proposed biosynthetic intermediate 1,2,3‐triketone **18** in a simplified model spirocyclic system (Scheme 2). First, methyl cyclohexanecarboxylate (**23**) was α‐prenylated with LDA/prenyl bromide to give **24**, followed by addition of MeLi to give methyl ketone **25**. An intermolecular aldol reaction between the lithium enolate derived from **25** and isobutyraldehyde then gave the aldol adduct **26**. Golec and co‐workers have shown that β‐hydroxycarbonyl compounds can be oxidized to 1,2,3‐tricarbonyl compounds in one step (via an intermediate β‐dicarbonyl compound) using Dess–Martin periodinane (DMP) in the presence of pyridine.^[21]^ Meyer and Schreiber subsequently optimized this oxidation by the addition of 3 equiv of water.^[22]^ Using these modified conditions, we were pleased to observe the oxidative cyclization of **26** to give the cyclopentyl α‐hydroxy‐β‐diketone **29** in high yield as a single diastereomer. The relative configuration of **29** (determined using X‐ray crystallography) supports a concerted, type I intramolecular carbonyl‐ene reaction of 1,2,3‐triketone **28**. Although the β‐diketone intermediate **27** was observed as an isolable intermediate, **28** was not detected, which indicates that its intramolecular carbonyl‐ene reaction is spontaneous at room temperature. We are aware of only one previous example of an uncatalyzed, intramolecular carbonyl‐ene reaction at room temperature.^[23]^

**Scheme 3:**
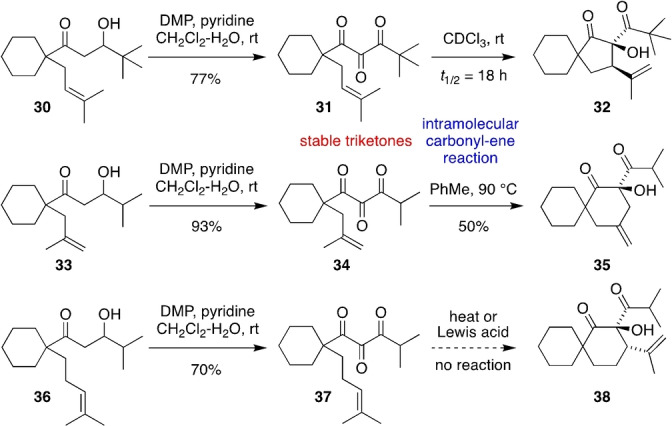
Isolation of some stable 1,2,3‐triketone intermediates in the oxidative intramolecular tricarbonyl‐ene reaction.

While screening substrates for the oxidative intramolecular tricarbonyl‐ene reaction, we found three examples of stable 1,2,3‐triketones (Scheme 3). Firstly, DMP‐mediated oxidation of the more sterically hindered *t*‐Bu derivative **30** gave triketone **31** in good yield. On standing in CDCl_3_ at room temperature, **31** gradually converted into **32** with a half‐life of 18 h via a slow type I intramolecular carbonyl‐ene reaction. Secondly, oxidation of the β‐methallyl derivative **33** gave a stable triketone **34** in high yield, which underwent a slow type II intramolecular carbonyl‐ene reaction to give the cyclohexyl α‐hydroxy‐β‐diketone **35** on heating in PhMe at 90 °C. Thirdly, oxidation of the homoprenyl derivative **36** gave triketone **37**, which did not undergo the anticipated type I intramolecular carbonyl‐ene reaction to give cyclohexanol **38** under thermal conditions or Lewis acid catalysis.

The substrate scope of the oxidative intramolecular tricarbonyl‐ene reaction was further extended to allow the fully diastereoselective synthesis of a variety of spirocyclic α‐hydroxy‐β‐dicarbonyl compounds (Table 1). The reaction tolerates variation at R_1_ to include β‐ketoesters (entry 1) and benzylic alcohols (entry 2), and at R_2_ to include a geranyl side chain (entry 3). In addition to cyclohexyl substrates, the spirocyclization also works on cyclobutyl (entry 4), cyclopentyl (entry 5) and cycloheptyl (entry 6) substrates. In all of these cyclizations, the intramolecular carbonyl‐ene reaction was too fast to allow observation of 1,2,3‐triketone intermediates, and only one diastereomer of product was formed. In general, all of these examples show rapid consumption of starting material, with some of the lower yields possibly due to slow oxidation of the intermediate 1,3‐diketones, or else undesired over‐oxidation by DMP. Highest yields were obtained for the formation of the less strained spircocyclic products such as **29** and **50**.

**Table 1:** Further substrate scope of the oxidative intramolecular tricarbonyl‐ene reaction.

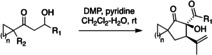			
			
Entry	Substrate	Product	Yield [%]
1	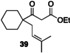	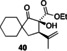	52
			
2	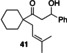	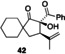	34
			
3	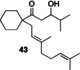	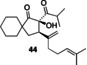	52
			
4	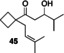	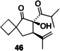	41
			
5	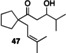	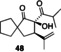	45
			
6	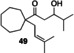	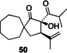	71

**Scheme 4:**
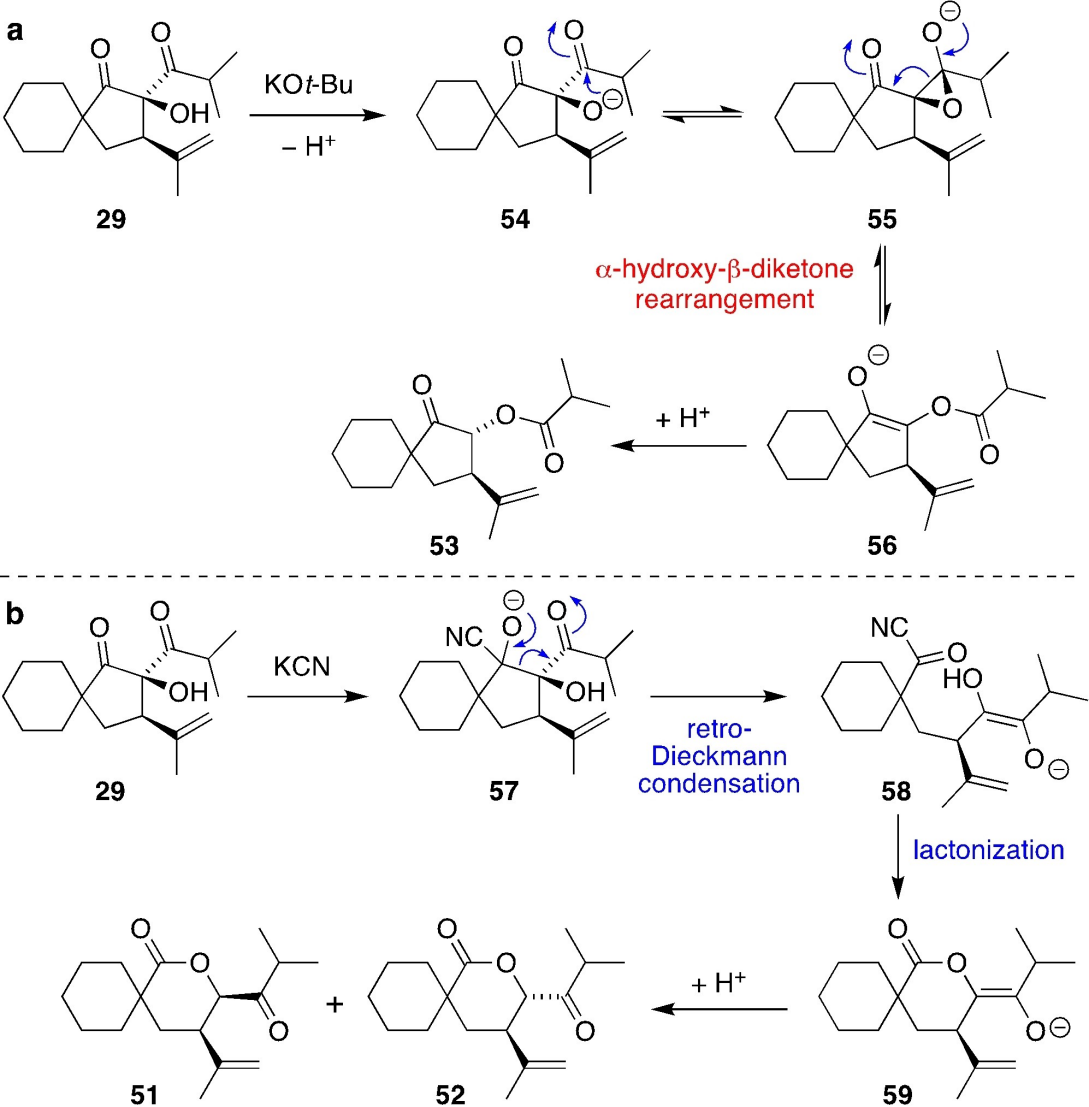
Proposed mechanism of the α‐hydroxy‐β‐diketone rearrangement of spirocycle **29** under a) base catalysis and b) nucleophilic catalysis.

Next, we explored some bioinspired α‐hydroxy‐β‐diketone rearrangements of **29**, which could occur in either an endocyclic mode to give δ‐lactones **51** and **52** (as required in hyperireflexolide biosynthesis), or in an exocyclic mode to give cyclopentyl ester **53** (Table 2). On heating in PhMe at 110 °C (entry 1), **29** was converted into a separable mixture of **51** (major product) and **52** (minor product) via a neutral ring expansion mechanism analogous to that proposed in Scheme 1. Alternatively, on exposure of **29** to a hindered base such as KO*t*‐Bu in THF (entry 2), *trans*‐cyclopentyl ester **53** was formed in good yield in 9 : 1 d.r. alongside the proposed *cis* diastereomer (which was not fully characterized). Under these basic conditions, the initially formed alkoxide anion **54** attacks the carbonyl group outside the 5‐membered ring, giving epoxide **55**, which fragments to give ester‐enolate **56** (Scheme 4a). Protonation of **56** then gives **53**, which has a similar cyclopentanone ring system to hybeanones A and B (**7** and **8**). The endocyclic α‐hydroxy‐β‐diketone rearrangement of **29** is clearly disfavoured under basic conditions. Recent work by Kieslich and Christoffers has shown that cyanide catalyses the ring expansion of cyclic α‐hydroxy‐β‐oxoesters.^[24]^ On exposure to 10 mol % KCN in PhMe at room temperature (entry 3), **29** was converted into a separable 2.5 : 1 mixture of spirocyclic δ‐lactones **51** and **52** in high overall yield. This ring expansion presumably proceeds via cyanide‐catalysed retro‐Dieckmann condensation of **29** to give **58** via **57**, followed by lactonization and then protonation of enolate **59** (Scheme 4b). Use of hydroxide, which could plausibly act as either a base or a nucleophilic catalyst for the α‐hydroxy‐β‐diketone rearrangement of **29**, gave a mixture of all three products in PhMe (entry 4), but only ester **53** in aqueous conditions (entry 5). We have therefore demonstrated that the carbonyl‐ene product **29** can be converted into the hyperireflexolide spirocyclic δ‐lactone ring system under simple reaction conditions via two distinct ring expansion mechanisms. However, since aqueous conditions exclusively favour the exocyclic rearrangement, the precise mechanism operating in the biosynthesis of hyperireflexolides A and B cannot be asserted at this stage.

**Table 2:** α‐Hydroxy‐β‐diketone rearrangements of spirocycle **29**.

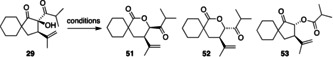				
				
Entry	Reagent	Solvent	*T*	Products
1	None	PhMe	110 °C	**51** (42 %)+**52** (7 %)
2	KO*t*‐Bu	THF	−78 °C	**53** (71 %, 9 : 1 d.r.)
3	10 mol % KCN	PhMe	rt	**51** (57 %)+**52** (23 %)
4	KOH	PhMe	rt	complex mixture of **51**/**52**/**53**
5	KOH	H_2_O	rt	**53** (35 %, 12 : 1 d.r.)

**Scheme 5:**
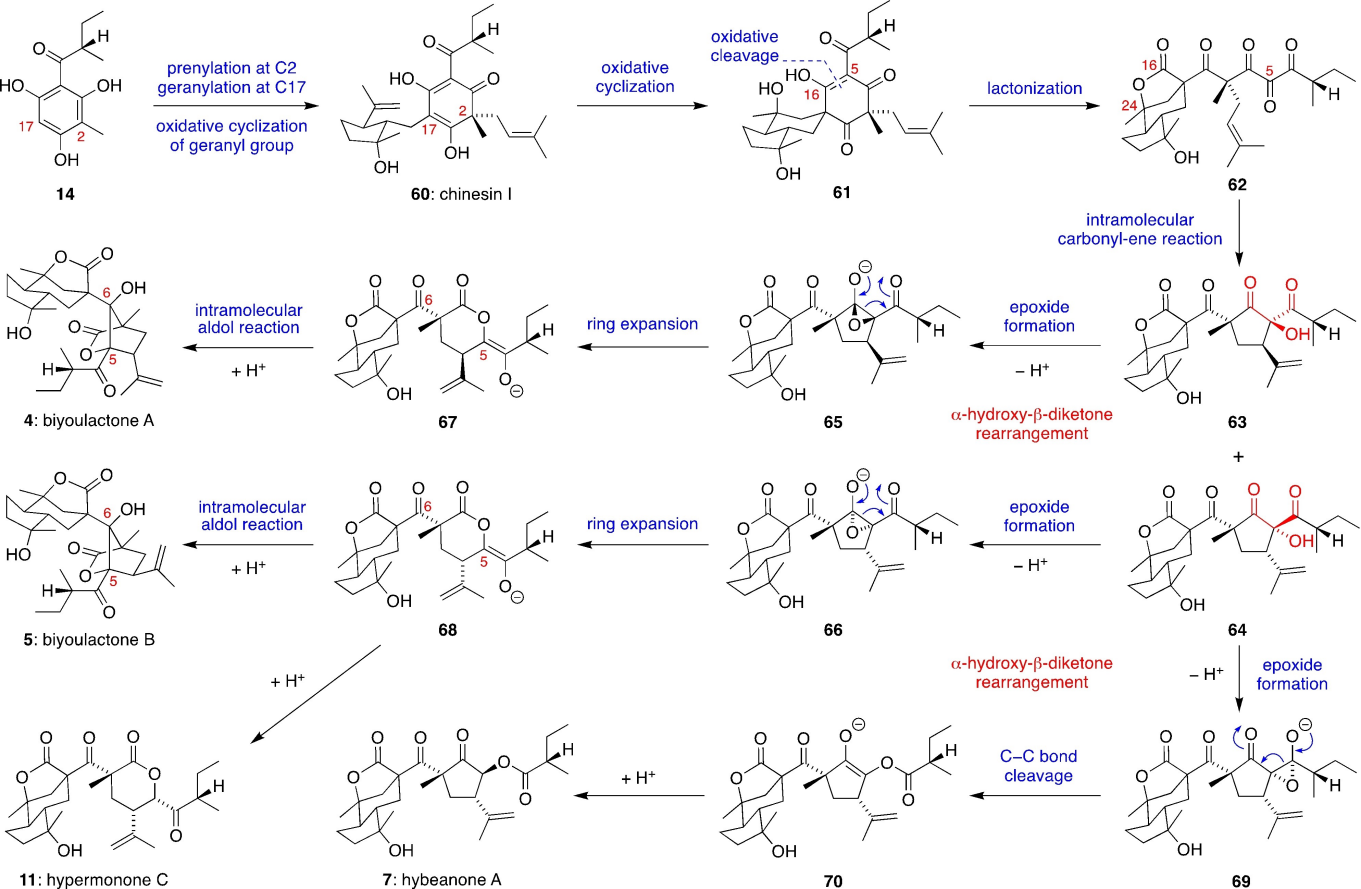
Proposed biosynthesis of biyoulactone, hypermonone and hybeanone natural products via divergent intramolecular carbonyl‐ene reactions, α‐hydroxy‐β‐diketone rearrangements and intramolecular aldol reactions.

Having investigated the chemical viability of our proposed biosynthesis of the hyperireflexolides, we searched for PPAP meroterpenoids with similar δ‐lactone ring systems, leading to the biyoulactones, the hypermonones, and the biosynthetically related hybeanone cyclopentanones.^[25]^ Our unified biosynthesis of this PPAP family (Scheme 5) is analogous to that of the hyperireflexolides, with key intramolecular carbonyl‐ene reactions and α‐hydroxy‐β‐diketone rearrangements. Firstly, prenylation and geranylation of acylphloroglucinol **14** ^[26]^ at C2 and C17, respectively (biyoulactone numbering), followed by oxidative cyclization of the geranyl group, would give the dearomatized natural product chinesin I (**60**). This PPAP meroterpenoid has been co‐isolated from *Hypericum chinense* alongside the biyoulactones.^[27]^ Oxidative cyclization of **60** could then form **61**, a diastereomer of chipericumin D^[28]^ (another PPAP found in *Hypericum chinense*). The oxidative cyclization of **60** could proceed by a 6‐endo‐trig radical cyclization of a stabilized β‐diketo radical followed by trapping with molecular oxygen, or else via 6‐endo‐tet cyclization of an epoxide intermediate.

Next, oxidative cleavage of the C5–C16 alkene of **61** and lactonization of the resultant carboxylic acid with the nearby tertiary alcohol at C24 would give the key 1,2,3‐triketone intermediate **62**. The tricyclic γ‐lactone ring system of **62** is also found in related meroterpenoids such as furanmonogones A and B,^[29]^ and hypercohone G.^[30]^ A spontaneous intramolecular carbonyl‐ene reaction between the 1,2,3‐triketone and the prenyl side chain of **62** would then give the diastereomeric cyclopenantone intermediates **63** and **64**. The cyclic α‐hydroxy‐β‐diketone motifs of **63** and **64** (which are perhaps undiscovered natural products^[31]^) are now poised to undergo further rearrangements. Firstly, deprotonation of **63**/**64** could trigger endocyclic α‐hydroxy‐β‐diketone rearrangements to give lactone‐enolates **67**/**68** via fragmentation of the intermediate epoxides **65**/**66**. The enediolates **67**/**68** are then primed for an intramolecular aldol reaction between the nucleophilic C5 position and the C6 ketone to form the bicyclic δ‐lactones of biyoulactones A and B (**4** and **5**). Alternatively, protonation of **68** at C5 would give hypermonone C (**11**). Finally, an exocyclic α‐hydroxy‐β‐diketone rearrangement of **64** could give ester‐enolate **70** via C−C bond cleavage of epoxide **69**, followed by protonation to give hybeanone A (**7**).

**Scheme 6:**
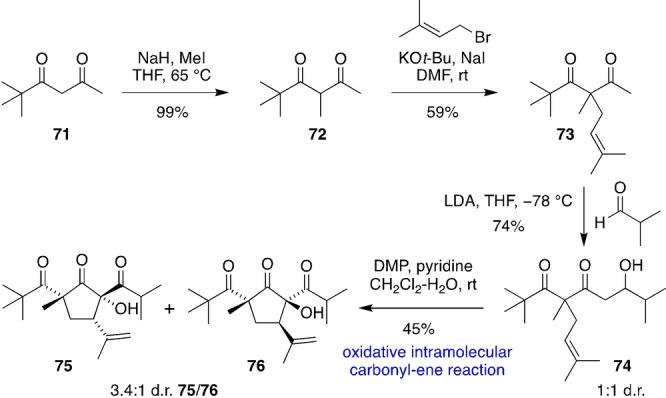
Biyoulactone‐inspired oxidative intramolecular tricarbonyl‐ene reaction. DMF=*N*,*N*‐dimethylformamide.

We mimicked the key steps of the proposed biosynthesis of the biyoulactone/hypermonone/hybeanone PPAP family in a simplified model system (Scheme 6). Methylation of the commercially available β‐diketone **71** with NaH/MeI gave **72**, which was prenylated with LDA/prenyl bromide to give **73**. An intermolecular aldol reaction between the lithium enolate derived from **73** and isobutyraldehyde formed β‐hydroxyketone **74** as an inconsequential 1 : 1 mixture of diastereomers. DMP‐mediated oxidative intramolecular tricarbonyl‐ene reaction of **74** then gave the highly functionalized cyclopentanones **75** and **76** as an inseparable 3.4 : 1 mixture of diastereomers in favour of **75**. The relative configurations of **75** and **76** were determined via NOESY correlations.

With **75** and **76** in hand, which are simplified models for the proposed biosynthetic intermediates **63** and **64**, we could explore some further bioinspired cascade reactions (Table 3). Under thermodynamic, basic conditions of KO*t*‐Bu in THF room temperature (entry 1), the anionic α‐hydroxy‐β‐diketone rearrangement of **75**/**76** occurs exclusively outside the ring, to give hybeanone analogue **79** as the major product alongside significant decomposition. Use of lithium bis(trimethylsilyl)amide (LiHMDS) in THF at −78 °C (entry 2) gave a higher yield of **79**, alongside a small amount of biyoulactone A analogue **80**. Upon further lowering the temperature (LDA in THF at −98 °C, entry 3), we observed the formation of the biyoulactone substructures **77** and **80** (33 % combined yield) via an intricate cascade featuring an endocyclic α‐hydroxy‐β‐diketone rearrangement and an intramolecular aldol reaction that closely mirrors the proposed biosynthesis of the biyoulactones. The NMR spectra of **77** and **80** show strong similarity to biyoulactones A and B, and their structures were confirmed by X‐ray crystallography. Given that the biyoulactone A analogue **80** must derive from the minor α‐hydroxy‐β‐diketone diastereomer **76**, its formation in 21 % yield in this reaction indicates that the anionic rearrangement and aldol reaction of this diastereomer is efficient and selective. A small amount of the hypermonone δ‐lactone **78** was also formed using LDA at at −98 °C. Use of KCN in PhMe (entry 4) allowed synthesis of the hypermonone analogue **78** in greater yield via an endocyclic α‐hydroxy‐β‐diketone rearrangement under nucleophilic catalysis. Finally, under thermal conditions **75**/**76** underwent extensive decomposition, although the ring expanded lactone **78** was isolated in low yield (entry 5). Although some of these cascade reactions are low yielding, the formation of four complex natural product scaffolds in two steps from the simple aldol adduct **74** gives some chemical evidence in favour of the unified biosynthetic pathway proposed in Scheme 5.

**Table 3:** Anionic rearrangement of α‐hydroxy‐β‐diketones **75** and **76** to give four distinct natural product scaffolds.

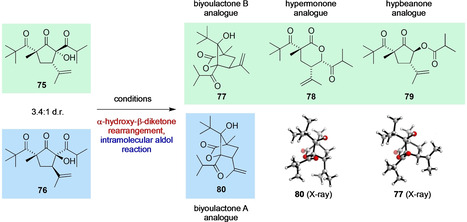				
				
Entry	Reagent	Solvent	*T*	Products
1	KO*t*‐Bu	THF	rt	**79** (43 %)+decomposition
2	LiHMDS	THF	−78 °C	**79** (50 %)+**80** (8 %)
3	LDA	THF	−98 °C	**77** (12 %)+**78** (6 %)+**79** (22 %)+**80** (21 %)
4	10 mol % KCN	PhMe	rt	**78** (41 %)
5	None	PhMe	110 °C	**78** (13 %)+decomposition

**Scheme 7:**
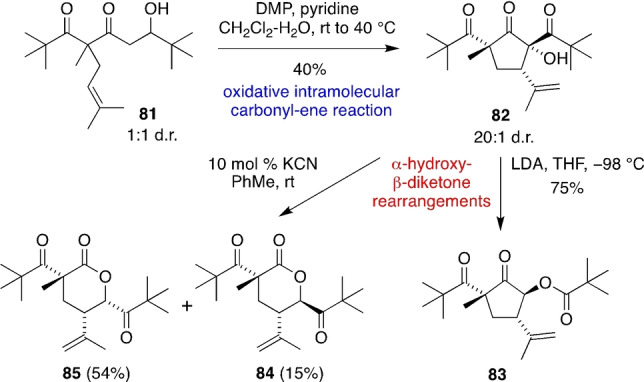
A highly diastereoselective oxidative intramolecular tricarbonyl‐ene reaction and subsequent α‐hydroxy‐β‐diketone rearrangements.

Given the complex product distribution of the anionic cascade reactions of **75**/**76**, we designed a slower, more diastereoselective oxidative intramolecular carbonyl‐ene reaction of a more sterically hindered substrate (Scheme 7). Thus, oxidation of the di‐*t*‐butylated aldol adduct **81** gave cyclic α‐hydroxy‐β‐diketone **82** in 20 : 1 d.r. via a now highly diastereoselective tricarbonyl‐ene reaction. Deprotonation of **82** with LDA then initiated an anionic α‐hydroxy‐β‐diketone rearrangement to give the hybeanone analogue **83** in 75 % yield, alongside trace quantities of isomeric by‐products. Alternatively, treatment of **82** with catalytic KCN triggered a nucleophilic α‐hydroxy‐β‐diketone rearrangement to give the ring‐expanded hypermonone analogues **84** and **85** in good overall yield. This sequence demonstrates the potential for the stereoselective synthesis of complex cyclopentanones and δ‐lactones via our bioinspired strategy.

**Scheme 8:**
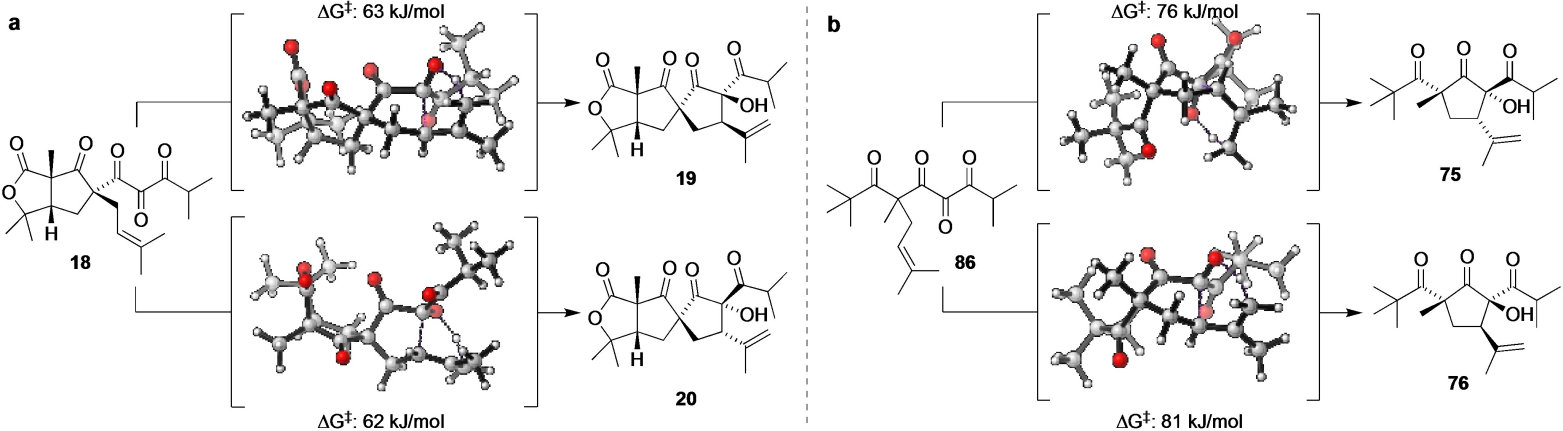
Computational analysis of the intramolecular tricarbonyl‐ene reaction of a) hyperireflexolide precursor **18** and b) model biyoulactone precursor **86**.

To gain further insight into the bioinspired intramolecular tricarbonyl‐ene reactions and anionic α‐hydroxy‐β‐diketone rearrangements discovered in this work, we turned to computational modelling using density functional theory (DFT) calculations. Geometry optimizations and frequency calculations were conducted using the M06‐2X method^[33]^ and 6‐31+G(d) basis set with single point energies calculated at 6‐311+G(d,p) using a SMD solvent continuum model^[34]^ (in water or THF). As proposed in the biosynthesis of hyperireflexolides A and B (Scheme 1), our modelling reveals concerted pericyclic carbonyl‐ene reactions of 1,2,3‐triketone **18** to give **19** and **20**, with remarkably low reaction barriers of 63 kJ mol^−1^ and 62 kJ mol^−1^, respectively (Scheme 8a). This result is consistent with the co‐isolation of both natural products, and supports our structure revision of hyperireflexolide B. Modelling of the carbonyl‐ene reaction of biyoulactone model 1,2,3‐triketone **86** (derived from oxidation of aldol adduct **74**) also revealed low energy transition states leading to diastereomers **75** and **76**, and correctly predicts **75** as the major product (Scheme 8b).

**Scheme 9:**
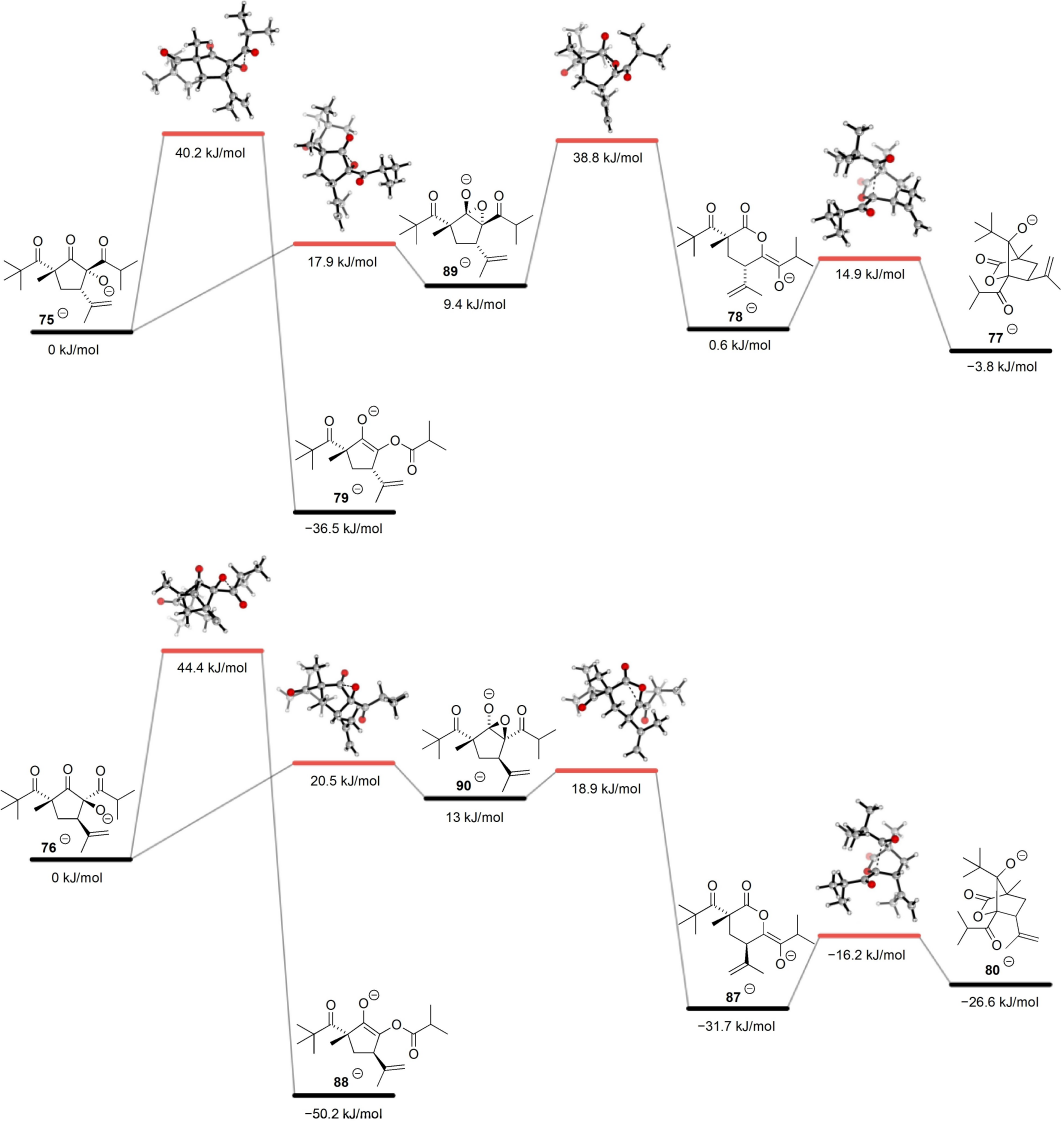
Computational analysis of the anionic α‐hydroxy‐β‐diketone rearrangement/aldol cascade of **75**/**76**.

The anionic α‐hydroxy‐β‐diketone rearrangement/aldol cascade of **75**/**76** models our proposed biosynthesis of the biyoulactones, hypermonones and hybeanones, with diverse reaction outcomes presenting a complex mechanistic and kinetic/thermodynamic scenario. Modelling of the anionic rearrangement pathways of **75** ^−^ and **76** ^−^ is presented in Scheme 9. Initially, the ground state anionic structures were modelled to predict their relative stabilities. For the reaction of **75** ^−^, the starting material, lactone‐enolate **78** ^−^ (−0.6 kJ mol^−1^) and aldol adduct **77** ^−^ (−3.8 kJ mol^−1^) are all very close in relative energy, with the ester‐enolate **79** ^−^ (−36.5 kJ mol^−1^) representing the thermodynamic anionic product. In the reaction of **76** ^−^, the aldol product **80** ^−^ has greater relative stability (−26.6 kJ mol^−1^), but with the ester‐enolate **88** ^−^ still the thermodynamic product (−50.2 kJ mol^−1^). In both cases, the extra stability of the more conformationally flexible ester‐enolates compared to the rigid, bicyclic aldol products is due to a significant thermal (largely entropic) contribution of around 19 kJ mol^−1^. The kinetic barriers to the intramolecular aldol reactions of lactone‐enolates **78** ^−^ and **87** ^−^ are both very low.

**Scheme 10:**
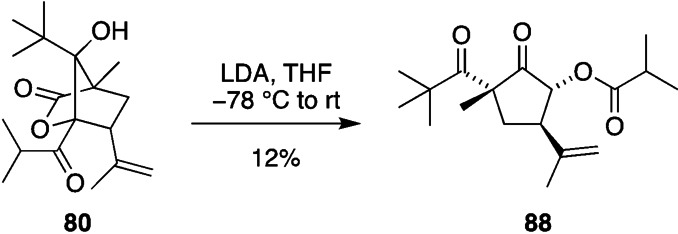
Anionic rearrangement of aldol product **80** to give ester **88**.

The key to reaction selectivity, therefore, is whether the initial α‐hydroxy‐β‐diketone rearrangement proceeds in an endo or exo sense relative to the five membered ring. For the endocyclic α‐hydroxy‐β‐diketone rearrangement of **75** ^−^, a low energy reaction path includes an epoxide intermediate **89** ^−^ (9.4 kJ mol^−1^) as a local minimum, which then collapses to the lactone‐enolate **78** ^−^. A very similar reaction profile is evident for diastereomer **76** ^−^, but this endocyclic α‐hydroxy‐β‐diketone rearrangement is more favored as the transition states for the formation and collapse of the transient epoxide **90** ^−^ are remarkably low in energy due to less steric crowding between the *tert*‐butyl and isopropenyl substituents. For the exocyclic α‐hydroxy‐β‐diketone rearrangements of both **75** ^−^ and **76** ^−^, a concerted reaction mechanism is predicted, with a higher barrier than the endocyclic pathway. While the mechanism of the anionic α‐hydroxy‐β‐diketone rearrangement has traditionally been proposed to proceed via an epoxide intermediate, no direct evidence for this intermediate has been demonstrated.^[35]^ Acyl substitution reactions generally proceed through a tetrahedral intermediate, although in some cases concerted substitution mechanisms have been established.^[36]^ For our anionic α‐hydroxy‐β‐diketone rearrangements there appears to be a fine line between a stepwise (endocyclic) or concerted (exocyclic) mechanisms. However, the kinetic preference for the endocyclic pathway is critical in determining the reaction outcome. Under low temperature, basic reaction conditions (Table 3, entry 3), we expect the rapid formation of a mixture of alkoxide **75** ^−^, lactone‐enolate **78** ^−^ and aldol product **77** ^−^, as well as gradual formation of the thermodynamic ester‐enolate **79** ^−^. This is consistent with the experimental observations, and the preference for ester **79** at higher temperatures. From alkoxide **76** ^−^, we expect the rapid formation of aldol product **80** ^−^, which should offer some kinetic stability to the formation of the thermodynamic ester‐enolate **88** ^−^. Indeed, when aldol product **80** was treated with LDA and warmed to room temperature, an ester **88** derived from protonation of **88** ^−^ was isolated in low yield via a remarkable sequence of α‐hydroxy‐β‐diketone rearrangements (Scheme 10).

## Conclusion

In summary, we used speculation on the biosynthesis of some unusual meroterpenoids to discover a series of oxidative intramolecular tricarbonyl‐ene reactions and α‐hydroxy‐β‐diketone rearrangements. These predisposed cascade reactions can be combined in sequence to generate highly substituted cyclopentanones and δ‐lactones found in the hyperireflexolide, biyoulactone, hypermonone and hybeanone frameworks, with a remarkable increase in molecular complexity in each case. Our bioinspired model syntheses also provide compelling chemical support for the proposed biosynthesis of these complex PPAPs from simple acylphloroglucinol precursors via a cryptic dearomatization pathway.

## Conflict of interest

The authors declare no conflict of interest.

1

## Supporting information

As a service to our authors and readers, this journal provides supporting information supplied by the authors. Such materials are peer reviewed and may be re‐organized for online delivery, but are not copy‐edited or typeset. Technical support issues arising from supporting information (other than missing files) should be addressed to the authors.

Supporting Information

Supporting Information

Supporting Information

Supporting Information

## Data Availability

The data that support the findings of this study are available in the Supporting Information of this article.
