# Supplementary material for: Intramolecular Tricarbonyl‐Ene Reactions and α‐Hydroxy‐β‐Diketone Rearrangements Inspired by the Biosynthesis of Polycyclic Polyprenylated Acylphloroglucinols
Source: Angew Chem Int Ed Engl. 2022 Jul 14;61(34):e202203311. doi: 10.1002/anie.202203311 (PMC9541541; doi:10.1002/anie.202203311)
Supplement: Supplementary file 4 — Supporting Information [file ANIE-61-0-s003.pdf]

Angewandte Chemie
Eine Zeitschrift der Gesellschaft Deutscher Chemiker

Supporting Information

Intramolecular Tricarbonyl-Ene Reactions and α-Hydroxy-β-Diketone Rearrangements Inspired by the Biosynthesis of Polycyclic Polyprenylated Acylphloroglucinols

*A. B. zur Bonsen, R. A. Peralta, T. Fallon, D. M. Huang, J. H. George**

Supporting Information

Table of Contents

1. General Methods..2
2. Synthetic Procedures..3
3. NMR Spectra..37
4. Single Crystal X-Ray Data..123
5. Computational Analysis..128

1. General Methods

All reactions using dry solvents were carried out under an inert nitrogen atmosphere. All chemicals were purchased from commercial suppliers and used as received. Thin layer chromatography was performed using aluminium sheets coated with silica gel F_{254}. Visualization was aided by viewing under a UV lamp and staining with vanillin or $KMnO_4$ stain followed by heating. The TLC stain solutions were prepared as follows. Vanillin stain: vanillin (6 g) was dissolved in EtOH (95 mL) followed by addition of conc. sulfuric acid (1.5 mL). $KMnO_4$ stain: $KMnO_4$ (0.75 g) and K_2CO_3 (5 g) were dissolved in H_2O (100 mL) and aq. NaOH (10%, 1 mL) was added. All R_f values were measured to the nearest 0.01. Flash column chromatography was performed using 40–63-micron grade silica gel. Melting points were recorded on a digital melting point apparatus and are uncorrected. Infrared spectra were recorded as the neat compounds using an FT-IR Shimadzu IRSpirit spectrometer fitted with an ATR head. High field NMR spectra were recorded using either a 500 MHz Agilent DD2 NMR spectrometer (^{1}H at 500 MHz, ^{13}C at 125 MHz) or a Agilent 600 MHz DD2 console spectrometer (^{1}H at 600 MHz, ^{13}C at 150 MHz) fitted with a Agilent One NMR broadband probe. The solvent used for NMR spectra was $CDCl_3$. ^{1}H chemical shifts are reported in ppm on the δ-scale relative to TMS (δ 0.0) or residual $CHCl_3$ (δ 7.26) and ^{13}C NMR chemical shifts are reported in ppm relative to residual $CHCl_3$ (δ 77.16). Multiplicities are reported as (br) broad, (s) singlet, (d) doublet, (t) triplet, (q) quartet, (quin) quintet, (sext) sextet, (sept) septet and (m) multiplet. All *J*-values were rounded to the nearest 0.1 Hz. ESI high resolution mass spectra were recorded on an Agilent 6230 TOF LC/MS mass spectrometer.

2. Synthetic Procedures

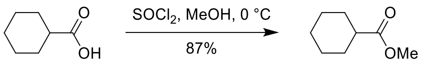

SI-1 23

Cyclohexane carboxylic acid (**SI-1**, 10.0 g, 78.0 mmol, 1.0 eq.) was dissolved in MeOH (320 mL) and cooled to 0 °C. $SOCl_2$ (6.8 mL, 93.6 mmol, 1.2 eq.) was added dropwise and the mixture was stirred at 0 °C for 2 h. The mixture was concentrated *in vacuo* to give **23** as a colourless oil (9.62 g, 67.7 mmol, 87%). Data for **23** matched that previously reported.[1]

Data for 23:
R_f = 0.60 (10:1, petroleum ether/EtOAc).
IR (neat): 2982, 2932, 2856, 1733, 1168, 1040 cm^{-1}.
^{1}H NMR (500 MHz, $CDCl_3$): δ 3.63 (s, 3H), 2.27 (tt, *J* = 11.3, 3.7 Hz, 1H), 1.89 – 1.84 (m, 2H), 1.73 – 1.69 (m, 2H), 1.63 – 1.58 (m, 1H), 1.45 – 1.37 (m, 2H), 1.29 – 1.17 (m, 3H) ppm.
^{13}C NMR (125 MHz, $CDCl_3$): δ 176.6, 51.5, 43.2, 29.1, 25.9, 25.5 ppm.

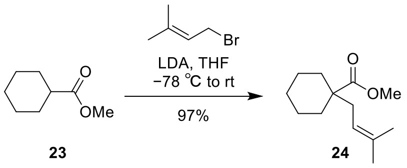

23 24

To a solution of diisopropylamine (4.48 mL, 31.9 mmol, 1.5 eq.) in dry THF (50 mL) at 0 °C was added *n*-BuLi (2.5 M in hexanes, 12.8 mL, 31.9 mmol, 1.5 eq.). The mixture was stirred for 30 min at 0 °C, then cooled to -78 °C. Ester **23** (2.95 g, 20.7 mmol, 1.0 eq.) was added and the mixture was stirred for 30 min at -78 °C, followed by addition of prenyl bromide (3.69 mL, 31.9 mmol, 1.5 eq.). The reaction was then slowly warmed to room temperature and stirred for 1.5 h. The reaction was quenched with sat. aq. NH_4Cl solution (50 mL). The organic layer was separated and the aqueous layer was extracted with Et_2O (2 × 50 mL). The combined organic layers were dried over Na_2SO_4, filtered and concentrated *in vacuo*. The residue was purified by flash column chromatography on SiO_2 (50:1, petroleum ether/EtOAc) to give **24** as a colourless oil (4.22 g, 20.1 mmol, 97%).

Data for 24:
R_f = 0.55 (20:1, petroleum ether/EtOAc).
IR (neat): 2982, 2928, 1728, 1452, 1211, 1129 cm^{-1}.
HRMS (ESI): calculated for $C_{13}H_{23}O_2$ 211.1693 $[M+H]^+$, found 211.1701.
^{1}H NMR (500 MHz, $CDCl_3$): δ 5.03 (t, *J* = 7.6 Hz, 1H), 3.65 (s, 3H), 2.17 (d, *J* = 7.5 Hz, 2H), 2.06 – 2.02 (m, 2H), 1.68 (s, 3H), 1.57 (s, 3H), 1.58 – 1.53 (overlapped m, 3H), 1.36 – 1.28 (m, 2H), 1.26 – 1.19 (m, 3H) ppm.
^{13}C NMR (125 MHz, $CDCl_3$): δ 177.2, 134.1, 119.3, 51.5, 47.9, 38.9, 34.0, 26.1, 26.1, 23.5, 17.9 ppm.

[1] R. Lerebours, C. Wolf, *J. Am. Chem. Soc.* **2006**, *128*, 13052.

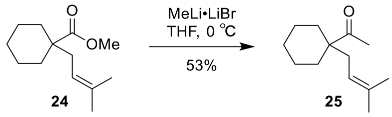

To a solution of **24** (200 mg, 0.95 mmol, 1.0 eq.) in dry THF (1.5 mL) at 0 °C was added methyllithium lithium bromide complex (1.5 M in Et_2O, 1.48 mL, 2.22 mmol, 2.33 eq.). The mixture was stirred for 1.5 h at 0 °C, quenched with sat. aq. NH_4Cl solution (10 mL) and extracted with Et_2O (3 × 10 mL). The combined organic layers were dried over Na_2SO_4, filtered and concentrated *in vacuo*. The residue was purified by flash column chromatography on SiO_2 (50:1→20:1 gradient elution, petroleum ether/EtOAc) to give **25** as a tan liquid (97.3 mg, 0.50 mmol, 53%).

Data for 25:
R_f = 0.53 (20:1, petroleum ether/EtOAc).
IR (neat): 2981, 2931, 1713, 1448, 1380, 1154 cm^{-1}.
HRMS (ESI): calculated for $C_{13}H_{23}O$ 195.1743 $[M+H]^+$, found 195.1752.
^{1}H NMR (500 MHz, $CDCl_3$) δ 4.94 (t, *J* = 7.5 Hz, 1H), 2.16 (d, *J* = 7.6 Hz, 2H), 2.09 (s, 3H), 1.99 – 1.95 (m, 2H), 1.67 (s, 3H), 1.58 (s, 3H), 1.57 – 1.47 (m, 3H), 1.32 – 1.24 (m, 5H) ppm.
^{13}C NMR (125 MHz, $CDCl_3$): 213.7, 134.3, 119.0, 52.8, 37.5, 33.3, 26.2, 26.1, 25.6, 23.2, 18.1 ppm.

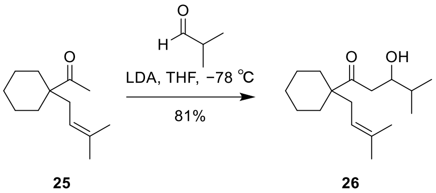

Diisopropylamine (1.73 mL, 12.3 mmol, 1.6 eq.) was dissolved in dry THF (45 mL) and cooled to 0 °C. *n*-BuLi (2.5 M in hexanes, 4.92 mL, 12.3 mmol, 1.6 eq.) was added and the mixture was stirred for 15 min. The mixture was cooled to -78 °C and **25** (1.50 g, 7.68 mmol, 1.0 eq.) was added. After stirring for 20 min isobutyrylaldehyde (0.98 mL, 10.8 mmol, 1.4 eq.) was added and the reaction was stirred for 1 h. Sat. aq. NH_4Cl (10 mL) and Et_2O (10 mL) was added and the dry ice bath was removed to warm the mixture to room temperature. The phases were separated and the aqueous phase was extracted with Et_2O (2 × 10 mL). The combined organic layers were dried over Na_2SO_4, filtered and concentrated *in vacuo*. The residue was purified by flash column chromatography on SiO_2 (60:1→40:1 gradient elution, petroleum ether/EtOAc) to give **26** as a colourless oil (1.66 g, 6.23 mmol, 81%).

Data for 26:
R_f = 0.51 (10:1, petroleum ether/EtOAc).
IR (neat): 2971, 2928, 1690, 1258, 1008 cm^{-1}.
HRMS (ESI): calculated for $C_{17}H_{31}O_2$ 267.2319 $[M+H]^+$, found 267.2318.
^{1}H NMR (500 MHz, $CDCl_3$): δ 4.94 (t, *J* = 7.5 Hz, 1H), 3.75 – 3.71 (m, 1H), 3.38 (d, *J* = 2.5 Hz, 1H), 2.67 (dd, *J* = 17.6, 1.7 Hz, 1H), 2.38 (dd, *J* = 17.7, 9.9 Hz, 1H), 2.17 (d, *J* = 7.5 Hz, 2H), 1.99 – 1.97 (m, 2H), 1.67 (s, 3H), 1.72 – 1.64 (overlapped m, 1H), 1.58 (s, 3H), 1.57 – 1.44 (m, 3H), 1.36 – 1.25 (m, 5H), 0.93 (d, *J* = 6.8 Hz, 3H), 0.91 (d, *J* = 6.7 Hz, 3H) ppm.
^{13}C NMR (125 MHz, $CDCl_3$): δ 218.0, 134.6, 118.8, 72.5, 53.0, 40.8, 37.6, 33.1, 33.1, 26.0, 23.2, 23.1, 18.6, 18.1, 18.0 ppm.

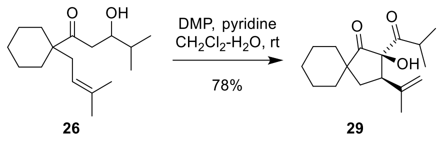

To a solution of **26** (50 mg, 0.19 mmol, 1.0 eq.) in CH_2Cl_2 (1 mL) was added pyridine (35 μL, 0.43 mmol, 2.3 eq.) and Dess-Martin periodinane (238 mg, 0.56 mmol, 3.0 eq.) and the mixture was stirred at room temperature for 24 h. Sat. aq. $NaHCO_3$ (10 mL) was added to the reaction mixture and it was extracted with CH_2Cl_2 (3 × 10 mL). The combined organic layers were dried over Na_2SO_4, filtered and concentrated *in vacuo*. The residue was purified by flash column chromatography on SiO_2 (100:1→20:1 gradient elution, petroleum ether/EtOAc) to give **29** as a white crystalline solid (40.8 mg, 0.15 mmol, 78%).

Data for 29:
R_f = 0.47 (10:1, petroleum ether/EtOAc).
Mp: 62 – 65 °C.
IR (neat): 2982, 2971, 1741, 1687, 1182, 1135 cm^{-1}.
HRMS (ESI): calculated for $C_{17}H_{27}O_3$ 279.1955 $[M+H]^+$, found 279.1955.
^{1}H NMR (500 MHz, $CDCl_3$): δ 5.02 (s, 1H), 4.89 (s, 1H), 3.64 (s, 1H), 3.30 (dd, *J* = 12.7, 6.4 Hz, 1H), 2.96 (sept, *J* = 6.7 Hz, 1H), 2.27 (dd, *J* = 12.7, 6.5 Hz, 1H), 1.99 (t, *J* = 12.7 Hz, 1H), 1.78 – 1.59 (overlapped m, 5H), 1.69 (s, 3H), 1.55 – 1.41 (m, 3H), 1.34 (m, 2H), 1.15 (d, *J* = 6.8 Hz, 3H), 1.08 (d, *J* = 6.7 Hz, 3H) ppm.
^{13}C NMR (125 MHz, $CDCl_3$): δ 216.4, 214.6, 141.8, 114.5, 87.3, 50.5, 47.6, 36.2, 35.2, 32.2, 25.6, 23.4, 22.4, 22.3, 19.2, 18.9 ppm.

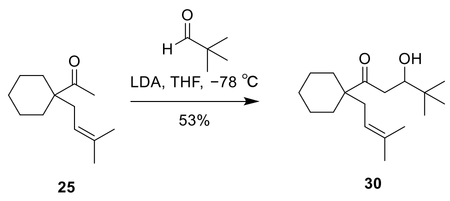

Diisopropylamine (0.23 mL, 1.65 mmol, 1.6 eq.) was dissolved in dry THF (6 mL) and cooled to 0 °C. *n*-BuLi (2.0 M in hexanes, 0.83 mL, 1.65 mmol, 1.6 eq.) was added and the mixture was stirred for 15 min. The mixture was cooled to -78 °C and **25** (200 mg, 1.03 mmol, 1.0 eq.) was added. After stirring for 20 min pivaldehyde (0.16 mL, 0.79 mmol, 1.4 eq.) was added and the reaction was stirred for 2 h. Sat. aq. NH_4Cl (10 mL) was added and the dry ice bath was removed to warm the mixture to room temperature. After extraction with Et_2O (3 × 10 mL) the combined organic layers were dried over Na_2SO_4, filtered and concentrated *in vacuo*. The residue was purified by flash column chromatography on SiO_2 (60:1, petroleum ether/EtOAc) to give **30** as a colourless oil (149 mg, 0.53 mmol, 53%).

Data for 30:
R_f = 0.49 (20:1, petroleum ether/EtOAc).
IR (neat): 2931, 1689, 1453, 1363, 1008 cm^{-1}.
HRMS (ESI): calculated for $C_{18}H_{33}O_2$ 281.2475 $[M+H]^+$, found 281.2476.
^{1}H NMR (500 MHz, $CDCl_3$): δ 4.95 (t, *J* = 7.5 Hz, 1H), 3.63 (dt, *J* = 10.5, 1.9 Hz, 1H), 3.35 (d, *J* = 2.4 Hz, 1H), 2.72 (dd, *J* = 17.5, 1.1 Hz, 1H), 2.31 (dd, *J* = 17.4, 10.3 Hz, 1H), 2.18 (d, *J* = 7.5 Hz, 2H), 2.01 – 1.98 (m, 2H), 1.68 (s, 3H), 1.58 (s, 3H), 1.57 – 1.44 (overlapped m, 3H), 1.38 – 1.23 (m, 5H), 0.91 (s, 9H) ppm.
^{13}C NMR (125 MHz, $CDCl_3$): δ 218.2, 134.6, 118.8, 75.2, 53.1, 38.9, 37.6, 34.3, 33.3, 33.1, 26.1, 26.1, 25.9, 25.9, 23.2, 18.1 ppm.

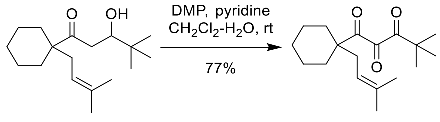

30 **31**

To a solution of **30** (50 mg, 0.18 mmol, 1.0 eq.) in CH_2Cl_2 (1 mL) was added pyridine (33 µL, 0.41 mmol, 2.3 eq.) and Dess-Martin periodinane (189 mg, 0.45 mmol, 2.5 eq.) and the mixture was stirred at room temperature for 3 h. The reaction was concentrated *in vacuo*. The residue was purified by flash column chromatography on SiO_2 (60:1, petroleum ether/EtOAc) to give **31** as an orange-yellow solid (40 mg, 0.14 mmol, 77%).

Data for 31:
R_f = 0.86 (20:1, petroleum ether/EtOAc).
Mp: 61 – 64 °C.
IR (neat): 2926, 2855, 2360, 2341,1712, 1456 cm^{-1}.
HRMS (ESI): calculated for $C_{18}H_{28}O_3K$ 331.1670 $[M+K]^+$, found 331.1706.
^1H NMR (500 MHz, $CDCl_3$): δ 5.01 – 4.94 (m, 1H), 2.47 (d, *J* = 7.6 Hz, 2H), 2.19 – 2.05 (m, 2H), 1.67 (s, 3H), 1.58 (s, 3H), 1.57 – 1.49 (m, 3H), 1.46 – 1.36 (m, 5H), 1.34 – 1.29 (m, 1H), 1.25 (s, 9H) ppm.
^{13}C NMR (125 MHz, $CDCl_3$): δ 210.0, 207.4, 187.4, 135.1, 118.6, 51.9, 43.0, 36.5, 32.4, 27.6, 26.0, 25.9, 22.8, 18.1 ppm.

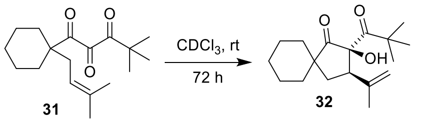

In an NMR tube **31** (40 mg, 0.14 mmol, 1.0 eq.) was dissolved in $CDCl_3$ (0.7 mL). After 72 h at room temperature full conversion was observed. Volatiles were removed *in vacuo* to yield **32** as a white solid (39 mg, 0.14 mmol, 98%).

Data for 32:
R_f = 0.59 (20:1, petroleum ether/EtOAc).
Mp: 77 – 81 °C.
IR (neat): 2932, 2360, 1743, 1680, 1449, 1092 cm^{-1}.
HRMS (ESI): calculated for $C_{18}H_{29}O_3$ 293.2111 $[M+H]^+$, found 293.2100.
^1H NMR (500 MHz, $CDCl_3$): δ 5.08 (s, 1H), 4.93 (s, 1H), 3.45 (dd, *J* = 12.8, 6.5 Hz, 1H), 3.37 (s, 1H), 2.16 (dd, *J* = 12.7, 6.5 Hz, 1H), 1.91 (t, *J* = 12.8 Hz, 1H), 1.72 (s, 3H), 1.71 – 1.57 (m, 4H), 1.56 – 1.27 (m, 6H), 1.23 (s, 9H) ppm.
^{13}C NMR (125 MHz, $CDCl_3$): δ 215.6, 215.5, 142.3, 114.7, 88.0, 50.0, 49.1, 44.7, 36.1, 35.9, 32.4, 27.0, 25.6, 24.1, 22.5, 22.4 ppm.

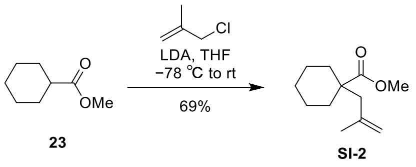

To a solution of diisopropylamine (1.52 mL, 10.8 mmol, 1.54 eq.) in dry THF (17 mL) at 0 °C was added *n*-BuLi (11.0 M in hexanes, 0.98 mL, 10.8 mmol, 1.54 eq.). The mixture was stirred for 5 min at 0 °C, then cooled to -78 °C. Ester **23** (1.00 g, 7.03 mmol, 1.00 eq.) was added and the mixture and stirred for 15 min at -78 °C, followed by addition of 3-chloro-2-methyl-1-propene (1.06 mL, 10.8 mmol, 1.54 eq.). The reaction was stirred for 5 min at -78 °C before the dry ice bath was removed and the reaction was stirred for another 1 h while slowly warming up to room temperature. Sat. aq. NH_4Cl solution (20 mL) was added and the mixture was extracted with Et_2O (3 × 20 mL). The combined organic layers were dried over Na_2SO_4, filtered and concentrated *in vacuo*. The residue was purified by flash column chromatography on SiO_2 (60:1→50:1 gradient elution, petroleum ether/EtOAc) to give **SI-2** as pale-yellow oil (956 mg, 4.87 mmol, 69%).

Data for SI-2:
R_f = 0.70 (20:1, petroleum ether/EtOAc).
IR (neat): 2930, 2855, 1728, 1452, 1192, 1129 cm^{-1}.
HRMS (ESI): calculated for $C_{12}H_{21}O_2$ 197.1536 $[M+H]^+$, found 197.1537.
^1H NMR (500 MHz, $CDCl_3$): δ 4.79 (s, 1H), 4.63 (s, 1H), 3.66 (s, 3H), 2.26 (s, 2H), 2.07 (m, 2H), 1.66 (s, 3H), 1.62 – 1.50 (m, 3H), 1.41 – 1.17 (m, 5H) ppm.
^{13}C NMR (125 MHz, $CDCl_3$): δ 177.3, 142.1, 114.4, 51.5, 48.7, 47.3, 34.8, 26.0, 23.9, 23.4 ppm.

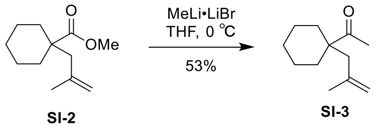

To a solution of **SI-2** (500 mg, 2.55 mmol, 1.00 eq.) in dry THF (3 mL) at 0 °C was added methyllithium lithium bromide complex (1.5 M in Et_2O, 3.96 mL, 5.94 mmol, 2.33 eq.). The mixture was stirred for 20 min at 0 °C. The reaction was quenched with sat. aq. NH_4Cl solution (20 mL) and extracted with Et_2O (3 × 10 mL). The combined organic layers were dried over Na_2SO_4, filtered and concentrated *in vacuo*. The residue was purified by flash column chromatography on SiO_2 (60:1→50:1 gradient elution, petroleum ether/EtOAc) to give **SI-3** as a colourless liquid (245 mg, 1.36 mmol, 53%).

Data for SI-3:
R_f = 0.59 (20:1, petroleum ether/EtOAc).
IR (neat): 2931, 2854, 1701, 1455, 1352, 1259, 1014 cm^{-1}.
HRMS (ESI): calculated for $C_{12}H_{21}O$ 181.1587 $[M+H]^+$, found 181.1586.
^1H NMR (500 MHz, $CDCl_3$): δ 4.80 (s, 1H), 4.62 (s, 1H), 2.26 (s, 2H), 2.15 (s, 3H), 2.01 (m, 2H), 1.65 (s, 3H), 1.61 – 1.44 (m, 3H), 1.30 (m, 5H) ppm.
^{13}C NMR (125 MHz, $CDCl_3$): δ 208.6, 136.8, 109.7, 47.5, 42.2, 28.9, 21.1, 19.4, 19.3, 18.1 ppm.

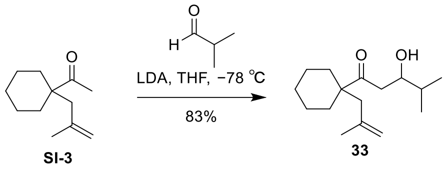

Diisopropylamine (0.27 mL, 1.95 mmol, 1.6 eq.) was dissolved in dry THF (7 mL) and cooled to 0 °C. *n*-BuLi (2.5 M in hexanes, 0.78 mL, 1.95 mmol, 1.6 eq.) was added and the mixture was stirred for 10 min. The mixture was cooled to -78 °C and **SI-3** (220 mg, 1.22 mmol, 1.0 eq.) was added. After stirring for 15 min isobutyrylaldehyde (0.16 mL, 1.71 mmol, 1.4 eq.) was added and the reaction was stirred for 2 h at -78 °C. Sat. aq. NH_4Cl (20 mL) and was added and the dry ice bath was removed to warm the mixture to room temperature. After extraction with Et_2O (2 × 10 mL) the combined organic layers were dried over Na_2SO_4, filtered and concentrated *in vacuo*. The residue was purified by flash column chromatography on SiO_2 (60:1→40:1 gradient elution, petroleum ether/EtOAc) to give **33** as a colourless oil (257 mg, 1.02 mmol, 83%).

Data for 33:
R_f = 0.44 (10:1, petroleum ether/EtOAc).
IR (neat): 2931, 1689, 1456, 1258, 1005 cm^{-1}.
HRMS (ESI): calculated for $C_{16}H_{29}O_2$ 253.2162 $[M+H]^+$, found 253.2162.
^{1}H NMR (500 MHz, $CDCl_3$): δ 4.81 (s, 1H), 4.62 (s, 1H), 3.77 (dd, *J* = 8.8, 6.5 Hz, 1H), 3.41 (d, *J* = 1.9 Hz, 1H), 2.76 (d, *J* = 17.8 Hz, 1H), 2.42 (dd, *J* = 17.8, 9.9 Hz, 1H), 2.27 (s, 2H), 2.08 – 1.92 (m, 2H), 1.73 – 1.66 (m, 1H), 1.65 (s, 3H), 1.62 – 1.45 (m, 3H), 1.44 – 1.27 (m, 5H), 0.94 (d, *J* = 6.8 Hz, 3H), 0.92 (d, *J* = 6.8 Hz, 3H) ppm.
^{13}C NMR (125 MHz, $CDCl_3$): δ 218.0, 141.8, 115.1, 72.6, 52.7, 47.2, 41.1, 34.1, 33.8, 33.2, 26.0, 24.7, 23.2, 23.1, 18.7, 18.1 ppm.

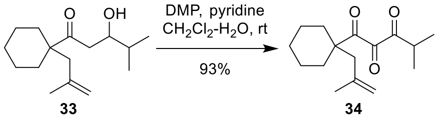

To a solution of **33** (100 mg, 0.40 mmol, 1.0 eq.) in CH_2Cl_2 (2 mL) was added Dess-Martin periodinane (420 mg, 0.99 mmol, 2.5 eq.) and pyridine (74 µL, 0.91 mmol, 2.3 eq.) and the mixture was stirred at room temperature for 1.5 h. Sat. aq. $NaHCO_3$ (10 mL) was added and the reaction mixture was extracted with CH_2Cl_2 (3 × 10 mL). The combined organic layers were dried over Na_2SO_4, filtered and concentrated *in vacuo*. The residue was purified by flash column chromatography on SiO_2 (60:1, petroleum ether/EtOAc) to give **34** as an orange oil (97 mg, 0.37 mmol, 93%).

Data for 34:
R_f = 0.85 (10:1, petroleum ether/EtOAc).
IR (neat): 2962, 2935, 2360, 1714, 1691, 1456, 1257, 1082, 1011 cm^{-1}.
HRMS (ESI): calculated for $C_{16}H_{24}O_3Na$ 287.1618 $[M+Na]^+$, found 287.1605.
^{1}H NMR (500 MHz, $CDCl_3$): δ 4.84 (s, 1H), 4.67 (s, 1H), 3.23 (sept, *J* = 6.9 Hz, 1H), 2.56 (s, 2H), 2.06 (m, 2H), 1.67 (s, 3H), 1.63 – 1.28 (m, 8H), 1.16 (d, *J* = 7.0 Hz, 6H) ppm.
^{13}C NMR (125 MHz, $CDCl_3$): δ 208.3, 205.0, 187.8, 141.4, 115.6, 51.1, 45.5, 35.9, 32.7, 25.8, 24.4, 22.6, 16.9 ppm.

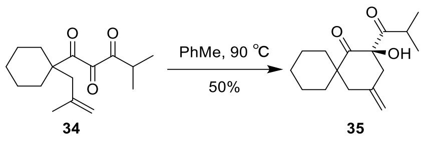

A solution of **34** (50 mg, 0.19 mmol, 1.0 eq.) in toluene (1 mL) was heated in a sealed vial at 90 °C for 3 h. The reaction was cooled down to room temperature and concentrated *in vacuo*. The residue was purified by flash column chromatography on SiO_2 (60:1→50:1 gradient elution, petroleum ether/EtOAc) to give **35** as a colourless oil (25 mg, 0.10 mmol, 50%).

Data for 35:
R_f = 0.54 (10:1, petroleum ether/EtOAc).
IR (neat): 2928, 2859, 2360, 1716, 1670, 1453, 1021 cm^{-1}.
HRMS (ESI): calculated for $C_{16}H_{24}O_3K$ 303.1357 $[M+K]^+$, found 303.1351.
^{1}H NMR (500 MHz, $CDCl_3$): δ 5.06 (s, 1H), 4.97 (s, 1H), 4.75 (s, 1H), 3.25 (d, *J* = 14.8 Hz, 1H), 2.86 (sept, *J* = 6.7 Hz, 1H), 2.54 (d, *J* = 14.1 Hz, 1H), 2.39 (d, *J* = 14.1 Hz, 1H), 2.33 (d, *J* = 14.8 Hz, 1H), 1.94 – 1.79 (m, 1H), 1.63 – 1.22 (m, 9H), 1.16 (d, *J* = 6.7 Hz, 3H), 1.02 (d, *J* = 6.7 Hz, 3H) ppm.
^{13}C NMR (125 MHz, $CDCl_3$): δ 213.0, 211.9, 138.5, 114.7, 84.8, 49.1, 42.3, 42.3, 35.7, 33.6, 32.9, 25.6, 21.5, 21.4, 20.7, 19.8 ppm.

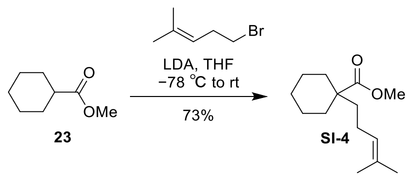

To a solution of diisopropylamine (1.52 mL, 10.8 mmol, 1.5 eq.) in dry THF (17 mL) at 0 °C was added *n*-BuLi (11.0 M in hexanes, 0.98 mL, 10.8 mmol, 1.5 eq.). The mixture was stirred for 5 min at 0 °C, then cooled to -78 °C. Ester **23** (1.00 g, 7.03 mmol, 1.0 eq.) was added and the mixture was stirred for 20 min at -78 °C, followed by addition of homoprenyl bromide (1.41 mL, 10.8 mmol, 1.5 eq.). The reaction was stirred for 10 min at -78 °C and then slowly warmed to room temperature and stirred for another 1.5 h. The reaction was quenched with sat. aq. NH_4Cl solution (100 mL) and extracted with Et_2O (3 × 20 mL). The combined organic layers were dried over Na_2SO_4, filtered and concentrated *in vacuo*. The residue was purified by flash column chromatography on SiO_2 (60:1, petroleum ether/EtOAc) to give **SI-4** as a colourless oil (1.16 g, 5.16 mmol, 73%).

Data for SI-4:
R_f = 0.54 (20:1, petroleum ether/EtOAc).
IR (neat): 2926, 2854, 1728, 1452, 1208, 1189, 1162, 1129 cm^{-1}.
HRMS (ESI): calculated for $C_{14}H_{24}O_2Na$ 247.1669 $[M+Na]^+$, found 247.1663.
^{1}H NMR (500 MHz, $CDCl_3$): δ 5.10 – 4.98 (m, 1H), 3.67 (s, 3H), 2.07 (d, *J* = 12.7 Hz, 2H), 1.85 (q, *J* = 7.5 Hz, 2H), 1.66 (s, 3H), 1.56 (s, 3H), 1.59 – 1.53 (overlapped m, 3H), 1.52 – 1.46 (m, 2H), 1.39 – 1.17 (m, 5H) ppm.
^{13}C NMR (125 MHz, $CDCl_3$): δ 177.4, 131.9, 124.2, 51.5, 47.1, 40.6, 34.3, 26.2, 25.8, 23.4, 23.0, 17.7 ppm.

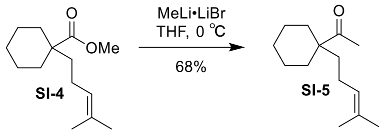

To a solution of **SI-4** (600 mg, 2.67 mmol, 1.00 eq.) in dry THF (4 mL) at 0 °C was added methyllithium lithium bromide complex (1.5 M in Et_2O, 4.2 mL, 6.23 mmol, 2.33 eq.). The mixture was stirred for 10 min at 0 °C. The reaction was quenched with sat. aq. NH_4Cl solution (10 mL) and extracted with Et_2O (3 × 10 mL). The combined organic layers were dried over Na_2SO_4, filtered and concentrated *in vacuo*. The residue was purified by flash column chromatography on SiO_2 (60:1→50:1 gradient elution, petroleum ether/EtOAc) to give **SI-5** as a colourless oil (378 mg, 1.81 mmol, 68%).

Data for SI-5:
R_f = 0.69 (20:1, petroleum ether/EtOAc).
IR (neat): 2928, 2854, 1701, 1456, 1352, 1155 cm^{-1}.
HRMS (ESI): calculated for $C_{14}H_{25}O$ 209.1900 $[M+H]^+$, found 209.1904.
^{1}H NMR (500 MHz, $CDCl_3$): δ 5.08 – 4.98 (m, 1H), 2.10 (s, 3H), 2.01 – 1.93 (m, 2H), 1.76 (q, *J* = 7.4 Hz, 2H), 1.66 (s, 3H), 1.55 (s, 3H), 1.57 – 1.48 (m, 5H), 1.29 (m, 5H) ppm.
^{13}C NMR (125 MHz, $CDCl_3$): δ 213.6, 132.1, 124.1, 52.2, 39.1, 33.5, 26.3, 25.8, 25.3, 23.1, 22.7, 17.7 ppm.

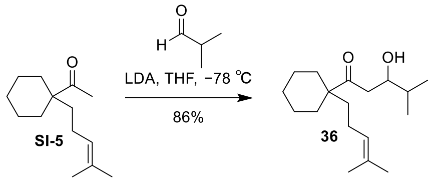

Diisopropylamine (0.32 mL, 2.30 mmol, 1.9 eq.) was dissolved in dry THF (10 mL) and cooled to 0 °C. *n*-BuLi (2.5 M in hexanes, 0.92 mL, 2.30 mmol, 1.9 eq.) was added and the mixture was stirred for 10 min. The mixture was cooled to -78 °C and **SI-5** (250 mg, 1.20 mmol, 1.0 eq.) was added. After stirring for 15 min isobutyrylaldehyde (0.18 mL, 2.02 mmol, 1.7 eq.) was added and the reaction was stirred for 90 min at -78 °C. Sat. aq. NH_4Cl (10 mL) and was added and the dry ice bath was removed to warm the mixture to room temperature. After extraction with Et_2O (3 × 10 mL) the combined organic layers were dried over Na_2SO_4, filtered and concentrated *in vacuo*. The residue was purified by flash column chromatography on SiO_2 (60:1→40:1 gradient elution, petroleum ether/EtOAc) to give **36** as a colourless oil (288 mg, 1.03 mmol, 86%).

Data for 36:
R_f = 0.42 (20:1, petroleum ether/EtOAc).
IR (neat): 2982, 2971, 2931, 2360, 1690, 1456, 1028 cm^{-1}.
HRMS (ESI): calculated for $C_{18}H_{31}O$ 263.2369 $[M-H_2O+H]^+$, found 263.2368.
^{1}H NMR (500 MHz, $CDCl_3$): δ 5.06 – 4.95 (m, 1H), 3.77 (ddd, *J* = 7.8, 5.7, 2.8 Hz, 1H), 3.36 (d, *J* = 2.8 Hz, 1H), 2.68 (dd, *J* = 17.6, 1.8 Hz, 1H), 2.39 (dd, *J* = 17.6, 9.8 Hz, 1H), 1.99 (d, *J* = 10.8 Hz, 2H), 1.82 – 1.77 (m, 2H), 1.72 – 1.67 (m, 1H), 1.66 (s, 3H), 1.55 (s, 3H), 1.58 – 1.47 (overlapped m, 5H), 1.37 – 1.23 (m, 5H), 0.94 (d, *J* = 6.8 Hz, 3H), 0.92 (d, *J* = 6.8 Hz, 3H) ppm.
^{13}C NMR (125 MHz, $CDCl_3$): δ 217.9, 132.2, 123.9, 72.6, 52.4, 40.4, 39.1, 33.4, 33.2, 26.2, 25.8, 23.0, 22.7, 18.6, 18.0, 17.7 ppm.

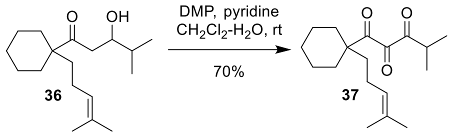

To a solution of **36** (100 mg, 0.36 mmol, 1.0 eq.) in CH_2Cl_2 (2 mL) was added Dess-Martin periodinane (378 mg, 0.89 mmol, 2.5 eq.) and pyridine (66 µL, 0.82 mmol, 2.3 eq.) and the mixture was stirred at room temperature for 4 h. Sat. aq. $NaHCO_3$ (10 mL) was added and the reaction mixture was extracted with CH_2Cl_2 (3 × 10 mL). The combined organic layers were dried over Na_2SO_4, filtered and concentrated *in vacuo*. The residue was purified by flash column chromatography on SiO_2 (60:1→40:1 gradient elution, petroleum ether/EtOAc) to give **37** as a orange oil (74 mg, 0.25 mmol, 70%).

Data for 37:
R_f = 0.48 (50:1, petroleum ether/EtOAc).
IR (neat): 2982, 2971, 2932, 2342, 1713, 1700, 1456 cm^{-1}.
HRMS (ESI): calculated for $C_{18}H_{28}O_3Na$ 315.1931 $[M+Na]^+$, found 315.1940.
^1H NMR (500 MHz, $CDCl_3$): δ 5.10 – 4.99 (m, 1H), 3.26 (sept, *J* = 7.0 Hz, 1H), 2.10 – 1.96 (m, 2H), 1.87 (dd, *J* = 10.4, 6.6 Hz, 2H), 1.75 – 1.69 (m, 2H), 1.66 (s, 3H), 1.58 (s, 3H), 1.56 – 1.28 (m, 8H), 1.17 (d, *J* = 7.0 Hz, 6H) ppm.
^{13}C NMR (125 MHz, $CDCl_3$): δ 209.3, 204.5, 188.0, 132.3, 123.8, 51.1, 37.6, 35.7, 32.4, 26.0, 25.8, 22.8, 22.6, 17.7, 17.0 ppm.

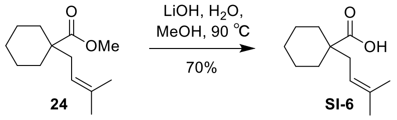

To a solution of **24** (1.62 g, 7.70 mmol, 1.0 eq.) in a mixture of H_2O (30 mL) and MeOH (30 mL) was added LiOH (1.84 g, 77.0 mmol, 10.0 eq.). The mixture was heated to 90 °C in a sealed vial for 3 h while being stirred. The reaction was cooled to room temperature and 1 M HCl (100 mL) was added. After extraction with Et_2O (3 × 50 mL) the combined organic layers were dried over Na_2SO_4, filtered and concentrated *in vacuo* to give **SI-6** as a white crystalline solid (1.05 g, 5.36 mmol, 70%).

Data for SI-6:
R_f = 0.48 (4:1, petroleum ether/EtOAc).
Mp: 99 – 102 °C.
IR (neat): 2931, 2855, 1690, 1455, 1248, 1191 cm^{-1}.
HRMS (ESI): calculated for $C_{12}H_{19}O_2$ 195.1391 $[M-H]^-$, found 195.1381.
^1H NMR (500 MHz, $CDCl_3$): δ 11.72 (bs, 1H), 5.12 (t, *J* = 7.6 Hz, 1H), 2.24 (d, *J* = 7.6 Hz, 2H), 2.05 (d, *J* = 13.1 Hz, 2H), 1.70 (s, 3H), 1.60 (s, 3H), 1.62 – 1.53 (overlapped m, 3H), 1.47 – 1.34 (m, 2H), 1.31 – 1.19 (m, 3H) ppm.
^{13}C NMR (125 MHz, $CDCl_3$): δ 183.5, 134.6, 119.1, 47.8, 38.8, 33.7, 26.1, 26.0, 23.4, 18.0 ppm.

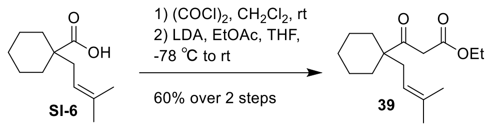

To a solution of **SI-6** (3.50 g, 17.8 mmol, 1.0 eq.) in CH_2Cl_2 (35 mL) was added oxalyl chloride (1.84 mL, 21.3 mmol, 1.2 eq.). The solution was stirred for 3 h at room temperature before volatiles were removed *in vacuo* to give the crude acid chloride which was directly used without further purification. In a separate flask diisopropylamine (4.00 mL, 28.5 mmol, 1.6 eq.) was dissolved in dry THF (10 mL) and cooled to 0 °C. *n*-BuLi (2.5 M in pentane, 11.4 mL, 28.5 mmol, 1.6 eq.) was added and the mixture was stirred for 10 min at 0 °C before it was cooled to -78 °C. EtOAc (2.63 mL, 26.7 mmol, 1.5 eq.) was added and the mixture was stirred for 15 min. The crude acid chloride was added as a solution in dry THF (15 mL) and the reaction was stirred for 30 min at -78 °C. The dry ice bath was removed and the reaction was stirred for another 1 h followed by addition of sat. aq. NH_4Cl (20 mL). The mixture was extracted with Et_2O (3 × 5 mL) and the combined organic phases were dried over Na_2SO_4, filtered and concentrated *in vacuo*. The residue was purified by flash column chromatography on SiO_2 (30:1→15:1 gradient elution, petroleum ether/EtOAc) to give **39** as a yellow oil (2.87 g, 10.8 mmol, 60% over 2 steps).

Data for 39:
R_f = 0.56 (10:1, petroleum ether/EtOAc).
IR (neat): 2931, 2856, 1744, 1703, 1449, 1299, 1222, 1041 cm^{-1}.
HRMS (ESI): calculated for $C_{16}H_{27}O_3$ 267.1955 $[M+H]^+$, found 267.1950.

Ketone:
^{1}H NMR (500 MHz, $CDCl_3$): δ 4.97 (t, *J* = 8.2 Hz, 1H), 4.19 (overlapped q, *J* = 7.1 Hz, 2H), 3.48 (s, 2H), 2.2 (overlapped d, *J* = 7.7 Hz, 2H), 1.97 – 1.94 (m, 2H), 1.68 (s, 3H), 1.60 (s, 3H), 1.59 – 1.50 (m, 3H), 1.35 – 1.29 (m, 5H), 1.27 (t, *J* = 7.1 Hz, 3H) ppm.
^{13}C NMR (125 MHz, $CDCl_3$): δ 207.63, 168.01, 134.87, 118.42, 88.83, 61.19, 53.11, 44.81, 32.95, 26.06, 25.98, 22.93, 18.06, 14.25 ppm.

Enol:
^{1}H NMR (500 MHz, $CDCl_3$): δ 12.43 (s, 1H), 5.05 – 5.02 (m, 2H), 4.20 (overlapped q, *J* = 6.9 Hz, 2H), 2.18 (overlapped d, *J* = 9.4 Hz, 2H), 1.85 – 1.82 (m, 2H), 1.57 (s, 3H), 1.56 (s, 3H), 1.59 – 1.55 (m, 3H), 1.35 – 1.33 (m, 5H), 1.31 (t, *J* = 7.2 Hz, 3H) ppm.
^{13}C NMR (125 MHz, $CDCl_3$): δ 183.7, 173.4, 133.9, 119.6, 107.5, 60.0, 44.6, 37.1, 33.0, 26.2, 26.1, 22.6, 18.0, 14.4 ppm.

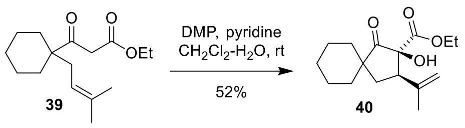

Dess-Martin periodinane (3.52 g, 8.31 mmol, 5.5 eq.) was dissolved in dry CH_2Cl_2 (30 mL). A solution of H_2O (84 µL) in dry CH_2Cl_2 (84 mL) was added dropwise over 30 min. Keto-ester **39** (400 mg, 1.50 mmol, 1.0 eq.) was added as a solution in dry CH_2Cl_2 (12 mL) and the reaction mixture was stirred for 16 h at room temperature. Volatiles were removed *in vacuo* and Et_2O (80 mL) was added to the residue. The suspension was washed with 10% aq. $Na_2S_2O_3$ (40 mL) and sat. aq. $NaHCO_3$ (40 mL) and the aq. phases were back extracted with Et_2O (2 × 40 mL). The combined organic phases were washed with brine (40 mL), dried over Na_2SO_4, filtered and concentrated *in vacuo*. The residue was purified by flash column chromatography on SiO_2 (25:1→20:1 gradient elution, petroleum ether/EtOAc) to give **40** as a pale-yellow oil (220 mg, 0.78 mmol, 52%).

Data for 40:
R_f = 0.31 (10:1, petroleum ether/EtOAc).
IR (neat): 2391, 2856, 1751, 1449, 1258, 1221, 1011 cm^{-1}.
HRMS (ESI): calculated for $C_{16}H_{22}O_3$ 263.1642 $[M-H_2O+H]^+$, found 263.1647.
^{1}H NMR (500 MHz, $CDCl_3$): δ 5.00 (s, 1H), 4.88 (s, 1H), 4.38 – 4.17 (m, 2H), 3.37 (s, 1H), 3.24 (dd, *J* = 13.1, 6.3 Hz, 1H), 2.22 (dd, *J* = 12.6, 6.4 Hz, 1H), 2.02 – 1.92 (t, *J* = 12.9 Hz, 1H), 1.77 – 1.71 (m, 2H), 1.74 (s, 3H), 1.70 – 1.57 (m, 3H), 1.55 – 1.41 (m, 3H), 1.38 – 1.32 (m, 2H), 1.29 (t, *J* = 7.1 Hz, 3H) ppm.
^{13}C NMR (125 MHz, $CDCl_3$): δ 215.3, 172.4, 141.6, 113.5, 81.6, 62.7, 50.2, 47.5, 35.3, 35.0, 32.1, 25.6, 22.8, 22.4, 22.2, 14.3 ppm.

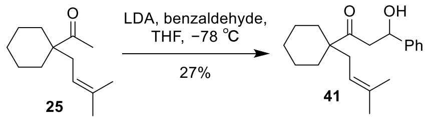

Diisopropylamine (0.20 mL, 1.44 mmol, 1.4 eq.) was dissolved in dry THF (6 mL) and cooled to 0 °C. *n*-BuLi (2.5 M in hexanes, 0.58 mL, 1.44 mmol, 1.4 eq.) was added and the mixture was stirred for 10 min. The mixture was cooled to -78 °C and **25** (200 mg, 1.03 mmol, 1.0 eq.) was added. After stirring for 25 min benzaldehyde (0.15 mL, 1.44 mmol, 1.4 eq.) was added and the reaction was stirred for 4 h. Sat. aq. NH_4Cl (20 mL) was added and the dry ice bath was removed to warm the mixture to room temperature. After extraction with Et_2O (3 × 10 mL) the combined organic layers were dried over Na_2SO_4, filtered and concentrated *in vacuo*. The residue was purified by flash column chromatography on SiO_2 (50:1→10:1 gradient elution, petroleum ether/EtOAc) to give **41** as a colourless oil (82.5 mg, 0.27 mmol, 27%).

Data for 41:
R_f = 0.36 (10:1, petroleum ether/EtOAc).
IR (neat): 2929, 2854, 1690, 1452, 1062, 1028 cm^{-1}.
HRMS (ESI): calculated for $C_{20}H_{28}O_2Na$ 323.1982 $[M+Na]^+$, found 323.1990.
^{1}H NMR (500 MHz, $CDCl_3$): δ 7.38 – 7.27 (m, 5H), 5.10 (d, *J* = 9.4 Hz, 1H), 4.96 – 4.84 (m, 1H), 3.76 (d, *J* = 2.7 Hz, 1H), 2.86 (dd, *J* = 17.9, 2.7 Hz, 1H), 2.76 (dd, *J* = 17.9, 9.4 Hz, 1H), 2.16 (d, *J* = 7.6 Hz, 2H), 2.03 – 1.93 (m, 2H), 1.68 (s, 3H), 1.61 – 1.48 (overlapped m, 3H), 1.59 (s, 3H), 1.38 – 1.23 (m, 5H) ppm.
^{13}C NMR (125 MHz, $CDCl_3$): δ 216.8, 143.3, 134.7, 128.6, 127.7, 125.8, 118.7, 70.3, 52.9, 46.5, 37.7, 33.1, 26.1, 26.0, 23.2, 18.1 ppm.

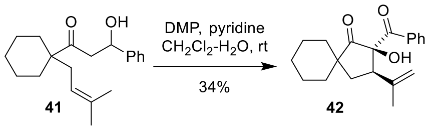

To a solution of **41** (50 mg, 0.166 mmol, 1.0 eq.) in CH_2Cl_2 (0.8 mL) was added pyridine (31 μL, 0.382 mmol, 2.3 eq.) and Dess-Martin periodinane (176 mg, 0.416 mmol, 2.5 eq.) and the mixture was stirred at room temperature for 5 h. Sat. aq. $NaHCO_3$ (10 mL) was added to the reaction mixture and it was extracted with CH_2Cl_2 (2 × 10 mL). The combined organic layers were dried over Na_2SO_4, filtered and concentrated *in vacuo*. The residue was purified by flash column chromatography on SiO_2 (25:1→15:1 gradient elution, petroleum ether/EtOAc) to give **42** as a pale-yellow solid (17.4 mg, 0.056 mmol, 34%).

Data for 42:
R_f = 0.45 (10:1, petroleum ether/EtOAc).
Mp: decomposition.
IR (neat): 2931, 2856, 1738, 1667, 1448, 1262, 1108 cm^{-1}.
HRMS (ESI): calculated for $C_{21}H_{25}O_3$ 313.1798 $[M+H]^+$, found 313.1803.
^{1}H NMR (500 MHz, $CDCl_3$): δ 7.72 (d, *J* = 7.7 Hz, 2H), 7.55 (t, *J* = 7.5 Hz, 1H), 7.44 (t, *J* = 7.8 Hz, 2H), 4.93 (s, 1H), 4.75 (s, 1H), 4.20 (s, 1H), 3.39 (dd, *J* = 12.7, 6.5 Hz, 1H), 2.27 (dd, *J* = 12.7, 6.5 Hz, 1H), 2.13 (t, *J* = 12.7 Hz, 1H), 1.82 – 1.64 (m, 6H), 1.60 (s, 3H), 1.52 – 1.22 (m, 4H).
^{13}C NMR (125 MHz, $CDCl_3$): δ 217.1, 201.5, 141.3, 135.1, 133.1, 129.3, 128.6, 114.5, 87.6, 50.6, 49.7, 36.01, 32.9, 25.5, 23.3, 23.3, 22.6, 22.4 ppm.

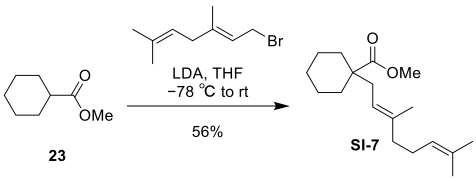

To a solution of diisopropylamine (2.22 mL, 15.8 mmol, 1.5 eq.) in dry THF (25 mL) at 0 °C was added *n*-BuLi (2.0 M in hexanes, 7.91 mL, 15.8 mmol, 1.5 eq.). The mixture was stirred for 10 min at 0 °C, then cooled to -78 °C. Ester **23** (1.50 g, 10.6 mmol, 1.0 eq.) was added and the mixture and stirred for 15 min at -78 °C, followed by addition of geranyl bromide (3.15 mL, 15.8 mmol, 1.5 eq.). The reaction was stirred for 10 min at -78 °C. The dry ice bath was removed to slowly warm the reaction to room temperature and it was stirred for another 1.5 h. The reaction was quenched with sat. aq. NH_4Cl solution (20 mL) and extracted with Et_2O (3 × 20 mL). The combined organic layers were dried over Na_2SO_4, filtered and concentrated *in vacuo*. The residue was purified by flash column chromatography on SiO_2 (100:0→50:1 gradient elution, petroleum ether/EtOAc) to give ester **SI-7** as a colourless oil (1.66 g, 5.95 mmol, 56%).

Data for SI-7:
R_f = 0.62 (20:1, petroleum ether/EtOAc).
IR (neat): 2935, 2859, 2360, 1701, 1449, 1375, 1134 cm^{-1}.
HRMS (ESI): calculated for $C_{18}H_{30}O_2K$ 317.1877 $[M+K]^+$, found 317.1883.
^{1}H NMR (500 MHz, $CDCl_3$): δ 5.09 – 5.03 (m, 2H), 3.65 (s, 3H), 2.19 (d, *J* = 7.6 Hz, 2H), 2.09 – 2.06 (m, 4H), 2.01 – 1.96 (m, 2H), 1.68 (s, 3H), 1.61 – 1.54 (overlapped m, 3H), 1.60 (s, 3H), 1.57 (s, 3H), 1.38 – 1.18 (m, 5H) ppm.
^{13}C NMR (125 MHz, $CDCl_3$): δ 177.3, 137.7, 131.5, 124.4, 119.4, 51.5, 48.0, 40.1, 38.8, 34.0, 26.8, 26.1, 25.8, 23.5, 17.8, 16.2 ppm.

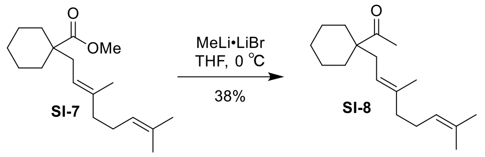

To a solution of **SI-7** (1.00 g, 3.59 mmol, 1.00 eq.) in dry THF (10 mL) at 0 °C was added methyllithium lithium bromide complex (1.5 M in Et_2O, 5.58 mL, 8.37 mmol, 2.33 eq.). The mixture was stirred for 10 min at 0 °C. The reaction was quenched with sat. aq. NH_4Cl solution (20 mL) and extracted with Et_2O (3 × 10 mL). The combined organic layers were dried over Na_2SO_4, filtered and concentrated *in vacuo*. The residue was purified by flash column chromatography on SiO_2 (60:1→50:1 gradient elution, petroleum ether/EtOAc) to give **SI-8** as a colourless liquid (354 mg, 1.35 mmol, 38%).

Data for SI-8:
R_f = 0.53 (20:1, petroleum ether/EtOAc).
IR (neat): 2932, 2858, 1697, 1449, 1375, 1074, 803 cm^{-1}.
HRMS (ESI): calculated for $C_{18}H_{30}OK$ 301.1928 $[M+K]^+$, found 301.1908.
^{1}H NMR (500 MHz, $CDCl_3$): δ 5.05 (m, 1H), 4.99 – 4.91 (m, 1H), 2.17 (d, *J* = 7.4 Hz, 2H), 2.09 (s, 3H), 2.07 – 2.02 (m, 2H), 1.97 (m, 4H), 1.67 (s, 3H), 1.58 (s, 3H), 1.58 (s, 3H), 1.52 (overlapped m, 3H), 1.30 (m, 5H) ppm.
^{13}C NMR (125 MHz, $CDCl_3$): δ 213.7, 137.9, 131.6, 124.3, 119.1, 52.8, 40.1, 37.3, 33.3, 26.7, 26.2, 25.8, 25.7, 23.2, 17.8, 16.3 ppm.

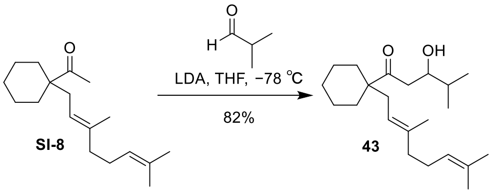

Diisopropylamine (0.22 mL, 1.58 mmol, 1.6 eq.) was dissolved in dry THF (8 mL) and cooled to 0 °C. *n*-BuLi (2.0 M in hexanes, 0.79 mL, 1.58 mmol, 1.6 eq.) was added and the mixture was stirred for 15 min. The mixture was cooled to -78 °C and **SI-8** (260 mg, 0.99 mmol, 1.0 eq.) was added. After stirring for 15 min isobutyrylaldehyde (0.13 mL, 1.39 mmol, 1.4 eq.) was added and the reaction was stirred for 3.5 h at -78 °C. Sat. aq. NH_4Cl (20 mL) and was added and the dry ice bath was removed to warm the mixture to room temperature. After extraction with Et_2O (3 × 10 mL) the combined organic layers were dried over Na_2SO_4, filtered and concentrated *in vacuo*. The residue was purified by flash column chromatography on SiO_2 (60:1→40:1 gradient elution, petroleum ether/EtOAc) to give **43** as a colourless oil (270 mg, 0.81 mmol, 82%).

Data for 43:
R_f = 0.53 (20:1, petroleum ether/EtOAc).
IR (neat): 3437, 2931, 2858, 1697, 1453, 1382, 1000, 950 cm^{-1}.
HRMS (ESI): calculated for $C_{22}H_{39}O_2$ 335.2945 $[M+H]^+$, found 335.2951.
^{1}H NMR (500 MHz, $CDCl_3$): δ 5.05 (t, *J* = 8.2 Hz, 1H), 4.95 (t, *J* = 7.4 Hz, 1H), 3.73 (m, 1H), 3.38 (d, *J* = 2.7 Hz, 1H), 2.66 (dd, *J* = 17.6, 1.8 Hz, 1H), 2.38 (dd, *J* = 17.6, 9.8 Hz, 1H), 2.18 (d, *J* = 7.4 Hz, 2H), 2.08 – 1.91 (m, 6H), 1.73 – 1.63 (overlapped m, 1H), 1.68 (s, 3H), 1.61 – 1.44 (overlapped m, 3H), 1.59 (s, 3H), 1.58 (s, 3H), 1.39 – 1.23 (m, 5H), 0.93 (d, *J* = 6.8 Hz, 3H), 0.91 (d, *J* = 6.8 Hz, 3H) ppm.
^{13}C NMR (125 MHz, $CDCl_3$): δ 217.9, 138.2, 131.7, 124.2, 118.8, 72.5, 53.0, 40.8, 40.1, 37.4, 33.1, 33.1, 33.0, 26.7, 26.1, 25.8, 23.2, 23.1, 18.6, 18.0, 17.9, 16.4 ppm.

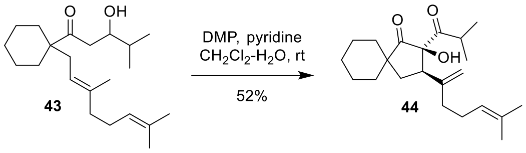

To a solution of **43** (100 mg, 0.30 mmol, 1.0 eq.) in CH_2Cl_2 (2 mL) was added Dess-Martin periodinane (317 mg, 0.75 mmol, 2.5 eq.) and pyridine (56 µL, 0.69 mmol, 2.3 eq.) and the mixture was stirred at room temperature for 4 h. Sat. aq. $NaHCO_3$ (10 mL) was added and the reaction mixture was extracted with CH_2Cl_2 (3 × 10 mL). The combined organic layers were dried over Na_2SO_4, filtered and concentrated *in vacuo*. The residue was purified by flash column chromatography on SiO_2 (60:1→40:1 gradient elution, petroleum ether/EtOAc) to give **44** as a colourless oil (54 mg, 0.16 mmol, 52%).

Data for 44:
R_f = 0.38 (10:1, petroleum ether/EtOAc).
IR (neat): 2978, 2932, 2869, 2360, 1743, 1701, 1449, 1382, 1142 cm^{-1}.
HRMS (ESI): calculated for $C_{22}H_{34}O_3Na$ 369.2400 $[M+Na]^+$, found 369.2401.
^{1}H NMR (500 MHz, $CDCl_3$): δ 5.09 – 5.02 (m, 2H), 4.98 (s, 1H), 3.44 (s, 1H), 3.36 (dd, *J* = 12.9, 6.3 Hz, 1H), 3.00 (sept, *J* = 6.8 Hz, 1H), 2.22 (dd, *J* = 12.7, 6.4 Hz, 1H), 2.18 – 2.06 (m, 2H), 1.99 – 1.92 (m, 2H), 1.90 – 1.84 (m, 1H), 1.78 – 1.55 (overlapped m, 5H), 1.67 (s, 3H), 1.60 (s, 3H), 1.48 (m, 3H), 1.38 – 1.29 (m, 2H), 1.13 (d, *J* = 6.8 Hz, 3H), 1.06 (d, *J* = 6.7 Hz, 3H) ppm.
^{13}C NMR (125 MHz, $CDCl_3$): δ 216.0, 215.1, 145.4, 132.3, 123.6, 113.4, 86.9, 50.4, 46.9, 36.3, 36.2, 36.2, 35.2, 31.9, 26.5, 25.8, 25.6, 22.4, 22.2, 18.9, 18.7, 17.9 ppm.

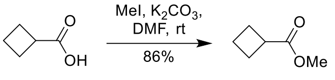

Cyclobutane carboxylic acid (**SI-9**, 2.00 g, 20.0 mmol, 1.0 eq.) was dissolved in DMF (40 mL) and K_2CO_3 (5.52 g, 40.0 mmol, 2.0 eq.) and MeI (2.49 mL, 40.0 mmol, 2.0 eq.) was added. The reaction was stirred at room temperature for 17 h. Sat. aq. NH_4Cl (100 mL) was added and the mixture was extracted with Et_2O (3 × 50 mL). The combined organic phases were washed with brine (100 mL), dried over Na_2SO_4, filtered and concentrated *in vacuo*. The product **SI-10** was obtained was obtained as a colourless oil (1.971 g, 17.3 mmol, 86%) without further purification. Data for **SI-10** matched that previously reported.[2]

Data for SI-10:
R_f = 0.41 (10:1, petroleum ether/EtOAc).
IR (neat): 2989, 2869, 1449, 1393, 1353, 1144, 1074 cm^{-1}.
^{1}H NMR (500 MHz, $CDCl_3$): δ 3.66 (s, 3H), 3.13 (p, *J* = 8.5 Hz, 1H), 2.28 (p, *J* = 9.0 Hz, 2H), 2.23 – 2.13 (m, 2H), 2.02 – 1.93 (m, 1H), 1.93 – 1.84 (m, 1H) ppm.
^{13}C NMR (125 MHz, $CDCl_3$): δ 176.1, 51.7, 38.1, 25.4, 18.6 ppm.

[2]R. I. Khusnutdinov, N. A. Shchadneva, Y. Y. Mayakova, *Russ. J. Gen. Chem.* **2018**, *88*, 15.

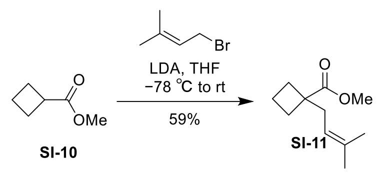

To a solution of diisopropylamine (2.36 mL, 16.8 mmol, 1.3 eq.) in dry THF (15 mL) at 0 °C was added *n*-BuLi (2.0 M in hexanes, 8.4 mL, 16.8 mmol, 1.3 eq.). The mixture was stirred for 10 min at 0 °C, then cooled to -78 °C. Ester **SI-10** (1.44 g, 12.6 mmol, 1.0 eq.) was added as solution in dry THF (15 mL) and the mixture was stirred for 20 min at -78 °C, followed by addition of prenyl bromide (1.94 mL, 16.8 mmol, 1.3 eq.). The reaction was stirred for 10 min at -78 °C before the dry ice bath was removed and the reaction was stirred for another 2 h while slowly warming to room temperature. The reaction was quenched with sat. aq. NH_4Cl solution (20 mL). After extraction with Et_2O (3 × 50 mL) the combined organic layers were dried over Na_2SO_4, filtered and concentrated *in vacuo*. The residue was purified by flash column chromatography on SiO_2 (100:0→60:1 gradient elution, petroleum ether/EtOAc) to give **SI-11** as a pale-yellow oil (1.36 g, 7.45 mmol, 59%).

Data for SI-11:
R_f = 0.66 (20:1, petroleum ether/EtOAc).
IR (neat): 2989, 2869, 1731, 1449, 1392, 1144, 1087 cm^{-1}.
HRMS (ESI): calculated for $C_{11}H_{19}O_2$ 183.1380 $[M+H]^+$, found 183.1388.
^{1}H NMR (500 MHz, $CDCl_3$): δ 5.07 – 4.98 (m, 1H), 3.66 (s, 3H), 2.45 (d, *J* = 7.3 Hz, 2H), 2.40 – 2.35 (m, 2H), 1.95 – 1.79 (m, 4H), 1.68 (s, 3H), 1.62 (s, 3H) ppm.
^{13}C NMR (125 MHz, $CDCl_3$): δ 177.7, 134.3, 119.6, 51.8, 47.8, 36.1, 29.6, 26.0, 18.0, 15.6 ppm.

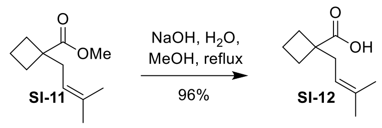

To a solution of **SI-11** (600 mg, 3.29 mmol, 1.0 eq.) in MeOH (13 mL) and H_2O (13 mL) NaOH (156 mg, 3.95 mmol, 1.2 eq.) was added and the reaction was heated to reflux for 7 h. The reaction was cooled to room temperature and 1 M HCl (50 mL) was added. After extraction with Et_2O (3 × 20 mL) the combined org. phases were dried over Na_2SO_4, filtered and concentrated *in vacuo* to give **SI-12** as a colourless oil (531 mg, 3.16 mmol, 96%).

Data for SI-12:
R_f = 0.45 (4:1, petroleum ether/EtOAc).
IR (neat): 2989, 2869, 1701, 1393, 1144, 1074, 943 cm^{-1}.
HRMS (ESI): calculated for $C_{10}H_{17}O_2$ 169.1223 $[M+H]^+$, found 169.1234.
^{1}H NMR (500 MHz, $CDCl_3$): δ 11.60 (s, 1H), 5.09 (t, *J* = 7.3 Hz, 1H), 2.49 (d, *J* = 7.3 Hz, 2H), 2.47 – 2.39 (m, 2H), 1.99 – 1.85 (m, 4H), 1.71 (s, 3H), 1.65 (s, 3H) ppm.
^{13}C NMR (125 MHz, $CDCl_3$): δ 183.7, 134.7, 119.4, 47.6, 35.8, 29.5, 26.1, 18.1, 15.6 ppm.

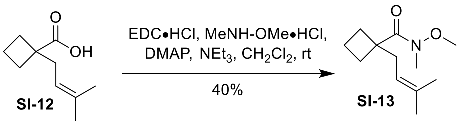

To a solution of **SI-12** (450 mg, 2.67 mmol, 1.00 eq.) in CH_2Cl_2 (10 mL) was added 1-ethyl-3-(3-dimethylaminopropyl)-carbodiimid hydrochloride (590 mg, 3.08 mmol, 1.15 eq.), N,O-dimethylhydroxylamine hydrochloride (300 mg,3.08 mmol, 1.15 eq.), DMAP (16 mg, 0.13 mmol, 0.05 eq.) and triethylamine (1.12 mL, 8.02 mmol, 3.00 eq.) and the reaction was stirred at room temperature for 16 h. 1 M HCl (50 mL) was added and the mixture was extracted with CH_2Cl_2 (2 × 20 mL). The combined org. phases were washed with sat. aq. $NaHCO_3$ (50 mL), dried over Na_2SO_4, filtered and concentrated *in vacuo*. The residue was purified by flash column chromatography on SiO_2 (10:1→5:1 gradient elution, petroleum ether/EtOAc) to give **SI-13** as a colourless oil (224 g, 1.06 mmol, 40%).

Data for SI-13:
R_f = 0.47 (4:1, petroleum ether/EtOAc).
IR (neat): 2989, 2982, 2869, 1392, 1349, 1144, 1074 cm^{-1}.
HRMS (ESI): calculated for $C_{12}H_{21}O_2NNa$ 234.1465 $[M+Na]^+$, found 234.1465.
^{1}H NMR (500 MHz, $CDCl_3$): δ 5.10 (t, *J* = 6.2 Hz, 1H), 3.63 (s, 3H), 3.13 (s, 3H), 2.52 (d, *J* = 7.3 Hz, 2H), 2.46 – 2.35 (m, 2H), 1.98 – 1.83 (m, 3H), 1.75 – 1.70 (m, 1H), 1.69 (s, 3H), 1.62 (s, 3H) ppm.
^{13}C NMR (125 MHz, $CDCl_3$): δ 181.1, 134.0, 119.8, 60.7, 48.3, 35.8, 34.4, 30.0, 26.1, 18.0, 15.4 ppm.

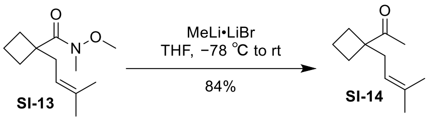

To a solution of **SI-13** (195 mg, 0.92 mmol, 1.0 eq.) in dry THF (3.5 mL) at -78 °C methyllithium lithium bromide complex (1.5 M in Et_2O, 1.23 mL, 1.85 mmol, 2.0 eq.) was added and the reaction was stirred for 20 min. Sat. aq. NH_4Cl (10 mL) was added and the dry ice bath was removed to warm the mixture to room temperature. After extraction with Et_2O (3 × 10 mL) the combined organic layers were dried over Na_2SO_4, filtered and concentrated *in vacuo*. The residue was purified by flash column chromatography on SiO_2 (50:1, petroleum ether/EtOAc) to give **SI-14** as a colourless oil (129 mg, 0.78 mmol, 84%).

Data for SI-14:
R_f = 0.52 (10:1, petroleum ether/EtOAc).
IR (neat): 2989, 2982, 2869, 1392, 1259, 1142, 1017, 796 cm^{-1}.
HRMS (ESI): calculated for $C_{11}H_{18}ONa$ 189.1250 $[M+Na]^+$, found 189.1243.
^{1}H NMR (500 MHz, $CDCl_3$): δ 4.93 (m, 1H), 2.47 (d, *J* = 7.2 Hz, 2H), 2.38 – 2.27 (m, 2H), 2.04 (s, 3H), 1.94 – 1.69 (m, 4H), 1.68 (s, 3H), 1.63 (s, 3H) ppm.
^{13}C NMR (125 MHz, $CDCl_3$): δ 212.4, 134.5, 119.2, 54.2, 35.9, 28.5, 26.0, 24.4, 18.1, 14.8 ppm.

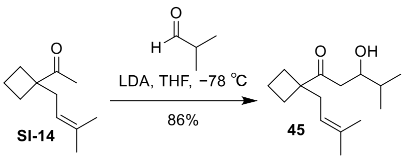

Diisopropylamine (0.13 mL, 0.89 mmol, 1.6 eq.) was dissolved in dry THF (5 mL) and cooled to 0 °C. n-BuLi (2.0 M in hexanes, 0.45 mL, 0.89 mmol, 1.6 eq.) was added and the mixture was stirred for 10 min. The mixture was cooled to -78 °C and **SI-14** (93 mg, 0.56 mmol, 1.0 eq.) was added. After stirring for 30 min isobutyrylaldehyde (71 µL, 0.78 mmol, 1.4 eq.) was added and the reaction was stirred for 2 h. Sat. aq. NH_4Cl (10 mL) was added and the dry ice bath was removed to warm the mixture to room temperature. After extraction with Et_2O (3 × 10 mL) the combined organic layers were dried over Na_2SO_4, filtered and concentrated *in vacuo*. The residue was purified by flash column chromatography on SiO_2 (50:1→15:1 gradient elution, petroleum ether/EtOAc) to give **45** as a colourless oil (114 mg, 0.48 mmol, 86%).

Data for 45:
R_f = 0.40 (10:1, petroleum ether/EtOAc).
IR (neat): 3532, 2959, 2934, 2912, 1687, 1470, 1383 cm^{-1}.
HRMS (ESI): calculated for $C_{15}H_{27}O_2$ 239.2006 $[M+H]^+$, found 239.2005.
^{1}H NMR (500 MHz, $CDCl_3$): δ 4.94 – 4.91 (m, 1H), 3.79 – 3.71 (m, 1H), 3.26 (d, *J* = 3.0 Hz, 1H), 2.55 (dd, *J* = 17.4, 2.1 Hz, 1H), 2.48 (d, *J* = 7.2 Hz, 2H), 2.42 – 2.30 (m, 3H), 1.99 – 1.88 (m, 1H), 1.89 – 1.80 (m, 2H), 1.79 – 1.71 (m, 1H), 1.70 – 1.66 (m, 1H), 1.68 (s, 3H), 1.63 (s, 3H), 0.93 (d, *J* = 6.8 Hz, 3H), 0.91 (d, *J* = 6.9 Hz, 3H) ppm.
^{13}C NMR (125 MHz, $CDCl_3$): δ 216.4, 134.7, 119.0, 72.6, 54.2, 39.9, 35.9, 33.2, 28.6, 28.5, 26.0, 18.6, 18.2, 18.0, 14.9 ppm.

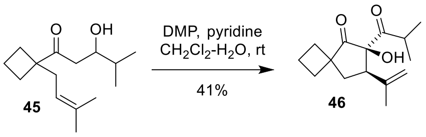

To a solution of **45** (32 mg, 0.13 mmol, 1.0 eq.) in CH_2Cl_2 (0.8 mL) was added pyridine (25 µL, 0.31 mmol, 2.3 eq.) and Dess-Martin periodinane (143 mg, 0.34 mmol, 2.5 eq.) and the mixture was stirred at room temperature for 5 h. Sat. aq. $NaHCO_3$ (10 mL) was added and the reaction mixture was extracted with CH_2Cl_2 (3 × 10 mL). The combined organic layers were dried over Na_2SO_4, filtered and concentrated *in vacuo*. The residue was purified by flash column chromatography on SiO_2 (40:1→20:1 gradient elution, petroleum ether/EtOAc) to give **46** as a yellow oil (14 mg, 0.056 mmol, 41%).

Data for 46:
R_f = 0.49 (10:1, petroleum ether/EtOAc).
IR (neat): 2961, 2936, 2926, 2363, 1700, 1446, 1369, 1101, 1023 cm^{-1}.
HRMS (ESI): calculated for $C_{15}H_{22}O_3K$ 289.1201 $[M+K]^+$, found 289.1251.
^{1}H NMR (500 MHz, $CDCl_3$): δ 5.00 (s, 1H), 4.83 (s, 1H), 3.61 (s, 1H), 3.19 (dd, *J* = 12.1, 5.8 Hz, 1H), 2.97 (sept, *J* = 6.8 Hz, 1H), 2.53 (m, 1H), 2.37 (dd, *J* = 12.5, 6.1 Hz, 1H), 2.32 (m, 1H), 2.20 (t, *J* = 12.2 Hz, 1H), 2.11 – 1.99 (m, 3H), 1.95 (m, 1H), 1.65 (s, 3H), 1.16 (d, *J* = 6.7 Hz, 3H), 1.09 (d, *J* = 6.8 Hz, 3H) ppm.
^{13}C NMR (125 MHz, $CDCl_3$): δ 214.4, 214.3, 141.7, 114.3, 86.6, 51.0, 47.9, 38.4, 36.1, 34.4, 27.4, 23.2, 19.4, 18.9, 16.0 ppm.

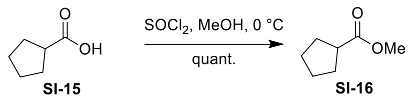

Cyclopentane carboxylic acid (**SI-15**, 2.00 g, 17.5 mmol, 1.0 eq.) was dissolved in MeOH (20 mL) and cooled to 0 °C. $SOCl_2$ (1.53 mL, 21.0 mmol, 1.2 eq.) was added dropwise and the mixture was stirred at 0 °C for 3 h. Sat. aq. $NaHCO_3$ (100 mL) was added followed by extraction with Et_2O (3 × 50 mL). The combined organic layers were washed with brine (100 mL), dried over Na_2SO_4 and volatiles were removed *in vacuo* to give **SI-16** as a colourless oil (2.40 g, 17.5 mmol, quant.). Data for **SI-16** matched that previously reported.[3]

Data for SI-16:
R_f = 0.35 (20:1, petroleum ether/EtOAc).
IR (neat): 2953, 2872, 1734, 1701, 1197, 1158 cm^{-1}.
^{1}H NMR (500 MHz, $CDCl_3$): δ 3.66 (s, 3H), 2.72 (quin, J = 8.0 Hz, 1H), 1.94 – 1.83 (m, 2H), 1.83 – 1.74 (m, 2H), 1.74 – 1.64 (m, 2H), 1.61 – 1.51 (m, 2H) ppm.
^{13}C NMR (125 MHz, $CDCl_3$): δ 177.4, 51.7, 43.8, 30.2, 25.9 ppm.

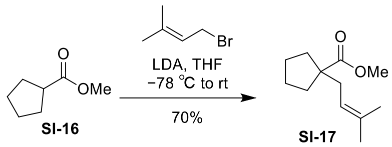

To a solution of diisopropylamine (3.75 mL, 26.7 mmol, 1.5 eq.) in dry THF (50 mL) at 0 °C was added *n*-BuLi (2.0 M in hexanes, 13.4 mL, 26.7 mmol, 1.5 eq.). The mixture was stirred for 10 min at 0 °C, then cooled to -78 °C. Ester **SI-16** (2.28 g, 17.8 mmol, 1.0 eq.) was added and the mixture and stirred for 20 min at -78 °C, followed by addition of prenyl bromide (3.08 mL, 26.7 mmol, 1.5 eq.). The reaction was then slowly warmed to room temperature and stirred for 30 min. The reaction was quenched with sat. aq. NH_4Cl solution (100 mL). The organic layer was separated and the aqueous layer was extracted with Et_2O (2 × 50 mL). The combined organic layers were dried over Na_2SO_4, filtered and concentrated *in vacuo*. The residue was purified by flash column chromatography on SiO_2 (60:1→50:1 gradient elution, petroleum ether/EtOAc) to give ester **SI-17** as a colourless oil (2.44 g, 12.4 mmol, 70%).

Data for SI-17:
R_f = 0.64 (20:1, petroleum ether/EtOAc).
IR (neat): 2971, 1706, 1378, 1239, 1154 cm^{-1}.
HRMS (ESI): calculated for $C_{12}H_{21}O_2$ 197.1536 $[M+H]^+$, found 197.1538.
^{1}H NMR (500 MHz, $CDCl_3$): δ 5.05 – 5.01 (m, 1H), 3.65 (s, 3H), 2.30 (d, J = 7.4 Hz, 2H), 2.10 – 1.99 (m, 2H), 1.68 (s, 3H), 1.64 – 1.60 (m, 4H), 1.59 (s, 3H), 1.54 – 1.49 (m, 2H) ppm.
^{13}C NMR (125 MHz, $CDCl_3$): δ 178.4, 133.9, 120.7, 54.4, 51.8, 37.1, 35.6, 26.1, 25.2, 18.0 ppm.

[3] X. Zhang, C. Shen, C. Xia, X. Tian, L. He, *Green Chem.* **2018**, *20*, 5533.

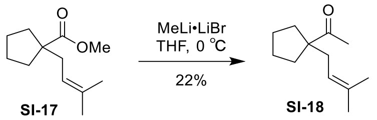

To a solution of **SI-17** (1.00 g, 5.09 mmol, 1.0 eq.) in dry THF (10 mL) at 0 °C was added methyllithium lithium bromide complex (1.5 M in Et_2O, 7.91 mL, 11.9 mmol, 2.33 eq.). The mixture was stirred for 5 min at 0 °C. The reaction was quenched with sat. aq. NH_4Cl solution (10 mL) and extracted with Et_2O (3 × 10 mL). The combined organic layers were dried over Na_2SO_4, filtered and concentrated *in vacuo*. The residue was purified by flash column chromatography on SiO_2 (50:1, petroleum ether/EtOAc) to give **SI-18** as a colourless liquid (202 mg, 1.12 mmol, 22%).

Data for SI-18:
R_f = 0.50 (20:1, petroleum ether/EtOAc).
IR (neat): 2938, 2925, 1704, 1446, 1368, 1100, 978 cm^{-1}.
HRMS (ESI): calculated for $C_{12}H_{20}ONa$ 203.1406 $[M+Na]^+$, found 203.1408.
^{1}H NMR (500 MHz, $CDCl_3$): δ 5.06 – 4.53 (m, 1H), 2.32 (d, *J* = 7.1 Hz, 2H), 2.12 (s, 3H), 2.03 – 1.97 (m, 2H), 1.67 (s, 3H), 1.66 – 1.52 (overlapped m, 4H), 1.61 (s, 3H), 1.50 – 1.41 (m, 2H) ppm.
^{13}C NMR (125 MHz, $CDCl_3$): δ 212.7, 134.0, 120.4, 60.5, 36.9, 34.3, 26.0, 25.9, 25.3, 18.1 ppm.

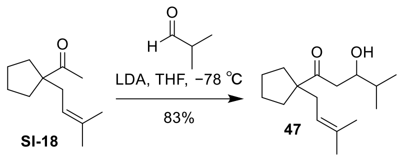

Diisopropylamine (0.19 mL, 1.33 mmol, 1.6 eq.) was dissolved in dry THF (6 mL) and cooled to 0 °C. *n*-BuLi (2.5 M in hexanes, 0.53 mL, 1.3 mmol, 1.6 eq.) was added and the mixture was stirred for 10 min. The mixture was cooled to -78 °C and **SI-18** (150 mg, 0.83 mmol, 1.0 eq.) was added. After stirring for 25 min isobutyrylaldehyde (0.11 mL, 1.16 mmol, 1.4 eq.) was added and the reaction was stirred for 2.5 h. Sat. aq. NH_4Cl (10 mL) was added and the dry ice bath was removed to warm the mixture to room temperature. After extraction with Et_2O (3 × 10 mL) the combined organic layers were dried over Na_2SO_4, filtered and concentrated *in vacuo*. The residue was purified by flash column chromatography on SiO_2 (50:1→20:1 gradient elution, petroleum ether/EtOAc) to give **47** as a colourless oil (173 mg, 0.69 mmol, 83%).

Data for 47:
R_f = 0.54 (10:1, petroleum ether/EtOAc).
IR (neat): 2961, 1690, 1410, 13378, 1259, 1013, 793 cm^{-1}.
HRMS (ESI): calculated for $C_{16}H_{29}O_2$ 253.2162 $[M+H]^+$, found 253.2165.
^{1}H NMR (500 MHz, $CDCl_3$): δ 4.93 (t, *J* = 7.1 Hz, 1H), 3.80 – 3.66 (m, 1H), 3.27 (d, *J* = 2.8 Hz, 1H), 2.66 (dd, *J* = 17.3, 1.5 Hz, 1H), 2.44 (dd, *J* = 17.3, 9.8 Hz, 1H), 2.32 (d, *J* = 7.1 Hz, 2H), 2.08 – 1.93 (m, 2H), 1.73 – 1.63 (overlapped m, 3H), 1.67 (s, 3H), 1.63 – 1.55 (overlapped m, 2H), 1.60 (s, 3H), 1.53 – 1.43 (m, 2H), 0.94 (d, *J* = 6.8 Hz, 3H), 0.91 (d, *J* = 6.8 Hz, 3H) ppm.
^{13}C NMR (125 MHz, $CDCl_3$): δ 216.9, 134.3, 120.2, 72.8, 60.7, 41.4, 36.8, 34.3, 34.2, 33.2, 26.0, 25.2, 18.6, 18.2, 18.0 ppm.

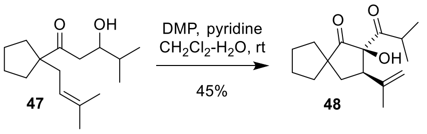

To a solution of **47** (60 mg, 0.24 mmol, 1.0 eq.) in CH_2Cl_2 (1.5 mL) was added Dess-Martin periodinane (252 mg, 0.60 mmol, 2.5 eq.) and pyridine (44 µL, 0.55 mmol, 2.3 eq.) and the mixture was stirred at room temperature for 2 h. Sat. aq. $NaHCO_3$ (10 mL) was added and the reaction mixture was extracted with CH_2Cl_2 (3 × 10 mL). The combined organic layers were dried over Na_2SO_4, filtered and concentrated *in vacuo*. The residue was purified by flash column chromatography on SiO_2 (50:1→25:1 gradient elution, petroleum ether/EtOAc) to give **48** as a colourless oil (28 mg, 0.106 mmol, 45%).

Data for 48:
R_f = 0.52 (10:1, petroleum ether/EtOAc).
IR (neat): 2945, 2872, 2349, 1738, 1700, 1452, 1097 cm^{-1}.
HRMS (ESI): calculated for $C_{16}H_{25}O_3$ 287.1608 $[M+H]^+$, found 287.1618.
^{1}H NMR (500 MHz, $CDCl_3$): δ 5.01 (m, 1H), 4.85 (m, 1H), 3.60 (s, 1H), 3.30 (dd, *J* = 12.4, 6.5 Hz, 1H), 3.02 (sept, *J* = 6.7 Hz, 1H), 2.17 – 2.01 (m, 3H), 1.88 – 1.67 (overlapped m, 5H), 1.67 (s, 3H), 1.65 – 1.57 (m, 2H), 1.16 (d, *J* = 6.8 Hz, 3H), 1.08 (d, *J* = 6.8 Hz, 3H) ppm.
^{13}C NMR (125 MHz, $CDCl_3$): δ 217.1, 214.7, 141.7, 114.4, 86.5, 56.8, 48.6, 39.7, 39.2, 37.1, 36.1, 26.1, 26.1 23.3, 19.3, 18.9 ppm.

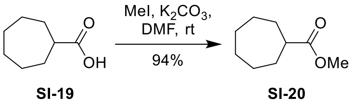

Cycloheptane carboxylic acid (**SI-19**, 3.00 g, 21.1 mmol, 1.0 eq.) was dissolved in DMF (40 mL) and K_2CO_3 (5.83 g, 42.2 mmol, 2.0 eq.) and MeI (2.63 mL, 42.2 mmol, 2.0 eq.) was added. The reaction was stirred at room temperature for 17 h. Sat. aq. NH_4Cl (100 mL) was added and the mixture was extracted with Et_2O (3 × 50 mL). The combined organic phases were dried over Na_2SO_4, filtered and concentrated *in vacuo*. The product **SI-20** was obtained was obtained as a colourless oil (3.09 g, 19.8 mmol, 94%) without further purification. Data for **SI-20** matched that previously reported.[3]

Data for SI-20:
R_f = 0.58 (20:1, petroleum ether/EtOAc).
IR (neat): 2921, 2856, 1736, 1433, 1316, 1194, 1155 cm^{-1}.
^{1}H NMR (500 MHz, $CDCl_3$): δ 3.65 (s, 3H), 2.55 – 2.43 (m, 1H), 1.96 – 1.86 (m, 2H), 1.74 – 1.63 (m, 4H), 1.60 – 1.41 (m, 6H) ppm.
^{13}C NMR (125 MHz, $CDCl_3$): δ 177.7, 51.6, 45.1, 31.0, 28.4, 26.5 ppm.

[3] X. Zhang, C. Shen, C. Xia, X. Tian, L. He, *Green Chem.* **2018**, *20*, 5533.

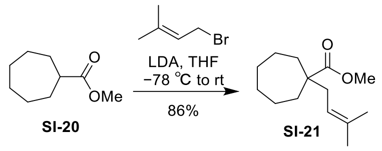

To a solution of diisopropylamine (2.52 mL, 17.9 mmol, 1.4 eq.) in dry THF (15 mL) at 0 °C was added *n*-BuLi (2.0 M in hexanes, 8.96 mL, 17.9 mmol, 1.4 eq.). The mixture was stirred for 10 min at 0 °C, then cooled to -78 °C. Ester **SI-20** (2.00 g, 12.8 mmol, 1.0 eq.) was added and the mixture and stirred for 20 min at -78 °C, followed by addition of prenyl bromide (1.77 mL, 15.4 mmol, 1.2 eq.). The reaction was stirred for 10 min at -78 °C before the dry ice bath was removed and the reaction was stirred for another 1 h while slowly warming up to room temperature. Sat. aq. NH_4Cl solution (100 mL) was added and the mixture was extracted with Et_2O (3 × 50 mL). The combined organic layers were dried over Na_2SO_4, filtered and concentrated *in vacuo*. The residue was purified by flash column chromatography on SiO_2 (60:1, petroleum ether/EtOAc) to give ester **SI-21** as pale-yellow oil (2.47 g, 11.0 mmol, 86%).

Data for SI-21:
R_f = 0.53 (20:1, petroleum ether/EtOAc).
IR (neat): 2919, 2855, 1731, 1460, 1261, 1192, 1165, 1058, 804 cm^{-1}.
HRMS (ESI): calculated for $C_{14}H_{25}O_2$ 225.1849 $[M+H]^+$, found 225.1888.
^{1}H NMR (500 MHz, $CDCl_3$): δ 4.99 (t, *J* = 7.5 Hz, 1H), 3.65 (s, 3H), 2.20 (d, *J* = 7.5 Hz, 2H), 2.08 – 2.03 (m, 2H), 1.67 (s, 3H), 1.58 (s, 3H), 1.49 (m, 10H) ppm.
^{13}C NMR (125 MHz, $CDCl_3$): δ 178.3, 134.1, 120.1, 51.6, 50.6, 39.3, 36.1, 30.2, 26.1, 23.8, 18.0 ppm.

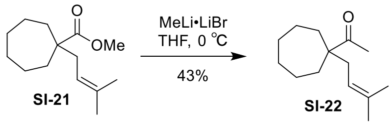

To a solution of **SI-21** (1.00 g, 4.46 mmol, 1.0 eq.) in dry THF (15 mL) at 0 °C was added methyllithium lithium bromide complex (1.5 M in Et_2O, 6.84 mL, 10.3 mmol, 2.33 eq.). The mixture was stirred for 10 min at 0 °C. The reaction was quenched with sat. aq. NH_4Cl solution (20 mL) and extracted with Et_2O (3 × 20 mL). The combined organic layers were dried over Na_2SO_4, filtered and concentrated *in vacuo*. The residue was purified by flash column chromatography on SiO_2 (60:1, petroleum ether/EtOAc) to give **SI-22** as a colourless liquid (395 mg, 1.90 mmol, 43%).

Data for SI-22:
R_f = 0.55 (20:1, petroleum ether/EtOAc).
IR (neat): 2921, 2856, 1704, 1460, 1378, 1151 cm^{-1}.
HRMS (ESI): calculated for $C_{14}H_{24}OK$ 247.1459 $[M+K]^+$, found 247.1522.
^{1}H NMR (500 MHz, $CDCl_3$): δ 4.89 (t, *J* = 7.3 Hz, 1H), 2.20 (d, *J* = 7.3 Hz, 2H), 2.09 (s, 3H), 2.02 (dd, *J* = 13.9, 8.4 Hz, 2H), 1.66 (s, 3H), 1.59 (s, 3H), 1.57 – 1.34 (m, 10H) ppm.
^{13}C NMR (125 MHz, $CDCl_3$): δ 213.8, 134.3, 119.7, 55.7, 38.5, 35.0, 30.5, 26.1, 25.7, 23.6, 18.1 ppm.

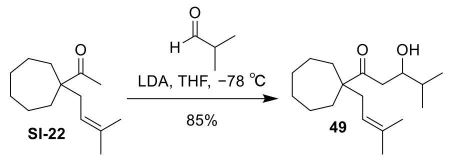

Diisopropylamine (0.32 mL, 2.30 mmol, 1.6 eq.) was dissolved in dry THF (10 mL) and cooled to 0 °C. *n*-BuLi (2.5 M in hexanes, 1.15 mL, 2.30 mmol, 1.6 eq.) was added and the mixture was stirred for 10 min. The mixture was cooled to -78 °C and **SI-22** (300 mg, 1.44 mmol, 1.0 eq.) was added. After stirring for 30 min isobutyrylaldehyde (0.18 mL, 2.02 mmol, 1.4 eq.) was added and the reaction was stirred for 2 h at -78 °C. Sat. aq. NH_4Cl (10 mL) and was added and the dry ice bath was removed to warm the mixture to room temperature. After extraction with Et_2O (3 × 10 mL) the combined organic layers were dried over Na_2SO_4, filtered and concentrated *in vacuo*. The residue was purified by flash column chromatography on SiO_2 (60:1→20:1 gradient elution, petroleum ether/EtOAc) to give **49** as a colourless oil (343 mg, 1.22 mmol, 85%).

Data for 49:
R_f = 0.47 (20:1, petroleum ether/EtOAc).
IR (neat): 2919, 2854, 1687, 1463, 1446 cm^{-1}.
HRMS (ESI): calculated for $C_{18}H_{32}O_2Na$ 303.2295 $[M+Na]^+$, found 303.2300.
^{1}H NMR (500 MHz, $CDCl_3$): δ 4.89 (t, *J* = 7.2 Hz, 1H), 3.72 (m, 1H), 3.38 (d, *J* = 2.6 Hz, 1H), 2.66 (dd, *J* = 17.4, 1.4 Hz, 1H), 2.39 (dd, *J* = 17.4, 9.8 Hz, 1H), 2.20 (d, *J* = 7.3 Hz, 2H), 2.09 – 1.95 (m, 2H), 1.72 – 1.63 (overlapped m, 1H), 1.67 (s, 3H), 1.59 (s, 3H), 1.57 – 1.34 (m, 10H), 0.93 (d, *J* = 6.8 Hz, 3H), 0.91 (d, *J* = 6.8 Hz, 3H) ppm.
^{13}C NMR (125 MHz, $CDCl_3$): δ 218.1, 134.6, 119.4, 72.8, 55.9, 40.9, 38.5, 35.0, 34.9, 33.2, 30.5, 26.1, 23.6, 23.6, 18.6, 18.1, 18.0 ppm.

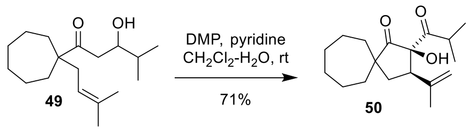

To a solution of **49** (100 mg, 0.36 mmol, 1.0 eq.) in CH_2Cl_2 (2 mL) was added Dess-Martin periodinane (378 mg, 0.89 mmol, 2.5 eq.) and pyridine (66 μL, 0.82 mmol, 2.3 eq.) and the mixture was stirred at room temperature for 1.75 h. Sat. aq. $NaHCO_3$ (10 mL) was added and the reaction mixture was extracted with CH_2Cl_2 (3 × 10 mL). The combined organic layers were dried over Na_2SO_4, filtered and concentrated *in vacuo*. The residue was purified by flash column chromatography on SiO_2 (50:1→30:1 gradient elution, petroleum ether/EtOAc) to give **50** as a colourless oil (74 mg, 0.25 mmol, 71%).

Data for 50:
R_f = 0.45 (20:1, petroleum ether/EtOAc).
IR (neat): 2938, 2925, 1704, 1446, 1368, 1100, 1031, 978 cm^{-1}.
HRMS (ESI): calculated for $C_{18}H_{29}O_3$ 293.2111 $[M+H]^+$, found 293.2112.
^{1}H NMR (500 MHz, $CDCl_3$): δ 5.01 (s, 1H), 4.87 (s, 1H), 3.56 (s, 1H), 3.30 (dd, *J* = 12.9, 6.2 Hz, 1H), 3.01 (sept, *J* = 6.9 Hz, 1H), 2.12 (dd, *J* = 12.5, 6.3 Hz, 1H), 2.00 (t, *J* = 12.8 Hz, 1H), 1.97 – 1.90 (m, 1H), 1.81 – 1.43 (overlapped m, 11H), 1.67 (s, 3H), 1.14 (d, *J* = 6.8 Hz, 3H), 1.07 (d, *J* = 6.7 Hz, 3H) ppm.
^{13}C NMR (125 MHz, $CDCl_3$): δ 216.9, 214.9, 141.7, 114.1, 87.1, 52.7, 47.5, 38.6, 37.8, 36.1, 35.7, 29.8, 29.7, 24.0, 23.8, 23.4, 19.1, 18.8 ppm.

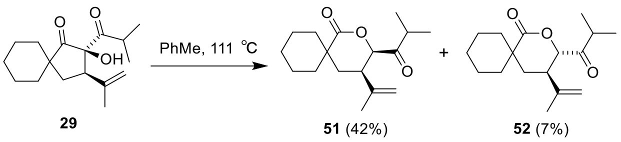

A solution of **29** (100 mg, 0.36 mmol, 1.0 eq.) in toluene (1 mL) was stirred at reflux for 24 h. Then the reaction was cooled to room temperature and volatiles were removed *in vacuo*. The residue was purified by flash column chromatography on SiO_2 (20:1→10:1 gradient elution, petroleum ether/EtOAc) to give **52** as a colourless oil (7 mg, 0.03 mmol, 7%). Further elution gave **51** as a white solid (42 mg, 0.15 mmol, 42%).

Data for 52:
R_f = 0.41 (10:1, petroleum ether/EtOAc).
IR (neat): 2982, 2971, 2908, 1756, 1738, 1691, 1480, 1380, 1279, 1144, 987 cm^{-1}.
HRMS (ESI): calculated for $C_{17}H_{27}O_3$ 279.1955 $[M+H]^+$, found 279.1944.
^{1}H NMR (500 MHz, $CDCl_3$): δ 4.89 (s, 1H), 4.82 (s, 1H), 4.79 (d, *J* = 10.0 Hz, 1H), 2.91 (sept, *J* = 6.9 Hz, 1H), 2.73 (td, *J* = 9.7, 3.6 Hz, 1H), 2.05 – 1.99 (m, 2H), 1.84 – 1.76 (overlapped m, 1H), 1.78 (s, 3H), 1.73 – 1.32 (m, 9H), 1.09 (d, *J* = 6.8 Hz, 6H) ppm.
^{13}C NMR (125 MHz, $CDCl_3$): δ 209.2, 175.5, 143.3, 114.3, 83.9, 41.9, 39.9, 37.5, 36.1, 34.7, 33.7, 25.5, 21.1, 20.9, 20.2, 18.3, 18.2 ppm.

Data for 51:
R_f = 0.24 (10:1, petroleum ether/EtOAc).
Mp: 105 – 107 °C.
IR (neat): 2981, 2971, 1724, 1382, 1137, 1098 cm^{-1}.
HRMS (ESI): calculated for $C_{17}H_{27}O_3$ 279.1955 $[M+H]^+$, found 279.1956.
^{1}H NMR (500 MHz, $CDCl_3$): δ 5.21 (d, *J* = 5.2, 1H), 4.98 (s, 1H), 4.79 (s, 1H), 2.86 (dt, *J* = 12.2, 4.1 Hz, 1H), 2.54 (sept, *J* = 6.9 Hz, 1H), 2.20 – 2.13 (m, 1H), 1.93 – 1.87 (overlapped m, 1H), 1,93 (s, 3H), 1.83 – 1.52 (m, 7H), 1.47 – 1.26 (m, 3H), 1.11 (d, *J* = 7.2 Hz, 3H), 0.99 (d, *J* = 6.5 Hz, 3H) ppm.
^{13}C NMR (125 MHz, $CDCl_3$): δ 210.7, 176.8, 141.9, 113.8, 80.8, 42.3, 40.0, 39.7, 35.8, 35.1, 30.0, 25.5, 23.0, 21.2, 20.9, 19.0, 16.1 ppm.

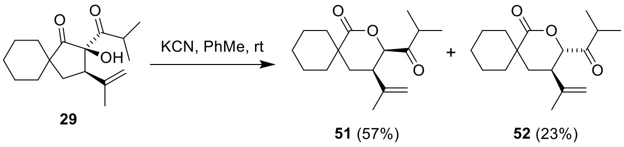

To a solution of **29** (100 mg, 0.36 mmol, 1.0 eq.) in toluene (1 mL) KCN (6 mg, 0.09 mmol, 0.25 eq.) was added. The mixture was stirred for 18 h at room temperature and then volatiles were removed *in vacuo*. The residue was purified by flash column chromatography on SiO_2 (12:1→7:1 gradient elution, petroleum ether/EtOAc) to give **52** as a colourless oil (23.2 mg, 0.08 mmol, 23%). Further elution gave **51** as a white solid (57 mg, 0.20 mmol, 57%). Data for **51** and **52** matched that previously obtained.

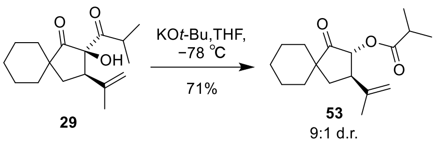

To a solution of **29** (100 mg, 0.36 mmol, 1.0 eq.) in dry THF (1.7 mL) at -78 °C KO*t*-Bu (48 mg, 0.43 mmol, 1.2 eq.) was added and the mixture was stirred for 10 min before sat. aq. NH_4Cl solution (10 mL) was added. The mixture was extracted with Et_2O (3 × 10 mL) and the combined organic layers were dried over Na_2SO_4, filtered and concentrated *in vacuo*. The residue was purified by flash column chromatography on SiO_2 (40:1→20:1 gradient elution, petroleum ether/EtOAc) to give **53** in a mixture of diastereoisomers (ratio 9:1) as a colourless oil (71 mg, 0.26 mmol, 71%).

Data for 53 (mixture of isomers):
R_f = 0.57 (10:1, petroleum ether/EtOAc).
IR (neat): 2925, 2855, 1754, 1743, 1740, 1469, 1449, 1187, 1151 cm^{-1}.
HRMS (ESI): calculated for $C_{17}H_{26}O_3Na$ 301.1774 $[M+Na]^+$, found 301.1771.

Major isomer:
^1H NMR (500 MHz, $CDCl_3$): δ 5.30 (d, *J* = 12.3 Hz, 1H), 4.85 (s, 1H), 4.83 (s,1H), 2.80 (td, *J* = 12.4, 6.8 Hz, 1H), 2.62 (sept, *J* = 7.0 Hz, 1H), 2.23 (dd, *J* = 13.2, 6.8 Hz, 1H), 1.78 (s, 3H), 1.72 – 1.52 (m, 6H), 1.47 – 1.23 (m, 5H), 1.20 (d, *J* = 7.0 Hz, 3H), 1.17 (d, *J* = 7.0 Hz, 3H) ppm.
^{13}C NMR (125 MHz, $CDCl_3$): δ 215.1, 176.3, 143.4, 112.3, 77.6, 47.4, 45.3, 35.3, 35.0, 34.0, 32.1, 25.5, 22.0, 21.8, 19.7, 19.2, 19.1 ppm.

Minor isomer:
^1H NMR (500 MHz, $CDCl_3$): δ 5.31 (d, *J* = 7.5 Hz, 1H), 4.89 – 4.87 (m, 1H), 4.80 – 4.78 (m, 1H), 2.92 (q, *J* = 7.7 Hz, 1H), 2.55 (sept, *J* = 7.0 Hz, 1H), 2.13 (dd, *J* = 12.6, 7.0 Hz, 1H), 1.98 (dd, *J* = 13.3, 8.8 Hz, 1H), 1.74 (s, 3H), 1.71 – 1.52 (m, 5H), 1.49 – 1.24 (m, 5H), 1.20 (d, *J* = 6.5 Hz, 3H), 1.16 (d, *J* = 6.5 Hz, 3H) ppm.
^{13}C NMR (125 MHz, $CDCl_3$, partially observed): δ 216.6, 175.8, 142.1, 113.0, 74.5, 43.4, 34.1, 29.9, 22.7, 22.2, 19.2, 19.01 ppm.

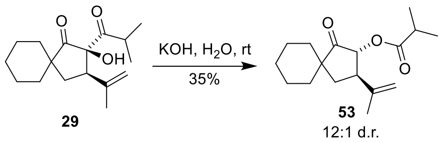

To a suspension of **29** (53 mg, 0.19 mmol, 1.0 eq.) in H_2O (0.5 mL) KOH (13 mg, 0.23 mmol, 1.2 eq.) was added. The mixture was stirred for 2.5 h at room temperature before sat. aq. NH_4Cl solution (10 mL) was added. The mixture was extracted with Et_2O (3 × 10 mL) and the combined organic layers were dried over Na_2SO_4, filtered and concentrated *in vacuo*. The residue was purified by flash column chromatography on SiO_2 (40:1, petroleum ether/EtOAc) to give **53** in a mixture of diastereoisomers (d.r. 12:1) as a colourless oil (19 mg, 0.07 mmol, 35%). Data for **53** matched that previously obtained.

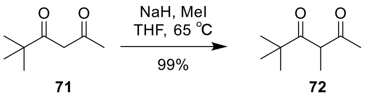

To a solution of **71** (1.74 g, 12.2 mmol, 1.0 eq.) in dry THF (65 mL) was added NaH (60% in mineral oil, 643 mg, 15.9 mmol, 1.3 eq.) and MeI (0.84 mL, 13.4 mmol, 1.1 eq.). The mixture was heated to reflux and stirred for 4 h. The mixture was cooled to room temperature and sat. aq. NH_4Cl (100 mL) was added. The mixture was extracted with Et_2O (3 × 50 mL) and the combined organic layers were dried over Na_2SO_4, filtered and concentrated *in vacuo* to yield **72** as a yellow liquid (1.89 g, 12.1 mmol, 99%).

Data for 72:
R_f = 0.37 (50:1, petroleum ether/EtOAc).
IR (neat): 2953, 2922, 2853, 1456, 1376 cm^{-1}.
HRMS (ESI): calculated for $C_9H_{16}O_2Na$ 179.1042 $[M+Na]^+$, found 179.1068.
^{1}H NMR (500 MHz, $CDCl_3$): δ 4.07 (q, *J* = 7.0 Hz, 1H), 2.14 (s, 3H), 1.31 (d, *J* = 7.0 Hz, 3H), 1.17 (s, 9H) ppm.
^{13}C NMR (125 MHz, $CDCl_3$): δ 212.6, 205.1, 55.5, 45.7, 27.6, 26.3, 15.3 ppm.

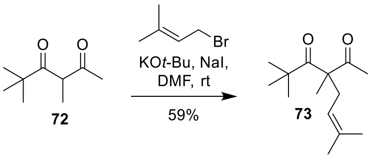

To a solution of **72** (959 mg, 6.14 mmol, 1.0 eq.) in DMF (30 mL) was added KO*t*-Bu (827 mg, 7.37 mmol, 1.2 eq.) and NaI (92 mg, 0.614 mmol, 0.1 eq.) and prenyl bromide (0.85 mL, 7.37 mmol, 1.2 eq.). The mixture was stirred for 2.5 h at room temperature. Sat. aq. NH_4Cl (50 mL) was added and the mixture was extracted with Et_2O (3 × 20 mL). The combined organic layers were dried over Na_2SO_4, filtered and concentrated *in vacuo*. The residue was purified by flash column chromatography on SiO_2 (50:1→40:1 gradient elution, petroleum ether/EtOAc) to give **73** as a colourless liquid (817 mg, 3.64 mmol, 59%).

Data for 73:
R_f = 0.49 (20:1, petroleum ether/EtOAc).
IR (neat): 1706, 1684, 1482, 1359, 980 cm^{-1}.
HRMS (ESI): calculated for $C_{14}H_{24}O_2Na$ 247.1669 $[M+Na]^+$, found 247.1604.
^{1}H NMR (500 MHz, $CDCl_3$): δ 4.84 (m, 1H), 2.55 (d, *J* = 7.3 Hz, 1H), 2.10 (s, 3H), 1.66 (s, 3H), 1.60 (s, 3H), 1.33 (s, 3H), 1.17 (s, 9H) ppm.
^{13}C NMR (125 MHz, $CDCl_3$): δ 213.9, 207.3, 135.5, 118.3, 66.8, 46.4, 34.4, 28.7, 27.6, 26.1, 19.3, 18.2 ppm.

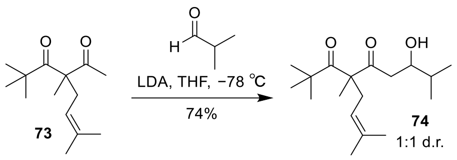

Diisopropylamine (4.39 mL, 31.2 mmol, 1.4 eq.) was dissolved in dry THF (65 mL) and cooled to 0 °C. n-BuLi (11 M in hexanes, 2.84 mL, 31.2 mmol, 1.4 eq.) was added and the mixture was stirred for 10 min. The mixture was cooled to -78 °C and **73** (5.00 g, 22.3 mmol, 1.0 eq.) was added. After stirring for 30 min isobutyrylaldehyde (4.07 mL, 44.6 mmol, 2.0 eq.) was added and the reaction was stirred for 1.5 h. Sat. aq. NH_4Cl (100 mL) and Et_2O (50 mL) was added and the dry ice bath was removed to warm the mixture to room temperature. The phases were separated and the aqueous phase was extracted with Et_2O (2 × 50 mL). The combined organic layers were dried over Na_2SO_4, filtered and concentrated *in vacuo*. The residue was purified by flash column chromatography on SiO_2 (30→10:1 gradient elution, petroleum ether/EtOAc) to give **74** in a mixture of diastereoisomers (ratio 1:1) as a colourless oil (4.89 g, 16.5 mmol, 74%).

Data for 74 (mixture of isomers):
R_f = 0.40 (10:1, petroleum ether/EtOAc).
IR (neat): 2968, 2935, 1727, 1710, 1283, 1158 cm^{-1}.
HRMS (ESI): calculated for $C_{18}H_{32}O_3Na$ 319.2244 $[M+Na]^+$, found 319.2242.
^{1}H NMR (500 MHz, $CDCl_3$): δ 4.88 – 4.84 (m, 2H), 3.82 – 3.75 (m, 2H), 3.17 (d, *J* = 2.8 Hz, 1H), 3.07 (d, *J* = 2.9 Hz, 1H), 2.63 – 2.46 (m, 8H), 2.33 (dd, *J* = 18.1, 9.8 Hz, 1H), 1.70 – 1.64 (overlapping m, 1H), 1.68 (s, 6H), 1.59 (s, 6H), 1.17 (s, 9H), 1.17 (s, 9H), 0.92 (d, *J* = 6.8 Hz, 3H), 0.91 (d, *J* = 6.8 Hz, 3H), 0.90 (d, *J* = 6.8 Hz, 3H), 0.88 (d, *J* = 6.8 Hz, 3H) ppm.
^{13}C NMR (125 MHz, $CDCl_3$): δ 213.6, 213.5, 211.7, 211.2, 135.8, 135.6, 118.0, 117.9, 72.1, 72.0, 66.6, 66.4, 46.11, 46.0, 43.2, 43.2, 34.8, 34.7, 32.9, 32.9, 28.6, 28.5, 26.0, 26.0, 19.2, 19.2, 18.9, 18.3, 18.3, 18.0, 17.8, 17.6 ppm.

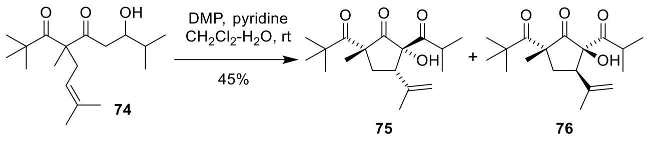

To a solution of **74** (3.00 g, 10.1 mmol, 1.0 eq.) in CH_2Cl_2 (50 mL) Dess-Martin periodinane (10.7 g, 25.3 mmol, 2.5 eq.) and pyridine (1.87 mL, 23.3 mmol, 2.3 eq.) were added. The mixture was stirred for 1.75 h at room temperature before sat. aq. $NaHCO_3$ (100 mL) was added. The phases were separated and the aqueous phase was extracted with CH_2Cl_2 (2 × 50 mL). The combined organic layers were dried over Na_2SO_4, filtered and concentrated *in vacuo*. The residue was purified by flash column chromatography on SiO_2 (30:1→20:1 gradient elution, petroleum ether/EtOAc) to give **75** and **76** in a mixture of diastereoisomers (ratio 3.4:1) as a yellow oil (1.41 g, 4.57 mmol, 45%).

Data for 75/76:
R_f = 0.44 (10:1, petroleum ether/EtOAc)
IR (neat): 3467, 2973, 2936, 1744, 1690, 1459, 1368 cm^{-1}.
HRMS (ESI): calculated for $C_{18}H_{29}O_4$ 309.2060 $[M+H]^+$, found 309.2060.

Data for 75:
^{1}H NMR (500 MHz, $CDCl_3$): δ 5.06 (s, 1H), 4.89 (s, 1H), 3.74 (s, 1H), 3.36 (dd, *J* = 12.7, 6.5 Hz, 1H), 3.13 (sept, *J* = 6.8 Hz, 1H), 2.64 (t, *J* = 12.8 Hz, 1H),), 2.04 (dd, *J* = 12.9, 6.6 Hz, 1H), 1.69 (s, 3H), 1.55 (s, 3H), 1.24 (s, 9H), 1.17 (d, *J* = 6.8 Hz, 3H), 1.09 (d, *J* = 6.7 Hz, 3H) ppm.
^{13}C NMR (125 MHz, $CDCl_3$): δ 214.6, 210.4, 140.9, 115.1, 86.7, 62.6, 47.4, 46.1, 37.8, 36.2, 27.7, 27.7, 23.4, 22.8, 18.8, 18.7 ppm.

Data for 76:
^{1}H NMR (500 MHz, $CDCl_3$): δ 4.99 (s, 1H), 4.82 (s, 1H), 3.75 (s, 1H), 3.31 (dd, *J* = 12.7, 6.3 Hz, 1H), 3.23 (sept, *J* = 6.7 Hz, 1H), 2.77 (dd, *J* = 12.9, 6.4 Hz, 1H), 1.92 (t, *J* = 12.8 Hz, 1H), 1.66 (s, 3H), 1.60 (s, 3H), 1.26 (s, 9H), 1.13 (d, *J* = 6.7 Hz, 3H), 1.10 (d, *J* = 6.7 Hz, 3H) ppm.
^{13}C NMR (125 MHz, $CDCl_3$): δ 212.9, 211.5, 141.5, 114.4, 86.8, 64.6, 48.0, 47.0, 38.2, 35.6, 29.9, 28.3, 23.2, 22.5, 19.3, 19.0 ppm.

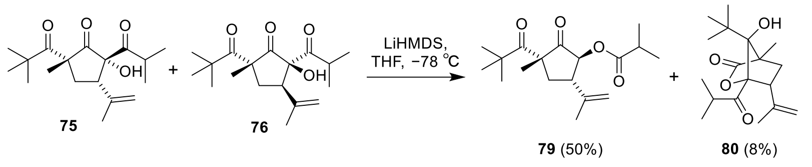

To a solution of **75/76** (100 mg, 0.32 mmol, 1.0 eq.) in dry THF (1 mL) at -78 °C was added LiHMDS (0.48 mL, 1.0 M in THF, 0.48 mmol, 1.5 eq.). The mixture was stirred for 1 h at -78 °C. Sat. aq. NH_4Cl (10 mL) was added and the dry ice bath was removed to slowly warm the reaction to room temperature. The mixture was extracted with Et_2O (3 × 10 mL) and the combined organic layers were dried over Na_2SO_4, filtered and concentrated *in vacuo*. The residue was purified by flash column chromatography on SiO_2 (30:1, petroleum ether/EtOAc) to give **79** as a colourless liquid (50 mg, 0.16 mmol, 50%). Further elution gave **80** as a white solid (8 mg, 0.026 mmol, 8%).

Data for 79:
R_f = 0.65 (10:1, petroleum ether/EtOAc).
IR (neat): 2975, 1756, 1743, 1691, 1470, 1185, 1149, 988, 896 cm^{-1}.
HRMS (ESI): calculated for $C_{18}H_{28}O_4K$ 347.1619 $[M+K]^+$, found 347.1660.
^{1}H NMR (500 MHz, $CDCl_3$): δ 5.49 (d, *J* = 12.2 Hz, 1H), 4.87 (s, 1H), 4.82 (s, 1H), 2.81 (td, *J* = 12.3, 7.0 Hz, 1H), 2.63 (sept, *J* = 6.9 Hz, 1H), 2.18 (t, *J* = 12.9 Hz, 1H), 2.04 (dd, *J* = 13.5, 7.0 Hz, 1H), 1.76 (s, 3H), 1.44 (s, 3H), 1.21 (d, *J* = 6.9 Hz, 3H), 1.19 (s, 9H), 1.17 (d, *J* = 7.4 Hz, 3H) ppm.
^{13}C NMR (125 MHz, $CDCl_3$): δ 212.3, 209.9, 176.2, 142.4, 112.9, 78.2, 59.3, 46.3, 43.8, 36.5, 33.9, 27.3, 23.7, 19.6, 19.1, 19.1 ppm.

Data for 80:
R_f = 0.35 (10:1, petroleum ether/EtOAc).
Mp: 118 – 124 °C.
IR (neat): 3485, 2981, 2935, 1788, 1694, 1215, 1092 cm^{-1}.
HRMS (ESI): calculated for $C_{18}H_{29}O_4$ 309.2060 $[M+H]^+$, found 309.2059.
^{1}H NMR (500 MHz, $CDCl_3$): δ 4.90 (s, 1H), 4.77 (s, 1H), 3.65 (dd, *J* = 10.5, 4.8 Hz, 1H), 3.10 (sept, *J* = 6.8 Hz, 1H), 2.95 – 2.94 (m, 1H), 2.50 (dd, *J* = 12.7, 10.5 Hz, 1H), 1.71 (s, 3H), 1.50 (dd, *J* = 12.7, 4.9 Hz, 1H), 1.33 (s, 3H), 1.13 (d, *J* = 6.7 Hz, 3H), 1.06 (d, *J* = 7.0 Hz, 3H), 1.01 (s, 9H) ppm.
^{13}C NMR (125 MHz, $CDCl_3$): δ 213.3, 177.7, 141.5, 117.3, 98.6, 93.8, 56.7, 52.2, 38.7, 38.3, 37.0, 28.5, 22.0, 17.9, 17.0, 13.8 ppm.

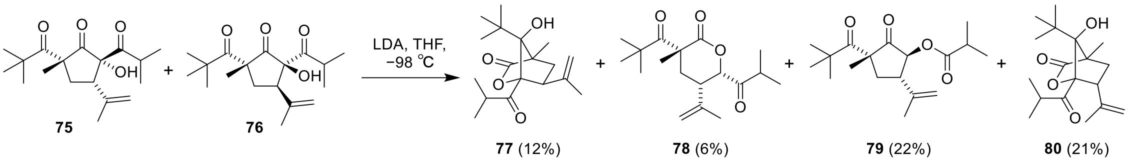

A solution of **75/76** (100 mg, 0.32 mmol, 1.0 eq.) in dry THF (0.5 mL) was cooled to -98 °C. In a separate flask diisopropylamine (55 µL, 0.39 mmol, 1.2 eq.) was dissolved in dry THF (0.5 mL) and cooled to 0 °C. *n*-BuLi (2.5 M in hexanes, 0.16 mL, 0.39 mmol, 1.2 eq.) was added and the mixture was stirred for 10 min. The mixture was cooled to -98 °C and then transferred to the first flask. The reaction was stirred for 1 min before 1 M HCl (10 mL) was added and the cooling bath was removed to warm the mixture to room temperature. The mixture was extracted with Et_2O (3 × 5 mL). The combined organic layers were dried over Na_2SO_4, filtered and concentrated *in vacuo*. The residue was purified by flash column chromatography on SiO_2 (25:1→5:1 gradient elution, petroleum ether/EtOAc) to give **79** (22 mg, 0.071 mmol, 22%). Further elution gave **77** as white solid (12 mg, 0.039 mmol, 12%), **80** (21 mg, 0.068 mmol, 21%) and **78** as a colourless oil (6 mg, 0.019 mmol, 6%). Data for **79** and **80** matched that previously obtained.

Data for 77:
R_f = 0.47 (10:1, petroleum ether/EtOAc).
Mp: 104 – 108 °C.
IR (neat): 2966, 2928, 2360, 1797, 1693, 1259, 1092 cm^{-1}.
HRMS (ESI): calculated for $C_{18}H_{28}O_4Na$ 331.1880 $[M+Na]^+$, found 331.1846.
^{1}H NMR (500 MHz, $CDCl_3$): δ 5.09 – 5.08 (m, 1H), 5.05 (s, 1H), 3.21 (sept, *J* = 6.9 Hz, 1H), 3.15 (s, 1H), 2.65 (t, *J* = 8.5 Hz, 1H), 2.52 (dd, *J* = 12.2, 7.6 Hz, 1H), 1.69 (s, 3H), 1.65 (dd, *J* = 12.2, 9.3 Hz, 1H), 1.38 (s, 3H), 1.23 (d, *J* = 7.2 Hz, 3H), 1.13 (d, *J* = 6.6 Hz, 3H), 0.97 (s, 9H) ppm.
^{13}C NMR (125 MHz, $CDCl_3$): δ 211.9, 177.7, 141.5, 117.6, 96.1, 92.6, 57.4, 55.5, 38.9, 38.6, 35.8, 28.7, 23.9, 17.9, 17.0, 13.7 ppm.

Data for 78:
R_f = 0.20 (10:1, petroleum ether/EtOAc).
IR (neat): 2982, 1736, 1382, 1259, 1054, 1008, 793 cm^{-1}.
HRMS (ESI): calculated for $C_{18}H_{29}O_4$ 309.2060 $[M+H]^+$, found 309.2060.
^{1}H NMR (500 MHz, $CDCl_3$): δ 5.29 (d, *J* = 4.9 Hz, 1H), 5.00 (s, 1H), 4.80 – 4.73 (s, 1H), 2.99 (dt, *J* = 10.8, 5.2 Hz, 1H), 2.71 (sept, *J* = 6.9 Hz, 1H), 2.68 (t, *J* = 13.1 Hz, 1H), 1.86 (s, 3H), 1.58 (s, 3H), 1.58 – 1.54 (overlapped m, 1H), 1.33 (s, 9H), 1.12 (d, *J* = 7.0 Hz, 3H), 1.05 (d, *J* = 6.7 Hz, 3H) ppm.
^{13}C NMR (125 MHz, $CDCl_3$): δ 211.9, 209.5, 171.5, 140.8, 114.5, 81.2, 55.1, 46.1, 40.1, 38.4, 32.0, 28.8, 25.9, 22.8, 18.2, 16.9 ppm.

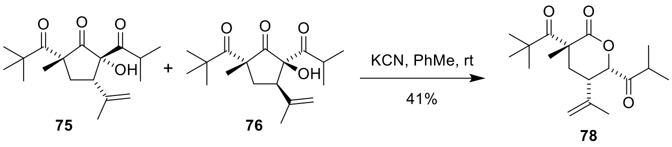

To a solution of **75/76** (120 mg, 0.39 mmol, 1.0 eq.) in toluene (1 mL) KCN (5 mg, 0.08 mmol, 0.2 eq.) was added. The mixture was stirred for 17 h at room temperature and then volatiles were removed *in vacuo*. The residue was purified by flash column chromatography on SiO_2 (50:1→10:1 gradient elution, petroleum ether/EtOAc) to give **78** (49 mg, 0.15 mmol, 41%). Data for **78** matched that previously obtained.

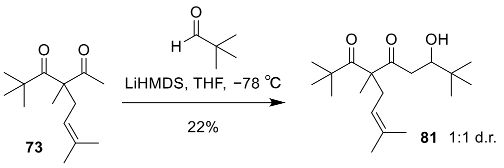

To a solution of **73** (2.00 g, 8.92 mmol, 1.0 eq.) in dry THF (20 mL) at -78 °C was added LiHMDS (14.3 mL, 1.0 M in THF, 14.3 mmol, 1.6 eq.). The mixture was stirred for 25 min before pivaldehyde (1.36 mL, 12.5 mmol, 1.4 eq.) was added and the reaction was stirred for 1 h. Sat. aq. NH_4Cl (50 mL) was added and the dry ice bath was removed to warm the mixture to room temperature. The phases were separated and the aqueous phase was extracted with Et_2O (3 × 10 mL). The combined organic layers were dried over Na_2SO_4, filtered and concentrated *in vacuo*. The residue was purified by flash column chromatography on SiO_2 (80:1→50:1 gradient elution, petroleum ether/EtOAc) to give **81** in a mixture of diastereoisomers (ratio 1:1) as a colourless oil (607 mg, 1.96 mmol, 22%).

Data for 81 (mixture of isomers):
R_f = 0.38 (20:1, petroleum ether/EtOAc).
IR (neat): 2982, 2909, 1683, 1480, 1365, 977 cm^{-1}.
HRMS (ESI): calculated for $C_{19}H_{35}O_3$ 311.2581 $[M+H]^+$, found 311.2582.
^{1}H NMR (500 MHz, $CDCl_3$): δ 4.89 – 4.82 (m, 2H), 3.72 – 3.61 (m, 2H), 3.13 (d, *J* = 2.8 Hz, 1H), 3.04 (d, *J* = 2.9 Hz, 1H), 2.68 – 2.46 (m, 6H), 2.40 (dd, *J* = 18.0, 10.0 Hz, 1H), 2.28 (dd, *J* = 18.0, 10.2 Hz, 1H), 1.67 (s, 6H), 1.59 (s, 6H), 1.36 (s, 6H), 1.17 (s, 9H), 1.16 (s, 9H), 0.88 (s, 9H), 0.88 (s, 9H) ppm.
^{13}C NMR (125 MHz, $CDCl_3$): δ 213.8, 213.8, 211.9, 211.5, 135.9, 135.7, 118.1, 118.0, 74.8, 74.8, 66.8, 66.6, 46.3, 46.2, 41.6, 41.5, 35.0, 34.9, 34.3, 34.3, 28.7, 28.7, 26.2, 26.1, 25.8, 25.8, 19.3, 19.1, 18.2, 18.2 ppm.

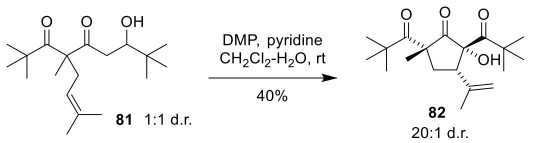

To a solution of **81** (100 mg, 0.32 mmol, 1.0 eq.) in CH_2Cl_2 (2 mL) Dess-Martin periodinane (342 mg, 0.81 mmol, 2.5 eq.) and pyridine (60 µL, 0.74 mmol, 2.3 eq.) were added. The mixture was stirred for 5 h at room temperature and then stirred for another 16 h at reflux. Sat. aq. $NaHCO_3$ (10 mL) was added and the phases were separated. The aqueous phase was extracted with CH_2Cl_2 (3 × 10 mL). The combined organic layers were dried over Na_2SO_4, filtered and concentrated *in vacuo*. The residue was purified by flash column chromatography on SiO_2 (40:1→30:1 gradient elution, petroleum ether/EtOAc) to give **82** in a mixture of diastereoisomers (ratio 20:1) as a yellow oil (42 mg, 0.13 mmol, 40%).

Data for 82:
R_f = 0.44 (10:1, petroleum ether/EtOAc).
IR (neat): 2982, 1757, 1687, 1482, 1366, 1159, 984 cm^{-1}.
HRMS (ESI): calculated for $C_{19}H_{30}O_4Na$ 345.2036 $[M+Na]^+$, found 345.2042.

Major Isomer:
^1H NMR (500 MHz, $CDCl_3$): δ 5.08 (s, 1H), 4.94 (s, 1H), 3.47 (dd, *J* = 13.0, 6.8 Hz, 1H), 3.44 (s, 1H), 2.49 (t, *J* = 12.9 Hz, 1H), 1.94 (dd, *J* = 13.0, 6.8 Hz, 1H), 1.73 (s, 3H), 1.56 (s, 3H), 1.24 (s, 18H) ppm.
^{13}C NMR (125 MHz, $CDCl_3$): δ 215.0, 214.6, 209.2, 141.4, 115.5, 88.6, 62.6, 48.8, 46.2, 45.1, 37.97, 27.5, 26.2, 23.8, 22.3 ppm.

Minor Isomer:
^1H NMR (500 MHz, $CDCl_3$): δ 5.03 (s, 1H), 4.81 (s, 1H), 3.36 (dd, *J* = 11.5, 6.9 Hz, 1H), 3.16 (s, 1H), 2.88 (dd, *J* = 12.9, 6.9 Hz, 1H), 1.69 (s, 3H), 1.61 – 1.58 (m, 1H), 1.51 (s, 3H), 1.22 (s, 18H) ppm.
^{13}C NMR (125 MHz, $CDCl_3$): not observed.

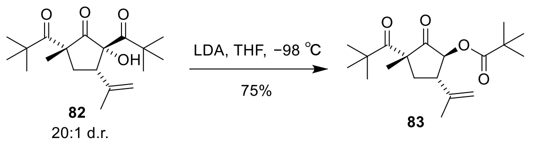

A solution of **82** (40 mg, 0.12 mmol, 1.0 eq.) in dry THF (0.5 mL) was cooled to -98 °C. In a separate flask diisopropylamine (21 µL, 0.15 mmol, 1.2 eq.) was dissolved in dry THF (0.5 mL) and cooled to 0 °C. *n*-BuLi (2.0 M in hexanes, 75 µL, 0.15 mmol, 1.2 eq.) was added and the mixture was stirred for 5 min. The mixture was cooled to -98 °C and then transferred to the first flask. The reaction was stirred for 3 min before 1 M HCl (20 mL) was added and the cooling bath was removed to warm the mixture to room temperature. The phases were separated and the aqueous phase was extracted with Et_2O (3 × 10 mL). The combined organic layers were dried over Na_2SO_4, filtered and concentrated *in vacuo*. The residue was purified by flash column chromatography on SiO_2 (25:1, petroleum ether/EtOAc) to give **83** as a colourless oil (30 mg, 0.093 mmol, 75%).

Data for 83:
R_f = 0.62 (10:1, petroleum ether/EtOAc).
IR (neat): 2982, 2908, 1756, 1738, 1480, 1279, 1144, 987 cm^{-1}.
HRMS (ESI): calculated for $C_{19}H_{31}O_4$ 323.2217 [M+H]$^+$, found 323.2219.
^{1}H NMR (500 MHz, $CDCl_3$): δ 5.47 (d, *J* = 12.3 Hz, 1H), 4.89 (s, 1H), 4.83 (s, 1H), 2.83 (td, *J* = 12.3, 7.0 Hz, 1H), 2.21 (t, *J* = 13.0 Hz, 1H), 2.06 (dd, *J* = 13.5, 7.0 Hz, 1H), 1.78 (s, 3H), 1.47 (s, 3H), 1.25 (s, 9H), 1.21 (s, 9H) ppm.
^{13}C NMR (125 MHz, $CDCl_3$): δ 212.4, 209.9, 177.6, 142.5, 112.8, 78.5, 59.3, 46.3, 43.6, 38.9, 36.5, 27.3, 27.3, 23.9, 19.8 ppm.

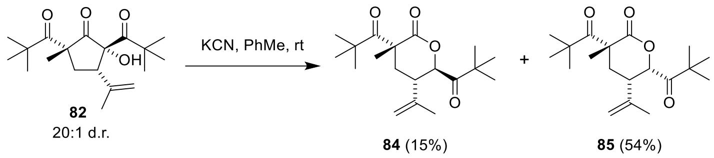
82
20:1 d.r.
84 (15%) **85** (54%)

To a solution of **82** (100 mg, 0.31 mmol, 1.0 eq.) in toluene (1 mL) KCN (5 mg, 0.08 mmol, 0.25 eq.) was added. The mixture was stirred for 20 h at room temperature and then volatiles were removed *in vacuo*. The residue was purified by flash column chromatography on SiO_2 (50:1→20:1 gradient elution, petroleum ether/EtOAc) to give **84** as a colourless oil (15 mg, 0.05 mmol, 15%). Further elution gave **85** as a white solid (54 mg, 0.17 mmol, 54%).

Data for 84:
R_f = 0.43 (10:1, petroleum ether/EtOAc).
IR (neat): 2982, 1736, 1697, 1479, 1375, 1110, 1003, 910 cm^{-1}.
HRMS (ESI): calculated for $C_{19}H_{30}O_4Na$ 345.2036 $[M+Na]^+$, found 345.2033.
^{1}H NMR (500 MHz, $CDCl_3$): δ 5.19 (d, *J* = 10.8 Hz, 1H), 4.95 (s, 1H), 4.84 (s, 1H), 3.05 (ddd, *J* = 12.6, 10.8, 4.3 Hz, 1H), 2.39 (t, *J* = 13.2 Hz, 1H), 1.76 (s, 3H), 1.64 (dd, *J* = 13.7, 4.3 Hz, 1H), 1.55 (s, 3H), 1.30 (s, 9H), 1.19 (s, 9H) ppm.
^{13}C NMR (125 MHz, $CDCl_3$): δ 210.3, 206.9, 170.8, 142.1, 115.4, 79.3, 54.6, 45.7, 44.2, 38.1, 35.6, 29.0, 26.0, 24.4, 21.3 ppm.

Data for 85:
R_f = 0.33 (10:1, petroleum ether/EtOAc).
Mp: 142 – 144 °C.
IR (neat): 2981, 1728, 1687, 1482, 1368, 1097, 907 cm^{-1}.
HRMS (ESI): calculated for $C_{19}H_{31}O_4$ 323.2217 $[M+H]^+$, found 323.2220.
^{1}H NMR (500 MHz, $CDCl_3$): δ 5.45 (d, *J* = 5.1 Hz, 1H), 4.99 (s, 1H), 4.84 (s, 1H), 3.20 (t, *J* = 13.3 Hz, 1H), 2.99 (ddd, *J* = 13.3, 6.3, 3.6 Hz, 1H), 1.74 (s, 3H), 1.55 (s, 3H), 1.47 (dd, *J* = 13.1, 3.6 Hz, 1H), 1.27 (s, 9H), 1.15 (s, 9H) ppm.
^{13}C NMR (125 MHz, $CDCl_3$): δ 211.9, 210.0, 171.3, 140.7, 115.8, 76.4, 54.9, 45.7, 44.7, 38.0, 31.9, 28.9, 26.1, 25.5, 22.6 ppm.

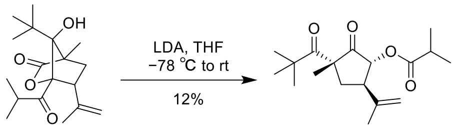

To a solution of diisopropylamine (20 μL, 0.14 mmol, 1.2 eq.) in dry THF (0.5 mL) at 0 °C was added *n*-BuLi (2.0 M in hexanes, 70 μL, 0.14 mmol, 1.2 eq.). The mixture was stirred for 10 min at 0 °C, then cooled to -78 °C. Aldol product **80** (37.0 mg, 0.12 mmol, 1.0 eq.) was added and the mixture was stirred for 30 min at -78 °C before the dry ice bath was replaced by ice water bath and stirred for another 75 min. The reaction was quenched with sat. aq. NH_4Cl solution (10 mL) and extracted with Et_2O (2 × 10 mL). The combined organic layers were dried over Na_2SO_4, filtered and concentrated *in vacuo*. The residue was purified by flash column chromatography on SiO_2 (30:1, petroleum ether/EtOAc) to give **88** as a colourless oil (5 mg, 0.016 mmol, 12%).

Data for 88:

R_f = 0.63 (10:1, petroleum ether/EtOAc).

IR (neat): 3020, 2253, 1690, 1262, 1215, 906, 754, 727 cm^{-1}.

HRMS (ESI): calculated for $C_{18}H_{29}O_4$ 309.2060 $[M+H]^+$, found 309.2062.

^1H NMR (500 MHz, $CDCl_3$): δ 5.31 (d, *J* = 11.6 Hz, 1H), 4.84 (s, 1H), 4.80 (s, 1H), 2.93 (dd, *J* = 13.3, 6.4 Hz, 1H), 2.62 (sept, *J* = 7.0 Hz, 1H), 2.60 – 2.54 (overlapped m, 1H), 1.76 (d, *J* = 1.3 Hz, 3H), 1.47 (dd, *J* = 13.1, 10.6 Hz, 1H), 1.43 (s, 3H), 1.22 (s, 9H), 1.19 (d, *J* = 7.0 Hz, 3H), 1.17 (d, *J* = 7.0 Hz, 3H) ppm.

^{13}C NMR (125 MHz, $CDCl_3$): δ 211.3, 211.1, 176.1, 142.6, 112.5, 76.2, 62.8, 46.7, 46.4, 37.3, 33.9, 27.4, 23.8, 19.9, 19.1, 19.1 ppm.

3. NMR Spectra

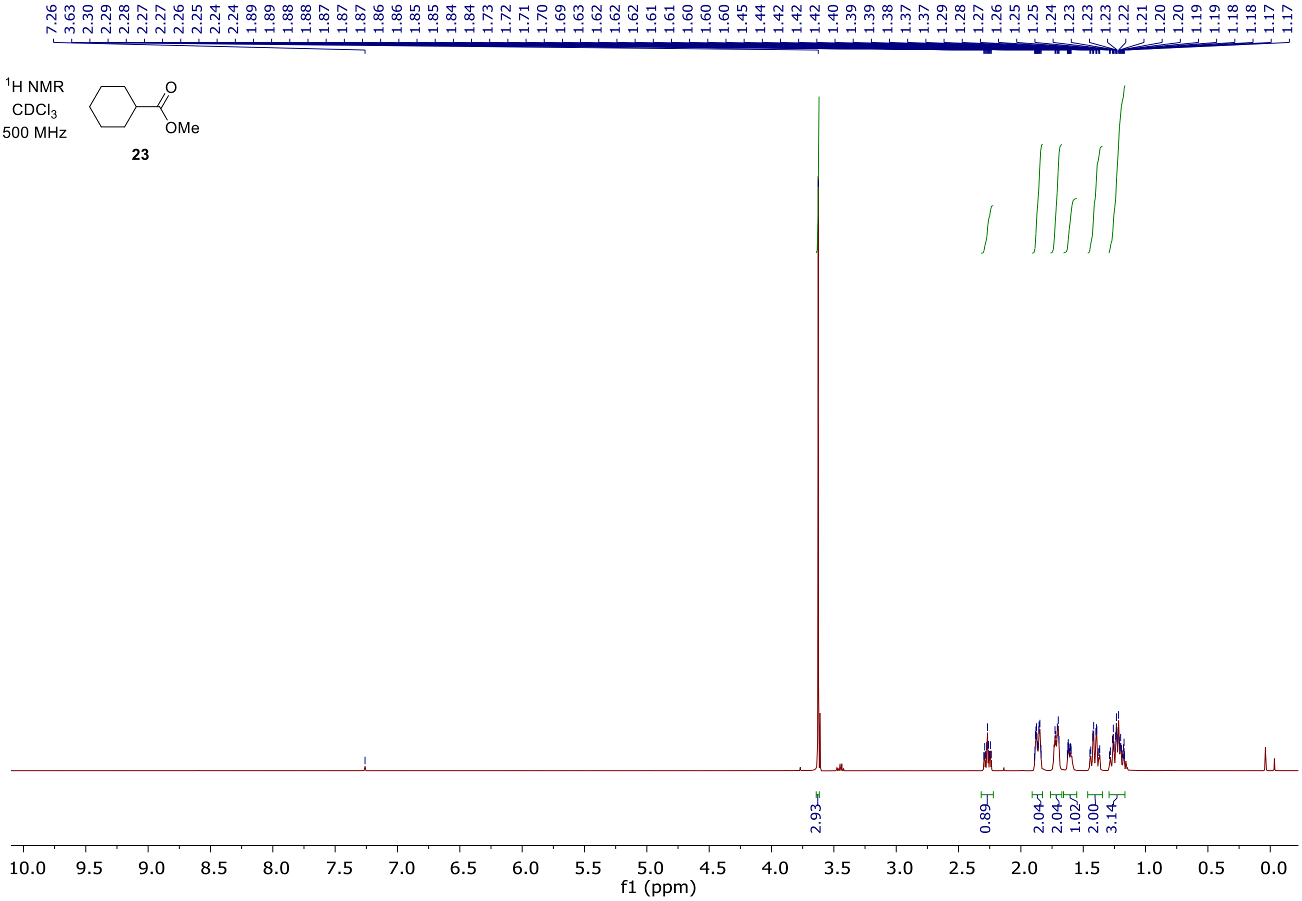

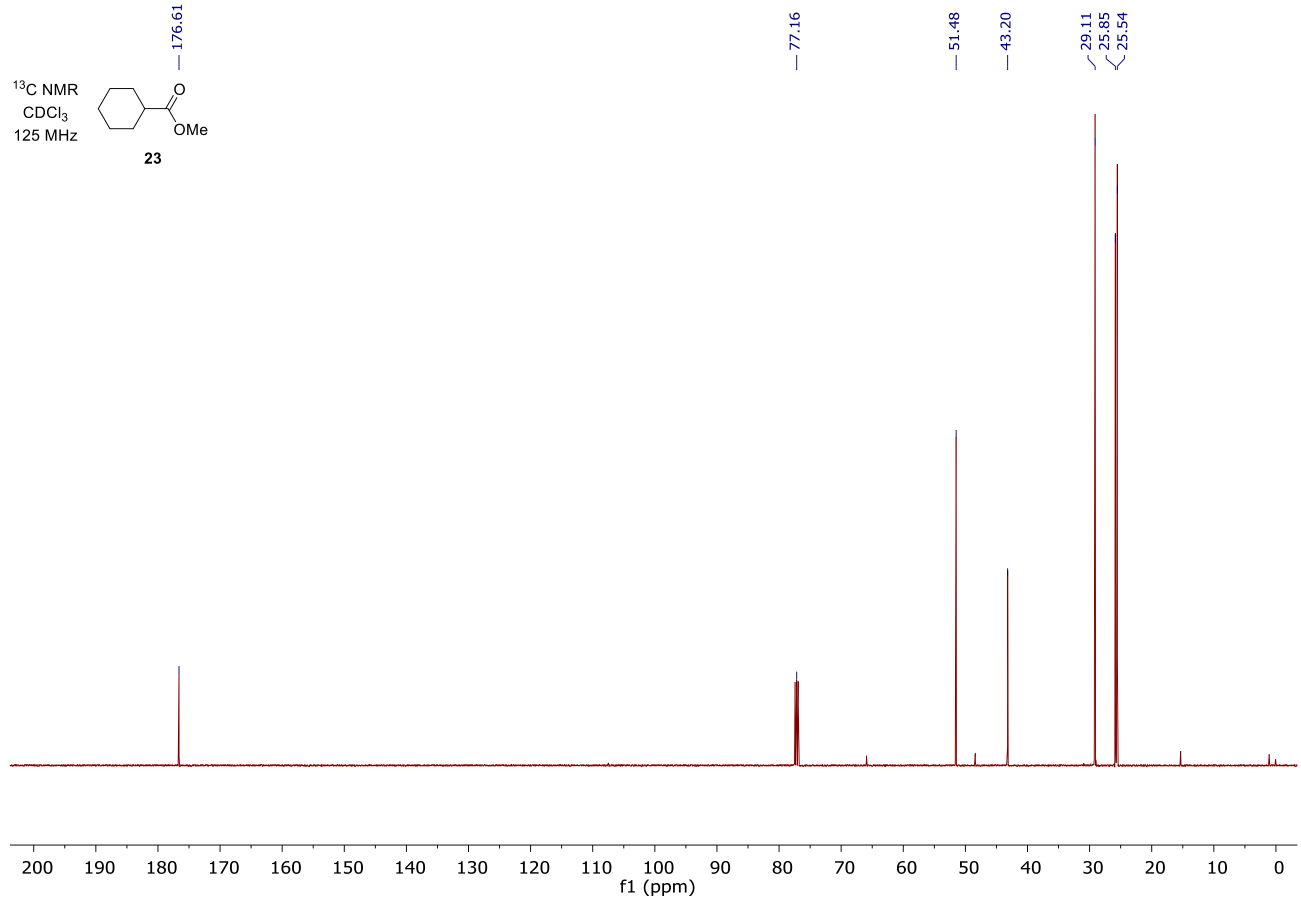

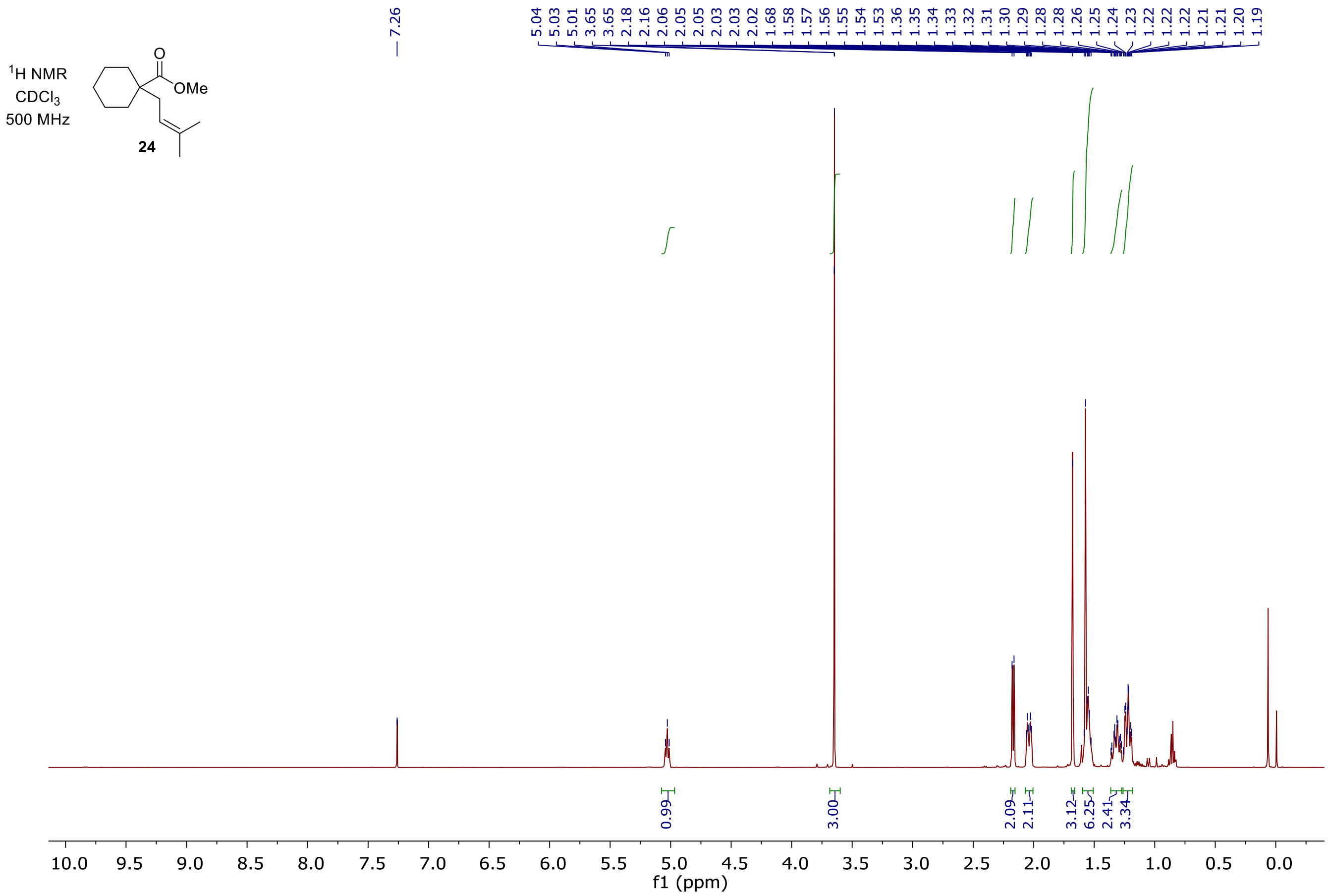

1H NMR
CDCl3
500 MHz
OMe
24
7.26
5.04 5.03 5.01 3.65 3.65 2.18 2.16 2.06 2.05 2.05 2.03 2.03 2.02 1.68 1.58 1.57 1.56 1.55 1.54 1.53 1.36 1.35 1.34 1.33 1.32 1.31 1.30 1.29 1.28 1.28 1.26 1.25 1.24 1.23 1.22 1.22 1.22 1.21 1.20 1.19
0.99 3.00 2.09 2.11 3.12 6.25 2.41 3.34
f1 (ppm)

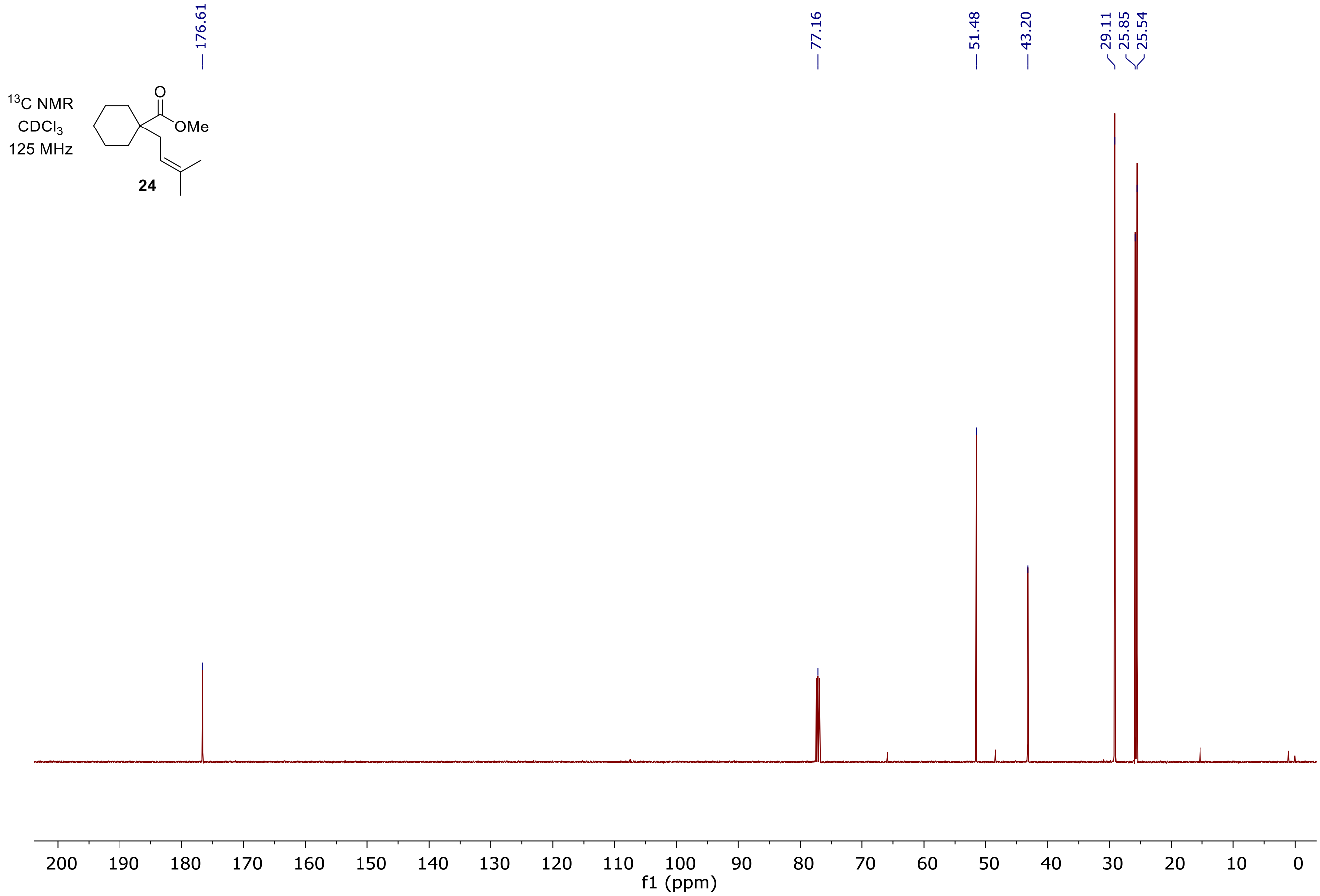

13C NMR
CDCl3
125 MHz
OMe
24
176.61
77.16
51.48
43.20
29.11 25.85 25.54
f1 (ppm)

^{1}H NMR
$CDCl_3$
500 MHz

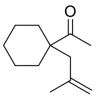

SI-3

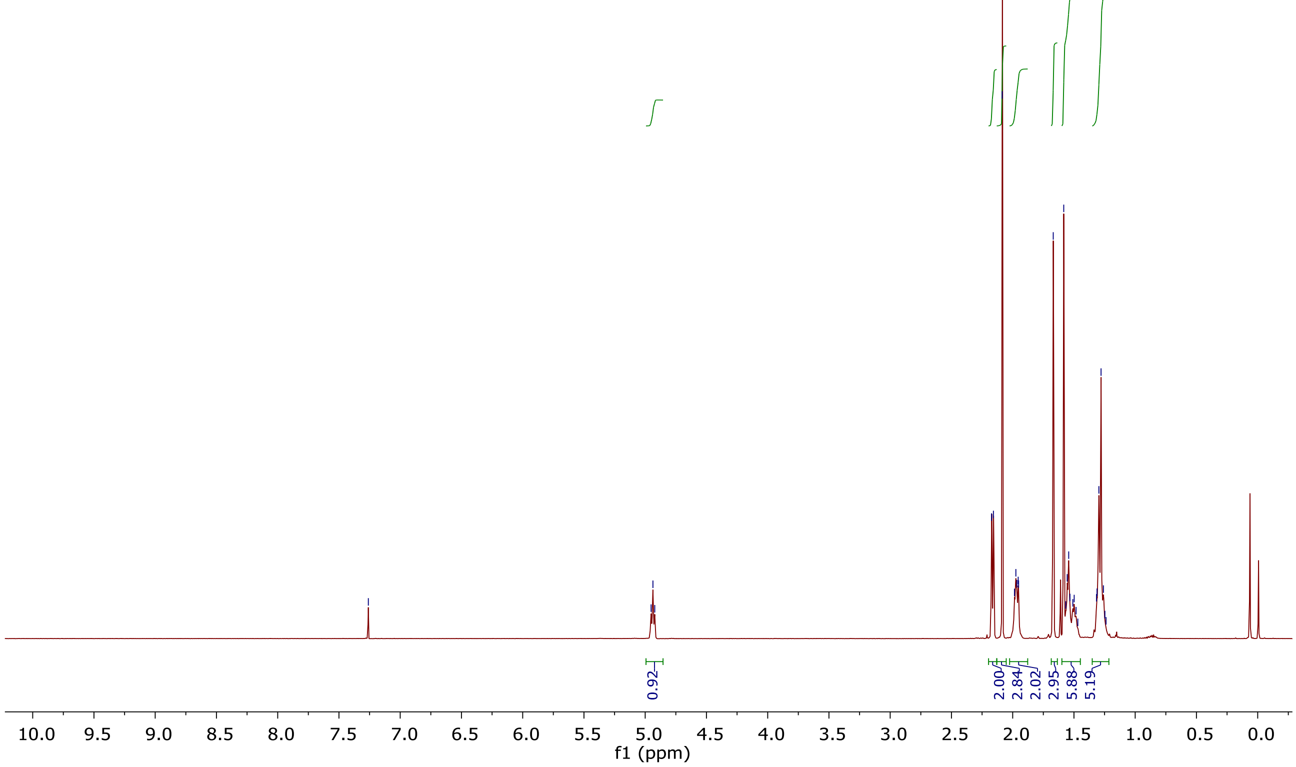

^{13}C NMR
$CDCl_3$
125 MHz

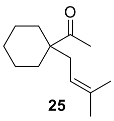

25

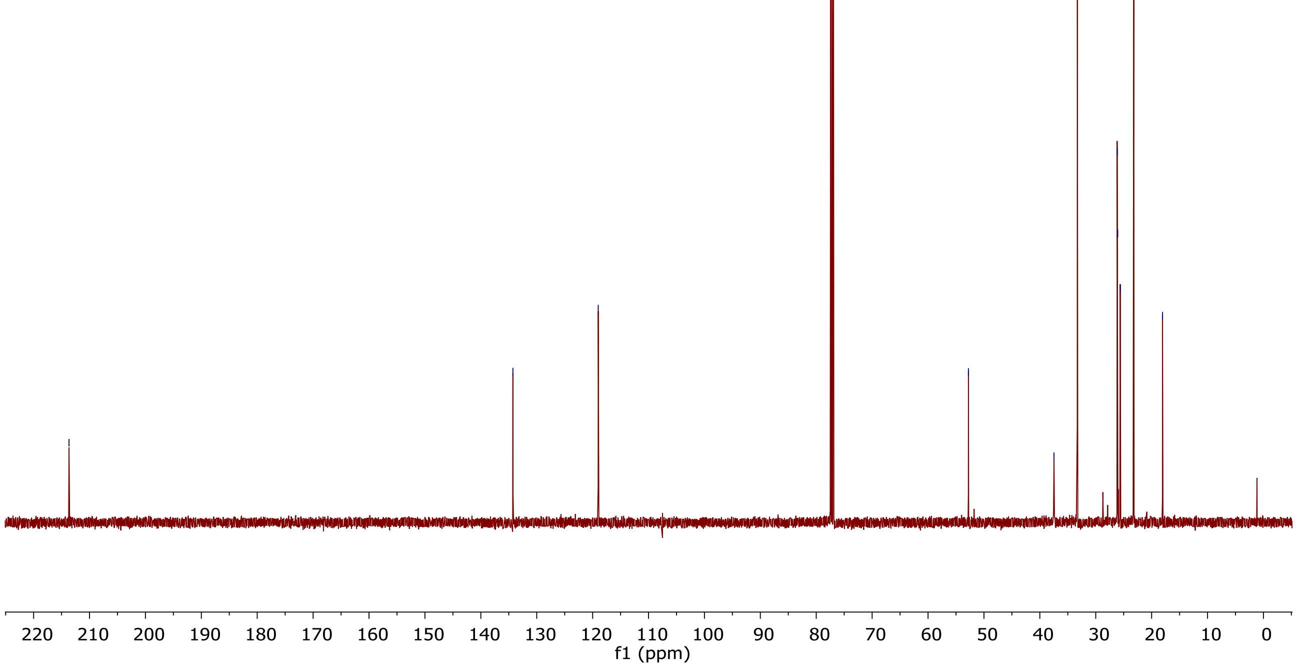

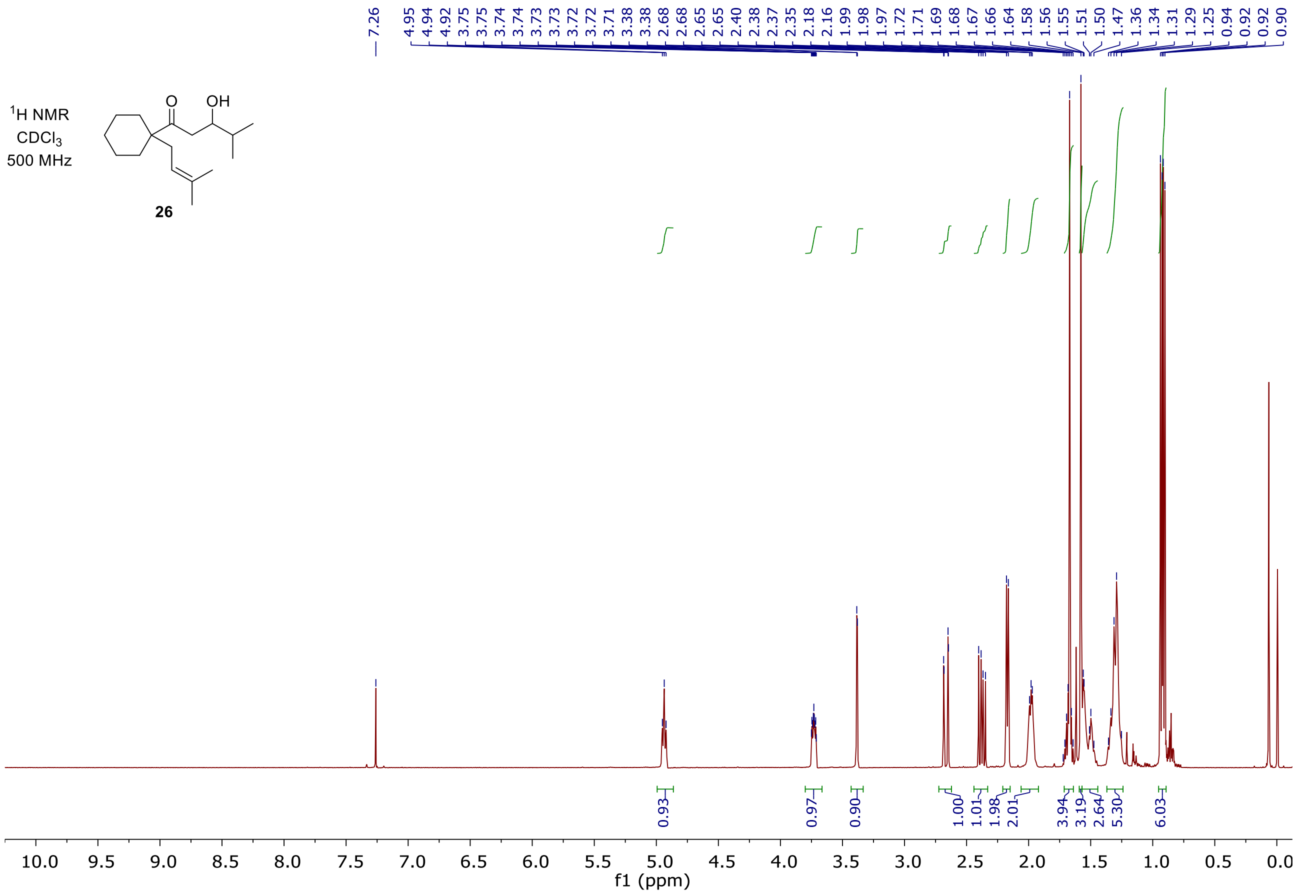

^{1}H NMR
$CDCl_3$
500 MHz

26

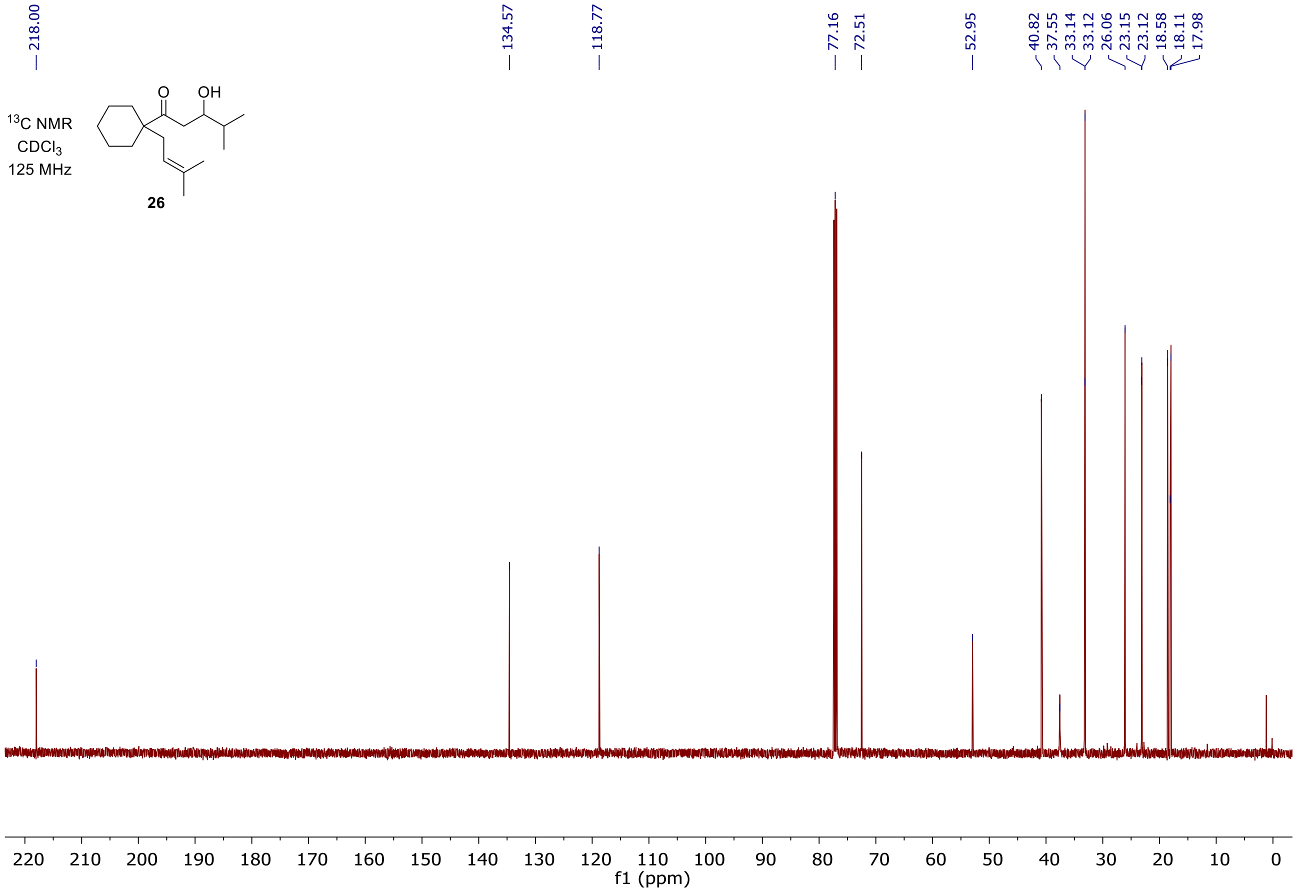

^{13}C NMR
$CDCl_3$
125 MHz

26

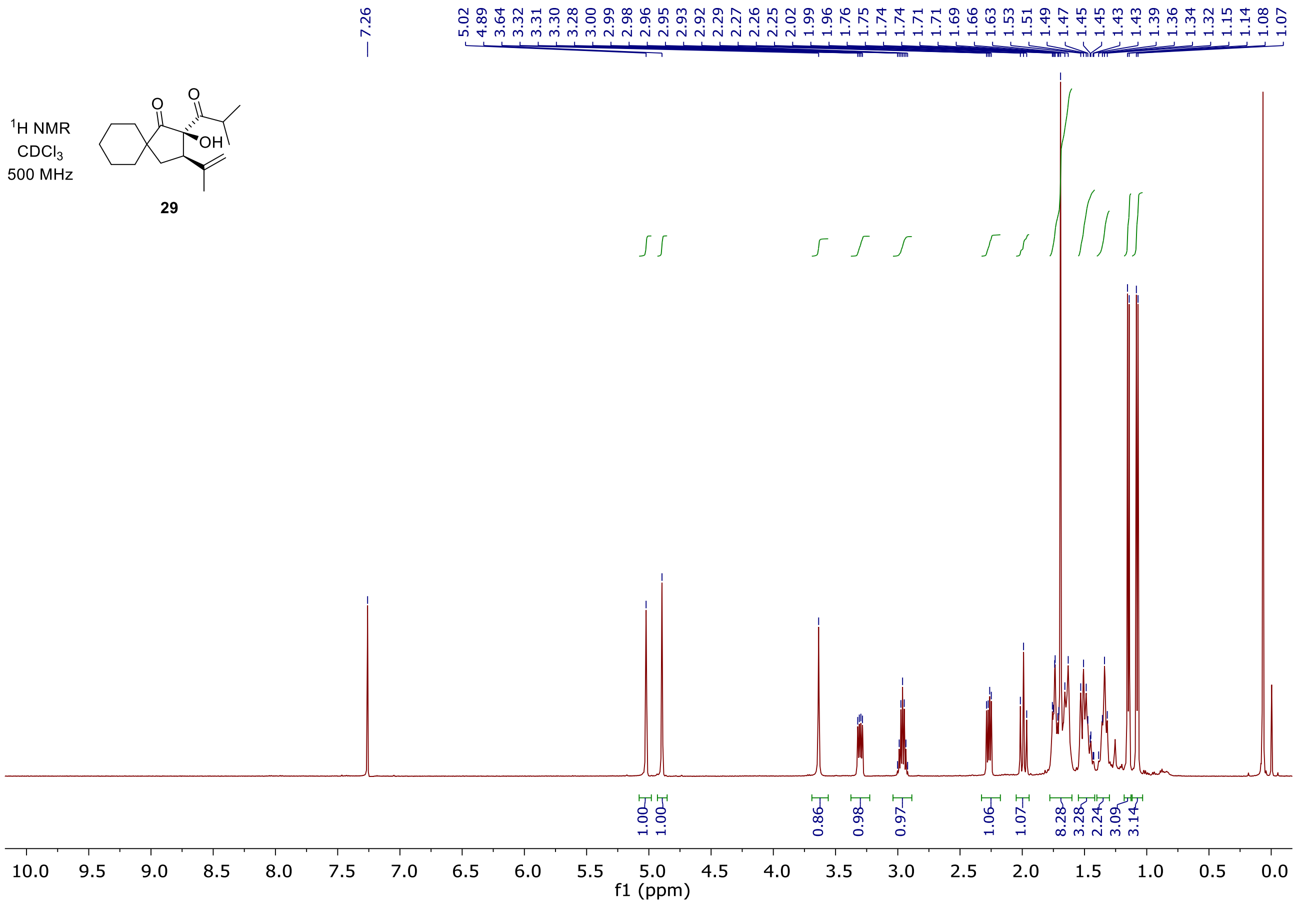

¹H NMR
CDCl3
500 MHz
29
f1 (ppm)

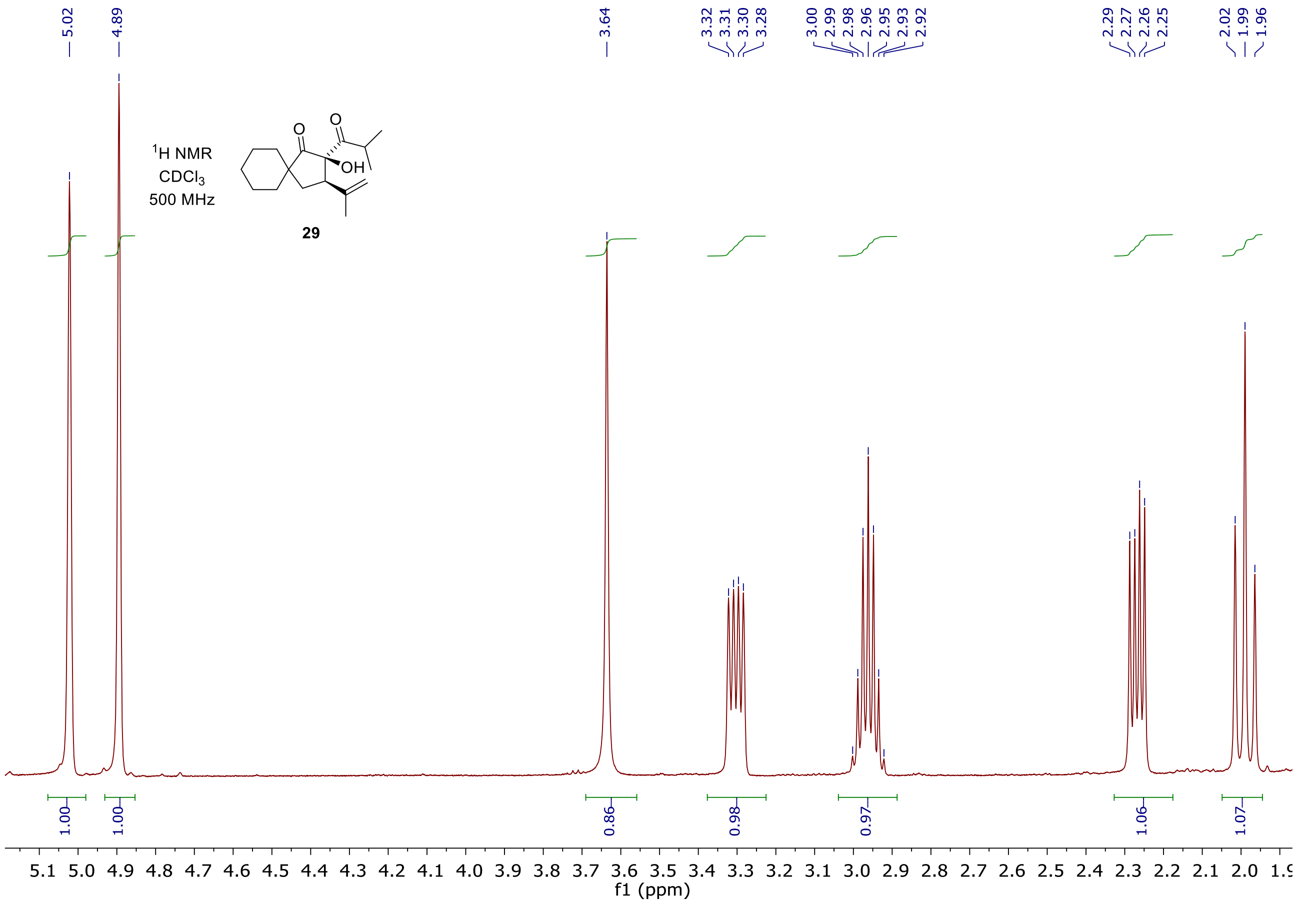

¹H NMR
CDCl3
500 MHz
29
f1 (ppm)

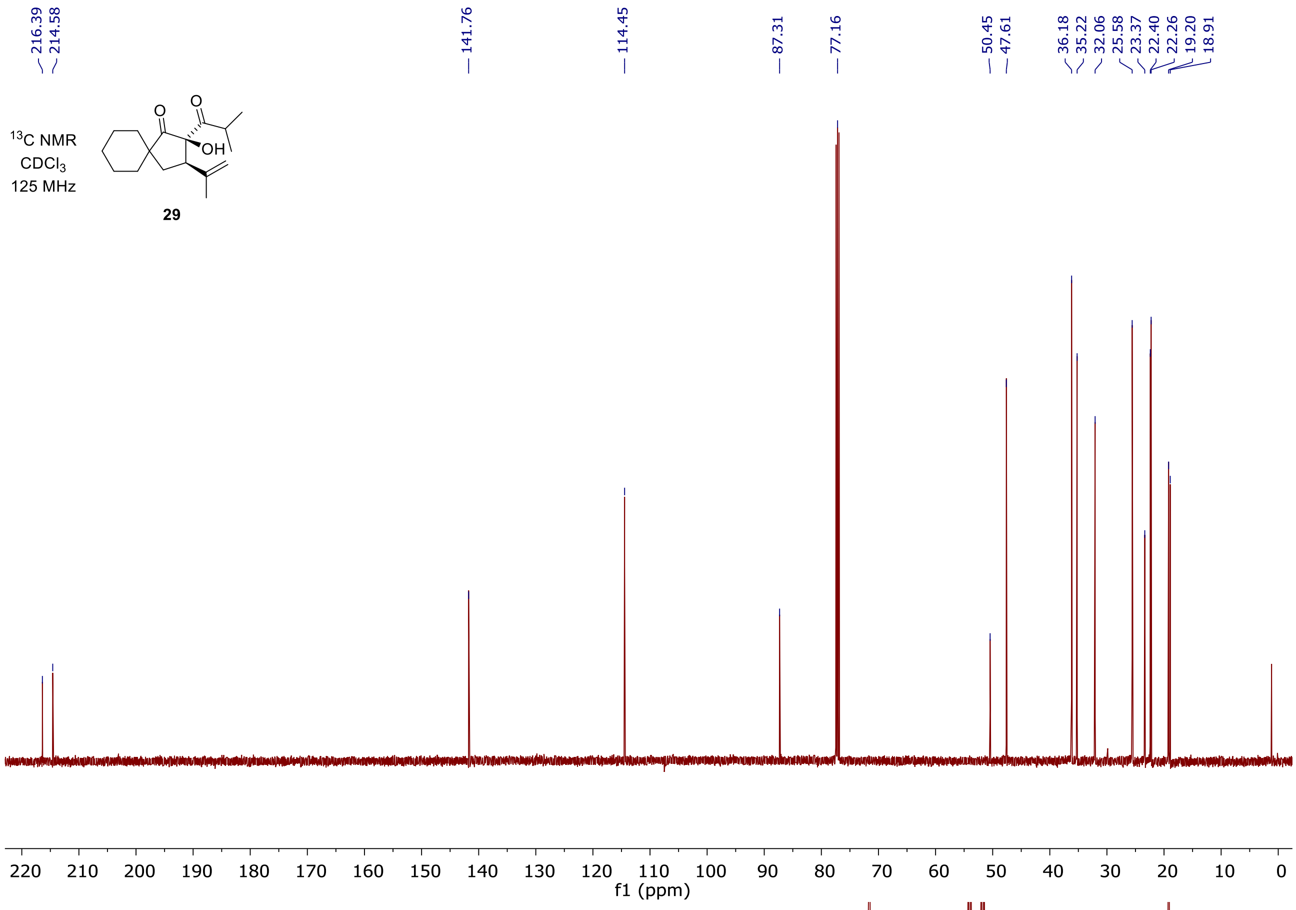
216.39
214.58
141.76
114.45
87.31
77.16
50.45
47.61
36.18
35.22
32.06
25.58
23.37
22.40
22.26
19.20
18.91
13C NMR
CDCl3
125 MHz
29
f1 (ppm)

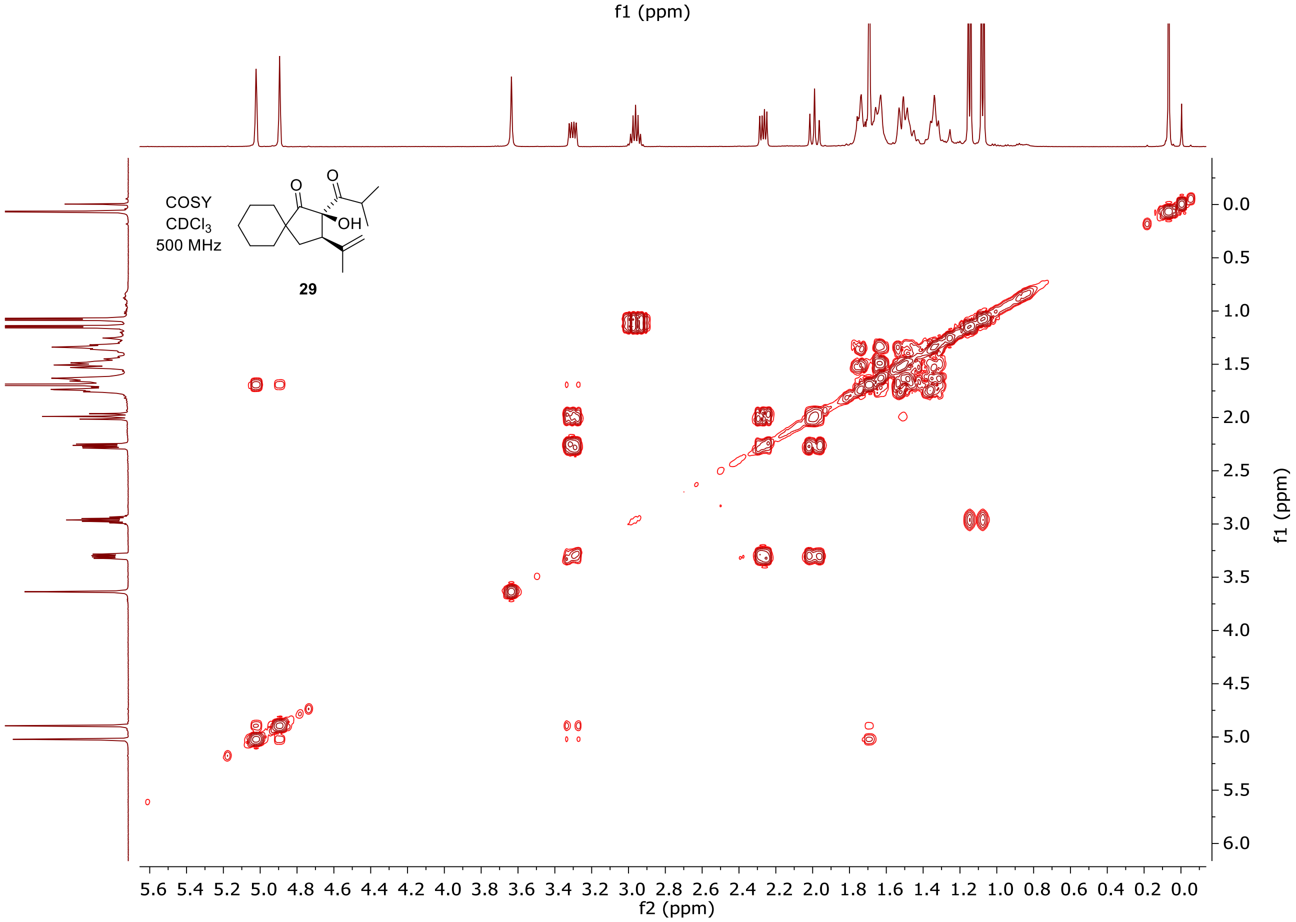
COSY
CDCl3
500 MHz
29
f2 (ppm)
f1 (ppm)

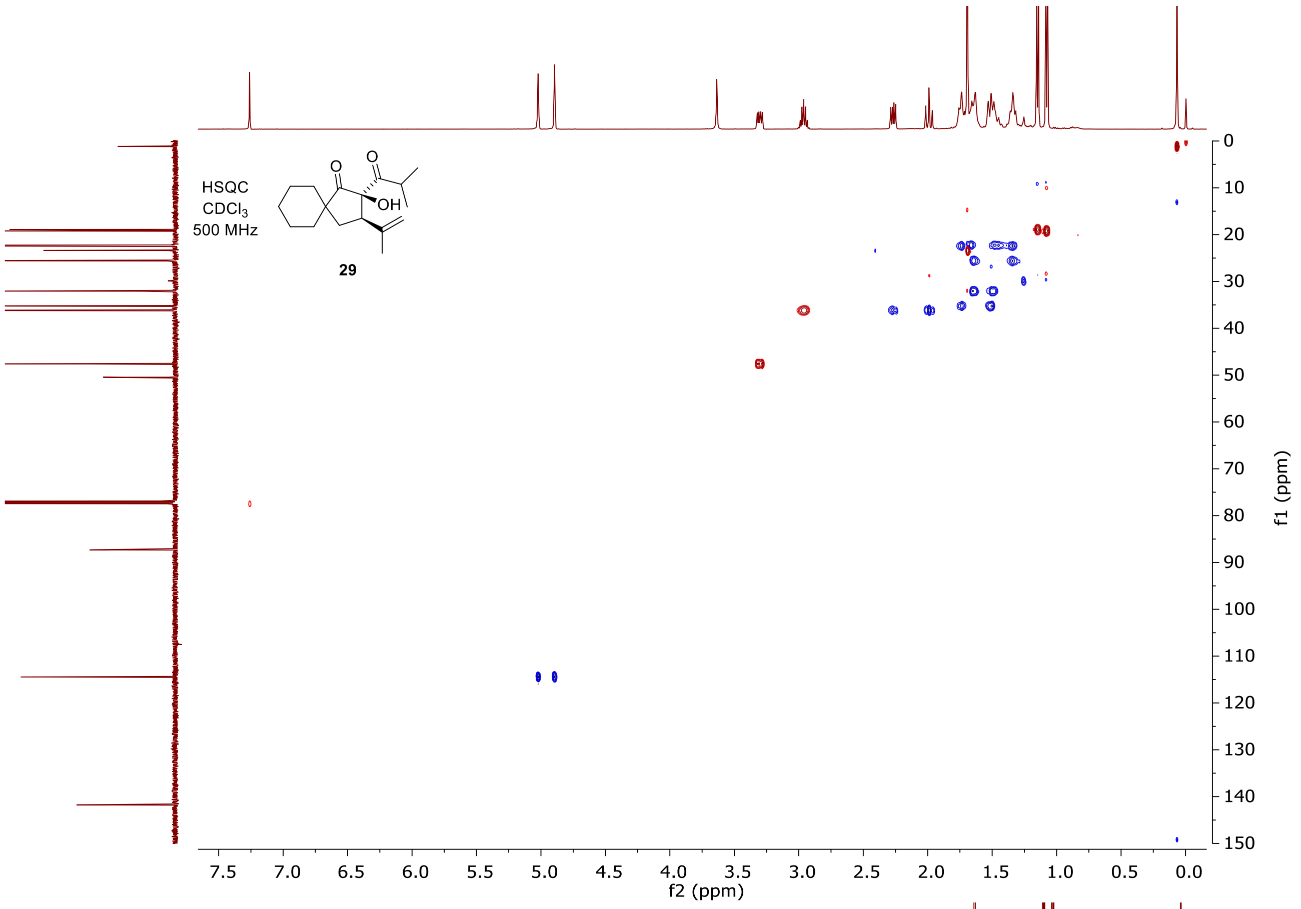
HSQC
CDCl3
500 MHz
29
f2 (ppm)
f1 (ppm)

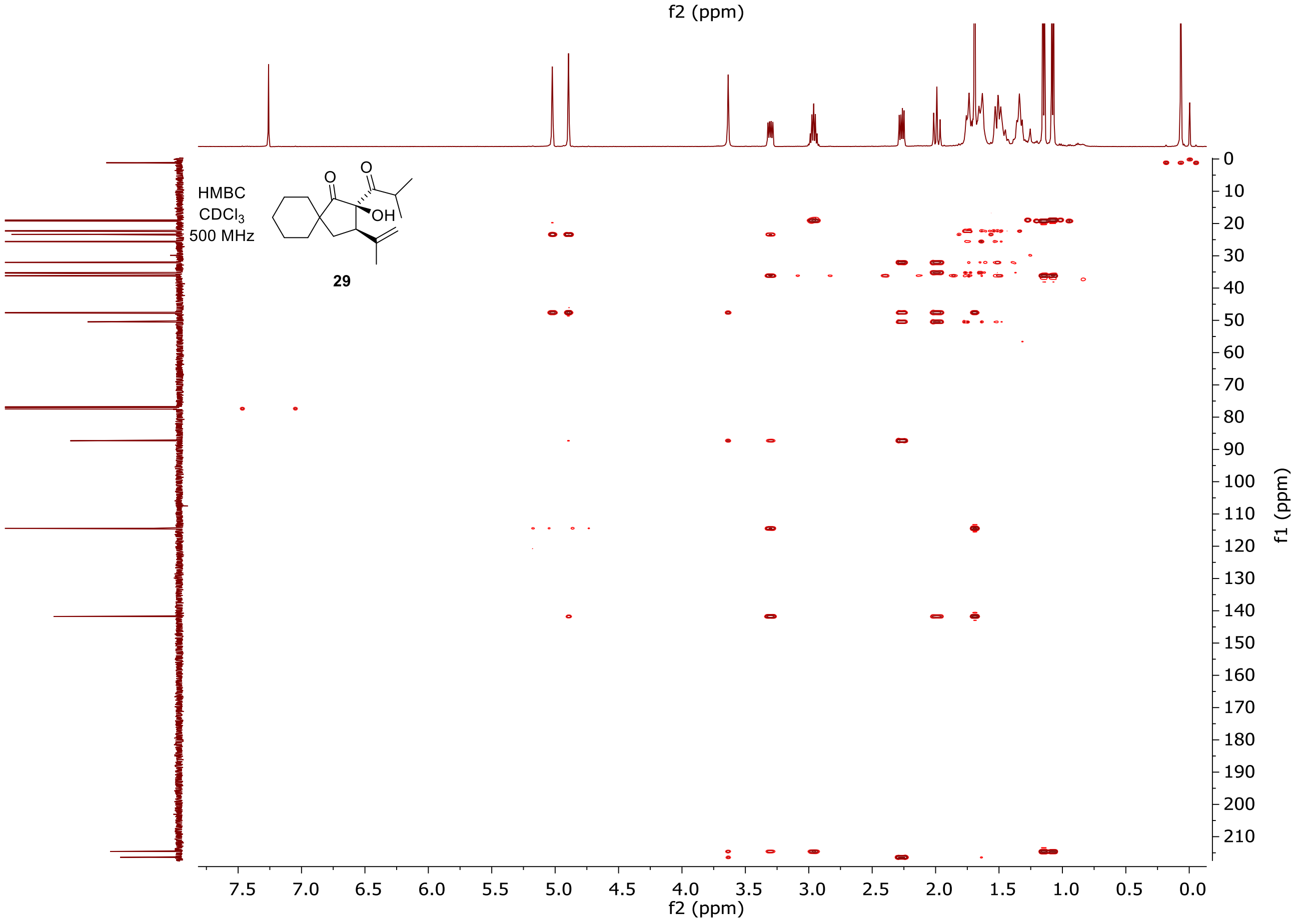
HMBC
CDCl3
500 MHz
29
f2 (ppm)
f1 (ppm)

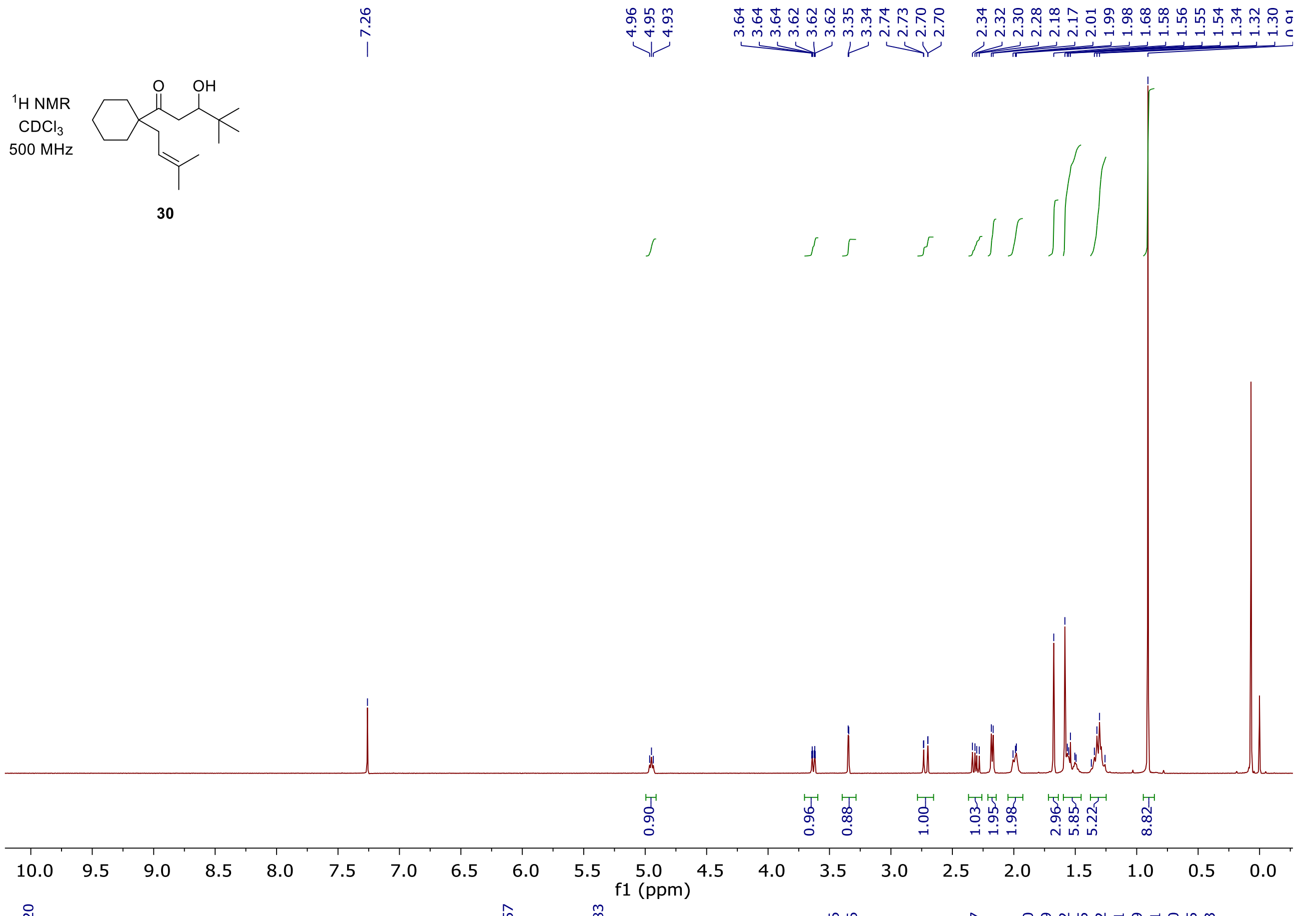

1H NMR
CDCl3
500 MHz
30
7.26
4.96 4.95 4.93
3.64 3.64 3.64 3.62 3.62 3.62 3.35 3.34 2.74 2.73 2.70 2.70
2.34 2.32 2.30 2.28 2.18 2.17 2.01 1.99 1.98 1.68 1.58 1.56 1.55 1.54 1.34 1.32 1.30
0.90 0.96 0.88 1.00 1.03 1.95 1.98 2.96 5.85 5.22 8.82
10.0 9.5 9.0 8.5 8.0 7.5 7.0 6.5 6.0 5.5 5.0 4.5 4.0 3.5 3.0 2.5 2.0 1.5 1.0 0.5 0.0
f1 (ppm)

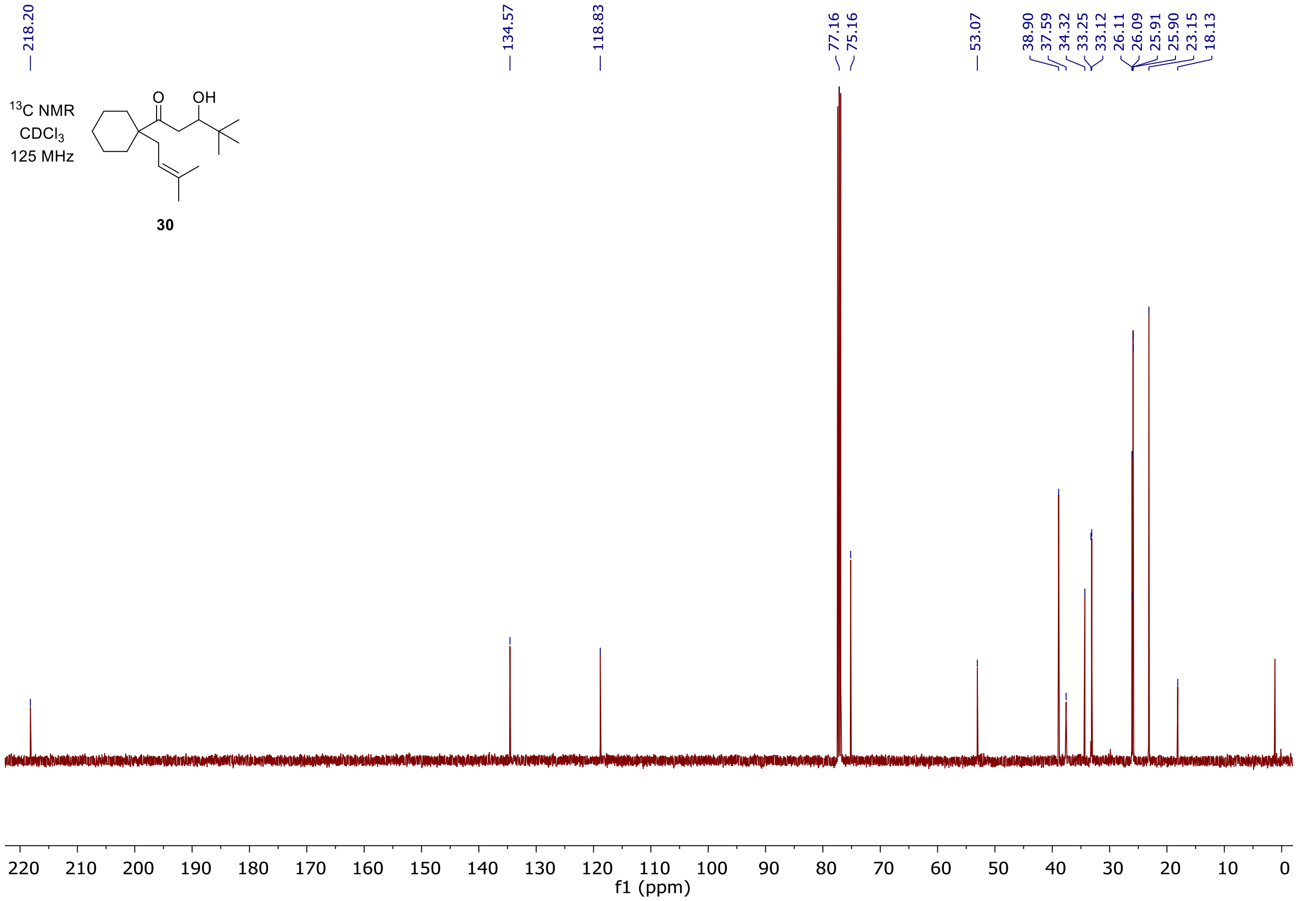

13C NMR
CDCl3
125 MHz
30
218.20
134.57
118.83
77.16 75.16
53.07
38.90 37.59 34.32 33.25 33.12 26.11 26.09 25.91 25.90 23.15 18.13
220 210 200 190 180 170 160 150 140 130 120 110 100 90 80 70 60 50 40 30 20 10 0
f1 (ppm)

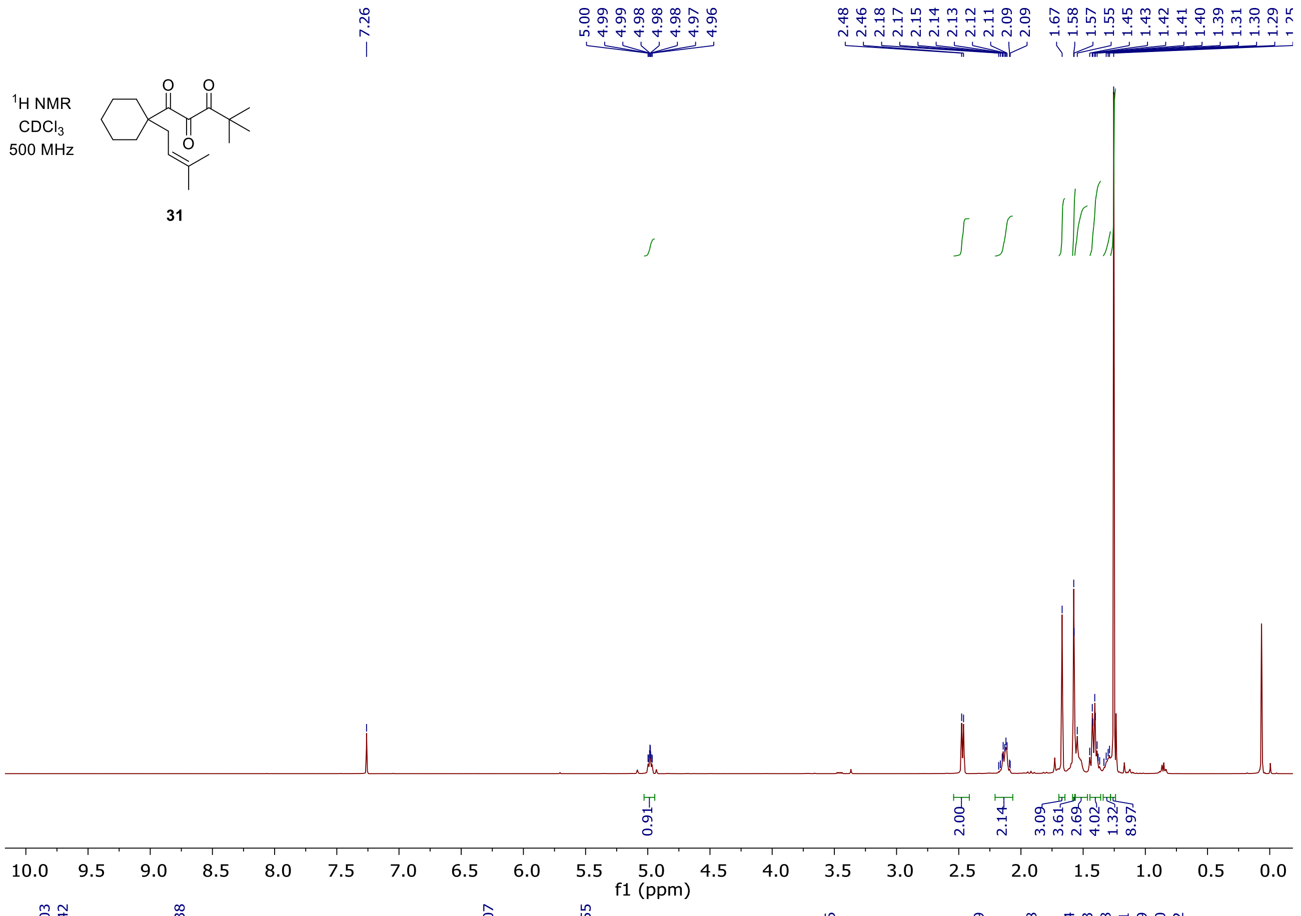

1H NMR
CDCl3
500 MHz
31
7.26
5.00
4.99
4.99
4.98
4.98
4.98
4.97
4.96
2.48
2.46
2.18
2.17
2.15
2.14
2.13
2.12
2.11
2.09
2.09
1.67
1.58
1.57
1.55
1.45
1.43
1.42
1.41
1.40
1.39
1.31
1.30
1.29
0.91
2.00
2.14
3.09
3.61
2.69
4.02
1.32
8.97
10.0 9.5 9.0 8.5 8.0 7.5 7.0 6.5 6.0 5.5 5.0 4.5 4.0 3.5 3.0 2.5 2.0 1.5 1.0 0.5 0.0
f1 (ppm)

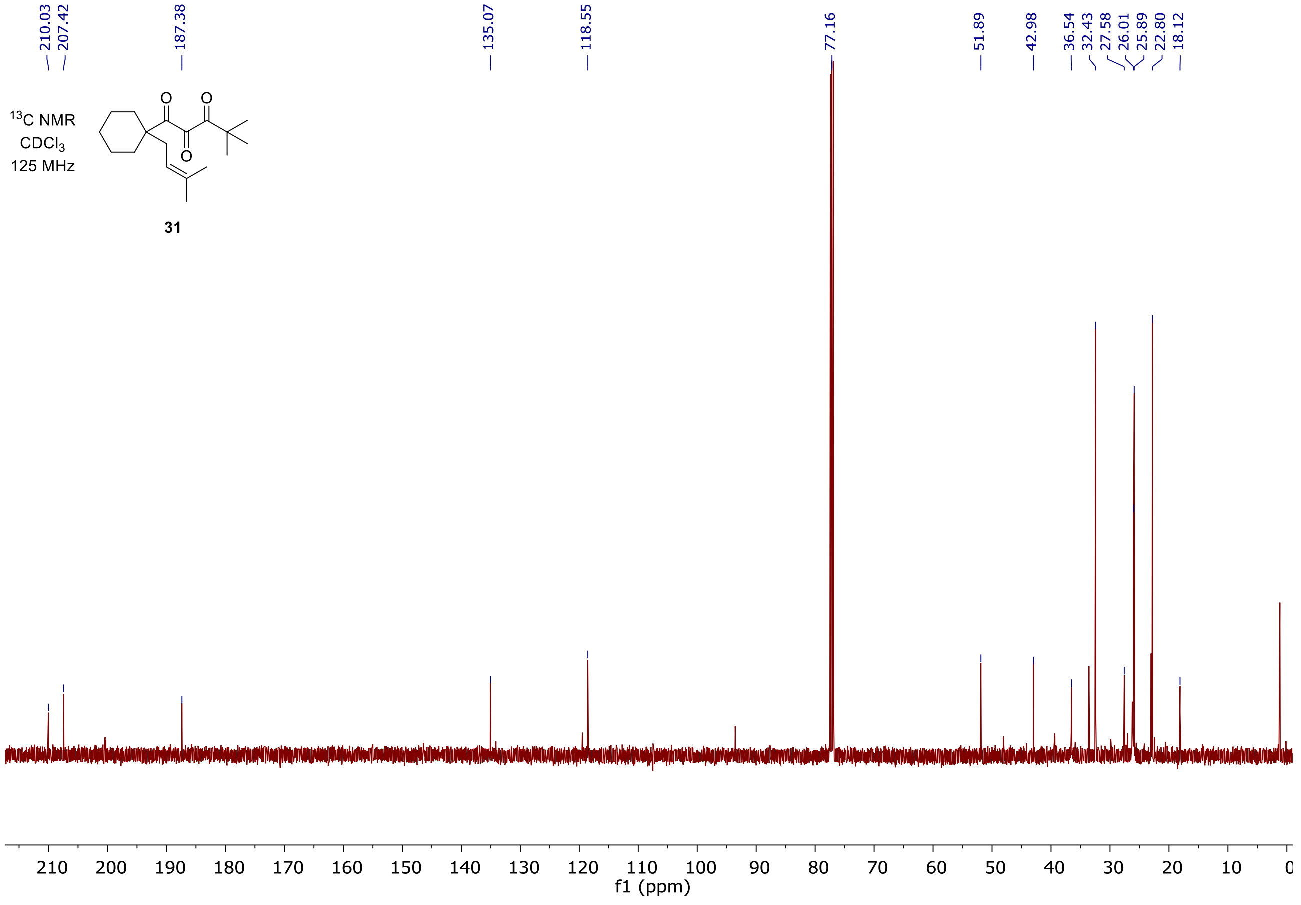

13C NMR
CDCl3
125 MHz
31
210.03
207.42
187.38
135.07
118.55
77.16
51.89
42.98
36.54
32.43
27.58
26.01
25.89
22.80
18.12
210 200 190 180 170 160 150 140 130 120 110 100 90 80 70 60 50 40 30 20 10 0
f1 (ppm)

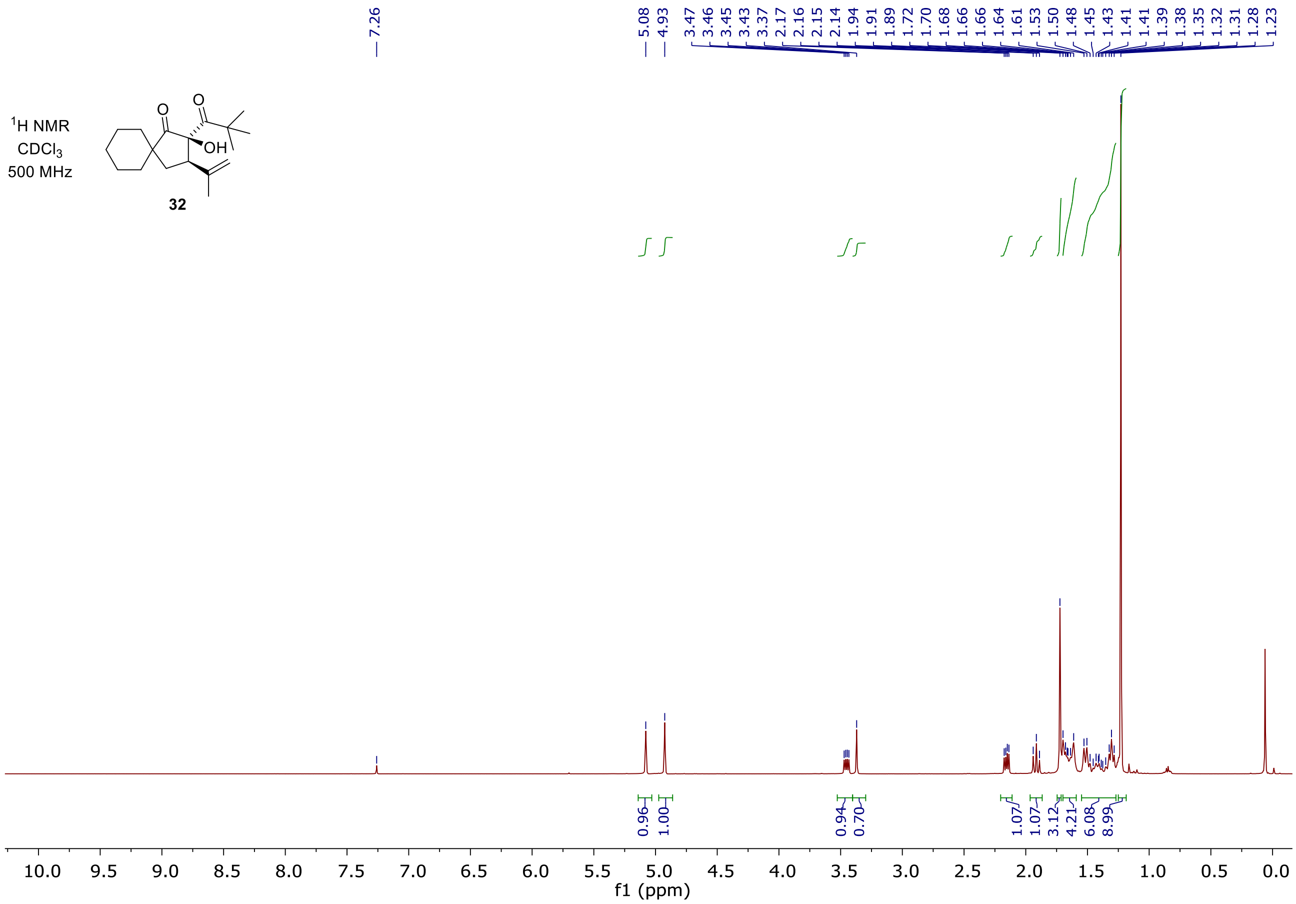

1H NMR
CDCl3
500 MHz
32
7.26
5.08
4.93
3.47
3.46
3.45
3.43
3.37
2.17
2.16
2.15
2.14
1.94
1.91
1.89
1.72
1.70
1.68
1.66
1.66
1.64
1.61
1.53
1.50
1.48
1.45
1.43
1.41
1.41
1.39
1.38
1.35
1.32
1.31
1.28
1.23
0.96
1.00
0.94
0.70
1.07
1.07
3.12
4.21
6.08
8.99
f1 (ppm)

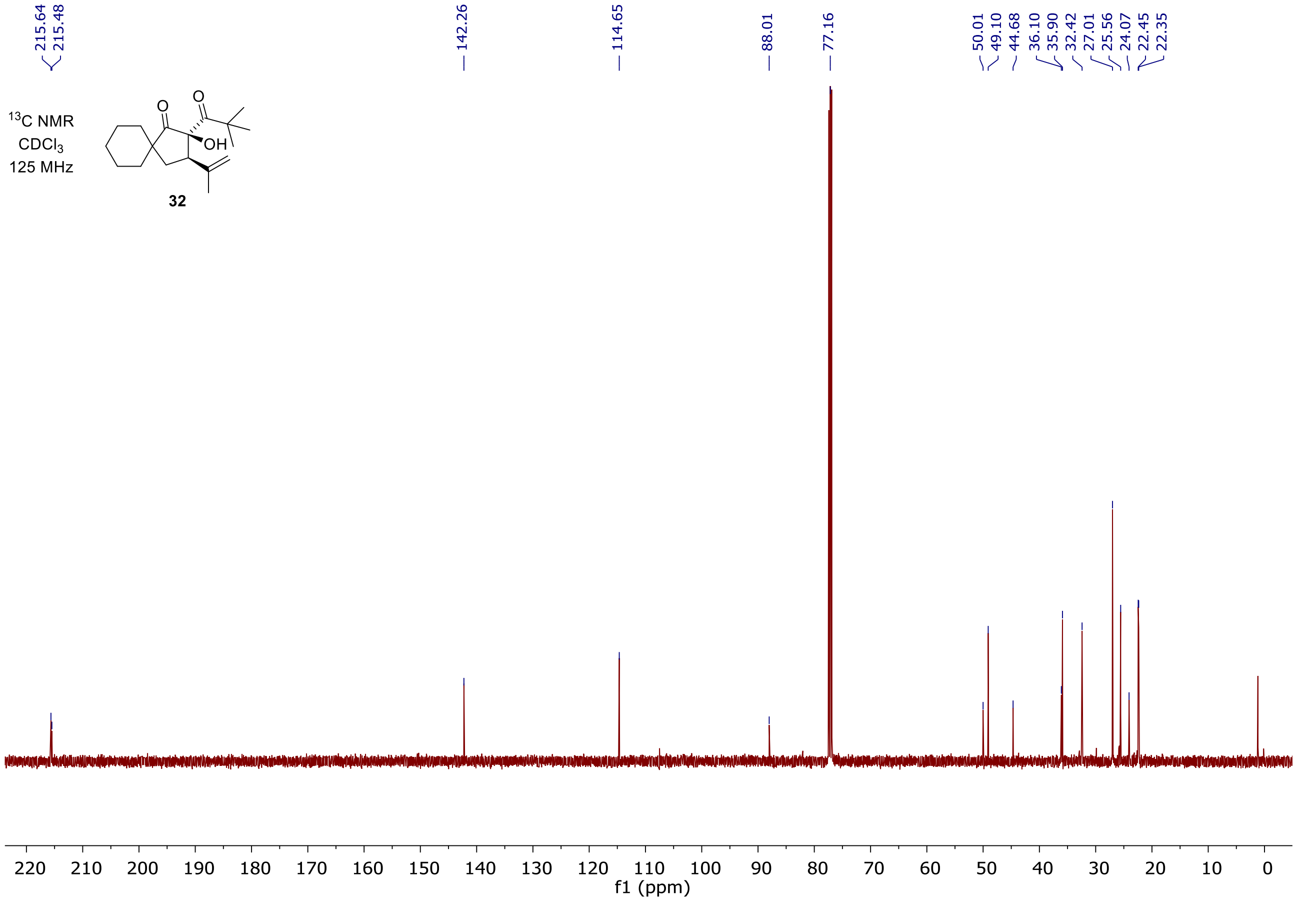

13C NMR
CDCl3
125 MHz
32
215.64
215.48
142.26
114.65
88.01
77.16
50.01
49.10
44.68
36.10
35.90
32.42
27.01
25.56
24.07
22.45
22.35
f1 (ppm)

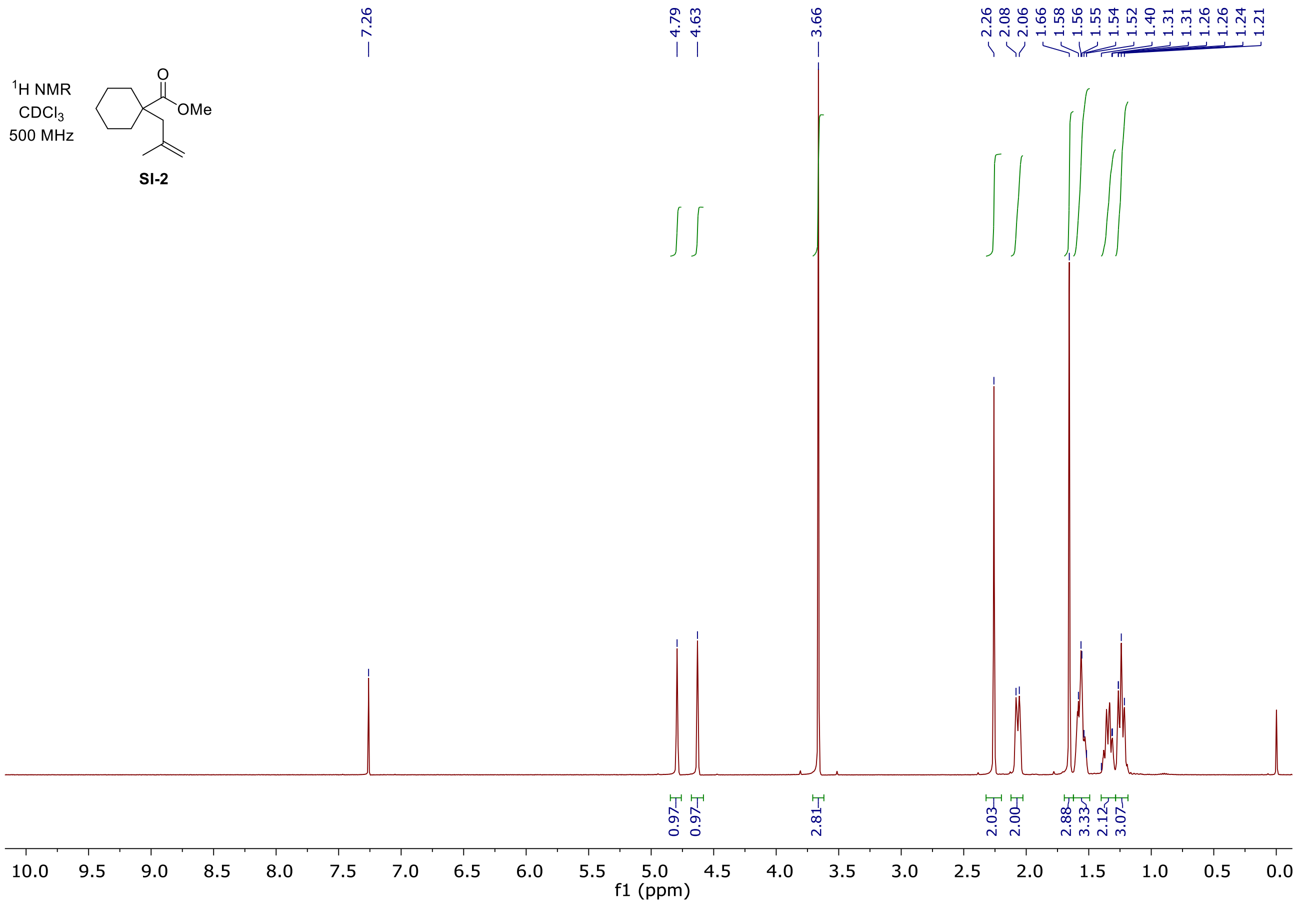

^{1}H NMR
CDCl$_3$
500 MHz
SI-2
O
OMe
7.26
4.79
4.63
3.66
2.26
2.08
2.06
1.66
1.58
1.56
1.55
1.54
1.52
1.40
1.31
1.31
1.26
1.26
1.24
1.21
0.97
0.97
2.81
2.03
2.00
2.88
3.33
2.12
3.07
f1 (ppm)

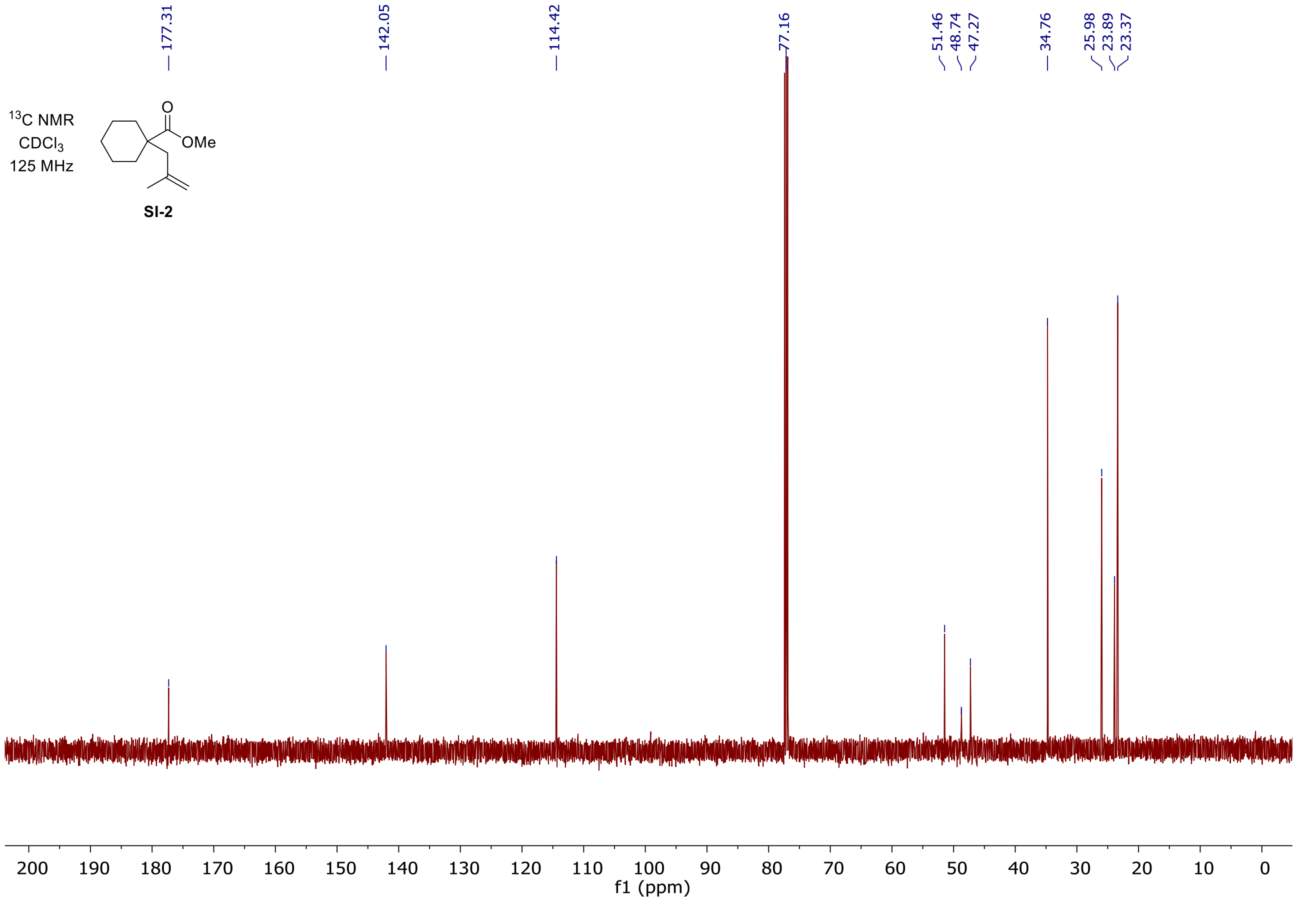

^{13}C NMR
CDCl$_3$
125 MHz
SI-2
O
OMe
177.31
142.05
114.42
77.16
51.46
48.74
47.27
34.76
25.98
23.89
23.37
f1 (ppm)

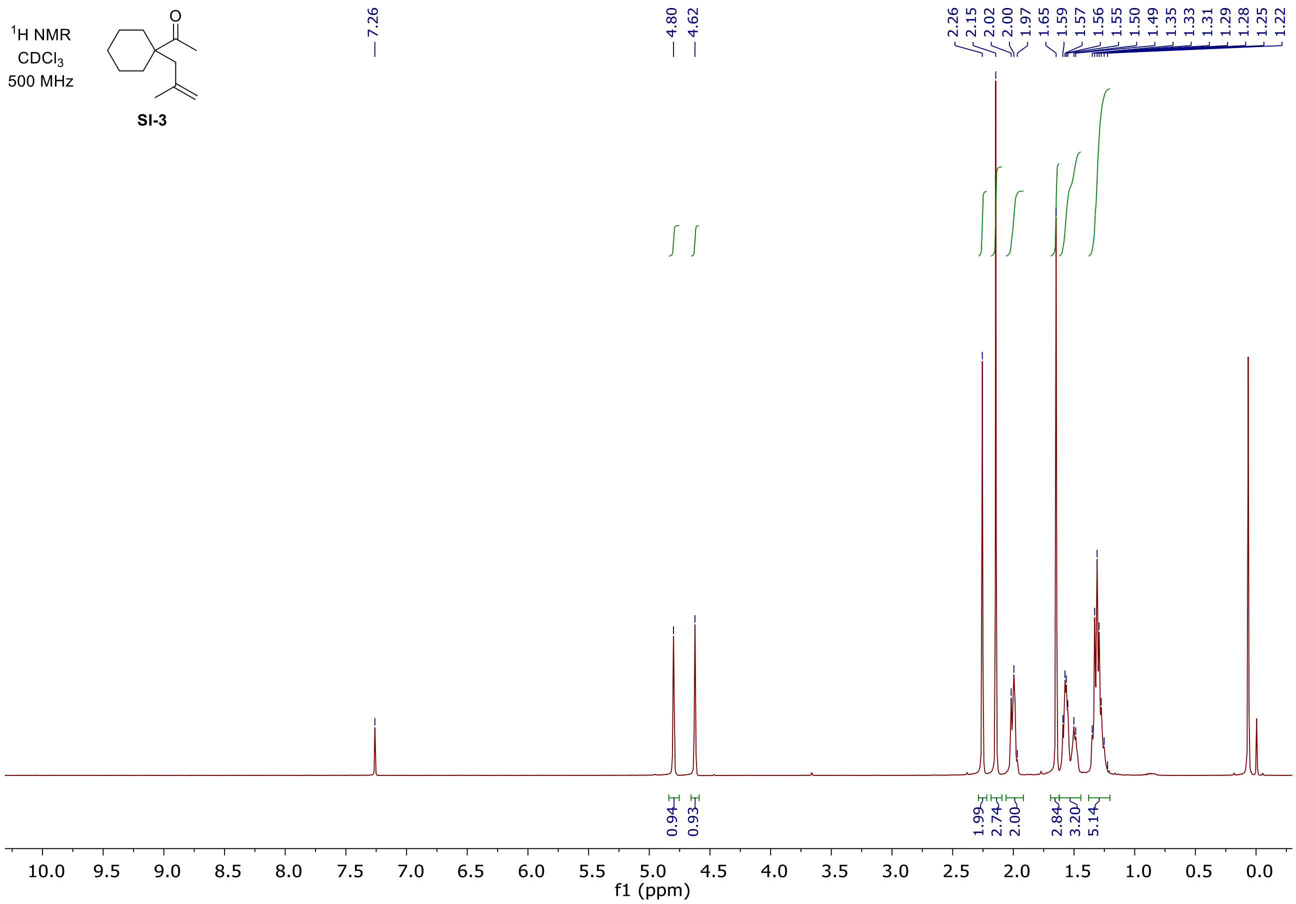

1H NMR
CDCl3
500 MHz
SI-3
7.26
4.80
4.62
2.26
2.15
2.02
2.00
1.97
1.65
1.59
1.57
1.56
1.55
1.50
1.49
1.35
1.33
1.31
1.29
1.28
1.25
1.22
0.94
0.93
1.99
2.74
2.00
2.84
3.20
5.14
f1 (ppm)

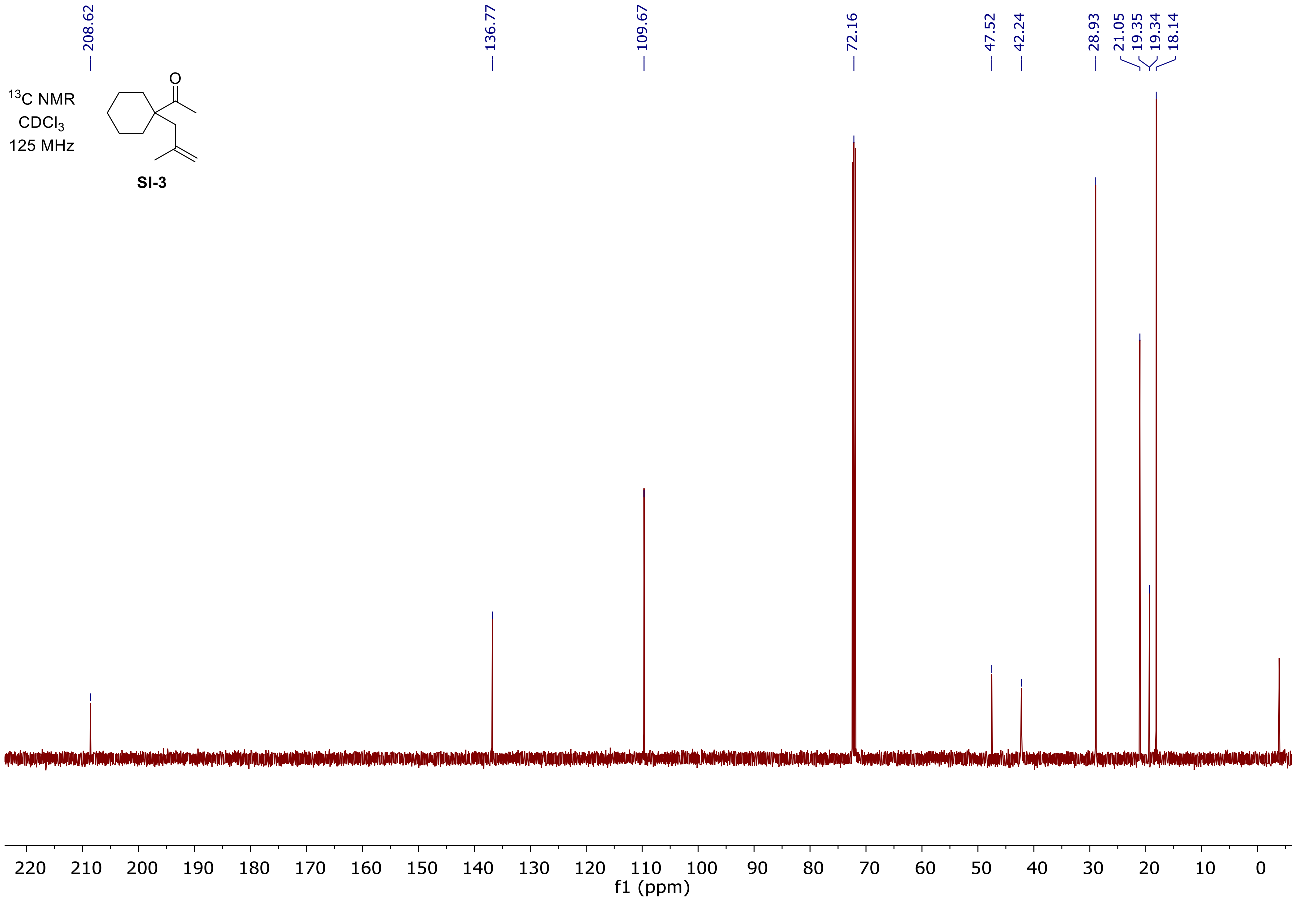

13C NMR
CDCl3
125 MHz
SI-3
208.62
136.77
109.67
72.16
47.52
42.24
28.93
21.05
19.35
19.34
18.14
f1 (ppm)

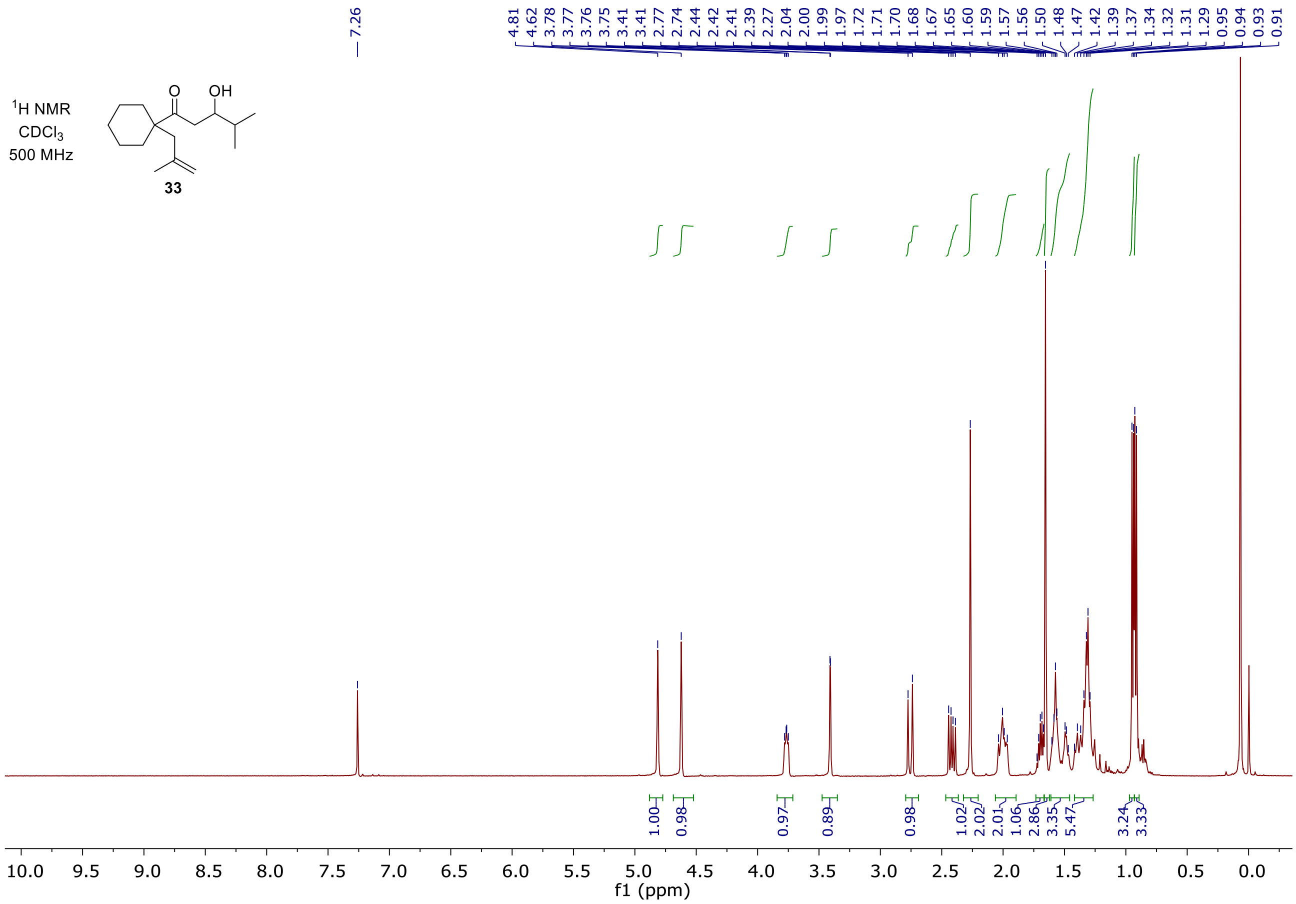

1H NMR
CDCl3
500 MHz
O
OH
33
7.26
4.81 4.62 3.78 3.77 3.76 3.75 3.41 3.41 2.77 2.74 2.44 2.42 2.41 2.39 2.27 2.04 2.00 1.99 1.97 1.72 1.71 1.70 1.68 1.67 1.65 1.60 1.59 1.57 1.56 1.50 1.48 1.47 1.42 1.39 1.37 1.34 1.32 1.31 1.29 0.95 0.94 0.93 0.91
1.00 0.98 0.97 0.89 0.98 1.02 2.02 2.01 1.06 2.86 3.35 5.47 3.24 3.33
10.0 9.5 9.0 8.5 8.0 7.5 7.0 6.5 6.0 5.5 5.0 4.5 4.0 3.5 3.0 2.5 2.0 1.5 1.0 0.5 0.0
f1 (ppm)

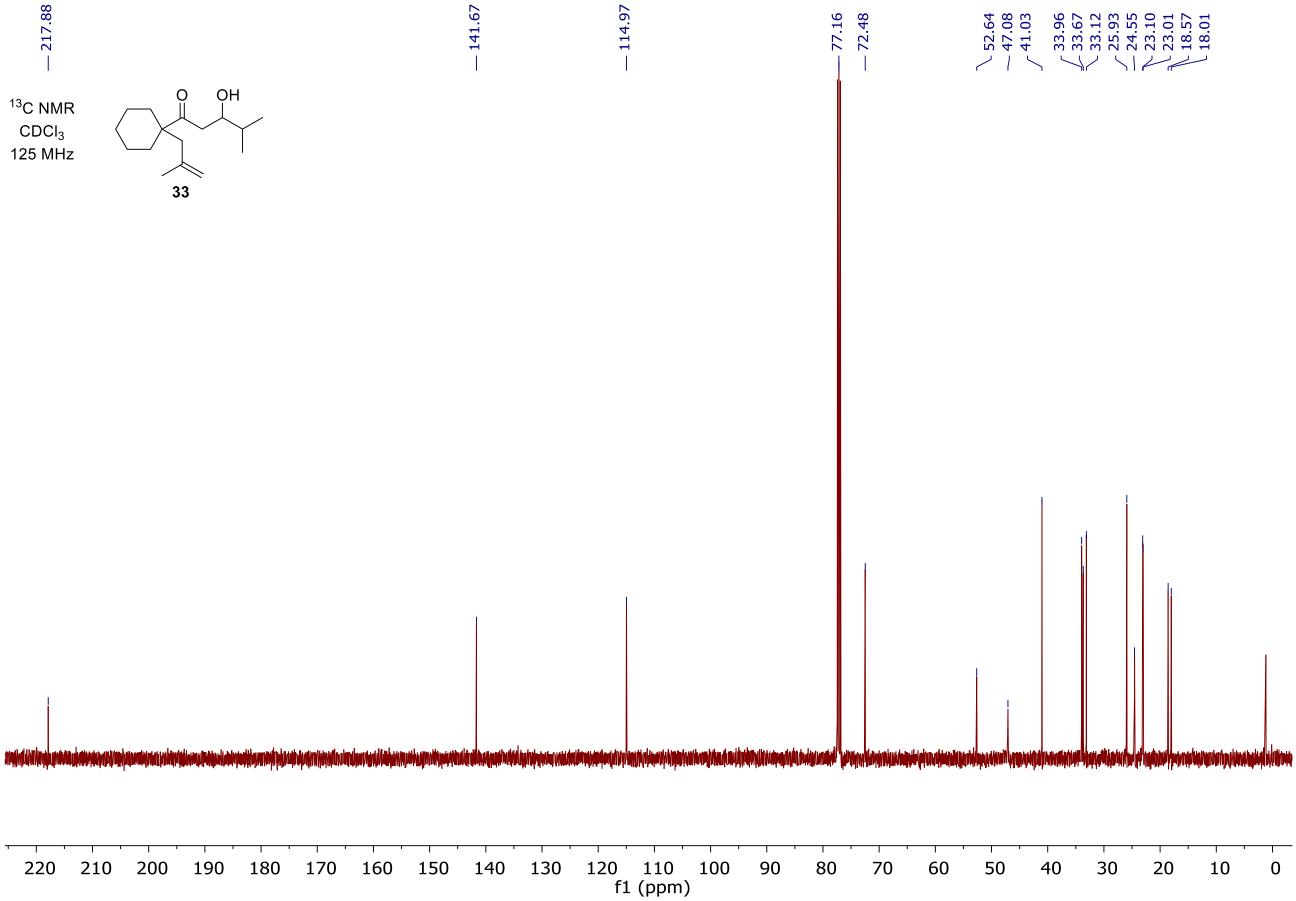

217.88
141.67
114.97
77.16
72.48
52.64 47.08 41.03 33.96 33.67 33.12 25.93 24.55 23.10 23.01 18.57 18.01
13C NMR
CDCl3
125 MHz
O
OH
33
220 210 200 190 180 170 160 150 140 130 120 110 100 90 80 70 60 50 40 30 20 10 0
f1 (ppm)

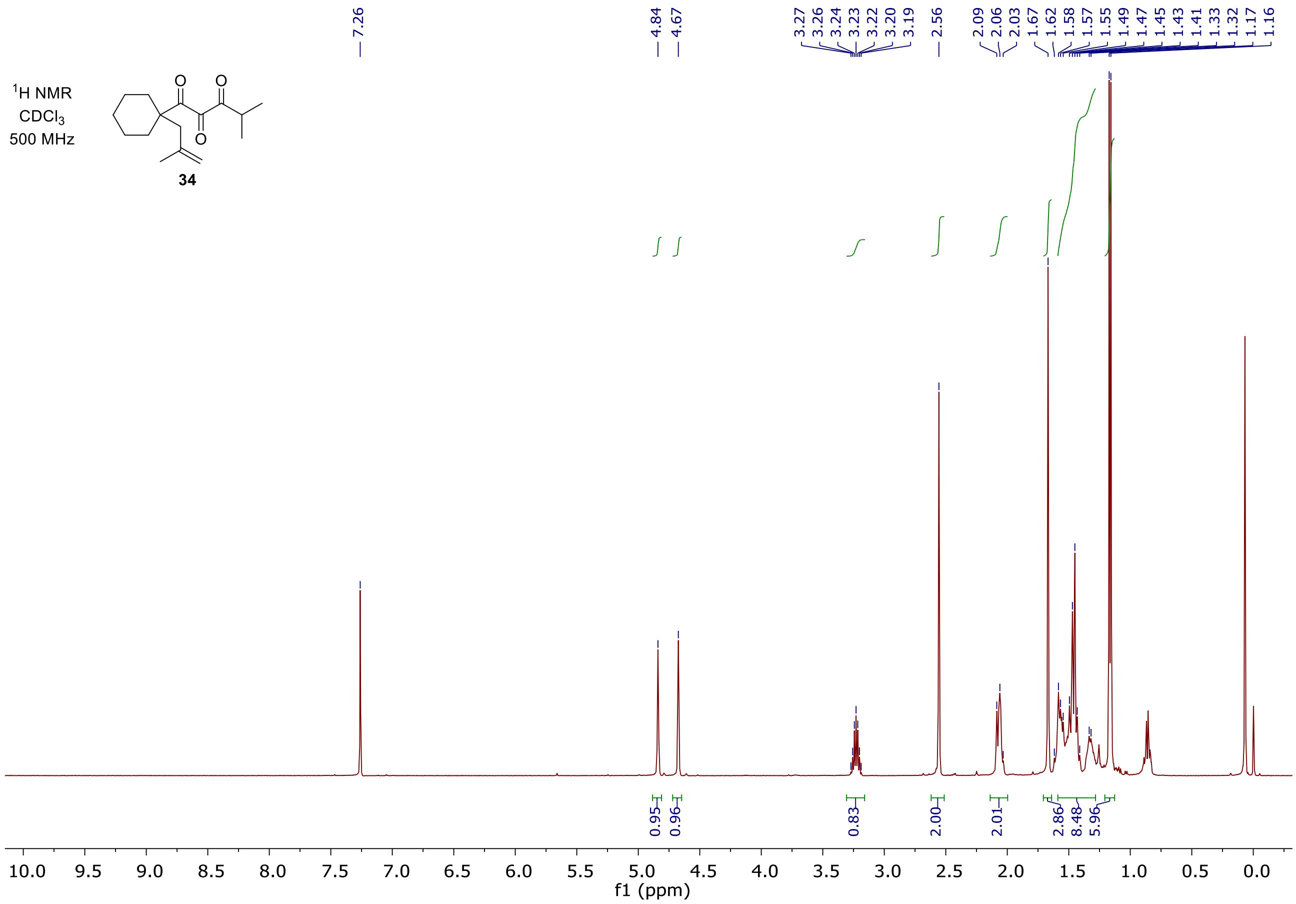

1H NMR
CDCl3
500 MHz
34
7.26
4.84
4.67
3.27
3.26
3.24
3.23
3.22
3.20
3.19
2.56
2.09
2.06
2.03
1.67
1.62
1.58
1.57
1.55
1.49
1.47
1.45
1.43
1.41
1.33
1.32
1.17
1.16
0.95
0.96
0.83
2.00
2.01
2.86
8.48
5.96
f1 (ppm)

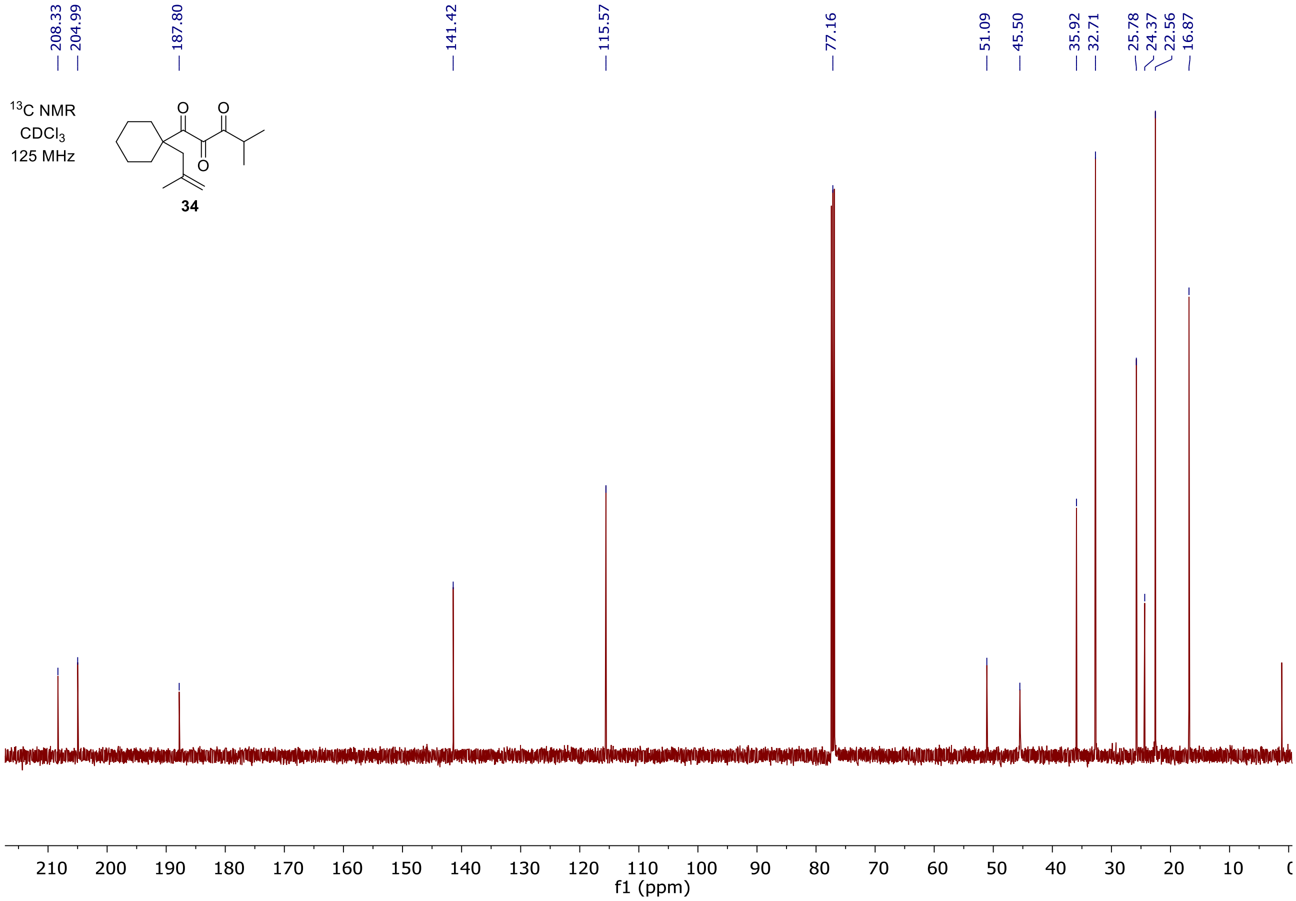

13C NMR
CDCl3
125 MHz
34
208.33
204.99
187.80
141.42
115.57
77.16
51.09
45.50
35.92
32.71
25.78
24.37
22.56
16.87
f1 (ppm)

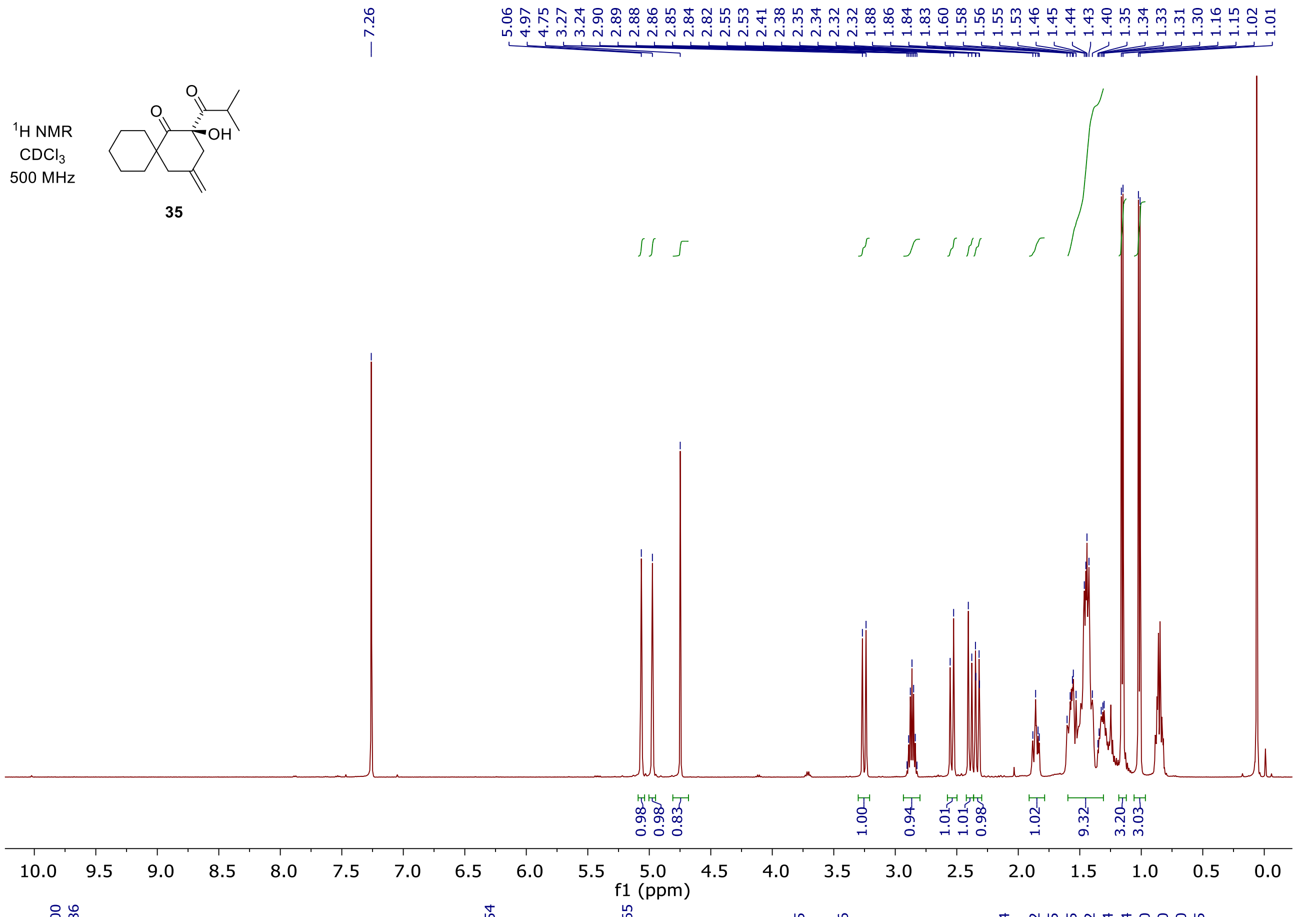
1H NMR
CDCl3
500 MHz
35
7.26
5.06 4.97 4.75 3.27 3.24 2.90 2.89 2.88 2.86 2.85 2.84 2.82 2.55 2.53 2.41 2.38 2.35 2.34 2.32 2.32 1.88 1.86 1.84 1.83 1.60 1.58 1.56 1.55 1.53 1.46 1.45 1.44 1.43 1.40 1.35 1.34 1.33 1.31 1.30 1.16 1.15 1.02 1.01
0.98 0.98 0.83 1.00 0.94 1.01 1.01 0.98 1.02 9.32 3.20 3.03
10.0 9.5 9.0 8.5 8.0 7.5 7.0 6.5 6.0 5.5 5.0 4.5 4.0 3.5 3.0 2.5 2.0 1.5 1.0 0.5 0.0
f1 (ppm)

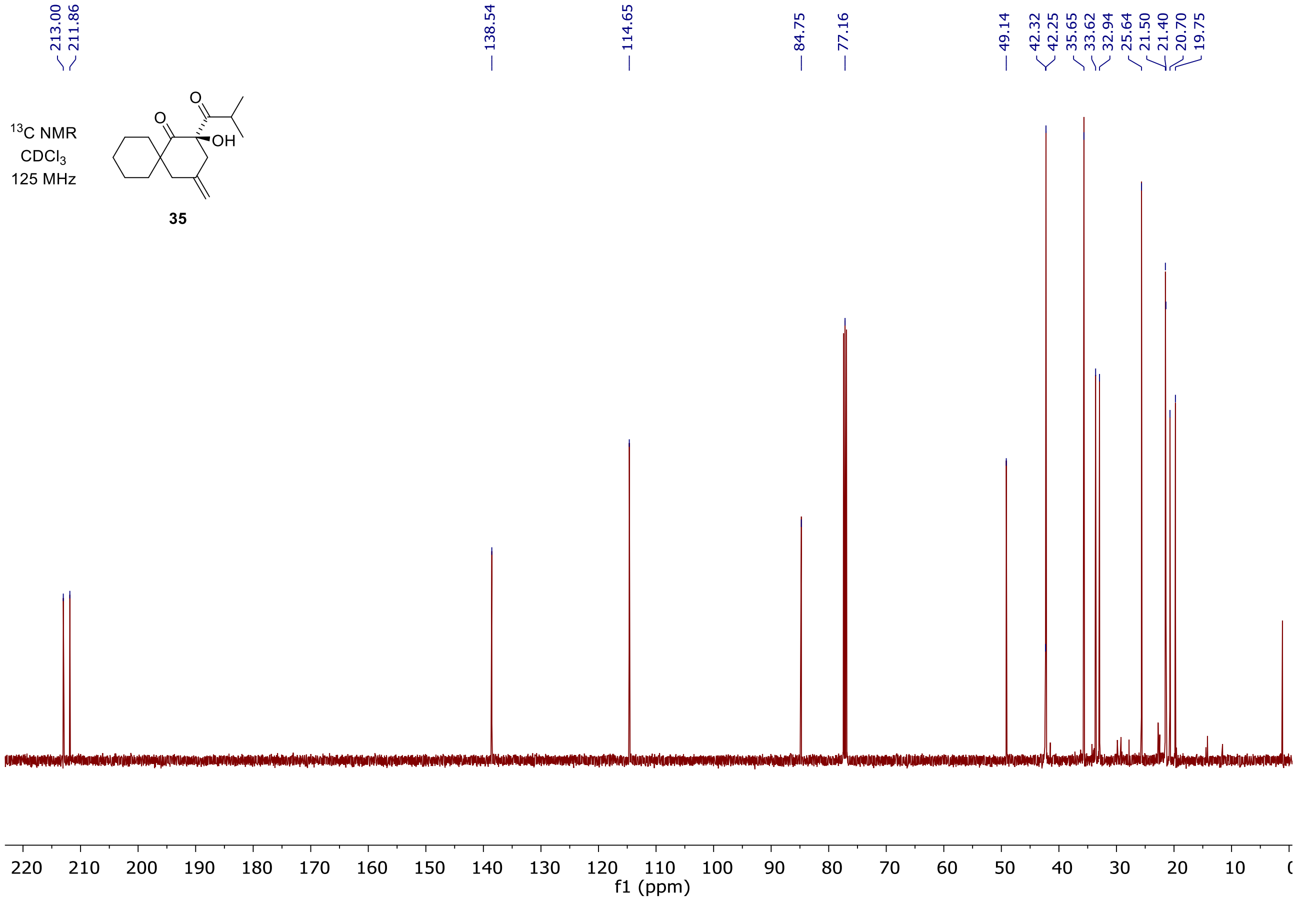
13C NMR
CDCl3
125 MHz
35
213.00 211.86
138.54
114.65
84.75
77.16
49.14 42.32 42.25 35.65 33.62 32.94 25.64 21.50 21.40 20.70 19.75
220 210 200 190 180 170 160 150 140 130 120 110 100 90 80 70 60 50 40 30 20 10 0
f1 (ppm)

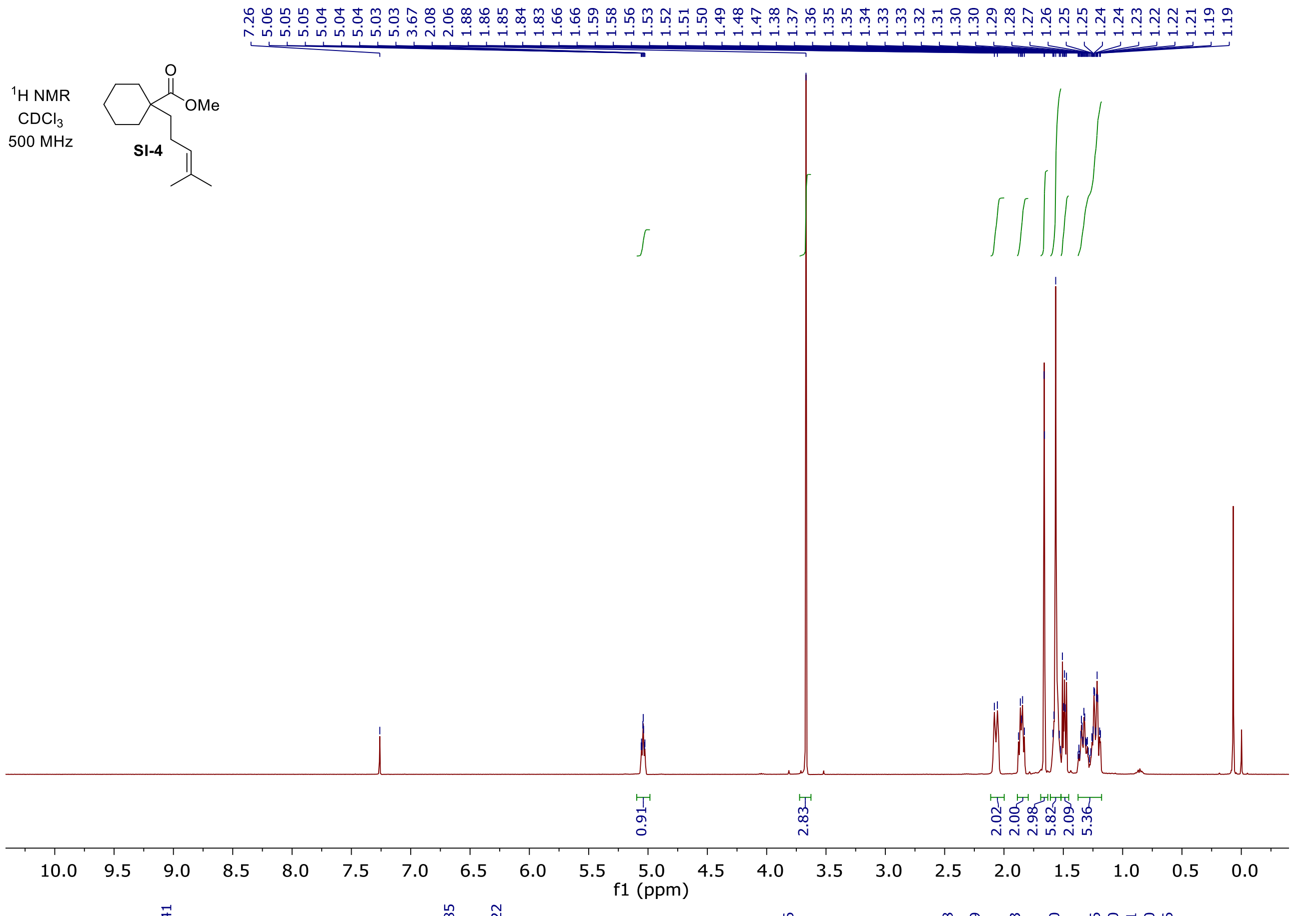

1H NMR
CDCl3
500 MHz
O
OMe
SI-4
7.26 5.06 5.05 5.05 5.04 5.04 5.03 5.03 3.67 2.08 2.06 1.88 1.86 1.85 1.84 1.83 1.66 1.66 1.59 1.58 1.56 1.53 1.52 1.51 1.50 1.49 1.48 1.47 1.38 1.37 1.36 1.35 1.35 1.34 1.33 1.33 1.32 1.31 1.30 1.30 1.29 1.28 1.27 1.26 1.25 1.25 1.24 1.24 1.23 1.22 1.22 1.21 1.19 1.19
0.91
2.83
2.02 2.00 2.98 5.82 2.09 5.36
10.0 9.5 9.0 8.5 8.0 7.5 7.0 6.5 6.0 5.5 5.0 4.5 4.0 3.5 3.0 2.5 2.0 1.5 1.0 0.5 0.0
f1 (ppm)

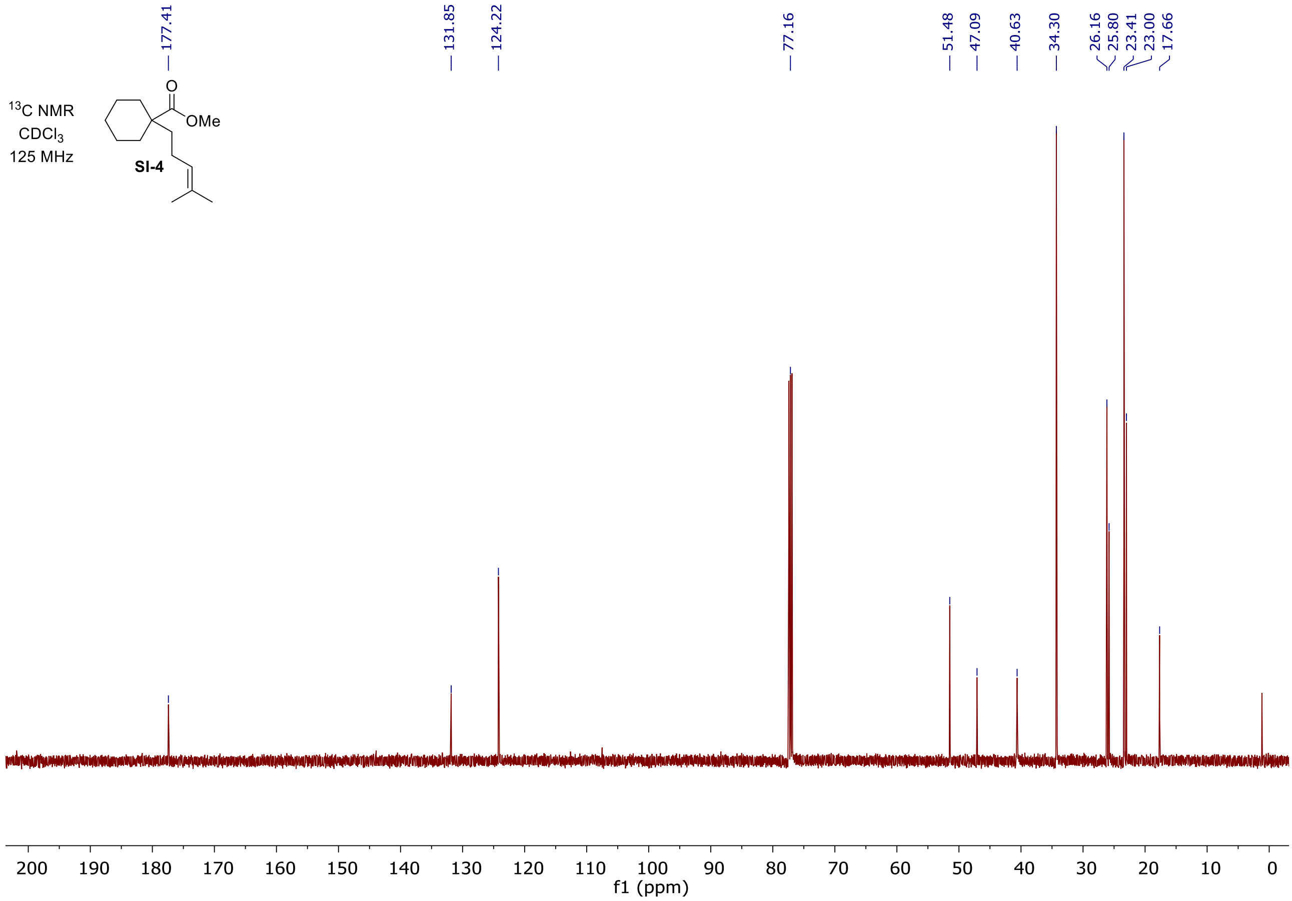

13C NMR
CDCl3
125 MHz
O
OMe
SI-4
177.41
131.85
124.22
77.16
51.48
47.09
40.63
34.30
26.16 25.80 23.41 23.00 17.66
200 190 180 170 160 150 140 130 120 110 100 90 80 70 60 50 40 30 20 10 0
f1 (ppm)

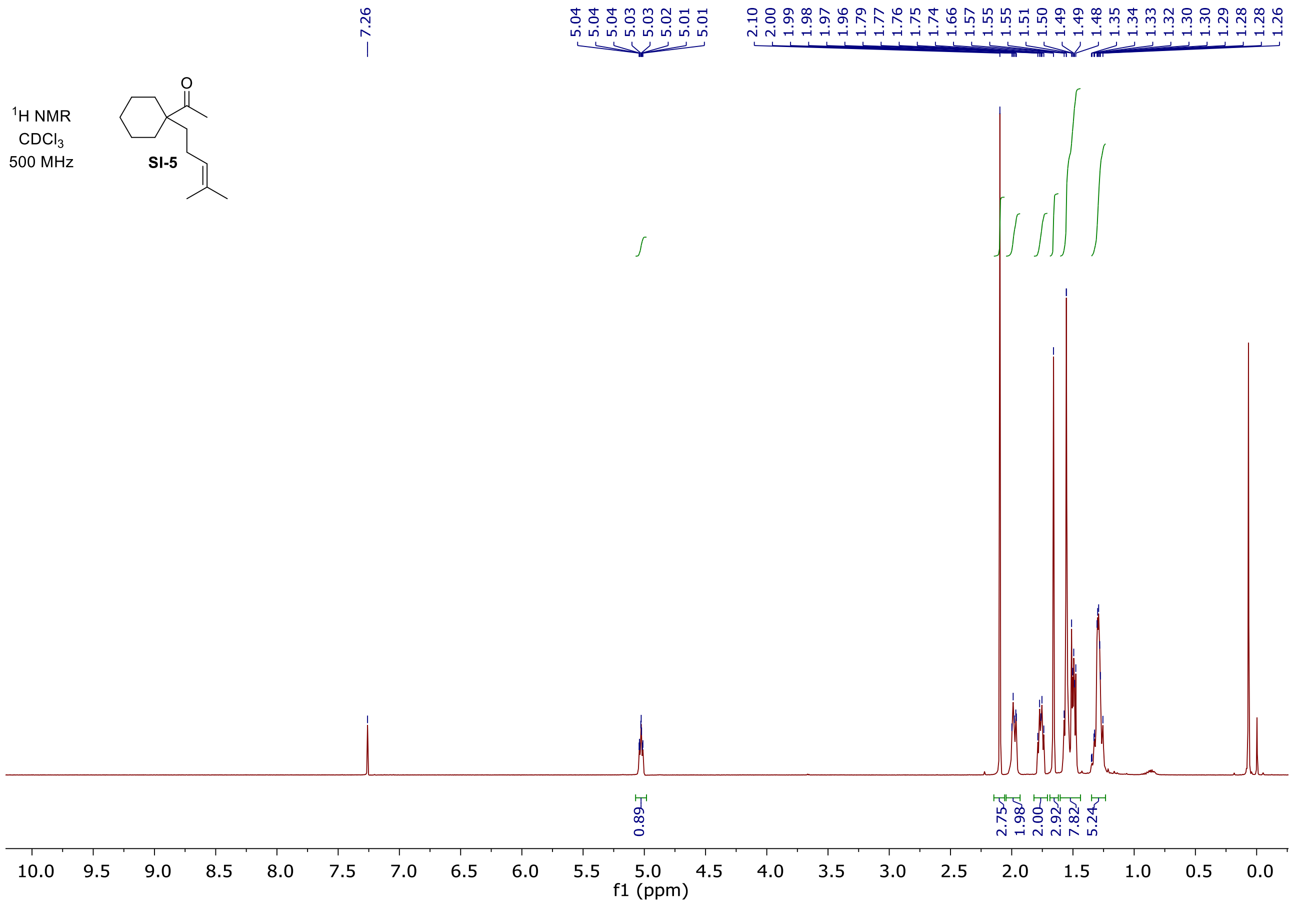

1H NMR
CDCl3
500 MHz
SI-5
O
7.26
5.04 5.04 5.04 5.03 5.03 5.02 5.01 5.01
2.10 2.00 1.99 1.98 1.97 1.96 1.79 1.77 1.76 1.75 1.74 1.66 1.57 1.55 1.55 1.51 1.50 1.49 1.49 1.48 1.35 1.34 1.33 1.32 1.30 1.29 1.28 1.26
0.89
2.75 1.98 2.00 2.92 7.82 5.24
10.0 9.5 9.0 8.5 8.0 7.5 7.0 6.5 6.0 5.5 5.0 4.5 4.0 3.5 3.0 2.5 2.0 1.5 1.0 0.5 0.0
f1 (ppm)

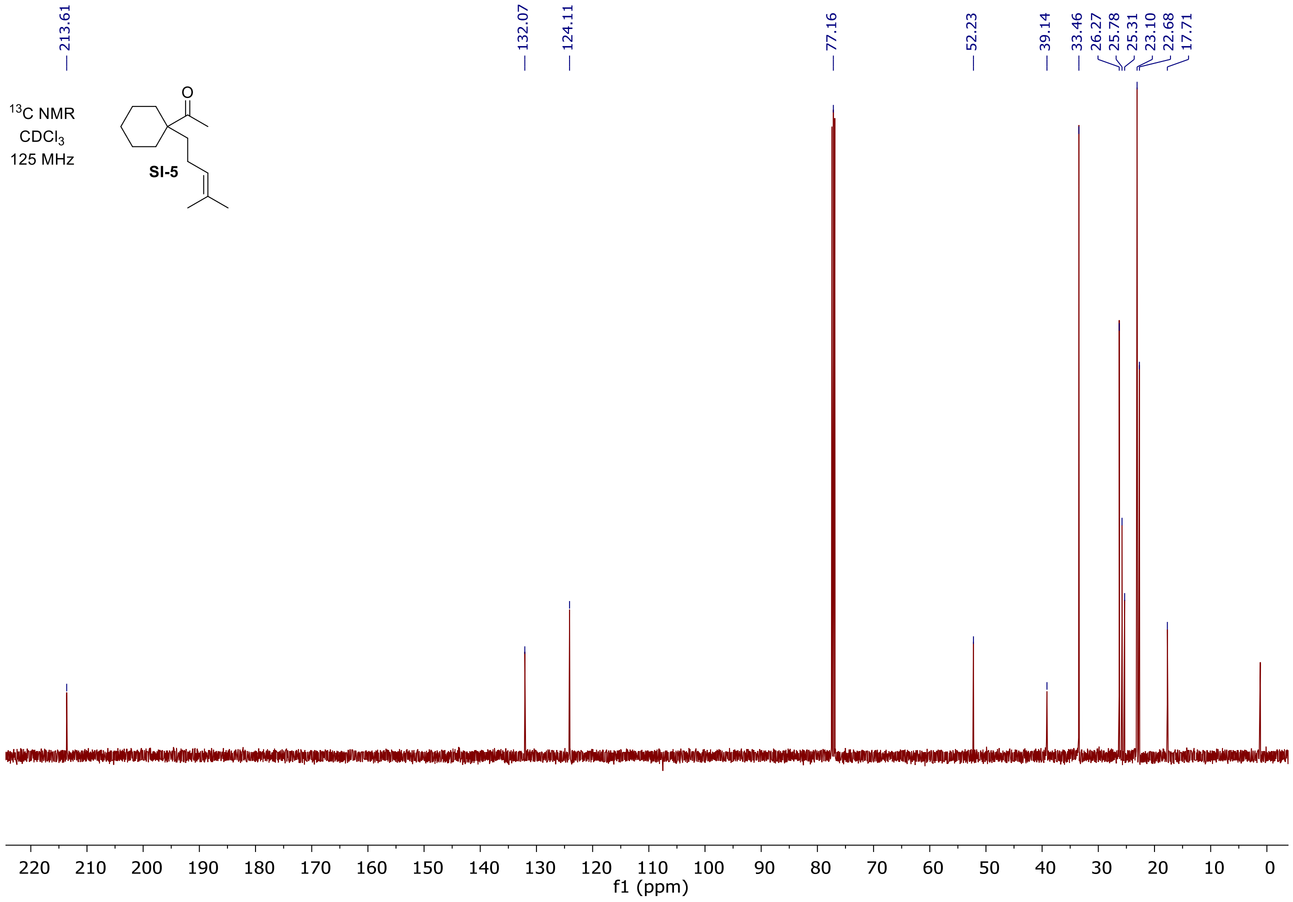

13C NMR
CDCl3
125 MHz
SI-5
O
213.61
132.07
124.11
77.16
52.23
39.14
33.46
26.27 25.78 25.31 23.10 22.68 17.71
220 210 200 190 180 170 160 150 140 130 120 110 100 90 80 70 60 50 40 30 20 10 0
f1 (ppm)

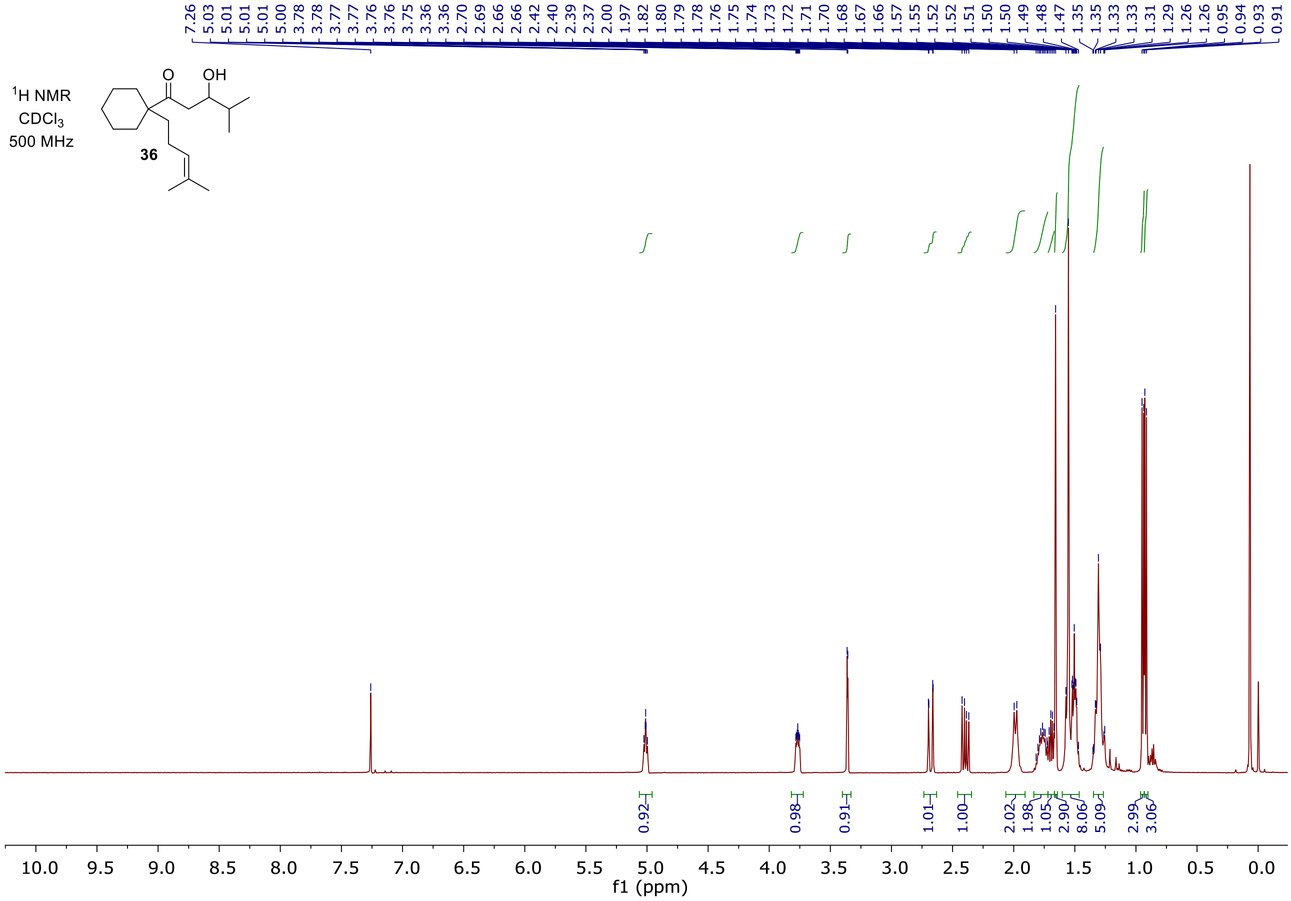
7.26 5.03 5.01 5.01 5.00 3.78 3.77 3.77 3.76 3.75 3.36 3.36 2.70 2.69 2.66 2.66 2.42 2.40 2.39 2.37 2.00 1.97 1.82 1.80 1.79 1.78 1.76 1.75 1.74 1.73 1.72 1.71 1.70 1.68 1.67 1.66 1.57 1.55 1.52 1.52 1.51 1.50 1.50 1.49 1.48 1.47 1.35 1.35 1.33 1.33 1.31 1.29 1.26 0.95 0.94 0.93 0.91
1H NMR
CDCl3
500 MHz
O
OH
36
0.92 0.98 0.91 1.01 1.00 2.02 1.98 1.05 2.90 8.06 5.09 2.99 3.06
f1 (ppm)

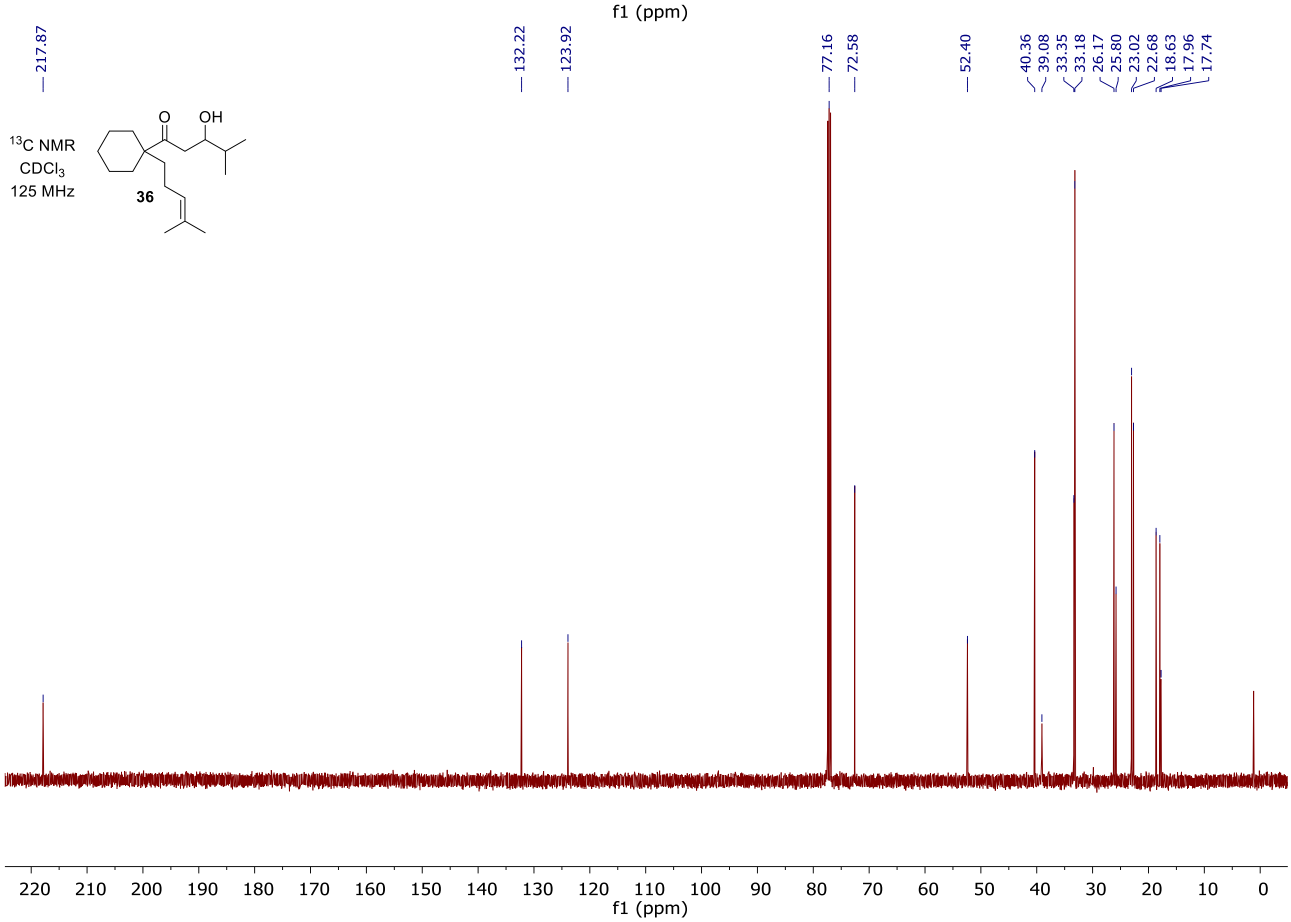
217.87 132.22 123.92 77.16 72.58 52.40 40.36 39.08 33.35 33.18 26.17 25.80 23.02 22.68 18.63 17.96 17.74
13C NMR
CDCl3
125 MHz
O
OH
36
f1 (ppm)

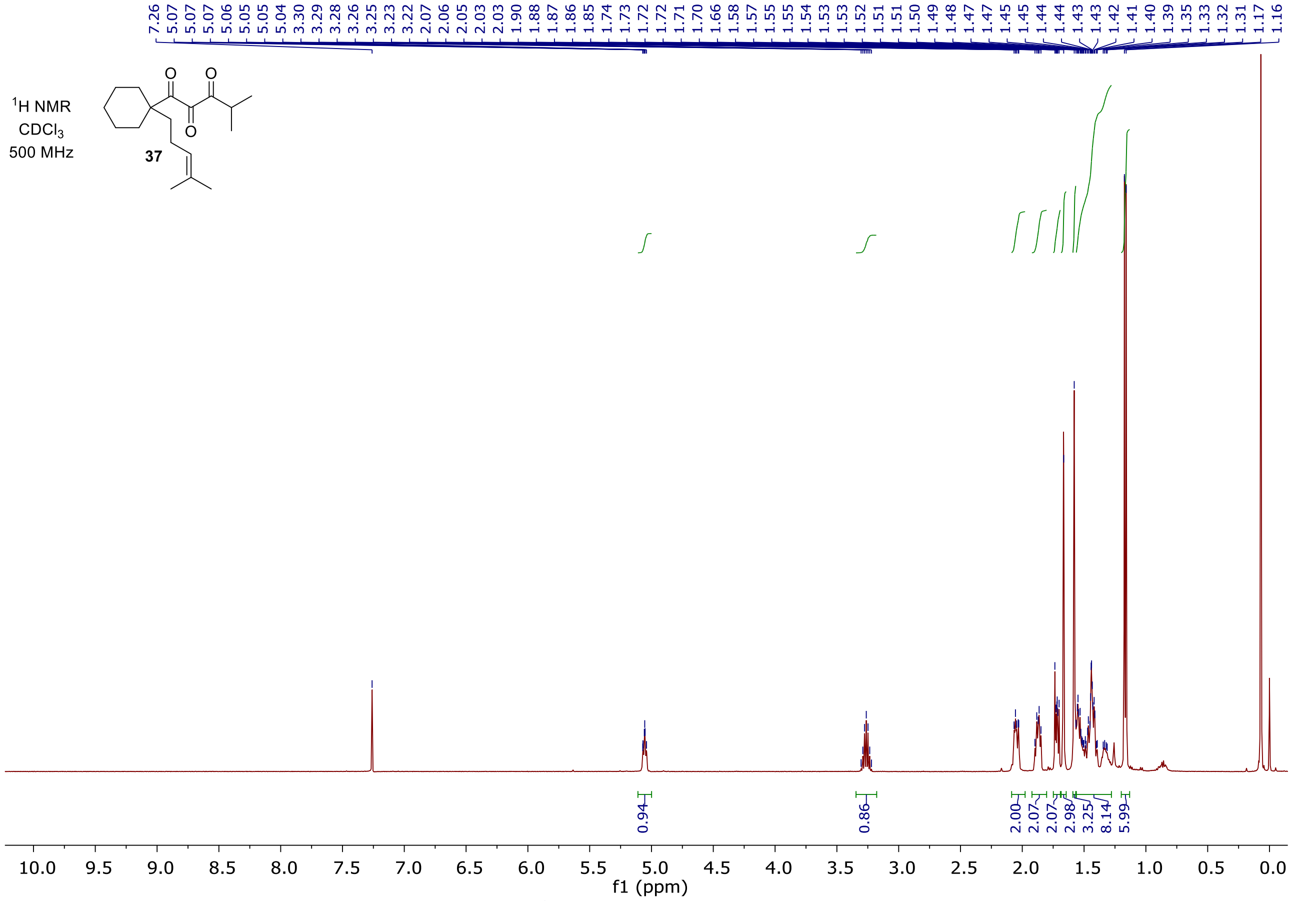
7.26 5.07 5.07 5.06 5.05 5.05 5.04 3.30 3.29 3.28 3.26 3.25 3.23 3.22 2.07 2.06 2.05 2.03 2.03 1.90 1.88 1.87 1.86 1.85 1.74 1.73 1.72 1.72 1.71 1.70 1.66 1.58 1.57 1.55 1.55 1.54 1.53 1.53 1.52 1.51 1.51 1.50 1.49 1.48 1.47 1.47 1.45 1.45 1.44 1.44 1.43 1.43 1.42 1.41 1.40 1.39 1.35 1.33 1.32 1.31 1.17 1.16
1H NMR
CDCl3
500 MHz
37
0.94
0.86
2.00
2.07
2.07
2.98
3.25
8.14
5.99
10.0 9.5 9.0 8.5 8.0 7.5 7.0 6.5 6.0 5.5 5.0 4.5 4.0 3.5 3.0 2.5 2.0 1.5 1.0 0.5 0.0
f1 (ppm)

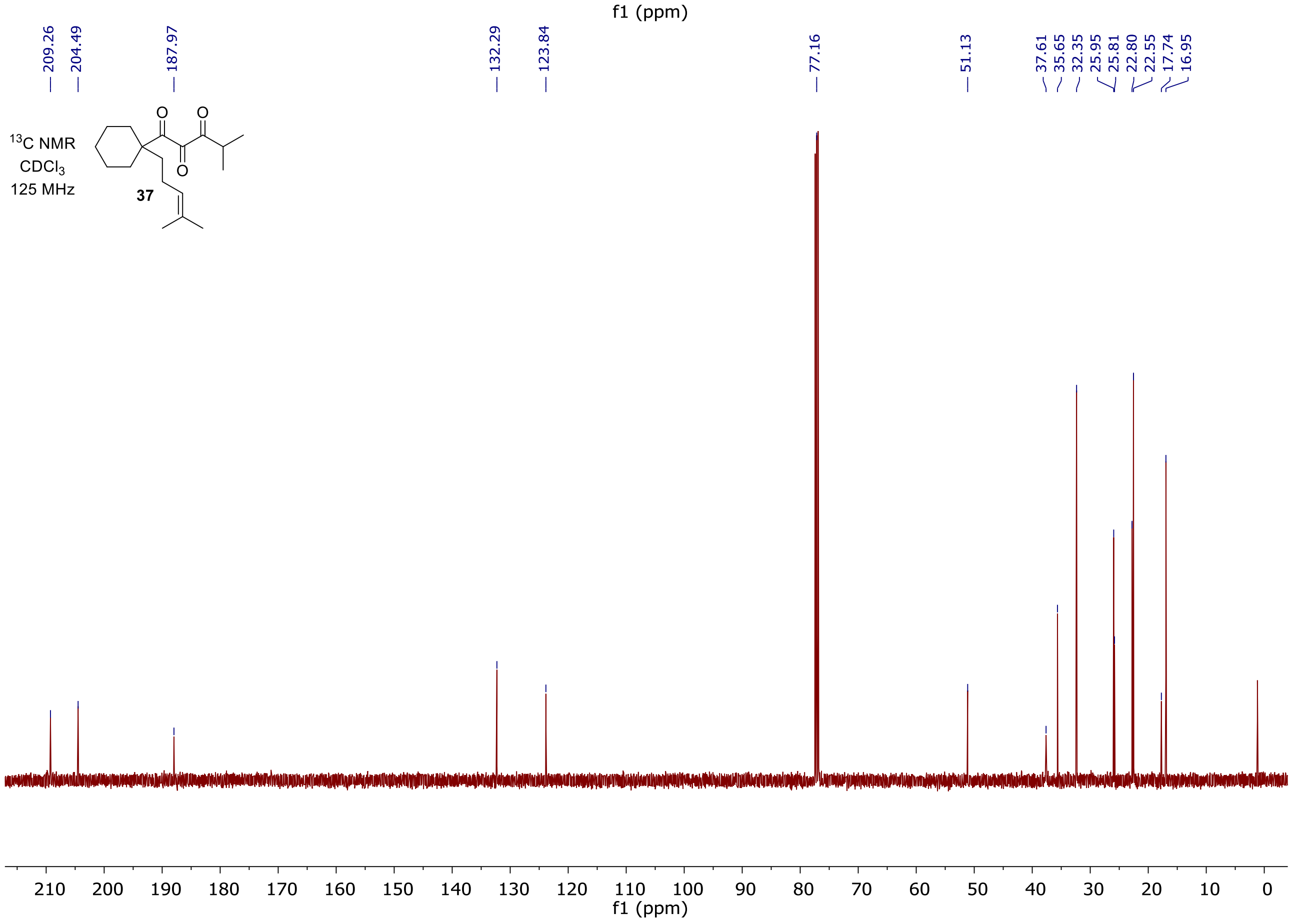
209.26
204.49
187.97
132.29
123.84
77.16
51.13
37.61 35.65 32.35 25.95 25.81 22.80 22.55 17.74 16.95
13C NMR
CDCl3
125 MHz
37
210 200 190 180 170 160 150 140 130 120 110 100 90 80 70 60 50 40 30 20 10 0
f1 (ppm)

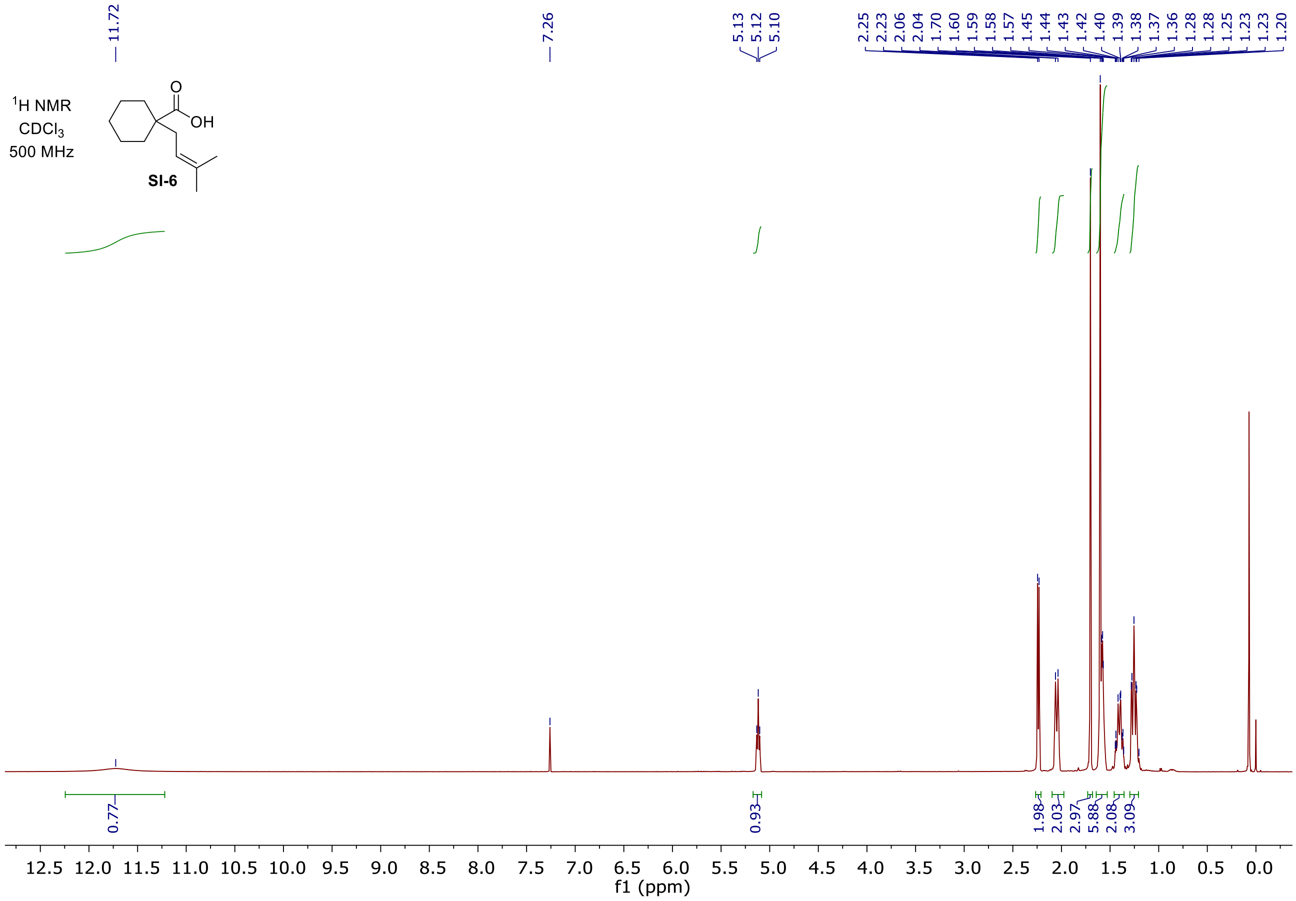

1H NMR
CDCl3
500 MHz
O
OH
SI-6
11.72
7.26
5.13
5.12
5.10
2.25
2.23
2.06
2.04
1.70
1.60
1.59
1.58
1.57
1.45
1.44
1.43
1.42
1.40
1.39
1.38
1.37
1.36
1.28
1.25
1.23
1.20
0.77
0.93
1.98
2.03
2.97
5.88
2.08
3.09
12.5 12.0 11.5 11.0 10.5 10.0 9.5 9.0 8.5 8.0 7.5 7.0 6.5 6.0 5.5 5.0 4.5 4.0 3.5 3.0 2.5 2.0 1.5 1.0 0.5 0.0
f1 (ppm)

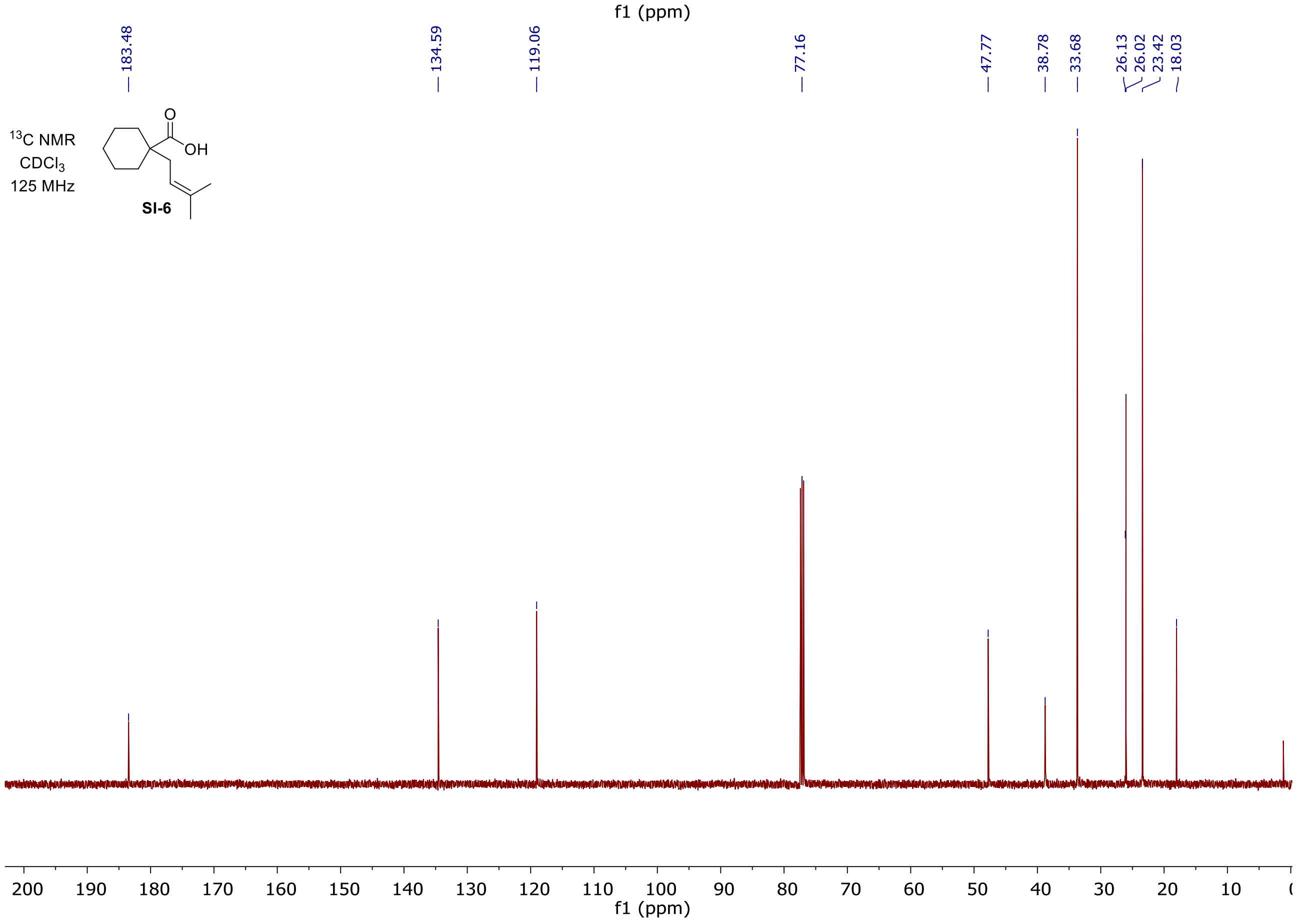

13C NMR
CDCl3
125 MHz
O
OH
SI-6
183.48
134.59
119.06
77.16
47.77
38.78
33.68
26.13
26.02
23.42
18.03
200 190 180 170 160 150 140 130 120 110 100 90 80 70 60 50 40 30 20 10
f1 (ppm)

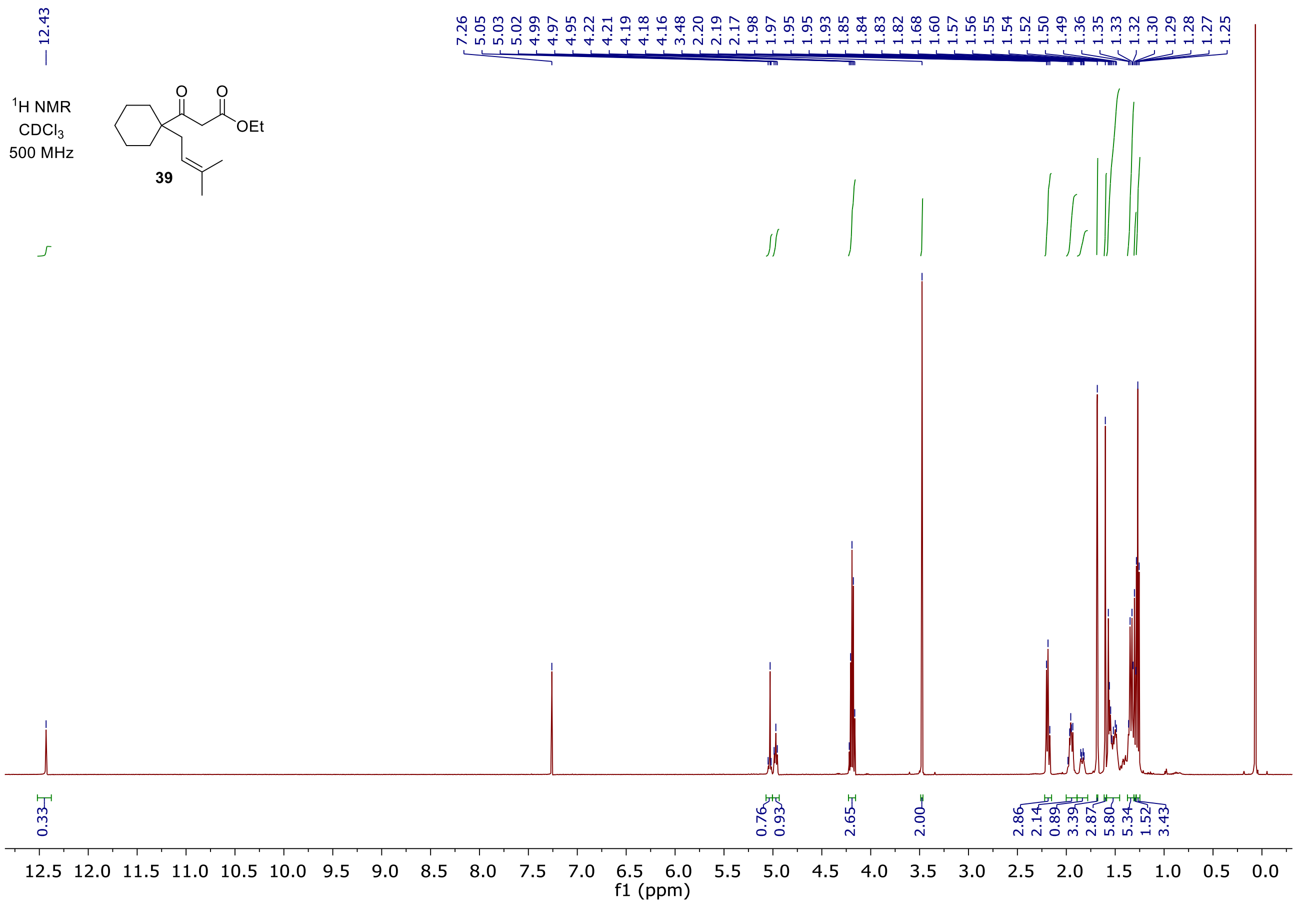

1H NMR
CDCl3
500 MHz
OEt
39
f1 (ppm)

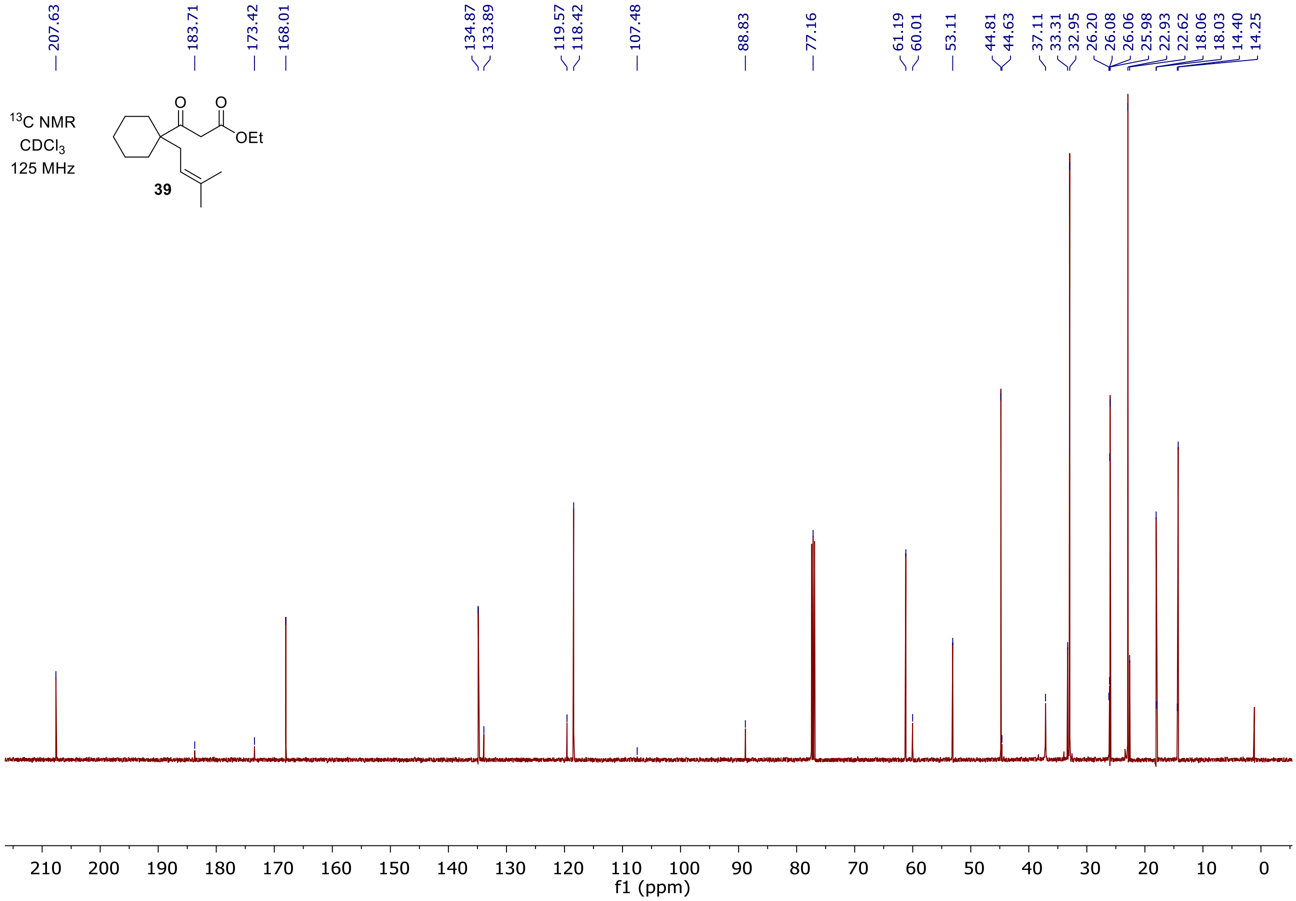

13C NMR
CDCl3
125 MHz
OEt
39
f1 (ppm)

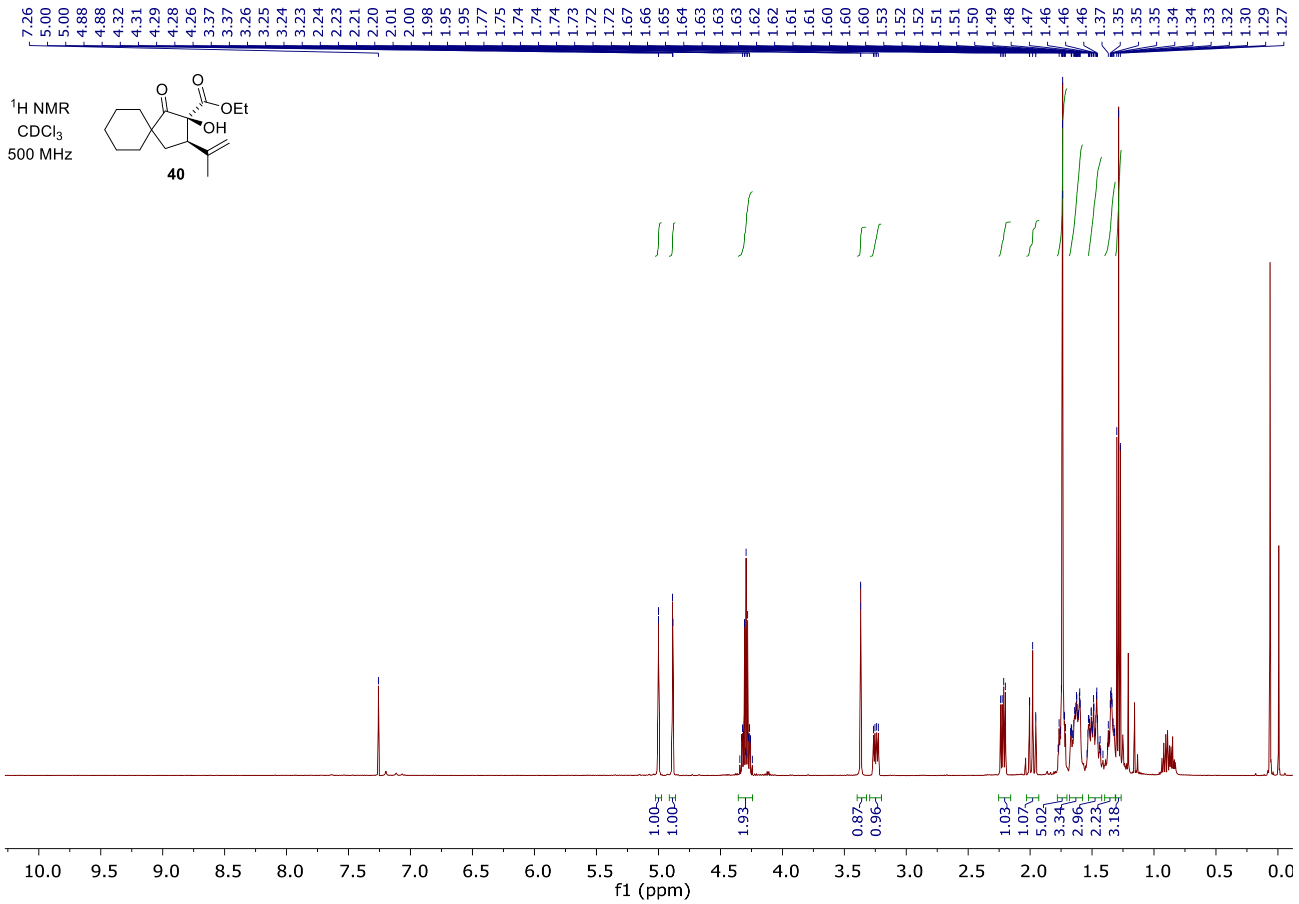
7.26 5.00 5.00 4.88 4.88 4.32 4.31 4.29 4.28 4.26 3.37 3.37 3.26 3.25 3.24 3.23 2.24 2.23 2.21 2.20 2.01 2.00 1.98 1.95 1.95 1.77 1.75 1.74 1.74 1.73 1.72 1.72 1.67 1.66 1.65 1.64 1.63 1.63 1.63 1.62 1.62 1.61 1.61 1.60 1.60 1.53 1.52 1.52 1.51 1.51 1.50 1.49 1.48 1.47 1.46 1.46 1.46 1.37 1.35 1.35 1.34 1.34 1.33 1.32 1.30 1.29 1.27
^{1}H NMR
$CDCl_3$
500 MHz
O
O
OEt
OH
40
1.00
1.00
1.93
0.87
0.96
1.03
1.07
5.02
3.34
2.96
2.23
3.18
10.0 9.5 9.0 8.5 8.0 7.5 7.0 6.5 6.0 5.5 5.0 4.5 4.0 3.5 3.0 2.5 2.0 1.5 1.0 0.5 0.0
f1 (ppm)

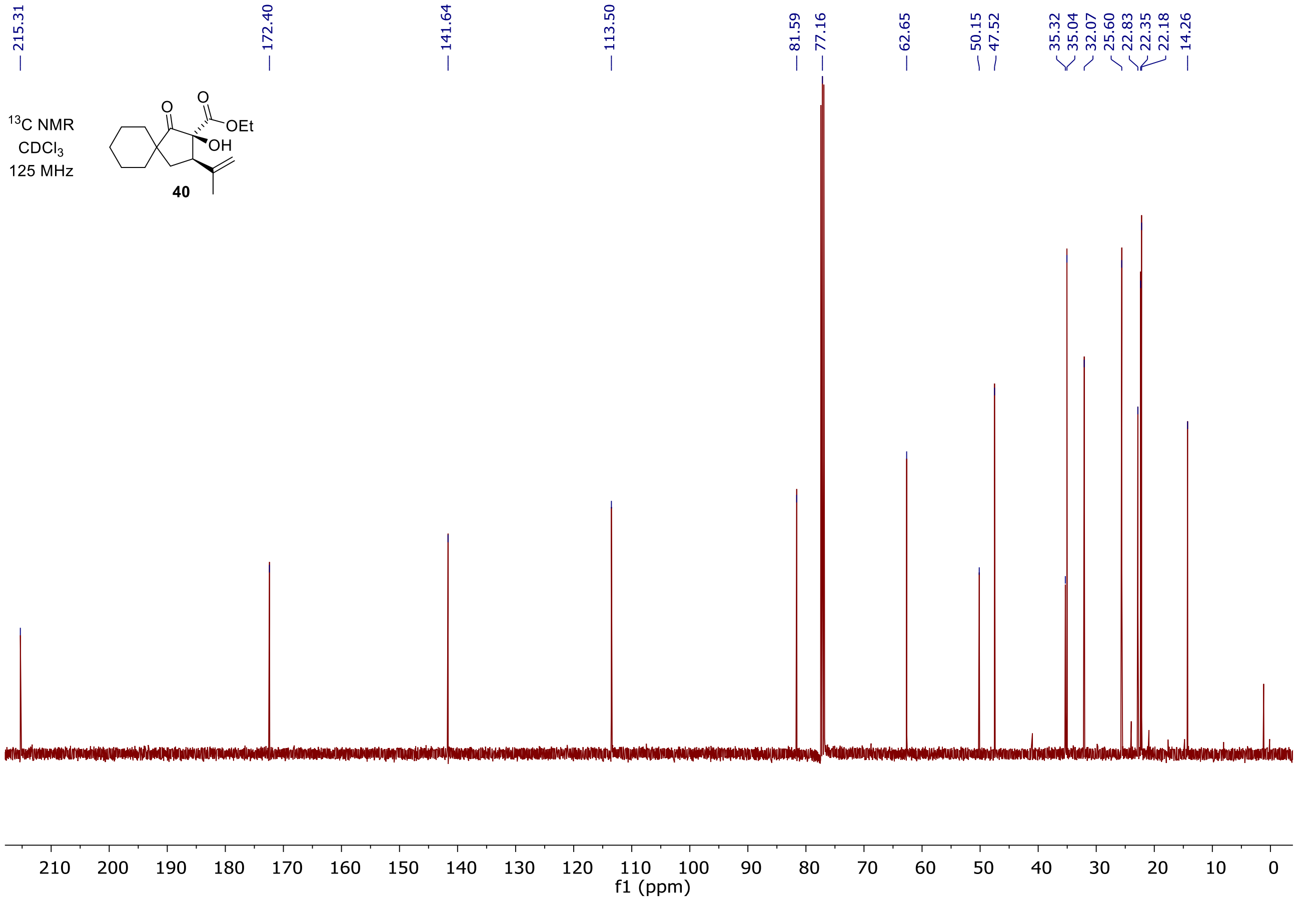
215.31 172.40 141.64 113.50 81.59 77.16 62.65 50.15 47.52 35.32 35.04 32.07 25.60 22.83 22.35 22.18 14.26
^{13}C NMR
$CDCl_3$
125 MHz
O
O
OEt
OH
40
210 200 190 180 170 160 150 140 130 120 110 100 90 80 70 60 50 40 30 20 10 0
f1 (ppm)

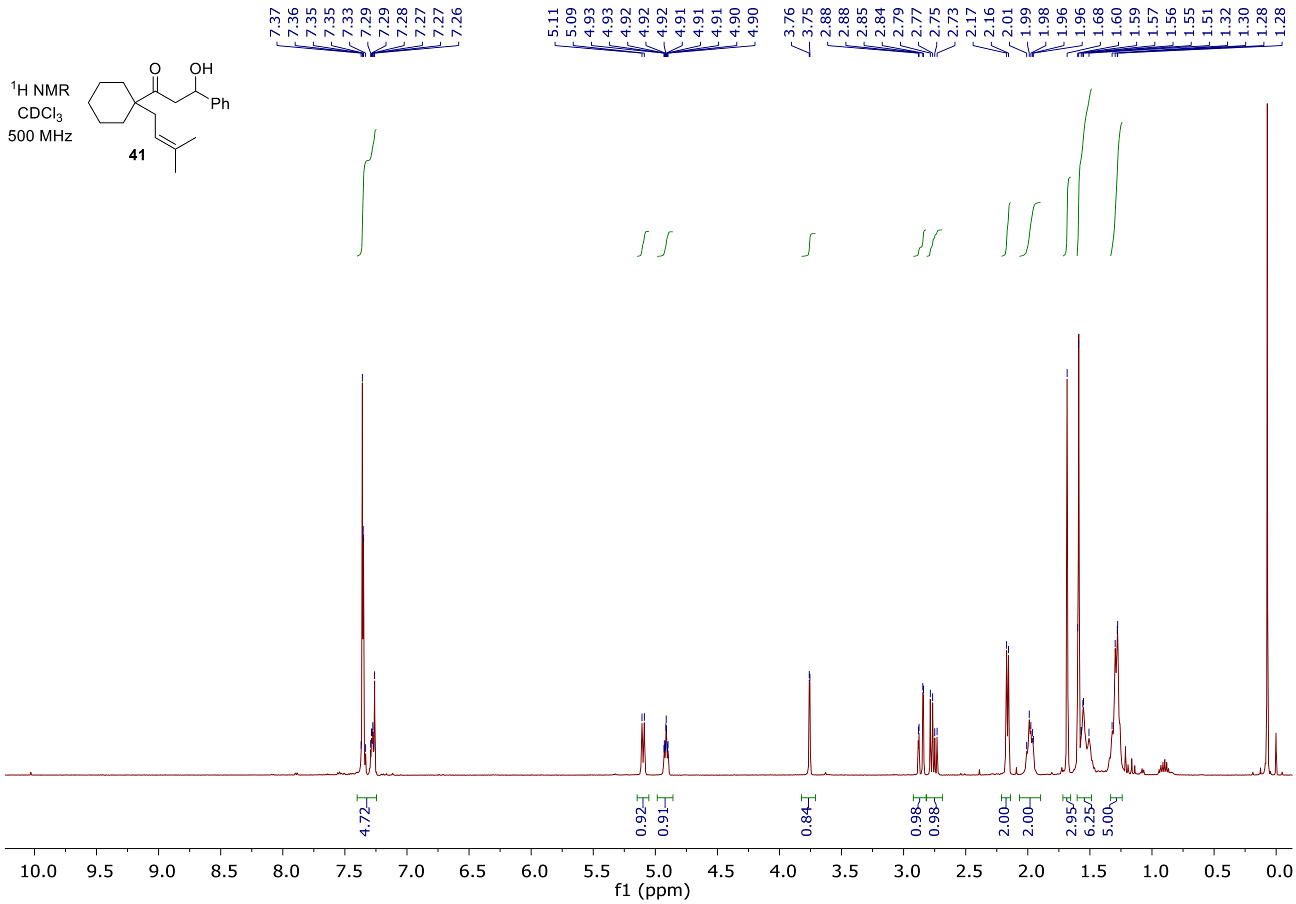

^{1}H NMR
CDCl$_3$
500 MHz
O
OH
Ph
41
7.37 7.36 7.35 7.35 7.33 7.29 7.29 7.28 7.27 7.27 7.26
5.11 5.09 4.93 4.93 4.92 4.92 4.92 4.91 4.91 4.91 4.90 4.90
3.76 3.75 2.88 2.88 2.85 2.84 2.79 2.77 2.75 2.73 2.17 2.16 2.01 1.99 1.98 1.96 1.96 1.68 1.60 1.59 1.57 1.56 1.55 1.51 1.32 1.30 1.28 1.28
4.72 0.92 0.91 0.84 0.98 0.98 2.00 2.00 2.95 6.25 5.00
10.0 9.5 9.0 8.5 8.0 7.5 7.0 6.5 6.0 5.5 5.0 4.5 4.0 3.5 3.0 2.5 2.0 1.5 1.0 0.5 0.0
f1 (ppm)

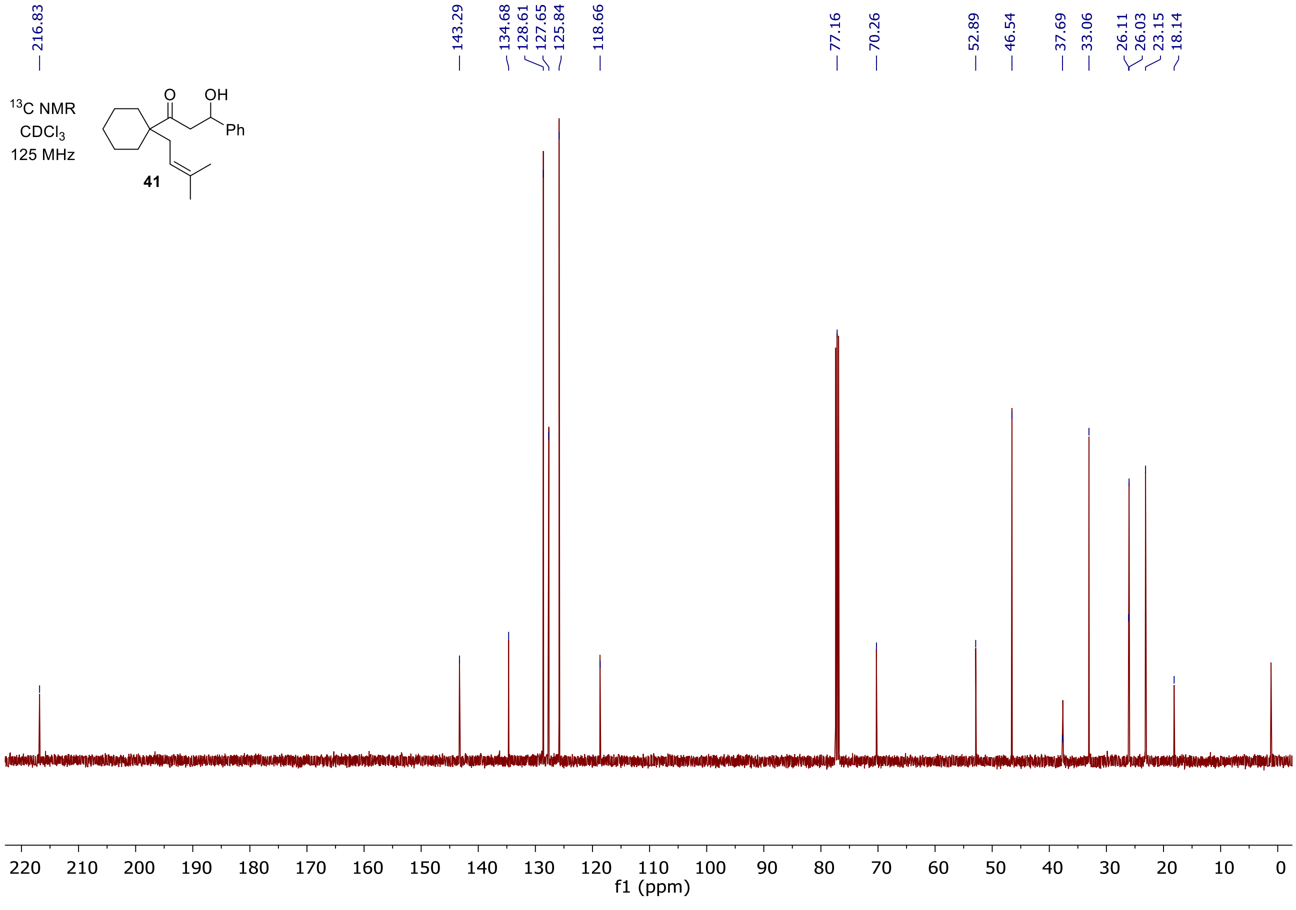

216.83
143.29 134.68 128.61 127.65 125.84 118.66
77.16 70.26
52.89 46.54 37.69 33.06 26.11 26.03 23.15 18.14
^{13}C NMR
CDCl$_3$
125 MHz
O
OH
Ph
41
220 210 200 190 180 170 160 150 140 130 120 110 100 90 80 70 60 50 40 30 20 10 0
f1 (ppm)

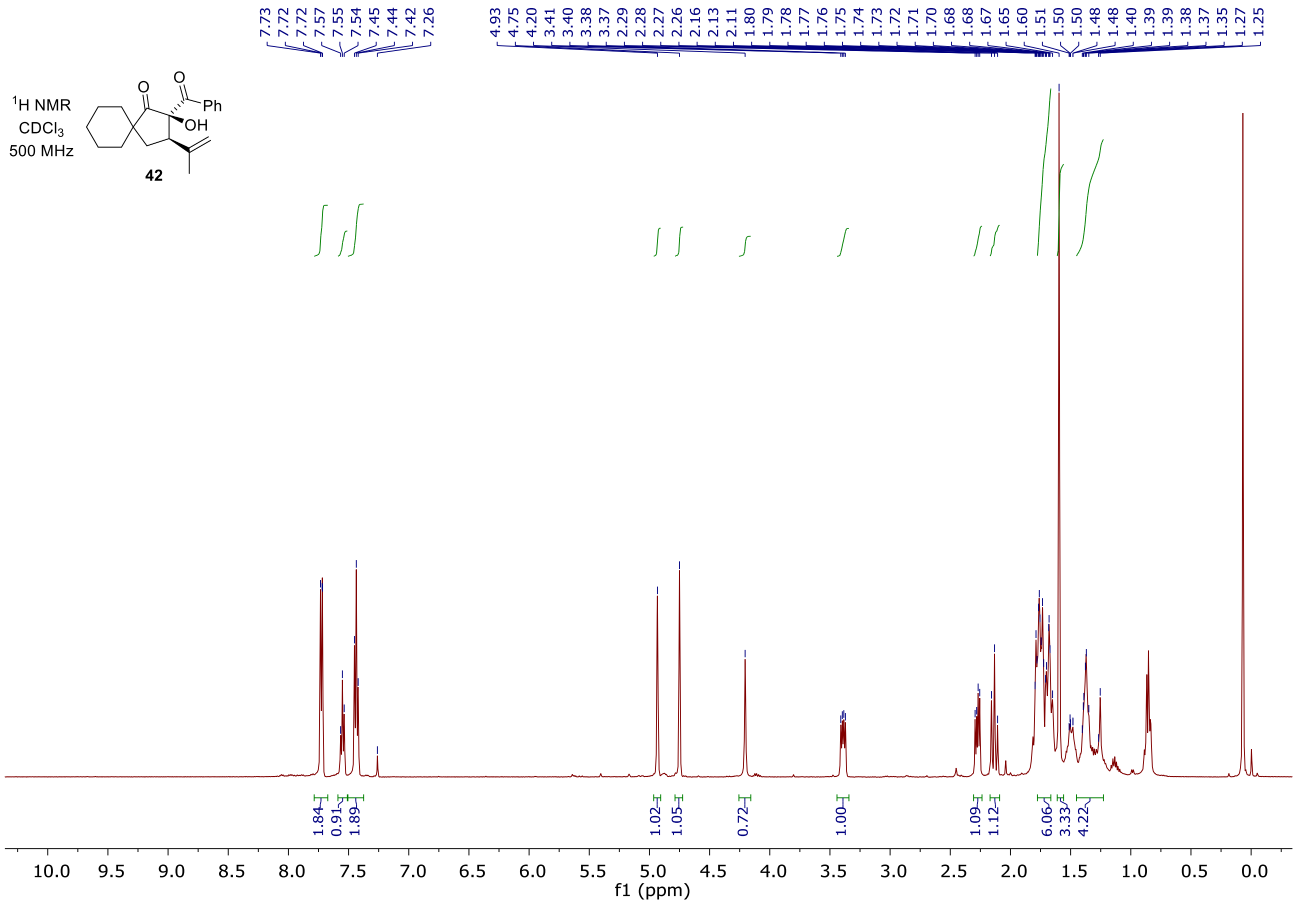
1H NMR
CDCl3
500 MHz
42
O
Ph
OH
f1 (ppm)

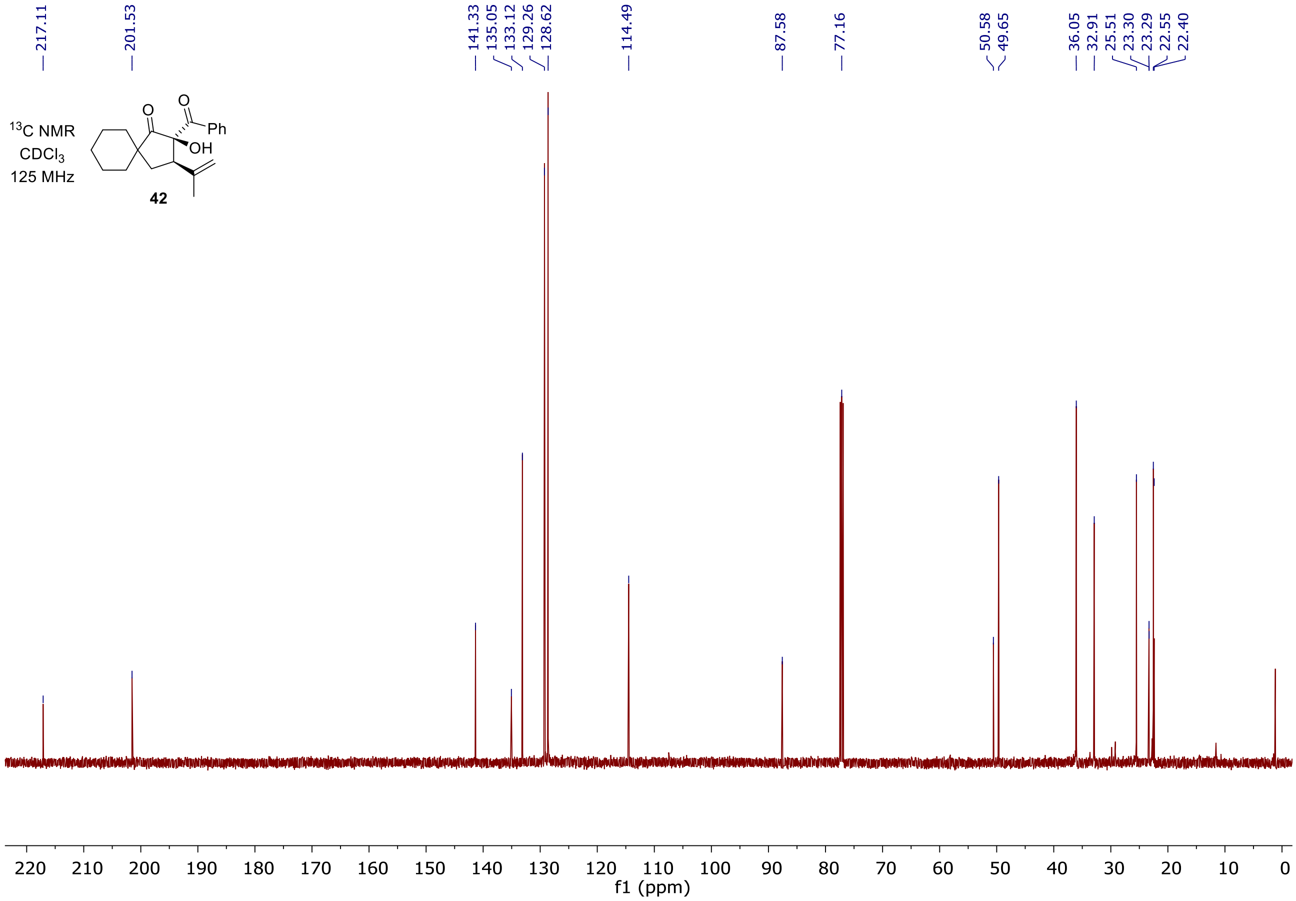
13C NMR
CDCl3
125 MHz
42
O
Ph
OH
f1 (ppm)

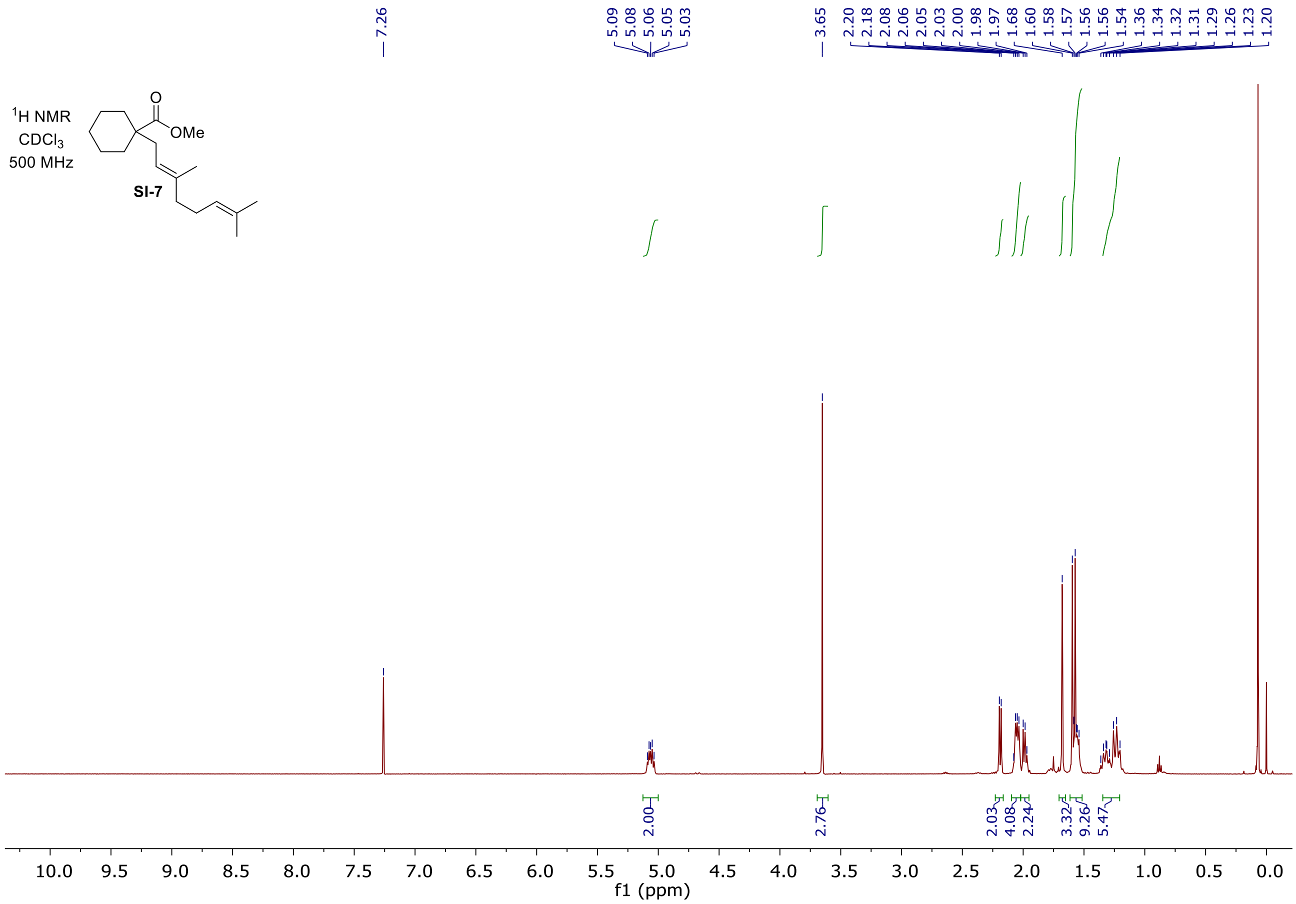
7.26
5.09 5.08 5.06 5.05 5.03
3.65
2.20 2.18 2.08 2.06 2.05 2.03 2.00 1.98 1.97 1.68 1.60 1.58 1.57 1.56 1.56 1.54 1.36 1.34 1.32 1.31 1.29 1.26 1.23 1.20
1H NMR
CDCl3
500 MHz
O
OMe
SI-7
2.00
2.76
2.03 4.08 2.24
3.32 9.26 5.47
10.0 9.5 9.0 8.5 8.0 7.5 7.0 6.5 6.0 5.5 5.0 4.5 4.0 3.5 3.0 2.5 2.0 1.5 1.0 0.5 0.0
f1 (ppm)

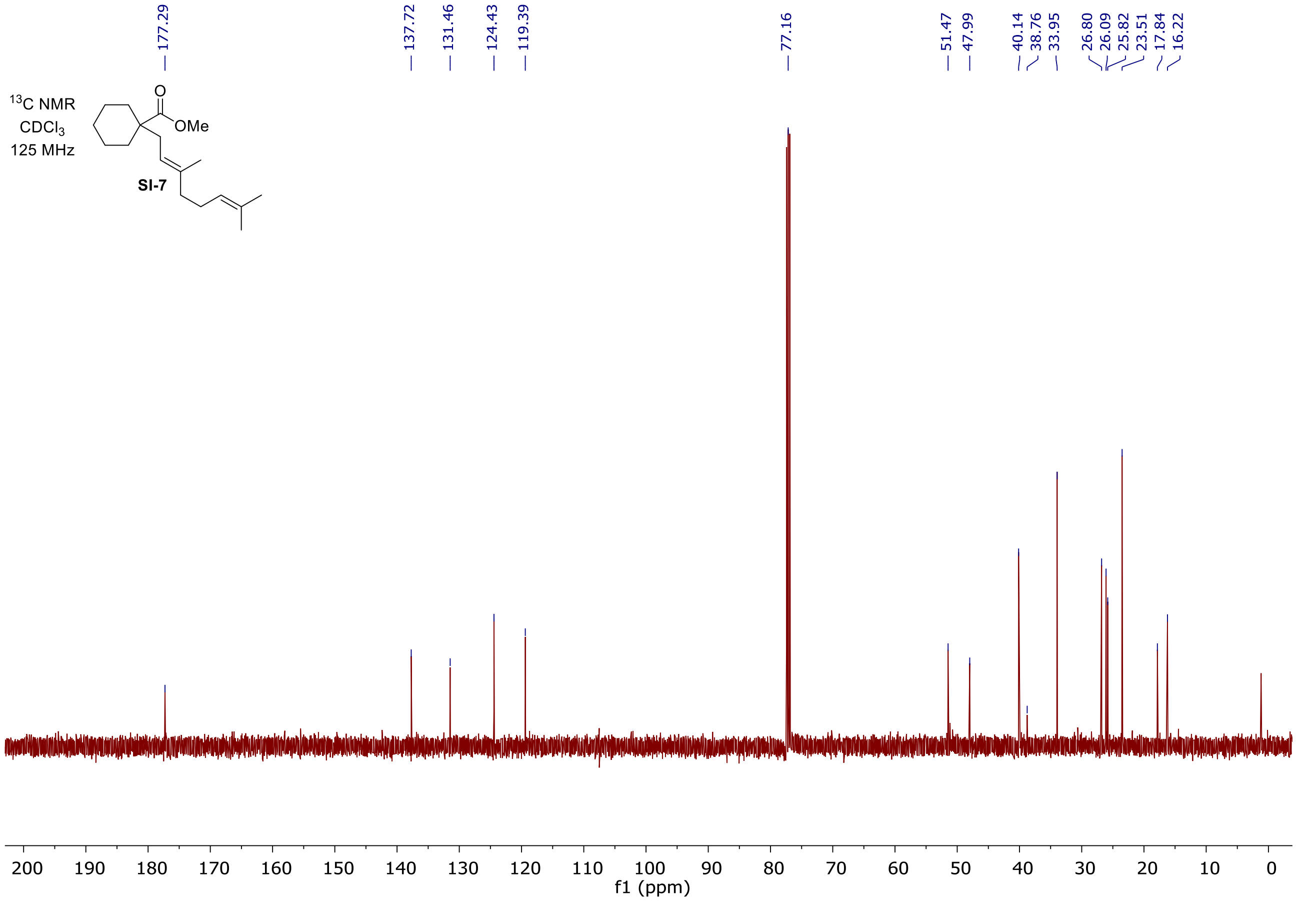
177.29
137.72 131.46 124.43 119.39
77.16
51.47 47.99
40.14 38.76 33.95
26.80 26.09 25.82 23.51 17.84 16.22
13C NMR
CDCl3
125 MHz
O
OMe
SI-7
200 190 180 170 160 150 140 130 120 110 100 90 80 70 60 50 40 30 20 10 0
f1 (ppm)

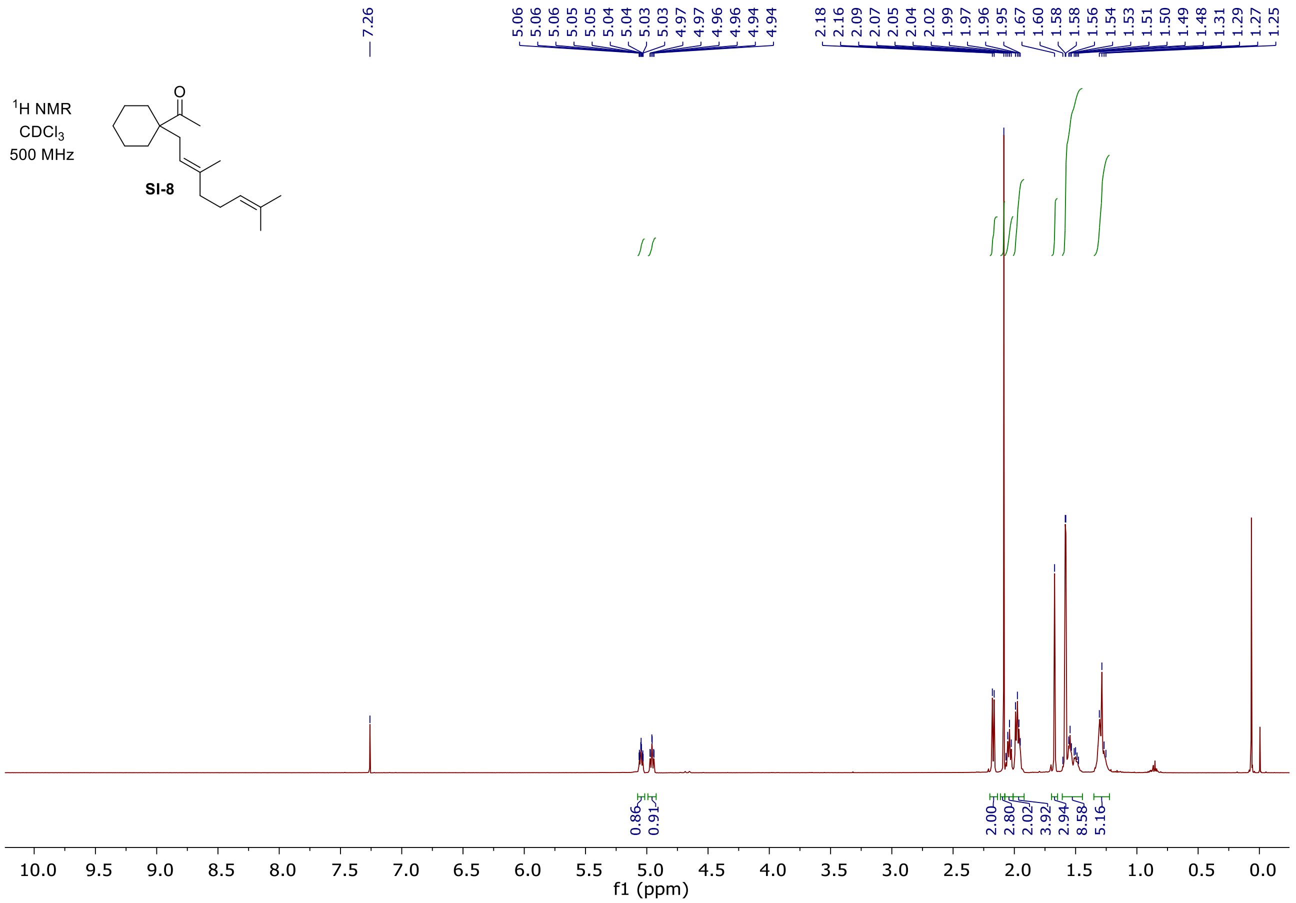

^{1}H NMR
$CDCl_3$
500 MHz
SI-8
f1 (ppm)

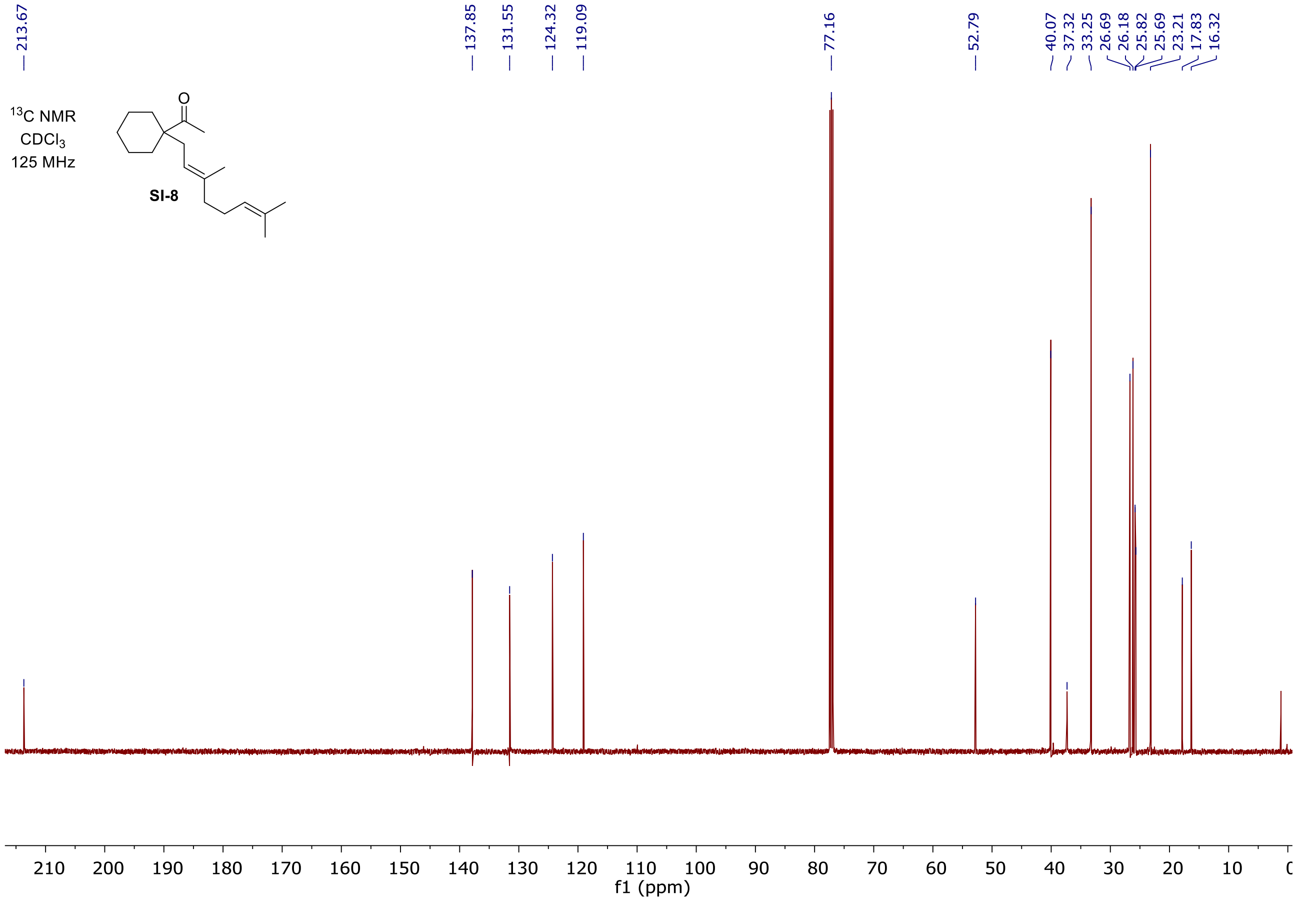

^{13}C NMR
$CDCl_3$
125 MHz
SI-8
f1 (ppm)

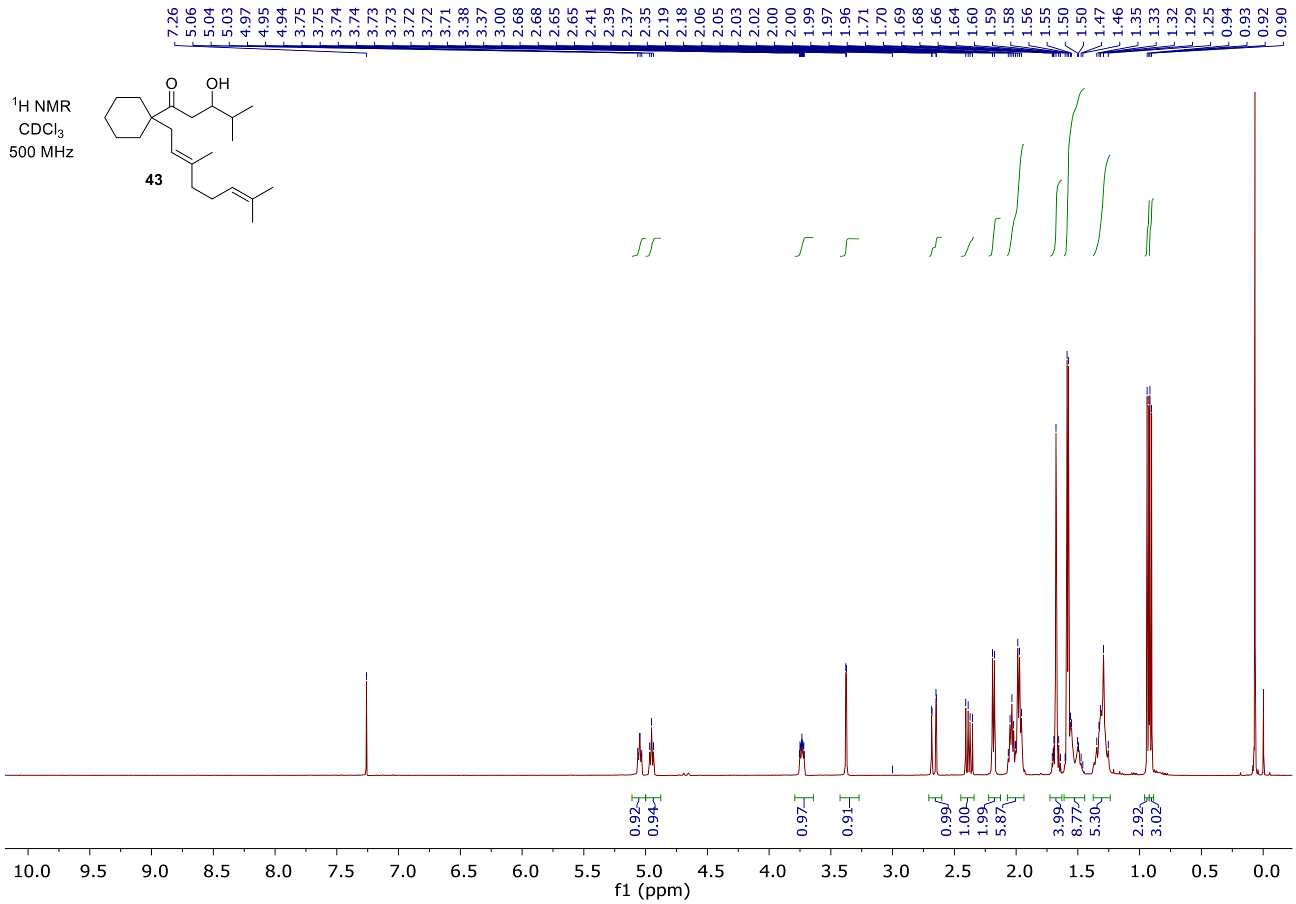

7.26 5.06 5.04 5.03 4.97 4.95 4.94 3.75 3.75 3.74 3.74 3.73 3.73 3.72 3.72 3.71 3.38 3.37 3.00 2.68 2.68 2.65 2.65 2.41 2.39 2.37 2.35 2.19 2.18 2.06 2.05 2.03 2.02 2.00 2.00 1.99 1.97 1.96 1.71 1.70 1.69 1.68 1.66 1.64 1.60 1.59 1.58 1.56 1.55 1.50 1.50 1.47 1.46 1.35 1.33 1.32 1.29 1.25 0.94 0.93 0.92 0.90
1H NMR
CDCl3
500 MHz
O
OH
43
0.92 0.94 0.97 0.91 0.99 1.00 1.99 5.87 3.99 8.77 5.30 2.92 3.02
10.0 9.5 9.0 8.5 8.0 7.5 7.0 6.5 6.0 5.5 5.0 4.5 4.0 3.5 3.0 2.5 2.0 1.5 1.0 0.5 0.0
f1 (ppm)

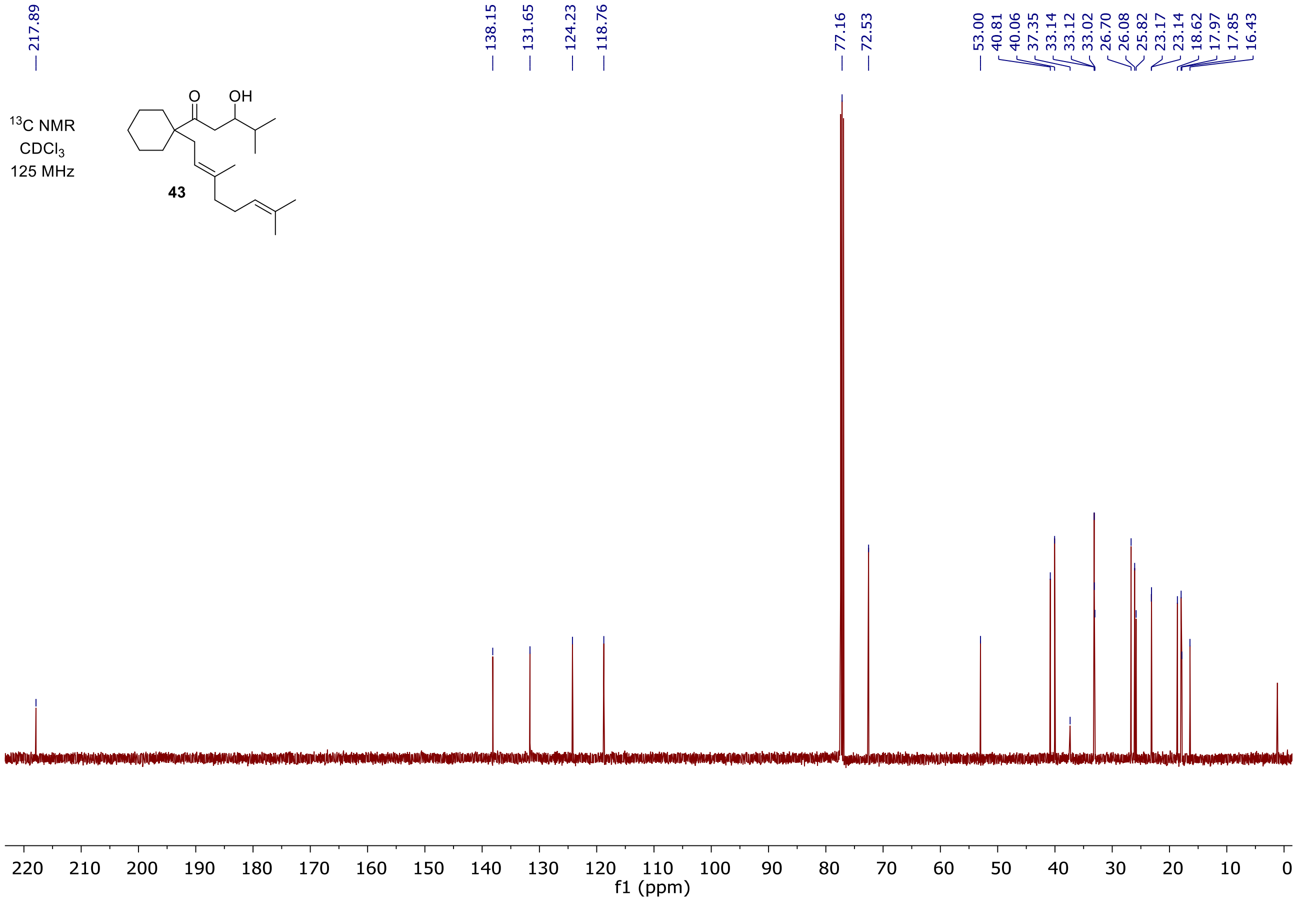

217.89 138.15 131.65 124.23 118.76 77.16 72.53 53.00 40.81 40.06 37.35 33.14 33.12 33.02 26.70 26.08 25.82 23.17 23.14 18.62 17.97 17.85 16.43
13C NMR
CDCl3
125 MHz
O
OH
43
220 210 200 190 180 170 160 150 140 130 120 110 100 90 80 70 60 50 40 30 20 10 0
f1 (ppm)

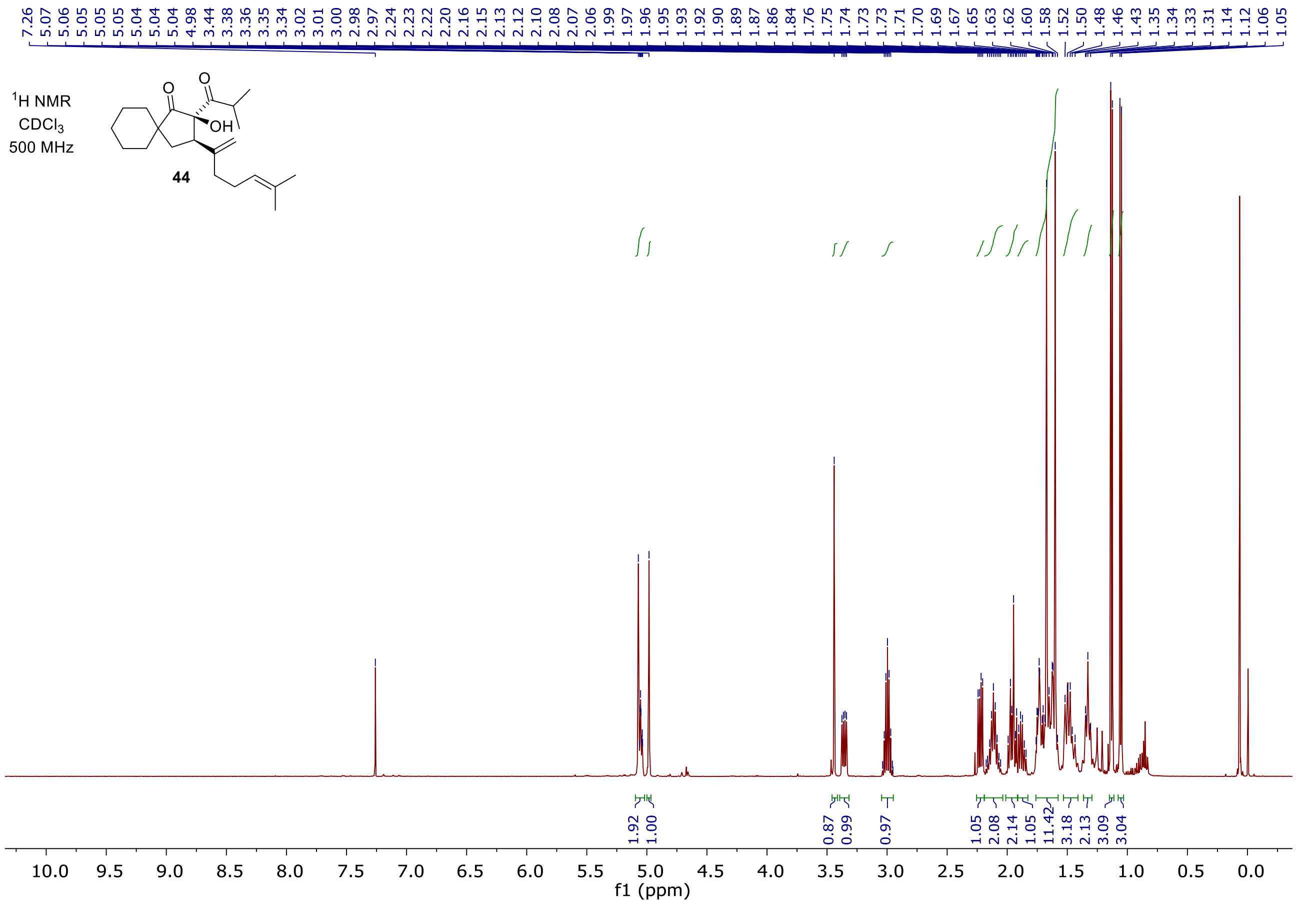
7.26 5.07 5.06 5.05 5.05 5.05 5.04 5.04 5.04 4.98 3.44 3.38 3.36 3.35 3.34 3.02 3.01 3.00 2.98 2.97 2.24 2.23 2.22 2.20 2.16 2.15 2.13 2.12 2.10 2.08 2.07 2.06 1.99 1.97 1.96 1.95 1.93 1.92 1.90 1.89 1.87 1.86 1.84 1.76 1.75 1.74 1.73 1.73 1.71 1.70 1.69 1.67 1.65 1.63 1.62 1.60 1.58 1.52 1.50 1.48 1.46 1.43 1.35 1.34 1.33 1.31 1.14 1.12 1.06 1.05
1H NMR
CDCl3
500 MHz
O
O
OH
44
1.92 1.00 0.87 0.99 0.97 1.05 2.08 2.14 1.05 11.42 3.18 2.13 3.09 3.04
10.0 9.5 9.0 8.5 8.0 7.5 7.0 6.5 6.0 5.5 5.0 4.5 4.0 3.5 3.0 2.5 2.0 1.5 1.0 0.5 0.0
f1 (ppm)

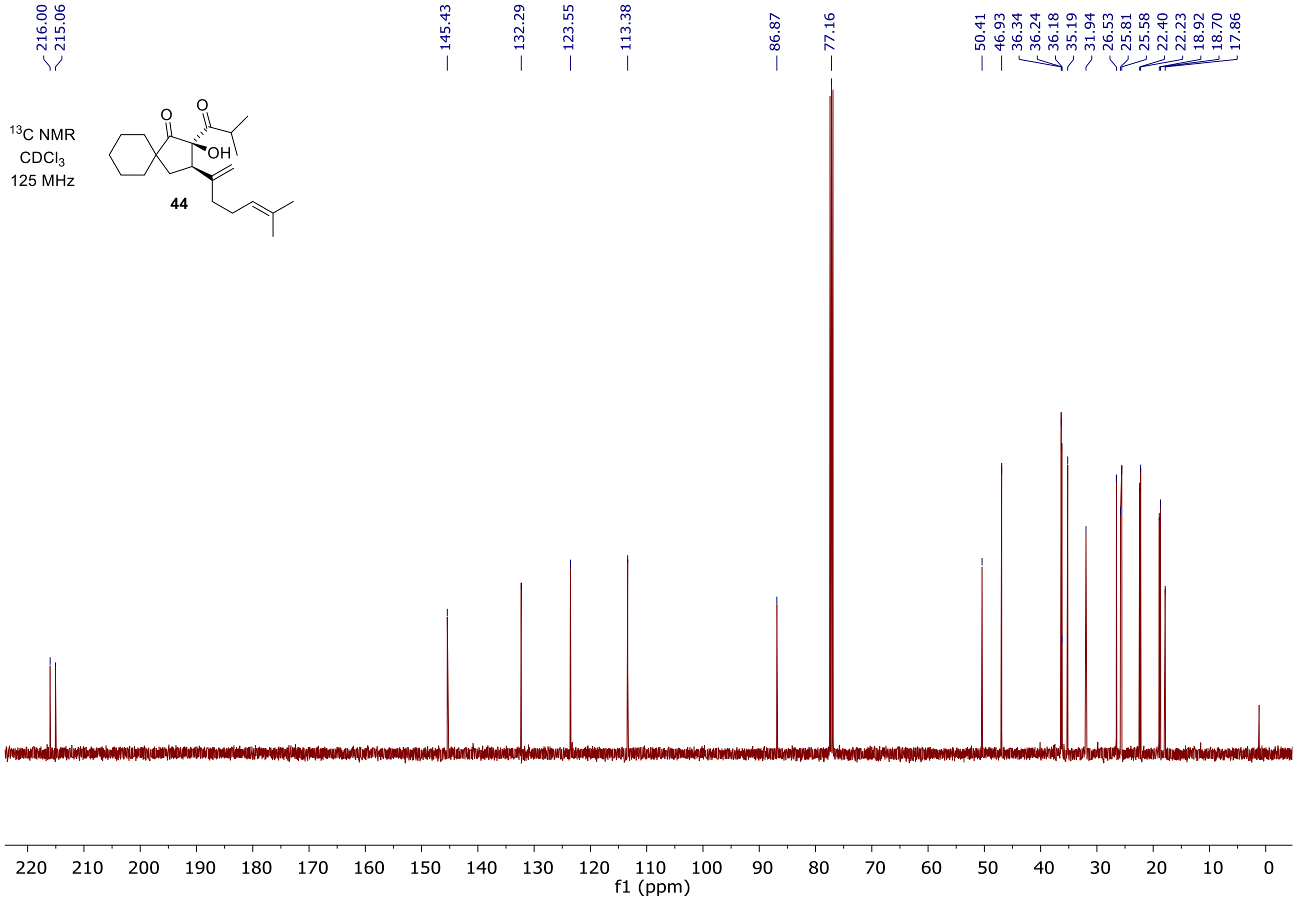
216.00 215.06 145.43 132.29 123.55 113.38 86.87 77.16 50.41 46.93 36.34 36.24 36.18 35.19 31.94 26.53 25.81 25.58 22.40 22.23 18.92 18.70 17.86
13C NMR
CDCl3
125 MHz
O
O
OH
44
220 210 200 190 180 170 160 150 140 130 120 110 100 90 80 70 60 50 40 30 20 10 0
f1 (ppm)

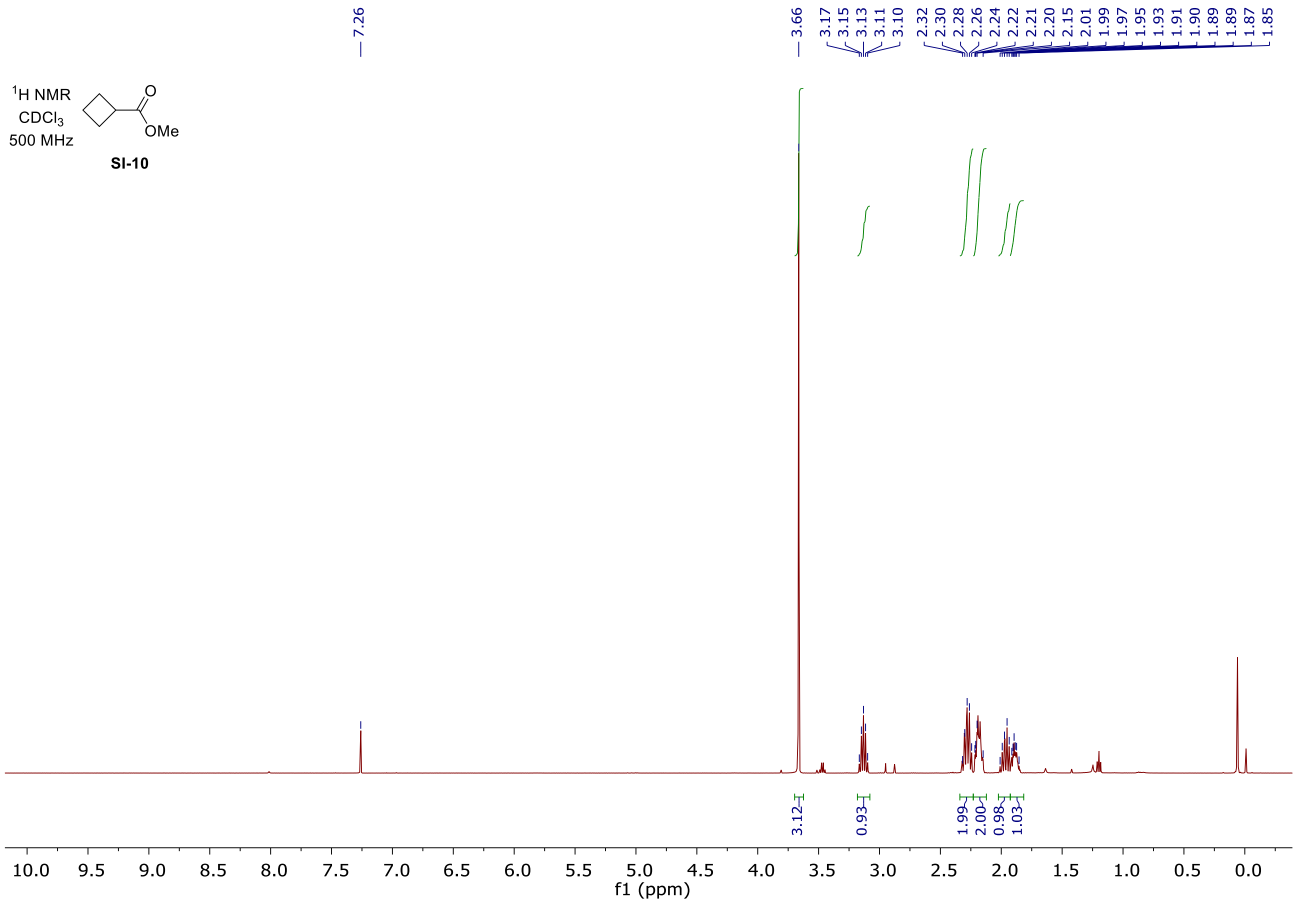

7.26
3.66
3.17
3.15
3.13
3.11
3.10
2.32
2.30
2.28
2.26
2.24
2.22
2.21
2.20
2.15
2.01
1.99
1.97
1.95
1.93
1.91
1.90
1.89
1.89
1.87
1.85
^{1}H NMR
$CDCl_3$
500 MHz
O
OMe
SI-10
3.12
0.93
1.99
2.00
0.98
1.03
10.0 9.5 9.0 8.5 8.0 7.5 7.0 6.5 6.0 5.5 5.0 4.5 4.0 3.5 3.0 2.5 2.0 1.5 1.0 0.5 0.0
f1 (ppm)

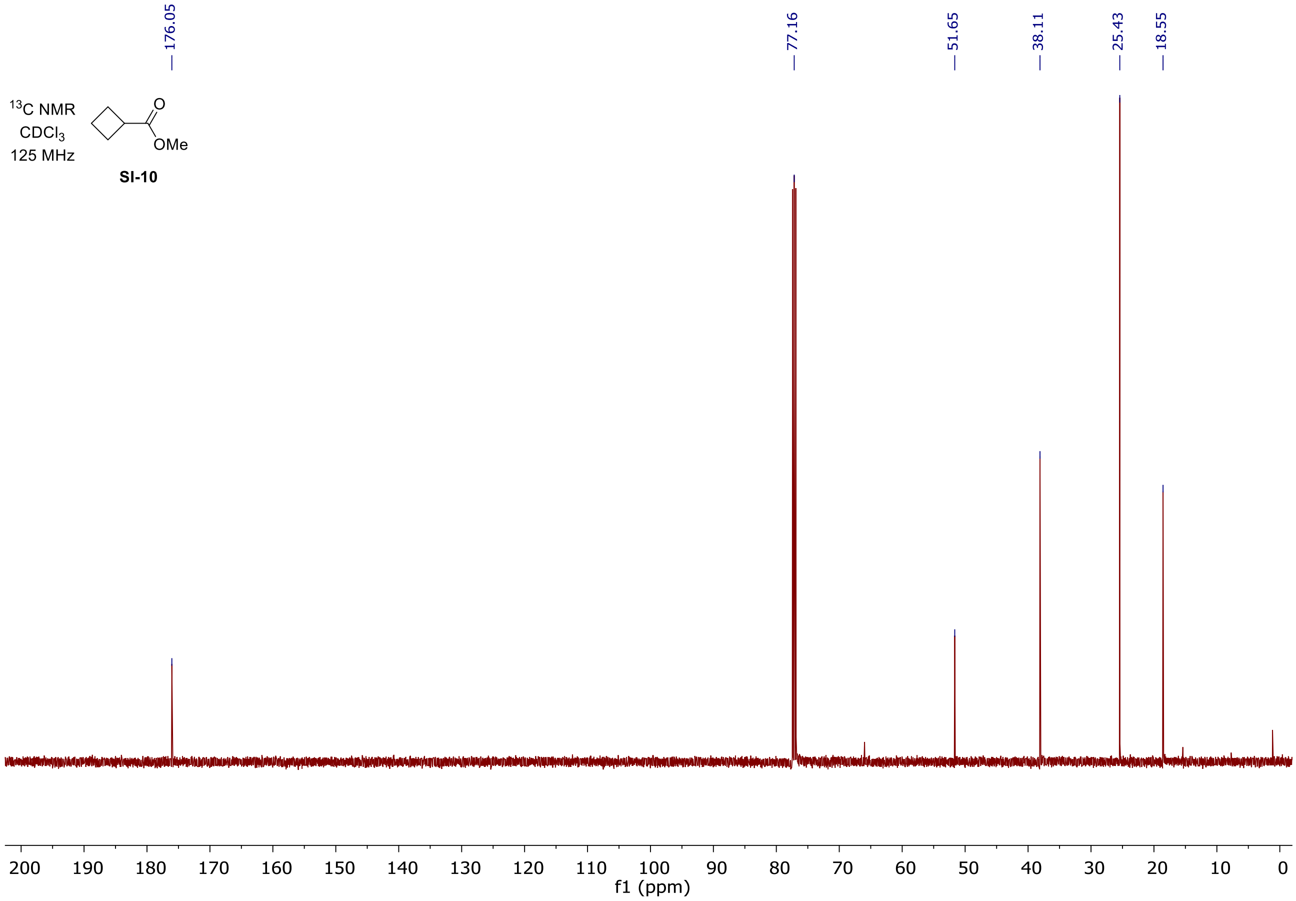

176.05
77.16
51.65
38.11
25.43
18.55
^{13}C NMR
$CDCl_3$
125 MHz
O
OMe
SI-10
200 190 180 170 160 150 140 130 120 110 100 90 80 70 60 50 40 30 20 10 0
f1 (ppm)

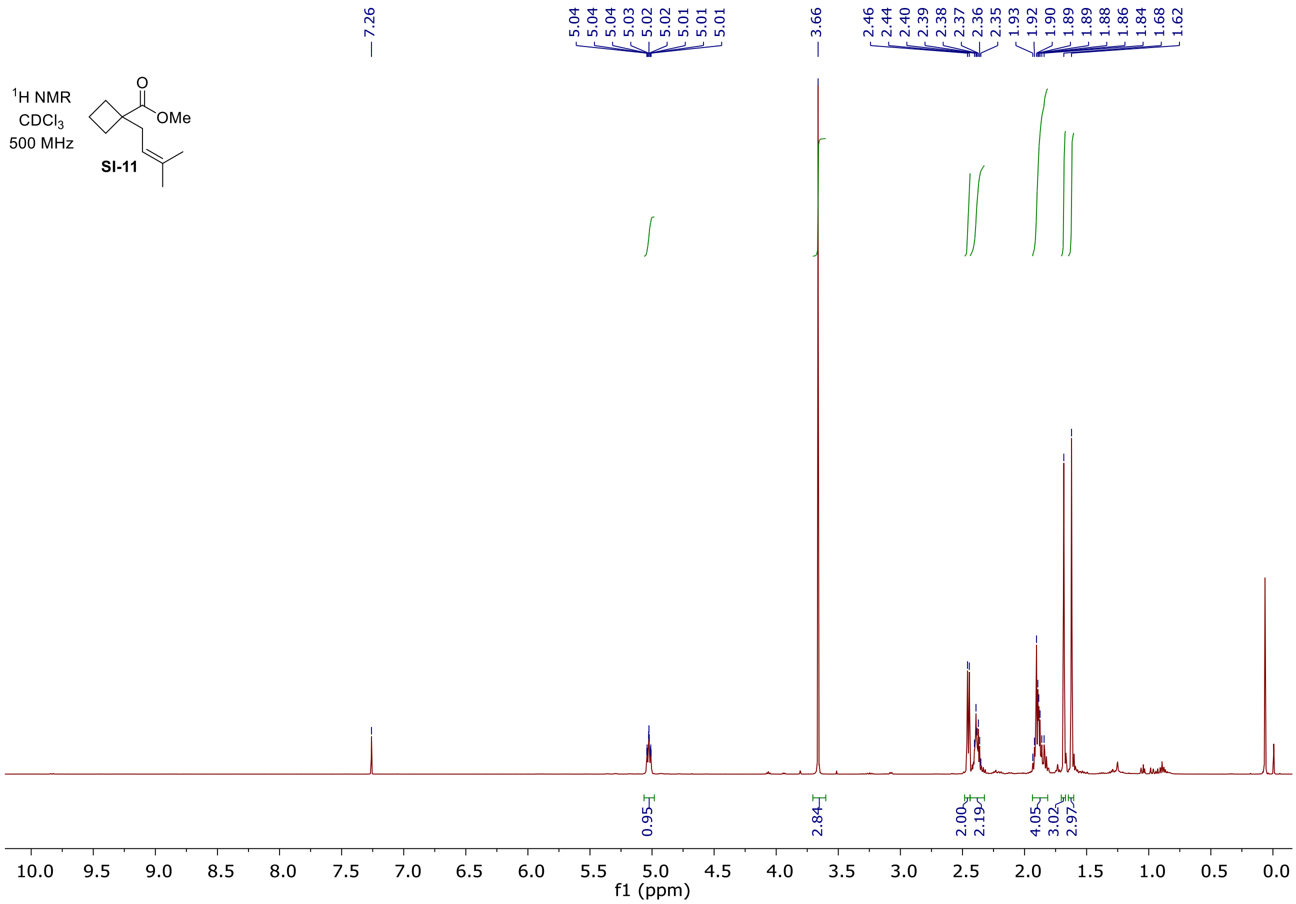

1H NMR
CDCl3
500 MHz
O
OMe
SI-11
7.26
5.04
5.04
5.04
5.03
5.02
5.02
5.01
5.01
5.01
3.66
2.46
2.44
2.40
2.39
2.38
2.37
2.36
2.35
1.93
1.92
1.90
1.89
1.89
1.88
1.86
1.84
1.68
1.62
0.95
2.84
2.00
2.19
4.05
3.02
2.97
10.0
9.5
9.0
8.5
8.0
7.5
7.0
6.5
6.0
5.5
5.0
4.5
4.0
3.5
3.0
2.5
2.0
1.5
1.0
0.5
0.0
f1 (ppm)

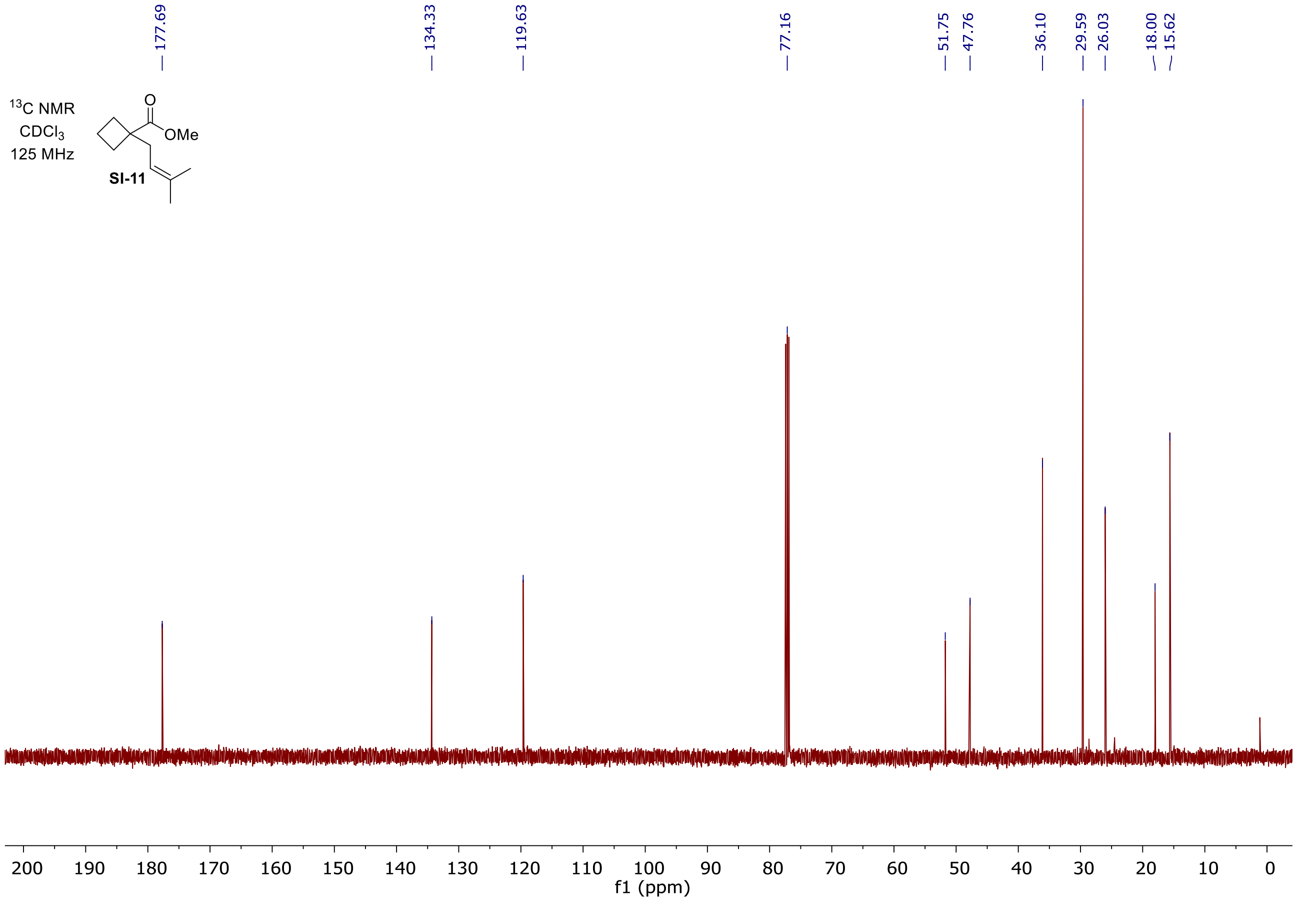

13C NMR
CDCl3
125 MHz
O
OMe
SI-11
177.69
134.33
119.63
77.16
51.75
47.76
36.10
29.59
26.03
18.00
15.62
200
190
180
170
160
150
140
130
120
110
100
90
80
70
60
50
40
30
20
10
0
f1 (ppm)

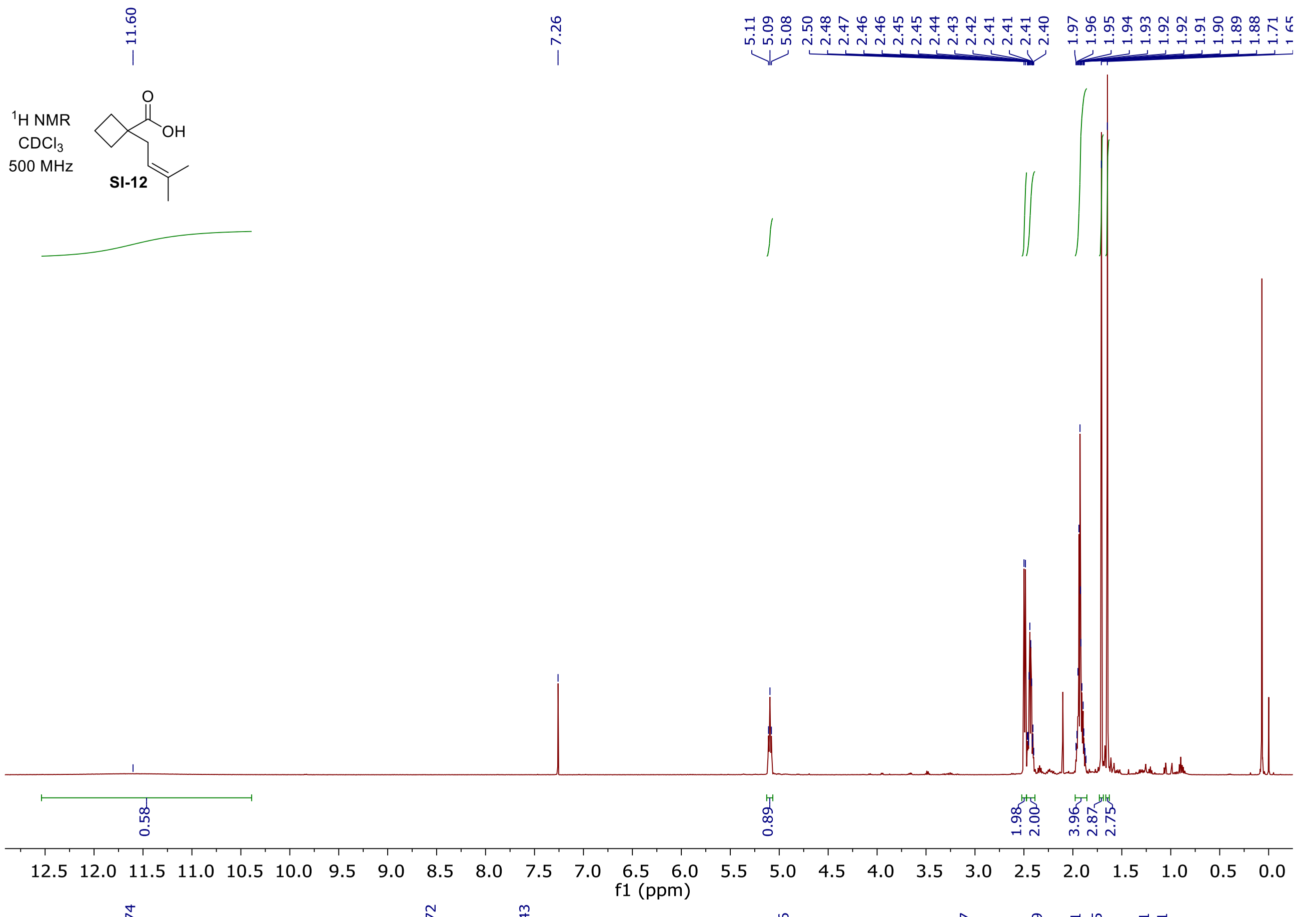

11.60
7.26
5.11
5.09
5.08
2.50
2.48
2.47
2.46
2.46
2.45
2.45
2.44
2.43
2.42
2.41
2.41
2.41
2.40
1.97
1.96
1.95
1.94
1.93
1.92
1.92
1.91
1.90
1.89
1.88
1.71
^{1}H NMR
$CDCl_3$
500 MHz
O
OH
SI-12
0.58
0.89
1.98
2.00
3.96
2.87
2.75
12.5 12.0 11.5 11.0 10.5 10.0 9.5 9.0 8.5 8.0 7.5 7.0 6.5 6.0 5.5 5.0 4.5 4.0 3.5 3.0 2.5 2.0 1.5 1.0 0.5 0.0
f1 (ppm)

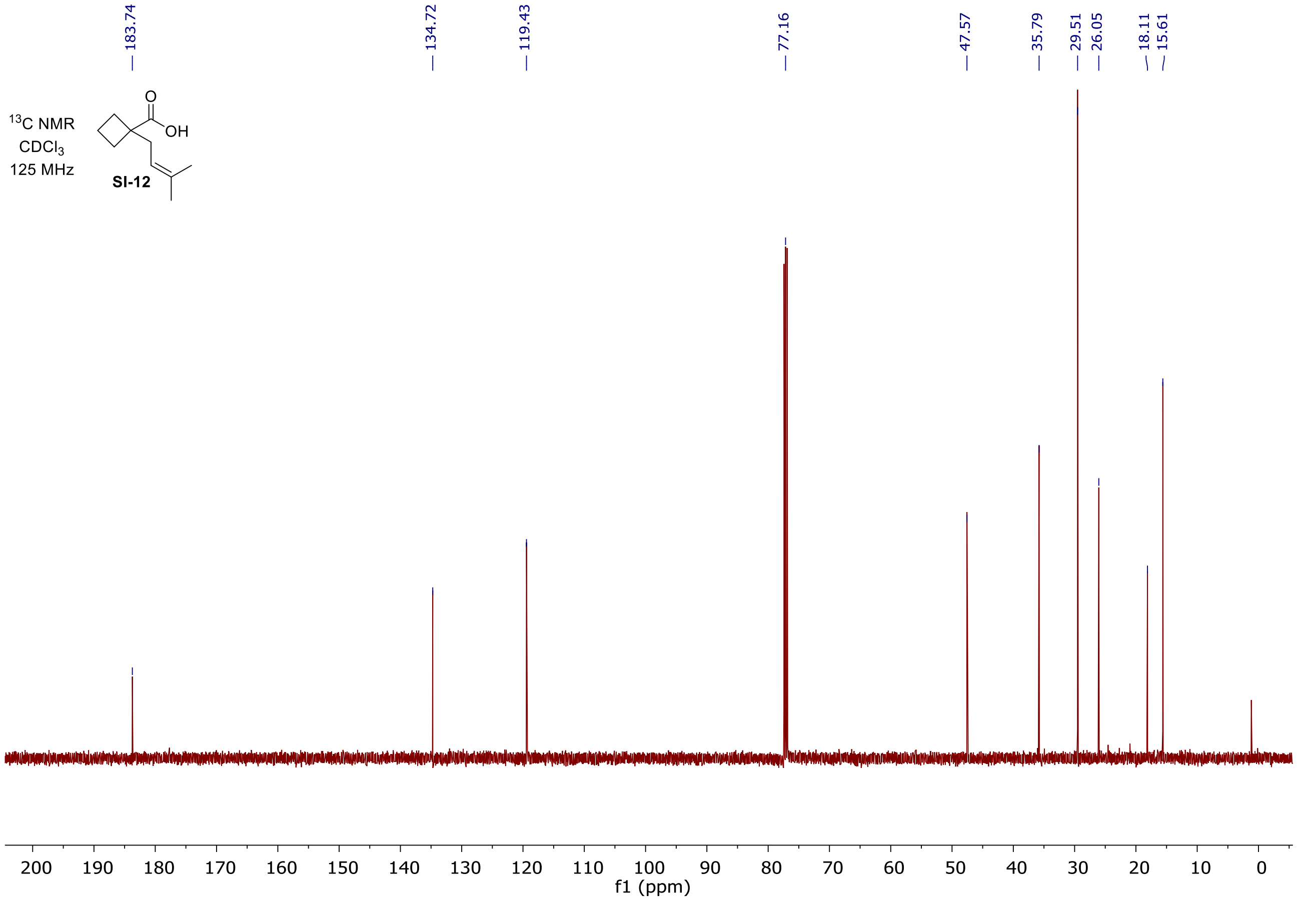

183.74
134.72
119.43
77.16
47.57
35.79
29.51
26.05
18.11
15.61
^{13}C NMR
$CDCl_3$
125 MHz
O
OH
SI-12
200 190 180 170 160 150 140 130 120 110 100 90 80 70 60 50 40 30 20 10 0
f1 (ppm)

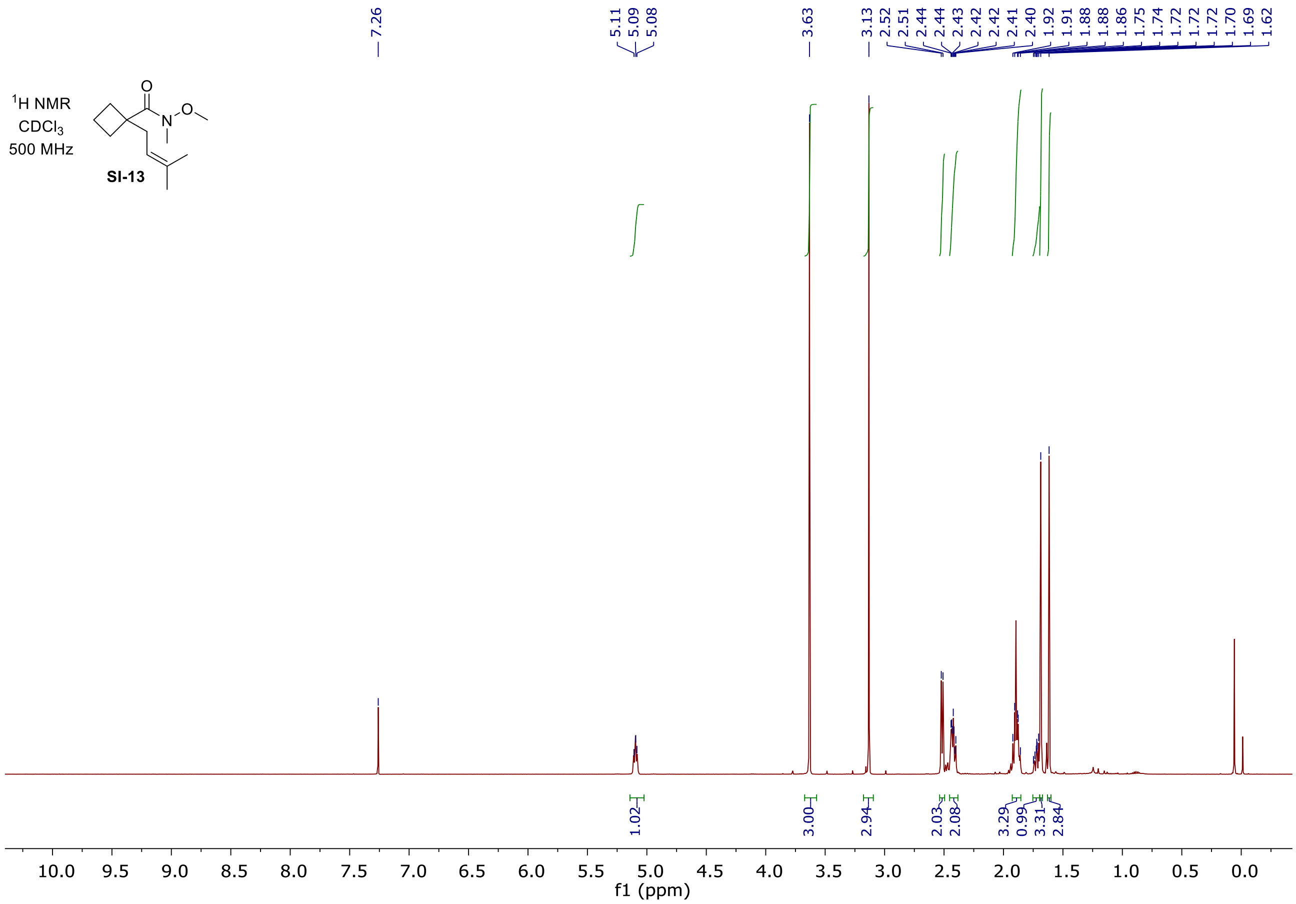

^{1}H NMR
CDCl3
500 MHz
SI-13
7.26
5.11
5.09
5.08
3.63
3.13
2.52
2.51
2.44
2.44
2.43
2.42
2.42
2.41
2.40
1.92
1.91
1.88
1.88
1.86
1.75
1.74
1.72
1.72
1.72
1.70
1.69
1.62
1.02
3.00
2.94
2.03
2.08
3.29
0.99
3.31
2.84
f1 (ppm)

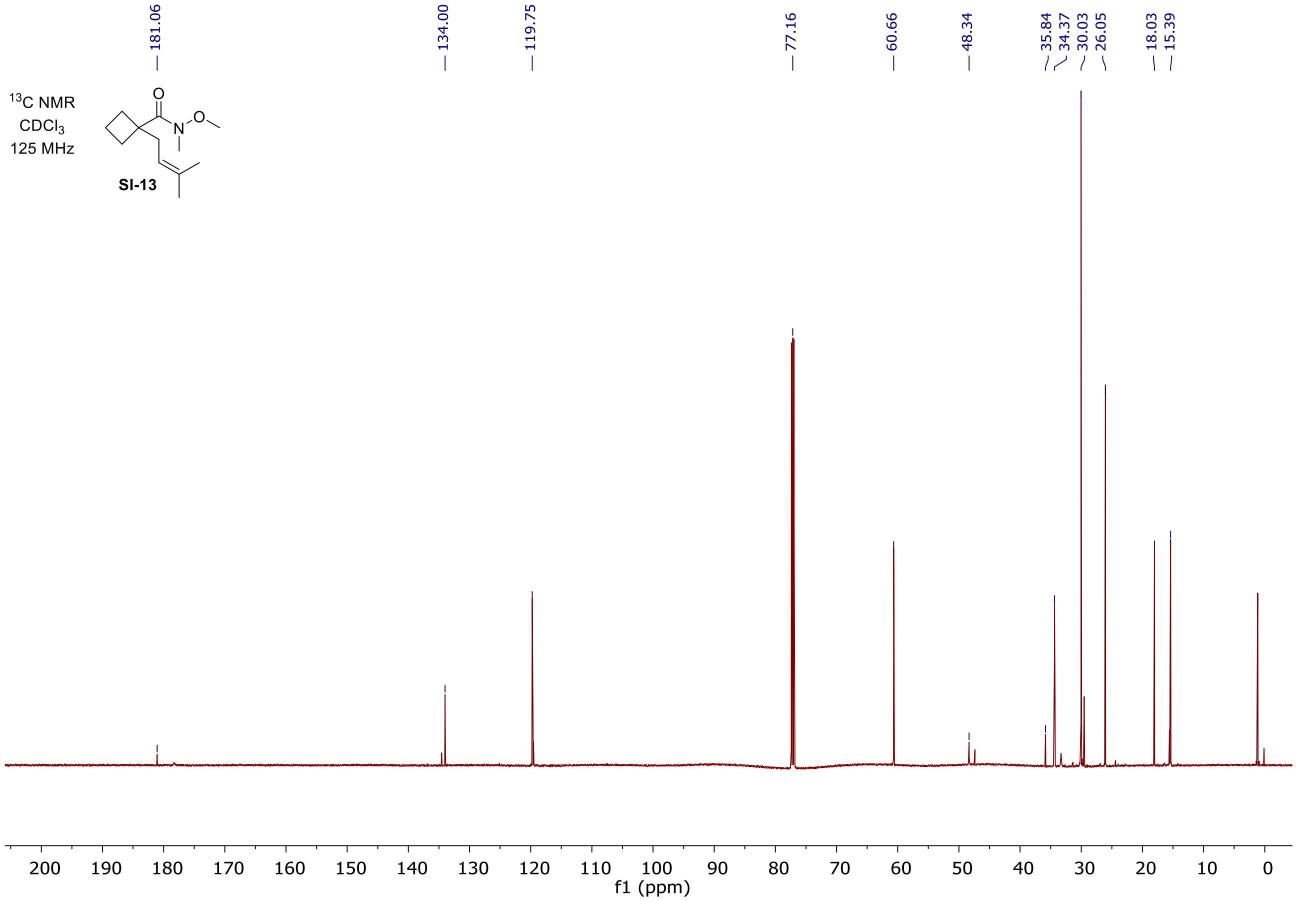

^{13}C NMR
CDCl3
125 MHz
SI-13
181.06
134.00
119.75
77.16
60.66
48.34
35.84
34.37
30.03
26.05
18.03
15.39
f1 (ppm)

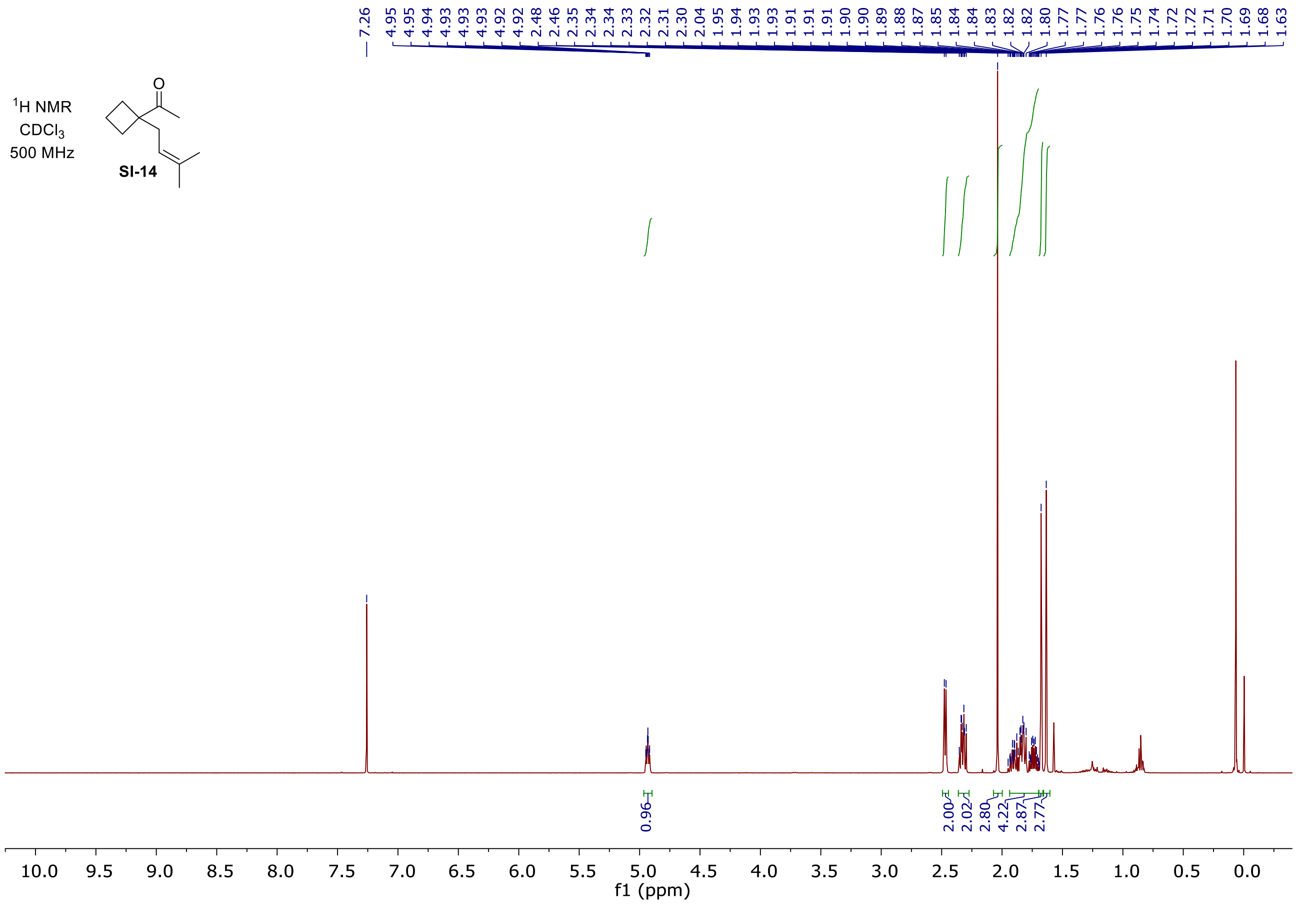

^{1}H NMR
$CDCl_3$
500 MHz
SI-14
O
7.26
4.95 4.95 4.94 4.93 4.93 4.93 4.92 4.92 2.48 2.46 2.35 2.34 2.34 2.33 2.32 2.31 2.30 2.04 1.95 1.94 1.93 1.93 1.91 1.91 1.91 1.90 1.89 1.88 1.87 1.85 1.84 1.84 1.83 1.82 1.82 1.80 1.77 1.76 1.76 1.75 1.74 1.72 1.72 1.71 1.70 1.69 1.68 1.63
0.96
2.00
2.02
2.80
4.22
2.87
2.77
10.0 9.5 9.0 8.5 8.0 7.5 7.0 6.5 6.0 5.5 5.0 4.5 4.0 3.5 3.0 2.5 2.0 1.5 1.0 0.5 0.0
f1 (ppm)

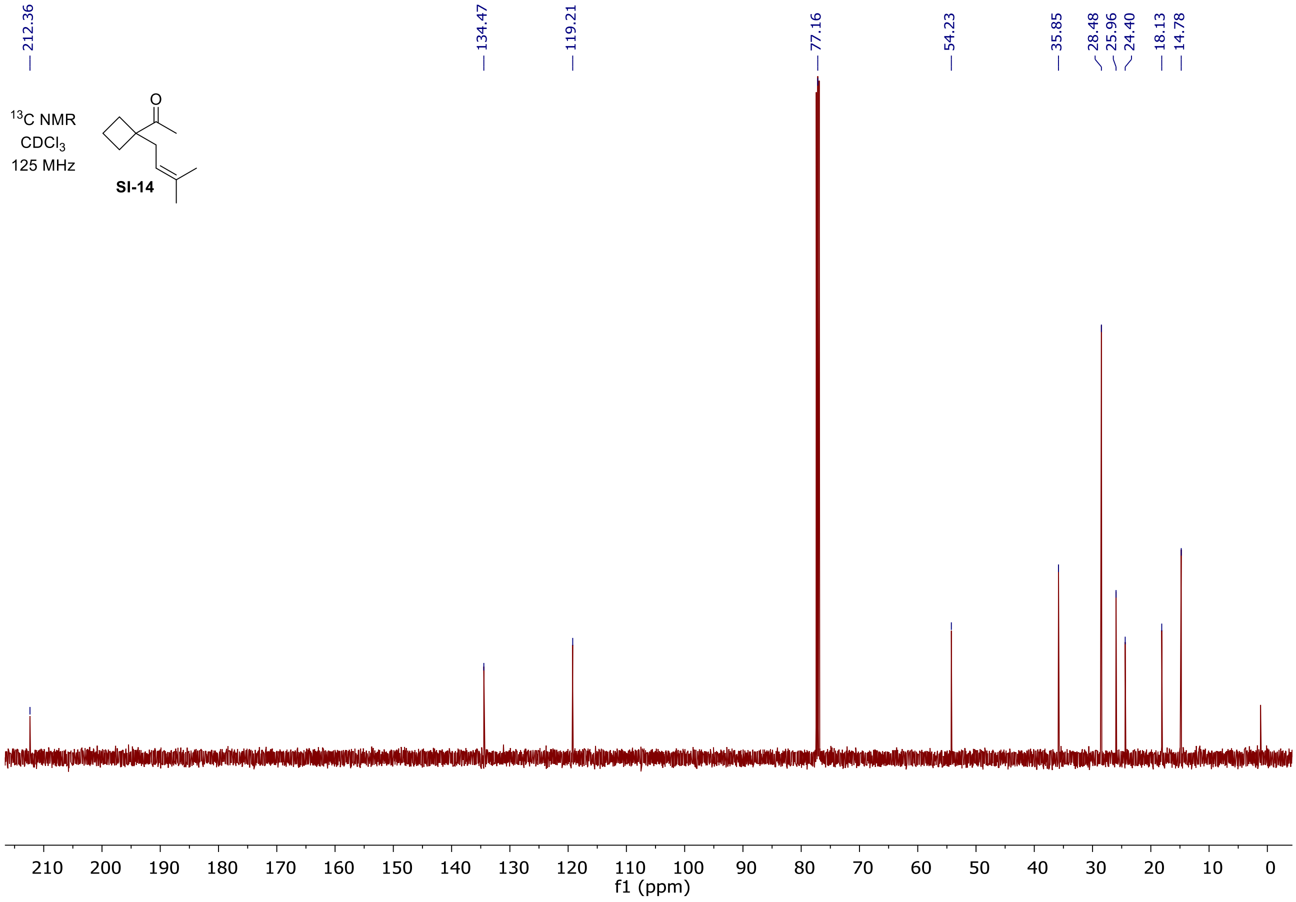

^{13}C NMR
$CDCl_3$
125 MHz
SI-14
O
212.36
134.47
119.21
77.16
54.23
35.85
28.48
25.96
24.40
18.13
14.78
210 200 190 180 170 160 150 140 130 120 110 100 90 80 70 60 50 40 30 20 10 0
f1 (ppm)

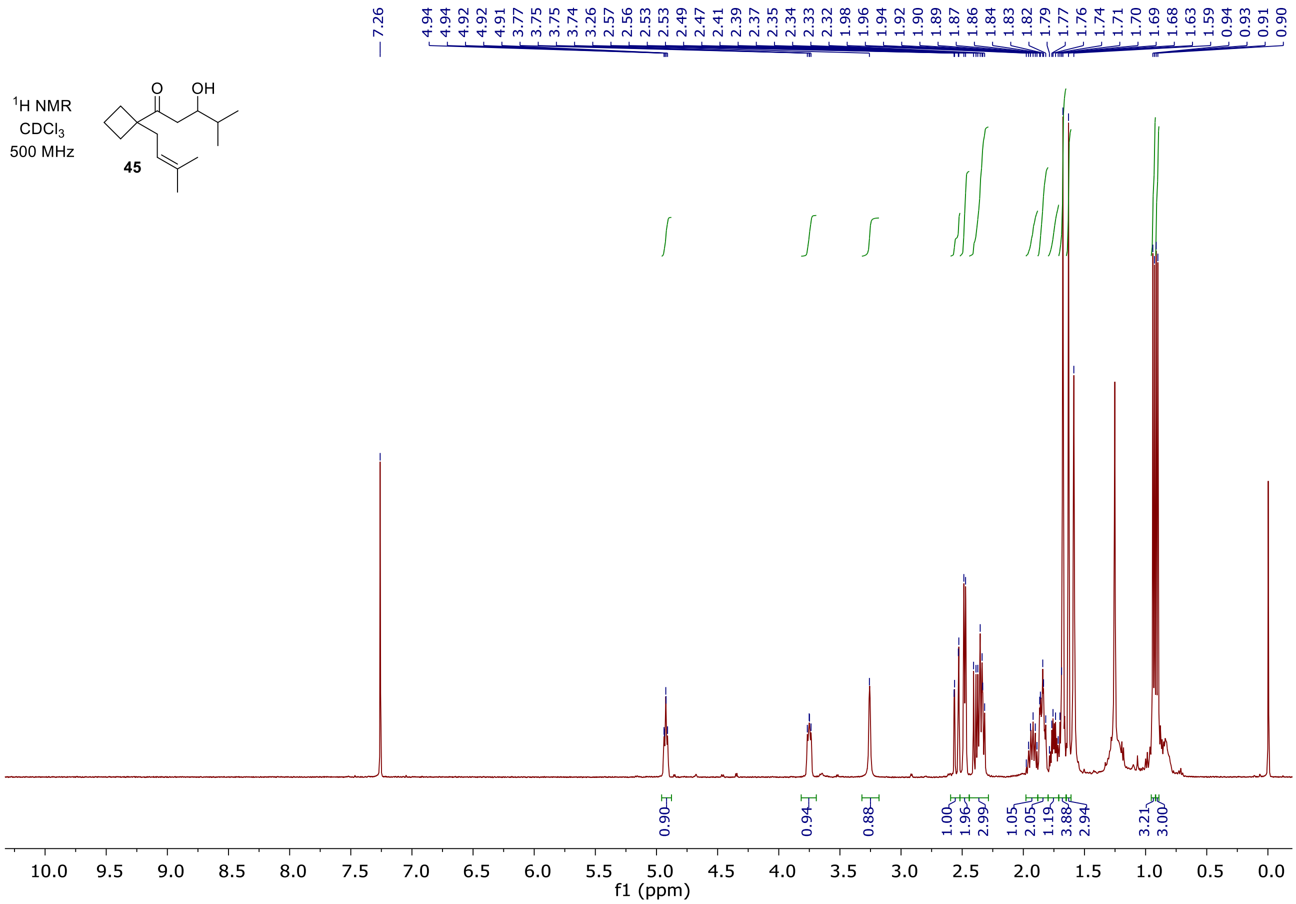

1H NMR
CDCl3
500 MHz
45
O
OH
7.26
4.94 4.94 4.92 4.92 4.91 3.77 3.75 3.75 3.74 3.26 2.57 2.56 2.53 2.53 2.49 2.47 2.41 2.39 2.37 2.35 2.34 2.33 2.32 1.98 1.96 1.94 1.92 1.90 1.89 1.87 1.86 1.84 1.83 1.82 1.79 1.77 1.76 1.74 1.71 1.70 1.69 1.68 1.63 1.59 0.94 0.93 0.91 0.90
0.90 0.94 0.88 1.00 1.96 2.99 1.05 2.05 1.19 3.88 2.94 3.21 3.00
f1 (ppm)

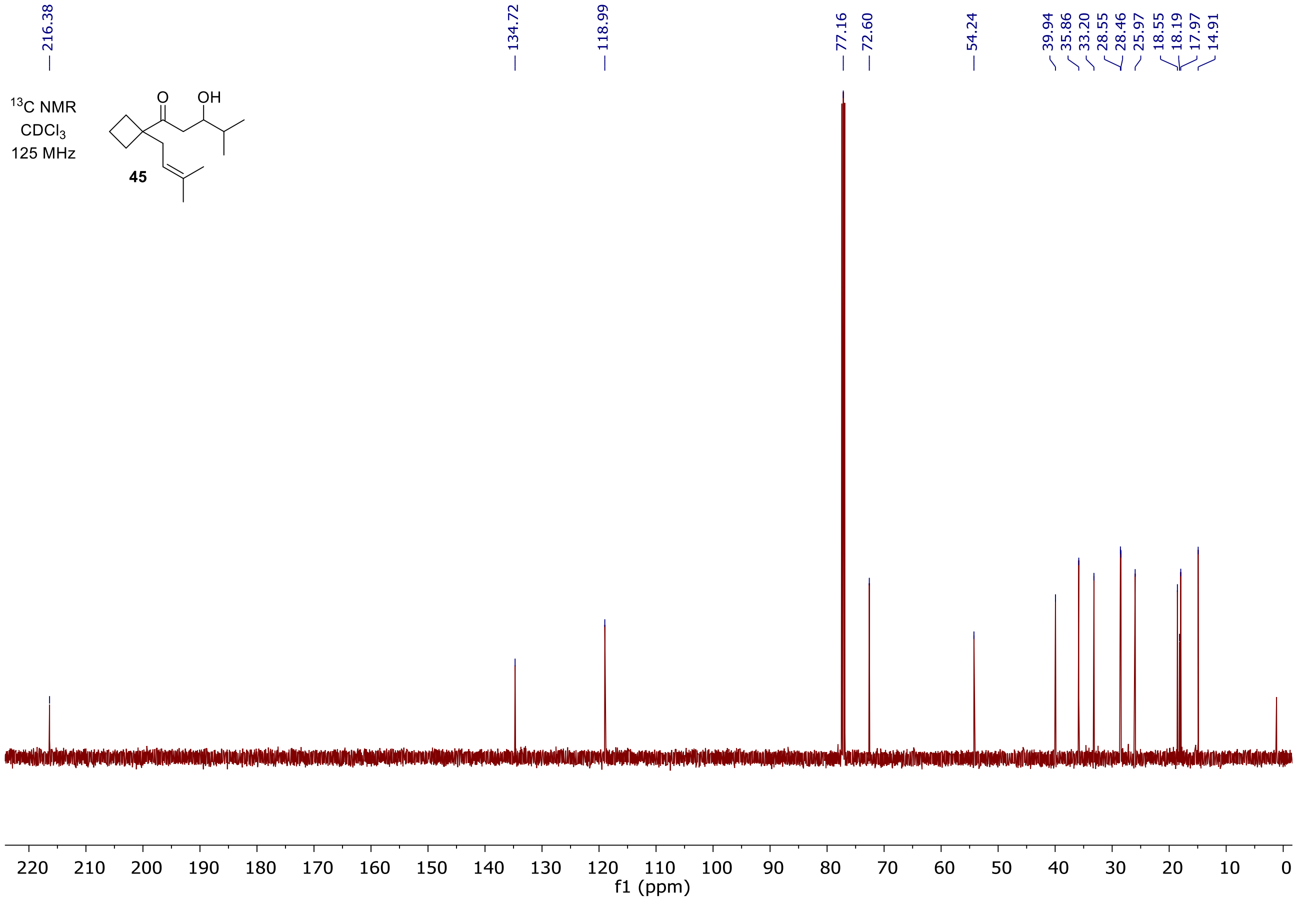

13C NMR
CDCl3
125 MHz
45
O
OH
216.38 134.72 118.99 77.16 72.60 54.24 39.94 35.86 33.20 28.55 28.46 25.97 18.55 18.19 17.97 14.91
f1 (ppm)

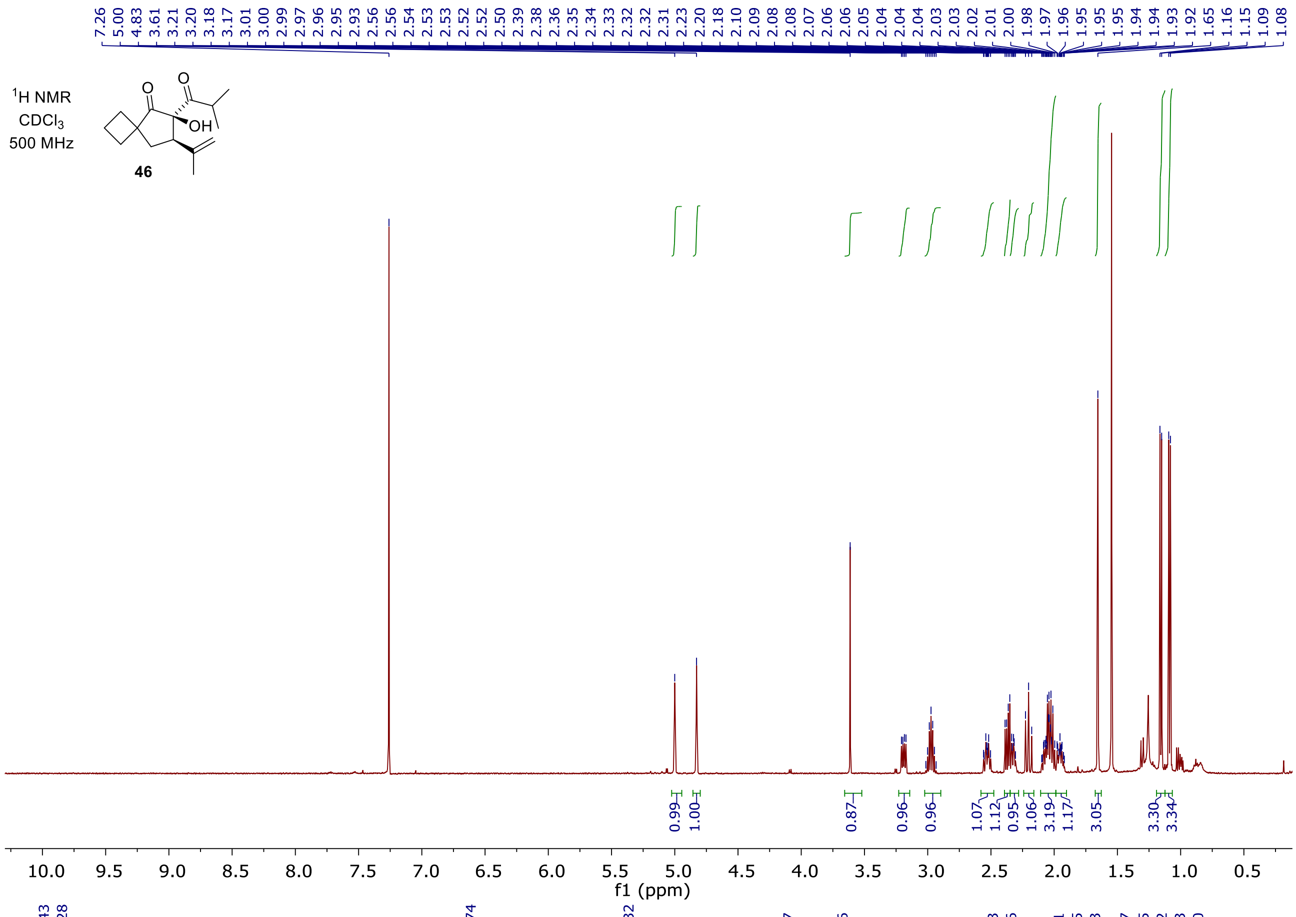
^{1}H NMR
$CDCl_3$
500 MHz
46
f1 (ppm)

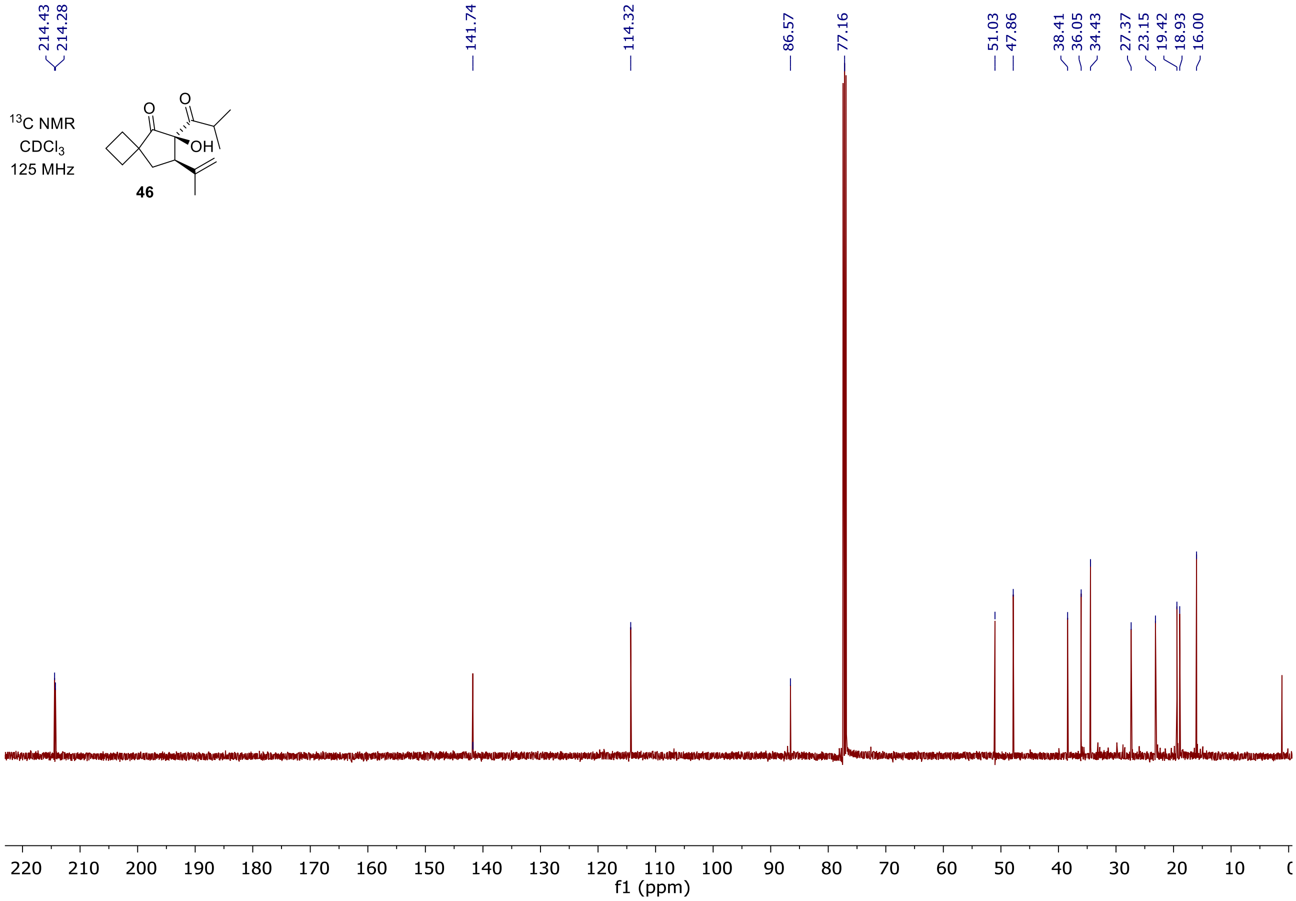
^{13}C NMR
$CDCl_3$
125 MHz
46
f1 (ppm)

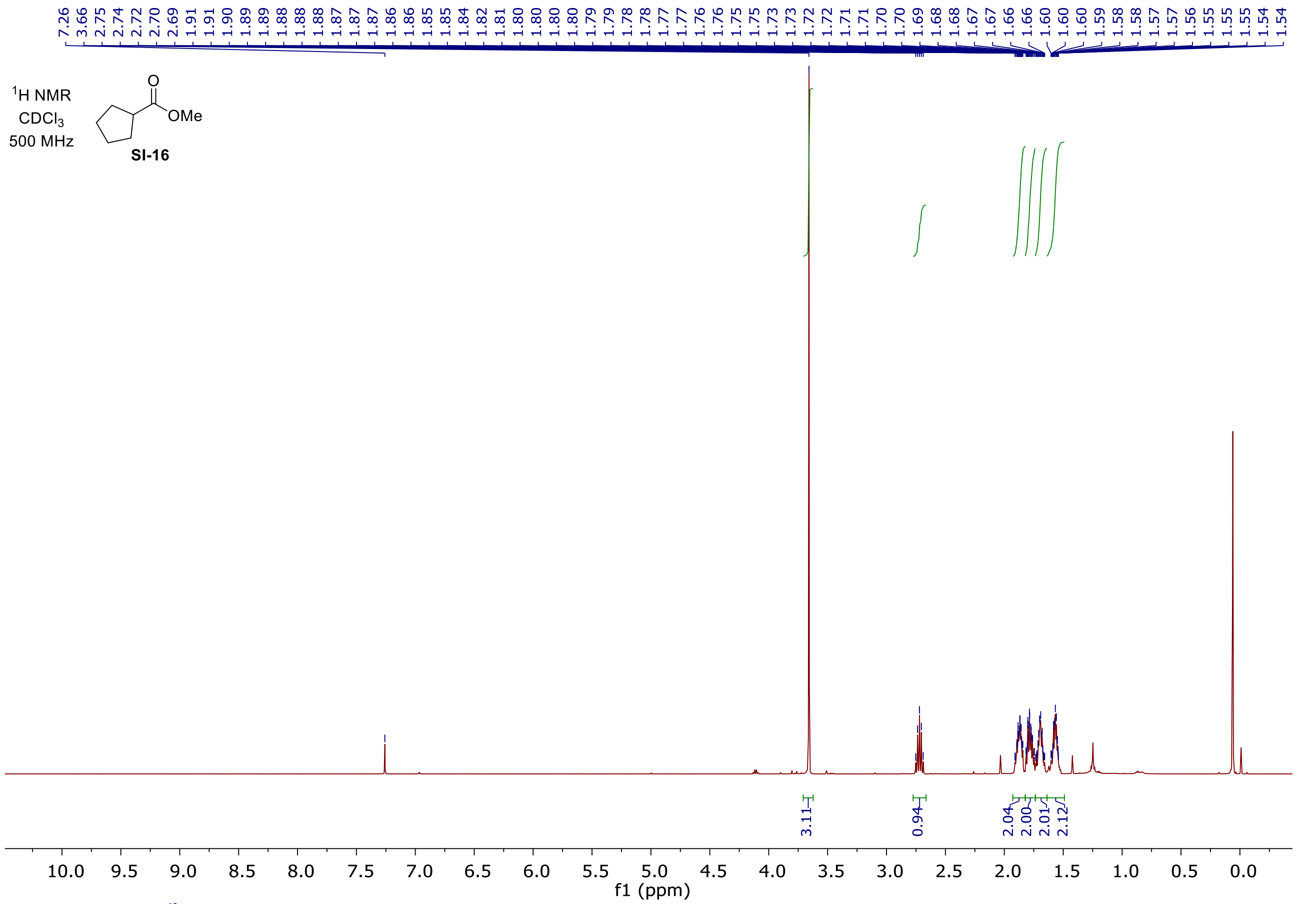
7.26 3.66 2.75 2.74 2.72 2.70 2.69 1.91 1.91 1.90 1.89 1.89 1.88 1.88 1.88 1.87 1.87 1.87 1.86 1.86 1.85 1.85 1.84 1.82 1.81 1.80 1.80 1.80 1.80 1.79 1.79 1.78 1.78 1.77 1.77 1.76 1.76 1.75 1.75 1.73 1.73 1.72 1.72 1.71 1.71 1.70 1.70 1.69 1.68 1.68 1.67 1.67 1.66 1.66 1.60 1.60 1.60 1.59 1.58 1.58 1.57 1.57 1.56 1.55 1.55 1.55 1.54 1.54
^{1}H NMR
$CDCl_3$
500 MHz
O
OMe
SI-16
3.11
0.94
2.04
2.00
2.01
2.12
10.0 9.5 9.0 8.5 8.0 7.5 7.0 6.5 6.0 5.5 5.0 4.5 4.0 3.5 3.0 2.5 2.0 1.5 1.0 0.5 0.0
f1 (ppm)

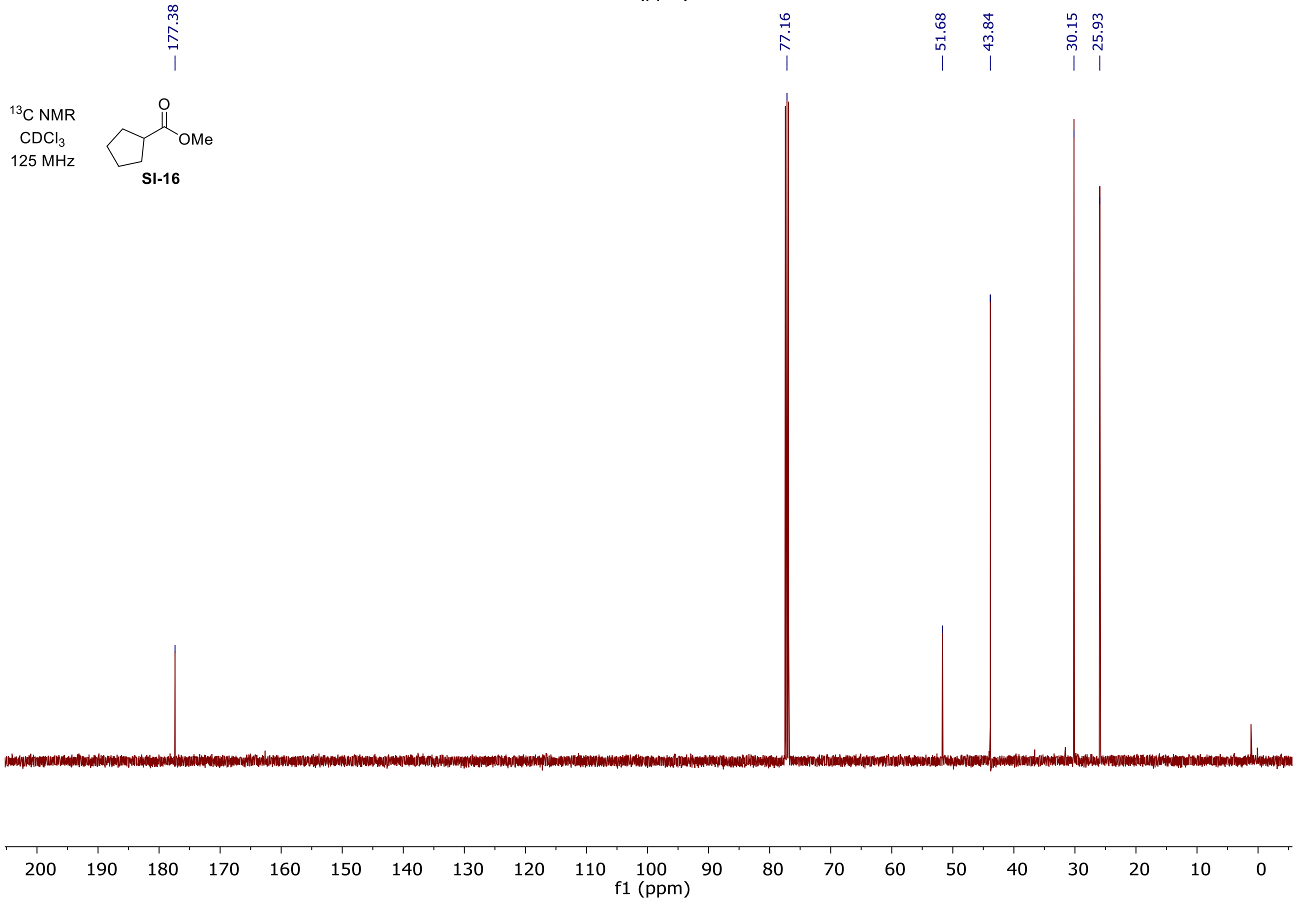
177.38
77.16
51.68
43.84
30.15
25.93
^{13}C NMR
$CDCl_3$
125 MHz
O
OMe
SI-16
200 190 180 170 160 150 140 130 120 110 100 90 80 70 60 50 40 30 20 10 0
f1 (ppm)

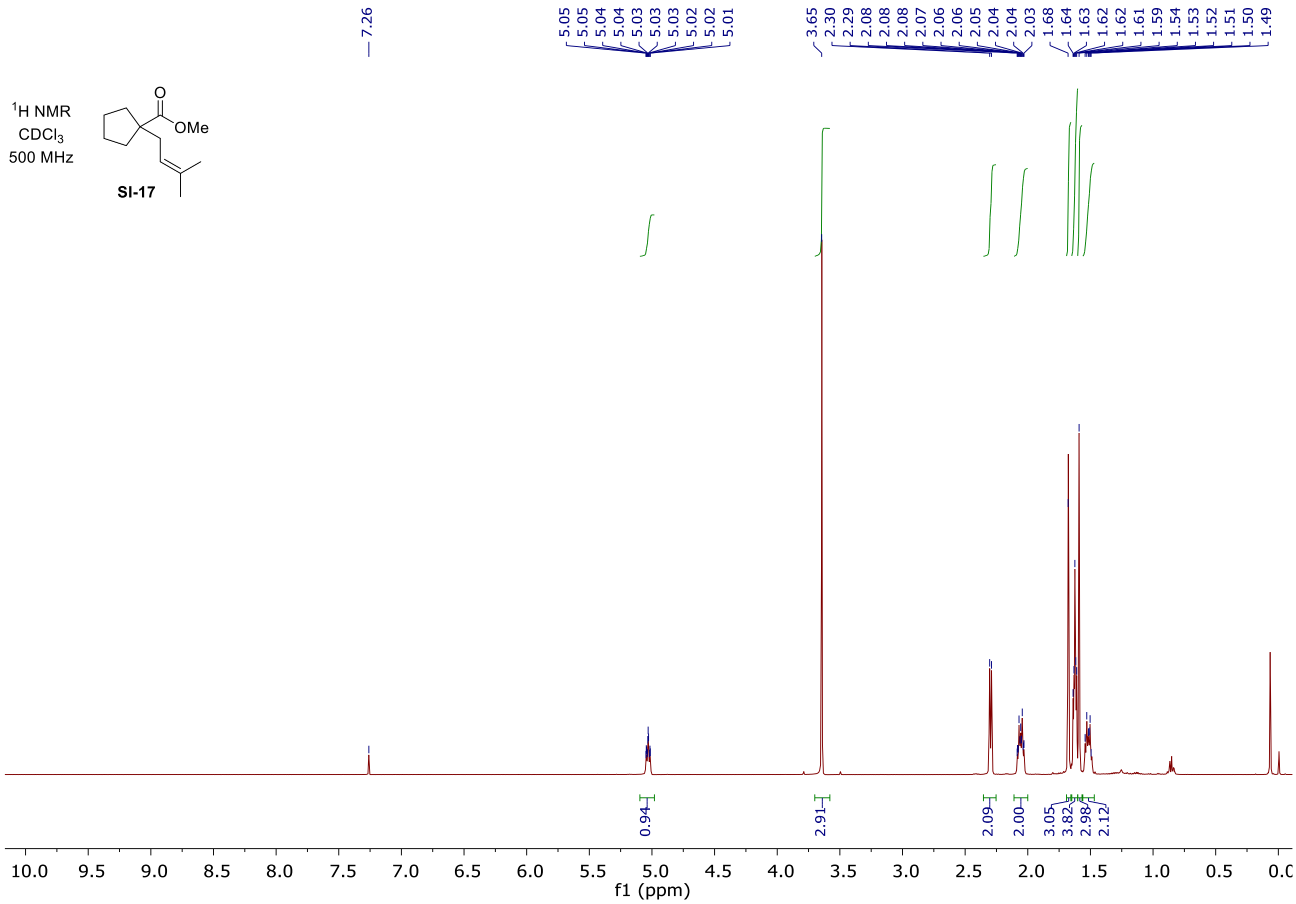

^{1}H NMR
CDCl$_3$
500 MHz
O
OMe
SI-17
7.26
5.05 5.05 5.04 5.04 5.03 5.03 5.03 5.02 5.02 5.01
3.65 2.30 2.29 2.08 2.08 2.08 2.07 2.06 2.06 2.05 2.04 2.04 2.03 1.68 1.64 1.63 1.62 1.62 1.61 1.59 1.54 1.53 1.52 1.51 1.50 1.49
0.94
2.91
2.09
2.00
3.05
3.82
2.98
2.12
f1 (ppm)

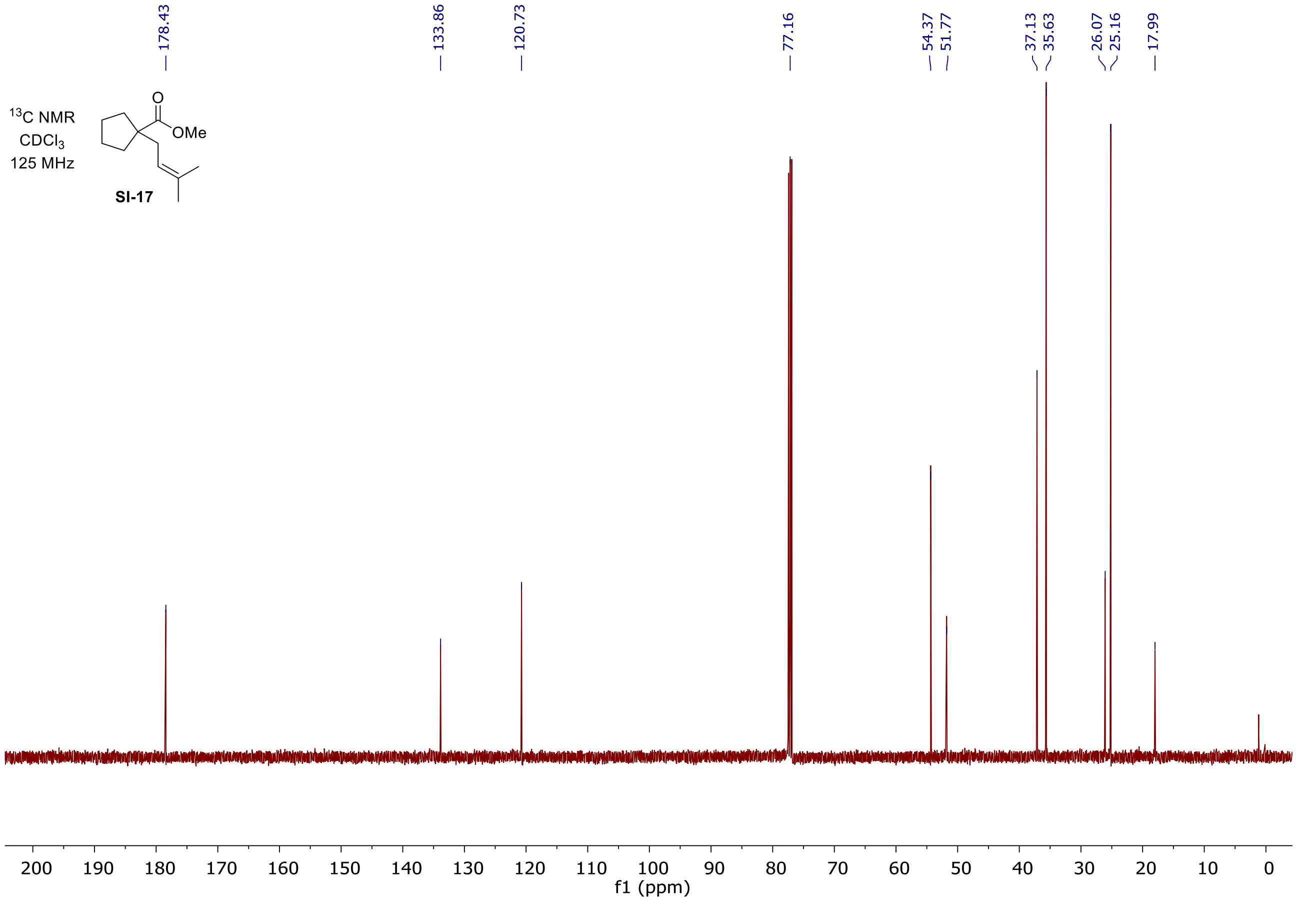

^{13}C NMR
CDCl$_3$
125 MHz
O
OMe
SI-17
178.43
133.86
120.73
77.16
54.37
51.77
37.13
35.63
26.07
25.16
17.99
f1 (ppm)

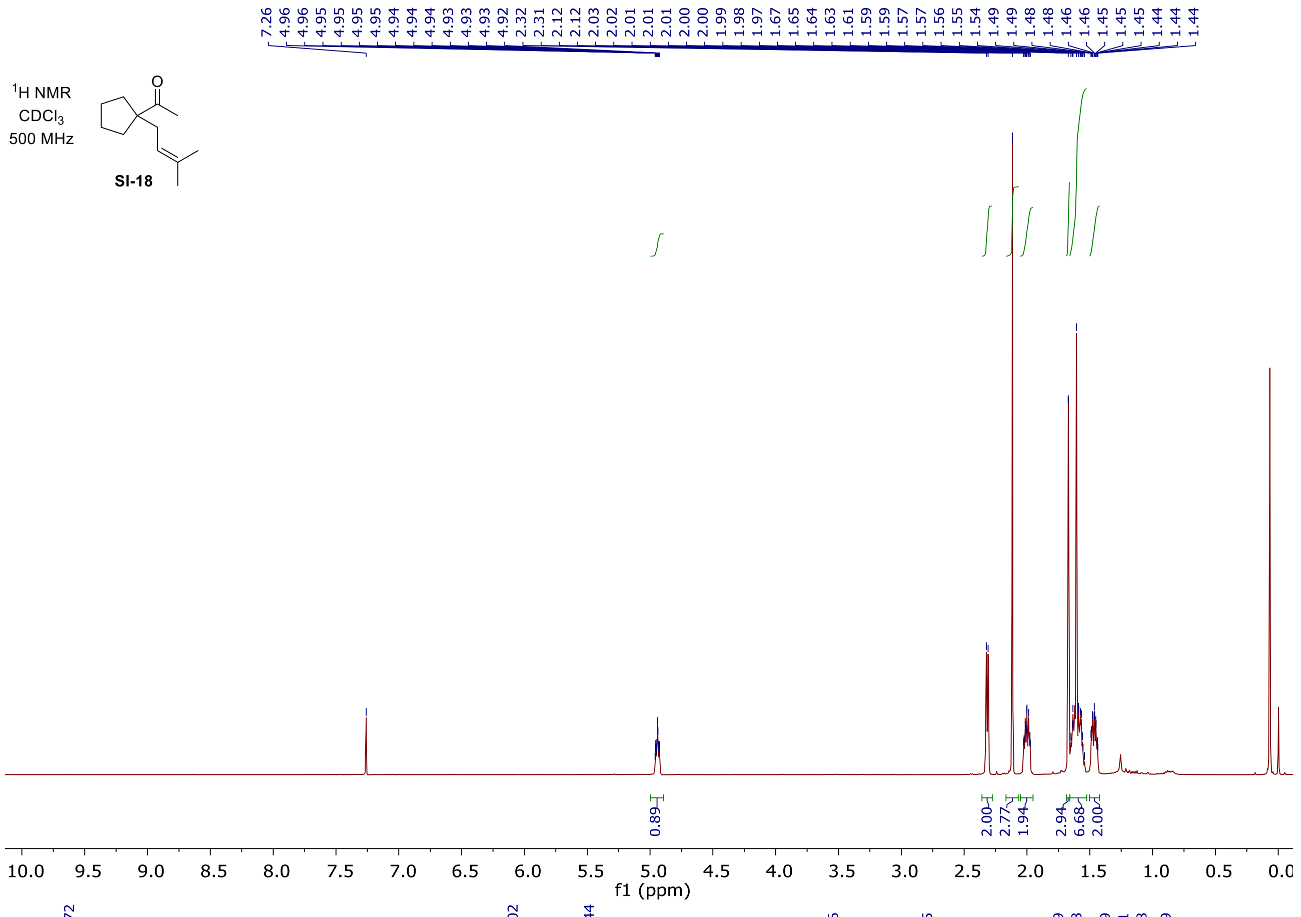
7.26 4.96 4.96 4.95 4.95 4.95 4.95 4.94 4.94 4.94 4.93 4.93 4.93 4.92 2.32 2.31 2.12 2.12 2.03 2.02 2.01 2.01 2.00 2.00 1.99 1.98 1.97 1.67 1.65 1.64 1.63 1.61 1.59 1.59 1.57 1.57 1.56 1.55 1.54 1.49 1.49 1.48 1.48 1.46 1.46 1.45 1.45 1.45 1.44 1.44 1.44
1H NMR
CDCl3
500 MHz
O
SI-18
0.89
2.00
2.77
1.94
2.94
6.68
2.00
10.0 9.5 9.0 8.5 8.0 7.5 7.0 6.5 6.0 5.5 5.0 4.5 4.0 3.5 3.0 2.5 2.0 1.5 1.0 0.5 0.0
f1 (ppm)

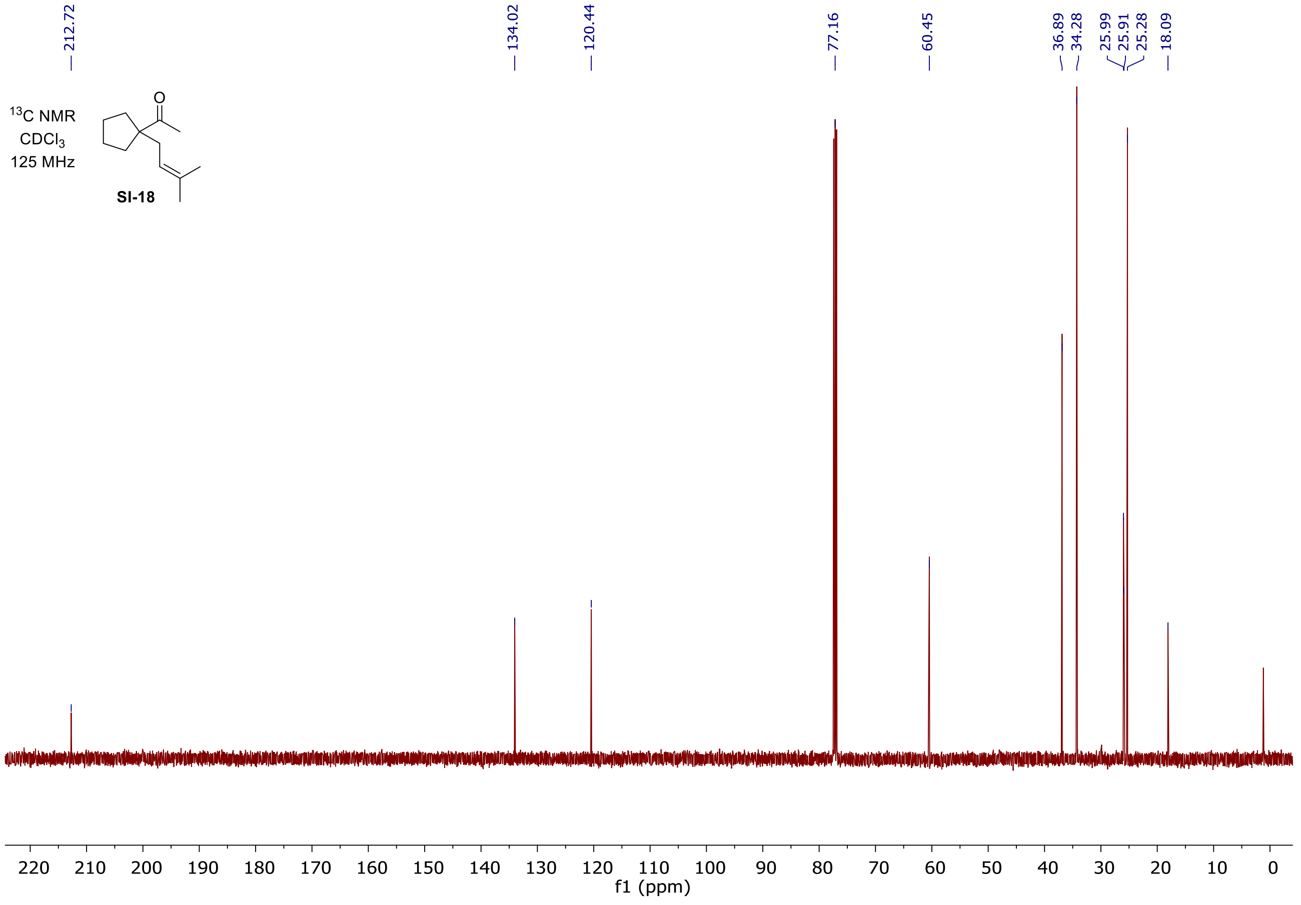
212.72
134.02
120.44
77.16
60.45
36.89
34.28
25.99
25.91
25.28
18.09
13C NMR
CDCl3
125 MHz
O
SI-18
220 210 200 190 180 170 160 150 140 130 120 110 100 90 80 70 60 50 40 30 20 10 0
f1 (ppm)

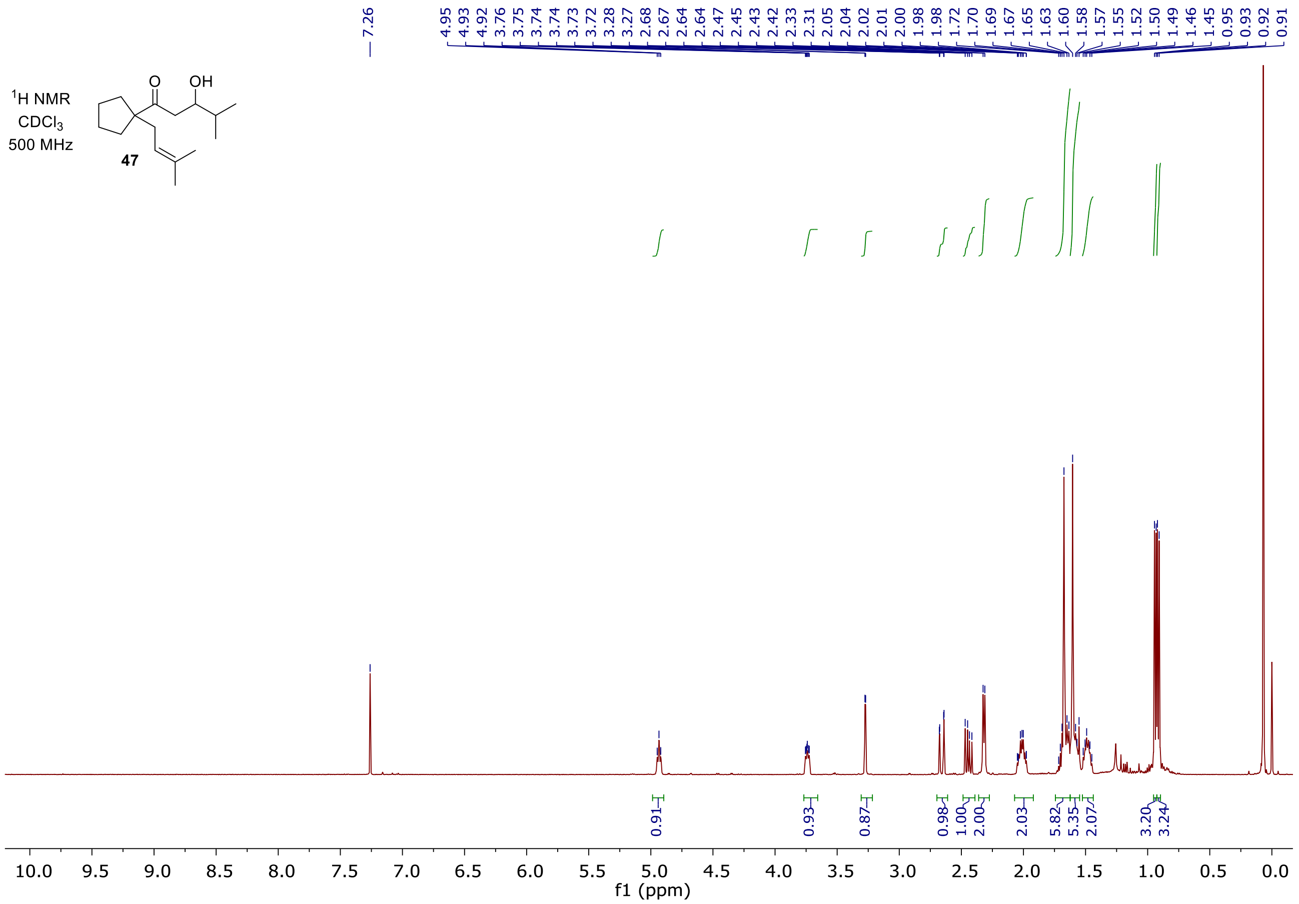
1H NMR
CDCl3
500 MHz
O
OH
47
7.26
4.95 4.93 4.92 3.76 3.75 3.74 3.74 3.73 3.72 3.28 3.27 2.68 2.67 2.64 2.64 2.47 2.45 2.43 2.42 2.33 2.31 2.05 2.04 2.02 2.01 2.00 1.98 1.98 1.72 1.70 1.69 1.67 1.65 1.63 1.60 1.58 1.57 1.55 1.52 1.50 1.49 1.46 1.45 0.95 0.93 0.92 0.91
0.91 0.93 0.87 0.98 1.00 2.00 2.03 5.82 5.35 2.07 3.20 3.24
10.0 9.5 9.0 8.5 8.0 7.5 7.0 6.5 6.0 5.5 5.0 4.5 4.0 3.5 3.0 2.5 2.0 1.5 1.0 0.5 0.0
f1 (ppm)

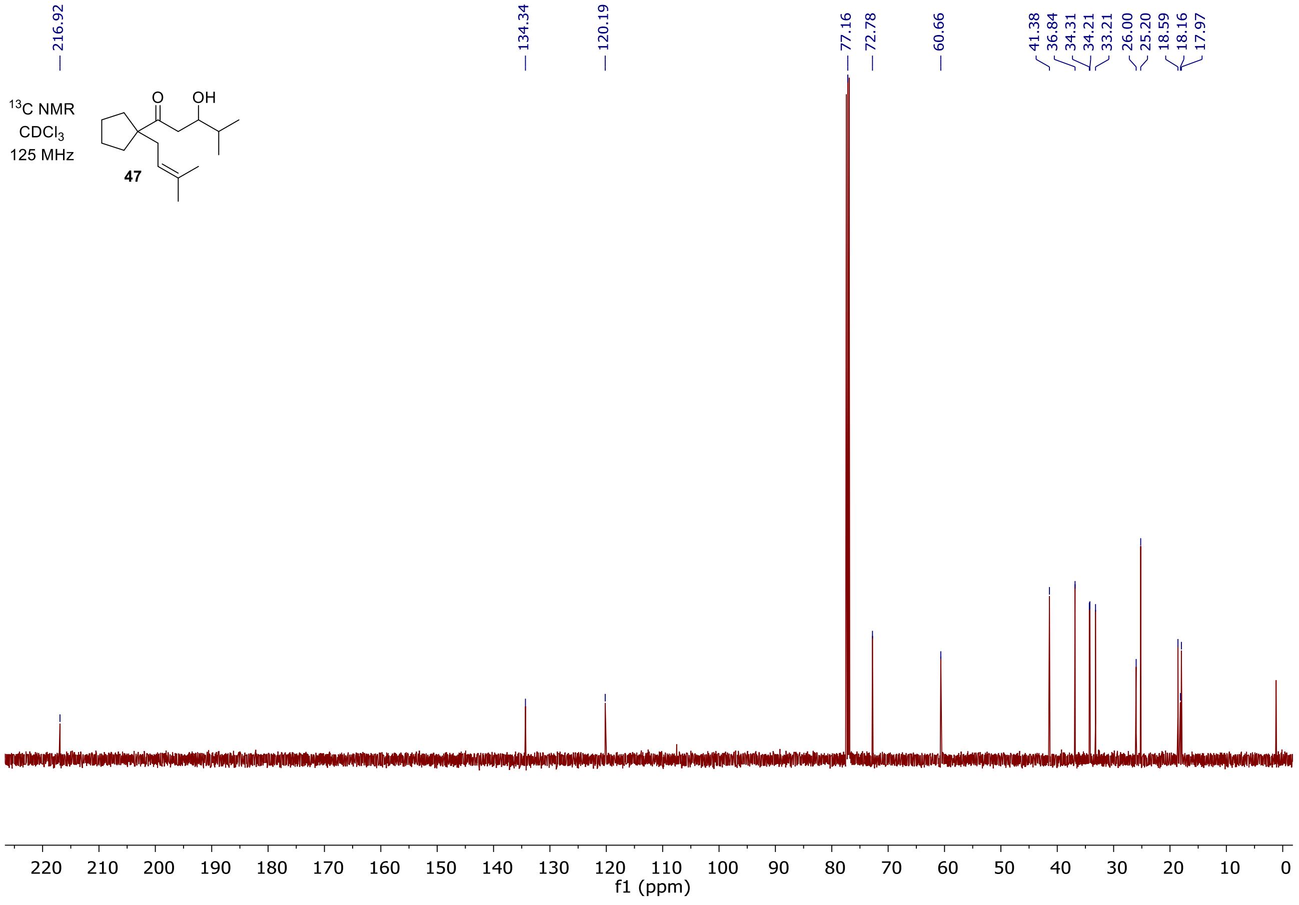
13C NMR
CDCl3
125 MHz
O
OH
47
216.92
134.34
120.19
77.16
72.78
60.66
41.38 36.84 34.31 34.21 33.21 26.00 25.20 18.59 18.16 17.97
220 210 200 190 180 170 160 150 140 130 120 110 100 90 80 70 60 50 40 30 20 10 0
f1 (ppm)

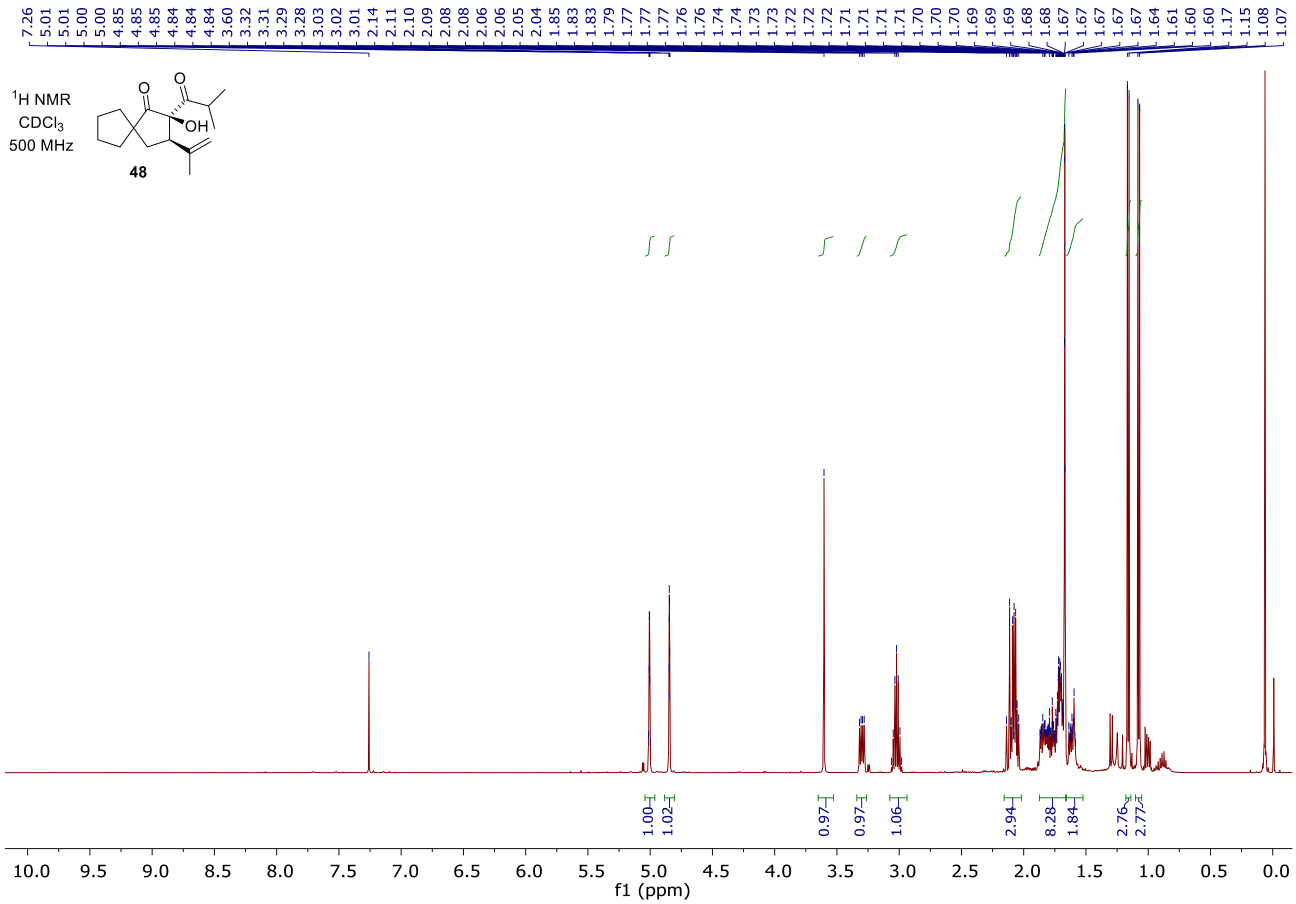

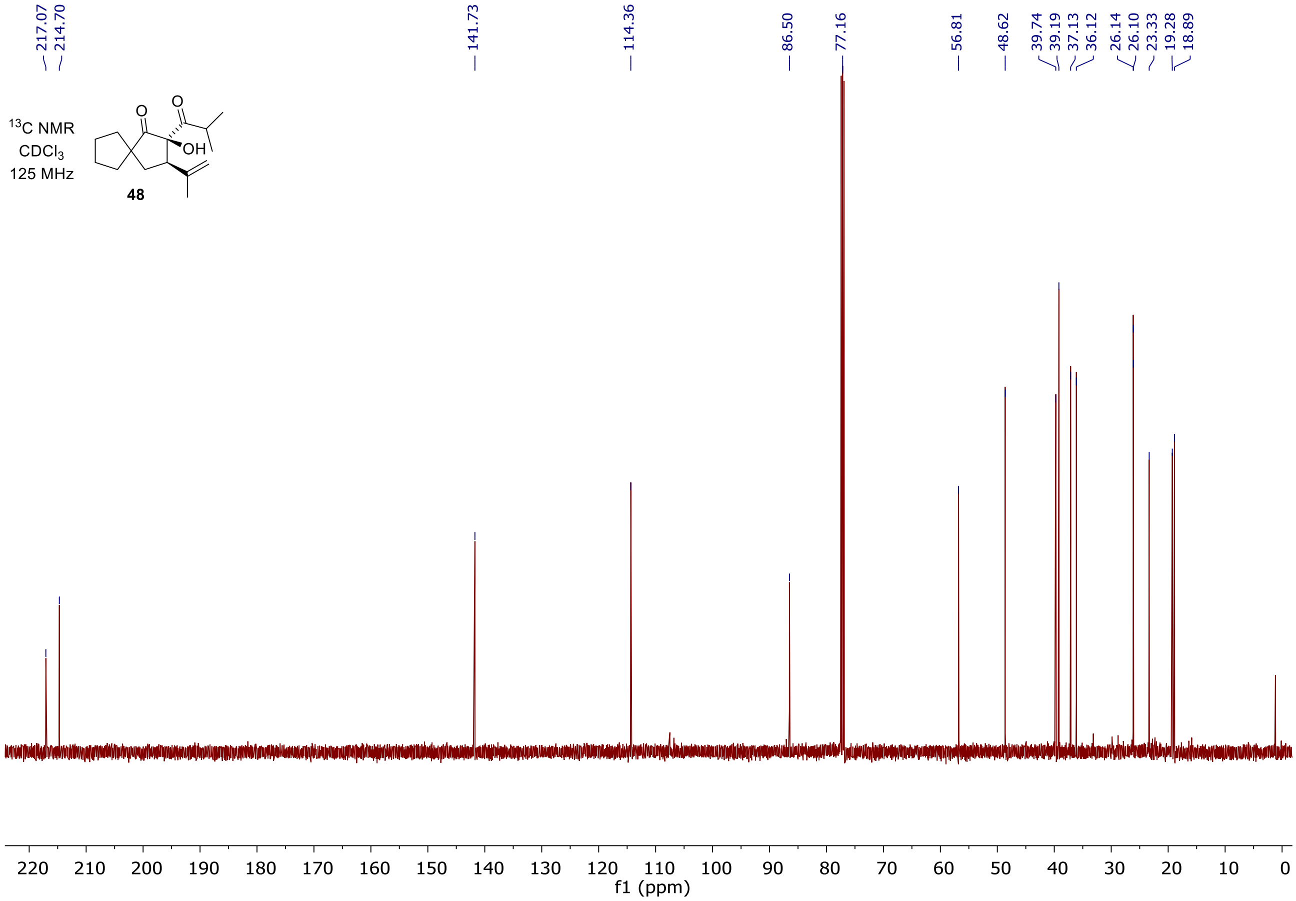

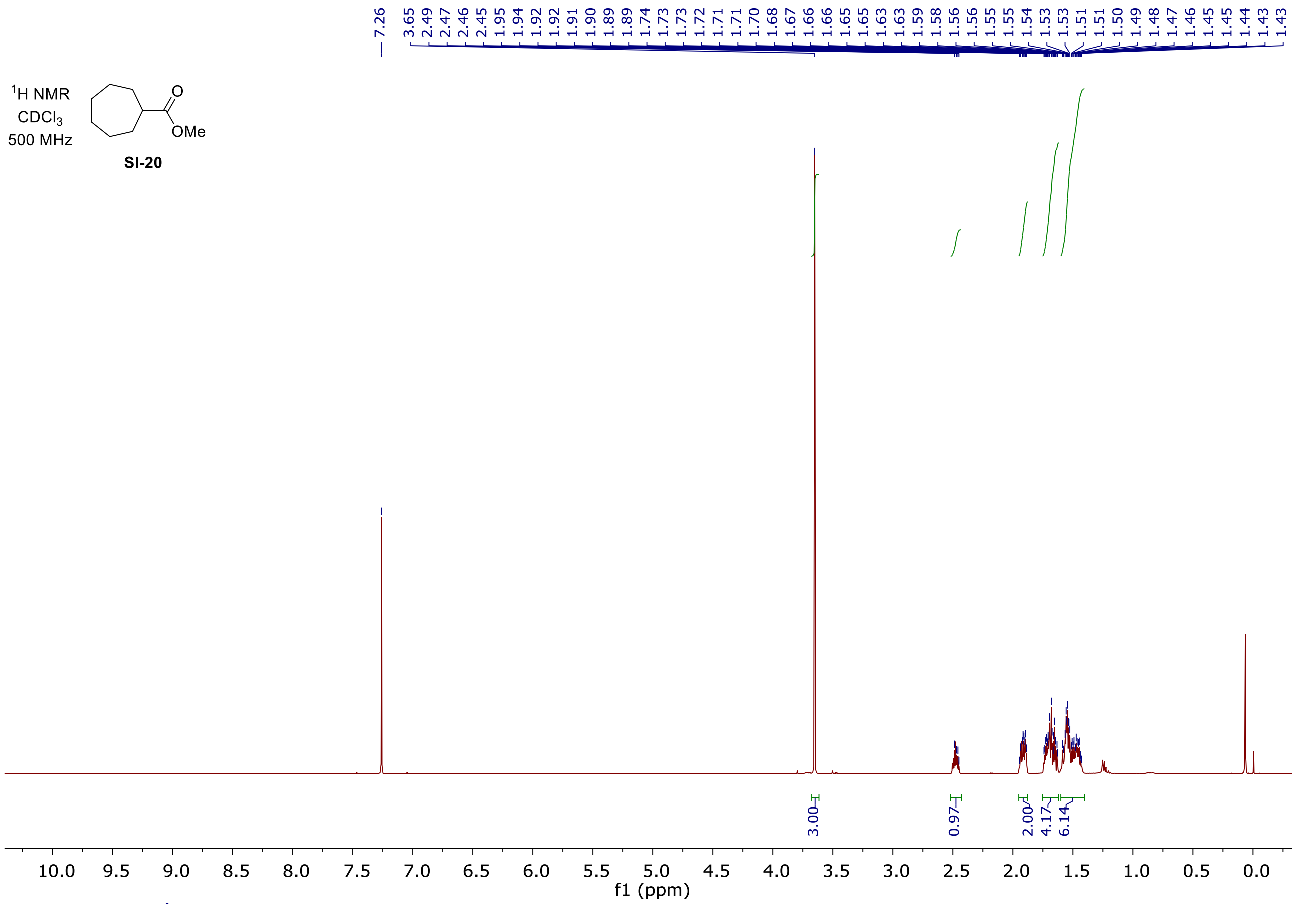

7.26
3.65
2.49
2.47
2.46
2.45
1.95
1.94
1.92
1.92
1.91
1.90
1.89
1.89
1.74
1.73
1.73
1.72
1.71
1.71
1.70
1.68
1.67
1.66
1.66
1.65
1.65
1.63
1.63
1.59
1.58
1.56
1.56
1.55
1.55
1.54
1.53
1.53
1.51
1.51
1.50
1.49
1.48
1.47
1.46
1.45
1.45
1.44
1.43
1.43
1H NMR
CDCl3
500 MHz
O
OMe
SI-20
3.00
0.97
2.00
4.17
6.14
10.0
9.5
9.0
8.5
8.0
7.5
7.0
6.5
6.0
5.5
5.0
4.5
4.0
3.5
3.0
2.5
2.0
1.5
1.0
0.5
0.0
f1 (ppm)

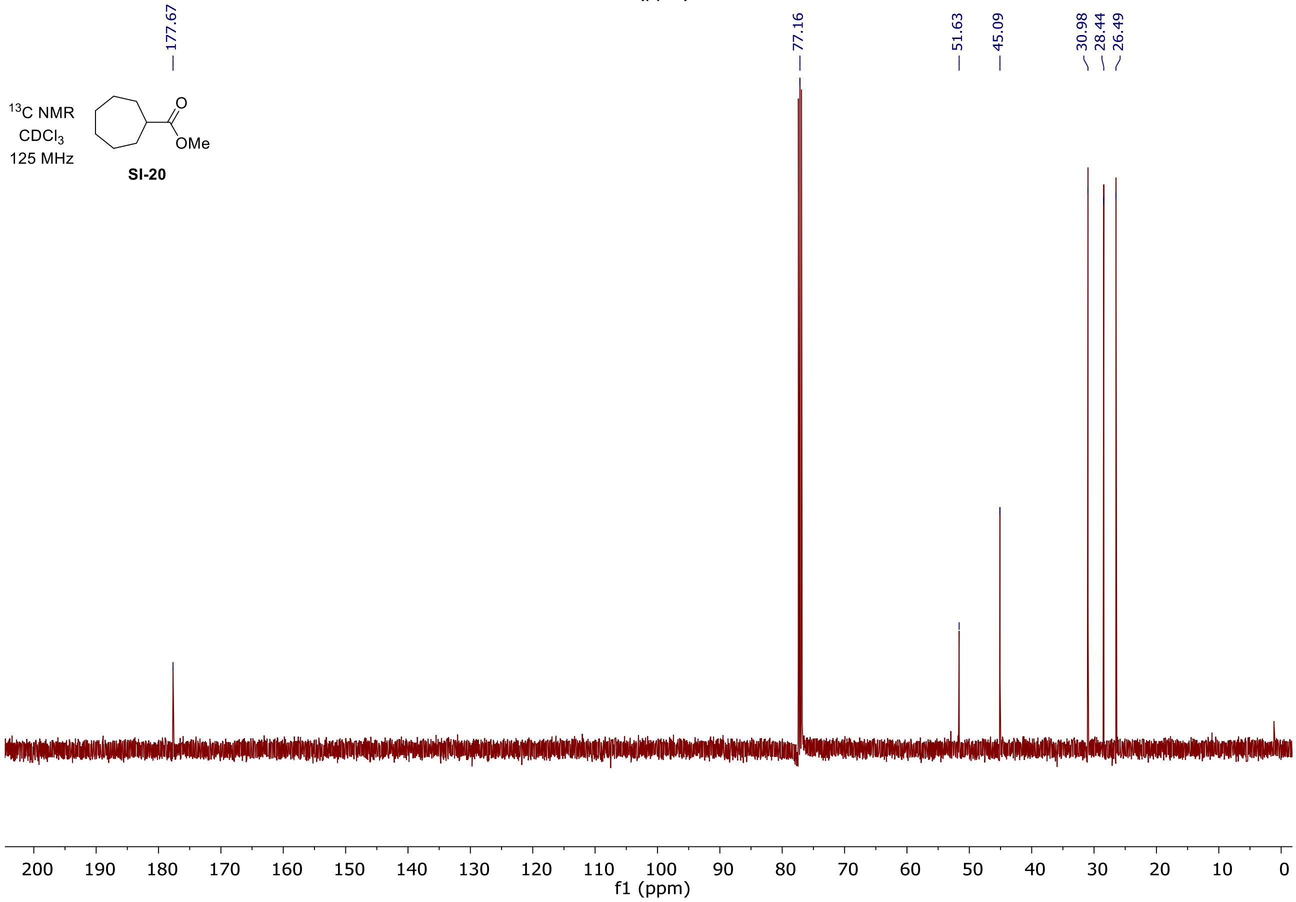

177.67
77.16
51.63
45.09
30.98
28.44
26.49
13C NMR
CDCl3
125 MHz
O
OMe
SI-20
200
190
180
170
160
150
140
130
120
110
100
90
80
70
60
50
40
30
20
10
0
f1 (ppm)

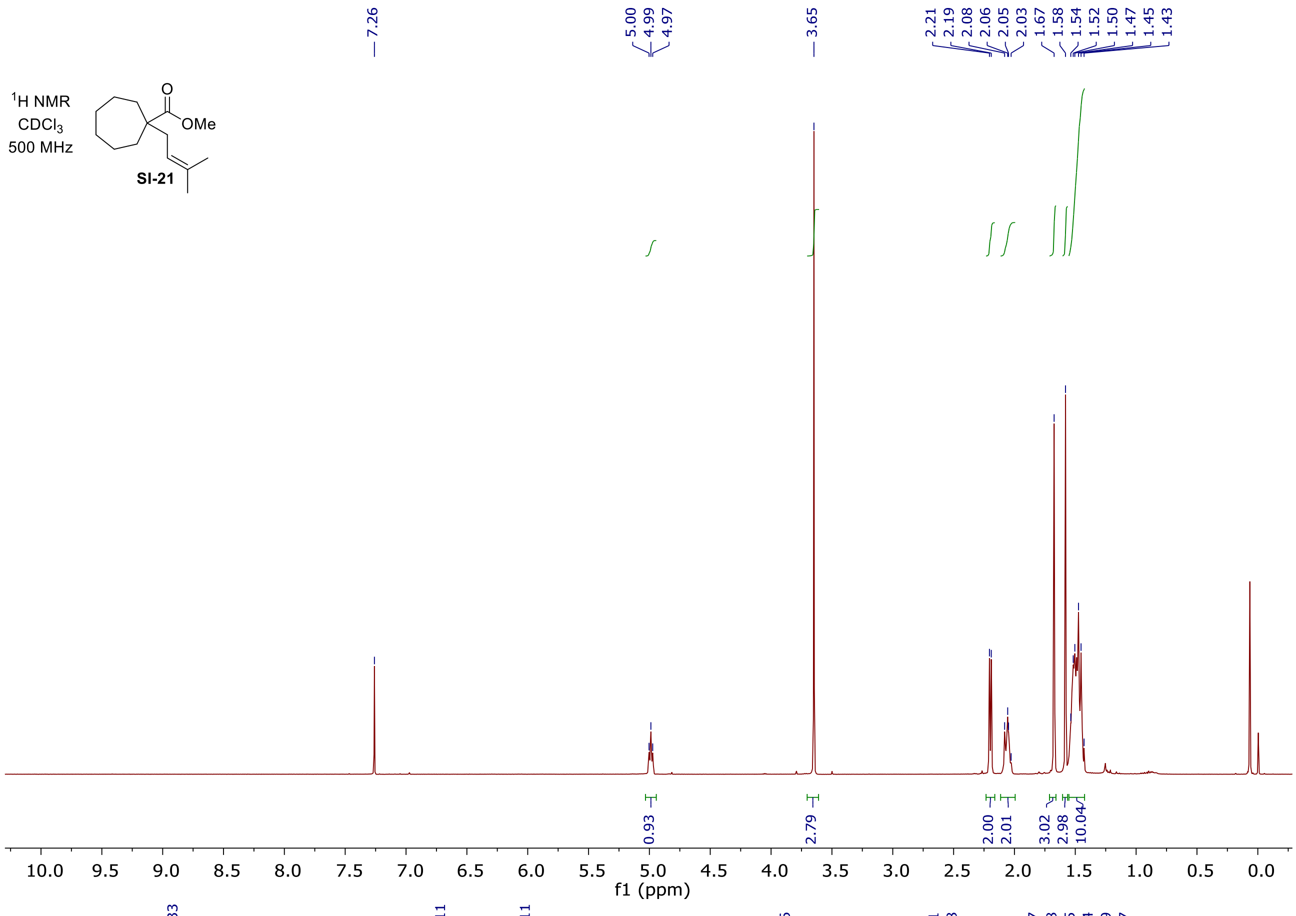
1H NMR
CDCl3
500 MHz
OMe
SI-21
7.26
5.00
4.99
4.97
3.65
2.21
2.19
2.08
2.06
2.05
2.03
1.67
1.58
1.54
1.52
1.50
1.47
1.45
1.43
0.93
2.79
2.00
2.01
3.02
2.98
10.04
f1 (ppm)

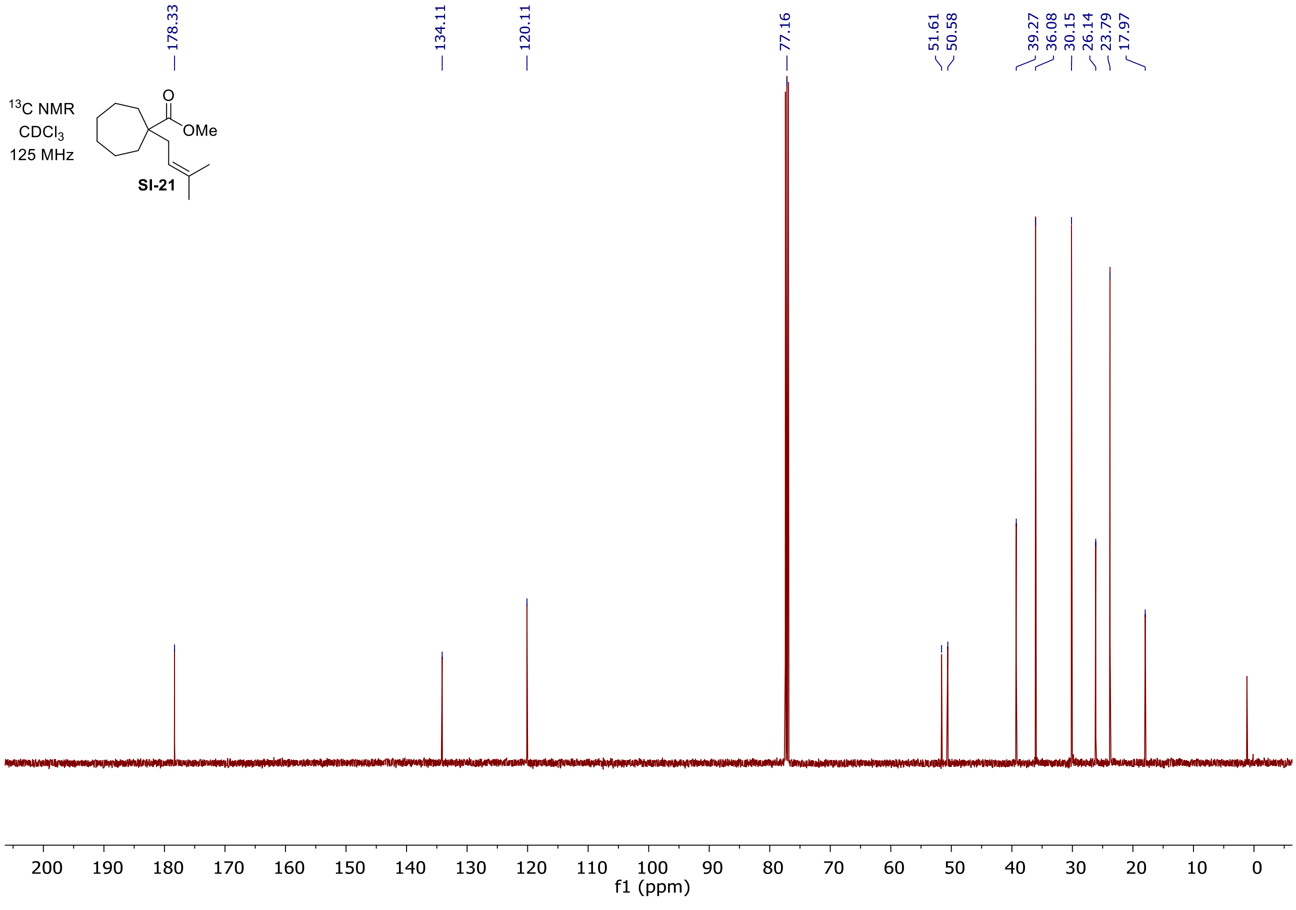
13C NMR
CDCl3
125 MHz
OMe
SI-21
178.33
134.11
120.11
77.16
51.61
50.58
39.27
36.08
30.15
26.14
23.79
17.97
f1 (ppm)

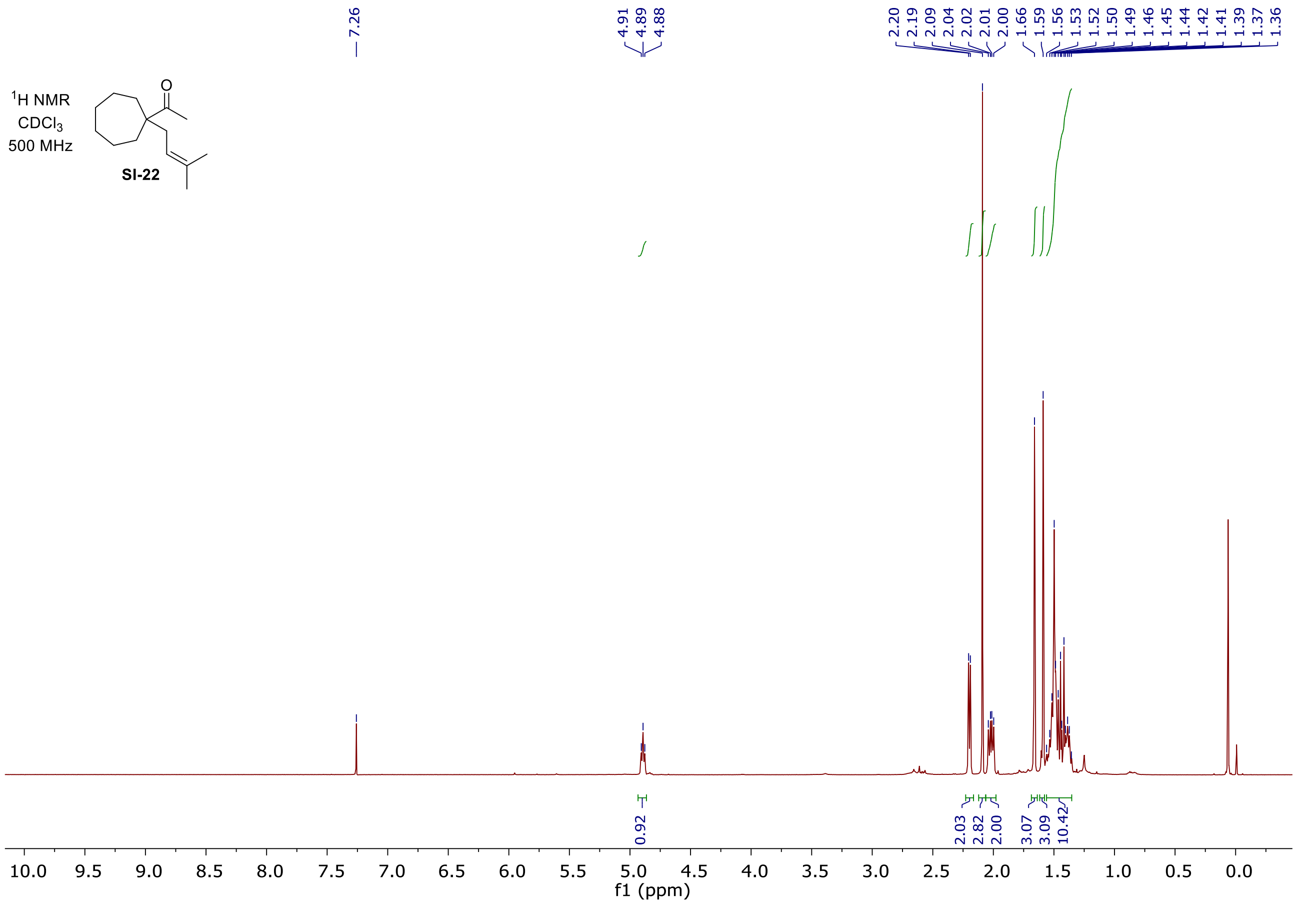

1H NMR
CDCl3
500 MHz
SI-22
7.26
4.91
4.89
4.88
2.20
2.19
2.09
2.04
2.02
2.01
2.00
1.66
1.59
1.56
1.53
1.52
1.50
1.49
1.46
1.45
1.44
1.42
1.41
1.39
1.37
1.36
0.92
2.03
2.82
2.00
3.07
3.09
10.42
f1 (ppm)

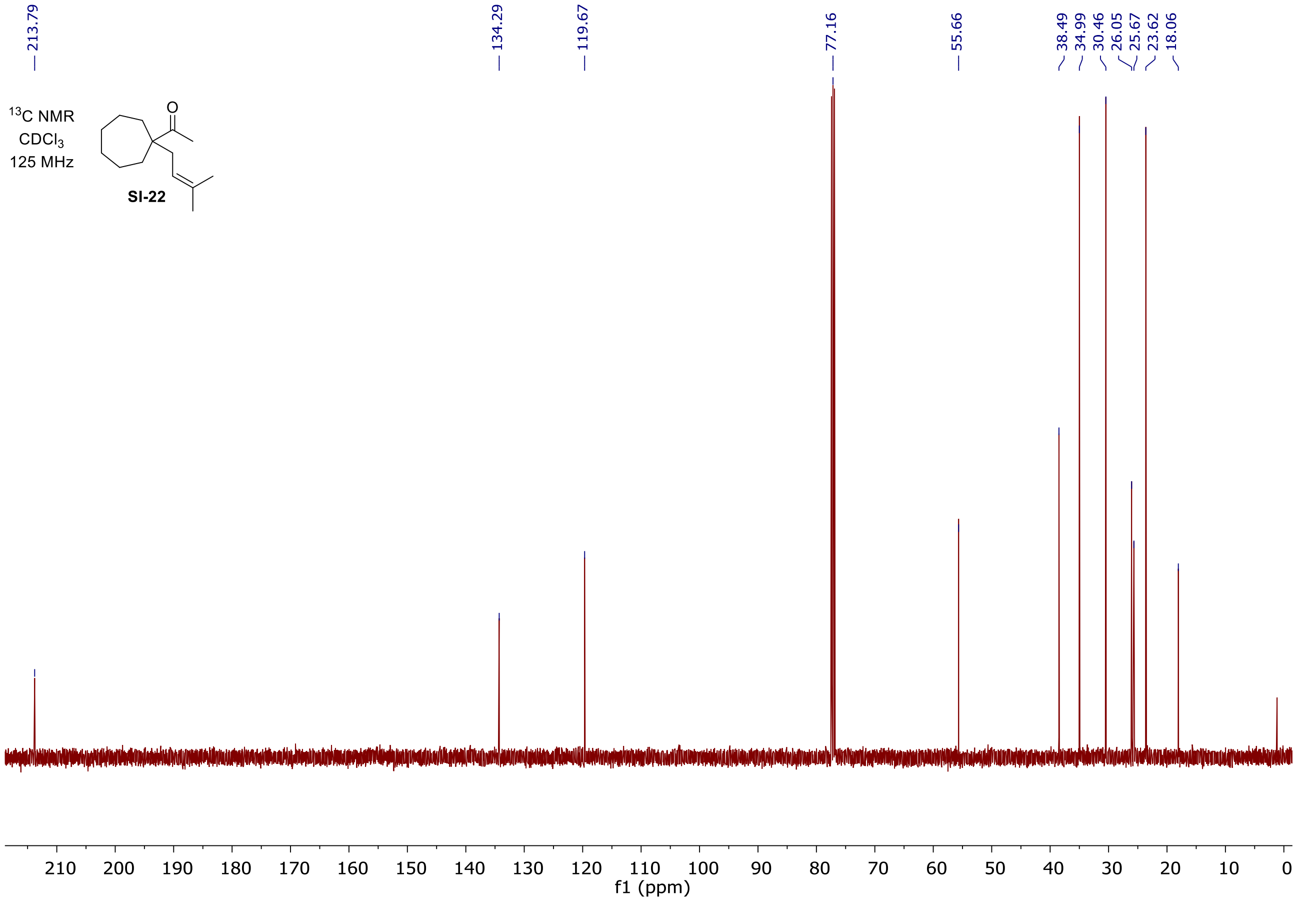

13C NMR
CDCl3
125 MHz
SI-22
213.79
134.29
119.67
77.16
55.66
38.49
34.99
30.46
26.05
25.67
23.62
18.06
f1 (ppm)

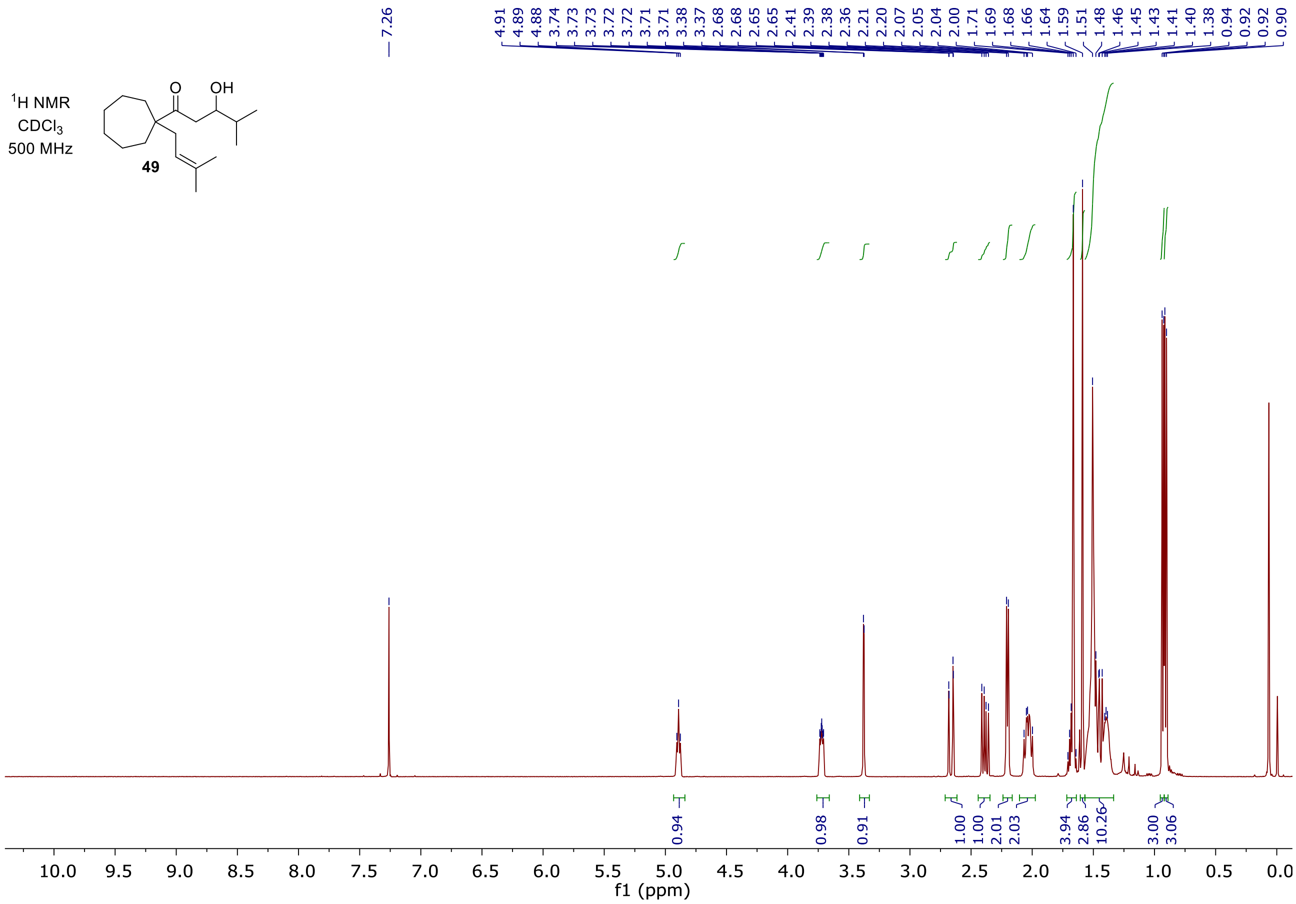

1H NMR
CDCl3
500 MHz
49

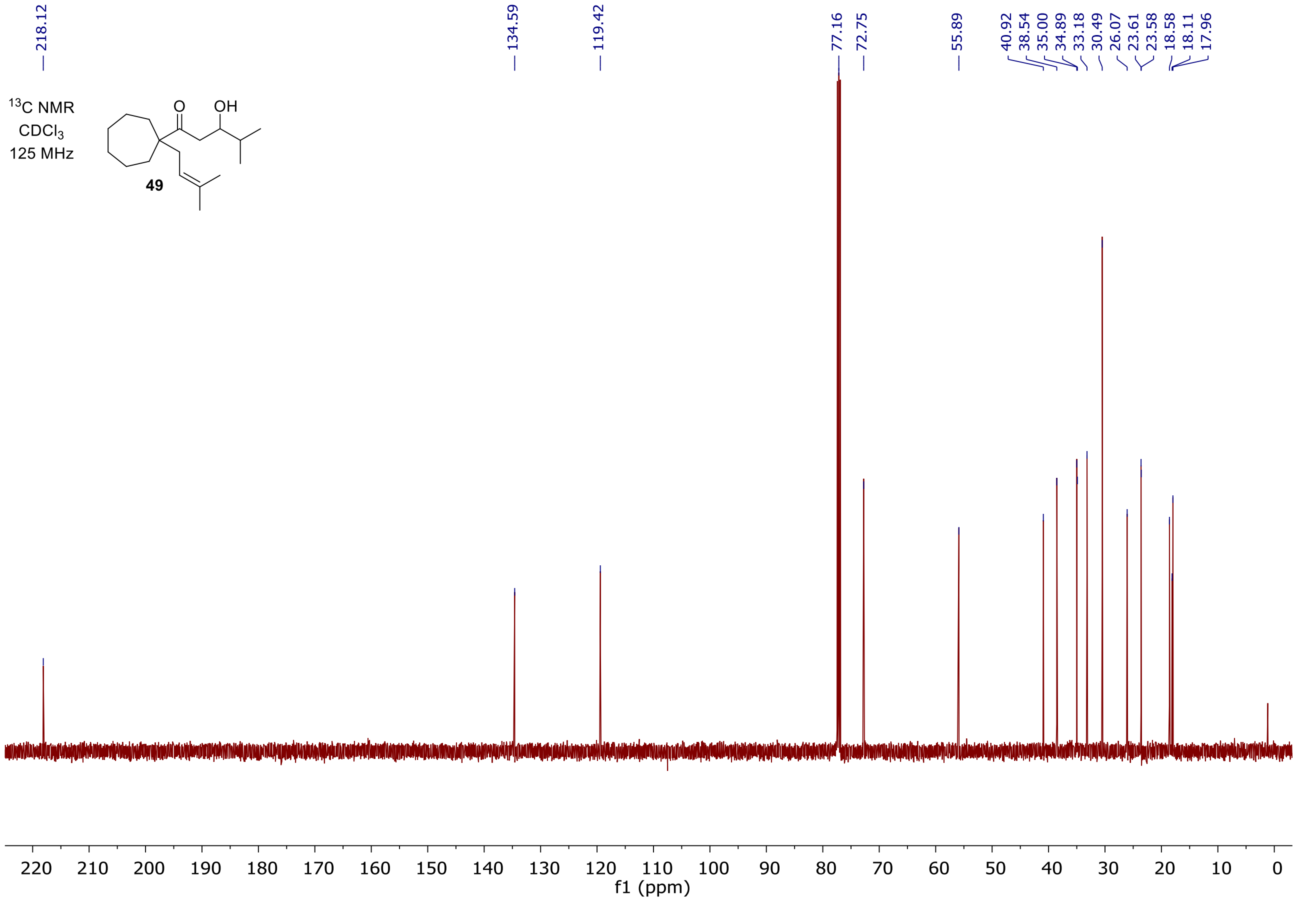

13C NMR
CDCl3
125 MHz
49

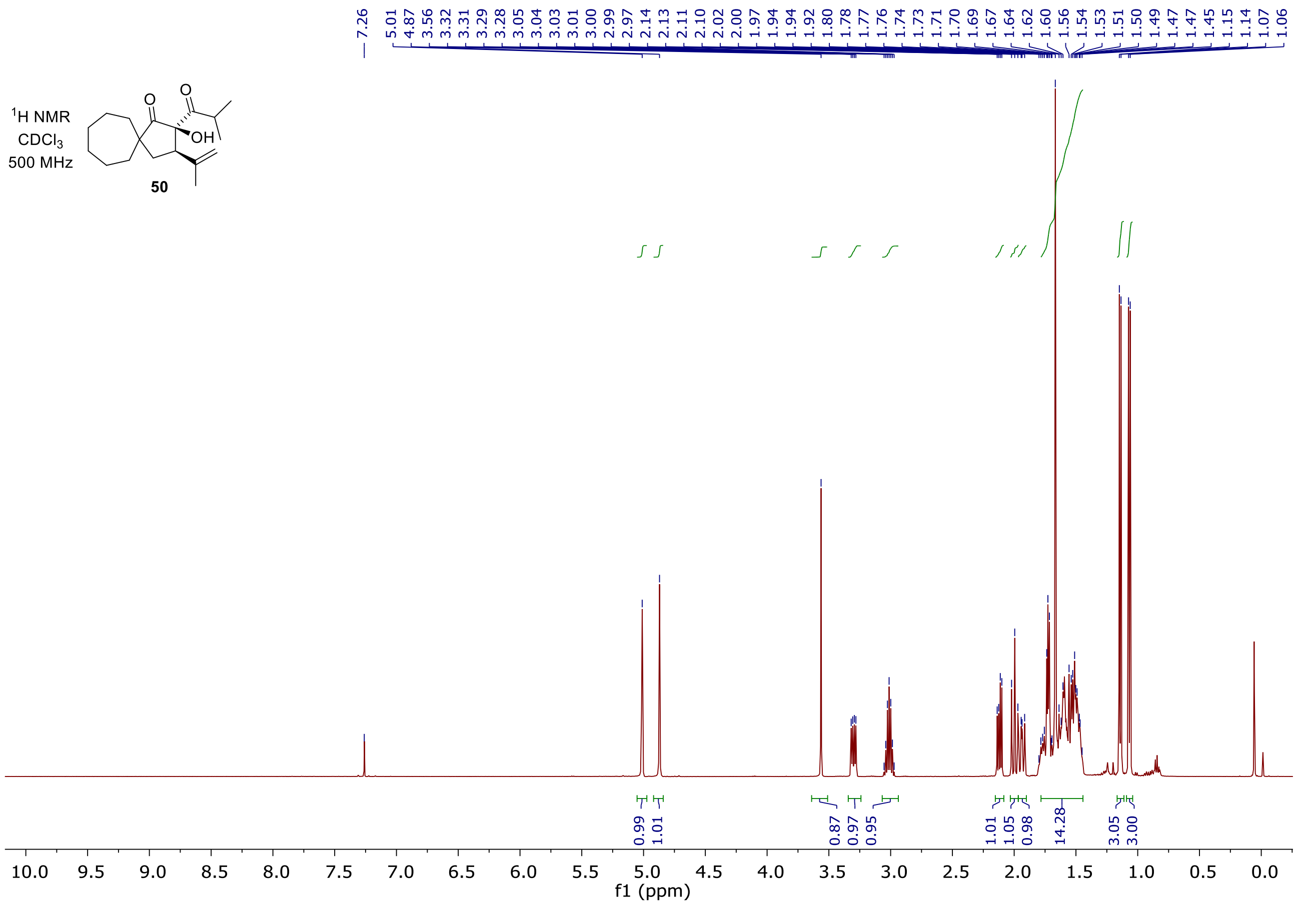

1H NMR
CDCl3
500 MHz
50
OH
7.26
5.01 4.87 3.56 3.32 3.31 3.29 3.28 3.05 3.04 3.03 3.01 3.00 2.99 2.97 2.14 2.13 2.11 2.10 2.02 2.00 1.97 1.94 1.94 1.92 1.80 1.78 1.77 1.76 1.74 1.73 1.71 1.70 1.69 1.67 1.64 1.62 1.60 1.56 1.54 1.53 1.51 1.50 1.49 1.47 1.47 1.45 1.15 1.14 1.07 1.06
0.99 1.01 0.87 0.97 0.95 1.01 1.05 0.98 14.28 3.05 3.00
10.0 9.5 9.0 8.5 8.0 7.5 7.0 6.5 6.0 5.5 5.0 4.5 4.0 3.5 3.0 2.5 2.0 1.5 1.0 0.5 0.0
f1 (ppm)

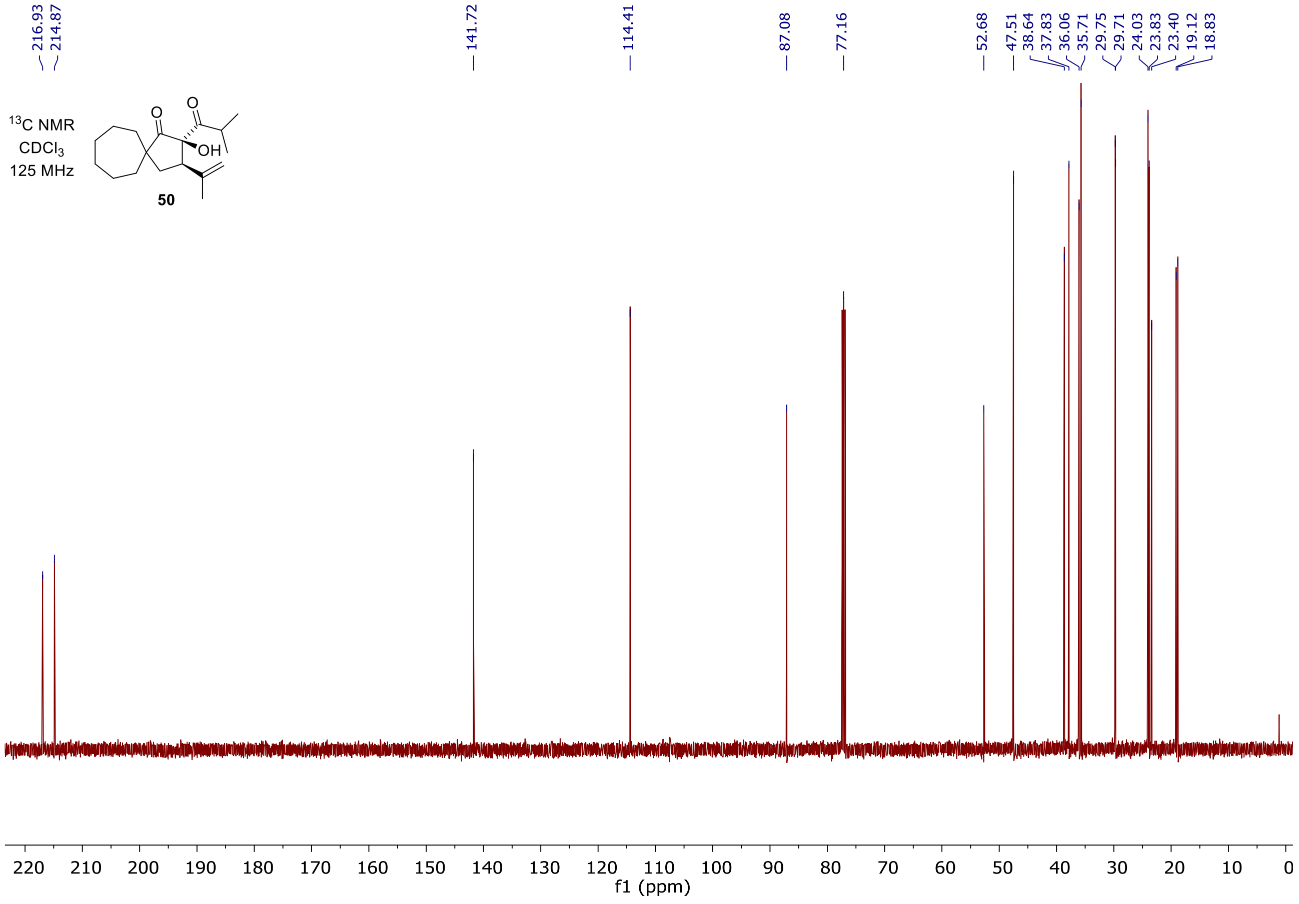

216.93 214.87
141.72
114.41
87.08
77.16
52.68
47.51 38.64 37.83 36.06 35.71 29.75 29.71 24.03 23.83 23.40 19.12 18.83
13C NMR
CDCl3
125 MHz
50
OH
220 210 200 190 180 170 160 150 140 130 120 110 100 90 80 70 60 50 40 30 20 10 0
f1 (ppm)

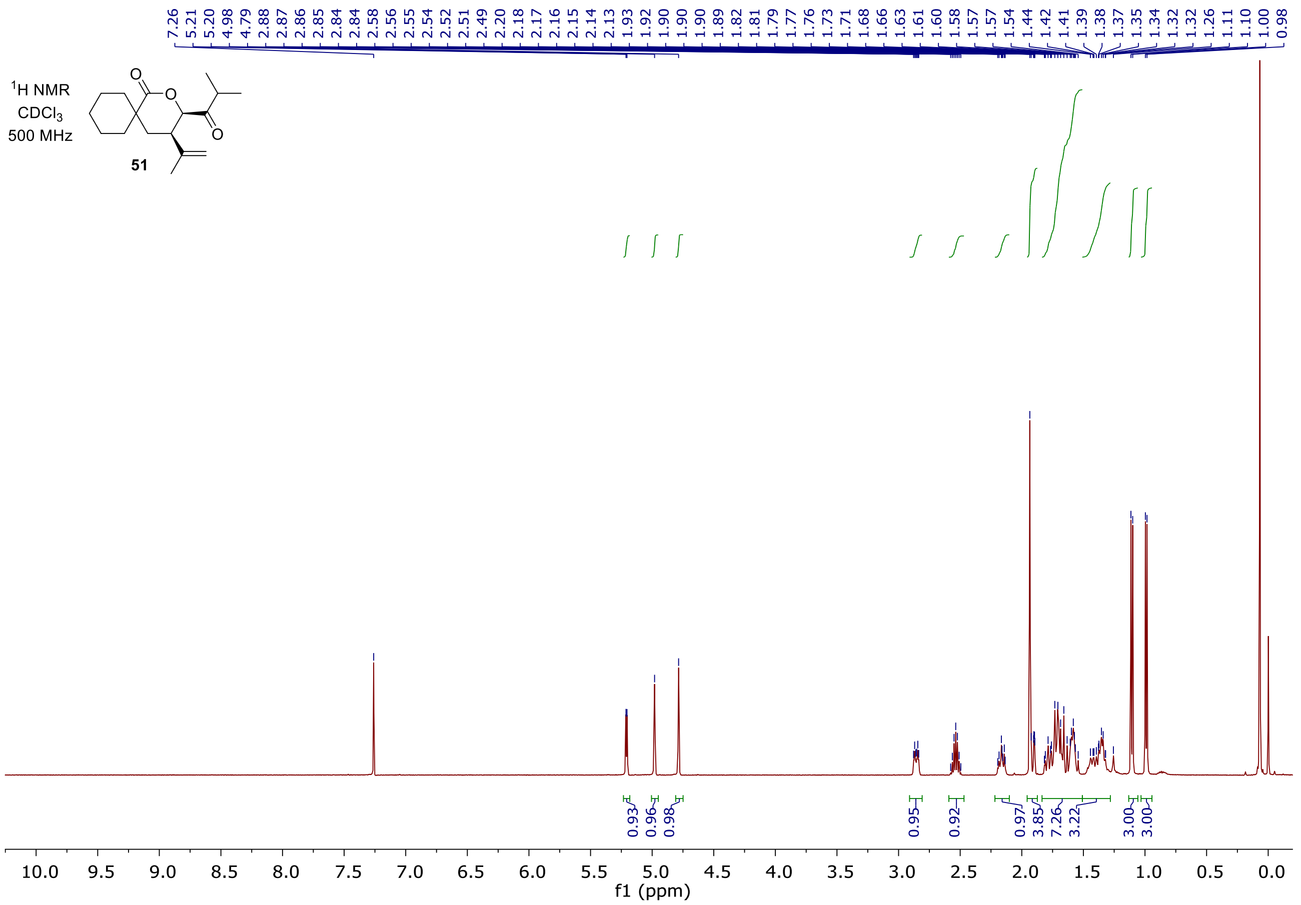
^{1}H NMR
$CDCl_3$
500 MHz
51
f1 (ppm)

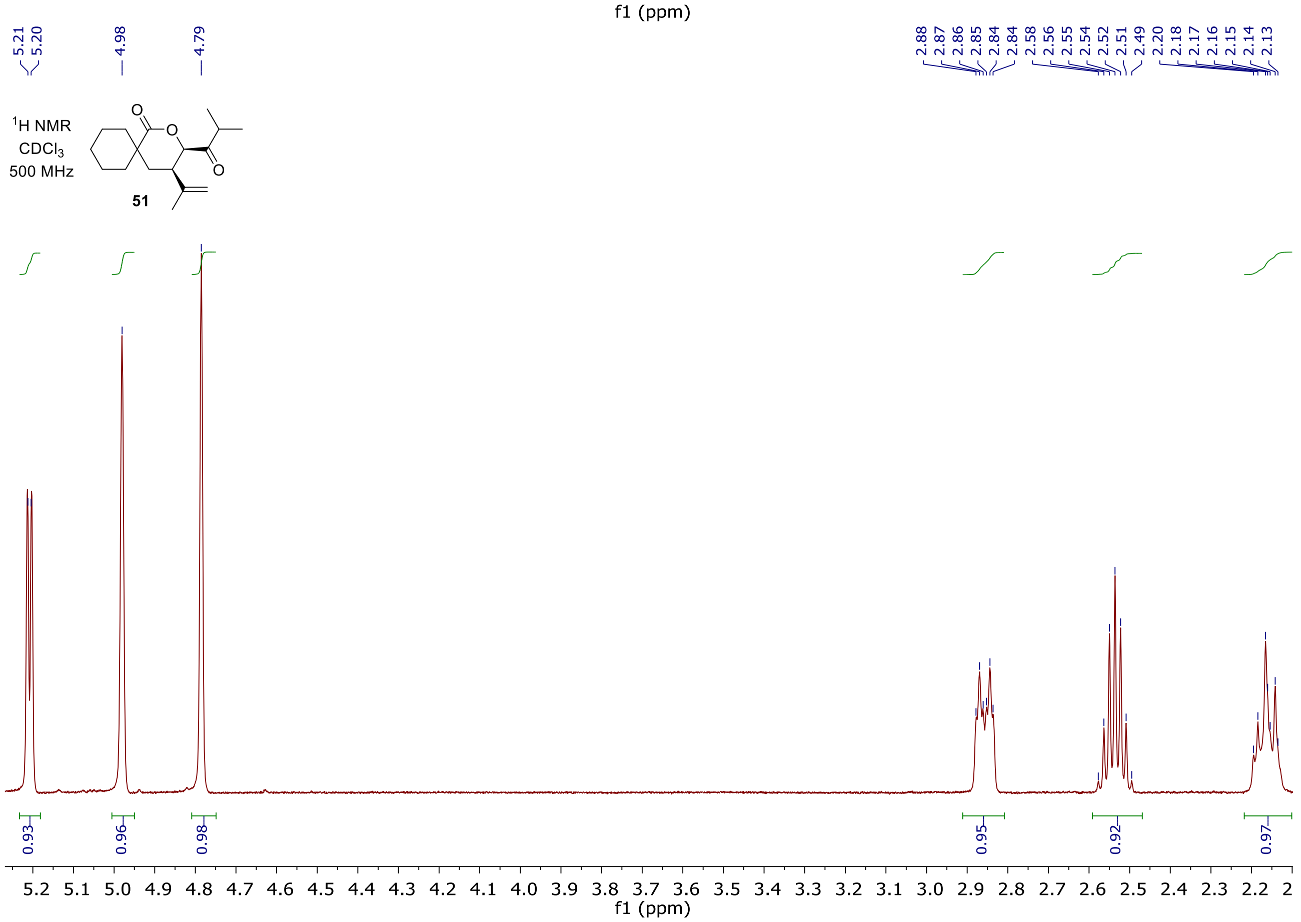
^{1}H NMR
$CDCl_3$
500 MHz
51
f1 (ppm)

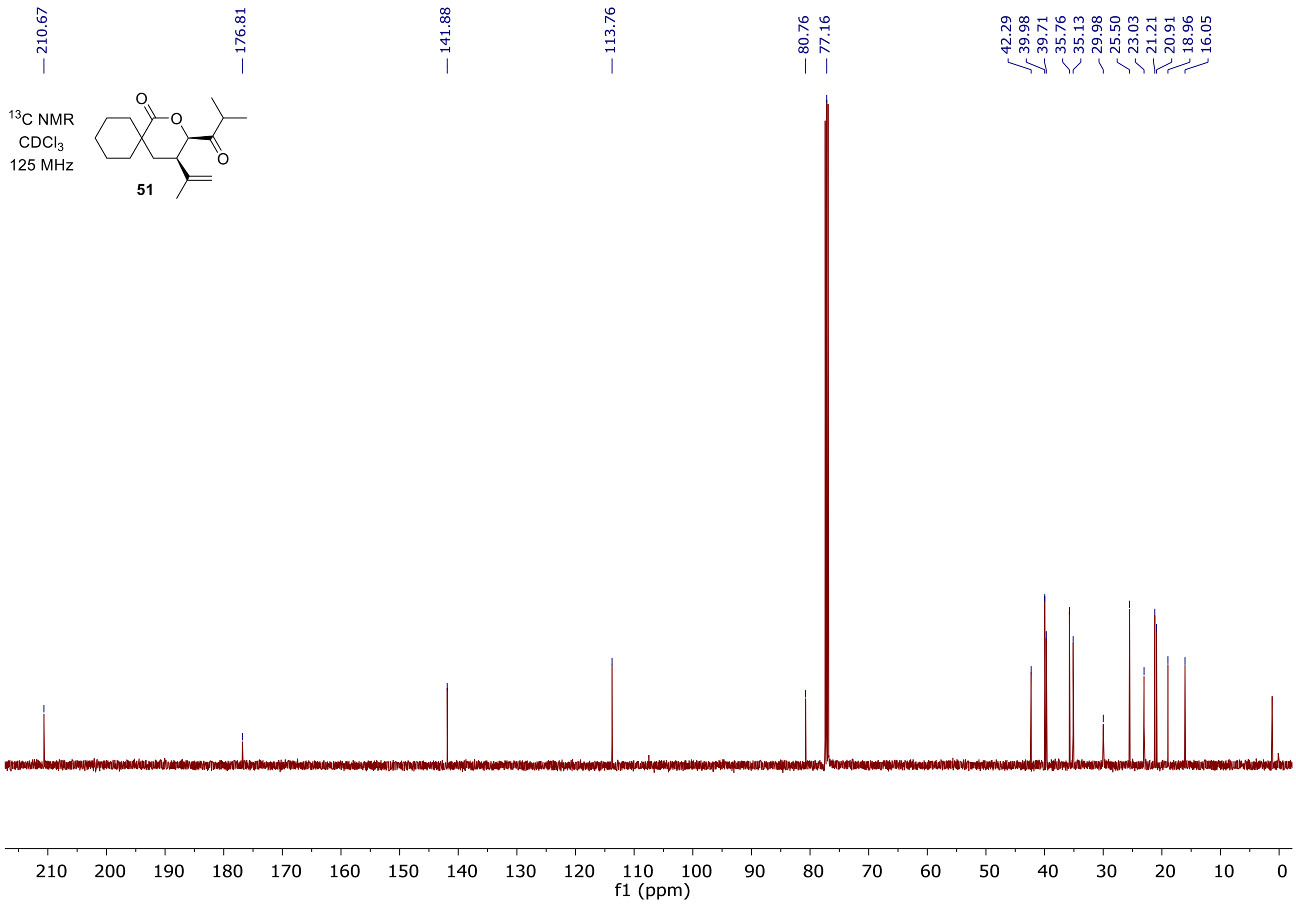
210.67
176.81
141.88
113.76
80.76
77.16
42.29
39.98
39.71
35.76
35.13
29.98
25.50
23.03
21.21
20.91
18.96
16.05
13C NMR
CDCl3
125 MHz
51
210 200 190 180 170 160 150 140 130 120 110 100 90 80 70 60 50 40 30 20 10 0
f1 (ppm)

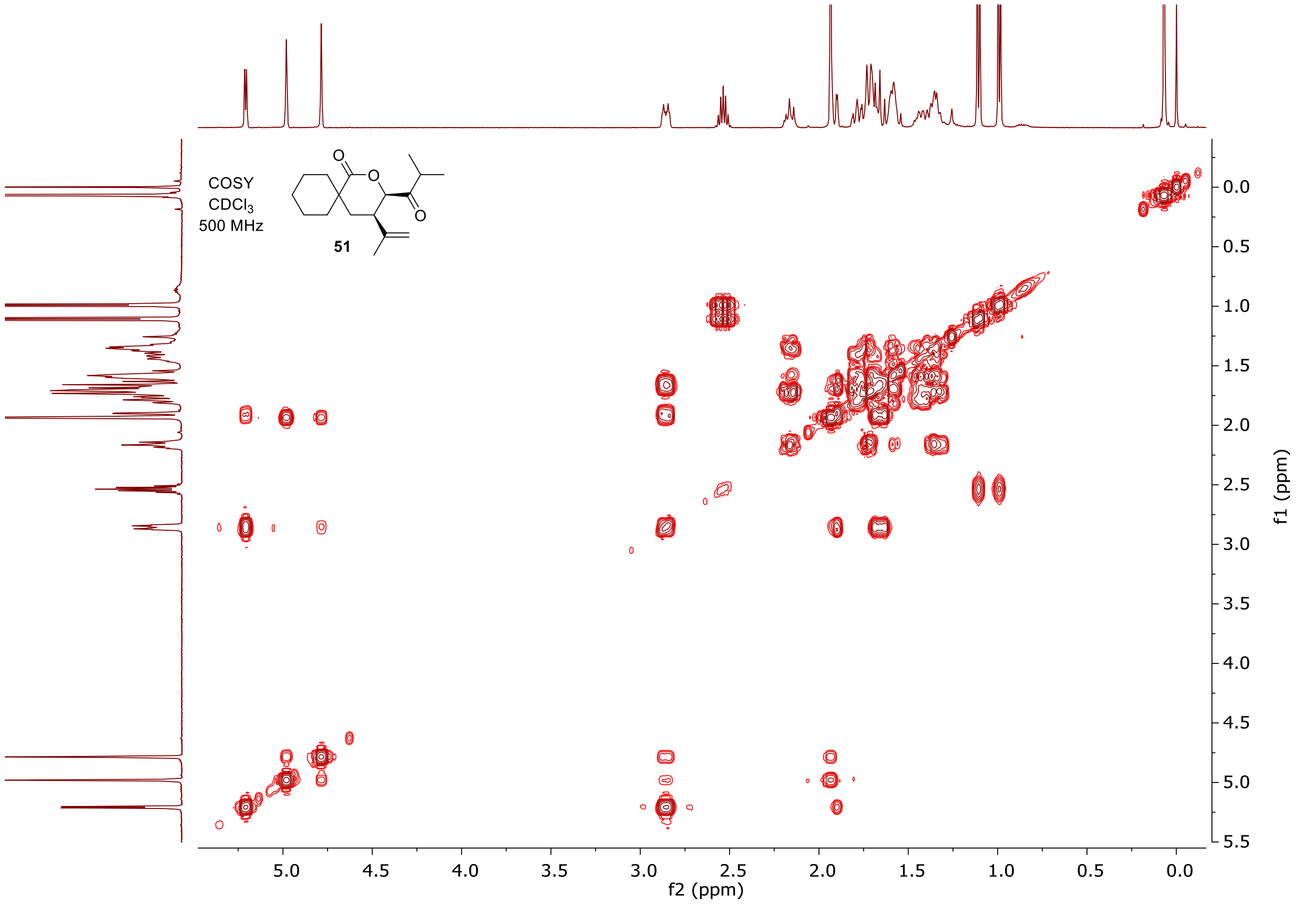
COSY
CDCl3
500 MHz
51
5.0 4.5 4.0 3.5 3.0 2.5 2.0 1.5 1.0 0.5 0.0
f2 (ppm)
f1 (ppm)

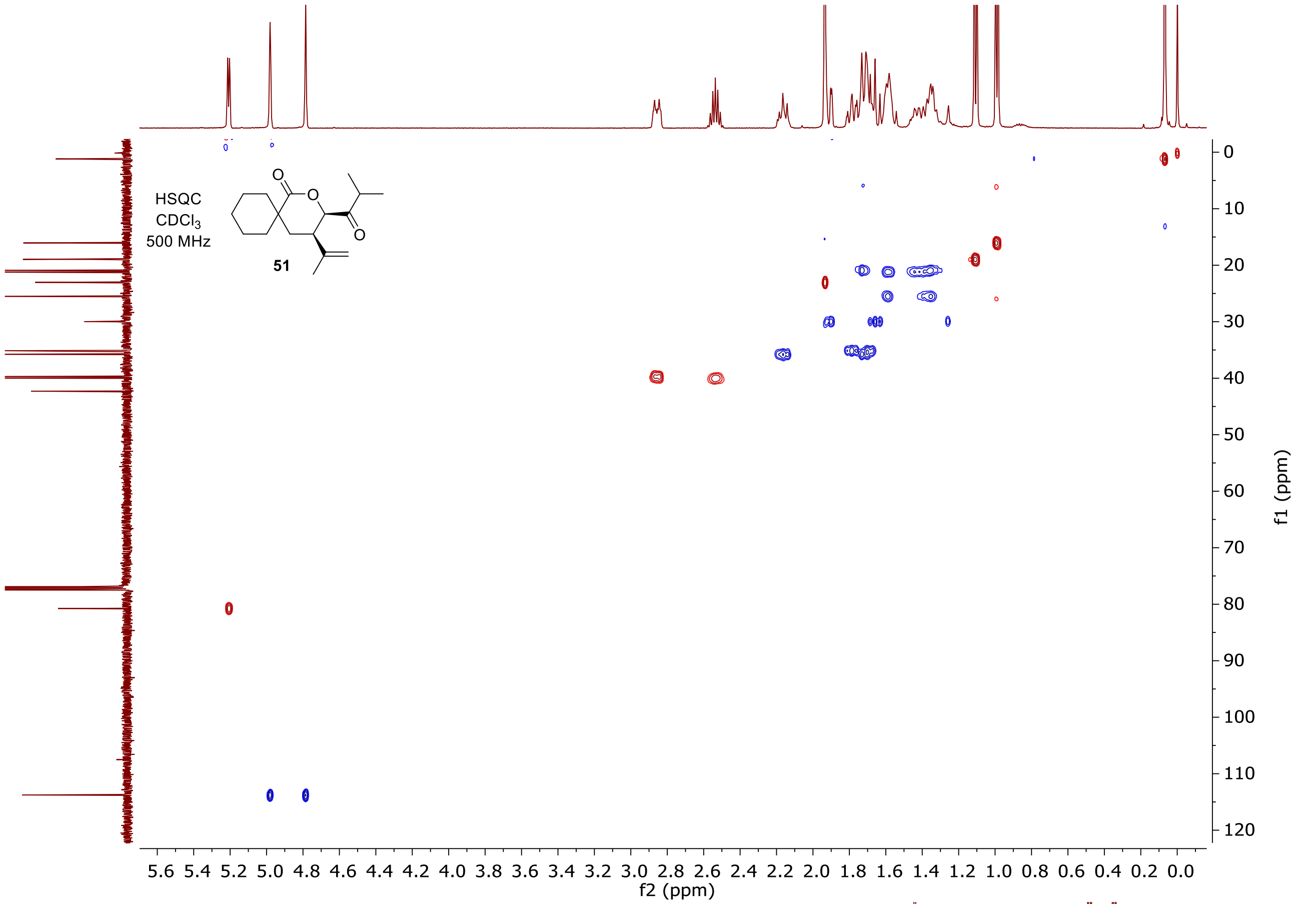
HSQC
CDCl3
500 MHz
51
f2 (ppm)
f1 (ppm)

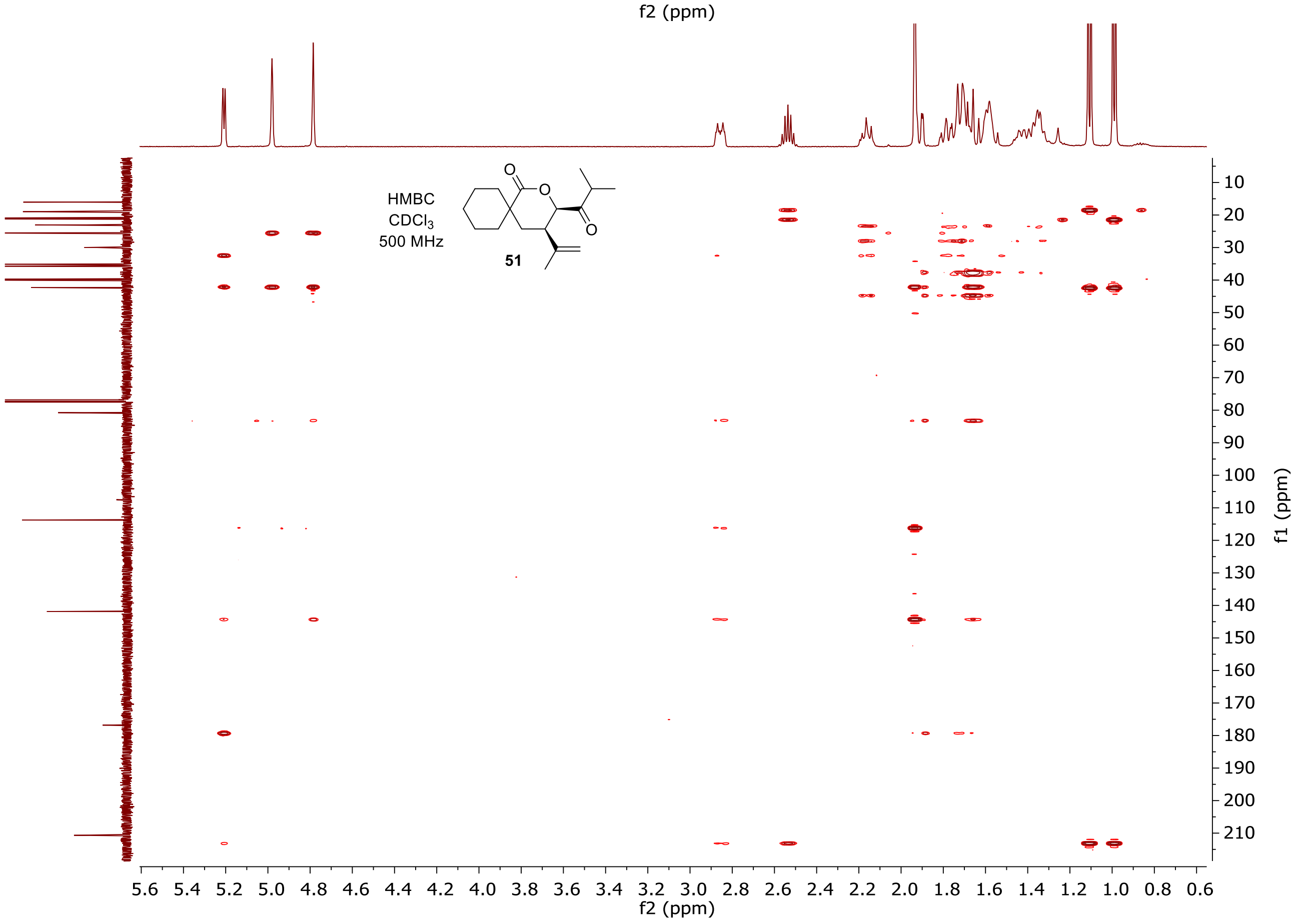
HMBC
CDCl3
500 MHz
51
f2 (ppm)
f1 (ppm)

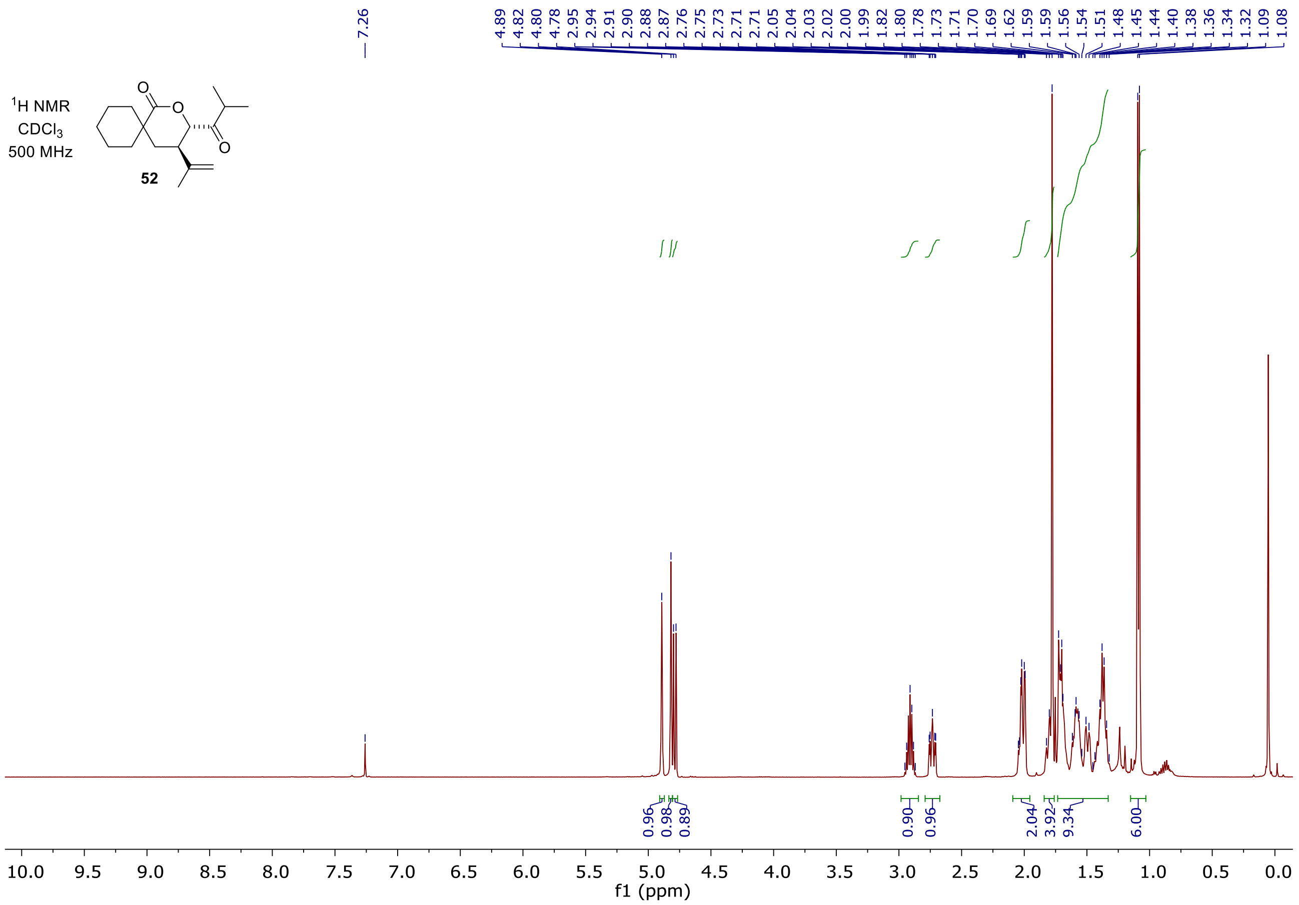

^{1}H NMR
CDCl$_3$
500 MHz
52
f1 (ppm)

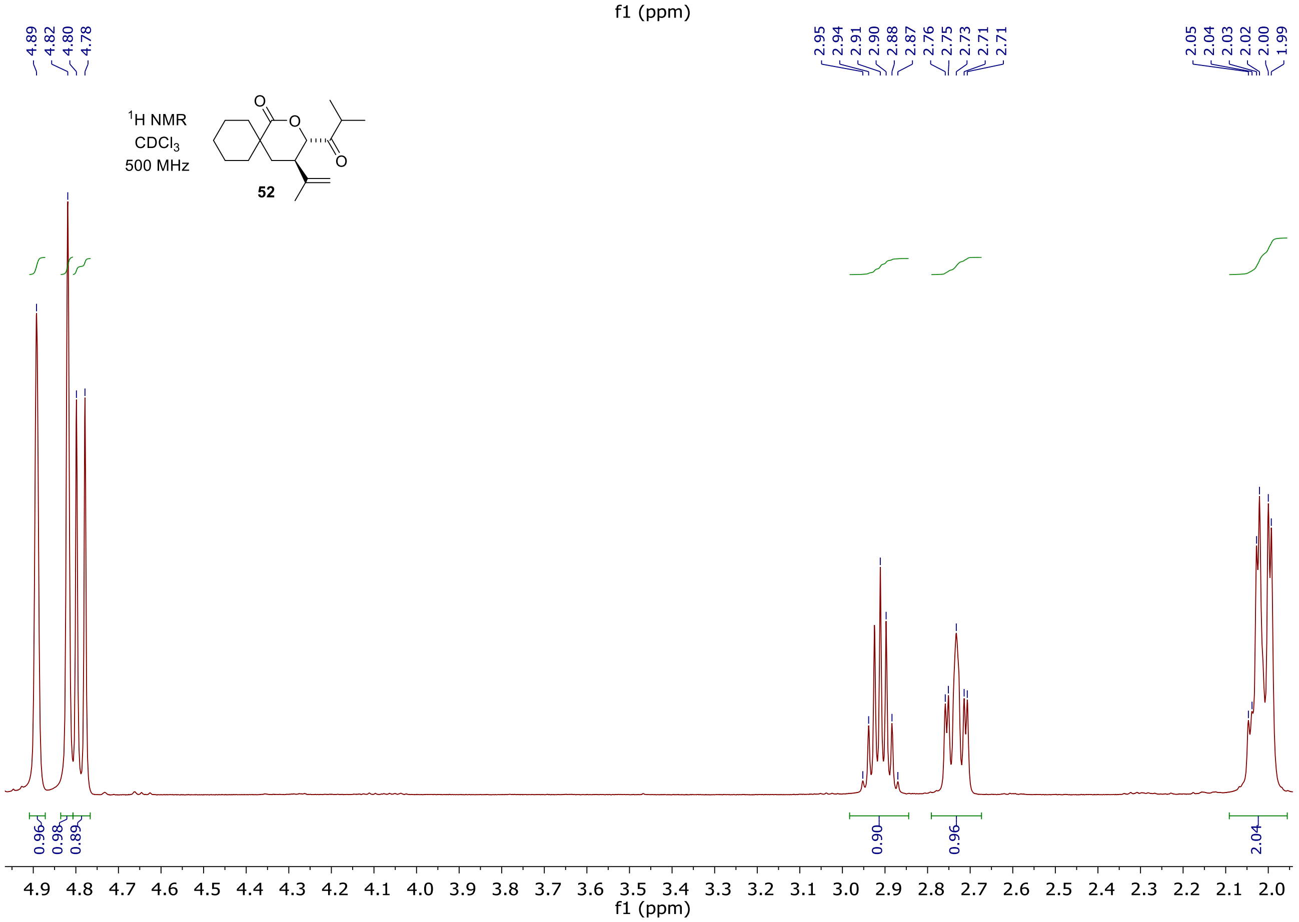

^{1}H NMR
CDCl$_3$
500 MHz
52
f1 (ppm)

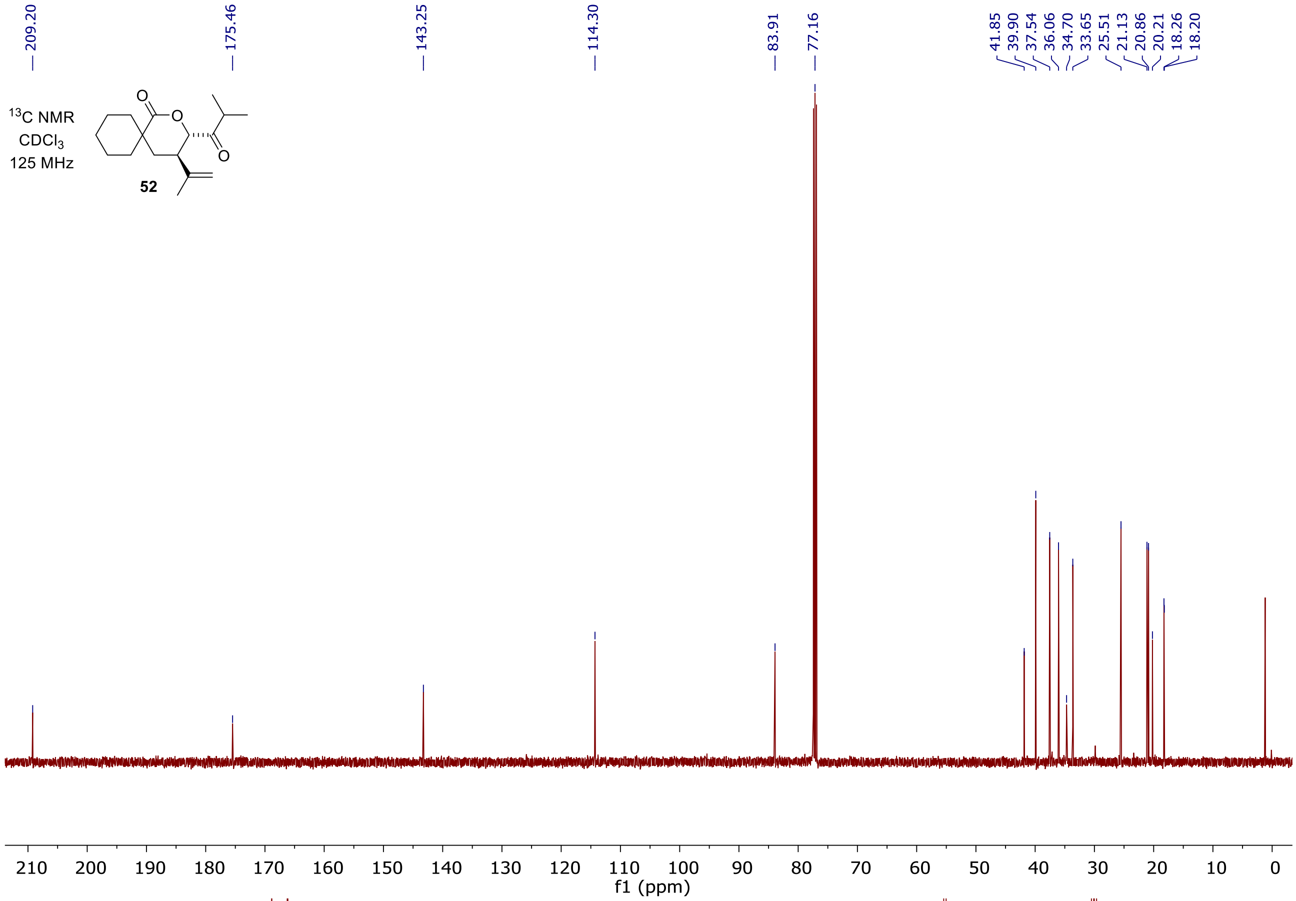
209.20
175.46
143.25
114.30
83.91
77.16
41.85
39.90
37.54
36.06
34.70
33.65
25.51
21.13
20.86
20.21
18.26
18.20
13C NMR
CDCl3
125 MHz
52
f1 (ppm)

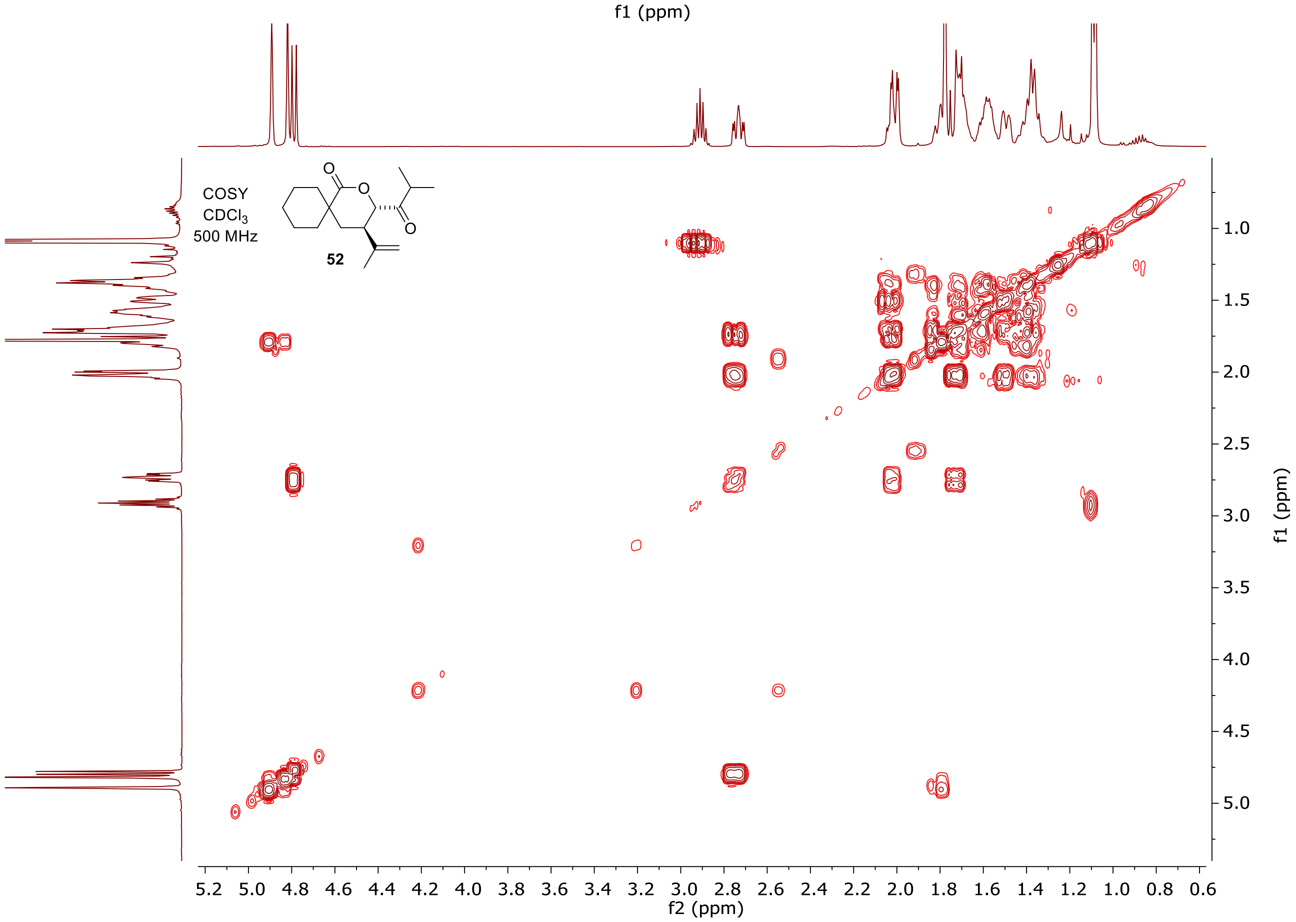
COSY
CDCl3
500 MHz
52
f2 (ppm)
f1 (ppm)

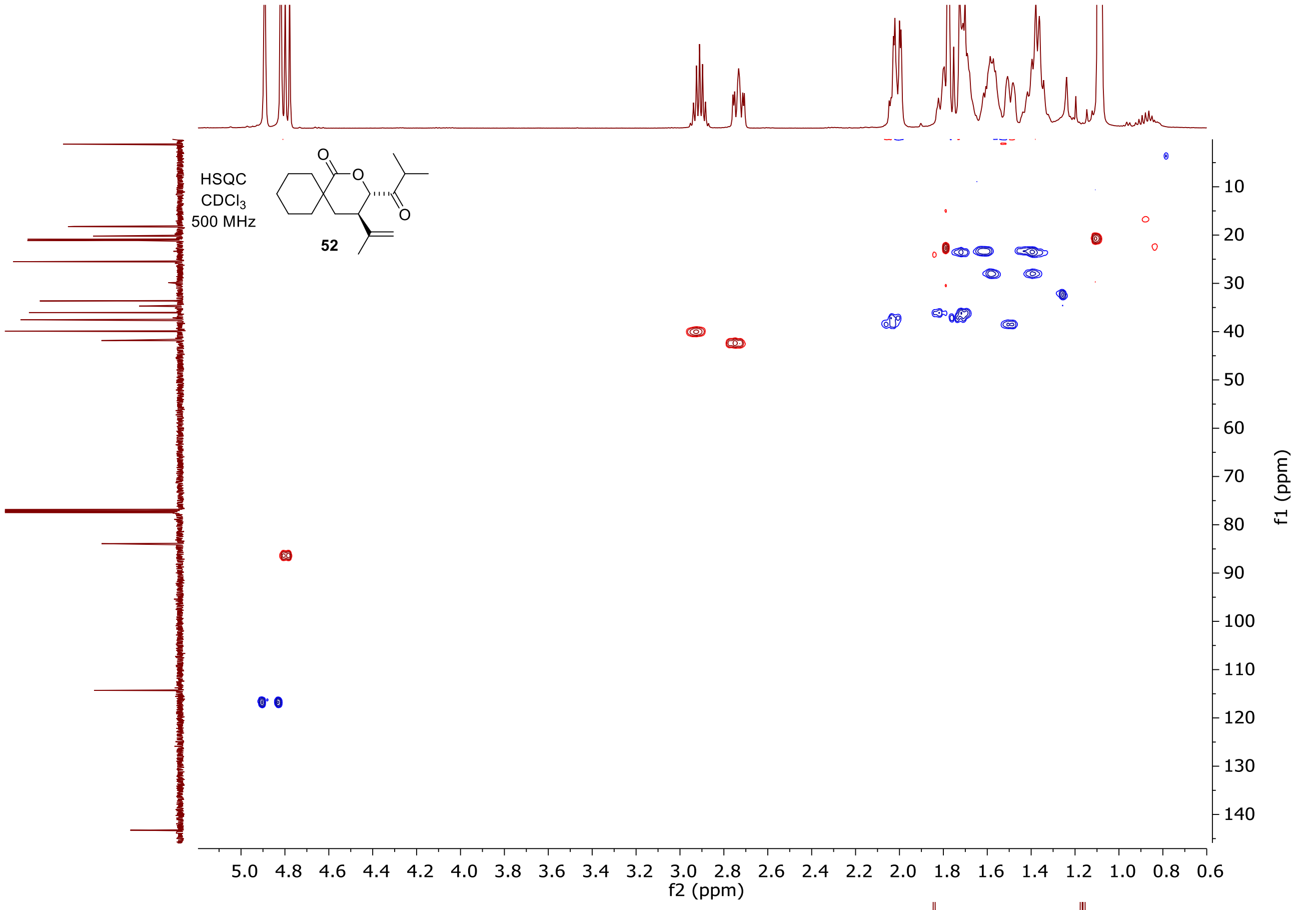
HSQC
CDCl3
500 MHz
52
f2 (ppm)
f1 (ppm)

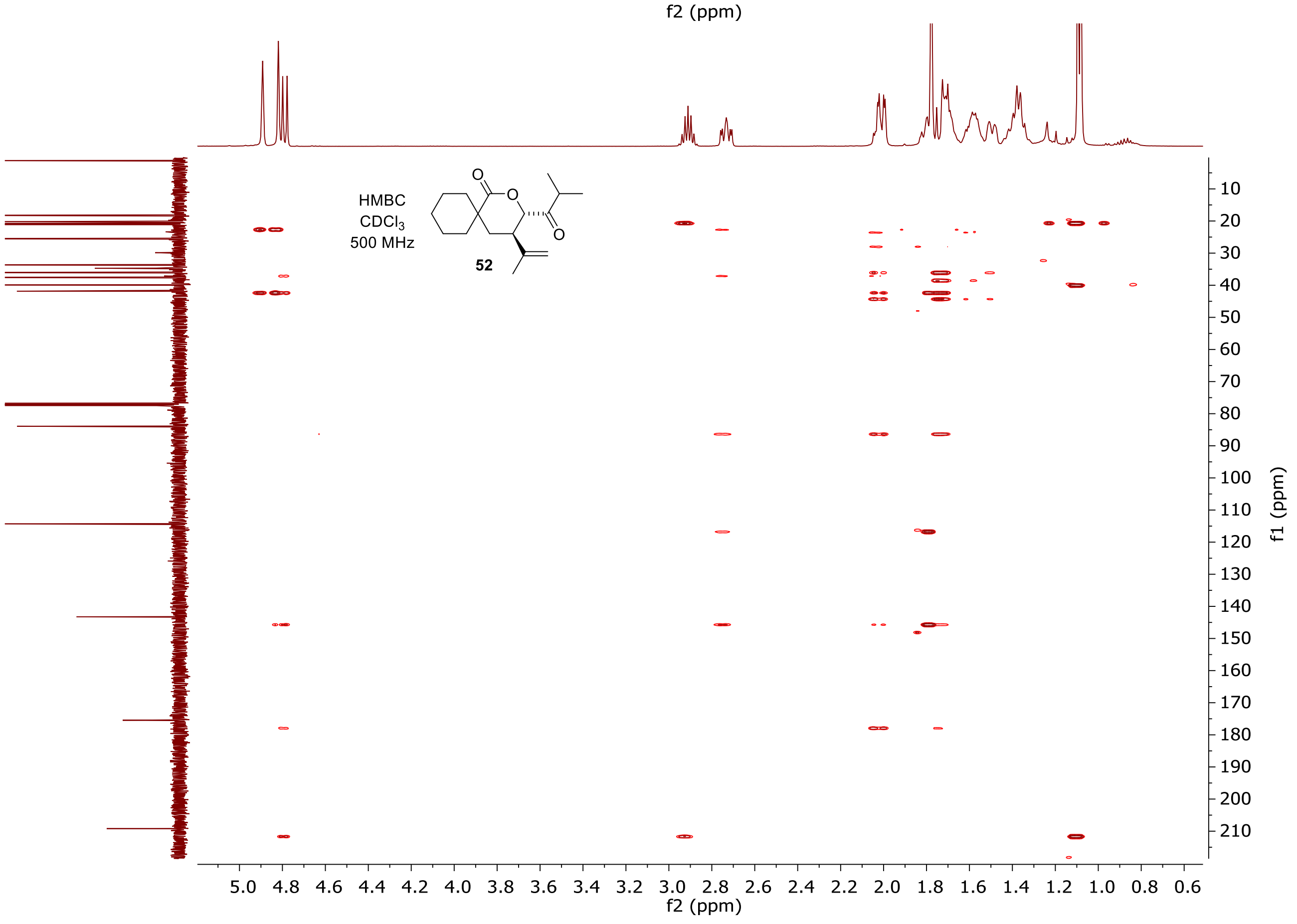
HMBC
CDCl3
500 MHz
52
f2 (ppm)
f1 (ppm)

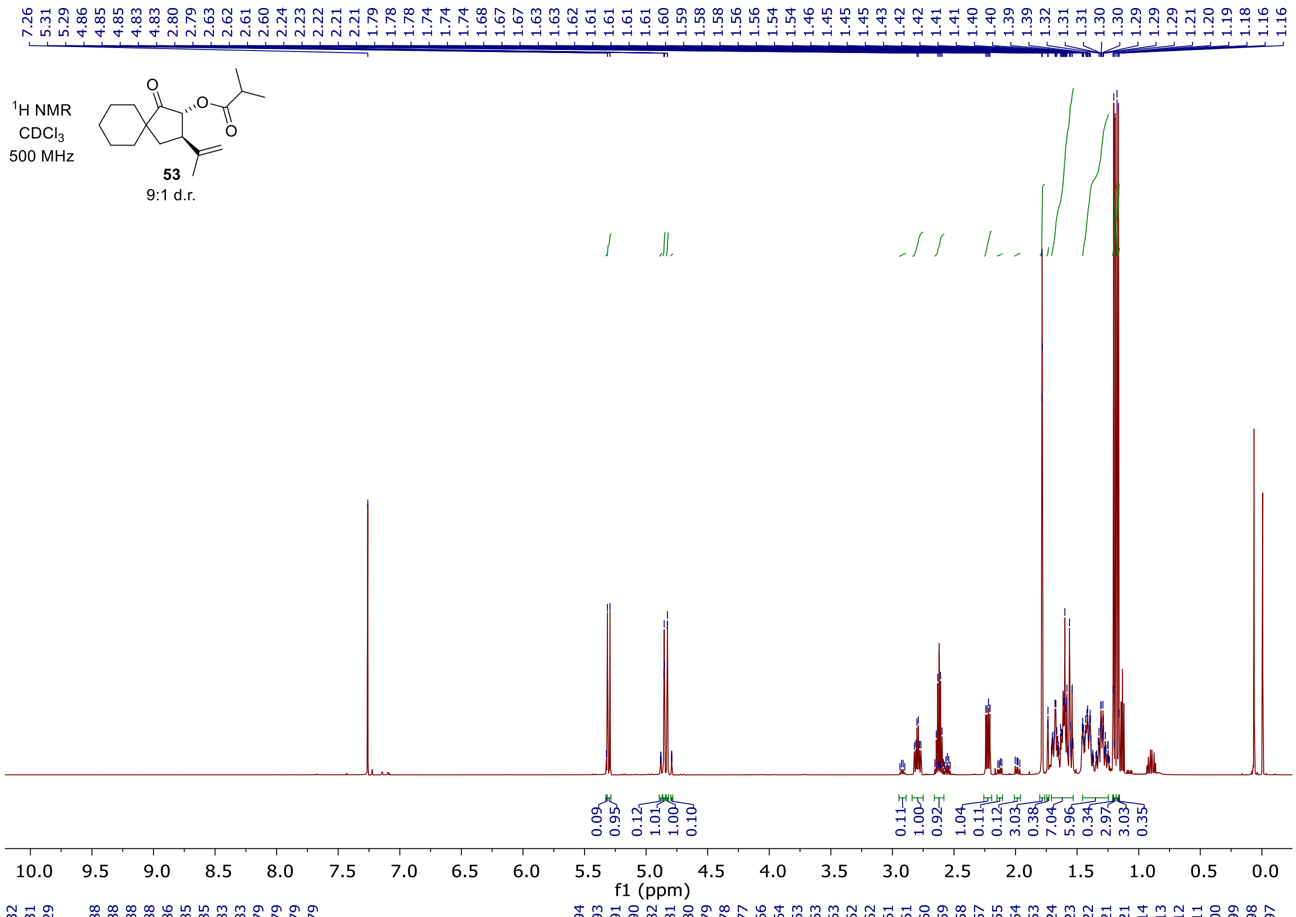

7.26 5.31 5.29 4.86 4.85 4.83 4.83 2.80 2.79 2.63 2.62 2.61 2.60 2.24 2.23 2.22 2.21 2.21 1.79 1.78 1.78 1.74 1.74 1.68 1.67 1.67 1.63 1.63 1.62 1.61 1.61 1.61 1.61 1.60 1.59 1.58 1.58 1.56 1.56 1.54 1.54 1.46 1.45 1.45 1.45 1.43 1.42 1.42 1.41 1.41 1.40 1.40 1.39 1.39 1.32 1.31 1.31 1.30 1.30 1.29 1.29 1.21 1.20 1.19 1.18 1.16 1.16
1H NMR
CDCl3
500 MHz
53
9:1 d.r.
0.09 0.95 0.12 1.01 1.00 0.10 0.11 1.00 0.92 1.04 0.11 0.12 3.03 0.38 7.04 5.96 0.34 2.97 3.03 0.35
10.0 9.5 9.0 8.5 8.0 7.5 7.0 6.5 6.0 5.5 5.0 4.5 4.0 3.5 3.0 2.5 2.0 1.5 1.0 0.5 0.0
f1 (ppm)

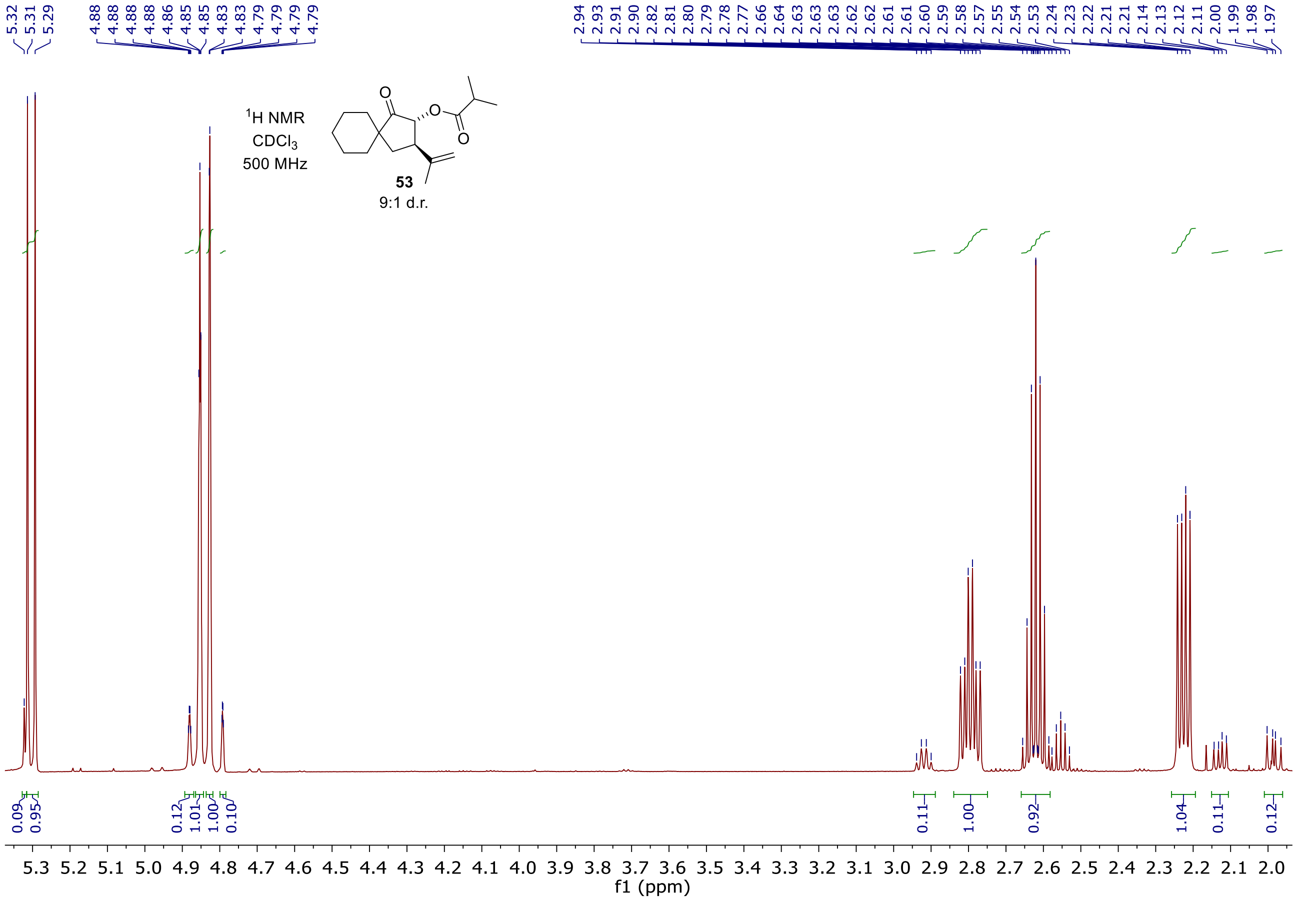

5.32 5.31 5.29
4.88 4.88 4.88 4.88 4.86 4.85 4.85 4.83 4.83 4.79 4.79 4.79 4.79
2.94 2.93 2.91 2.90 2.82 2.81 2.80 2.79 2.78 2.77 2.66 2.64 2.63 2.63 2.62 2.62 2.61 2.60 2.59 2.58 2.57 2.55 2.54 2.53 2.24 2.23 2.22 2.21 2.21 2.14 2.13 2.12 2.11 2.00 1.99 1.98 1.97
1H NMR
CDCl3
500 MHz
53
9:1 d.r.
0.09 0.95 0.12 1.01 1.00 0.10 0.11 1.00 0.92 1.04 0.11 0.12
5.3 5.2 5.1 5.0 4.9 4.8 4.7 4.6 4.5 4.4 4.3 4.2 4.1 4.0 3.9 3.8 3.7 3.6 3.5 3.4 3.3 3.2 3.1 3.0 2.9 2.8 2.7 2.6 2.5 2.4 2.3 2.2 2.1 2.0
f1 (ppm)

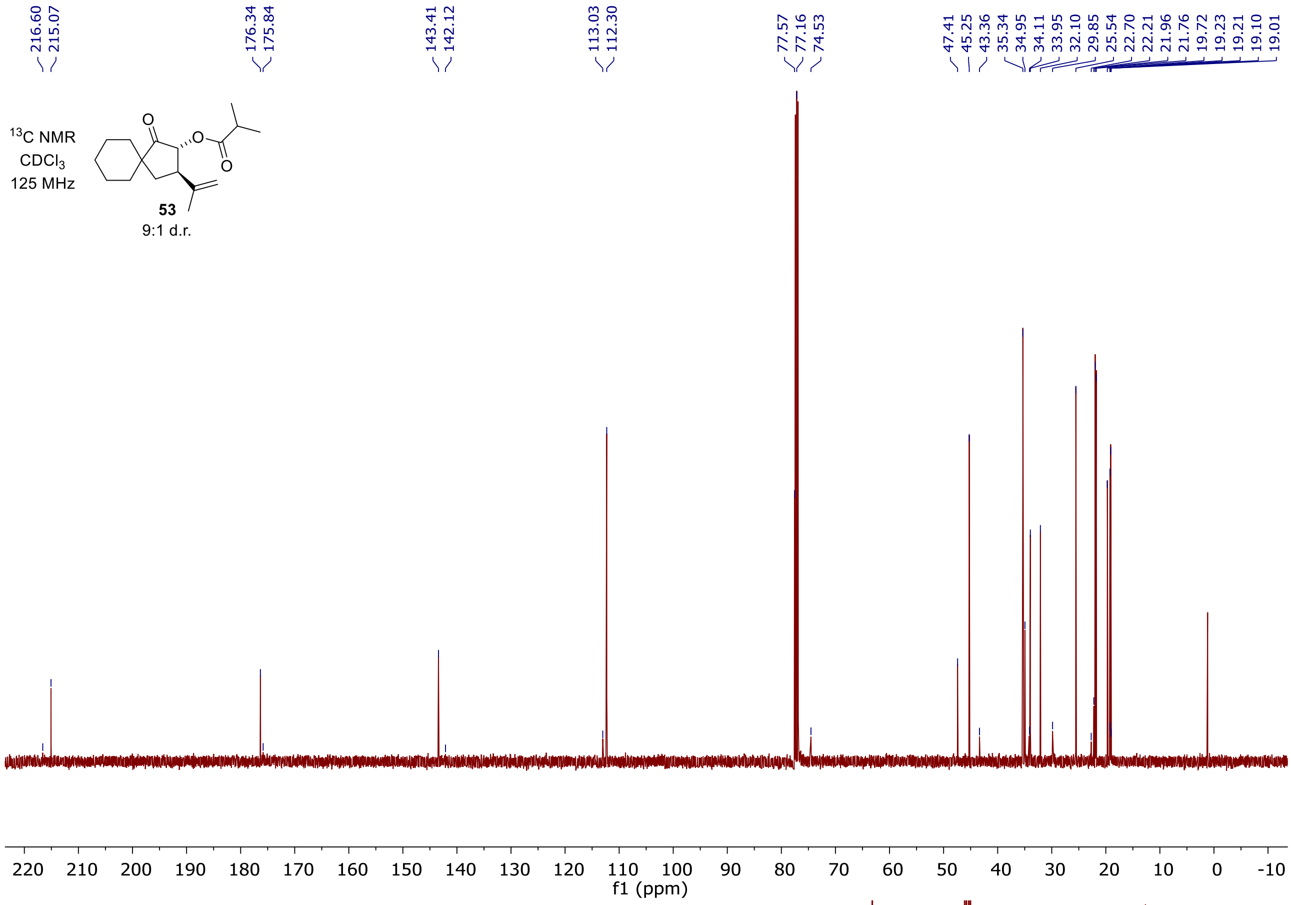
216.60
215.07
176.34
175.84
143.41
142.12
113.03
112.30
77.57
77.16
74.53
47.41
45.25
43.36
35.34
34.95
34.11
33.95
32.10
29.85
25.54
22.70
22.21
21.96
21.76
19.72
19.23
19.21
19.10
19.01
^{13}C NMR
CDCl$_3$
125 MHz
53
9:1 d.r.
f1 (ppm)

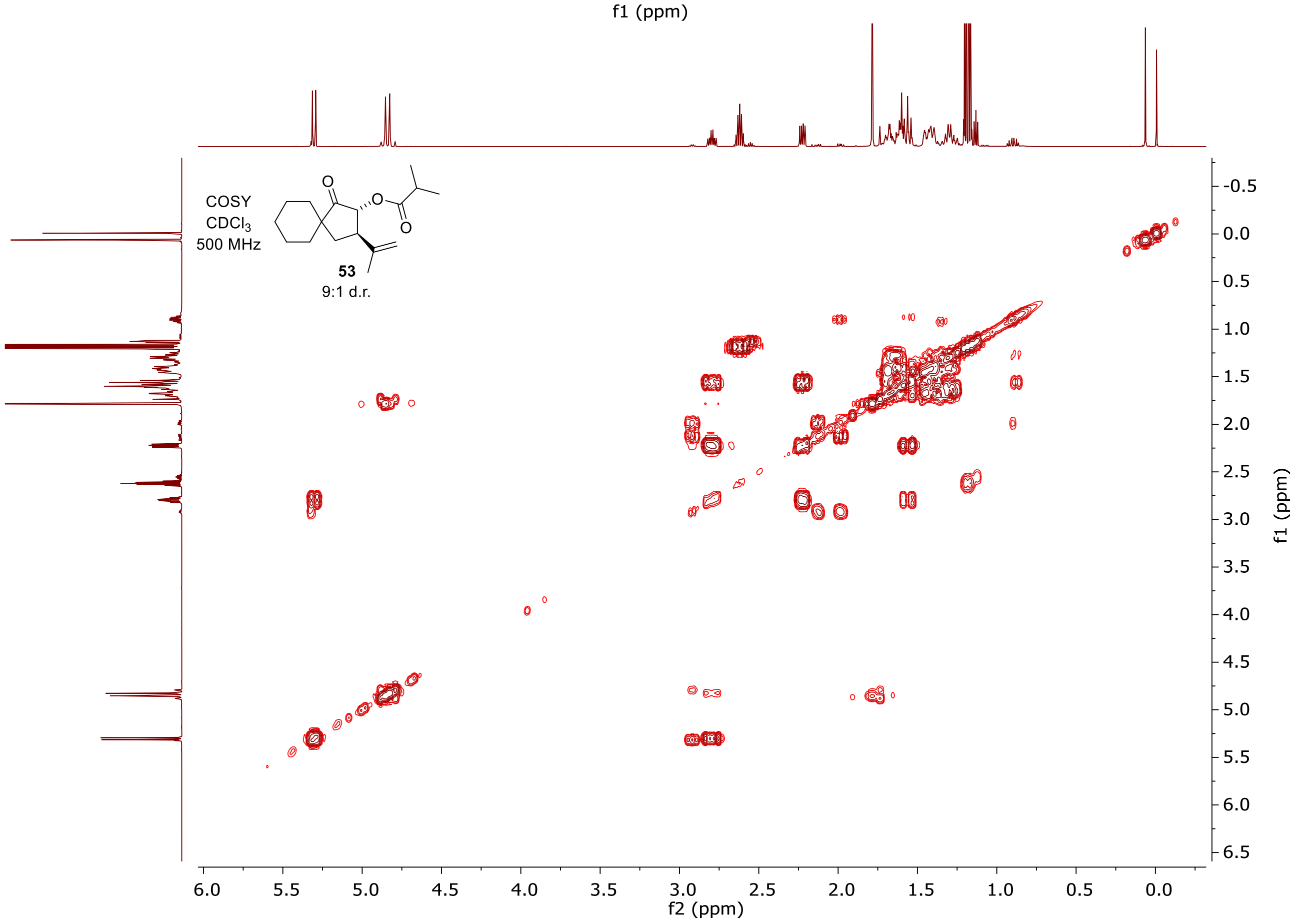
COSY
CDCl$_3$
500 MHz
53
9:1 d.r.
f2 (ppm)
f1 (ppm)

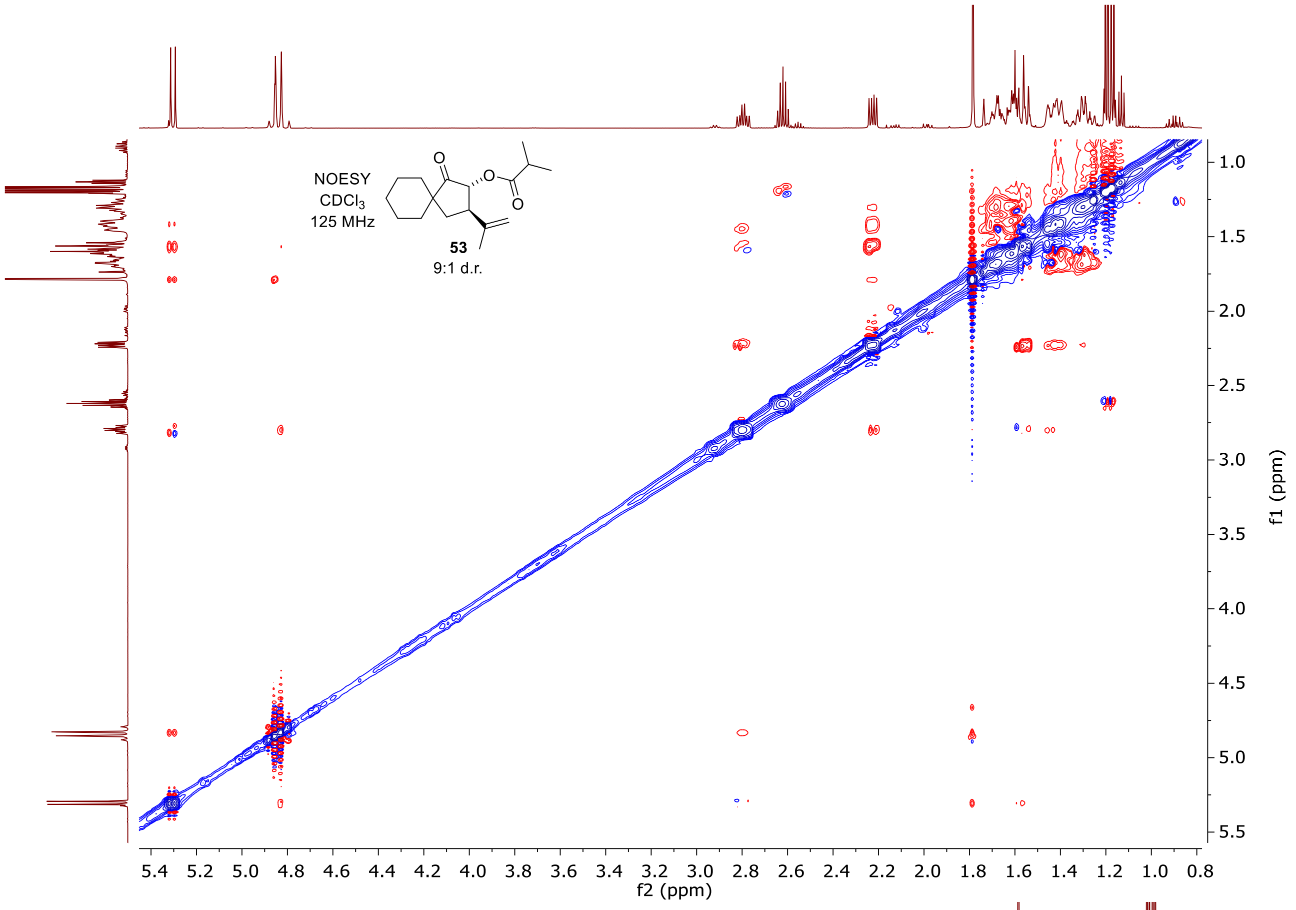
NOESY
CDCl3
125 MHz
53
9:1 d.r.
f2 (ppm)
f1 (ppm)

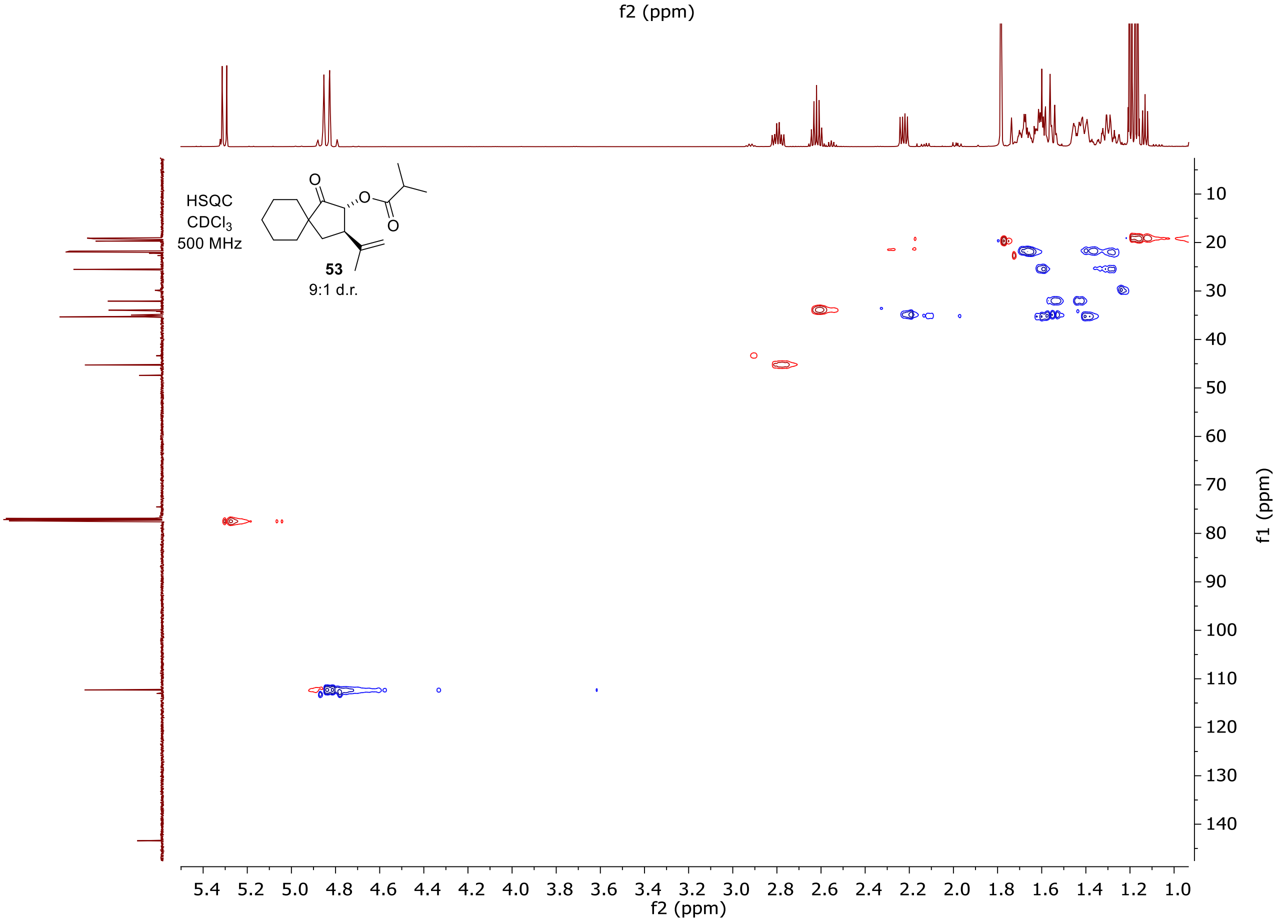
HSQC
CDCl3
500 MHz
53
9:1 d.r.
f2 (ppm)
f1 (ppm)

HMBC
$CDCl_3$
125 MHz

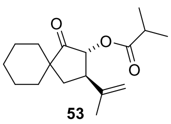

53
9:1 d.r.

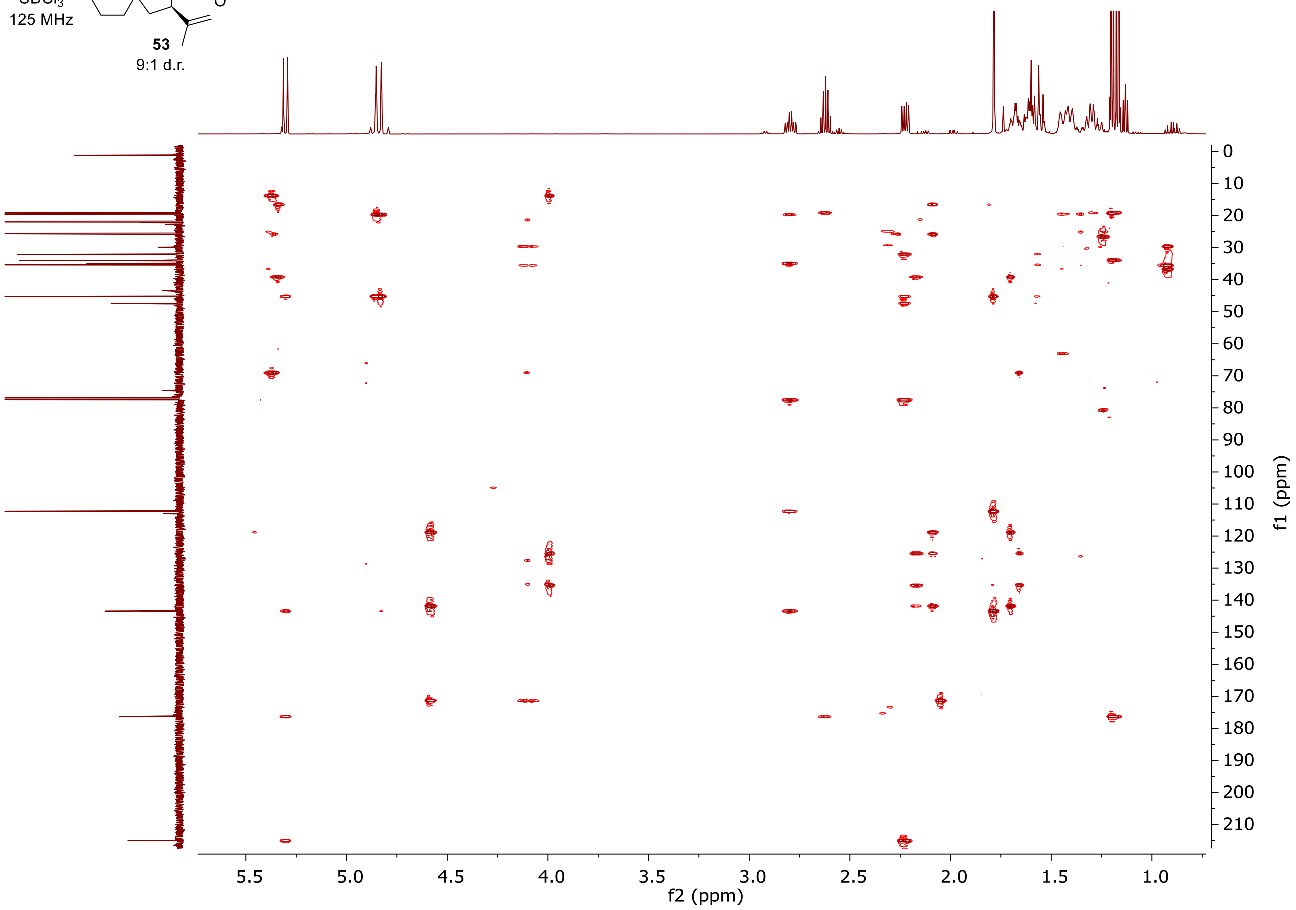

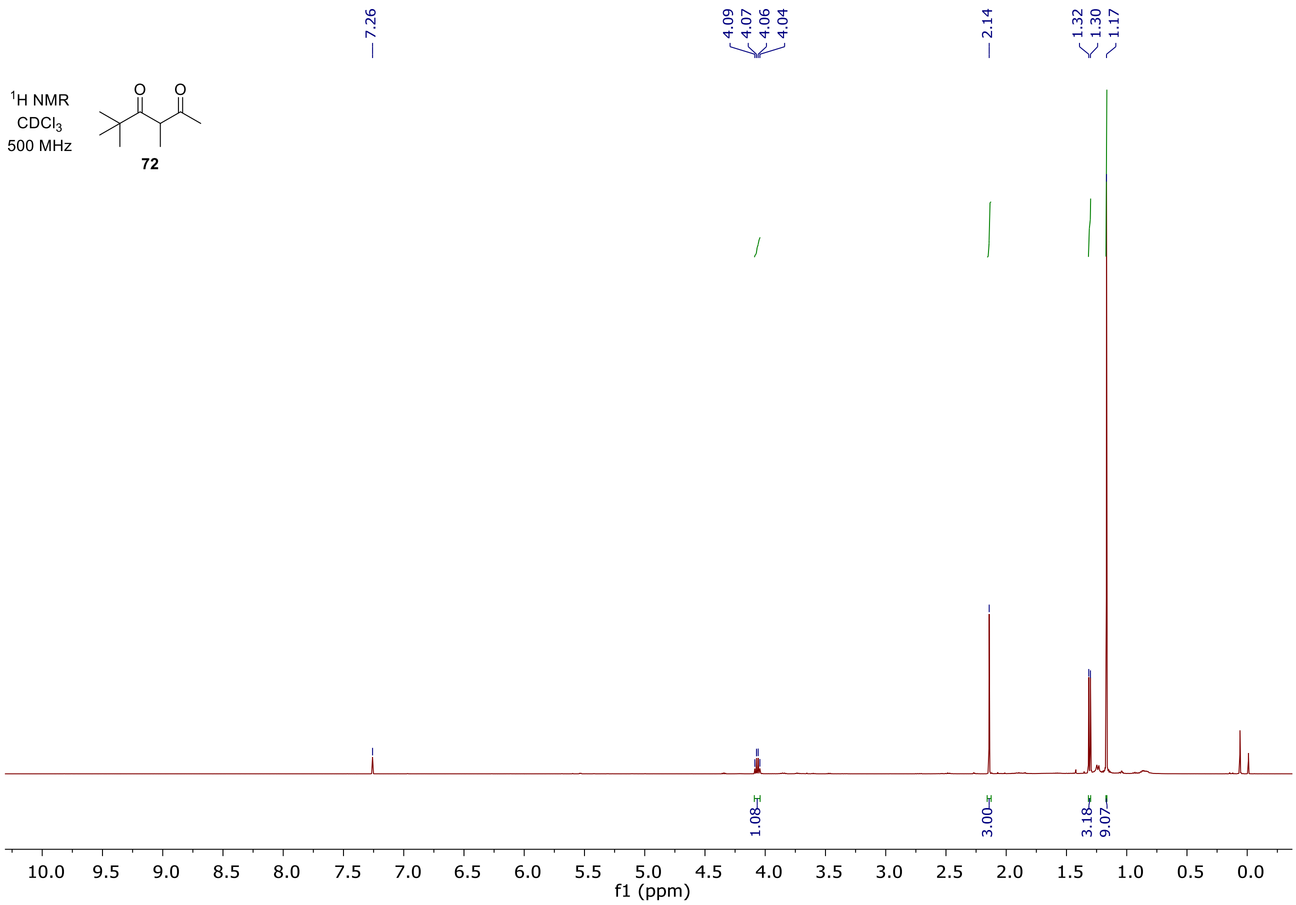
^{1}H NMR
$CDCl_3$
500 MHz
72
7.26
4.09
4.07
4.06
4.04
2.14
1.32
1.30
1.17
1.08
3.00
3.18
9.07
f1 (ppm)

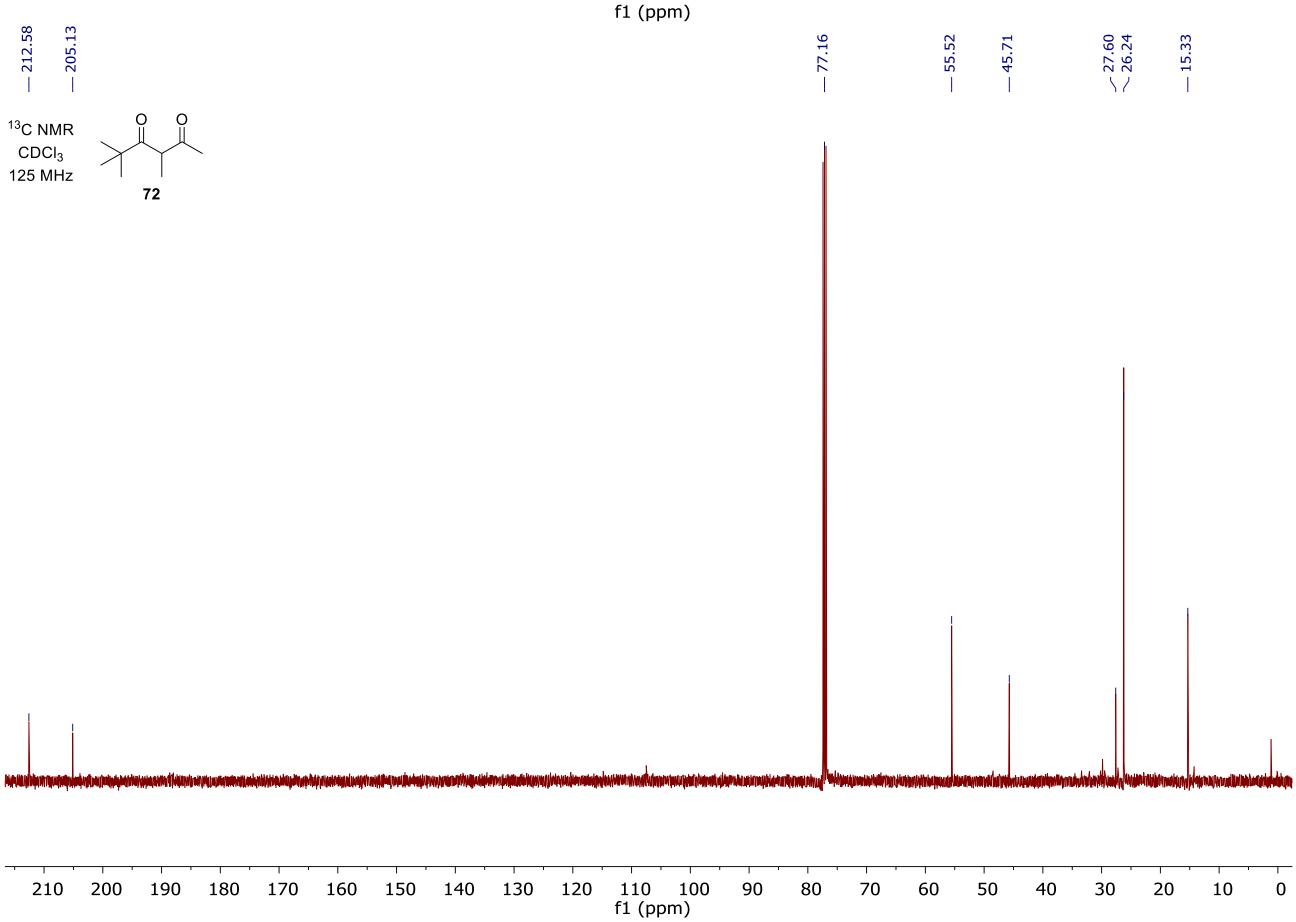
^{13}C NMR
$CDCl_3$
125 MHz
72
212.58
205.13
77.16
55.52
45.71
27.60
26.24
15.33
f1 (ppm)

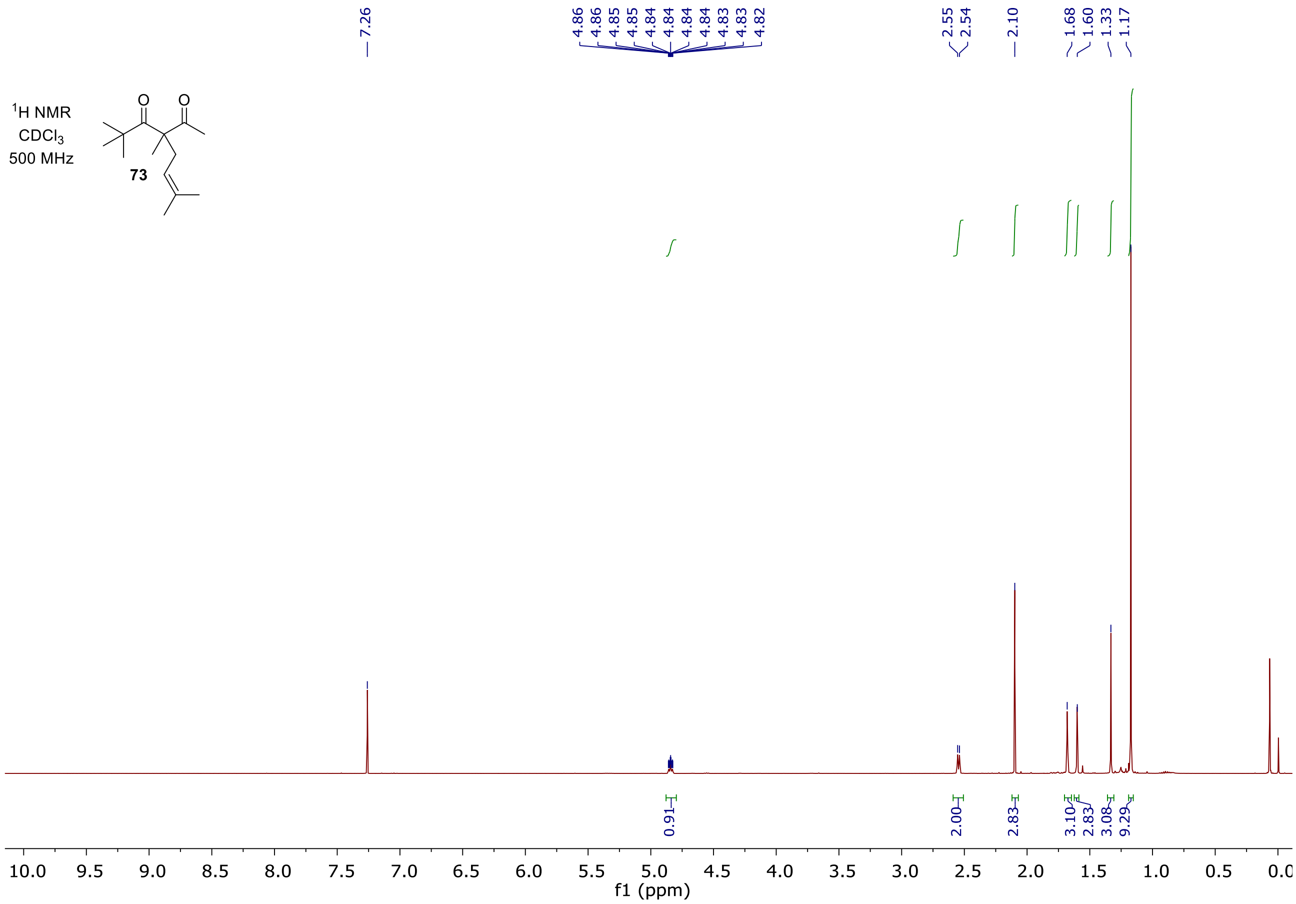

¹H NMR
CDCl₃
500 MHz
73
7.26
4.86 4.86 4.85 4.85 4.84 4.84 4.84 4.84 4.83 4.83 4.82
2.55 2.54
2.10
1.68 1.60 1.33 1.17
0.91
2.00
2.83
3.10 2.83 3.08 9.29
10.0 9.5 9.0 8.5 8.0 7.5 7.0 6.5 6.0 5.5 5.0 4.5 4.0 3.5 3.0 2.5 2.0 1.5 1.0 0.5 0.0
f1 (ppm)

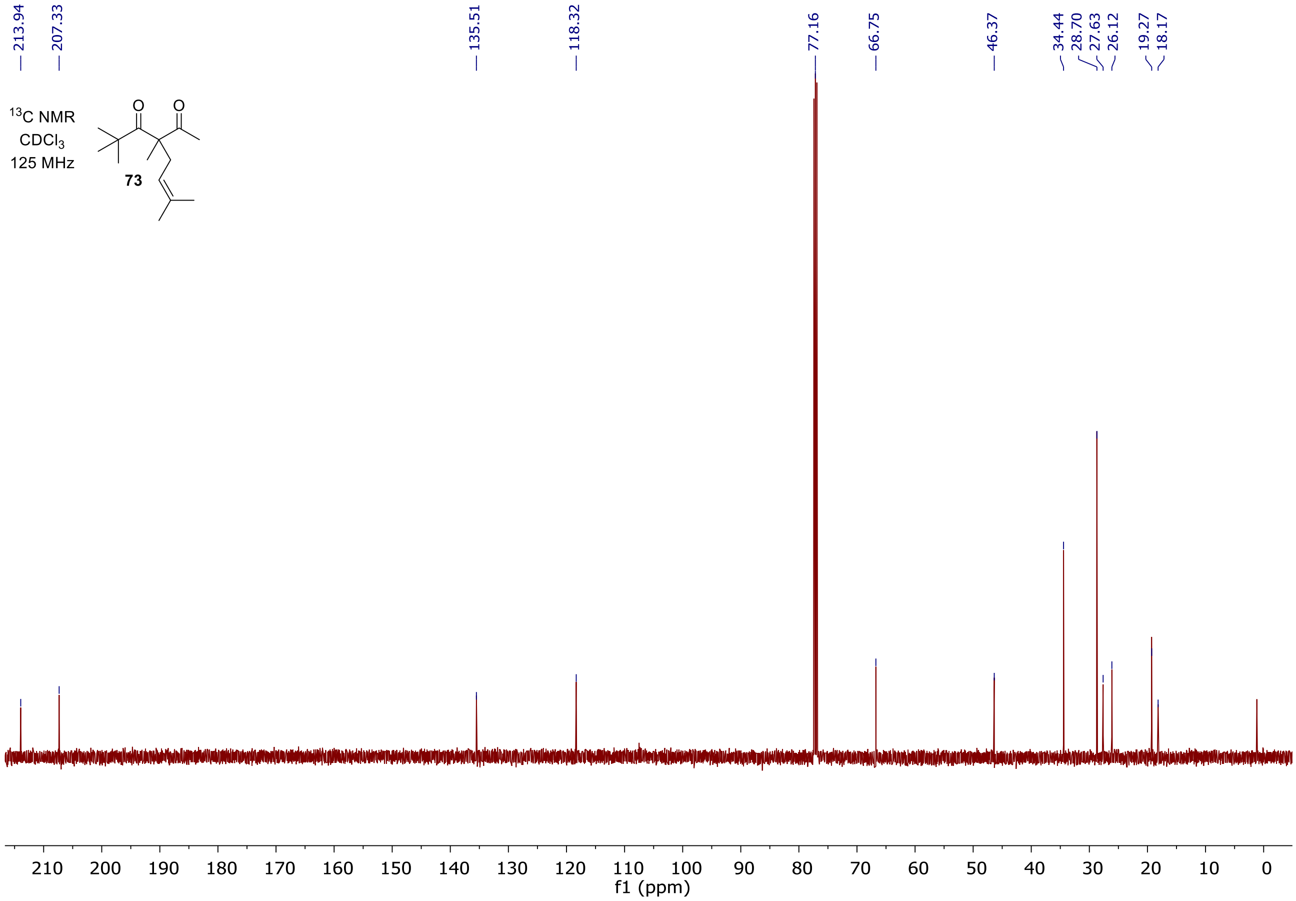

¹³C NMR
CDCl₃
125 MHz
73
213.94
207.33
135.51
118.32
77.16
66.75
46.37
34.44 28.70 27.63 26.12
19.27 18.17
210 200 190 180 170 160 150 140 130 120 110 100 90 80 70 60 50 40 30 20 10 0
f1 (ppm)

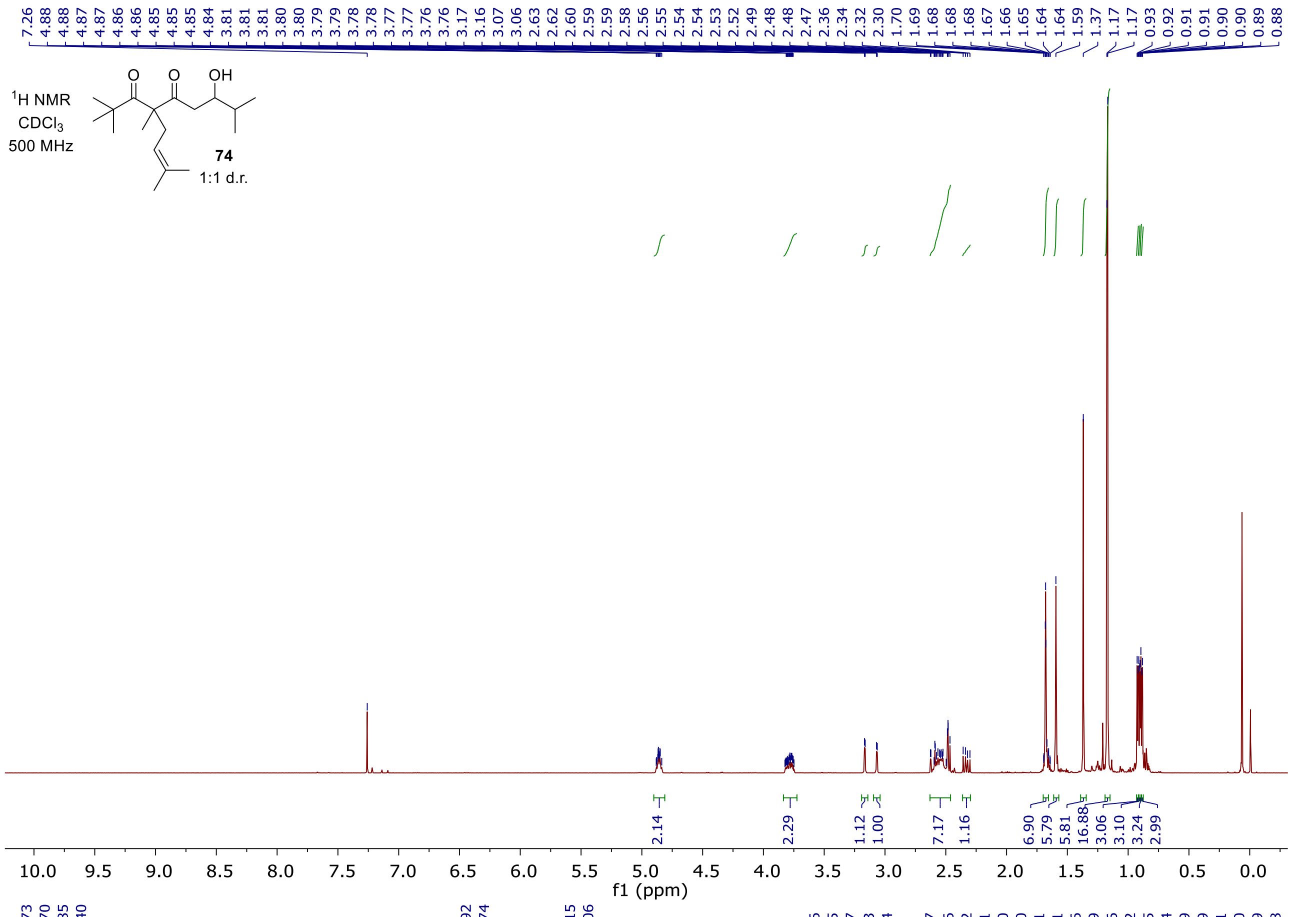

1H NMR
CDCl3
500 MHz
74
1:1 d.r.
f1 (ppm)

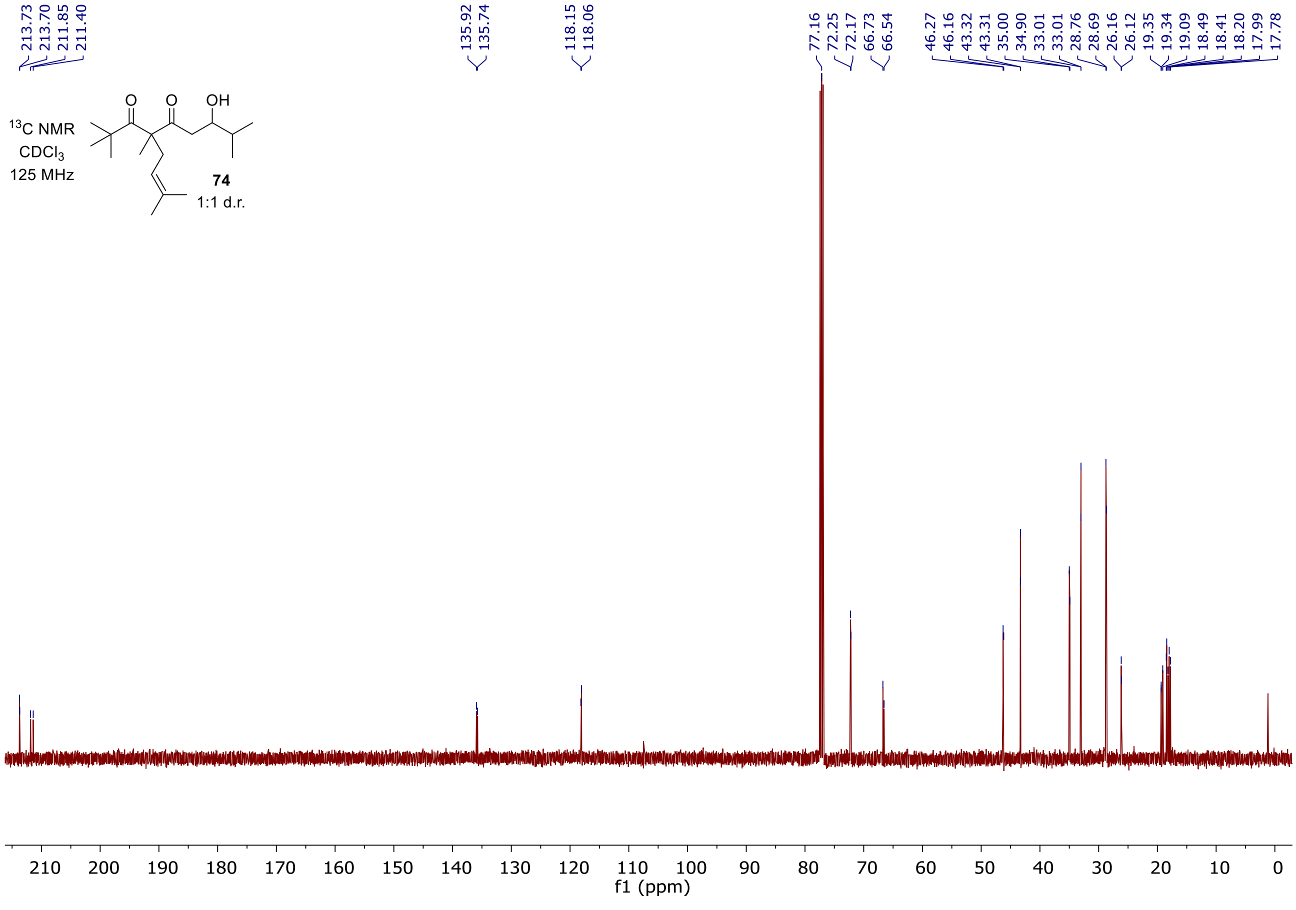

13C NMR
CDCl3
125 MHz
74
1:1 d.r.
f1 (ppm)

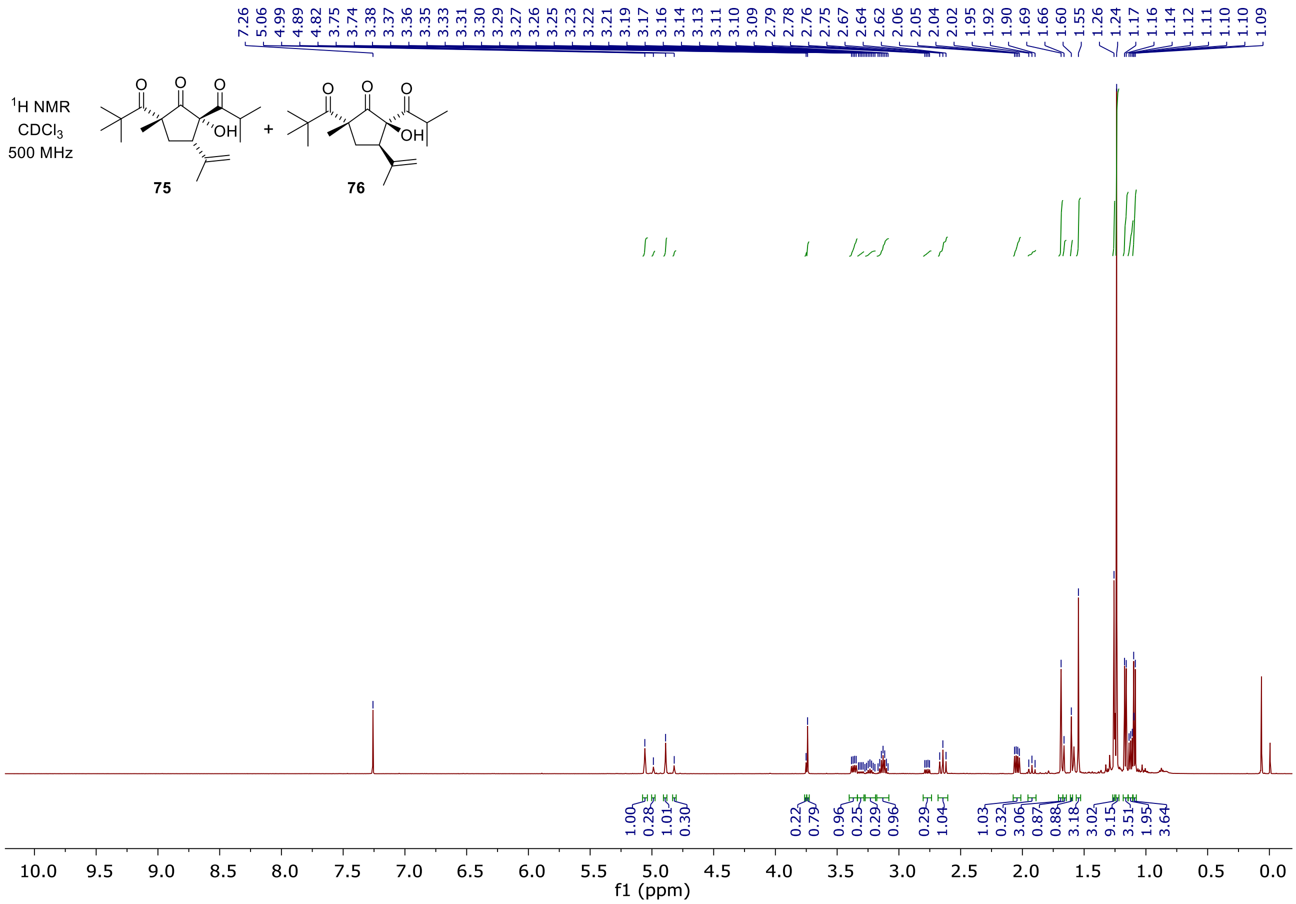

7.26 5.06 4.99 4.89 4.82 3.75 3.74 3.38 3.37 3.36 3.35 3.33 3.31 3.30 3.29 3.27 3.26 3.25 3.23 3.22 3.21 3.19 3.17 3.16 3.14 3.13 3.11 3.10 3.09 2.79 2.78 2.76 2.75 2.67 2.64 2.62 2.06 2.05 2.04 2.02 1.95 1.92 1.90 1.69 1.66 1.60 1.55 1.26 1.24 1.17 1.16 1.14 1.12 1.11 1.10 1.09
^{1}H NMR
$CDCl_3$
500 MHz
75
76
OH
1.00 0.28 1.01 0.30 0.22 0.79 0.96 0.25 0.29 0.96 0.29 1.04 1.03 0.32 3.06 0.87 0.88 3.18 3.02 9.15 3.51 1.95 3.64
10.0 9.5 9.0 8.5 8.0 7.5 7.0 6.5 6.0 5.5 5.0 4.5 4.0 3.5 3.0 2.5 2.0 1.5 1.0 0.5 0.0
f1 (ppm)

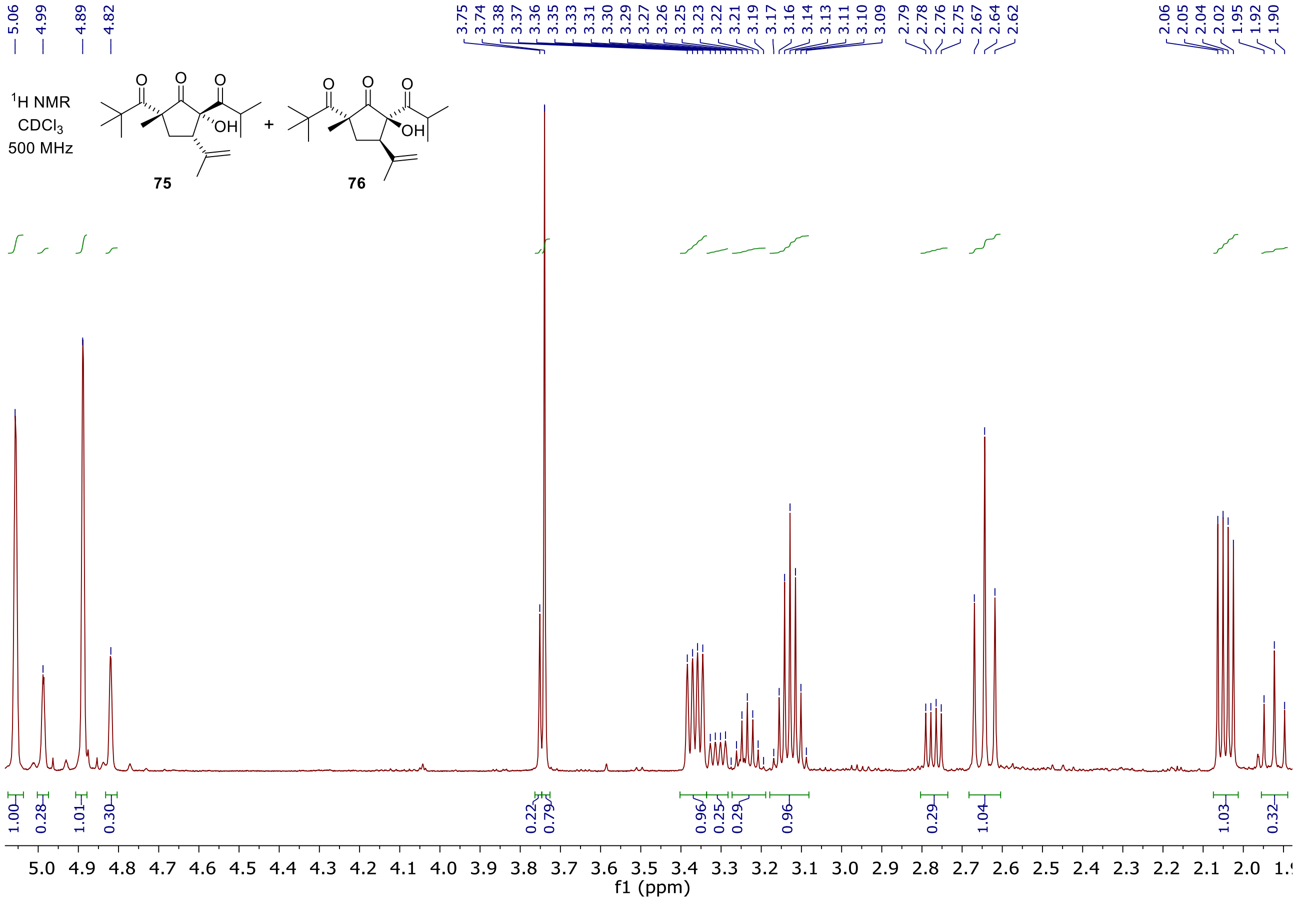

5.06 4.99 4.89 4.82 3.75 3.74 3.38 3.37 3.36 3.35 3.33 3.31 3.30 3.29 3.27 3.26 3.25 3.23 3.22 3.21 3.19 3.17 3.16 3.14 3.13 3.11 3.10 3.09 2.79 2.78 2.76 2.75 2.67 2.64 2.62 2.06 2.05 2.04 2.02 1.95 1.92 1.90
^{1}H NMR
$CDCl_3$
500 MHz
75
76
OH
1.00 0.28 1.01 0.30 0.22 0.79 0.96 0.25 0.29 0.96 0.29 1.04 1.03 0.32
5.0 4.9 4.8 4.7 4.6 4.5 4.4 4.3 4.2 4.1 4.0 3.9 3.8 3.7 3.6 3.5 3.4 3.3 3.2 3.1 3.0 2.9 2.8 2.7 2.6 2.5 2.4 2.3 2.2 2.1 2.0
f1 (ppm)

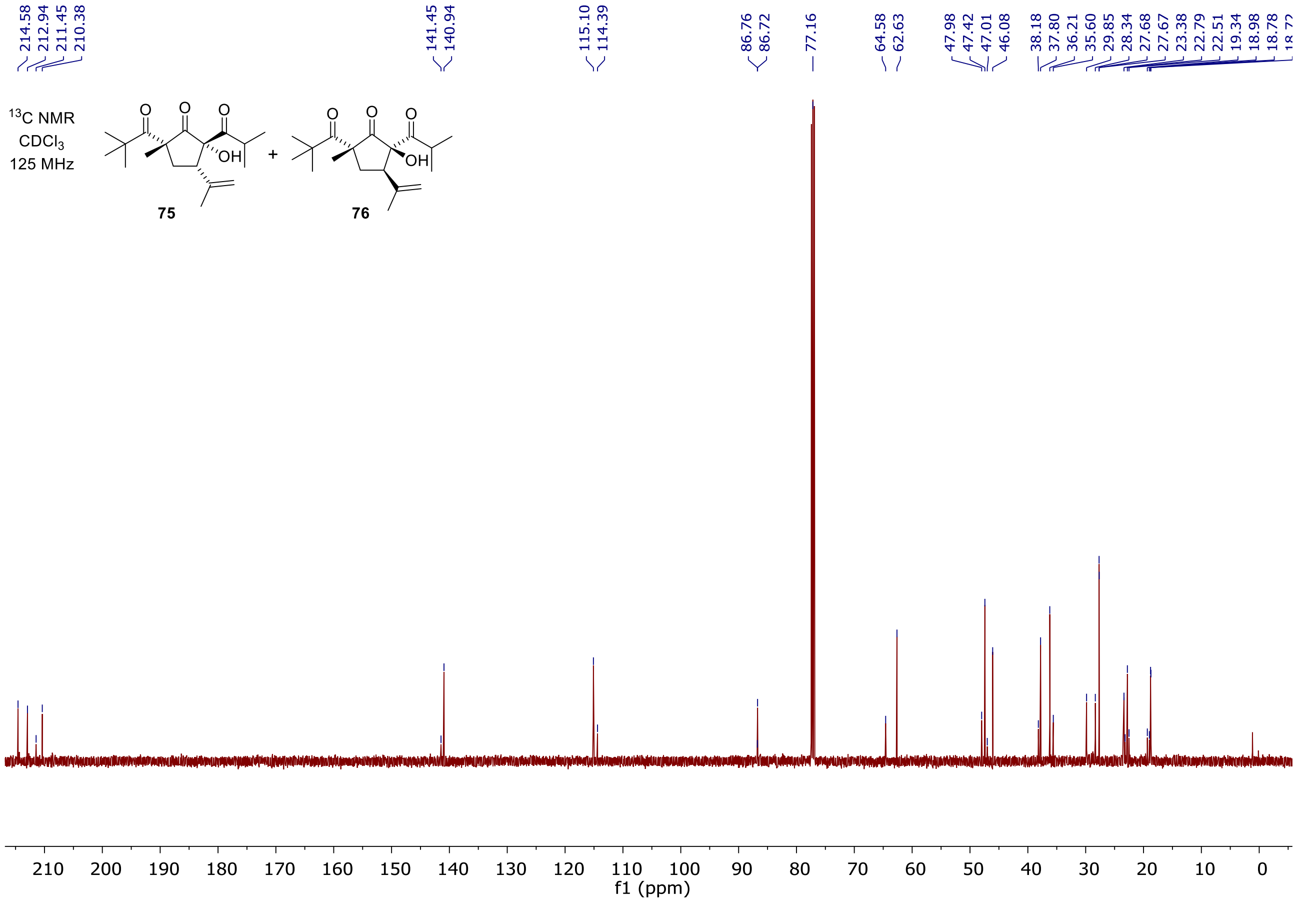
214.58
212.94
211.45
210.38
141.45
140.94
115.10
114.39
86.76
86.72
77.16
64.58
62.63
47.98
47.42
47.01
46.08
38.18
37.80
36.21
35.60
29.85
28.34
27.68
27.67
23.38
22.79
22.51
19.34
18.98
18.78
^{13}C NMR
$CDCl_3$
125 MHz
OH
OH
75
76
f1 (ppm)

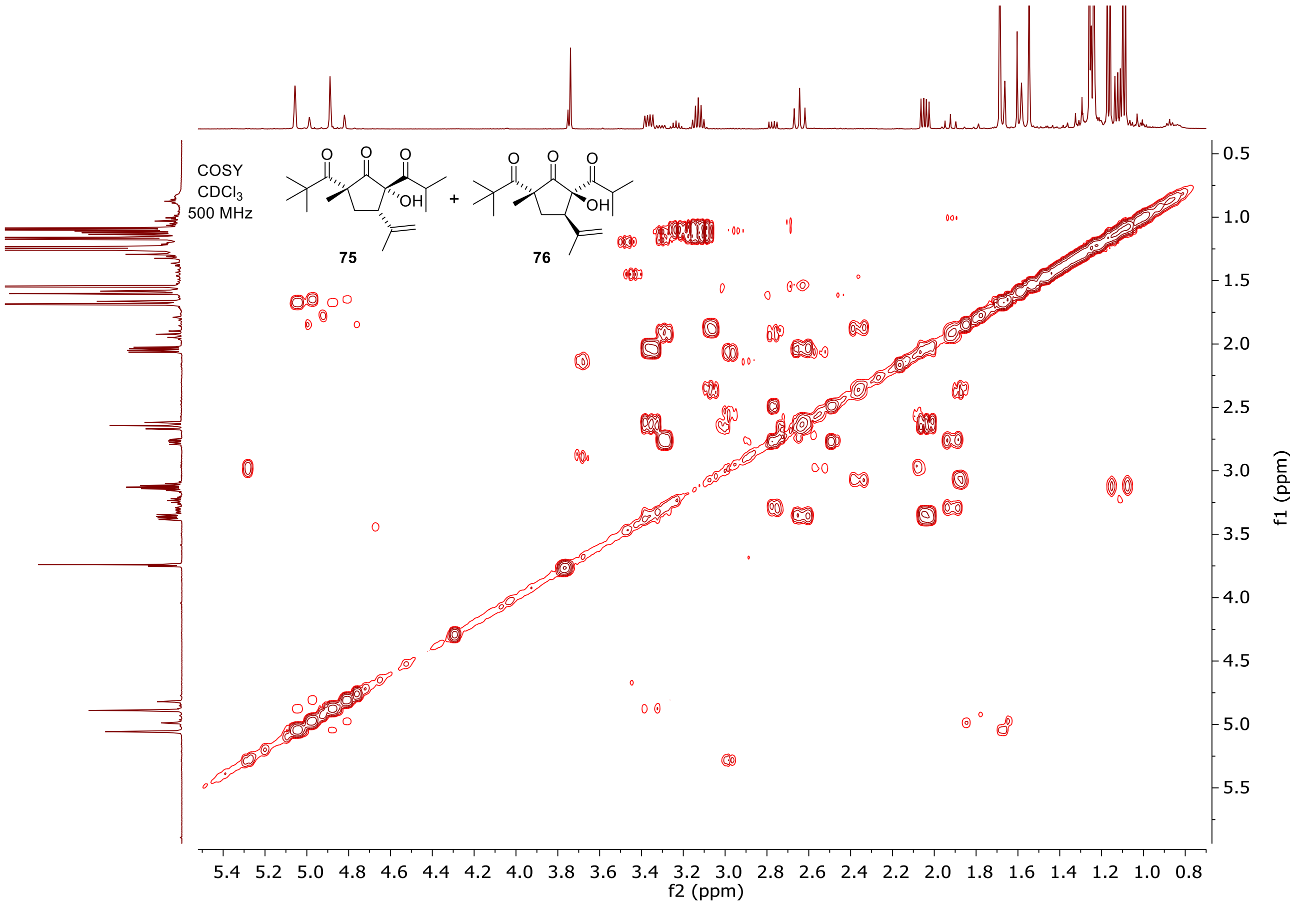
COSY
$CDCl_3$
500 MHz
OH
OH
75
76
f2 (ppm)
f1 (ppm)

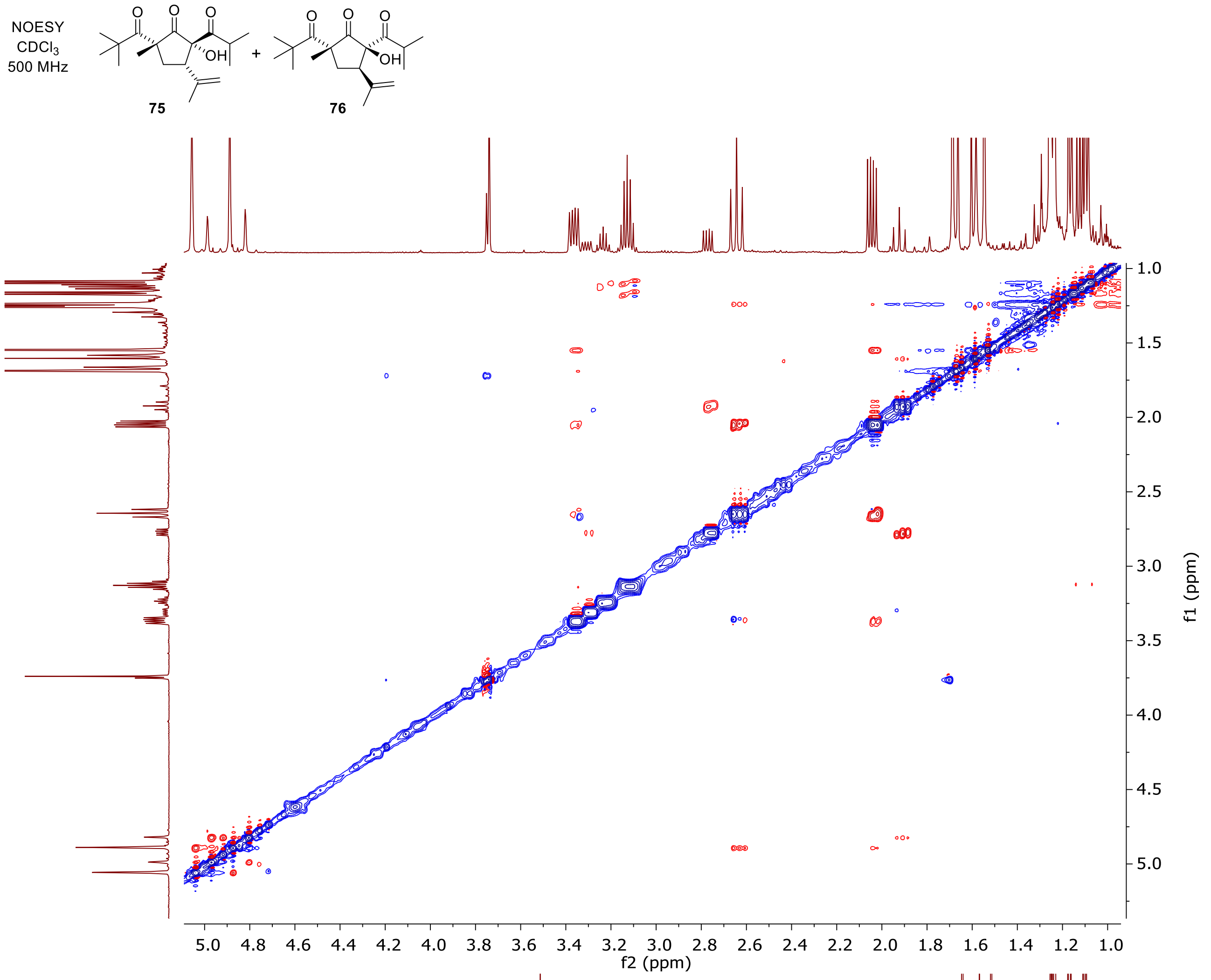
NOESY
CDCl3
500 MHz
75
76
f2 (ppm)
f1 (ppm)

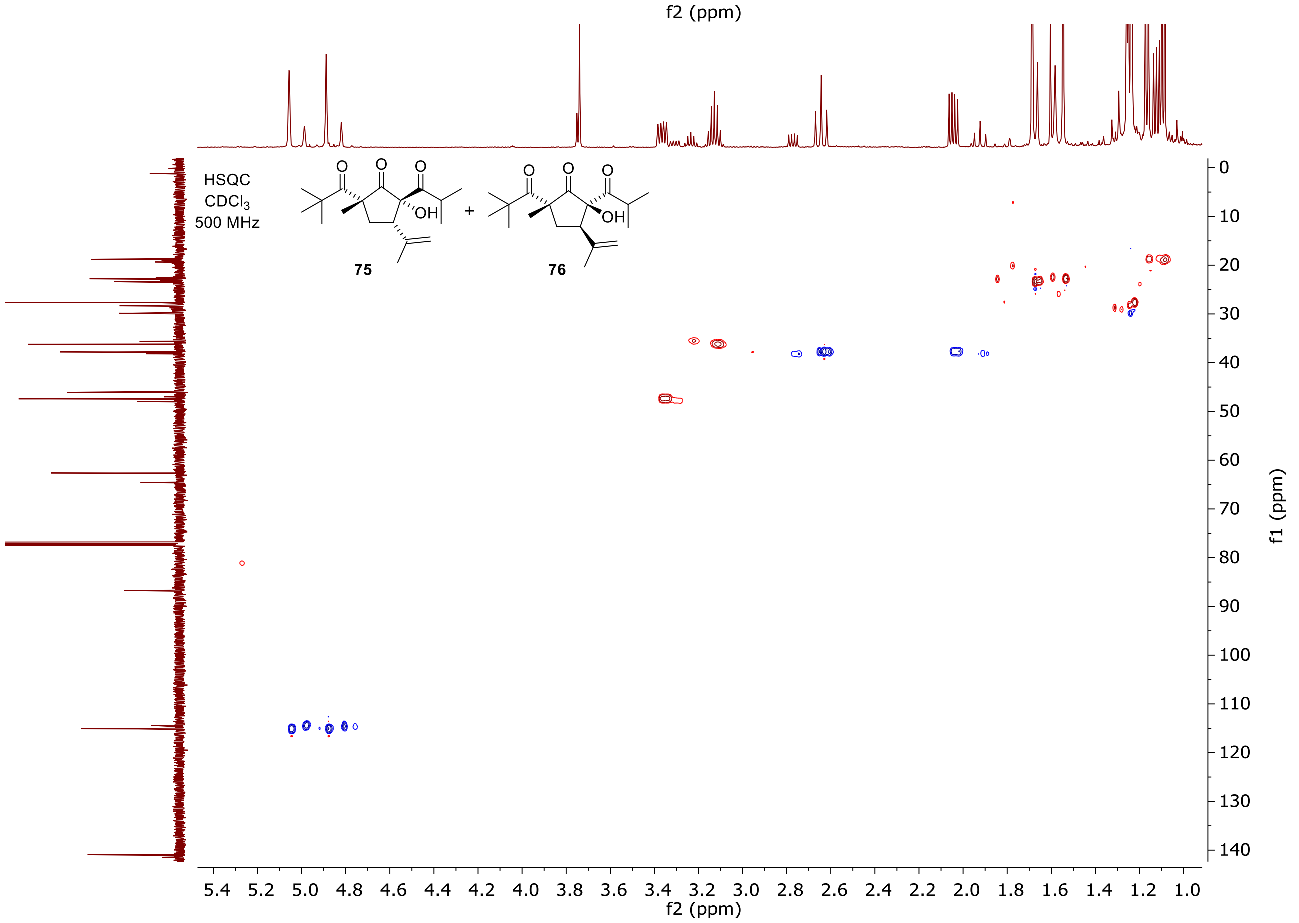
HSQC
CDCl3
500 MHz
75
76
f2 (ppm)
f1 (ppm)

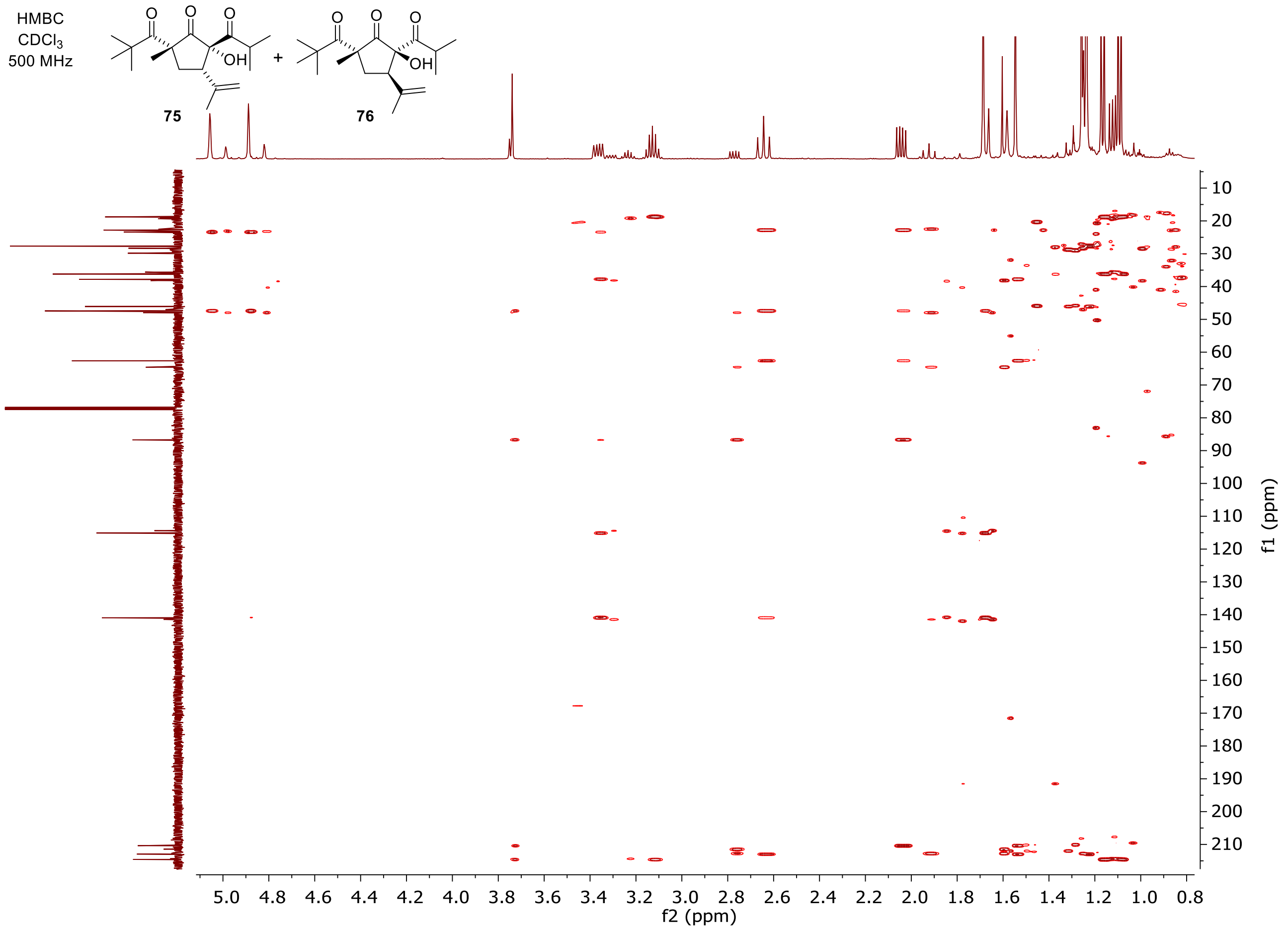
HMBC
CDCl3
500 MHz
75
76
f2 (ppm)
f1 (ppm)

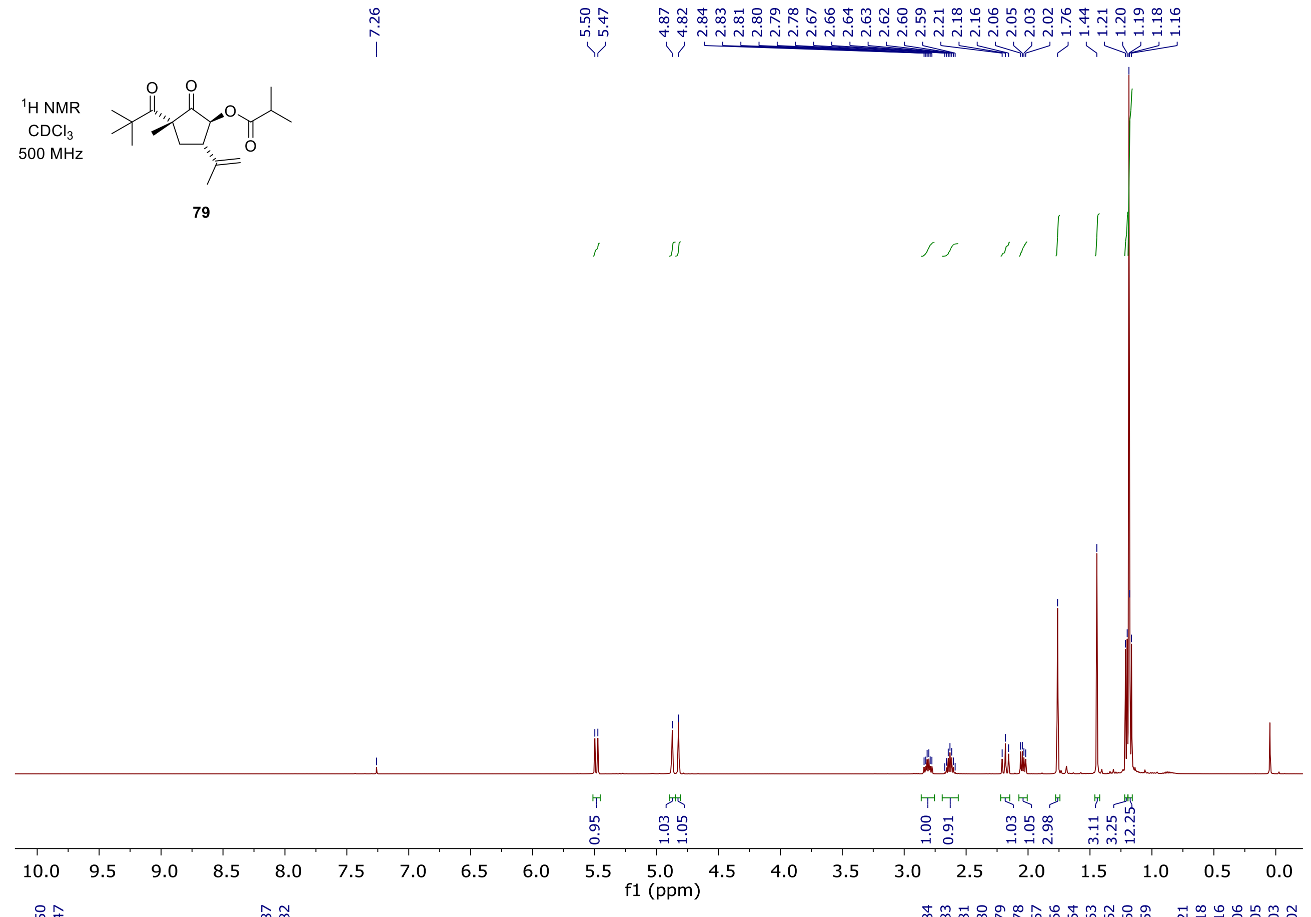

7.26
5.50
5.47
4.87
4.82
2.84
2.83
2.81
2.80
2.79
2.78
2.67
2.66
2.64
2.63
2.62
2.60
2.59
2.21
2.18
2.16
2.06
2.05
2.03
2.02
1.76
1.44
1.21
1.20
1.19
1.18
1.16
^{1}H NMR
$CDCl_3$
500 MHz
79
0.95
1.03
1.05
1.00
0.91
1.03
1.05
2.98
3.11
3.25
12.25
10.0 9.5 9.0 8.5 8.0 7.5 7.0 6.5 6.0 5.5 5.0 4.5 4.0 3.5 3.0 2.5 2.0 1.5 1.0 0.5 0.0
f1 (ppm)

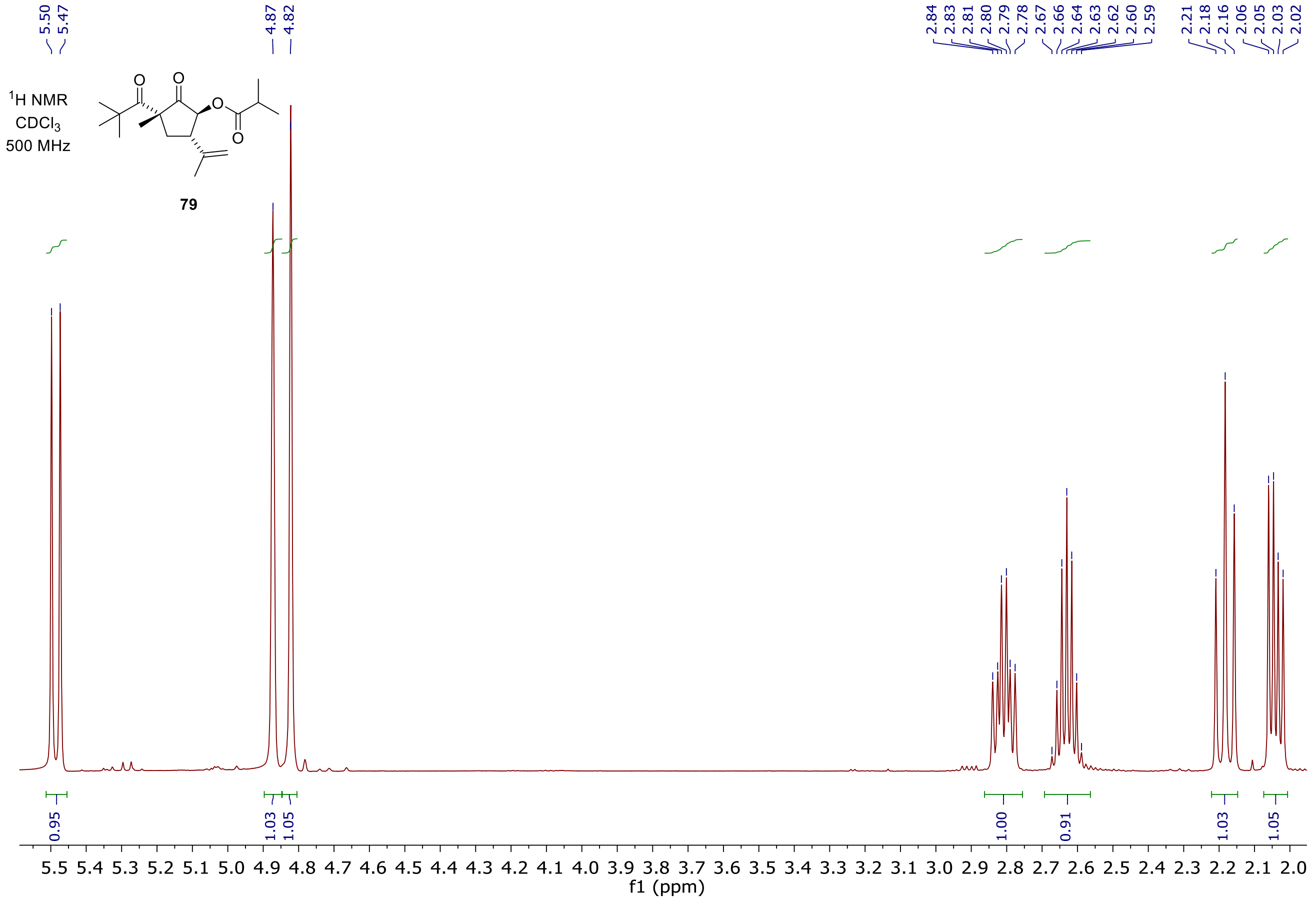

5.50
5.47
4.87
4.82
2.84
2.83
2.81
2.80
2.79
2.78
2.67
2.66
2.64
2.63
2.62
2.60
2.59
2.21
2.18
2.16
2.06
2.05
2.03
2.02
^{1}H NMR
$CDCl_3$
500 MHz
79
0.95
1.03
1.05
1.00
0.91
1.03
1.05
5.5 5.4 5.3 5.2 5.1 5.0 4.9 4.8 4.7 4.6 4.5 4.4 4.3 4.2 4.1 4.0 3.9 3.8 3.7 3.6 3.5 3.4 3.3 3.2 3.1 3.0 2.9 2.8 2.7 2.6 2.5 2.4 2.3 2.2 2.1 2.0
f1 (ppm)

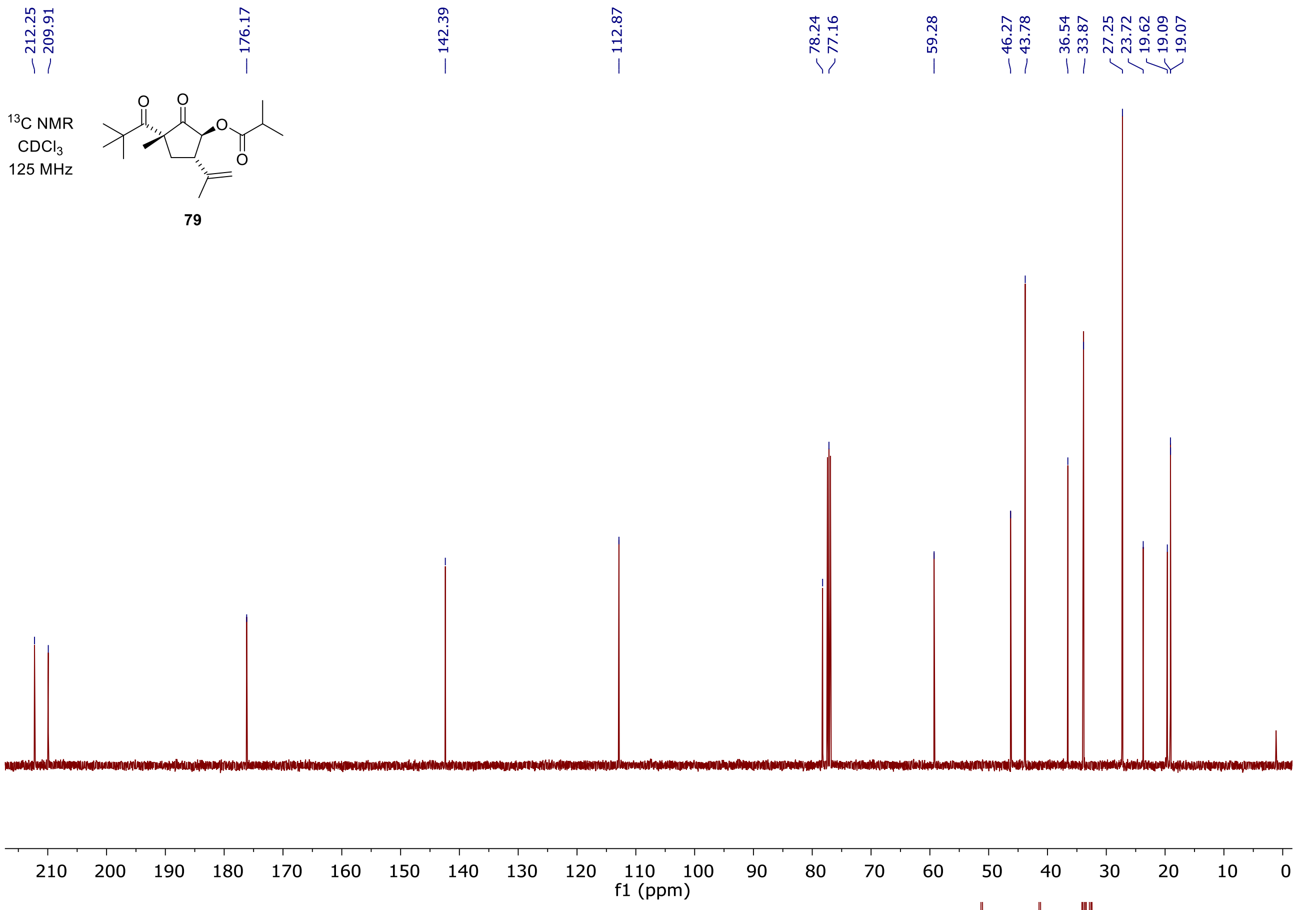
212.25
209.91
176.17
142.39
112.87
78.24
77.16
59.28
46.27
43.78
36.54
33.87
27.25
23.72
19.62
19.09
19.07
13C NMR
CDCl3
125 MHz
79
f1 (ppm)

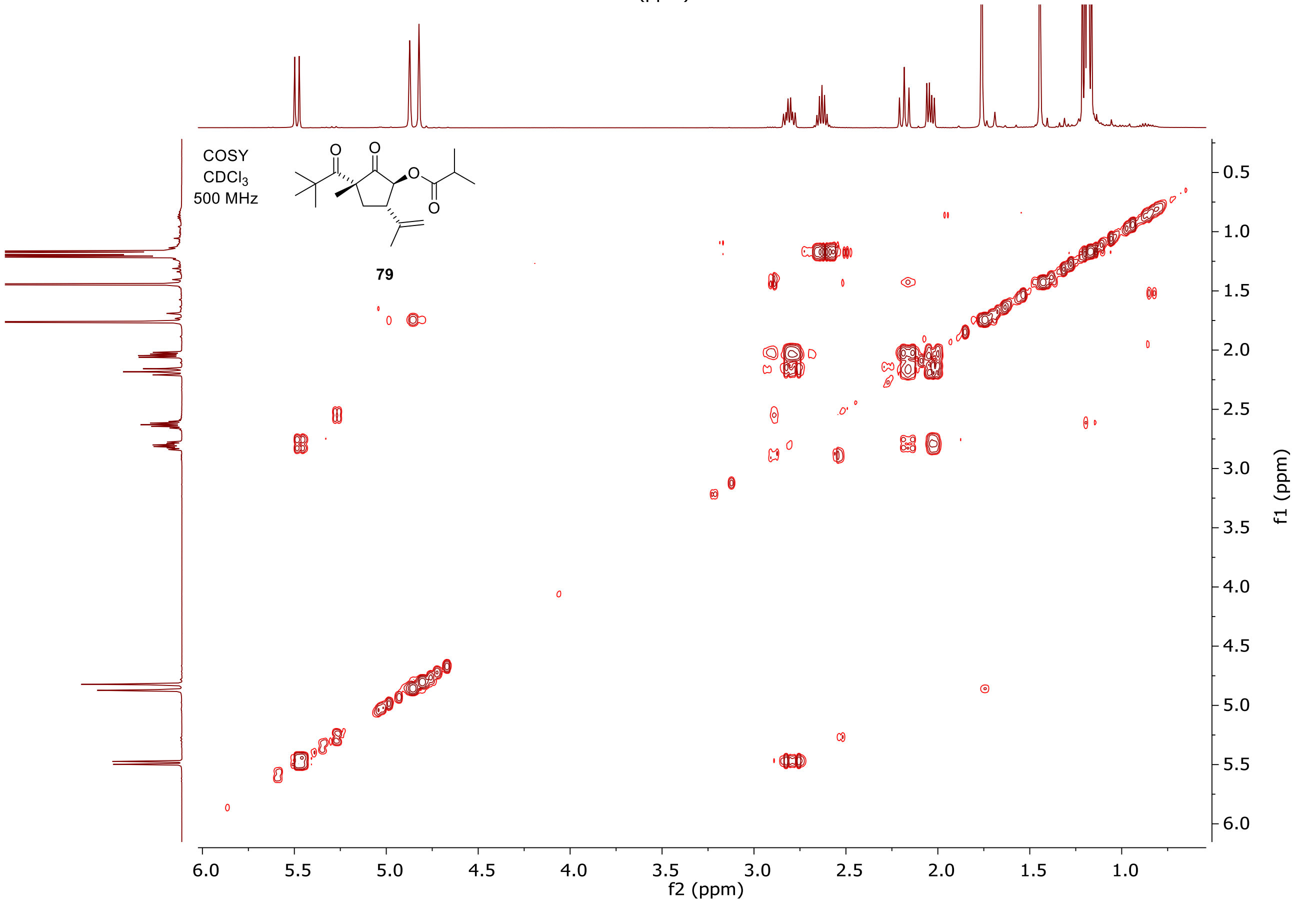
COSY
CDCl3
500 MHz
79
f2 (ppm)
f1 (ppm)

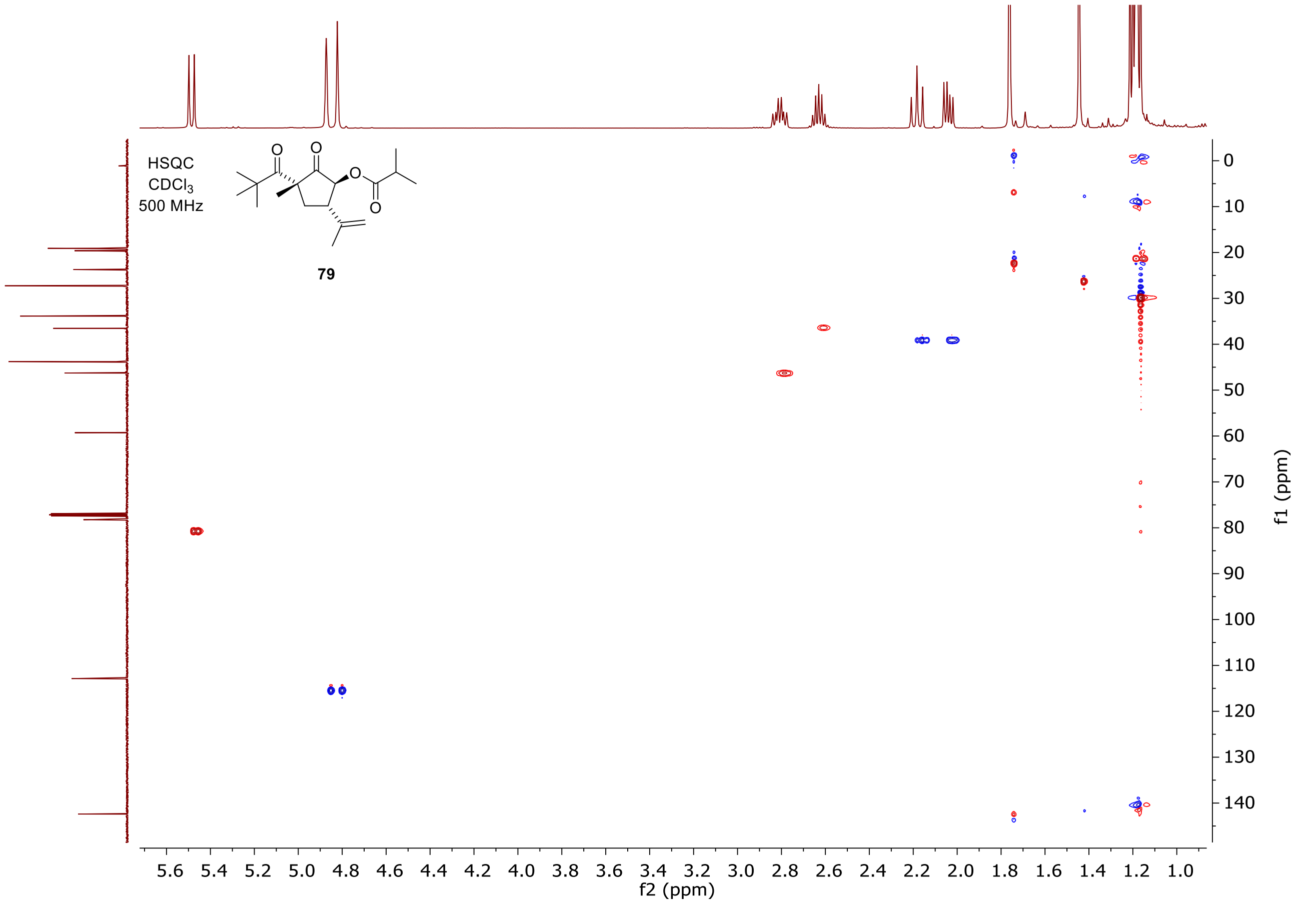
HSQC
CDCl3
500 MHz
79
f2 (ppm)
f1 (ppm)

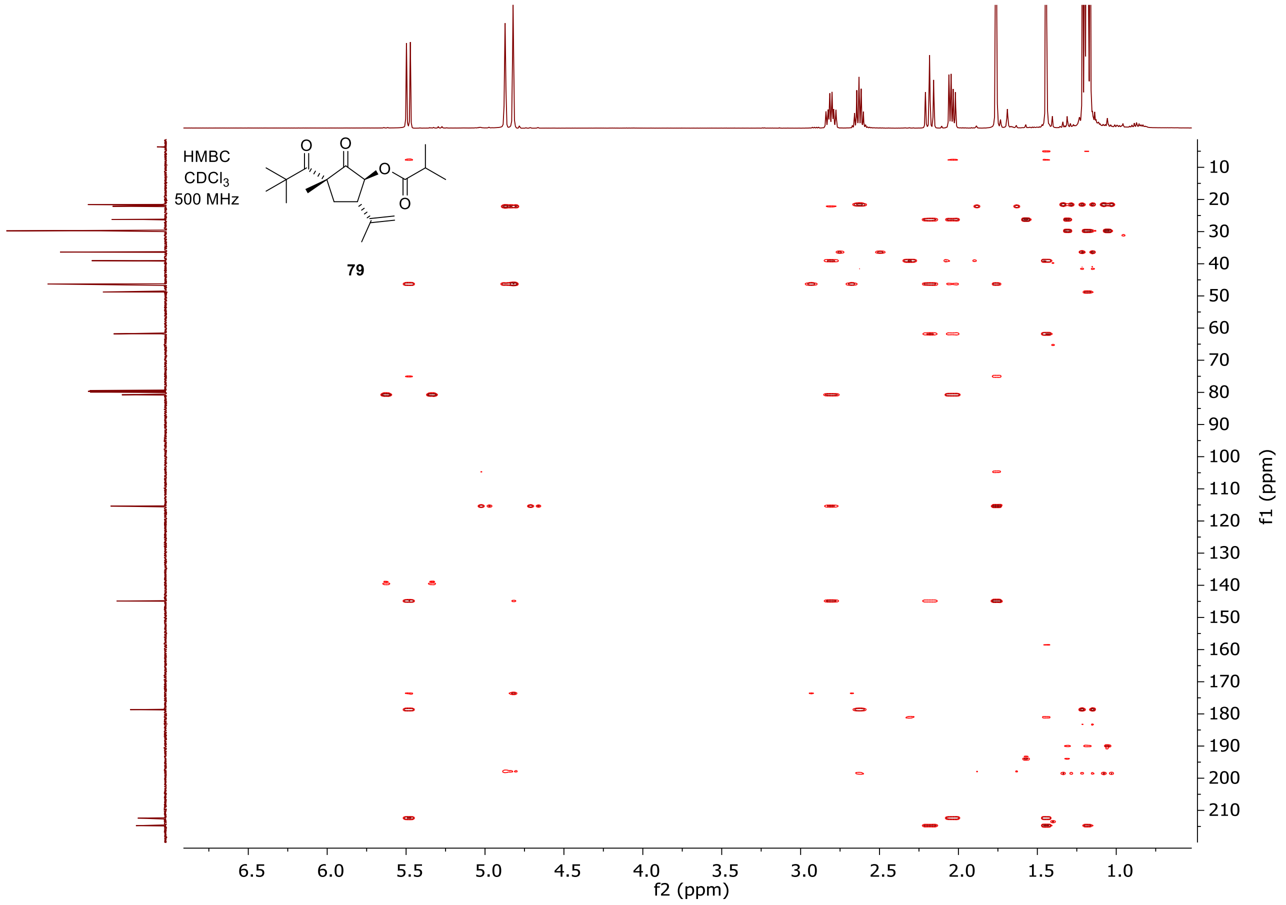
HMBC
CDCl3
500 MHz
79
f2 (ppm)
f1 (ppm)

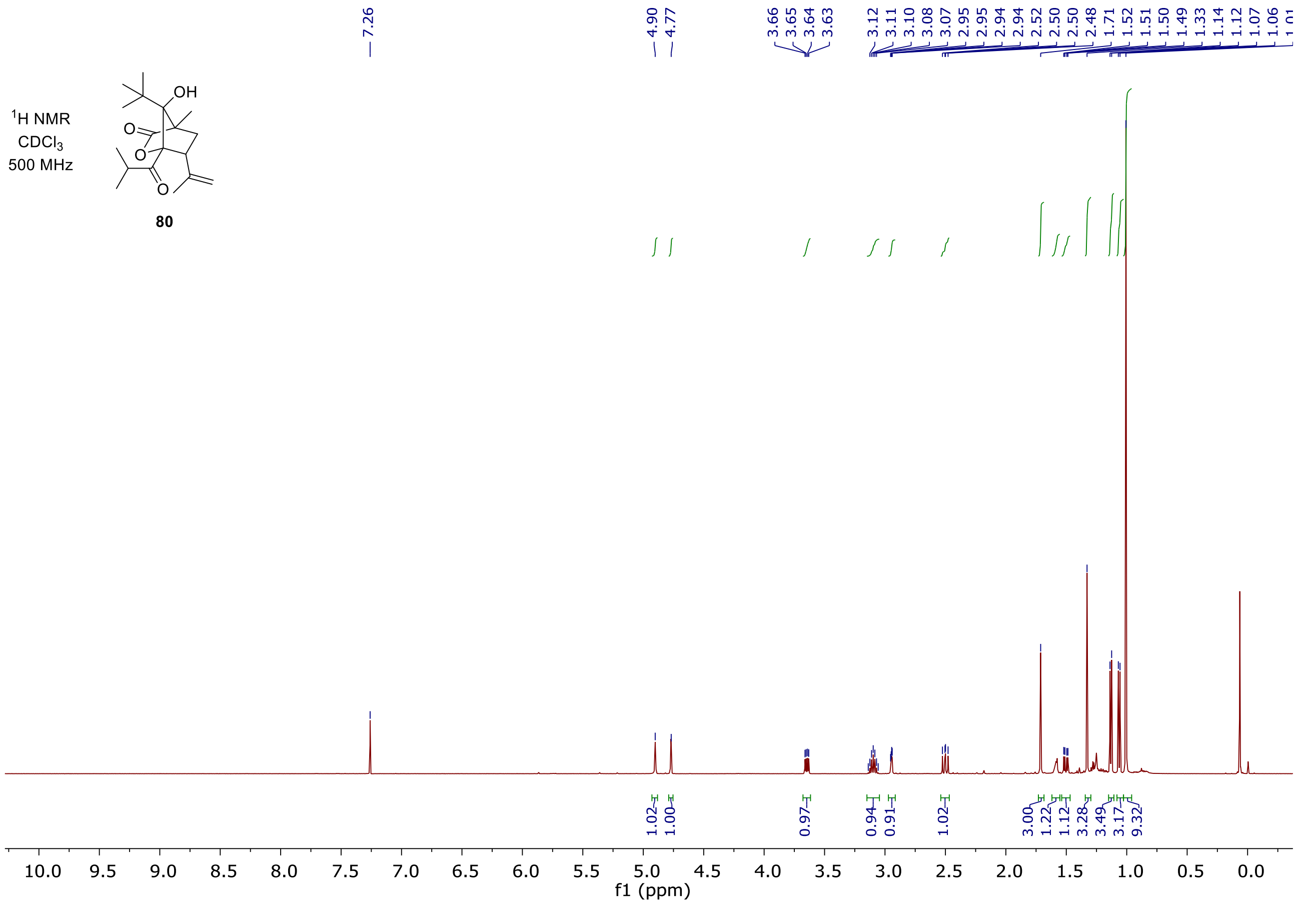
7.26
4.90
4.77
3.66
3.65
3.64
3.63
3.12
3.11
3.10
3.08
3.07
2.95
2.95
2.94
2.94
2.52
2.50
2.50
2.48
1.71
1.52
1.51
1.50
1.49
1.33
1.14
1.12
1.07
1.06
^{1}H NMR
$CDCl_3$
500 MHz
OH
O
O
O
80
1.02
1.00
0.97
0.94
0.91
1.02
3.00
1.22
1.12
3.28
3.49
3.17
9.32
10.0 9.5 9.0 8.5 8.0 7.5 7.0 6.5 6.0 5.5 5.0 4.5 4.0 3.5 3.0 2.5 2.0 1.5 1.0 0.5 0.0
f1 (ppm)

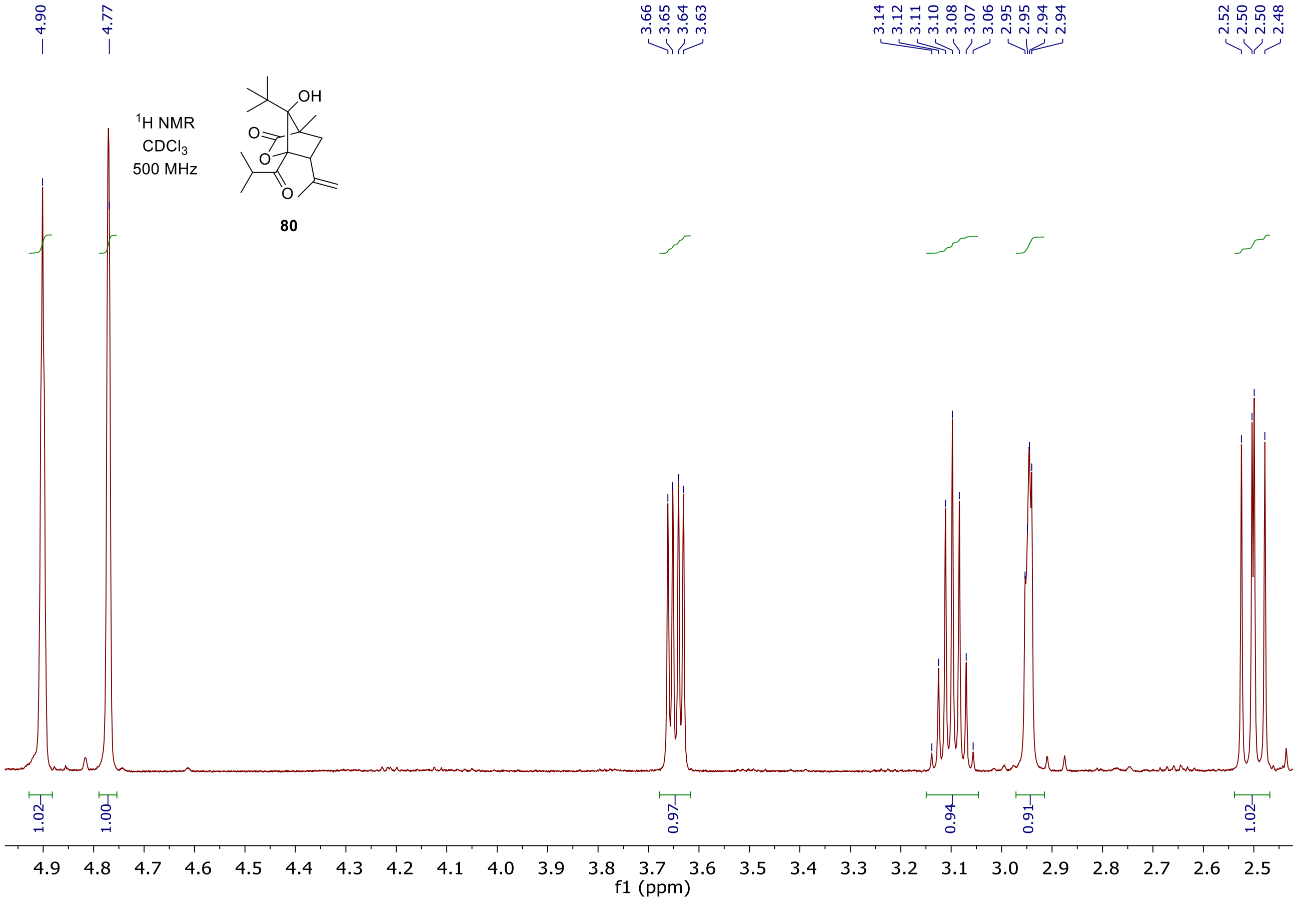
4.90
4.77
3.66
3.65
3.64
3.63
3.14
3.12
3.11
3.10
3.08
3.07
3.06
2.95
2.95
2.94
2.94
2.52
2.50
2.50
2.48
^{1}H NMR
$CDCl_3$
500 MHz
OH
O
O
O
80
1.02
1.00
0.97
0.94
0.91
1.02
4.9 4.8 4.7 4.6 4.5 4.4 4.3 4.2 4.1 4.0 3.9 3.8 3.7 3.6 3.5 3.4 3.3 3.2 3.1 3.0 2.9 2.8 2.7 2.6 2.5
f1 (ppm)

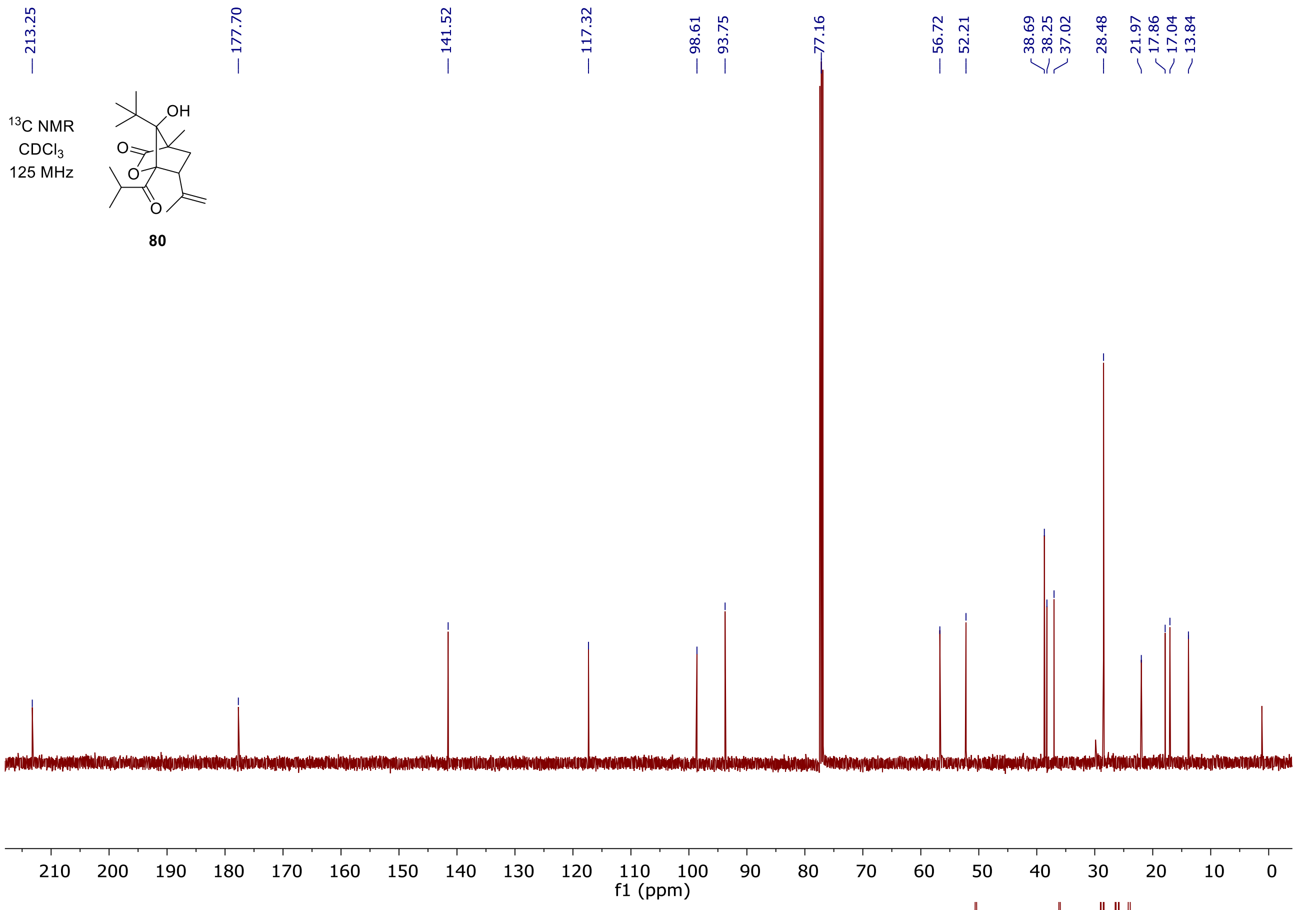

213.25
177.70
141.52
117.32
98.61
93.75
77.16
56.72
52.21
38.69
38.25
37.02
28.48
21.97
17.86
17.04
13.84
^{13}C NMR
$CDCl_3$
125 MHz
OH
O
O
O
80
f1 (ppm)

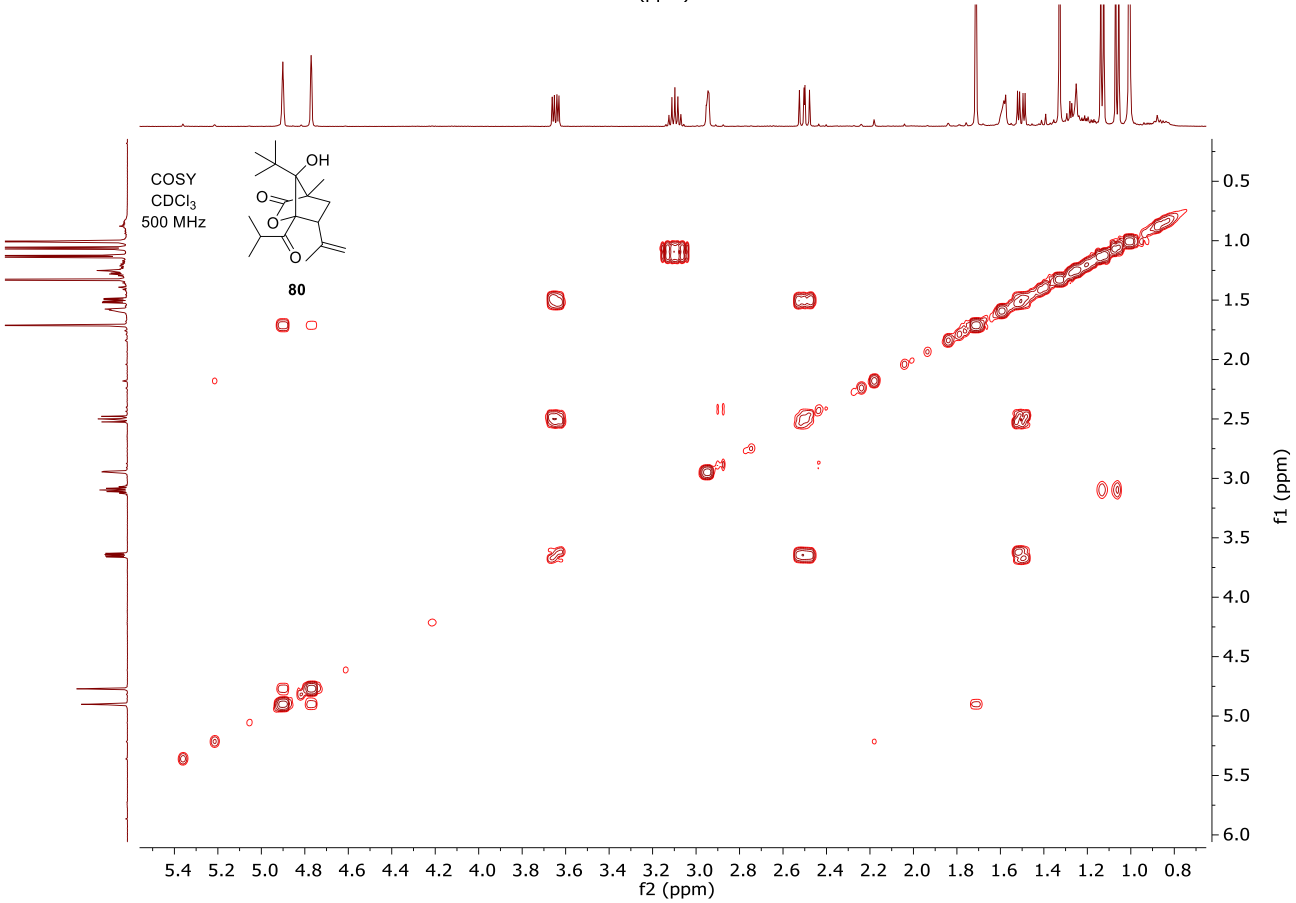

COSY
$CDCl_3$
500 MHz
OH
O
O
O
80
f2 (ppm)
f1 (ppm)

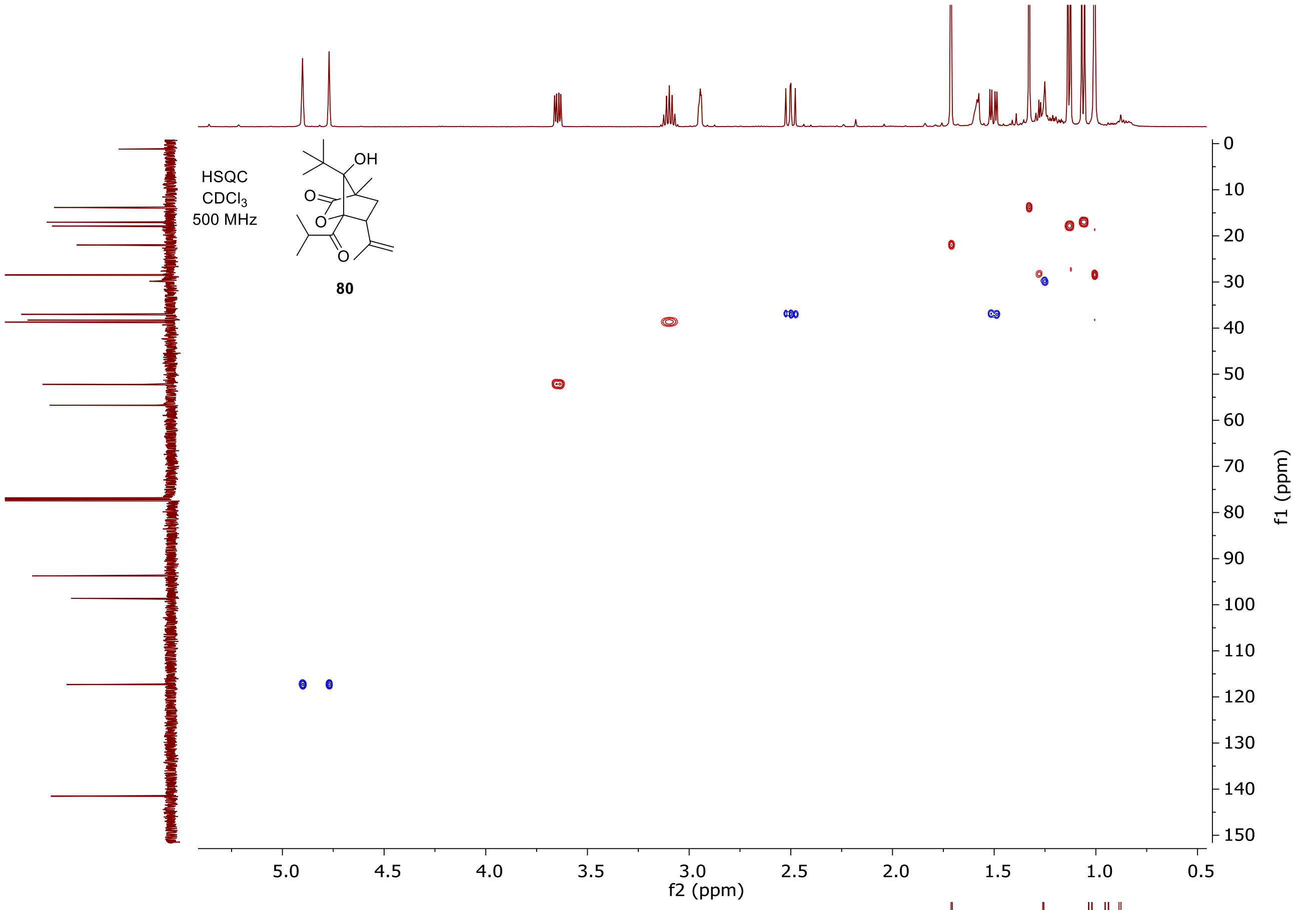
HSQC
CDCl3
500 MHz
OH
O
O
O
80
f2 (ppm)
f1 (ppm)

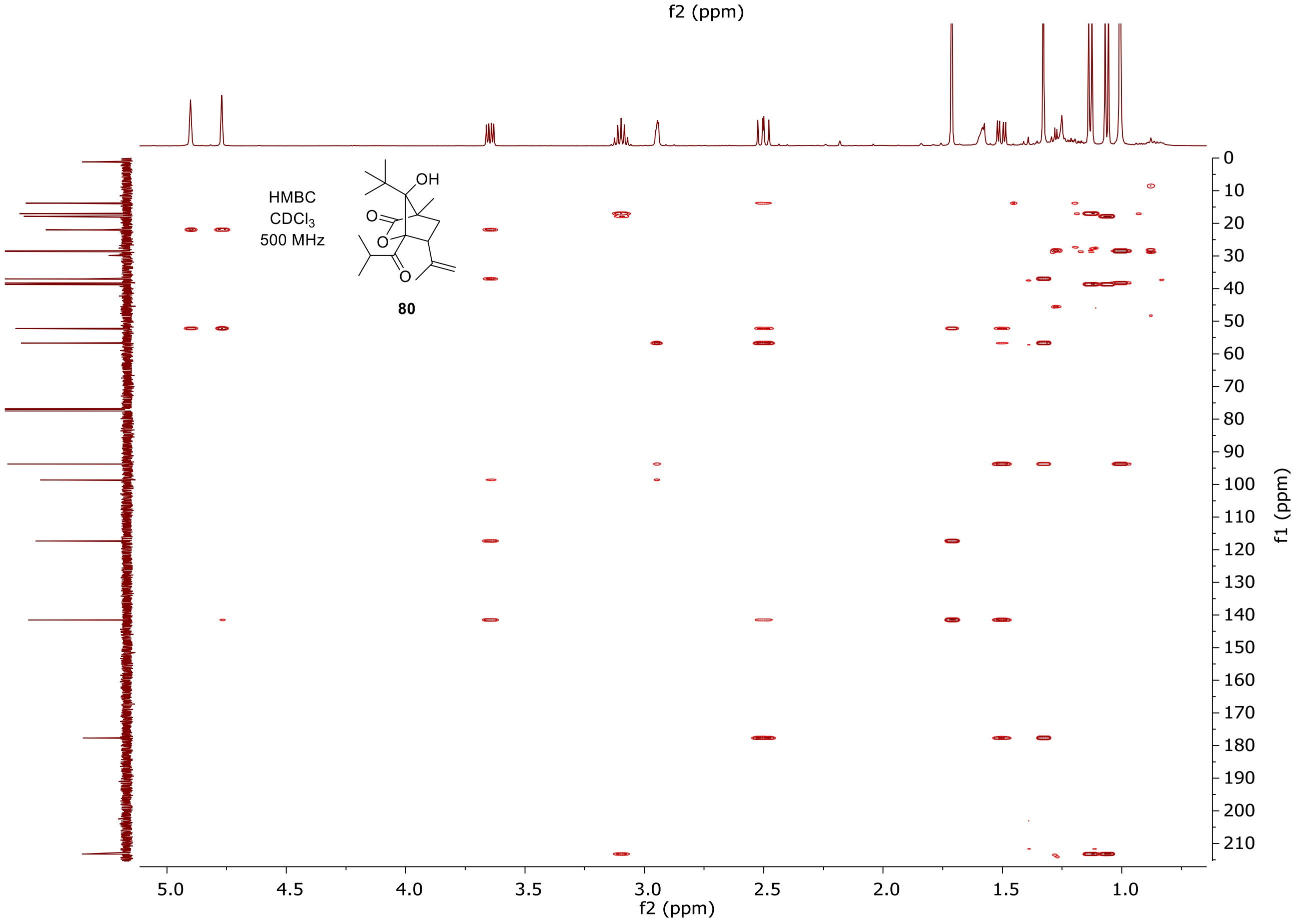
HMBC
CDCl3
500 MHz
OH
O
O
O
80
f2 (ppm)
f1 (ppm)

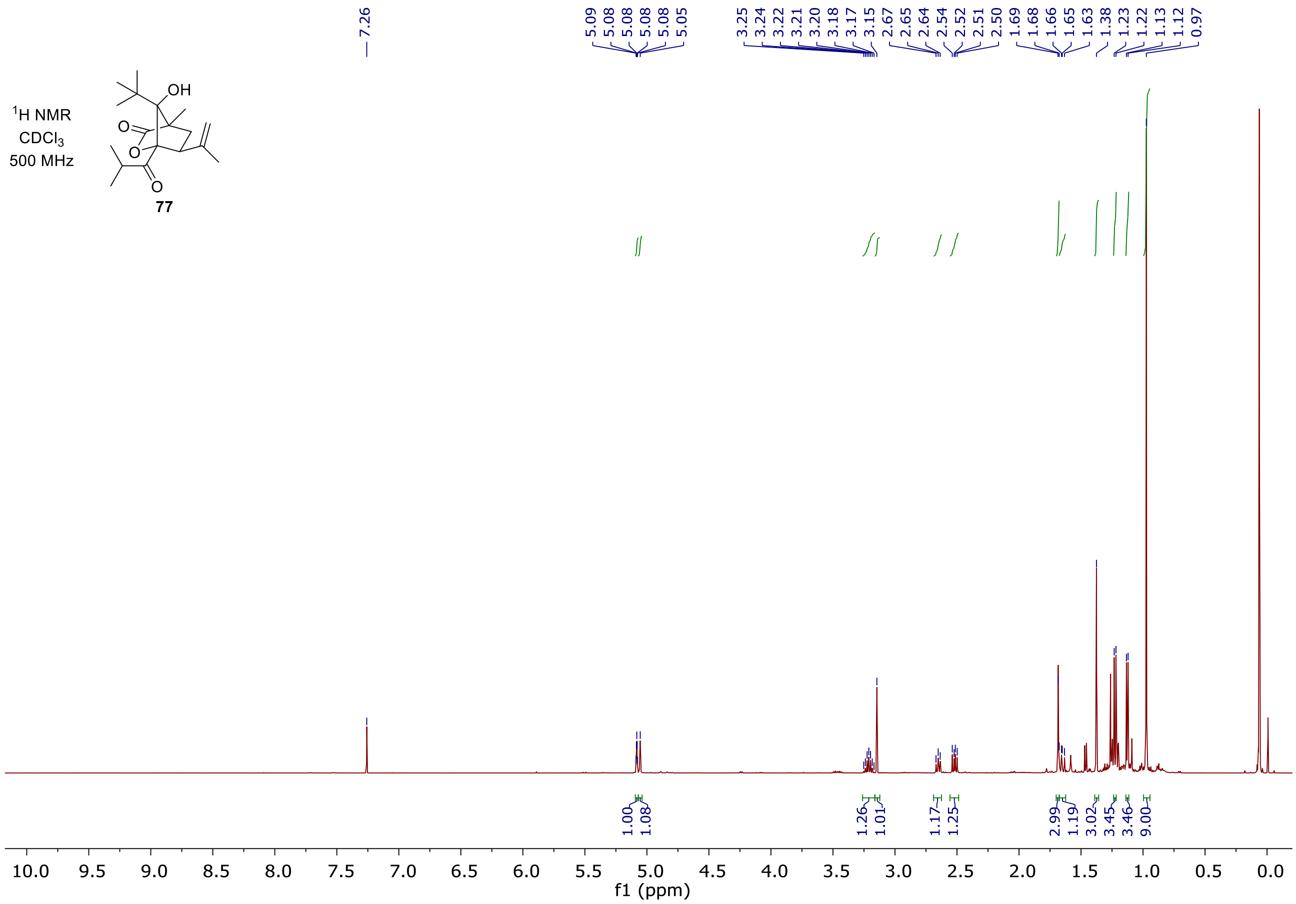
1H NMR
CDCl3
500 MHz
OH
O
77
f1 (ppm)

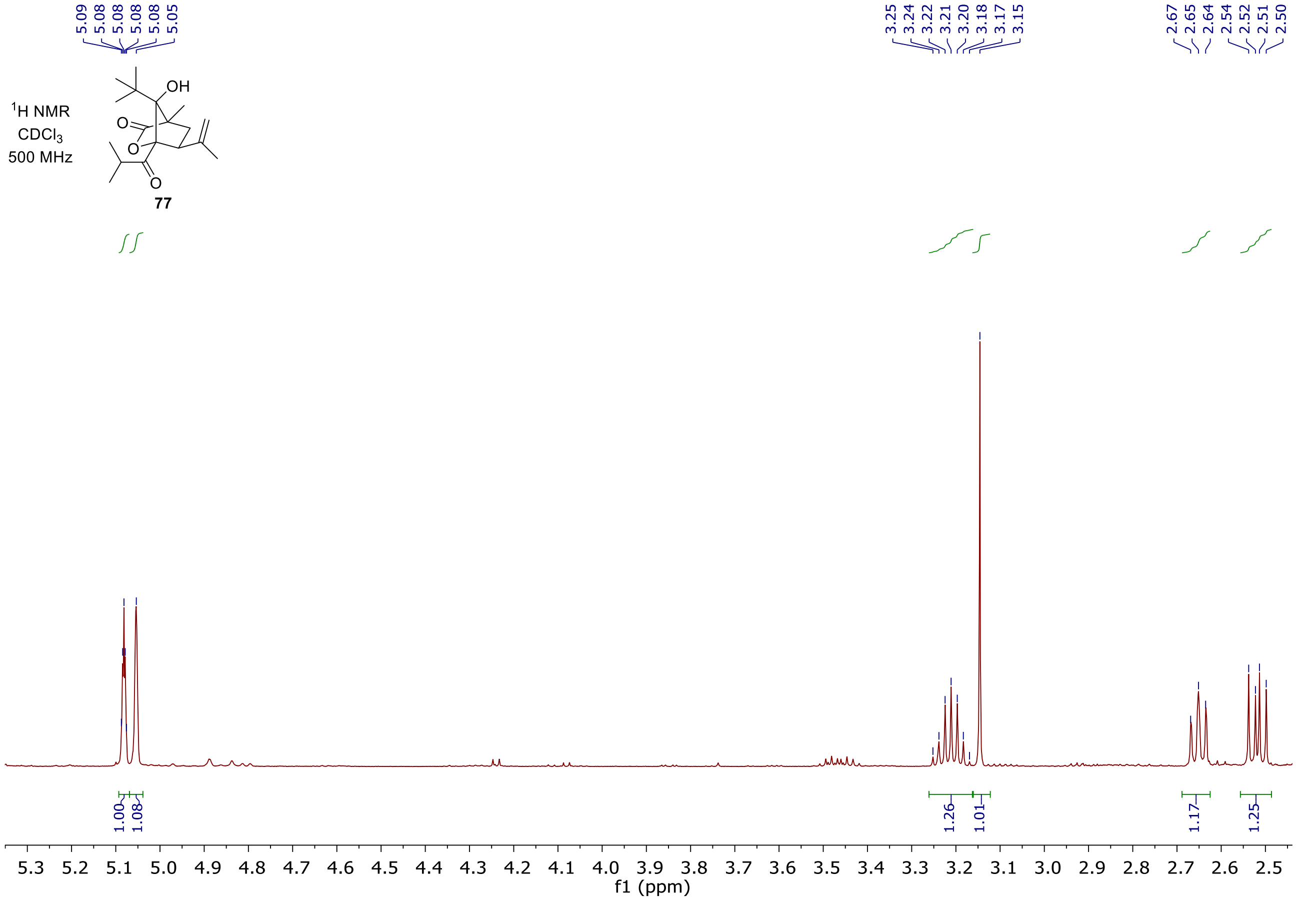
1H NMR
CDCl3
500 MHz
OH
O
77
f1 (ppm)

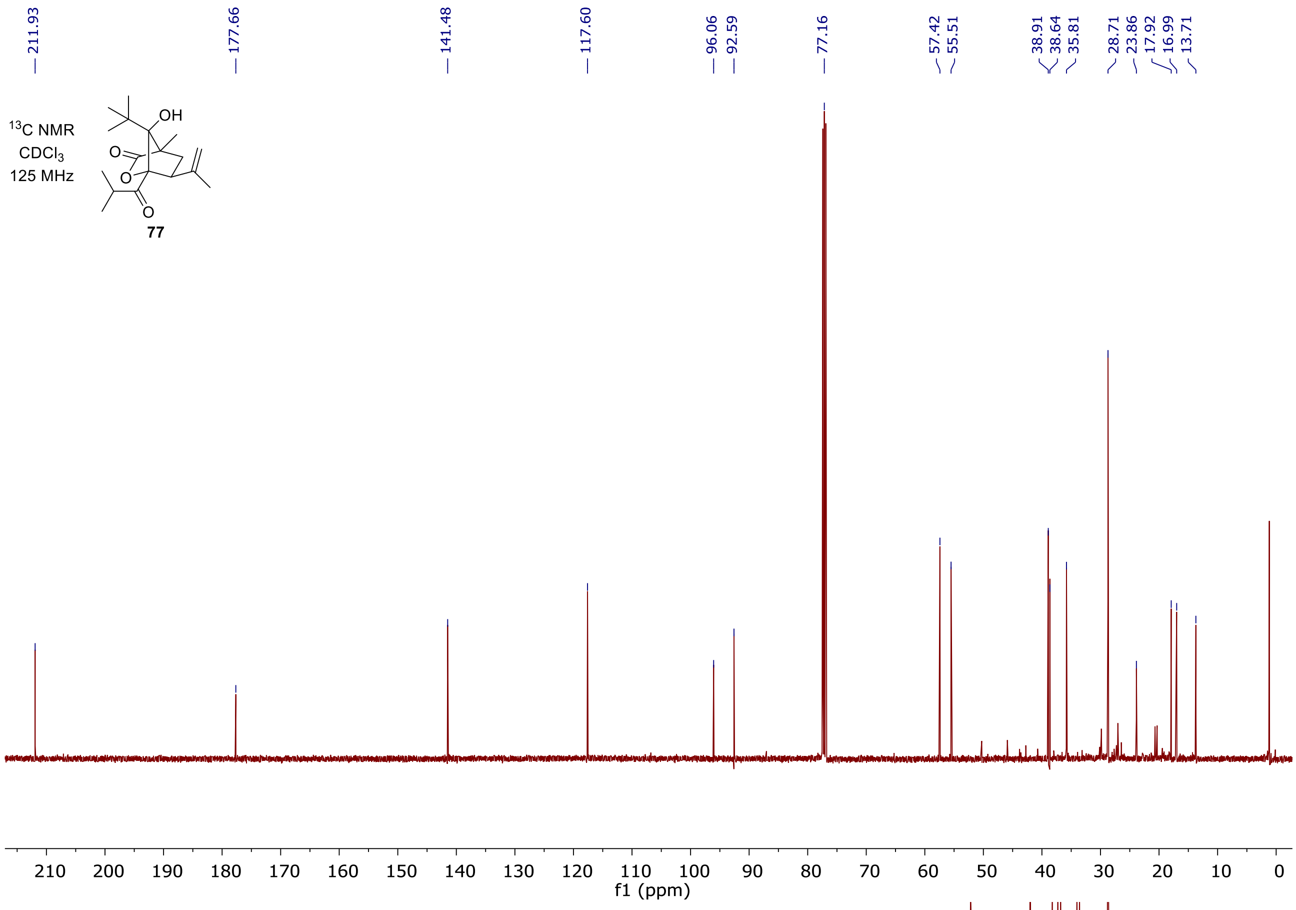

211.93
177.66
141.48
117.60
96.06
92.59
77.16
57.42
55.51
38.91
38.64
35.81
28.71
23.86
17.92
16.99
13.71
^{13}C NMR
CDCl3
125 MHz
OH
O
O
O
77
f1 (ppm)

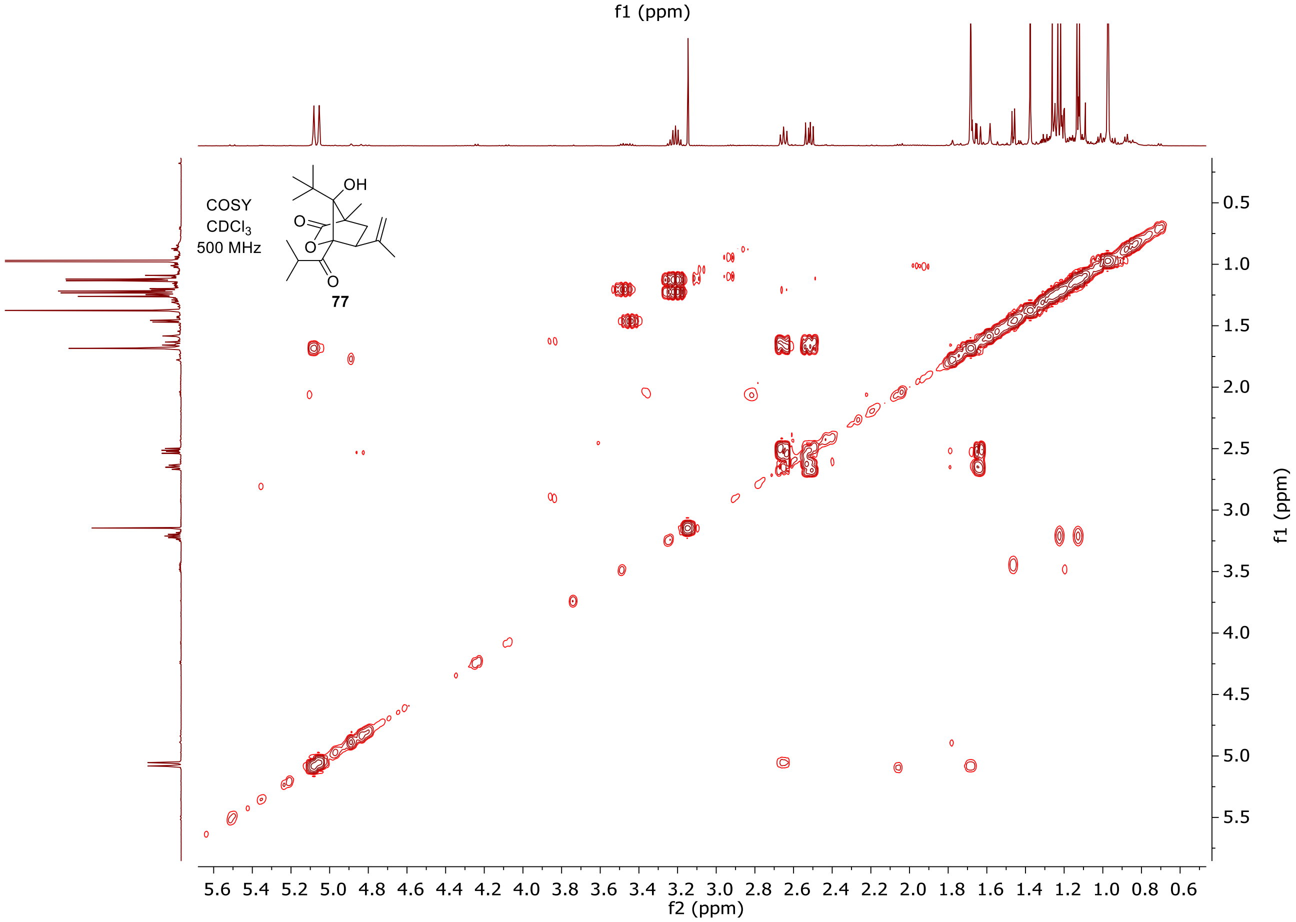

COSY
CDCl3
500 MHz
OH
O
O
O
77
f2 (ppm)
f1 (ppm)

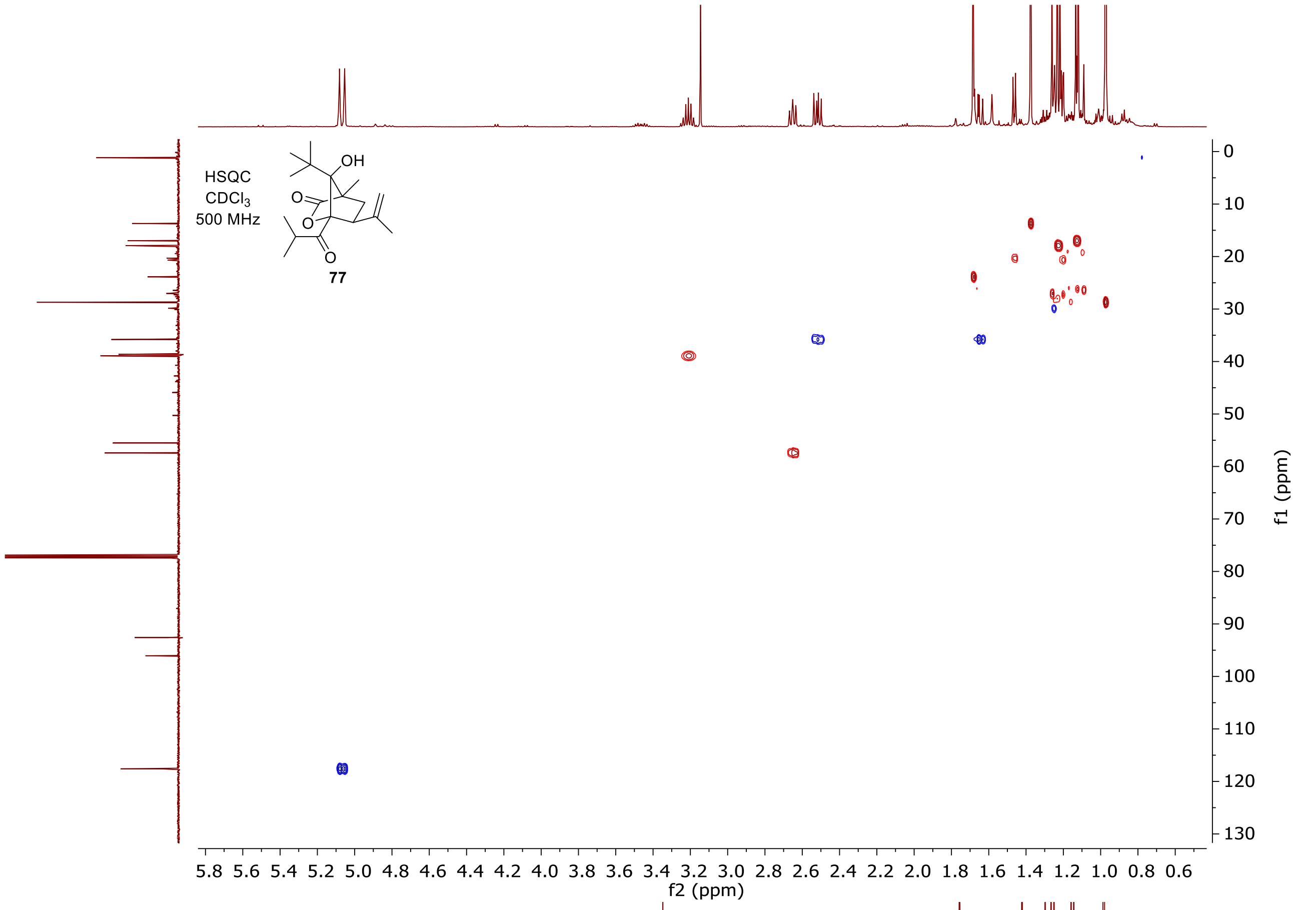
HSQC
CDCl3
500 MHz
OH
O
O
O
77
f2 (ppm)
f1 (ppm)

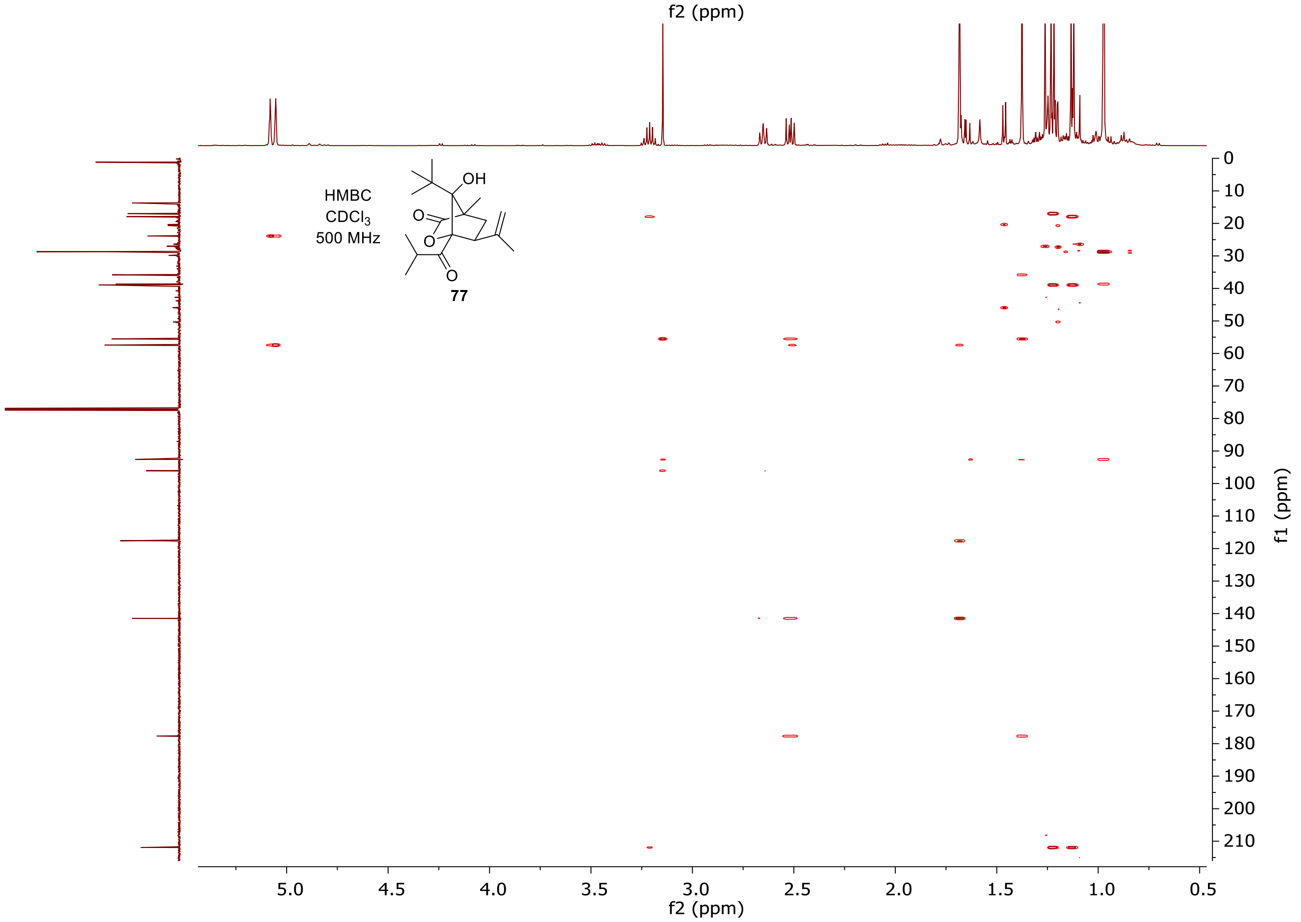
HMBC
CDCl3
500 MHz
OH
O
O
O
77
f2 (ppm)
f1 (ppm)

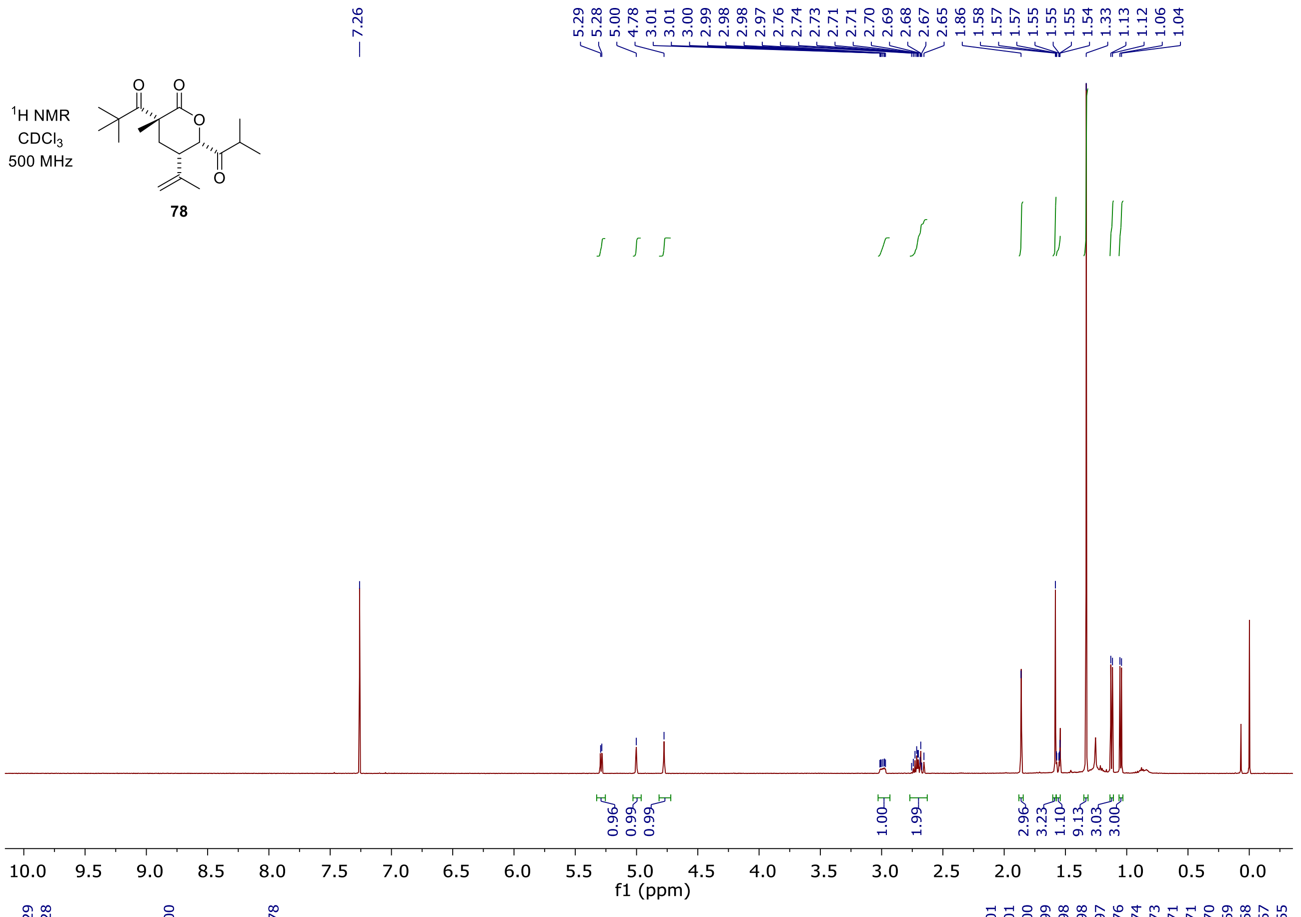
1H NMR
CDCl3
500 MHz
78
7.26
5.29 5.28 5.00 4.78 3.01 3.00 2.99 2.98 2.98 2.97 2.76 2.74 2.73 2.71 2.71 2.70 2.69 2.68 2.67 2.65 1.86 1.58 1.57 1.57 1.55 1.55 1.54 1.33 1.13 1.12 1.06 1.04
0.96 0.99 0.99 1.00 1.99 2.96 3.23 1.10 9.13 3.03 3.00
f1 (ppm)

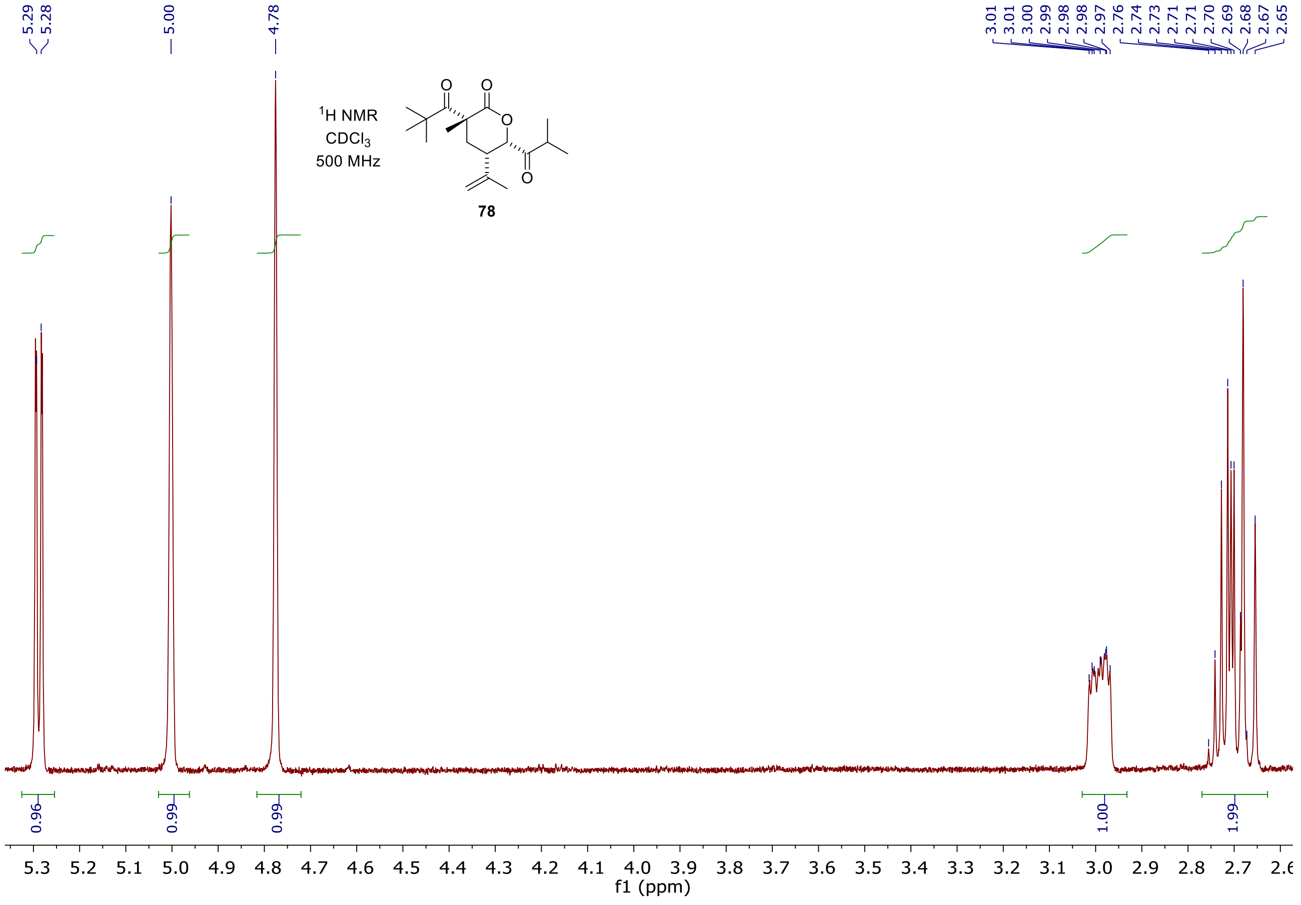
1H NMR
CDCl3
500 MHz
78
5.29 5.28
5.00
4.78
3.01 3.01 3.00 2.99 2.98 2.98 2.97 2.76 2.74 2.73 2.71 2.71 2.70 2.69 2.68 2.67 2.65
0.96 0.99 0.99 1.00 1.99
f1 (ppm)

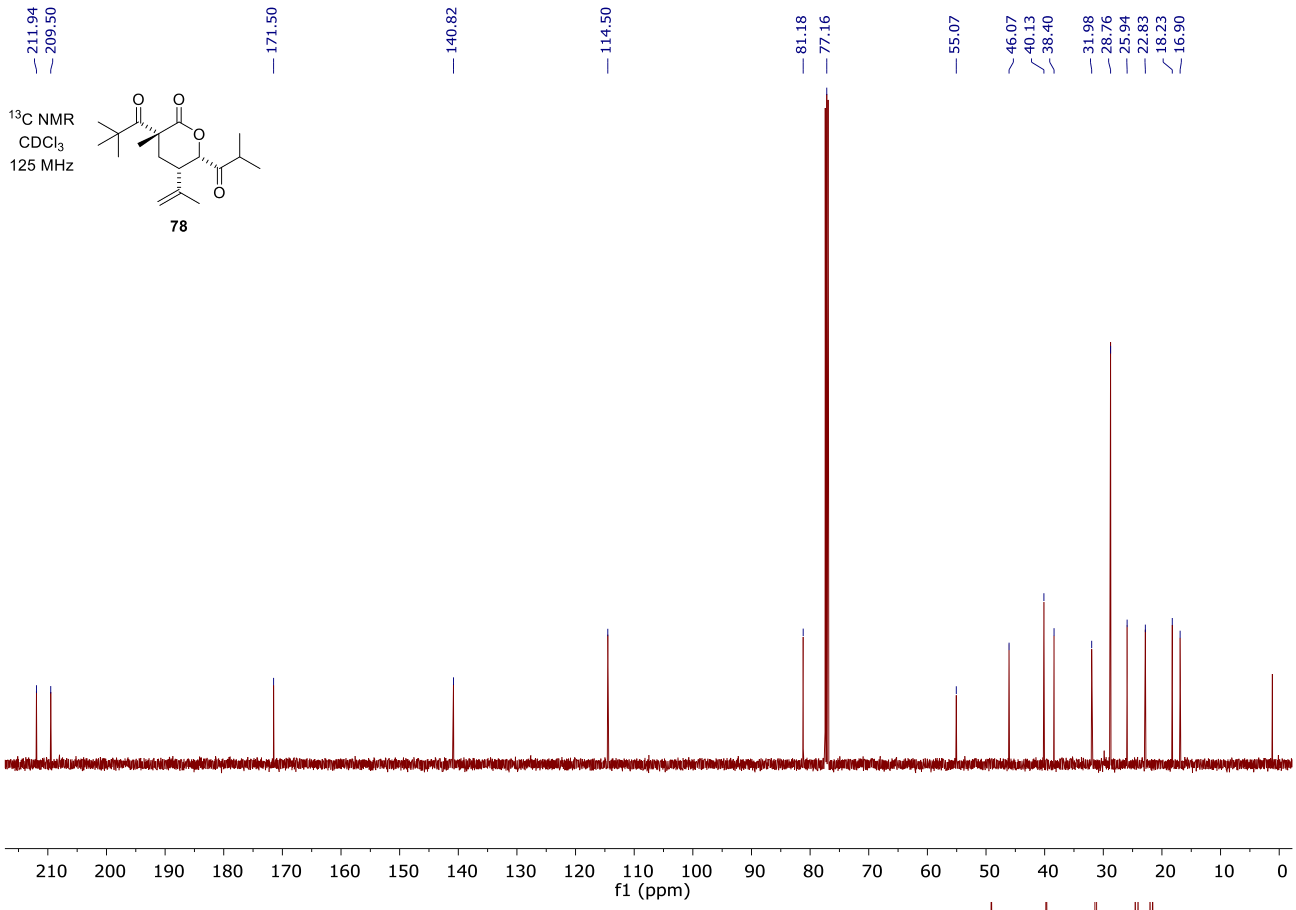
211.94
209.50
171.50
140.82
114.50
81.18
77.16
55.07
46.07
40.13
38.40
31.98
28.76
25.94
22.83
18.23
16.90
13C NMR
CDCl3
125 MHz
78
f1 (ppm)

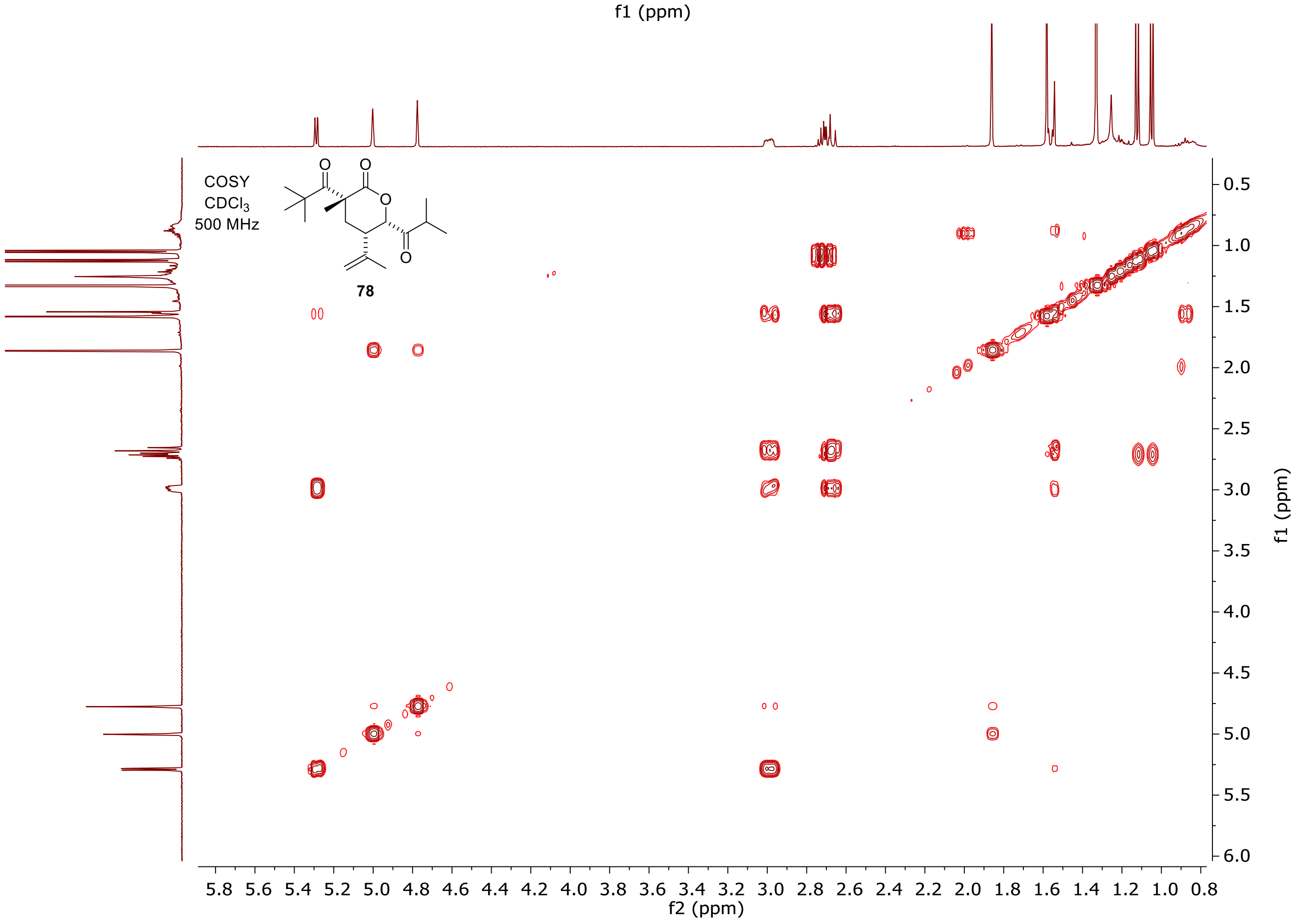
COSY
CDCl3
500 MHz
78
f2 (ppm)
f1 (ppm)

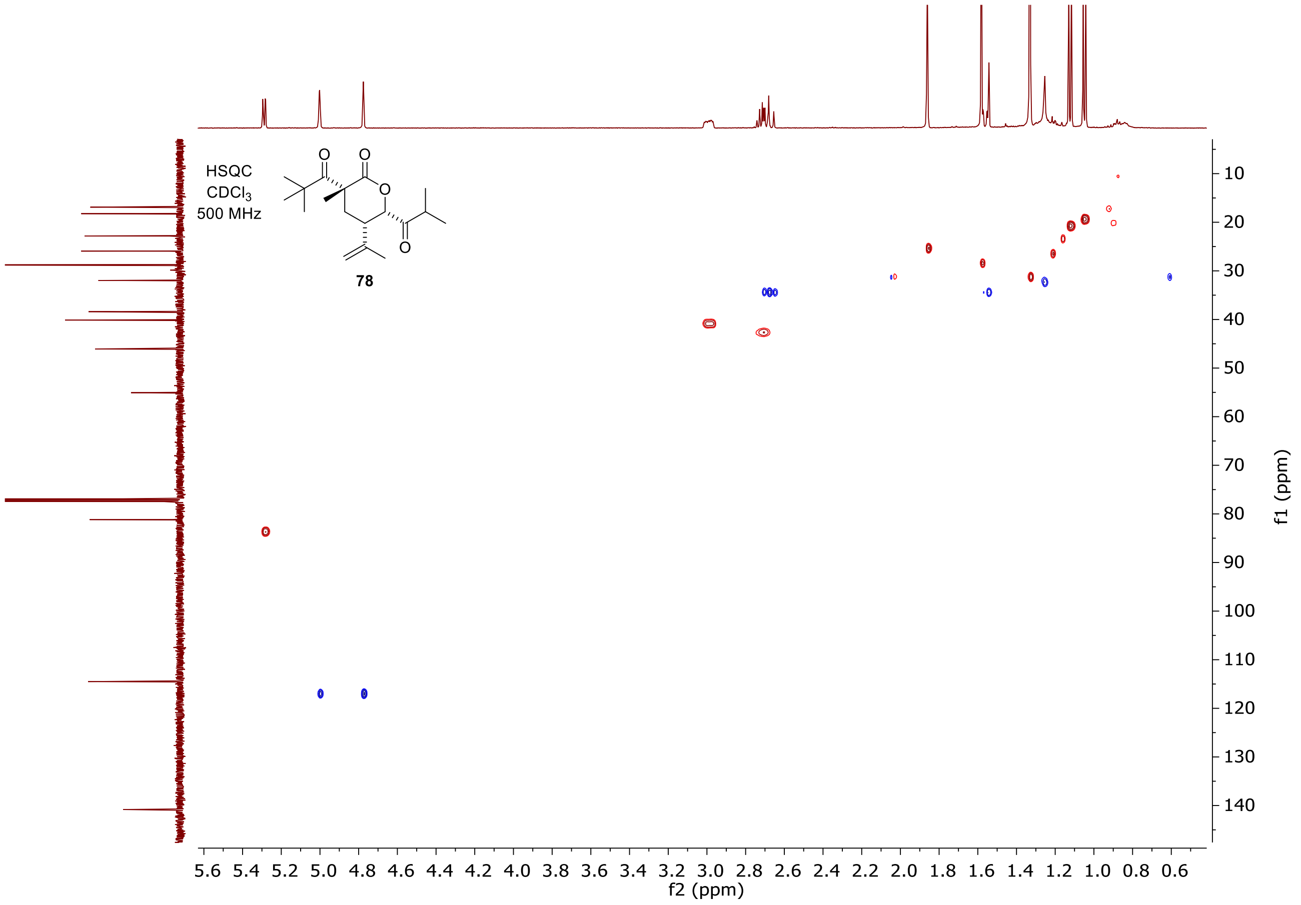

HSQC
CDCl3
500 MHz
78
f2 (ppm)
f1 (ppm)

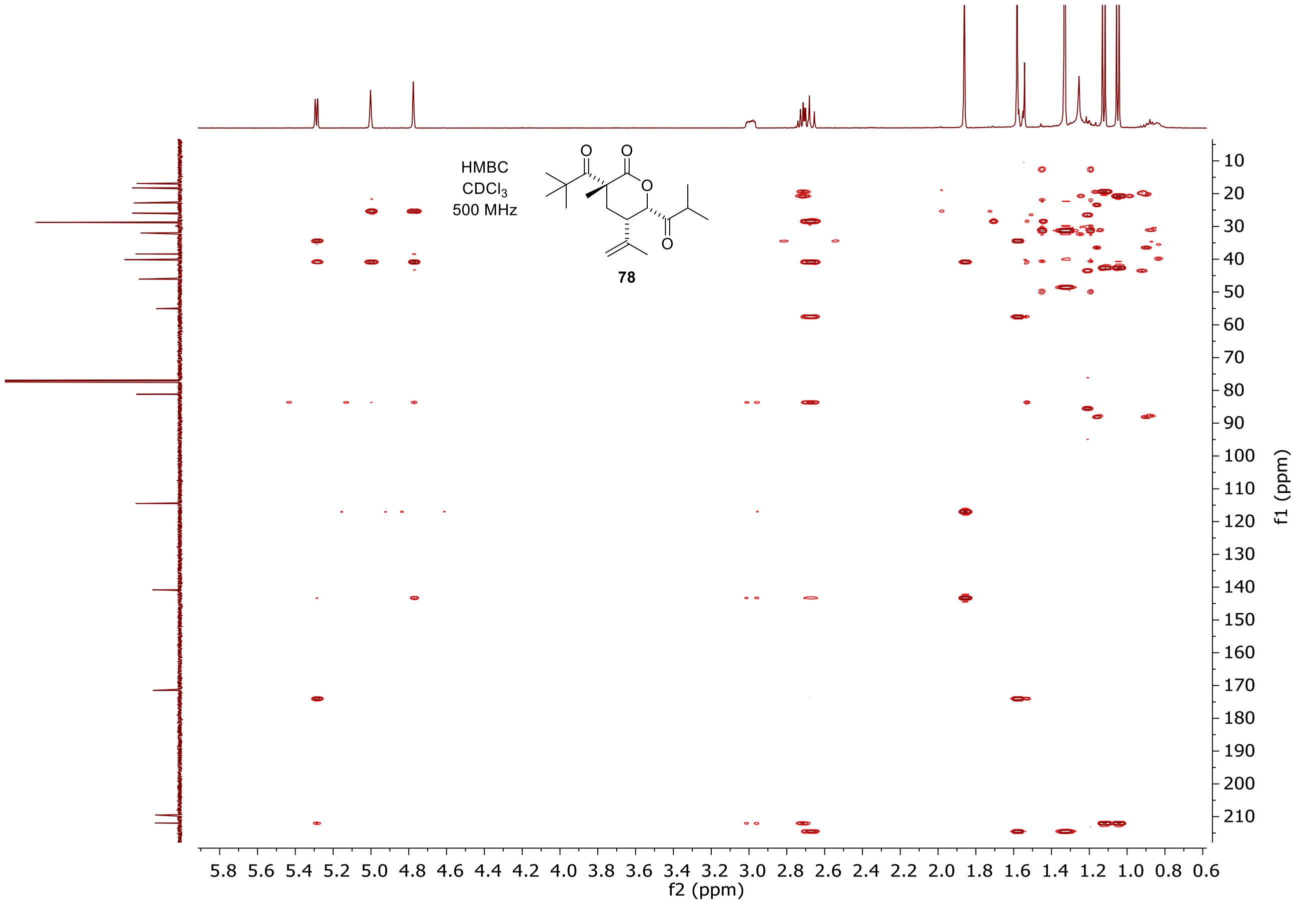

HMBC
CDCl3
500 MHz
78
f2 (ppm)
f1 (ppm)

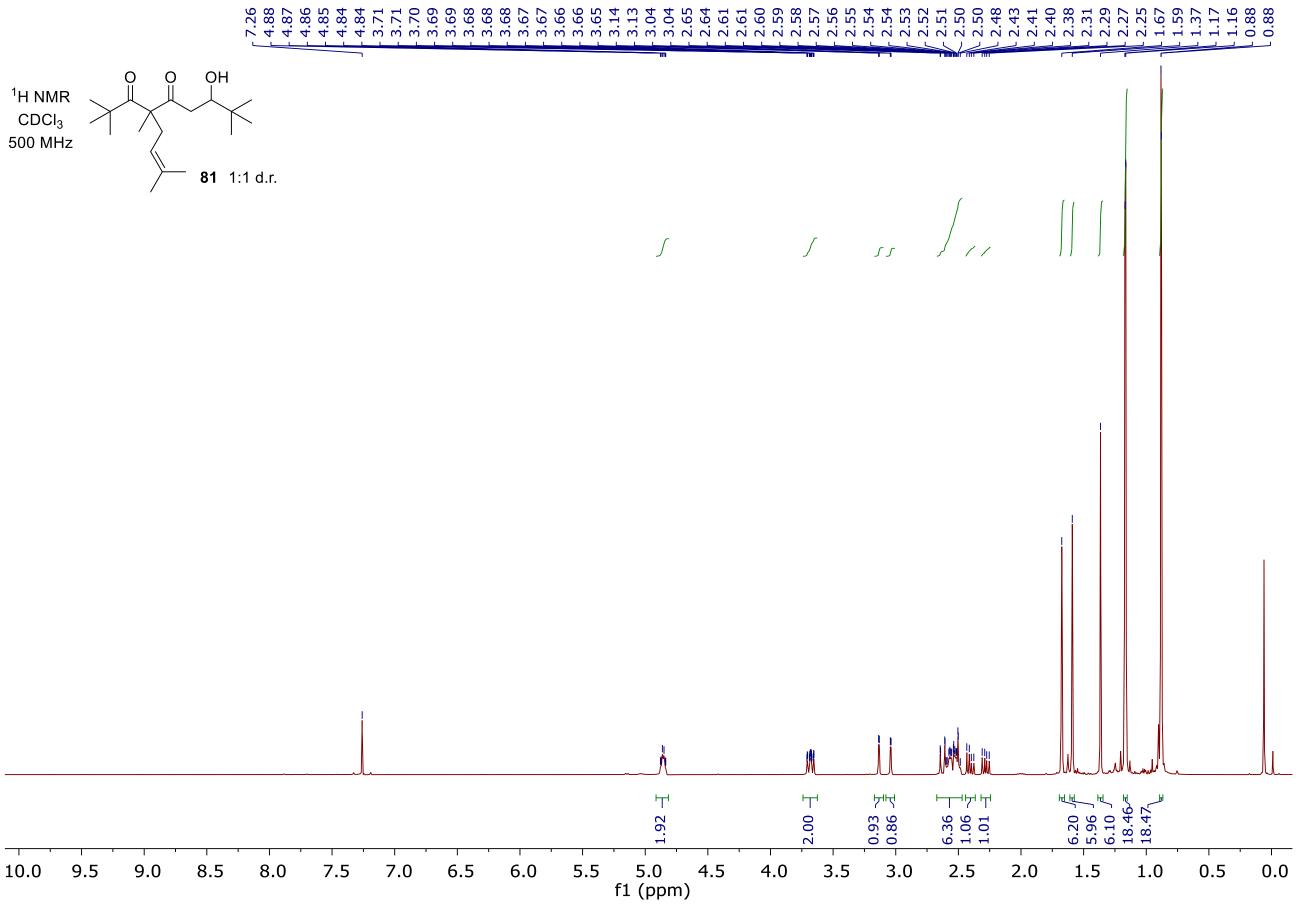

7.26 4.88 4.87 4.86 4.85 4.84 4.84 3.71 3.71 3.70 3.69 3.69 3.68 3.68 3.68 3.67 3.67 3.66 3.66 3.65 3.14 3.13 3.04 3.04 2.65 2.64 2.61 2.61 2.60 2.59 2.58 2.57 2.56 2.55 2.54 2.54 2.53 2.52 2.51 2.50 2.50 2.48 2.43 2.41 2.40 2.38 2.31 2.29 2.27 2.25 1.67 1.59 1.37 1.17 1.16 0.88 0.88
^{1}H NMR
$CDCl_3$
500 MHz
O O OH
81 1:1 d.r.
1.92 2.00 0.93 0.86 6.36 1.06 1.01 6.20 5.96 6.10 18.46 18.47
10.0 9.5 9.0 8.5 8.0 7.5 7.0 6.5 6.0 5.5 5.0 4.5 4.0 3.5 3.0 2.5 2.0 1.5 1.0 0.5 0.0
f1 (ppm)

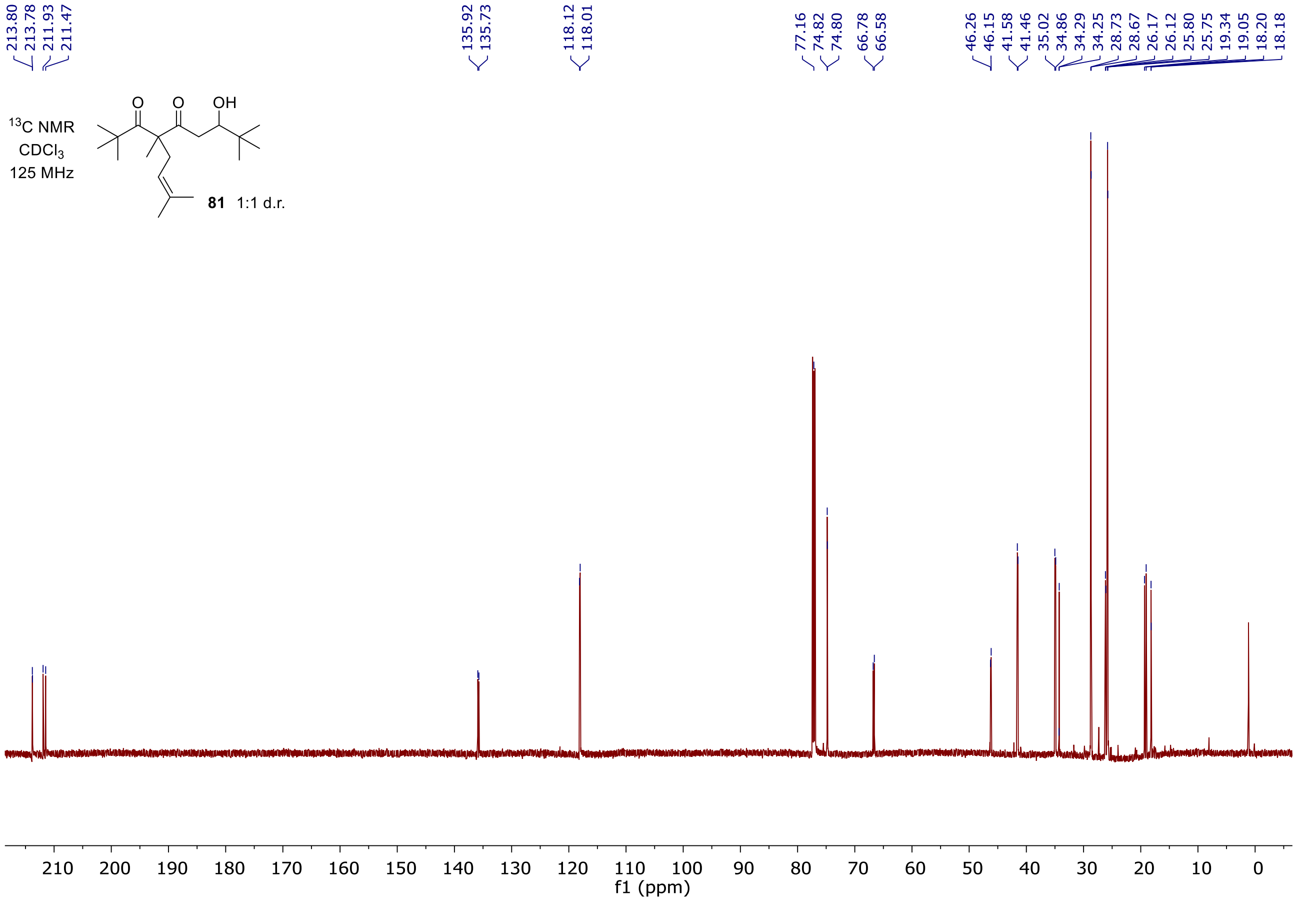

213.80 213.78 211.93 211.47 135.92 135.73 118.12 118.01 77.16 74.82 74.80 66.78 66.58 46.26 46.15 41.58 41.46 35.02 34.86 34.29 34.25 28.73 28.67 26.17 26.12 25.80 25.75 19.34 19.05 18.20 18.18
^{13}C NMR
$CDCl_3$
125 MHz
O O OH
81 1:1 d.r.
210 200 190 180 170 160 150 140 130 120 110 100 90 80 70 60 50 40 30 20 10 0
f1 (ppm)

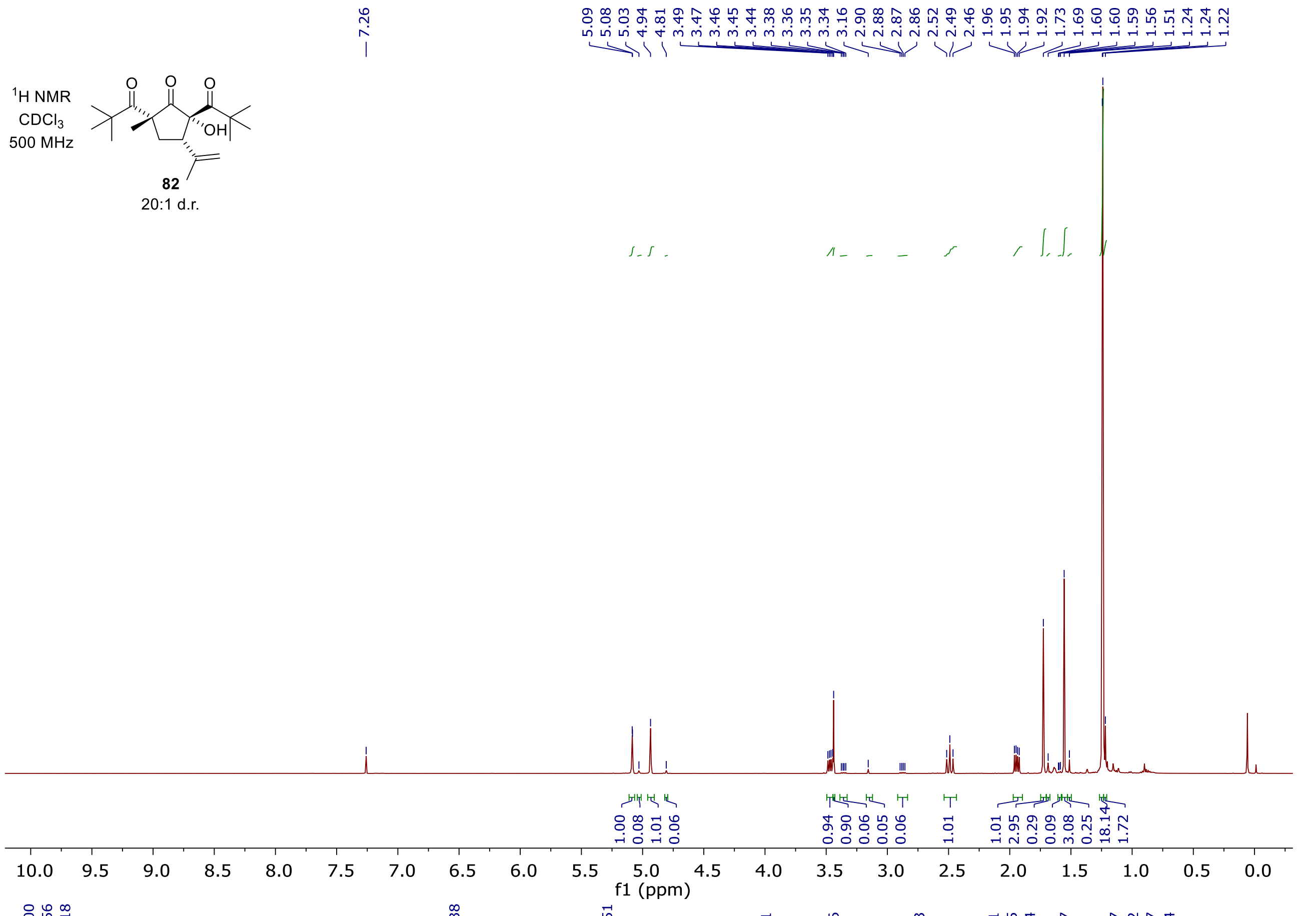

1H NMR
CDCl3
500 MHz
82
20:1 d.r.
f1 (ppm)

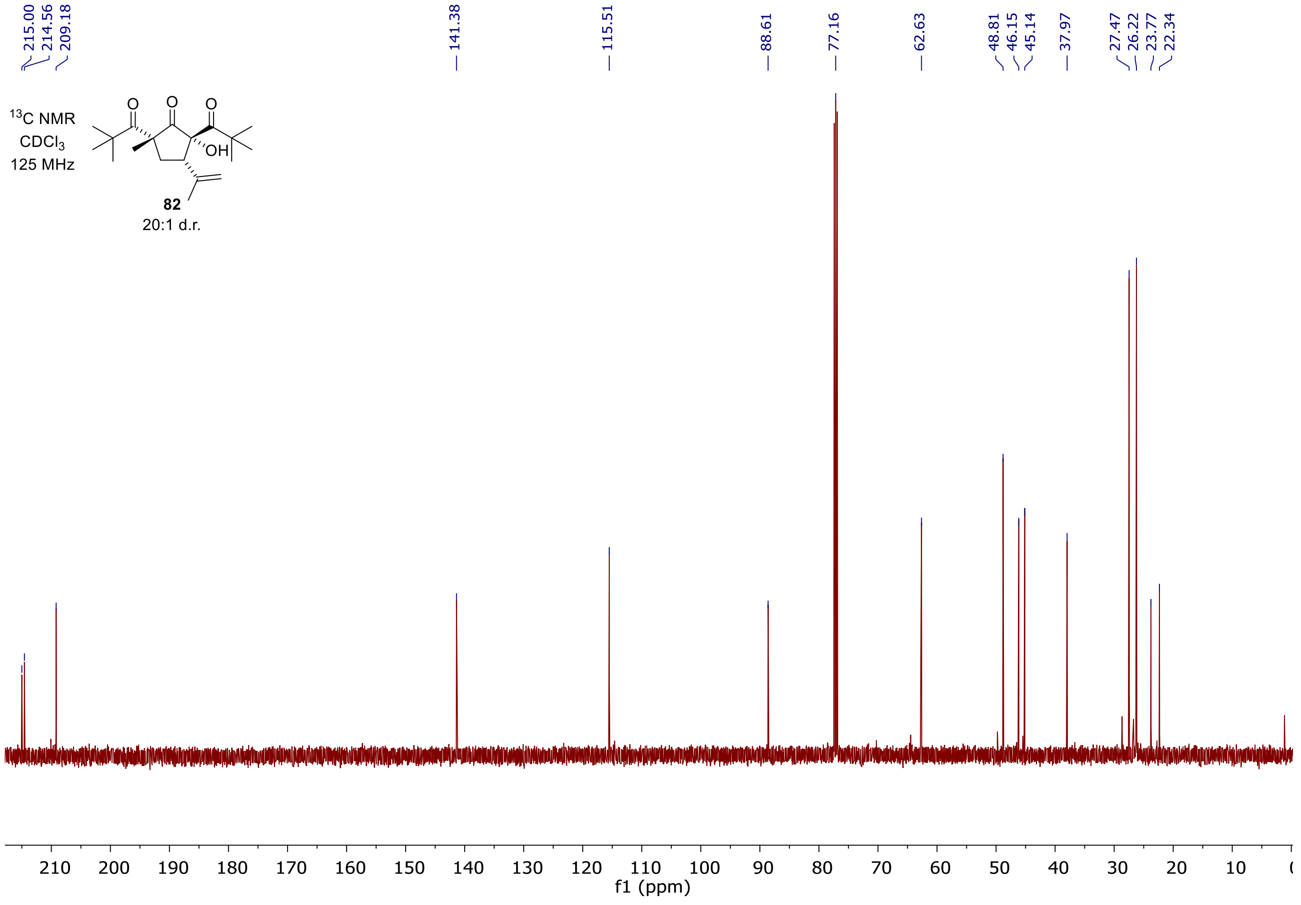

13C NMR
CDCl3
125 MHz
82
20:1 d.r.
f1 (ppm)

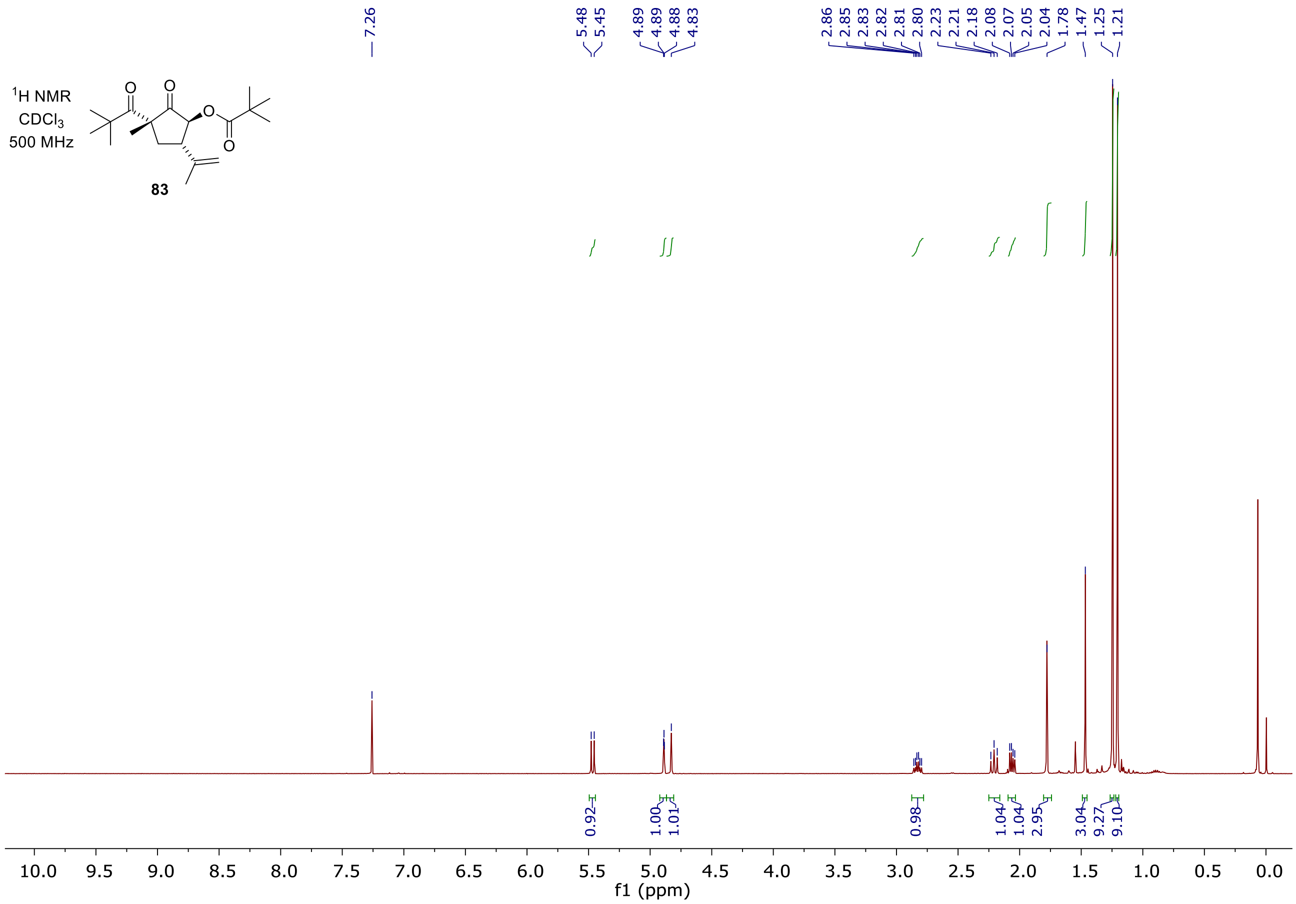
1H NMR
CDCl3
500 MHz
83
7.26
5.48 5.45
4.89 4.89 4.88 4.83
2.86 2.85 2.83 2.82 2.81 2.80 2.23 2.21 2.18 2.08 2.07 2.05 2.04 1.78 1.47 1.25 1.21
0.92 1.00 1.01 0.98 1.04 1.04 2.95 3.04 9.27 9.10
10.0 9.5 9.0 8.5 8.0 7.5 7.0 6.5 6.0 5.5 5.0 4.5 4.0 3.5 3.0 2.5 2.0 1.5 1.0 0.5 0.0
f1 (ppm)

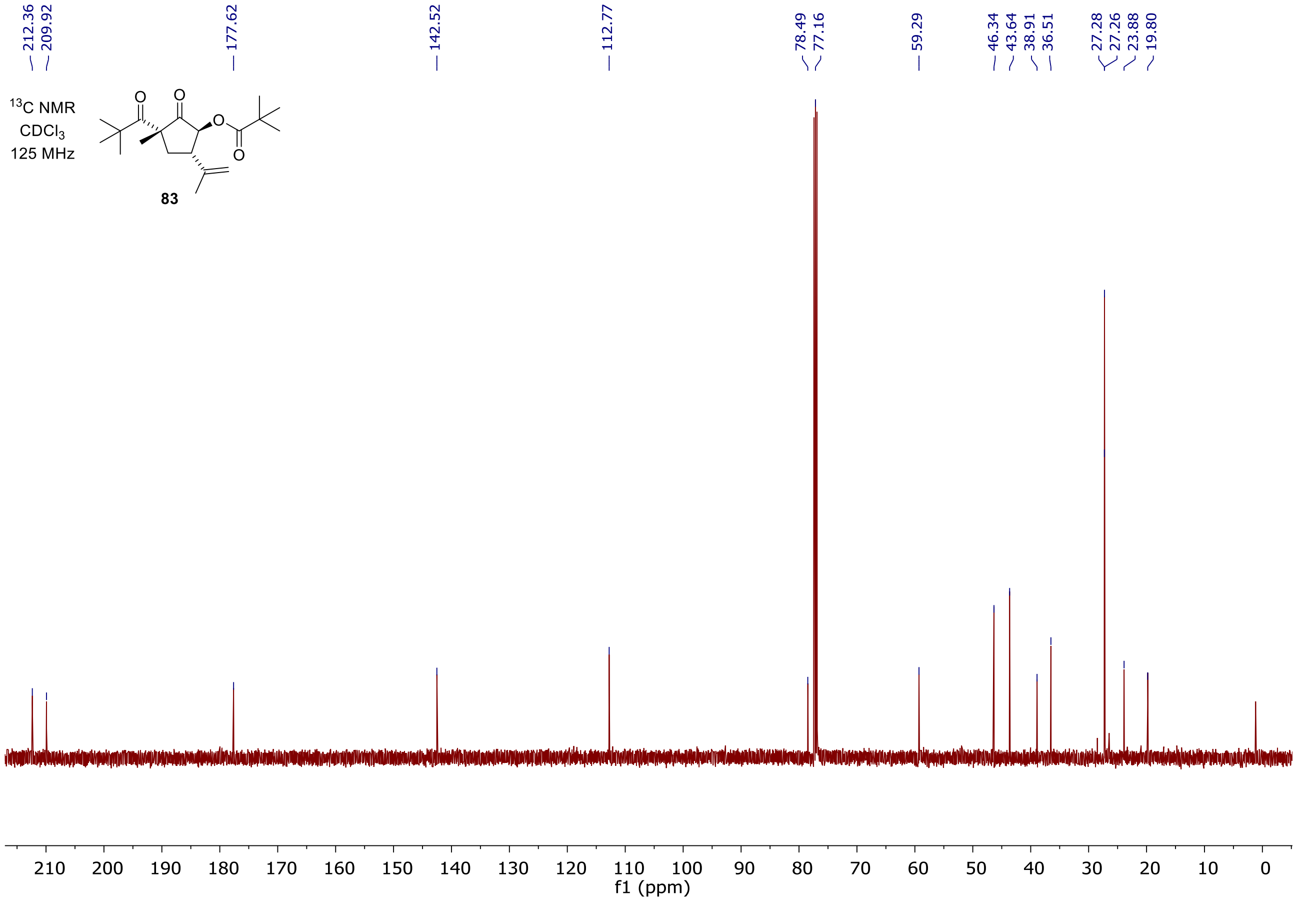
13C NMR
CDCl3
125 MHz
83
212.36 209.92
177.62
142.52
112.77
78.49 77.16
59.29
46.34 43.64 38.91 36.51
27.28 27.26 23.88 19.80
210 200 190 180 170 160 150 140 130 120 110 100 90 80 70 60 50 40 30 20 10 0
f1 (ppm)

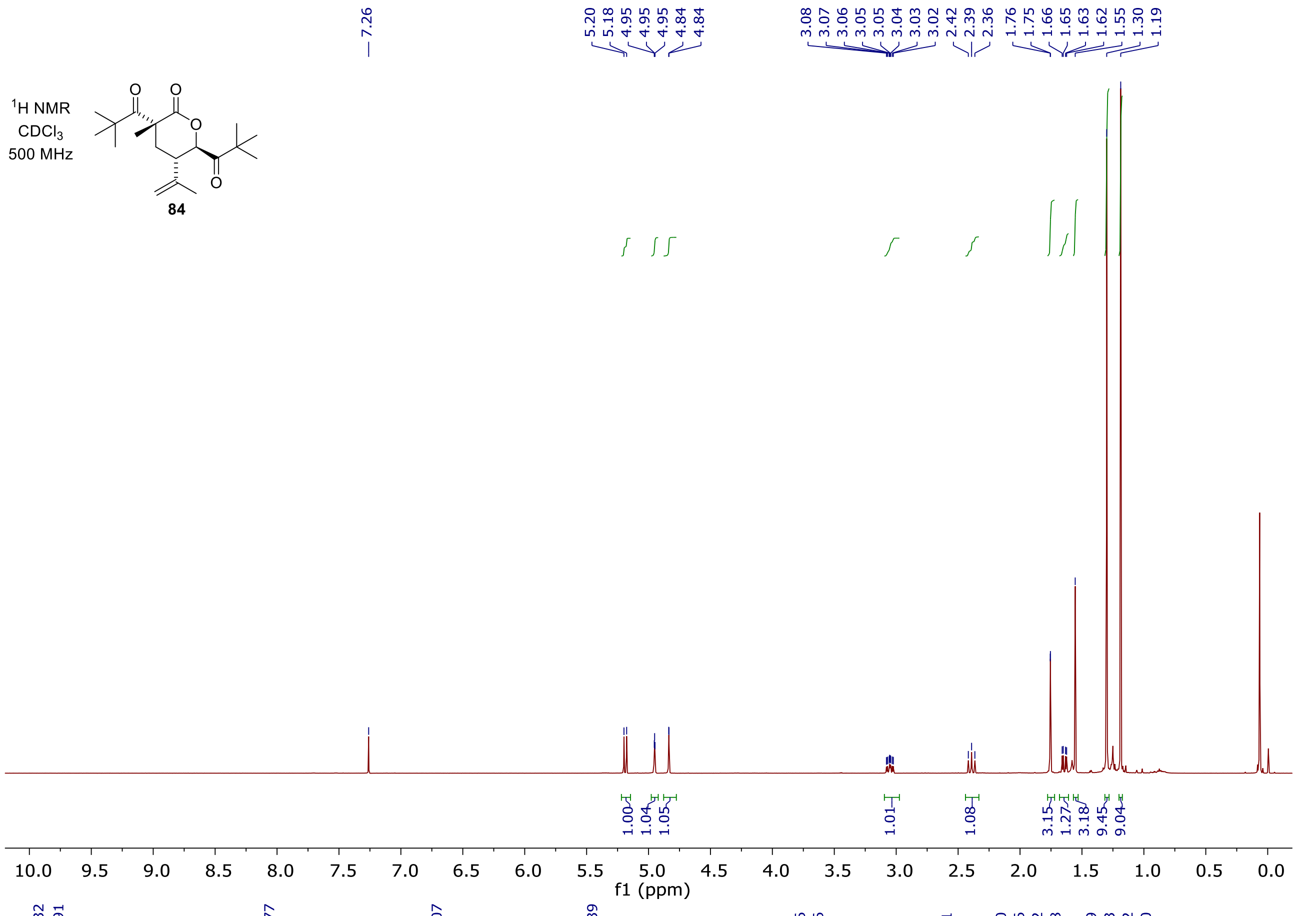

^{1}H NMR
CDCl$_3$
500 MHz
84
7.26
5.20 5.18 4.95 4.95 4.95 4.84 4.84
3.08 3.07 3.06 3.05 3.05 3.04 3.03 3.02 2.42 2.39 2.36 1.76 1.75 1.66 1.65 1.63 1.62 1.55 1.30 1.19
1.00 1.04 1.05 1.01 1.08 3.15 1.27 3.18 9.45 9.04
10.0 9.5 9.0 8.5 8.0 7.5 7.0 6.5 6.0 5.5 5.0 4.5 4.0 3.5 3.0 2.5 2.0 1.5 1.0 0.5 0.0
f1 (ppm)

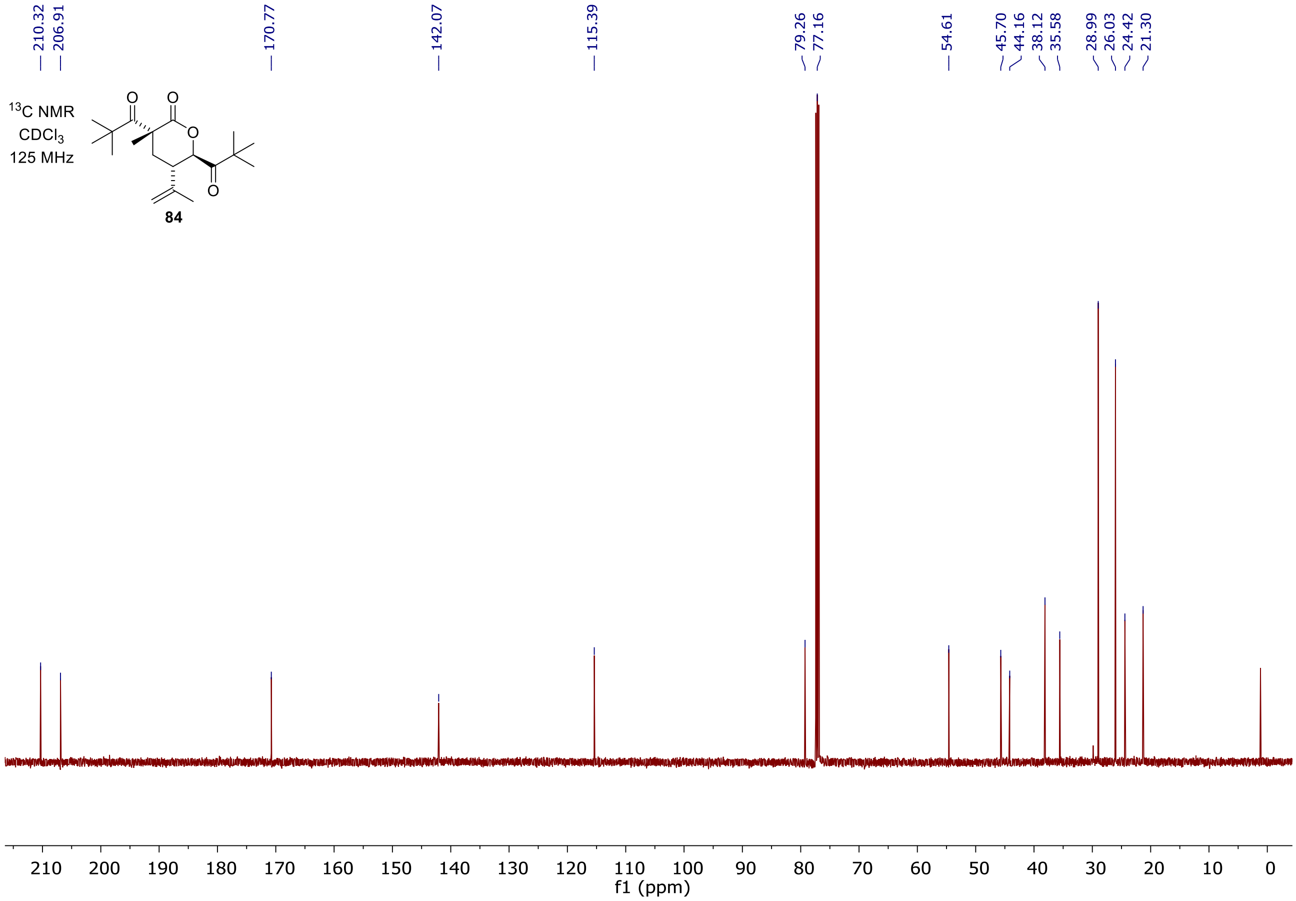

^{13}C NMR
CDCl$_3$
125 MHz
84
210.32 206.91 170.77 142.07 115.39 79.26 77.16 54.61 45.70 44.16 38.12 35.58 28.99 26.03 24.42 21.30
210 200 190 180 170 160 150 140 130 120 110 100 90 80 70 60 50 40 30 20 10 0
f1 (ppm)

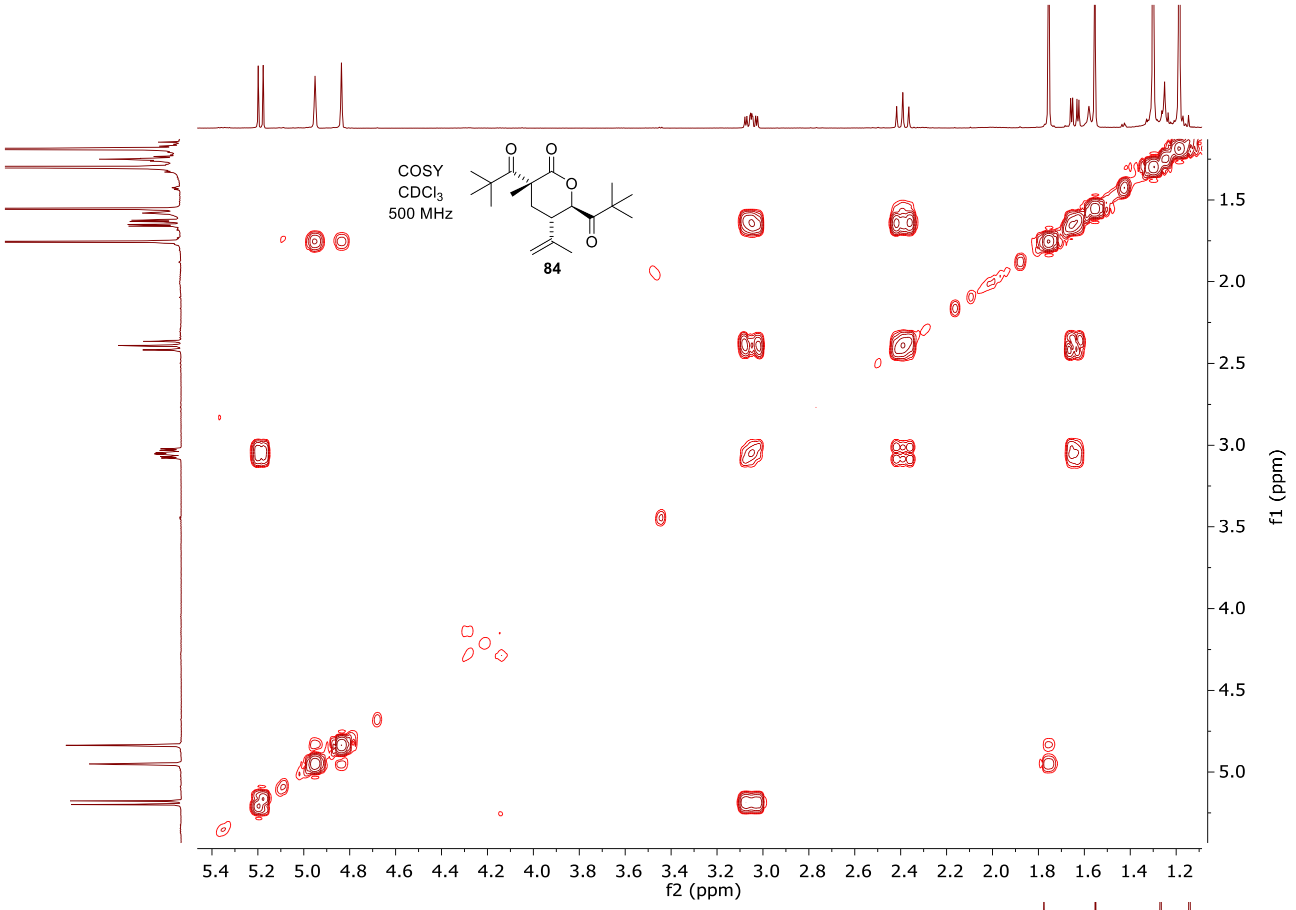
COSY
CDCl3
500 MHz
84
f2 (ppm)
f1 (ppm)

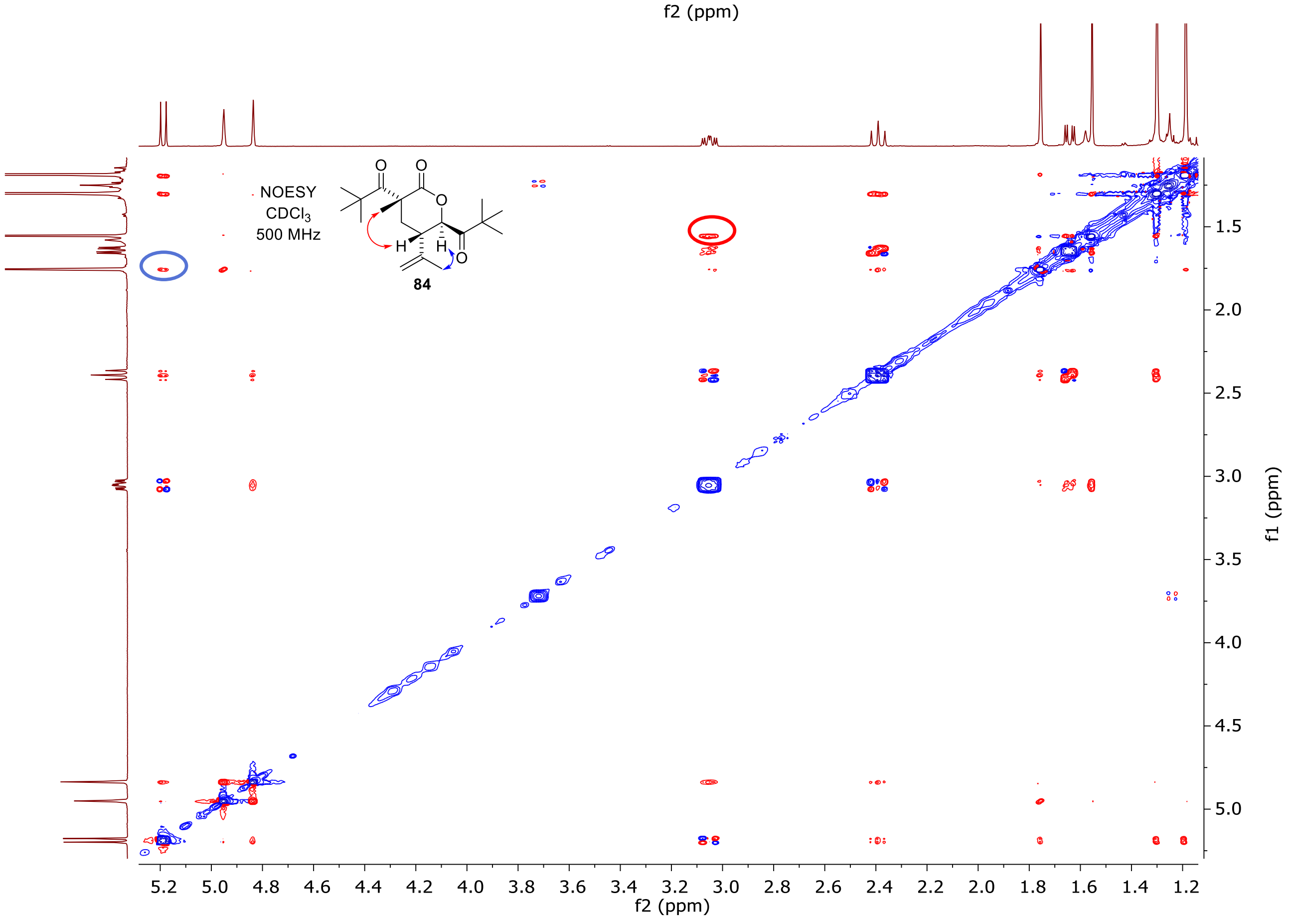
NOESY
CDCl3
500 MHz
84
f2 (ppm)
f1 (ppm)

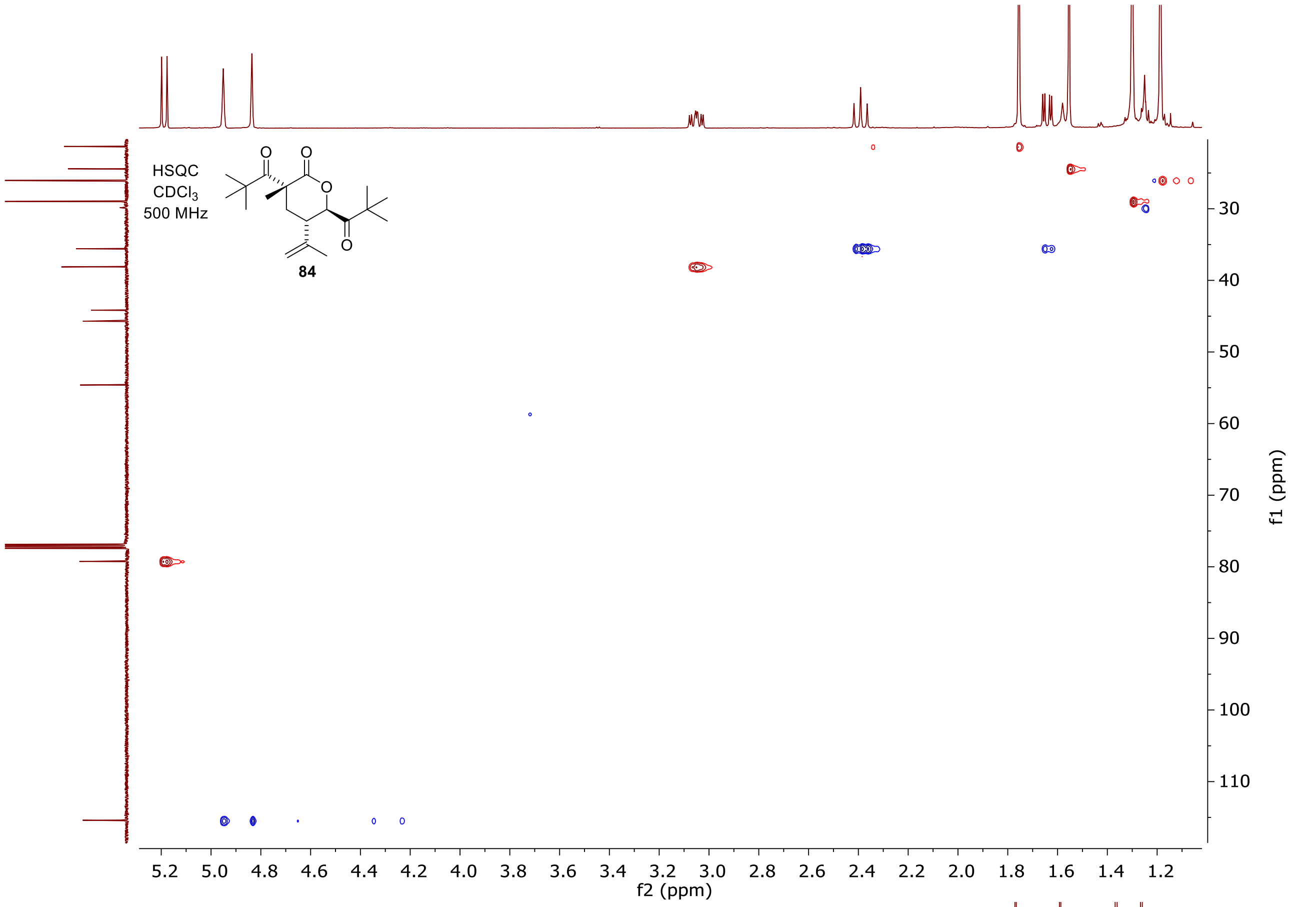
HSQC
CDCl3
500 MHz
84
f2 (ppm)
f1 (ppm)

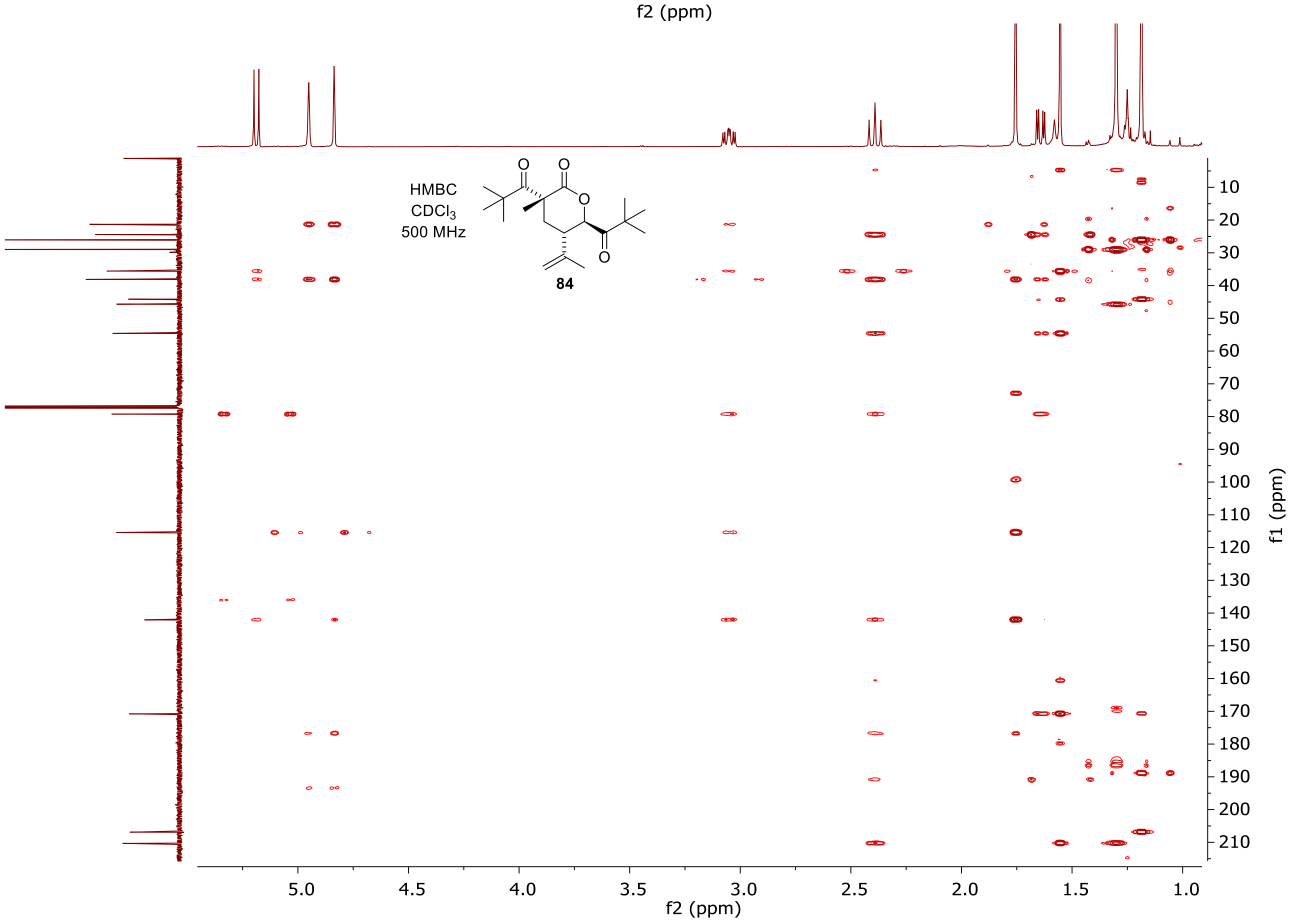
HMBC
CDCl3
500 MHz
84
f2 (ppm)
f1 (ppm)

^{1}H NMR
$CDCl_3$
500 MHz

85

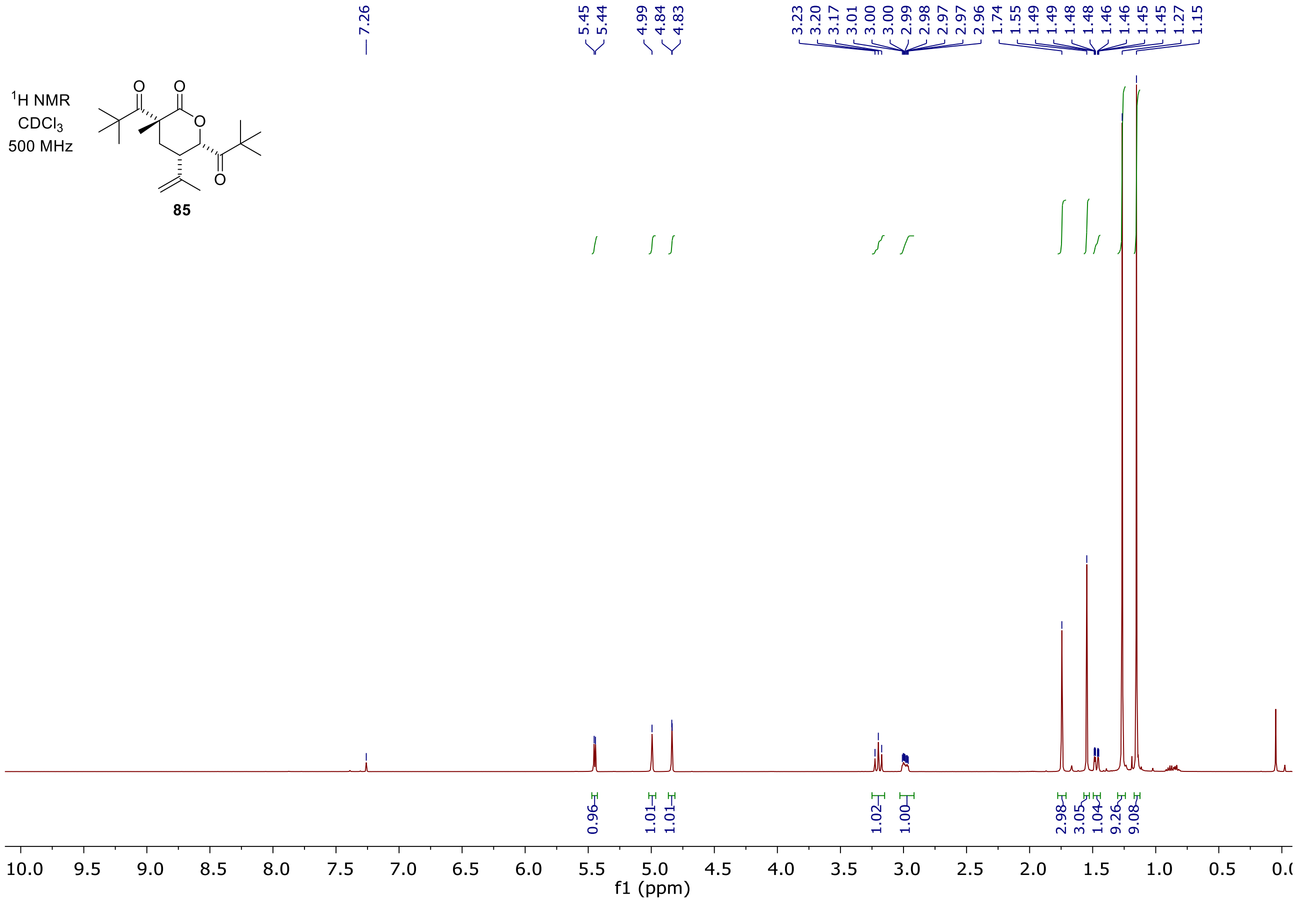

^{13}C NMR
$CDCl_3$
125 MHz

85

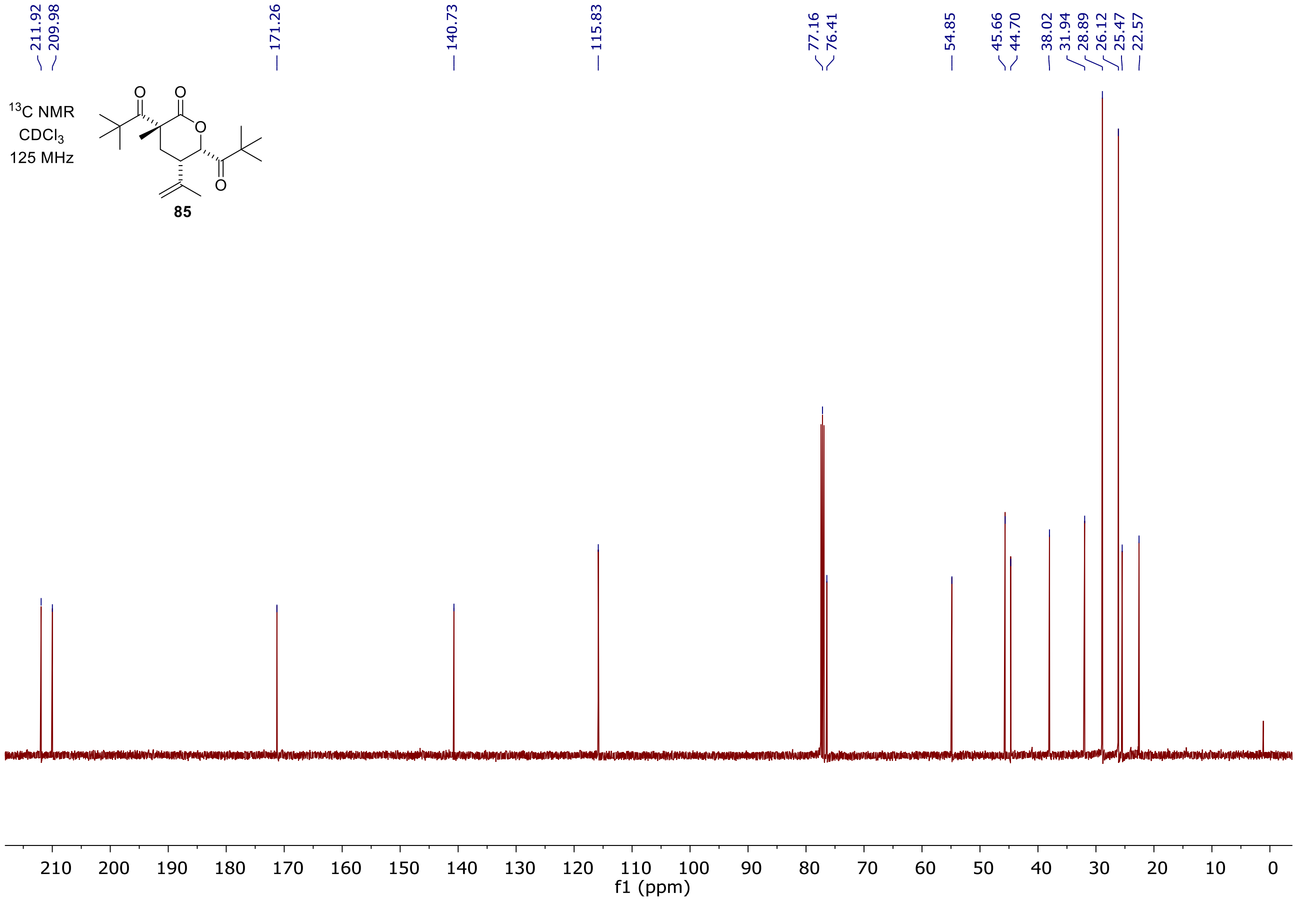

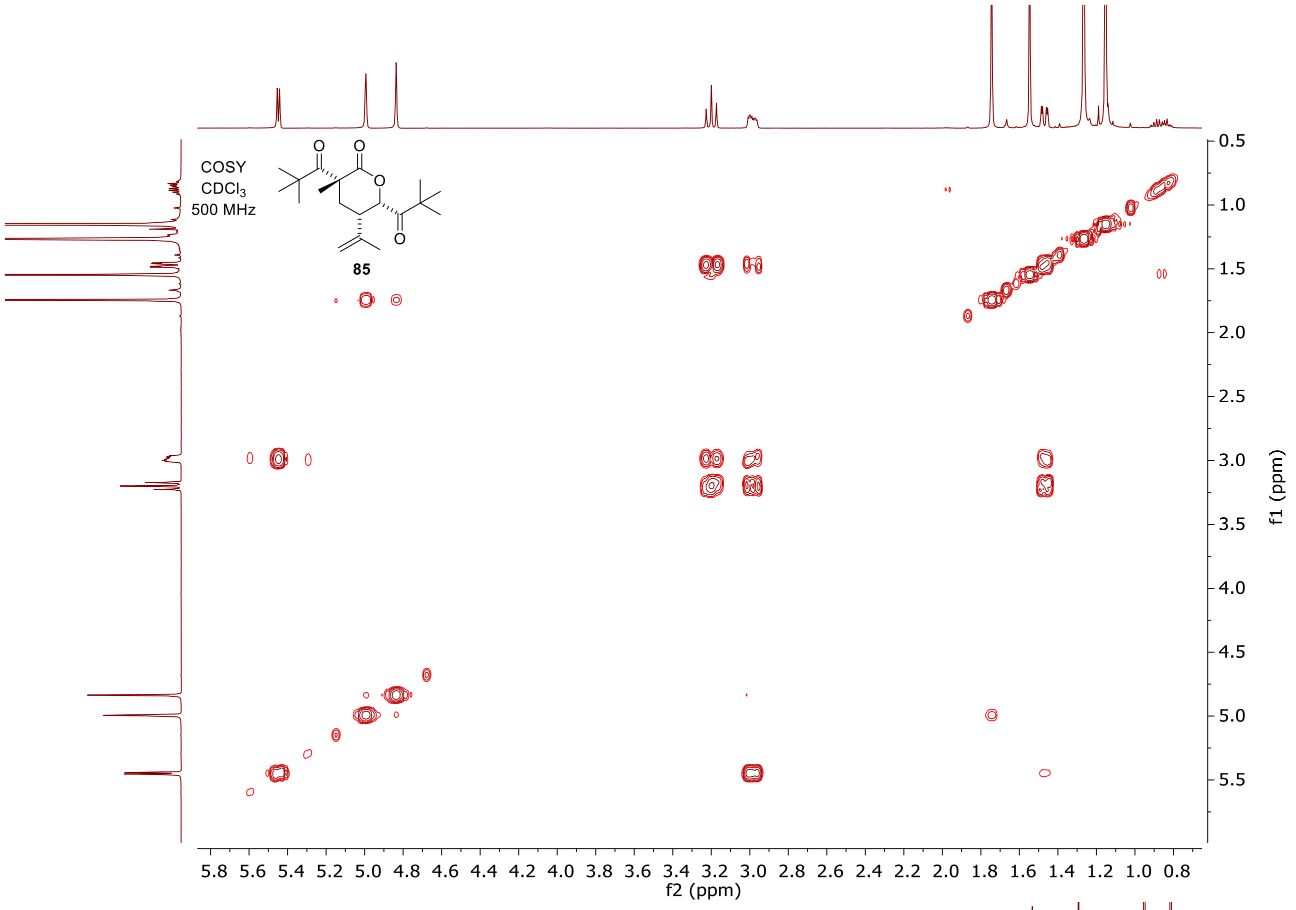
COSY
CDCl3
500 MHz
85
f2 (ppm)
f1 (ppm)

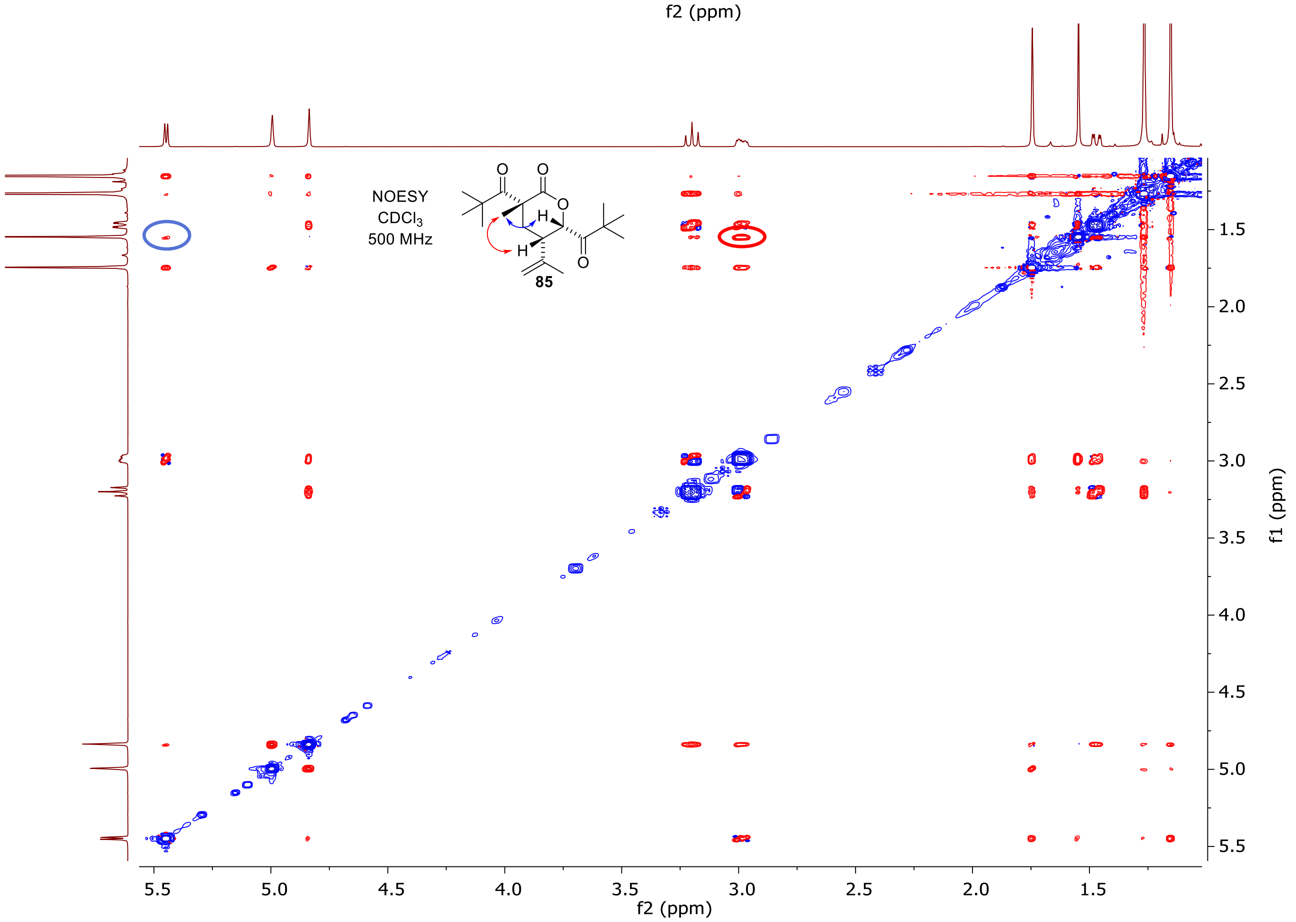
NOESY
CDCl3
500 MHz
85
f2 (ppm)
f1 (ppm)

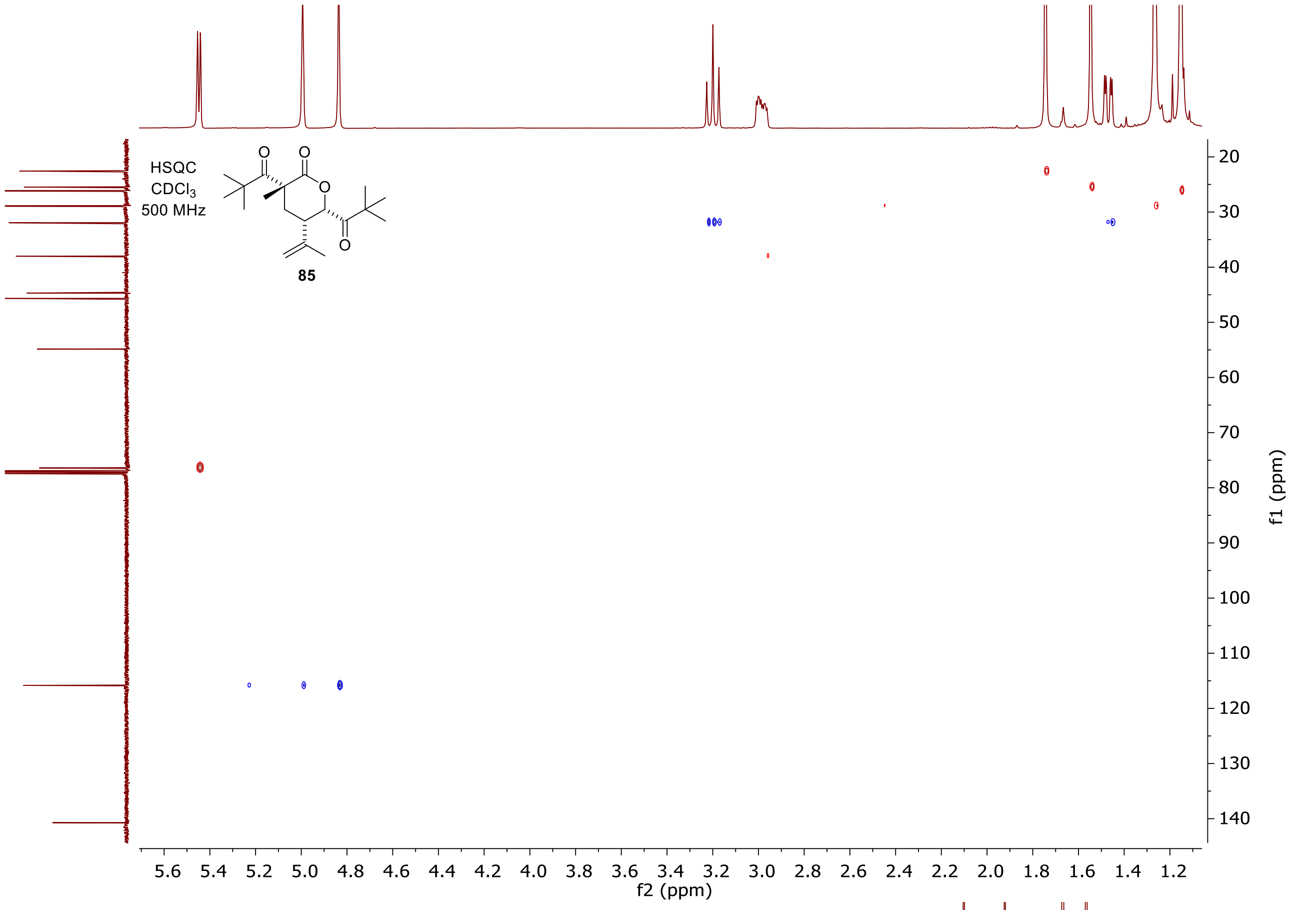
HSQC
$CDCl_3$
500 MHz
85
f2 (ppm)
f1 (ppm)

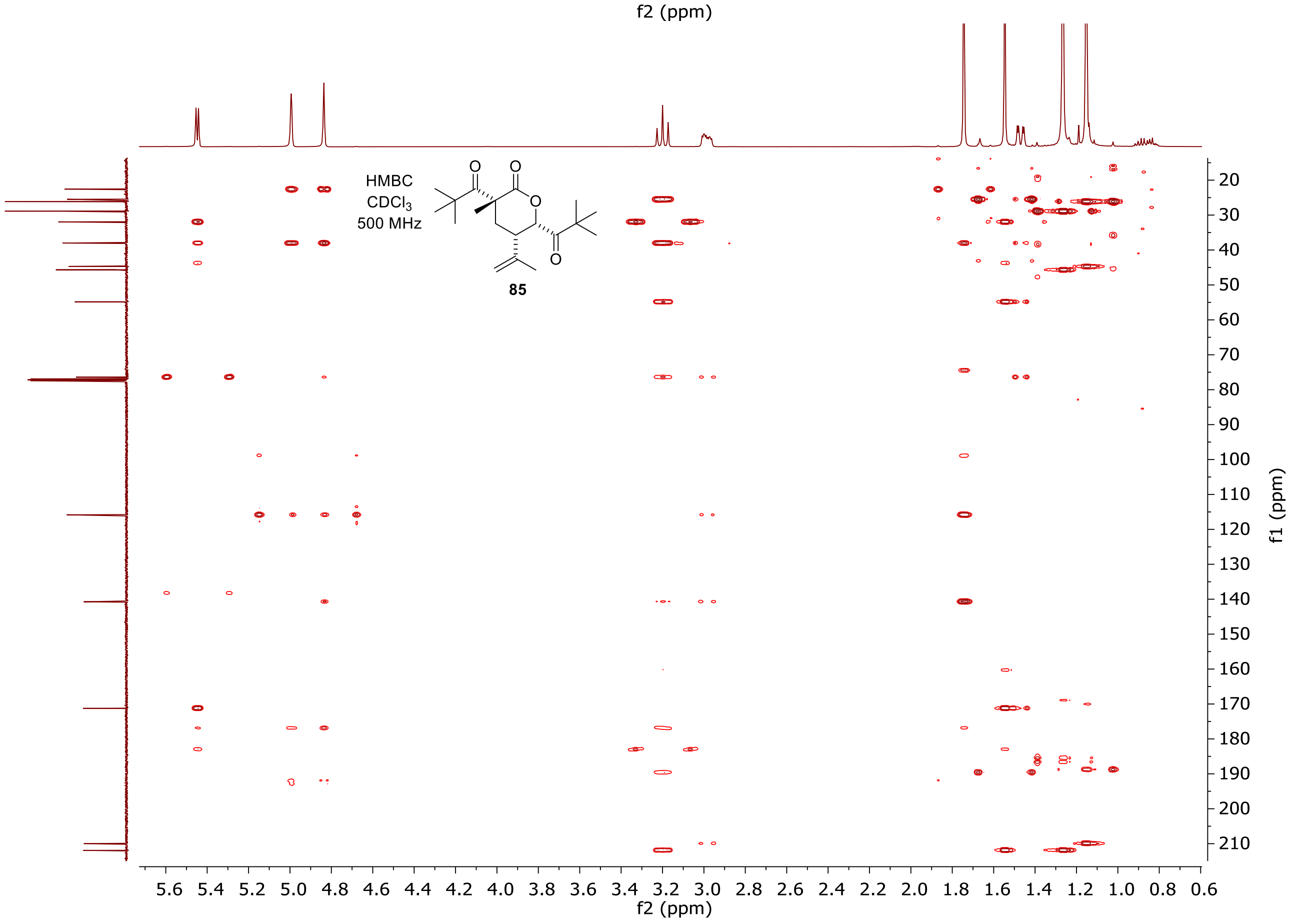
HMBC
$CDCl_3$
500 MHz
85
f2 (ppm)
f1 (ppm)

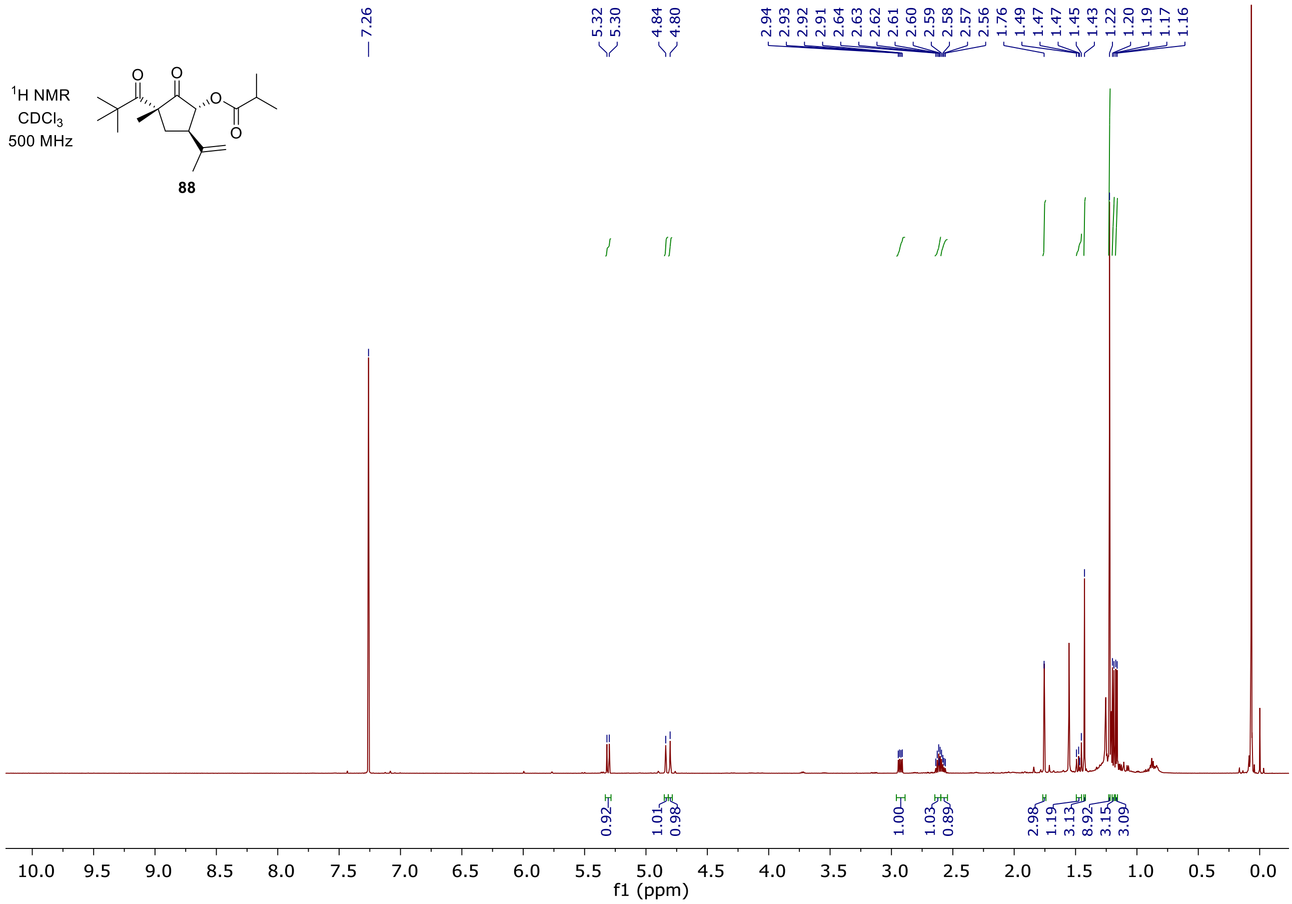
7.26
5.32
5.30
4.84
4.80
2.94
2.93
2.92
2.91
2.64
2.63
2.62
2.61
2.60
2.59
2.58
2.57
2.56
1.76
1.49
1.47
1.47
1.45
1.43
1.22
1.20
1.19
1.17
1.16
1H NMR
CDCl3
500 MHz
88
0.92
1.01
0.98
1.00
1.03
0.89
2.98
1.19
3.13
8.92
3.15
3.09
f1 (ppm)

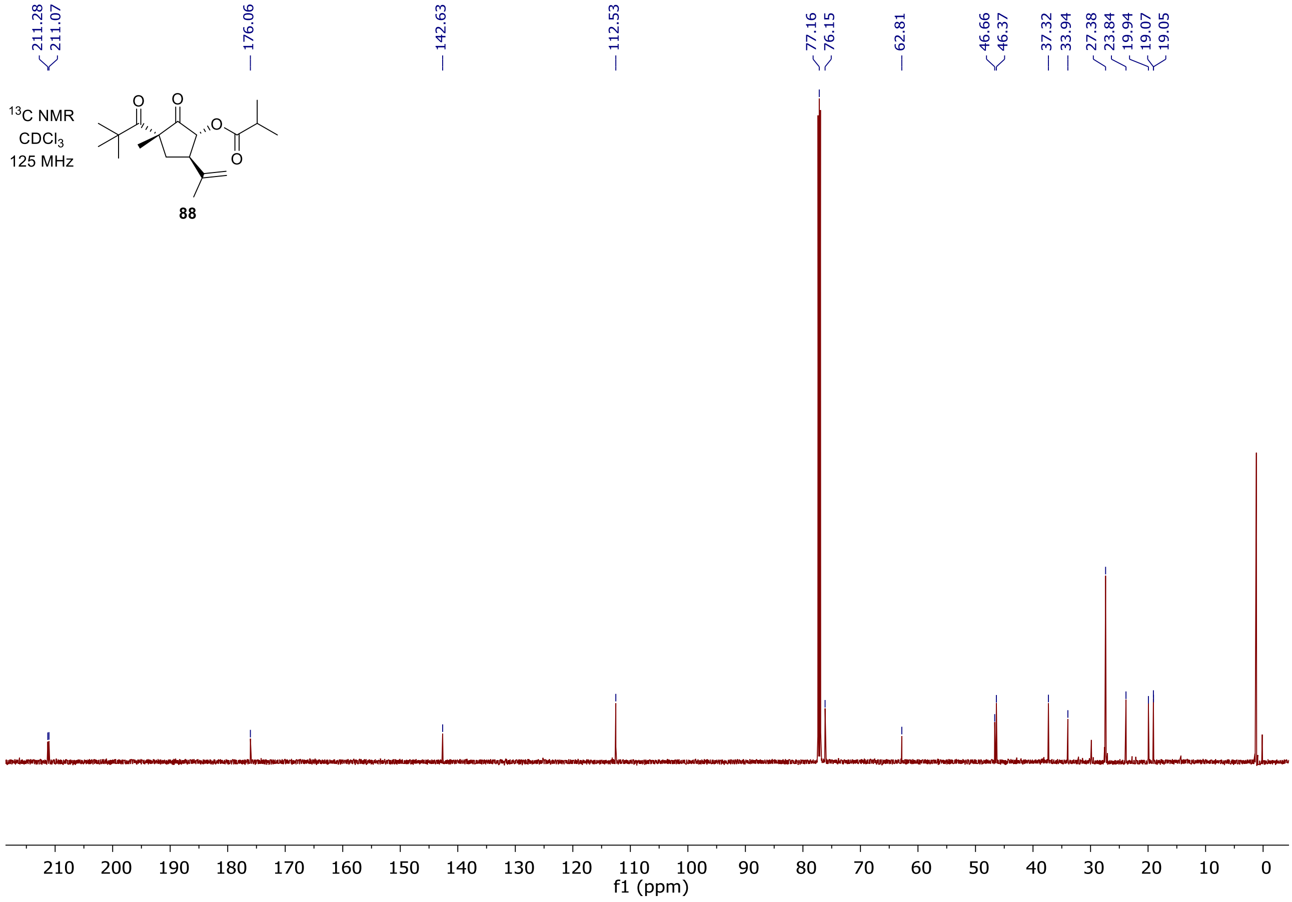
211.28
211.07
176.06
142.63
112.53
77.16
76.15
62.81
46.66
46.37
37.32
33.94
27.38
23.84
19.94
19.07
19.05
13C NMR
CDCl3
125 MHz
88
f1 (ppm)

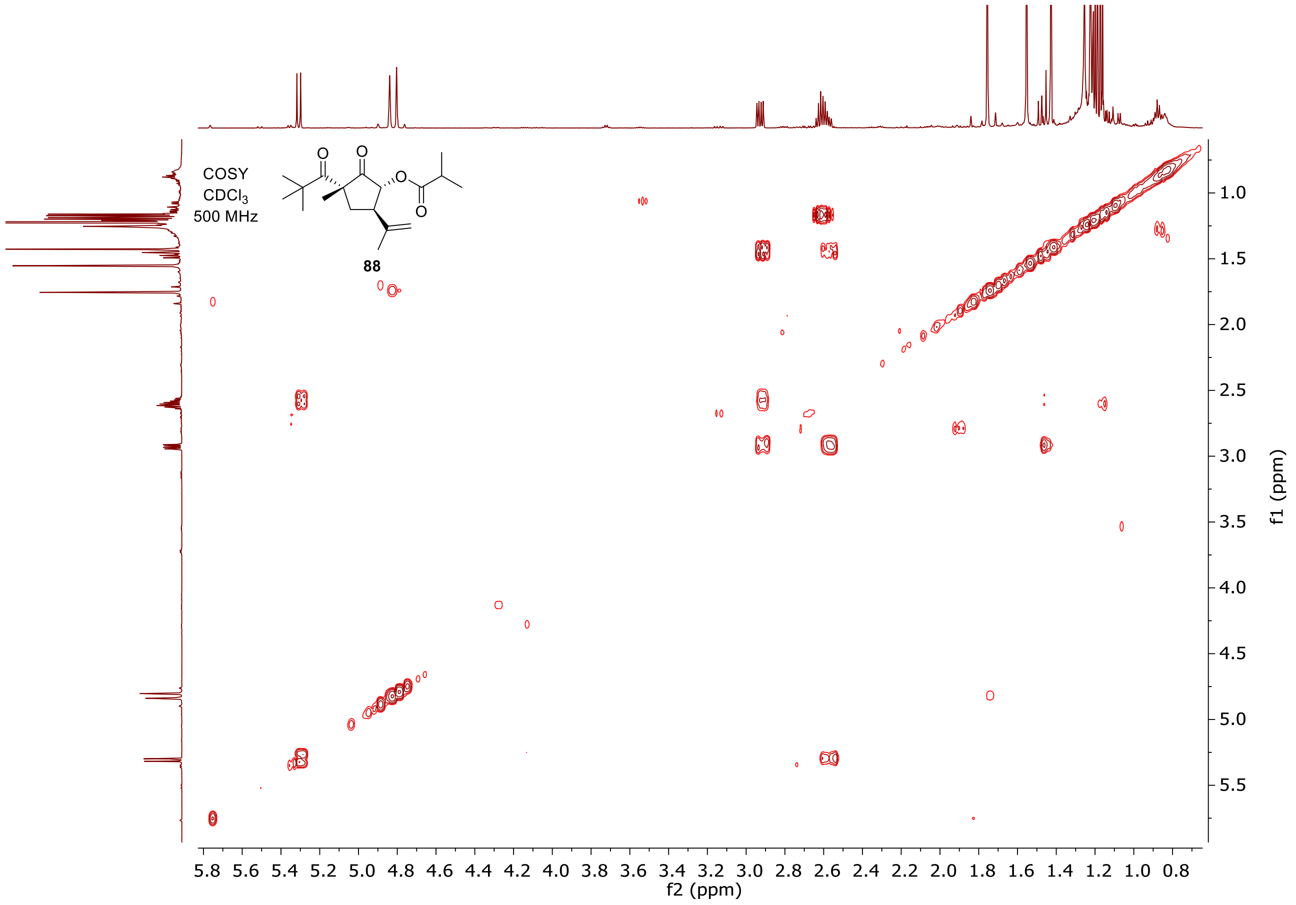
COSY
$CDCl_3$
500 MHz
88
f2 (ppm)
f1 (ppm)

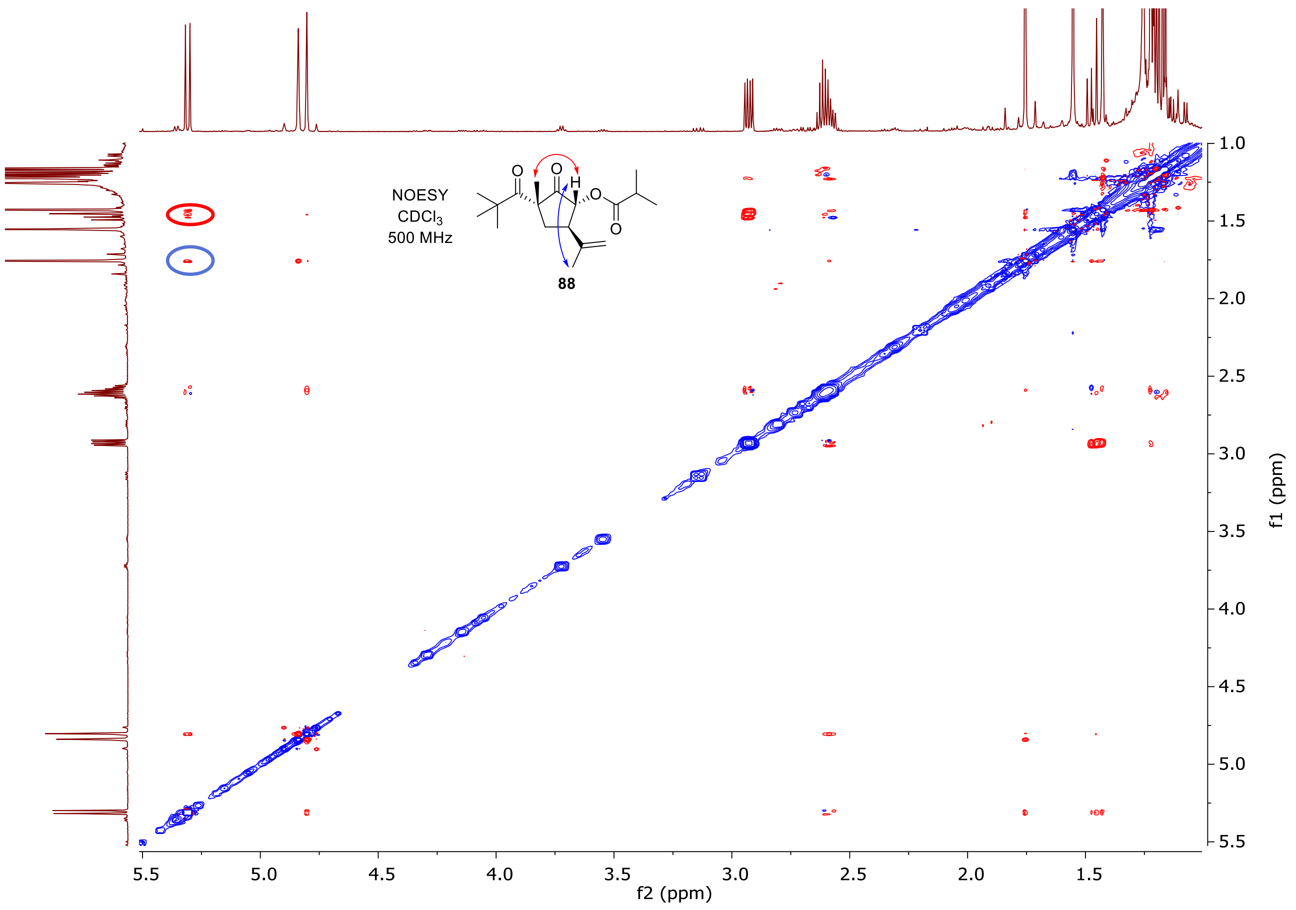
NOESY
$CDCl_3$
500 MHz
88
f2 (ppm)
f1 (ppm)

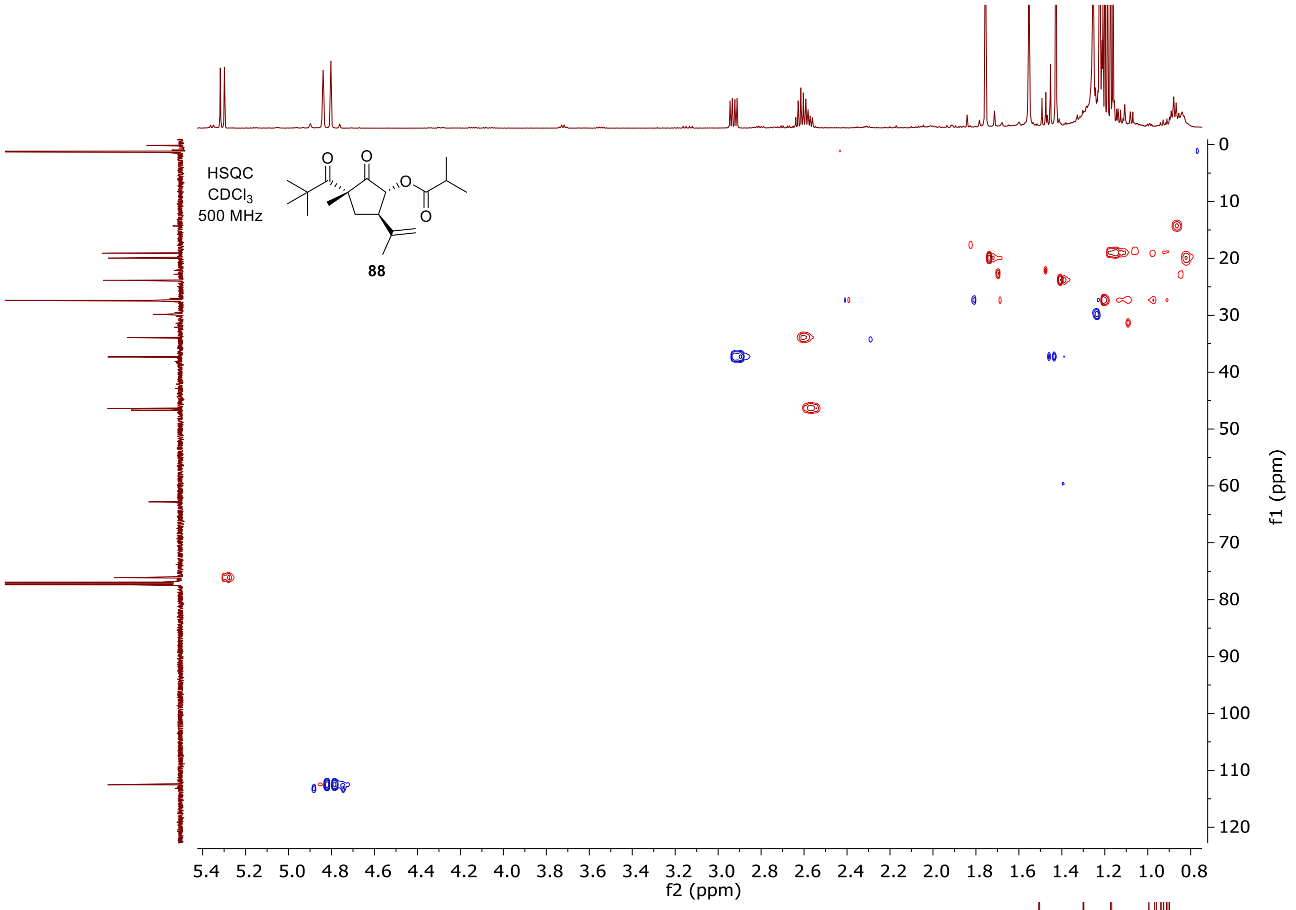
HSQC
CDCl3
500 MHz
88
f2 (ppm)
f1 (ppm)

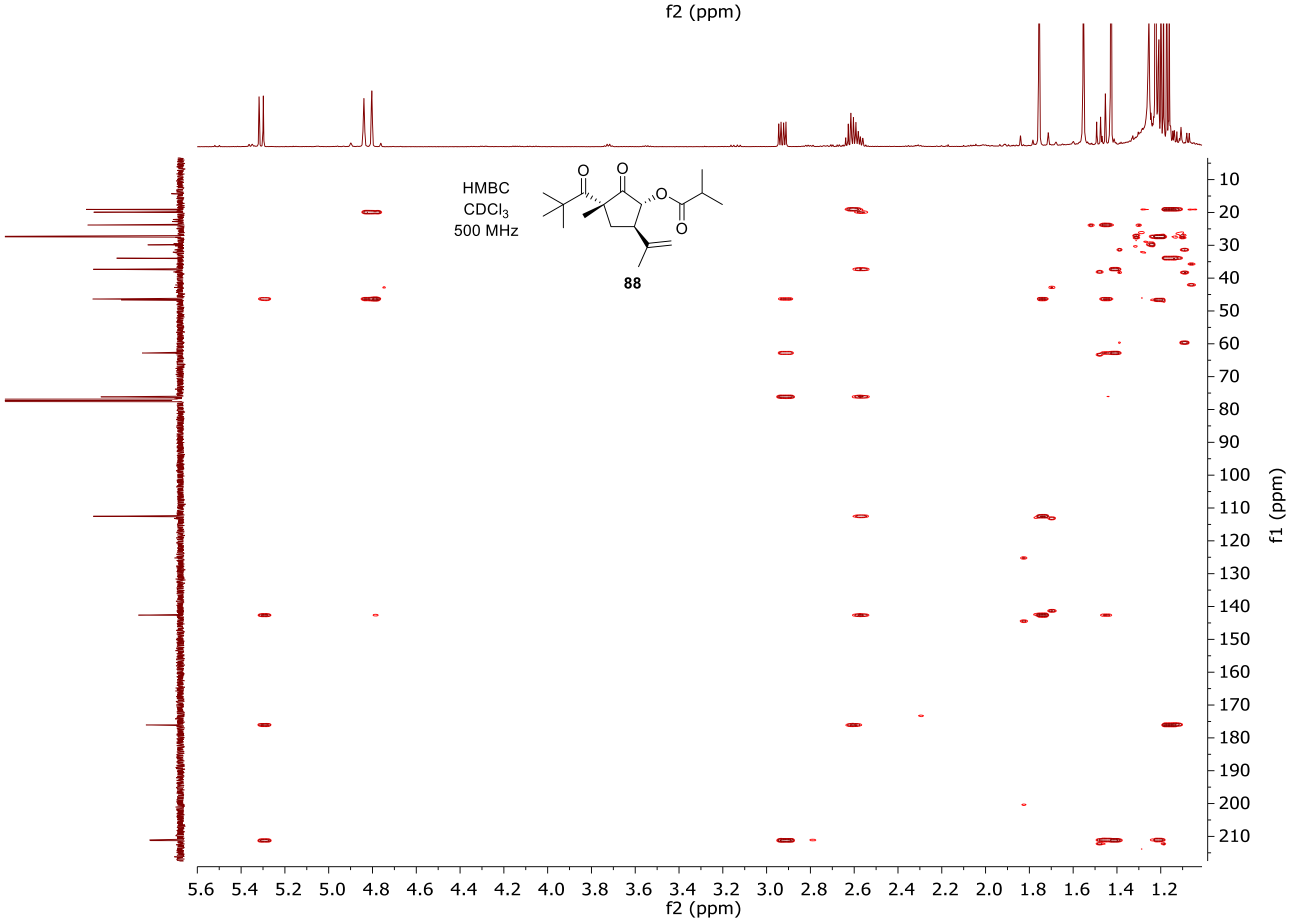
HMBC
CDCl3
500 MHz
88
f2 (ppm)
f1 (ppm)

4. Single Crystal X-Ray Data

To form single crystals suitable for X-ray analysis, **29** (20 mg, 72.4 µmol) **80** (10 mg, 32.4 µmol), **77** (10 mg, 32.4 µmol) were dissolved in toluene (0.5 mL) in a 5 mL vial respectively and left at room temperature overnight to afford colourless blocks. Single crystals were mounted in paratone-N oil on a MiteGen crystal mount and X-ray diffraction was collected at 150(2) K on an Oxford X-calibur single crystal diffractometer using Mo Ka radiation. The data set was corrected for absorption and the structures solved by direct methods using SHELXS-97[4] and refined by full-matrix least squares on F2 by SHELXL-2015,[5] interfaced through the programs X-Seed[6] and Olex2.[7] In general, all nonhydrogen atoms were refined anisotropically and hydrogen atoms were included as invariants at geometrically estimated positions. Full data for the structure determination have been deposited with the Cambridge Crystallographic Data Centre, CCDC reference numbers CCDC 2150336 (**29**), CCDC 2100744 (**80**) and CCDC 2100745 (**77**). Copies of this information may be obtained free of charge from The Director, CCDC, 12 Union Street, Cambridge CB2 1EZ, U.K. (fax, +44-1223-336-033; e-mail, deposit@ccdc.cam.ac.uk). **Table S1** below provides the crystal data and structure refinement details for **29**, **80** and **77**.

[4]G. M. Sheldrick, *Acta Crystallogr.* **1990**, *A46*, 467.
[5]G. M. Sheldrick, *Acta Crystallogr.* **2008**, *A64*, 112.
[6]L. J. Barbour, *J. Supramol. Chem.* **2001**, *1*, 189.
[7]L. J. Bourhis, O. V. Dolomanov, R. J. Gildea, J. A. K. Howard, H. Puschmann, *Acta Crystallogr.* **2015**, *A71*, 59.

**Table S1**: X-ray experimental data for compounds **29**, **80** and **77**			
**Identification code**	**29**	**80**	**77**
**Empirical formula**	$C_{34}H_{52}O_6$	$C_{18}H_{29}O_4$	$C_{24}H_{38.67}O_{5.33}$
**Formula weight**	450.53	309.41	412.55
**Temperature/K**	150.00(14)	104.2(6)	149.9(2)
**Crystal system**	monoclinic	monoclinic	orthorhombic
**Space group**	Pn	I2/a	Pbca
**a/Å**	6.0397(2)	15.9746(9)	9.48300(10)
**b/Å**	11.0543(4)	9.6759(4)	17.5388(2)
**c/Å**	11.9206(5)	22.2192(9)	20.3501(3)
**α/°**	90	90	90
**β/°**	100.153(4)	95.308(5)	90
**γ/°**	90	90	90
**Volume/Å^3**	783.41(5)	3419.7(3)	3384.64(7)
**Z**	1	8	6
**ρ_{calc}g/cm^3**	0.955	1.202	1.214
**μ/mm^{-1}**	0.066	0.083	0.084
**F(000)**	240.0	1352.0	1352.0
**Crystal size/mm^3**	0.27 × 0.22 × 0.08	0.3 × 0.24 × 0.05	0.35 × 0.26 × 0.05
**Radiation**	Mo Kα (λ = 0.71073)	MoKα (λ = 0.71073)	MoKα (λ = 0.71073)
**2Θ range for data collection/°**	6.946 to 58.772	6.58 to 58.646	6.638 to 58.76
**Index ranges**	-8 ≤ h ≤ 8, -15 ≤ k ≤ 15, -16 ≤ l ≤ 15	-21 ≤ h ≤ 20, -13 ≤ k ≤ 12, -30 ≤ l ≤ 30	-12 ≤ h ≤ 12, -24 ≤ k ≤ 24, -27 ≤ l ≤ 27
**Reflections collected**	27151	19940	139242
**Independent reflections**	3922 [Rint = 0.0372, Rsigma = 0.0242]	4219 [R_{int} = 0.0915, R_{sigma} = 0.0890]	4495 [R_{int} = 0.0653, R_{sigma} = 0.0242]
**Data/restraints/parameters**	3922/2/180	4219/0/208	4495/0/208
**Goodness-of-fit on F^2**	0.934	1.026	1.052
**Final R indexes [I>=2σ (I)]**	R1 = 0.0364, wR2 = 0.1078	R_1 = 0.0627, wR_2 = 0.1243	R_1 = 0.0541, wR_2 = 0.1345
**Final R indexes [all data]**	R1 = 0.0402, wR2 = 0.1132	R_1 = 0.1144, wR_2 = 0.1448	R_1 = 0.0719, wR_2 = 0.1436
**Largest diff. peak/hole/e Å^{-3}**	0.25/-0.20	0.34/-0.28	0.53/-0.56

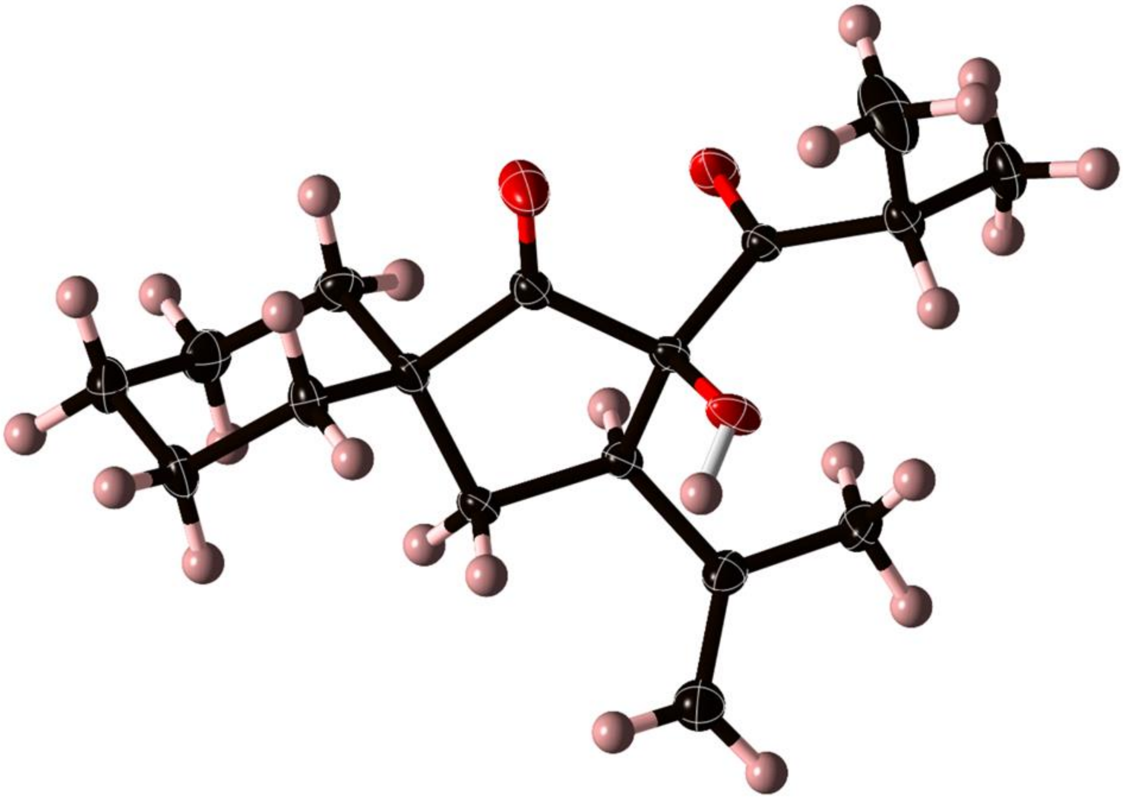

Figure S1: A perspective view of one selected molecule of the asymmetric unit of **29**, with all non-hydrogen atoms represented by ellipsoids at the 50% probability level (Carbon, black; Hydrogen, white; Oxygen, red).

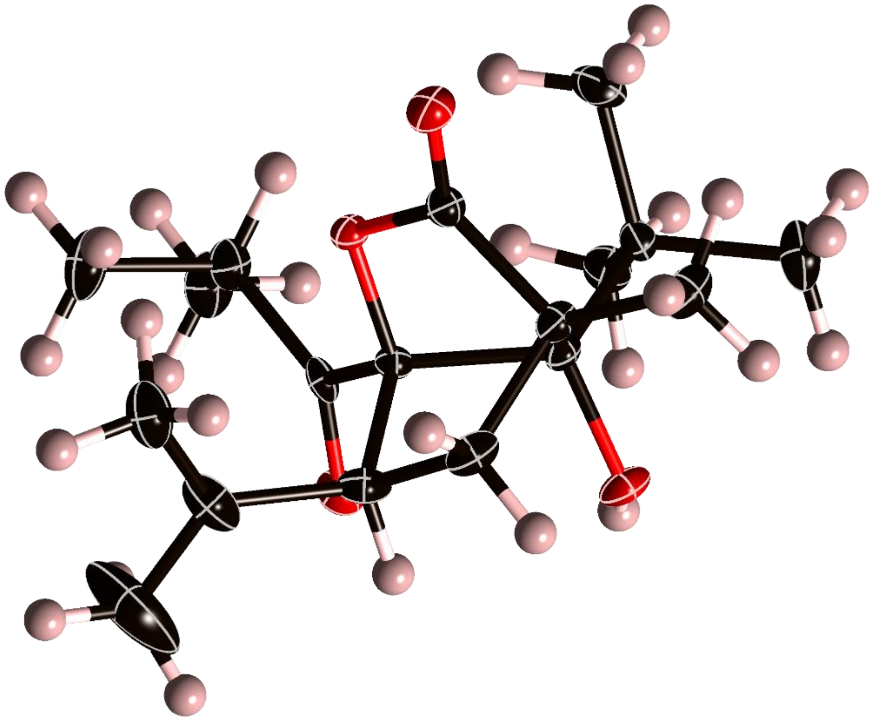

Figure S2: A perspective view of one selected molecule of the asymmetric unit of **80**, with all non-hydrogen atoms represented by ellipsoids at the 50% probability level (Carbon, black; Hydrogen, white; Oxygen, red).

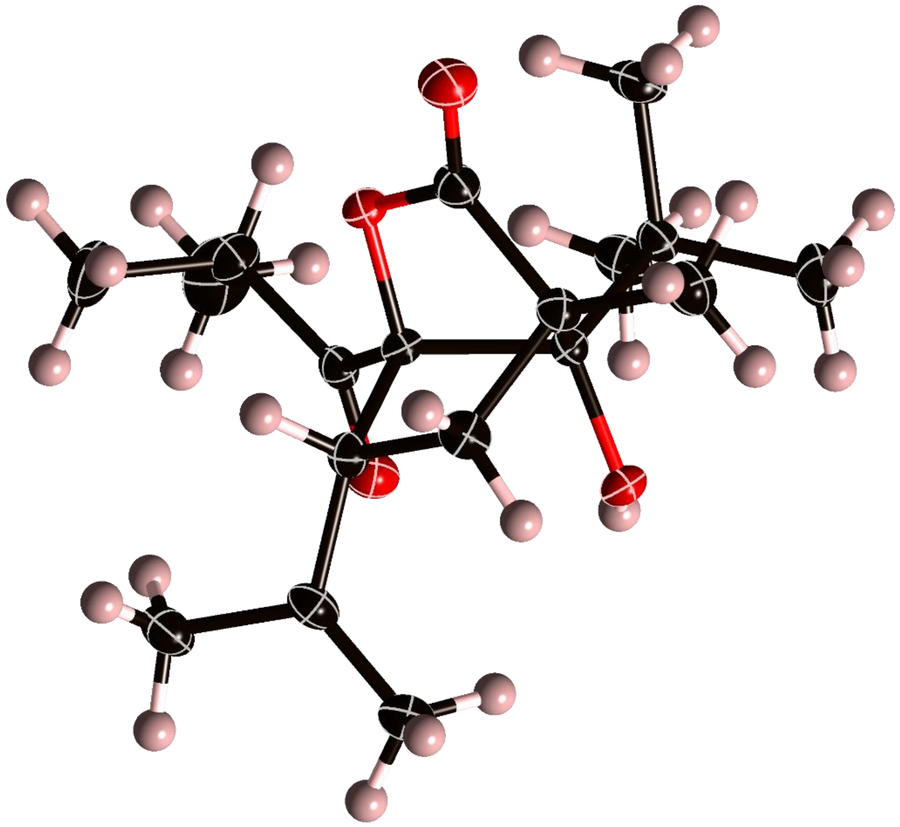

Figure S3: A perspective view of one selected molecule of the asymmetric unit of **77**, with all non-hydrogen atoms represented by ellipsoids at the 50% probability level (Carbon, black; Hydrogen, white; Oxygen, red).

5. Computational Analysis

Calculations were carried out with density functional theory (DFT) using Gaussian 16 (revision A.03).[8] Geometries were optimised in the gas phase at the M06-2X/6-31+G(d) level.[9] Stable ground states and transition states were identified by the number of imaginary vibrational frequencies (0 and 1, respectively). As a starting point for the DFT geometry optimisation of each reactant, product, and reaction intermediate in Scheme 9, we used the global minimum-energy conformer obtained from a conformer search using a metadynamics-based algorithm[11] and semiempirical tight-binding at the GFN2-xTB[11] level. This conformer search was carried out using xtb[12] and the Conformer–Rotamer Ensemble Sampling Tool (CREST)[13] with default parameters. The transition states in Scheme 9 of the main paper were obtained using the synchronous transit-guided quasi-Newton (STQN)[14] method. The transition states were shown to connect reactants and products by using a combination of intrinsic reaction coordinate (IRC) calculations[15] and relaxed potential energy scans. Single-point energy calculations of the optimised geometries were carried out at the M06-2X/6-311+G(d,p) level using the SMD continuum solvent model[16] (water or THF). The optimised geometries are given below in Cartesian coordinates and the energies given in units of Hartree. The Gibbs free energy in solution at 298.15 K was calculated as $G_{\text{soln}} = E_{\text{M06-2X/6-311+G(d,p),sol.}} + G_{\text{thermal,M06-2X/6-31+G(d)}}$, where $G_{\text{thermal,M06-2X/6-31+G(d)}}$ is the thermal correction to the free energy at 298.15 K determined from unscaled frequencies. The results are summarised in **Table S2**.

---

[8]M. J. Frisch, G. W. Trucks, H. B. Schlegel, G. E. Scuseria, M. A. Robb, J. R. Cheeseman, G. Scalmani, V. Barone, G. A. Petersson, H. Nakatsuji, X. Li, M. Caricato, A. V. Marenich, J. Bloino, B. G. Janesko, R. Gomperts, B. Mennucci, H. P. Hratchian, J. V. Ortiz, A. F. Izmaylov, J. L. Sonnenberg, D. Williams-Young, F. Ding, F. Lipparini, F. Egidi, J. G Coings, B. Peng, A. Petrone, T. Henderson, D. Ranasinghe, V. G. Zakrzewski, J. Gao, N. Rega, G. Zheng, W. Liang, M. Hada, M. Ehara, K. Toyota, R. Fukuda, J. Hasegawa, M. Ishida, T. Nakajima, Y. Honda, O. Kitao, H. Nakai, T. Vreven, K. Throssell, J. A. Montgomery, Jr., J. E. Peralta, F. Ogliaro, M. J. Bearpark, J. J. Heyd, E. N. Brothers, K. N. Kudin, V. N. Staroverov, T. A. Keith, R. Kobayashi, J. Normand, K. Raghavachari, A. P. Rendell, J. C. Burant, S. S. Iyengar, J. Tomasi, M. Cossi, J. M. Millam, M. Klene, C. Adamo, R. Cammi, J. W. Ochterski, R. L. Martin, K. Morokuma, O. Farkas, J. B. Foresman, and D. J. Fox, Gaussian, Inc., Wallingford CT, **2016**.
[9]Y. Zhao, D. Truhlar, *Theor. Chem. Acc.* **2008**, *120*, 215.
[10]S. Grimme, *J. Chem. Theory Comput.* **2019**, *15*, 2847.
[11]C. Bannwarth, S. Ehlert, S. Grimme, *J. Chem. Theory Comput.* **2019**, *15*, 1652.
[12]C. Bannwarth, E. Caldewyher, S. Ehlert, A. Hansen, P. Pracht, J. Seibert, S. Spicher, S. Grimme, *WIRES. Comput. Mol. Sci.* **2020**, *11*, e01493.
[13]P. Pracht, F. Bohle, S. Grimme, *Phys. Chem. Chem. Phys.* **2020**, *22*, 7169.
[14]C. Peng, H. B. Schlegel, *Israel J. Chem.* **1993**, *33*, 449.
[15]H. P. Hratchian, H. B. Schlegel, *J. Chem. Phys.* **2004**, *120*, 9918.
[16]A. V. Marenich, C. J. Cramer, D. G. Truhlar, *J. Phys. Chem. B* **2009**, *113*, 6378.

Table S2: Calculations Summary.

structure	# of imaginary frequencies	electronic energy<br><br>M06-2X/<br>6-311+(d,p)<br>(a.u.)	free energy correction<br><br>M06-2X/<br>6-31+(d)<br>(a.u., 298 K)	Gibbs free energy<br><br>M06-2X/<br>6-311+(d,p)<br>(a.u., 298 K)	relative free energy<br><br>kJ/mol (298K)
		SMD: water		***SMD: water***	
**18**	0	-1268.283473	0.40388	-1267.879593	0.0
TS **18** → **19**	1	-1268.260698	0.40504	-1267.855658	62.8
TS **18** → **20**	1	-1268.260239	0.404343	-1267.855896	62.2
**86**	0	-1003.498569	0.379081	-1003.119488	0.0
TS **86** → **75**	1	-1003.472205	0.381486	-1003.090719	75.5
TS **86** → **76**	1	-1003.46831	0.379817	-1003.088493	81.4
		SMD: THF		***SMD: THF***	
**75**$^-$	0	-1003.023142	0.371158	-1002.651984	0.0
**89**$^-$	0	-1003.018876	0.370476	-1002.648400	9.4
**77**$^-$	0	-1003.029196	0.375759	-1002.653437	-3.8
**78**$^-$	0	-1003.024649	0.372895	-1002.651754	0.6
**79**$^-$	0	-1003.034589	0.368699	-1002.665890	-36.5
TS **75**$^-$ → **89**$^-$ [a]	1	-1003.015255	0.370083	-1002.645172	17.9
TS **89**$^-$ → **78**$^-$ [a]	1	-1003.008754	0.371553	-1002.637201	38.8
TS **78**$^-$ → **77**$^-$	1	-1003.020614	0.374311	-1002.646303	14.9
TS **75**$^-$ → **79**$^-$	1	-1003.006099	0.369410	-1002.636689	40.2
**76**$^-$	0	-1003.021035	0.370332	-1002.650703	0.0
**90**$^-$	0	-1003.016912	0.371146	-1002.645766	13.0
**80**$^-$	0	-1003.035995	0.375177	-1002.660818	-26.6
**87**$^-$	0	-1003.034871	0.372110	-1002.662761	-31.7
**88**$^-$	0	-1003.037807	0.367998	-1002.669809	-50.2
TS **76**$^-$ → **90**$^-$ [a]	1	-1003.011990	0.369094	-1002.642896	20.5
TS **90**$^-$ → **87**$^-$ [a]	1	-1003.014912	0.371418	-1002.643494	18.9
TS **87**$^-$ → **80**$^-$	1	-1003.030681	0.373802	-1002.656879	-16.2
TS **76**$^-$ → **88**$^-$	1	-1003.004413	0.370604	-1002.633809	44.4

[a] The IRC calculation starting from this TS yielded a higher energy conformer than the minimum-energy conformer for one of the stable states (**75**$^-$ for TS **75**$^-$ → **89**$^-$, **76**$^-$ for TS **76**$^-$ → **90**$^-$, **78**$^-$ for TS **89**$^-$ → **78**$^-$, and **87**$^-$ for TS **90**$^-$ → **87**$^-$). However, from a relaxed potential energy scan of the relevant dihedral angle (corresponding to rotation of the pivaloyl group as a whole for 75$^-$ and 76$^-$, and to rotation of the t-butyl part of the pivaloyl group for 78$^-$ and 87$^-$) at the M06-2X/6-31+G(d) level, the barrier to convert the minimum-energy conformer to the higher energy conformer was found to be significantly lower than the transition-state barrier.

Cartesian Coordinates

Triketone 18

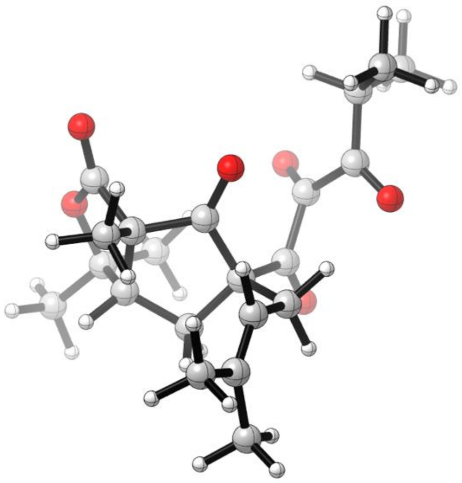

C	-0.30636100	0.51235500	-0.71645700
C	-0.17681800	2.07558200	-0.67813200
C	-1.10239800	2.72102400	0.31067900
C	2.16390800	-0.44918900	-0.93772300
C	0.85410800	0.04545800	-1.60638200
H	-0.35136900	2.41960800	-1.70207600
H	0.85477700	2.33439700	-0.42571600
C	3.09024800	0.58715200	-0.28279900
O	3.00273300	1.73577900	-0.65750700
O	0.77487200	0.03474500	-2.80869300
C	4.08069700	0.07776700	0.73376300
H	3.61966100	-0.77943000	1.23763400
C	-0.16508400	-0.07644900	0.68978400
C	-1.68861900	0.04961200	-1.21394200
H	-2.37449800	0.90180900	-1.18931900
H	-1.64568400	-0.29993000	-2.24947100
C	-2.19878100	-1.02075400	-0.22352000
H	-3.27824500	-0.91007600	-0.07042200
C	-1.45159300	-0.80309600	1.09458900
C	5.33332700	-0.40848000	-0.01916200
H	6.06092800	-0.79595600	0.70029800
H	5.79632100	0.42338900	-0.56064300
H	5.09501200	-1.20534800	-0.72874000
C	4.41361000	1.17301000	1.74286000
H	5.12886300	0.79415500	2.47900400
H	3.51507500	1.50468900	2.27166200
H	4.85573100	2.03841900	1.24049100
O	0.83132200	0.03462100	1.36633600
C	-2.18893900	0.00908000	2.16915100
H	-1.53335400	0.15389700	3.03214700
H	-3.07848800	-0.53835400	2.49450600
H	-2.49892800	0.98056400	1.77452900
C	-1.91850000	-2.51260000	-0.56594300
C	-0.70619400	-2.76141500	-1.45436500
H	0.21207200	-2.35980500	-1.01455500
H	-0.83448900	-2.33523700	-2.45328300
H	-0.55611600	-3.84017100	-1.55470000
C	-3.15358200	-3.20763900	-1.11281700
H	-3.98621400	-3.12199100	-0.40842600
H	-2.95311400	-4.26786600	-1.29048100
H	-3.44659500	-2.74476800	-2.06165400
C	-1.20297000	-2.21462200	1.62820400
O	-1.61154500	-3.11992700	0.71772600
O	-0.77998900	-2.50874500	2.70952700
O	2.44559300	-1.62089000	-0.98139800

C	-2.23214900	3.39707200	0.05895600
C	-3.03909500	3.99058100	1.18507900
H	-3.13381000	5.07655400	1.06400000
H	-2.58499400	3.79340300	2.16010700
H	-4.05801600	3.58257100	1.18819400
H	-0.79129300	2.62770300	1.35297100
C	-2.80629800	3.64270400	-1.31200000
H	-2.21379500	3.20836600	-2.11935600
H	-2.89256100	4.72035200	-1.49672700
H	-3.82186800	3.23146900	-1.37684600

TS 18 → 19

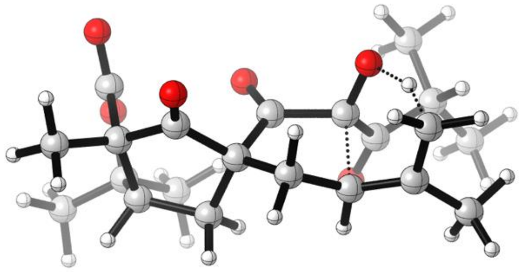

C	-0.52823000	-1.12211900	0.34048200
C	-1.75165300	-1.67994100	-0.38625800
C	-2.97043900	-0.80622600	-0.08926200
C	-2.60796300	0.00116200	1.16761300
C	-1.14565100	-0.36925700	1.54430300
H	-3.25652200	-0.27769100	2.00419000
H	-1.16026800	-1.05751000	2.39765400
H	-0.54819200	0.49754100	1.84062500
C	0.53300900	-2.15828000	0.68830800
H	0.68508100	-2.80785700	-0.17940600
H	0.21738400	-2.78662800	1.52904300
C	0.20267200	-0.10881700	-0.53199000
C	1.74949900	-0.21279500	-0.48801200
C	2.39446600	1.07302300	0.04783900
O	-0.33125700	0.72893900	-1.21162900
O	2.25763900	-0.78308500	-1.52507400
O	1.86393300	1.65962100	0.97103200
C	1.78613700	-1.37413400	1.04159100
H	1.64161700	-0.67250400	1.86513500
C	3.09788700	-1.85463700	0.88460700
C	4.22187600	-1.15499100	1.59676500
H	5.03480200	-0.91172000	0.90332100
H	4.64008800	-1.82312000	2.35940500
H	3.88768700	-0.24014400	2.09460900
C	3.39494500	-2.70835200	-0.20713200
H	3.04412300	-1.84622500	-1.06257900
H	2.69341900	-3.50600700	-0.44761600
H	4.43839300	-2.98476800	-0.35070500
C	3.63455300	1.56386800	-0.66040600
H	4.22916600	0.68212800	-0.93421600
C	3.17254300	2.23629800	-1.96799900
H	2.59969500	1.54532600	-2.59158300
H	4.04647200	2.57495300	-2.53285100
H	2.54799400	3.10718600	-1.74263600
C	-4.43693100	2.51630700	0.21824300
H	3.83143100	3.38497900	0.49256100
H	5.32388000	2.86591000	-0.31908200
H	4.76402700	2.03343500	1.14522700
C	-3.14636600	0.26177400	-1.18594200
O	-3.11815200	1.48826400	-0.64408400
C	-2.93251100	1.48193400	0.78891600
C	-4.26577900	1.90030800	1.40532700
H	-4.56929700	2.87579100	1.01536200
H	-4.17501400	1.96789900	2.49467200
H	-5.05077500	1.17481700	1.16668900

C	-1.85274200	2.50509700	1.10670300
H	-0.91869000	2.28660100	0.58451500
H	-1.66088900	2.53469100	2.18487000
H	-2.19519000	3.49527100	0.79234400
C	-4.23832400	-1.66134800	-0.04260100
H	-5.11931600	-1.04281100	0.15252600
H	-4.15693300	-2.41700900	0.74495500
H	-4.36644700	-2.16574100	-1.00306800
O	-3.35383900	0.04893600	-2.34576800
O	-1.76293500	-2.70017800	-1.02329200

TS 18 → 20

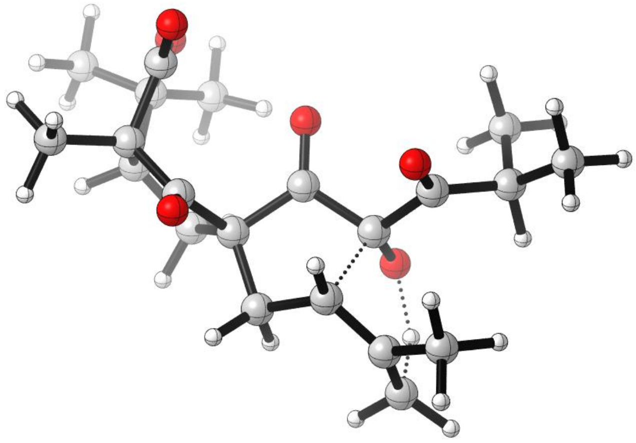

C	0.53246600	-1.06066800	-0.47741200
C	1.35564000	-1.41290800	0.77284000
C	2.75322800	-0.80764800	0.65655600
C	2.95178500	-0.49648500	-0.83509100
C	1.61467500	-0.84158700	-1.55108500
H	3.74028500	-1.12752200	-1.25737600
H	1.74101100	-1.78445000	-2.09521900
H	1.30731500	-0.09095300	-2.28262600
C	-0.54455200	-2.10481900	-0.77558800
H	-0.22114700	-3.10311700	-0.46296100
H	-0.73919000	-2.11844900	-1.85085100
C	-0.23426100	0.22407100	-0.16919200
C	-1.74885400	0.09818400	-0.42956000
C	-2.61233400	0.82125700	0.62055200
O	0.27406900	1.25928800	0.17641500
O	-2.02112500	0.27585100	-1.68816100
O	-2.44281700	0.58790400	1.79746600
C	-1.78834500	-1.69453400	0.00355100
H	-1.64222400	-1.69370800	1.08562900
C	-3.10153200	-1.96162300	-0.44318800
C	-4.24884600	-1.85892800	0.52018200
H	-3.92524700	-1.57658300	1.52365900
H	-4.76013800	-2.82753100	0.57282300
H	-4.98732800	-1.13029800	0.16188800
C	-3.36347700	-1.95202700	-1.84018500
H	-2.91794100	-0.79112500	-2.00986700
H	-4.40737000	-2.03395000	-2.14055600
H	-2.68593500	-2.50229400	-2.49228100
C	-3.62038200	1.82120500	0.09911800
H	-4.10566600	1.36705200	-0.77621400
C	-4.64570000	2.17350800	1.17001300
H	-5.17924800	1.28575200	1.52527900
H	-5.37826200	2.88231000	0.77148900
H	-4.15426200	2.62894500	2.03473500
C	-2.84668700	3.06031400	-0.38784400
H	-2.13525200	2.80121400	-1.17596000
H	-2.29820600	3.51425800	0.44417000
H	-3.55049600	3.79953700	-0.78300100
C	2.80631700	0.57251600	1.33978100

O	3.21983300	1.50169000	0.46602000
C	3.46531500	0.97925400	-0.85871700
C	4.97762900	1.00596800	-1.06881600
H	5.35807900	2.02106100	-0.92530000
H	5.22476400	0.67612000	-2.08353200
H	5.48288800	0.34506100	-0.35668800
C	2.77321900	1.91227600	-1.84040100
H	3.22645300	2.90545500	-1.77221000
H	1.70965700	2.00731700	-1.61253900
H	2.89721900	1.54782000	-2.86601500
C	3.78002600	-1.71659400	1.33613700
H	4.78240400	-1.28214000	1.28064000
H	3.79461700	-2.69866000	0.85332700
H	3.50802300	-1.84401200	2.38641600
O	2.58960500	0.79198300	2.49656100
O	0.97833700	-2.13440400	1.65835400

Triketone 86

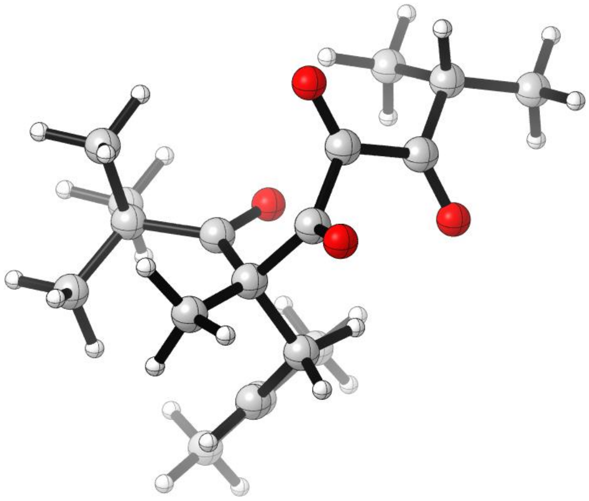

C	-0.80653700	-0.57284200	0.37033200
O	0.07381500	0.12370700	0.84845000
C	-0.70013500	-0.79968700	-1.15070500
C	0.75435100	-1.18423900	-1.47497200
C	1.86269500	-1.06512000	-0.41271600
C	2.65994300	0.25213500	-0.39197500
C	3.70275100	0.40528600	0.68670000
H	4.25331500	-0.54304400	0.73821700
C	-1.62941000	-1.80075400	-1.83883800
H	-1.62968900	-2.77778600	-1.34751100
H	-1.27163800	-1.94343000	-2.86168600
H	-2.65414100	-1.42797300	-1.88312600
C	-0.88414600	0.62291000	-1.79601600
H	-0.01067500	1.23287400	-1.55941600
H	-0.88849000	0.47126300	-2.88186900
C	-2.15421500	1.28258200	-1.33718000
H	-3.07772500	0.89276900	-1.76797100
C	-2.26053300	2.26823600	-0.43287200
C	-1.10250100	2.93633200	0.26116600
H	-1.14331500	2.73651900	1.33979800
H	-1.16941700	4.02370400	0.13310300
H	-0.12675000	2.60277200	-0.09468400
C	-3.61435200	2.79299900	-0.02784300
H	-3.76567700	2.67372100	1.05307300
H	-4.42605000	2.27618900	-0.54785100
H	-3.69568500	3.86580200	-0.24107800
O	1.07929100	-1.60921200	-2.55723800
O	2.13467500	-2.01054100	0.28618500
O	2.38502100	1.09082100	-1.22049800
C	-1.83711100	-1.20223600	1.31727900
C	2.98217300	0.60672400	2.03361800
H	3.72766800	0.72000500	2.82671300

H	2.33580900	-0.23970500	2.27833400
C	4.64477900	1.56147800	0.36951700
H	5.14127600	1.42163800	-0.59499400
H	5.41048900	1.64300700	1.14687500
H	4.09254400	2.50511000	0.32598600
H	2.36439600	1.51049300	2.00353700
C	-1.91949600	-0.32693600	2.57472000
H	-2.57922900	-0.80444300	3.30667000
H	-2.33488600	0.65791800	2.32910600
H	-0.93728100	-0.17861400	3.02733700
C	-3.25488800	-1.37311100	0.75302500
H	-3.33243500	-2.18731700	0.03146900
H	-3.61731800	-0.44969700	0.28888900
H	-3.92669000	-1.61357000	1.58424300
C	-1.24504200	-2.57875700	1.68784400
H	-1.12371900	-3.22548900	0.81219600
H	-1.92096400	-3.08298600	2.38776200
H	-0.26610700	-2.46791900	2.16518100

TS 86 → 75

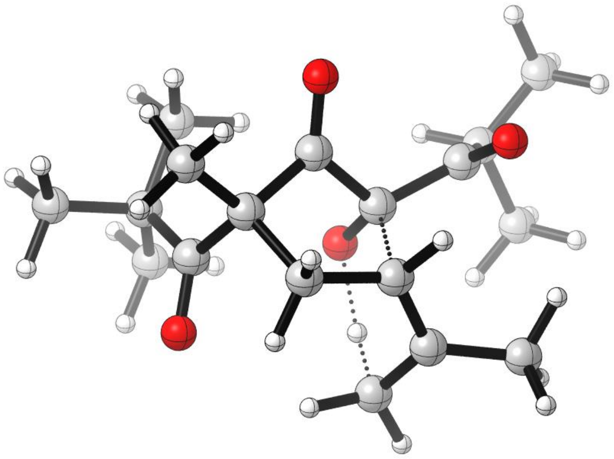

C	2.20386000	0.43767900	-0.23351600
O	2.46655100	1.46234300	-0.82877100
C	1.44352100	0.54410600	1.12104400
C	0.27554200	-0.42420400	1.27017400
C	-0.79217500	-0.07114800	0.21862100
C	-2.18697500	-0.64998900	0.51353300
C	-2.72422800	-1.64996900	-0.49239400
H	-1.87097700	-2.21392600	-0.88739300
C	2.45314000	0.38338100	2.26064400
H	2.91604800	-0.60606900	2.25571400
H	1.94551700	0.50221400	3.22225300
H	3.23878900	1.14145600	2.17743300
C	0.67761100	1.88878000	1.18738500
H	0.70585000	2.25878900	2.21705200
H	1.17279200	2.62566800	0.55228900
C	-2.85483300	2.50326100	-0.40724600
H	-3.38747100	1.91790300	0.34725900
H	-3.25628000	2.28024400	-1.40037100
O	0.14307600	-1.27093900	2.11432400
O	-2.80729100	-0.30953200	1.49792600
C	2.79133800	-0.88643600	-0.75667000
C	-3.34737300	-0.85534500	-1.65368500
H	-4.19312900	-0.25901000	-1.29162900
H	-3.72149100	-1.54632100	-2.41577300
C	-3.74102700	-2.58350400	0.15564300
H	-3.29749600	-3.13674600	0.98851300
H	-4.11287100	-3.30237100	-0.58138000
H	-4.58976000	-2.01504000	0.54717200
H	-3.05181800	3.56475200	-0.20723500
C	-0.80192900	1.70175900	0.80440900
H	-1.47622500	1.63314300	1.65786200

C	-1.37205300	2.27966900	-0.35050700
C	-0.60165800	2.33044900	-1.54315000
H	-1.08993100	2.73367400	-2.42917500
H	0.45625300	2.58490300	-1.45700700
H	-0.49036400	1.10470500	-1.61995500
O	-0.31368800	-0.20616300	-0.98149500
H	-2.61485700	-0.19222600	-2.12439700
C	2.58955900	-0.90936800	-2.27960300
H	3.05094900	-0.03603400	-2.74688300
H	1.52245900	-0.91720500	-2.52075400
H	3.05027900	-1.81403300	-2.69214600
C	2.18581600	-2.15656700	-0.15544700
H	2.29032600	-2.21361600	0.93176400
H	2.70593100	-3.02244400	-0.58071800
H	1.12577900	-2.24996600	-0.41078300
C	4.30375900	-0.83708000	-0.45583500
H	4.51158300	-0.85376300	0.61934000
H	4.75150000	0.06483000	-0.88362700
H	4.78806900	-1.71203700	-0.90372300

TS 86 → 76

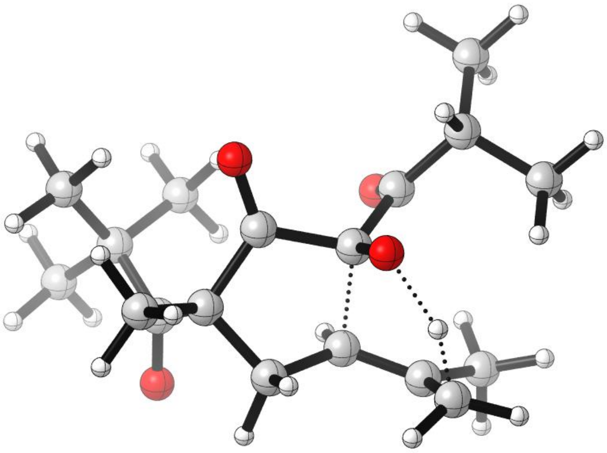

C	2.42820200	-0.56811800	0.35934700
O	2.66888200	-1.44778900	1.15736600
C	1.40362800	-0.91970500	-0.78109600
C	0.39613000	0.18522800	-1.09540900
C	-1.05967000	-0.13426900	-0.63595800
C	-1.67716700	1.04689500	0.13729600
C	-2.91858700	1.67803700	-0.45732500
H	-2.82070200	1.61229700	-1.54693300
C	2.19773900	-1.30049000	-2.04276800
H	1.50811200	-1.69209000	-2.79684700
H	2.93170000	-2.07691500	-1.80370500
H	2.71307300	-0.44104500	-2.47237600
C	0.50542700	-2.06225300	-0.29182300
H	0.03646900	-2.53563700	-1.15872600
H	1.07965300	-2.80879600	0.26015700
C	-2.58216100	-1.53783600	2.05988600
H	-2.38911800	-2.24427500	2.87822800
H	-3.65912900	-1.54513500	1.86970900
O	0.60485300	1.18475600	-1.73576400
O	-1.16033300	1.43600000	1.16550100
C	3.13062200	0.79345900	0.50070100
C	-3.06414100	3.12733700	-0.00599100
H	-3.15077100	3.18607500	1.08315200
H	-3.96046300	3.57042200	-0.45155200
C	-4.13315000	0.83279300	-0.03634400
H	-4.03371200	-0.20741300	-0.36242900
H	-5.04159500	1.24312200	-0.48833000
H	-4.25465500	0.85824700	1.05291100
H	-2.27101100	-0.54593300	2.40092600

C	-0.54622200	-1.40976600	0.59345300
C	-1.80470800	-1.98733400	0.85936000
C	-2.43827300	-2.73876200	-0.17158900
H	-3.45378900	-3.07885200	0.02714600
H	-1.83403300	-3.47975000	-0.69572600
H	-0.12669600	-0.82067100	1.41306200
H	-2.40448500	-1.82366100	-1.00300100
O	-1.75106800	-0.65474600	-1.60030700
C	3.63992500	1.37372600	-0.82643700
H	4.38373800	0.71403400	-1.28797200
H	4.13361100	2.32920300	-0.61598300
H	2.83372000	1.56330100	-1.53689700
C	4.32043400	0.61212100	1.45189500
H	5.03347100	-0.12028900	1.05990900
H	3.99677200	0.26879800	2.43728200
H	4.83615400	1.57150800	1.56557500
C	2.12630000	1.76830600	1.15122500
H	2.66267400	2.68528700	1.42105000
H	1.69657100	1.34672800	2.06649800
H	1.30747900	2.04539900	0.48563700
H	-2.19766800	3.72407900	-0.30567300

α-Hydroxy-β-Diketone Anion 75⁻

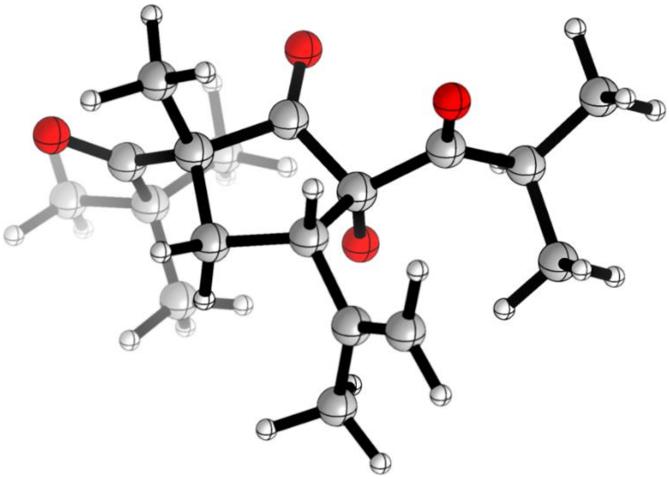

C	0.00000000	0.00000000	0.00000000
O	0.00000000	0.00000000	1.21919995
C	1.36884132	0.00000000	-0.71253157
C	1.52652116	0.94121545	-1.93053613
C	1.86584214	0.09133396	-3.16881624
C	2.81194096	0.86490079	-4.10365230
C	2.25659090	1.24490470	-5.46353381
H	1.21383655	1.53923702	-5.29523785
C	2.44170311	0.47565237	0.28310482
H	2.46556168	-0.18185105	1.15539616
H	2.22585904	1.49448812	0.61590679
H	3.42448929	0.47264429	-0.20090486
C	1.75286904	-1.39628041	-1.26869123
H	2.28565982	-1.98265295	-0.50951736
H	0.86819194	-1.95706097	-1.56807427
C	1.63260772	-3.11309371	-3.79978403
H	1.29965505	-3.70368146	-2.93588293
H	1.86477014	-3.80553459	-4.61637322
H	0.80767157	-2.44836251	-4.08291604
O	1.36862567	2.14179878	-1.87963361
O	3.94979251	1.14858442	-3.76856192
C	-1.36291555	-0.02318929	-0.73598329
C	2.24490113	-0.02038562	-6.33588940
H	3.26509909	-0.40013678	-6.47441359
H	1.82453587	0.20885956	-7.32312758
H	1.63698961	-0.79535822	-5.86066824
C	3.06305550	2.36827989	-6.10285289
H	3.07022305	3.26095601	-5.46904204

H	2.63945391	2.63554872	-7.07868777
H	4.10398514	2.05913946	-6.24782748
C	2.62362374	-1.11223842	-2.50813588
C	2.83092767	-2.27037469	-3.44842181
C	4.03257346	-2.49284973	-3.99804540
H	4.18485466	-3.28948360	-4.72400711
H	4.88713058	-1.86369444	-3.75975213
H	3.60788675	-0.74567232	-2.19023997
O	0.65003222	-0.22118110	-3.67221060
C	-1.52601284	1.21220022	-1.64331141
H	-1.26927727	2.13883428	-1.11844190
H	-2.57905259	1.26920537	-1.94958983
H	-0.91019526	1.12327118	-2.54222917
C	-2.46526483	-0.00972062	0.33052795
H	-2.39197487	-0.87681311	0.99448729
H	-3.44160274	-0.02988381	-0.16780939
H	-2.40801346	0.88912181	0.95273554
C	-1.52650877	-1.28146401	-1.60903447
H	-0.84024996	-1.24837980	-2.46240351
H	-2.55470926	-1.29657662	-1.99430917
H	-1.37694956	-2.19902180	-1.02472361

Endo Epoxide 89⁻

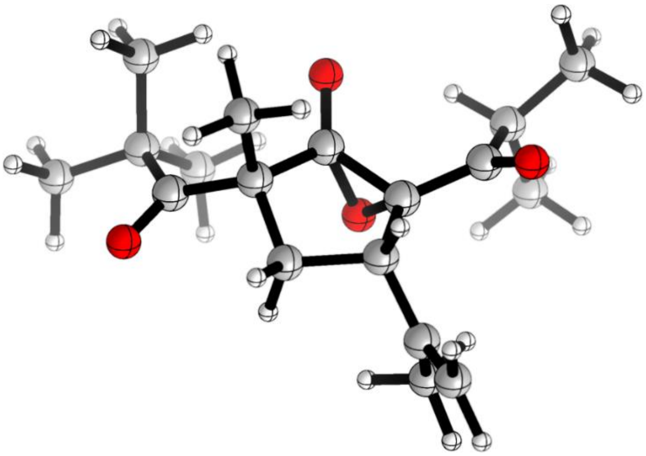

C	0.00000000	0.00000000	0.00000000
O	0.00000000	0.00000000	1.22068474
C	1.37480139	0.00000000	-0.69565075
C	1.46255224	-0.53714728	-2.15502464
C	2.76186010	-1.39692376	-2.16737295
C	3.54870591	-1.50220401	-3.40794595
C	2.81432202	-1.91090168	-4.67557483
H	1.83938811	-1.40843533	-4.63709187
C	1.87164717	1.45774398	-0.72464318
H	1.85988915	1.88476918	0.28460421
H	1.26097279	2.06850049	-1.39374274
H	2.89860304	1.47912388	-1.10808134
C	2.34084005	-0.87663509	0.13216197
H	2.74264109	-0.34052044	0.99735881
H	1.80064834	-1.74643779	0.51785344
C	3.33165635	-3.89978501	-0.63555161
H	2.31086070	-3.83335300	-0.24511015
H	3.83359229	-4.76585526	-0.19211523
H	3.23298521	-4.06449360	-1.71543254
O	0.94431319	0.09060950	-3.13110027
O	4.74736756	-1.23772216	-3.41285344
C	-1.35005334	0.04660290	-0.74084281
C	2.59118821	-3.43054883	-4.64988961
H	3.55208478	-3.96028652	-4.60504954
H	2.07220293	-3.75010418	-5.56191182
H	1.98260393	-3.71816098	-3.78797741
C	3.59139572	-1.48583445	-5.91661332
H	3.74469795	-0.40239594	-5.93198569

H	3.04328279	-1.77251755	-6.82219340
H	4.57835866	-1.96069618	-5.93854912
C	3.46027761	-1.33889397	-0.82531972
C	4.10481466	-2.63222524	-0.37792116
C	5.30235450	-2.64602751	0.21533667
H	5.75874388	-3.57262368	0.55803588
H	5.86967239	-1.72962886	0.36056230
H	4.24264240	-0.57320277	-0.89252906
O	1.46568902	-2.01217539	-2.12374498
C	-1.43525175	1.31386128	-1.60934561
H	-1.32240759	2.21668804	-0.99435078
H	-2.42637619	1.35092723	-2.08113835
H	-0.67091683	1.29346666	-2.39128958
C	-2.47258602	0.07388885	0.30203592
H	-2.45482427	-0.82248596	0.93033346
H	-3.43941140	0.12217129	-0.21371742
H	-2.38314030	0.94177578	0.96290372
C	-1.51765927	-1.20359678	-1.62424336
H	-0.84415638	-1.18357623	-2.48248434
H	-2.55250050	-1.23218091	-1.99062140
H	-1.33455297	-2.12038063	-1.05068561

Lactone Enolate 78⁻

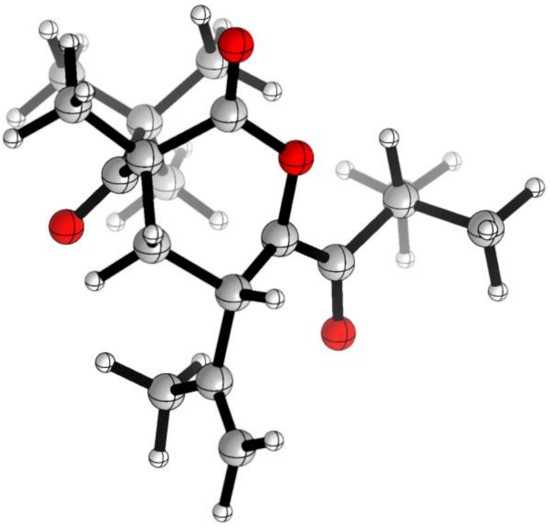

C	0.00000000	0.00000000	0.00000000
O	0.00000000	0.00000000	1.21923448
C	1.39047383	0.00000000	-0.68782544
C	1.43805389	-0.41715952	-2.15606568
C	0.73005260	-2.46655210	-1.24132072
C	-0.21481747	-3.46350143	-1.48281174
C	-0.90288864	-3.52145777	-2.86498057
H	-0.65088543	-2.63893212	-3.46025813
C	1.93160174	1.42898458	-0.55083551
H	2.93973885	1.48888564	-0.97067031
H	1.95582840	1.70861642	0.50688787
H	1.30844877	2.14351767	-1.09891844
C	2.28671257	-1.01187290	0.06492297
H	3.32826273	-0.83310443	-0.22951691
H	2.20842259	-0.80227410	1.13621487
C	0.49966212	-3.00950460	1.86387432
H	0.47114565	-3.69491653	2.71778360
H	-0.41666722	-3.13878327	1.27889216
H	0.52925230	-1.97985204	2.23413398
O	1.82340327	0.30842481	-3.05255791
O	-0.55153191	-4.35106736	-0.65063547
C	-1.35926194	0.26181802	-0.68686867
C	-2.42008619	-3.62377345	-2.70878047
H	-2.90057136	-3.82607408	-3.67518405
H	-2.84623897	-2.69678729	-2.30713589

H	-2.65809382	-4.43581108	-2.01377076
C	-0.35663752	-4.75755011	-3.59040975
H	0.72814124	-4.68095389	-3.72711225
H	-0.82187643	-4.87284532	-4.57817298
H	-0.56404447	-5.65298280	-2.99421833
C	1.88632275	-2.48833677	-0.26581601
C	1.68571407	-3.31184195	0.99020856
C	2.58648176	-4.24231513	1.32516882
H	2.47887375	-4.83012826	2.23417369
H	3.43721397	-4.46920999	0.68653468
H	2.74918452	-2.93237699	-0.79261270
O	1.12895976	-1.68730930	-2.38885748
C	-1.78940515	1.67791692	-0.25283393
H	-1.11916500	2.44403740	-0.66145463
H	-1.79152273	1.76134328	0.83767355
H	-2.80065628	1.88209290	-0.62610076
C	-2.35062888	-0.76097236	-0.10979987
H	-2.42736450	-0.64848970	0.97541990
H	-2.02068499	-1.78236530	-0.32693282
H	-3.34180876	-0.60423435	-0.55483177
C	-1.37119443	0.19395508	-2.21299492
H	-0.68310533	0.91472164	-2.66799426
H	-2.38310889	0.43271499	-2.56426909
H	-1.12019287	-0.80552984	-2.57410761

Anionic Aldol Product 77⁻

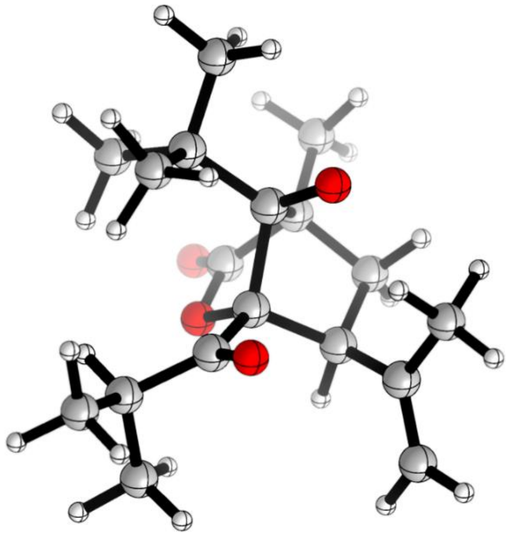

C	0.00000000	0.00000000	0.00000000
O	0.00000000	0.00000000	1.29981998
C	1.49613862	0.00000000	-0.64749262
C	1.25212460	-0.45792059	-2.05128861
C	-0.19996935	-1.50356219	-0.65325622
C	-1.53988681	-2.16507443	-0.58427700
C	-2.27409036	-2.48763953	-1.88636795
H	-2.13021102	-1.65430629	-2.58413010
C	2.36303594	1.23122729	-0.51779288
H	3.41105689	0.99103363	-0.73776948
H	2.29455769	1.60329800	0.51106639
H	2.05762706	2.02368153	-1.20815307
C	2.06621615	-1.21863792	0.11144233
H	3.02866660	-1.54653581	-0.29975391
H	2.19548032	-0.91068098	1.15266829
C	0.39584848	-2.54426604	2.53128087
H	0.31662914	-3.32182774	3.29987104
H	-0.53556422	-1.98020344	2.45134864
H	1.15507122	-1.81407036	2.82828861
O	1.81401438	-0.16702867	-3.08416243
O	-2.01139292	-2.48699071	0.49161011
C	-0.98774288	1.10880583	-0.58339892

C	-3.75893366	-2.72262772	-1.63146697
H	-4.27280724	-2.95410027	-2.57182719
H	-4.23217375	-1.84400414	-1.18145983
H	-3.89754334	-3.55868839	-0.93902904
C	-1.61332445	-3.73920801	-2.48857226
H	-0.55858257	-3.56084779	-2.71456686
H	-2.12206839	-4.02602624	-3.41642981
H	-1.68681505	-4.57601241	-1.78329307
C	0.97689015	-2.31419026	-0.01993580
C	0.72636010	-3.16965797	1.20464569
C	0.82533360	-4.50044077	1.07781230
H	0.66238641	-5.16334448	1.92484185
H	1.05301230	-4.96891485	0.12217937
H	1.27644473	-3.00957645	-0.81482459
O	0.20900270	-1.33052657	-2.03481640
C	-0.67179220	2.41764514	0.16042643
H	0.31824116	2.80635361	-0.09742006
H	-0.70074387	2.24143473	1.23824572
H	-1.41078667	3.18486567	-0.11056156
C	-2.42421478	0.71608832	-0.20851396
H	-2.45425810	0.33827022	0.81810361
H	-2.82376701	-0.05365106	-0.87921694
H	-3.08879700	1.58727495	-0.29329334
C	-0.94318589	1.40285273	-2.08992984
H	0.04147423	1.75492929	-2.41852384
H	-1.66542335	2.20020725	-2.31735671
H	-1.20716057	0.53323793	-2.69994320

Ester Enolate 79⁻

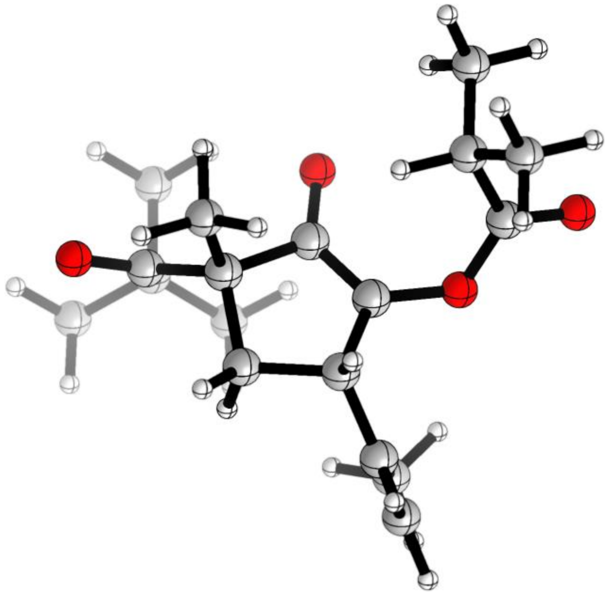

C	0.00000000	0.00000000	0.00000000
O	0.00000000	0.00000000	1.22053665
C	1.34457346	0.00000000	-0.73594327
C	1.38170583	0.65687181	-2.13645594
C	2.12523025	-0.16819163	-2.93779988
C	3.10447643	1.24837161	-4.62419555
C	3.75356549	2.12918337	-3.56287167
H	3.50042631	1.75719693	-2.56950966
C	2.39105064	0.70886085	0.13592930
H	2.40419245	0.30350410	1.15124484
H	2.16818534	1.78021899	0.18467999
H	3.38332912	0.58518560	-0.31460097
C	1.77207657	-1.46317704	-1.01994836
H	2.29346603	-1.90961085	-0.16459028
H	0.89325914	-2.08449261	-1.23171409
C	1.35275286	-2.86188578	-3.94915648
H	0.44816073	-2.78464457	-3.33551395

H	1.35702255	-3.83690585	-4.44687674
H	1.28693782	-2.06973657	-4.70314212
O	0.90323620	1.81060998	-2.36183955
O	3.20625921	1.50819519	-5.80481195
C	-1.37388961	0.00539412	-0.71165816
C	3.23494254	3.56054061	-3.70598228
H	3.43729807	3.93508292	-4.71583645
H	3.73780126	4.21198811	-2.98111542
H	2.15992628	3.58101440	-3.50919033
C	5.27251551	2.06035017	-3.76732964
H	5.64720608	1.03554188	-3.66261609
H	5.77813028	2.68675392	-3.02335057
H	5.53581873	2.42169714	-4.76712051
C	2.66503483	-1.40120853	-2.28825124
C	2.59229739	-2.66866267	-3.11803307
C	3.59089763	-3.55807047	-3.11646236
H	3.54041095	-4.47670892	-3.69694781
H	4.49646477	-3.38577768	-2.53828030
H	3.72283677	-1.28612670	-1.97725461
O	2.46896238	0.11772244	-4.26671267
C	-1.80560934	1.48584029	-0.75878071
H	-1.86260246	1.89724772	0.25556131
H	-2.80091163	1.55343459	-1.21791115
H	-1.09229214	2.06710031	-1.35172028
C	-2.36550295	-0.78555004	0.15269877
H	-2.07840219	-1.84290520	0.21311251
H	-3.36524254	-0.72810457	-0.29503859
H	-2.40750540	-0.38802934	1.16925949
C	-1.39548477	-0.56661271	-2.13428454
H	-0.77068355	0.00268267	-2.82421577
H	-2.43009213	-0.53089788	-2.50081956
H	-1.07971802	-1.61574634	-2.15351624

TS 75⁻ → 89⁻

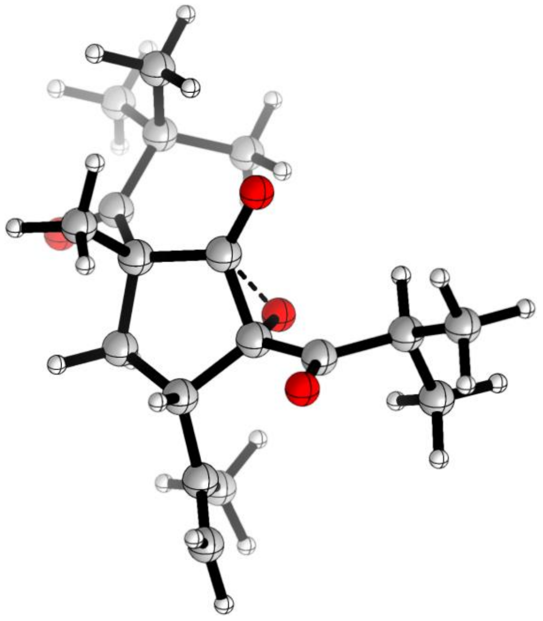

C	0.00000000	0.00000000	0.00000000
O	0.00000000	0.00000000	1.21981886
C	1.37499548	0.00000000	-0.69298074
C	1.49465638	-0.55602495	-2.13221340
C	2.50213716	-1.65699851	-2.09324333
C	3.24251677	-1.90373554	-3.38419091
C	2.52705559	-2.74563344	-4.42257785
H	1.47271282	-2.44765204	-4.38451680
C	1.85824484	1.46584825	-0.75613936
H	1.81903301	1.91812352	0.24117544
H	1.25596348	2.05600256	-1.45148427

H	2.89513210	1.49007518	-1.11187176
C	2.36173033	-0.85083033	0.14501430
H	2.84785210	-0.25514436	0.92550465
H	1.80320063	-1.64714730	0.63862785
C	3.18501762	-3.80016584	0.21517847
H	2.87552512	-3.52445022	1.23106847
H	3.72225790	-4.75302882	0.27008543
H	2.26999200	-3.91603383	-0.37704975
O	0.97344327	-0.04920304	-3.13658442
O	4.35304904	-1.43544422	-3.57694179
C	-1.34838825	0.06069402	-0.74315408
C	2.62863842	-4.21115082	-3.97138613
H	3.67868656	-4.52841436	-3.93659895
H	2.09730844	-4.86180503	-4.67662790
H	2.18363721	-4.33095344	-2.97912214
C	3.10958615	-2.53343407	-5.81372928
H	3.02763792	-1.48482476	-6.11665598
H	2.57884445	-3.15166706	-6.54783403
H	4.17120693	-2.80288453	-5.83226600
C	3.37392703	-1.45606444	-0.84543715
C	4.05242117	-2.72738267	-0.39465635
C	5.36228621	-2.91538747	-0.59741227
H	5.84862928	-3.85189597	-0.33006362
H	5.97470141	-2.14971989	-1.06689626
H	4.14926760	-0.72441233	-1.10527281
O	1.31688154	-2.35848030	-1.86480619
C	-1.40151181	1.29414755	-1.66093286
H	-1.25601187	2.21836107	-1.08586085
H	-2.39497139	1.34260123	-2.12609603
H	-0.64934320	1.22502179	-2.45036134
C	-2.46725861	0.16845198	0.29910314
H	-2.47282756	-0.69827808	0.96703695
H	-3.43353685	0.22092221	-0.21730145
H	-2.35183497	1.06322117	0.91949637
C	-1.56242485	-1.21971652	-1.57126551
H	-0.84931480	-1.30501307	-2.39114238
H	-2.58200748	-1.19687380	-1.97969972
H	-1.45974287	-2.11202594	-0.94366724

TS 89^{-} → 78^{-}

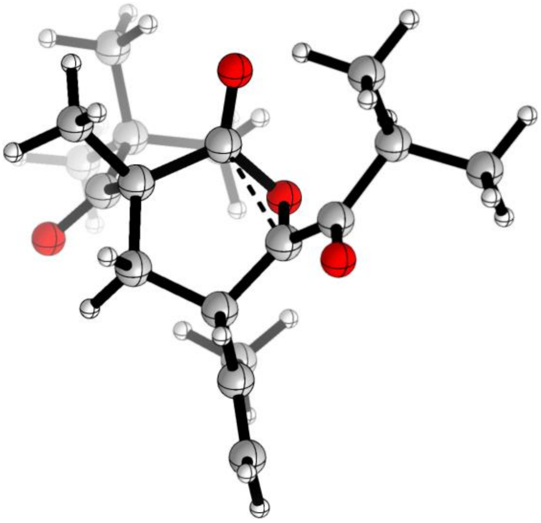

C	0.00000000	0.00000000	0.00000000
O	0.00000000	0.00000000	1.21605162
C	1.38633249	0.00000000	-0.71680788
C	1.27743877	-0.48813534	-2.17356039
C	2.55108557	-2.10053660	-2.00196931
C	3.51659462	-2.17261156	-3.00666164
C	3.09417534	-1.93385586	-4.46391113
H	2.00993207	-1.79789316	-4.51823388
C	1.83673940	1.47658360	-0.71610549

H	1.97126994	1.81114567	0.31803423
H	1.12800304	2.13537917	-1.22118443
H	2.79263156	1.54701463	-1.24449008
C	2.42482914	-0.82610455	0.09728858
H	3.34052540	-0.22507749	0.12113488
H	2.08262376	-0.92459806	1.13262858
C	0.69546057	-3.50228774	0.15616614
H	0.23075448	-2.60332770	0.58154522
H	0.36662471	-4.36659667	0.74344595
H	0.32627606	-3.60119880	-0.86975350
O	1.11237484	0.27201190	-3.11296133
O	4.73405385	-2.41820758	-2.78158179
C	-1.36287010	0.12960921	-0.73015849
C	3.50451241	-3.14360628	-5.30676071
H	4.58344210	-3.30597099	-5.21170834
H	3.25873531	-2.98604325	-6.36536372
H	2.99521114	-4.05267383	-4.96571158
C	3.77984452	-0.66288301	-4.97340224
H	3.43993259	0.21207020	-4.40887954
H	3.55368749	-0.49406985	-6.03474690
H	4.86481071	-0.76050104	-4.85341592
C	2.82276186	-2.18746103	-0.53771581
C	2.19710270	-3.38300483	0.16005814
C	2.96202027	-4.30546380	0.75453415
H	2.52777602	-5.17305225	1.24886631
H	4.04666792	-4.23298403	0.73979738
H	3.90855151	-2.29696600	-0.45305039
O	1.15885694	-1.83877787	-2.29993645
C	-1.42552083	1.45257153	-1.51619195
H	-1.26892268	2.31215324	-0.85308455
H	-2.42571123	1.54665374	-1.95791649
H	-0.69023155	1.48069187	-2.32372488
C	-2.46342337	0.14141395	0.33830134
H	-2.47003214	-0.79290267	0.90879498
H	-3.43697945	0.25959625	-0.15157197
H	-2.32366937	0.96111842	1.04853650
C	-1.63678449	-1.04419693	-1.68917168
H	-1.03374576	-1.00531621	-2.59696809
H	-2.69227897	-0.99835172	-1.98553302
H	-1.46143816	-2.00945507	-1.20355168

TS 78^{-} → 77^{-}

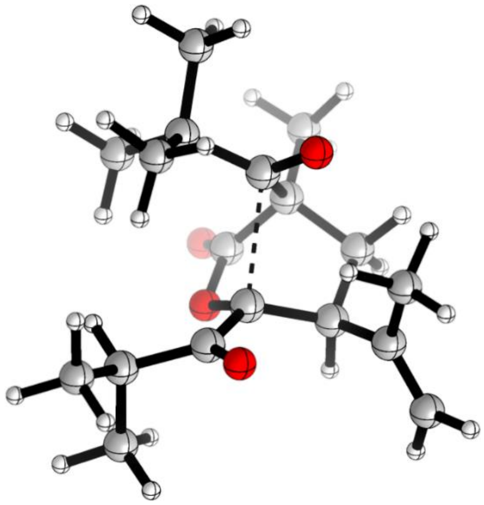

C	0.00000000	0.00000000	0.00000000
O	0.00000000	0.00000000	1.23622764
C	1.39871750	0.00000000	-0.69829736
C	1.30964308	-0.56296606	-2.10103430
C	0.22129038	-2.14752344	-0.81582630
C	-0.88844002	-3.04285944	-0.83141580
C	-1.66280448	-3.22618780	-2.15133415
H	-1.67386770	-2.28182009	-2.70639668

C	2.03504781	1.38474676	-0.69087306
H	3.07234348	1.32506730	-1.03740972
H	2.01765183	1.78915096	0.32695671
H	1.50901420	2.07283452	-1.36003757
C	2.21976150	-1.02936512	0.10755366
H	3.24739354	-1.07421121	-0.27234356
H	2.24971322	-0.69363906	1.14870727
C	0.58775484	-2.62991819	2.39050333
H	0.57950167	-3.33405393	3.22962952
H	-0.43704051	-2.45968872	2.05184734
H	0.97467487	-1.66442052	2.73067228
O	1.81981411	-0.09487360	-3.09839847
O	-1.25577953	-3.69354214	0.16304689
C	-1.25621859	0.64049887	-0.66732480
C	-3.09345454	-3.68893954	-1.89036305
H	-3.62278939	-3.84599321	-2.83863786
H	-3.65376046	-2.95797442	-1.29788901
H	-3.08637145	-4.62605742	-1.32587555
C	-0.90432101	-4.26321017	-2.99314779
H	0.10928776	-3.91947376	-3.21938215
H	-1.42601425	-4.45222052	-3.93999176
H	-0.83922439	-5.20978925	-2.44278436
C	1.51052234	-2.41173510	-0.03896797
C	1.41050210	-3.18387316	1.25999513
C	2.09599897	-4.32561096	1.39550278
H	2.06088478	-4.89891164	2.31943859
H	2.68816107	-4.73693640	0.58104479
H	2.13755237	-3.02235783	-0.70836586
O	0.63340909	-1.71705148	-2.13397697
C	-1.34055939	2.07385987	-0.09979385
H	-0.47380429	2.67772033	-0.39172016
H	-1.38696933	2.04460733	0.99209640
H	-2.24131859	2.56978057	-0.48462419
C	-2.49724406	-0.12533672	-0.19823088
H	-2.47237885	-0.25546256	0.88766014
H	-2.54253175	-1.11582794	-0.65707457
H	-3.40593828	0.42759017	-0.47247832
C	-1.25100957	0.73543250	-2.19501716
H	-0.40782612	1.32472838	-2.57294381
H	-2.17337838	1.23546633	-2.51925450
H	-1.21816323	-0.24628377	-2.67233010

TS $75^{-} \rightarrow 79^{-}$

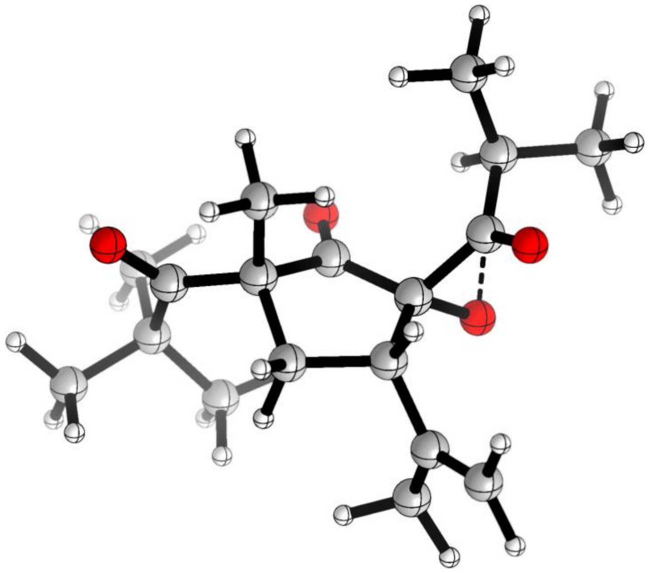

C	0.00000000	0.00000000	0.00000000
O	0.00000000	0.00000000	1.21790368
C	1.33033904	0.00000000	-0.74855515
C	1.39953113	1.01344075	-1.90619279

C	2.33874684	0.48691348	-2.95939884
C	3.64317731	1.24783825	-3.16873697
C	3.60795038	2.77198710	-3.11314489
H	2.56680978	3.09968374	-3.12035816
C	2.47559117	0.34525134	0.21573885
H	2.50376506	-0.37545511	1.03788473
H	2.33068797	1.34339536	0.64043270
H	3.43294269	0.32319854	-0.31680199
C	1.61070918	-1.34834022	-1.45200363
H	2.06053603	-2.06683640	-0.75672216
H	0.68092627	-1.79435300	-1.81647954
C	1.05771168	-1.88537501	-4.53717284
H	0.24244921	-2.25103982	-3.90053911
H	1.07915912	-2.50274605	-5.44123297
H	0.84126905	-0.84670955	-4.81564908
O	0.83089078	2.08971545	-1.88854122
O	4.71385411	0.64386611	-3.31617587
C	-1.38163256	0.00807209	-0.70689717
C	4.32653240	3.33218664	-4.33944676
H	5.35921838	2.96753250	-4.36872047
H	4.33916176	4.42986725	-4.31674070
H	3.81828358	2.99915850	-5.24934740
C	4.28498902	3.23507391	-1.81901400
H	3.74834944	2.86861101	-0.93542926
H	4.31005117	4.33088141	-1.76519968
H	5.31407249	2.85935010	-1.77584873
C	2.55653711	-1.01442433	-2.63450087
C	2.38524539	-1.92067688	-3.82777455
C	3.39319478	-2.68678080	-4.25502717
H	3.28767664	-3.32708583	-5.12886094
H	4.36708167	-2.65265545	-3.77394093
H	3.59818044	-1.10582735	-2.30284438
O	2.22397311	0.91083517	-4.26805281
C	-1.95358904	1.42423863	-0.48925316
H	-1.96158445	1.66935609	0.57762886
H	-2.98471377	1.45559930	-0.86333856
H	-1.35830086	2.17095962	-1.02079021
C	-2.27753071	-1.00469133	0.02396260
H	-1.87827393	-2.02203875	-0.06941530
H	-3.28118587	-0.99114598	-0.41829070
H	-2.35318808	-0.76254235	1.08670721
C	-1.40537202	-0.32047386	-2.20593586
H	-0.67774565	0.24154582	-2.79693624
H	-2.39923930	-0.06814306	-2.59638182
H	-1.25313934	-1.38829485	-2.38929936

α-Hydroxy-β-Diketone Anion 76⁻

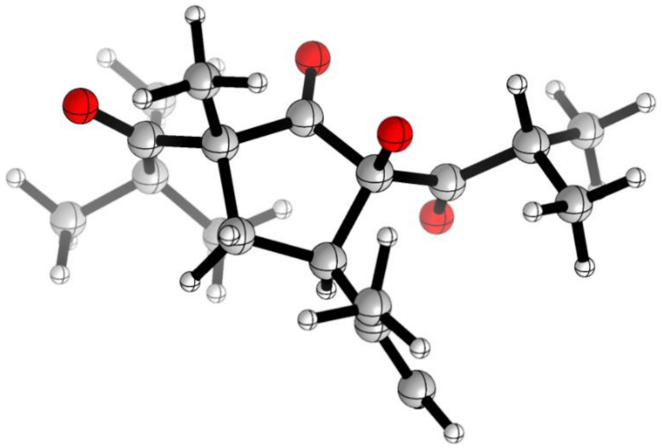

C	0.00000000	0.00000000	0.00000000
O	0.00000000	0.00000000	1.21755934
C	1.33917345	0.00000000	-0.74985387
C	1.39330909	1.02515839	-1.90219191

C	2.22714482	0.40524107	-3.04612202
C	1.78808995	1.01710190	-4.39273631
C	2.83171250	1.83087095	-5.13312609
H	3.37126571	2.39676212	-4.36439115
C	2.46040972	0.35453272	0.24025824
H	2.27969562	1.34121509	0.67784271
H	3.40378348	0.36849828	-0.30999743
H	2.48778970	-0.37938127	1.05223673
C	1.62334533	-1.36222839	-1.45348028
H	2.55268293	-1.76299239	-1.03848457
H	0.84032549	-2.10714438	-1.25785316
C	4.05801705	-2.29375399	-3.12169895
H	4.70982706	-2.79071738	-3.84881424
H	3.98571074	-2.93985402	-2.23694487
H	4.48936002	-1.33564027	-2.80653371
O	0.97760240	2.16241805	-1.81585513
O	0.66119574	0.85567633	-4.83406626
C	-1.38065467	0.01265341	-0.70224612
C	2.19506183	2.74777342	-6.16993599
H	1.64818982	2.16222234	-6.91725120
H	2.96499022	3.33637532	-6.68375261
H	1.48171994	3.43638122	-5.70544631
C	3.83121904	0.85189888	-5.76925731
H	4.28909347	0.23206227	-4.99337273
H	4.61735489	1.40914673	-6.29419130
H	3.32543620	0.20247485	-6.49517383
C	1.77163104	-1.09731395	-2.97277299
C	2.69848454	-2.02699841	-3.71322562
C	2.33646347	-2.53661794	-4.89908436
H	3.01597222	-3.15700410	-5.48073738
H	1.36358029	-2.31914001	-5.33454601
H	0.79076392	-1.16026090	-3.45219877
O	3.50521055	0.63326647	-2.68686742
C	-2.29472623	-0.96687754	0.04942650
H	-3.30432402	-0.92715770	-0.37743545
H	-2.34629075	-0.71601355	1.11154002
H	-1.92694438	-1.99628578	-0.04281412
C	-1.38467002	-0.35177536	-2.18795535
H	-0.76856068	0.30674730	-2.80669663
H	-2.41335935	-0.26719442	-2.56095697
H	-1.06380838	-1.38580664	-2.35260095
C	-1.92840741	1.44362643	-0.53047071
H	-1.29642016	2.16919099	-1.05024511
H	-1.97147455	1.70922353	0.53088809
H	-2.94267282	1.49306695	-0.94680517

Endo Epoxide 90⁻

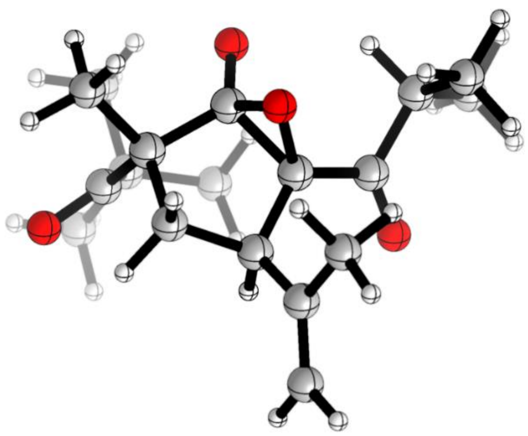

C	0.00000000	0.00000000	0.00000000
O	0.00000000	0.00000000	1.22052966
C	1.40130854	0.00000000	-0.67154421
C	1.43475955	-0.15649308	-2.21529319
C	1.70868685	-1.70359922	-2.42140027

C	1.15095200	-2.39758904	-3.58338458
C	1.35430988	-1.75217964	-4.94628622
H	1.26727767	-0.66843136	-4.78954596
C	2.07307475	1.32781040	-0.29699753
H	1.51296349	2.17000666	-0.71716102
H	3.08175544	1.34616454	-0.72387724
H	2.13336615	1.43611289	0.79071582
C	2.16880042	-1.22098857	-0.12484377
H	3.24220645	-1.00912402	-0.16900013
H	1.90565052	-1.44294138	0.91213447
C	4.15255772	-3.26570385	-1.76386723
H	4.86623241	-4.07608061	-1.58345527
H	4.61027073	-2.31096370	-1.48420906
H	3.95476587	-3.20492980	-2.84111823
O	0.85107307	0.67327706	-2.97609211
O	0.50402746	-3.43482625	-3.44775773
C	-1.36201048	0.06344162	-0.72357025
C	0.30590368	-2.23966453	-5.94044071
H	0.37407371	-3.32450406	-6.07599856
H	0.45098958	-1.75322565	-6.91257242
H	-0.70563015	-2.01458295	-5.58722774
C	2.77686646	-2.06361148	-5.43459613
H	3.52049569	-1.66250365	-4.74003122
H	2.94503659	-1.61173939	-6.41987124
H	2.92182423	-3.14795422	-5.53054107
C	1.83436925	-2.39369914	-1.07693983
C	2.86458640	-3.49903474	-1.01701821
C	2.64233954	-4.62361988	-0.33006829
H	3.39175193	-5.41003112	-0.26536816
H	1.69517905	-4.79729768	0.17529774
H	0.85637071	-2.81847526	-0.81278127
O	2.72308088	-0.70973019	-2.61491368
C	-2.46181549	-0.22374731	0.30728710
H	-3.43948114	-0.14270126	-0.18272818
H	-2.42520978	0.48114098	1.14202013
H	-2.36423213	-1.23436416	0.71957647
C	-1.49283686	-0.94715735	-1.87627828
H	-0.89440074	-0.64820466	-2.73764850
H	-2.54643619	-0.98420101	-2.18266421
H	-1.20237380	-1.96018539	-1.57293615
C	-1.54098219	1.49879343	-1.25681838
H	-0.77205004	1.72633167	-1.99958388
H	-1.49756380	2.22314638	-0.43302366
H	-2.52961919	1.57978347	-1.72834883

Lactone Enolate 87⁻

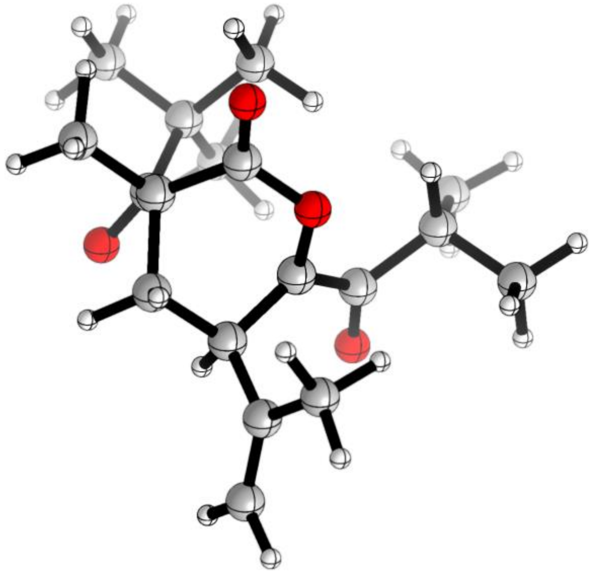

C	0.00000000	0.00000000	0.00000000
O	0.00000000	0.00000000	1.22025359
C	1.38583705	0.00000000	-0.69527550
C	1.47467716	-0.63427489	-2.08347143
C	0.70153517	-2.48030788	-0.87847865
C	-0.34066496	-3.39860741	-0.92076543
C	-0.98301407	-3.74514036	-2.28027732
H	-0.63499680	-3.04985055	-3.05212352
C	1.88766812	1.44443155	-0.75913874
H	2.92641957	1.46328607	-1.10400431
H	1.82763611	1.89826261	0.23553184
H	1.30066689	2.03977145	-1.46562403
C	2.29702914	-0.89886862	0.16971739
H	3.28290313	-0.95091851	-0.31136085
H	2.43259793	-0.45079505	1.15814729
C	3.45891318	-3.72302486	-0.87846682
H	3.94802511	-2.83907258	-1.30612860
H	2.76525988	-4.09175371	-1.64252665
H	4.22192866	-4.48592120	-0.69316040
O	1.89677454	-0.06226667	-3.06838079
O	-0.77957851	-4.00251086	0.09843753
C	-1.33979713	0.27503099	-0.71033985
C	-2.50839434	-3.69829991	-2.18868191
H	-2.87347881	-2.67403779	-2.05050002
H	-2.83641389	-4.29270060	-1.32929698
H	-2.96817233	-4.10156853	-3.10061649
C	-0.51908289	-5.15728320	-2.65928122
H	-0.96282265	-5.47793311	-3.61102012
H	-0.81672919	-5.86194464	-1.87479015
H	0.57188465	-5.19794153	-2.75761026
C	1.64541600	-2.30127916	0.28042914
C	2.70045396	-3.39081810	0.38103828
C	2.91907084	-4.04309773	1.52685236
H	3.66670238	-4.83091891	1.60255855
H	2.32872812	-3.82965322	2.41445514
H	1.03437643	-2.33540495	1.18877211
O	1.19154100	-1.93699168	-2.11255742
C	-1.68046780	1.75484963	-0.44008284
H	-1.66623257	1.95976731	0.63494451
H	-2.68397817	1.97425934	-0.82637109
H	-0.97418521	2.43178583	-0.93514437
C	-2.39710916	-0.61024176	-0.03376251
H	-2.10853369	-1.66590134	-0.07091275
H	-3.36287229	-0.48235961	-0.53996629
H	-2.50974614	-0.33236658	1.01770818
C	-1.33404055	0.03645724	-2.21971583
H	-1.10591278	-1.00541041	-2.46030827
H	-0.61602490	0.68051081	-2.73969890
H	-2.33135788	0.26257687	-2.61810126

Anionic Aldol Product 80⁻

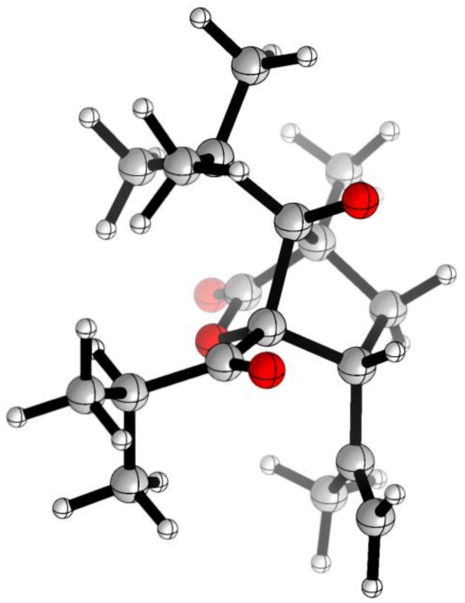

C	0.00000000	0.00000000	0.00000000
O	0.00000000	0.00000000	1.29608957
C	1.49116654	0.00000000	-0.64563175
C	1.27517289	-0.47476867	-2.04529126
C	-0.18422039	-1.53612884	-0.66667994
C	-1.50631481	-2.22821554	-0.61781523
C	-2.28118963	-2.45475185	-1.91761654
H	-2.14101613	-1.58351952	-2.56784483
C	2.35656379	1.23203461	-0.51766872
H	3.40545911	0.98997857	-0.73272898
H	2.28389596	1.60819998	0.50933065
H	2.05420594	2.02134245	-1.21313316
C	2.06974429	-1.22596982	0.10602926
H	2.98101087	-1.59082177	-0.38308175
H	2.31177269	-0.90512738	1.12288175
C	2.12508542	-3.81839381	-1.58302882
H	3.16138662	-3.55373922	-1.34042477
H	1.81640407	-3.16768922	-2.40801540
H	2.11725914	-4.85651714	-1.93161887
O	1.85473268	-0.19703952	-3.07301890
O	-1.93298771	-2.66134494	0.43968330
C	-1.00105958	1.08615492	-0.58990318
C	-3.76375530	-2.68064503	-1.63750791
H	-4.21214844	-1.82517390	-1.12234028
H	-3.89679482	-3.55594580	-0.99431864
H	-4.30542104	-2.84688137	-2.57611693
C	-1.66825935	-3.67971675	-2.61748807
H	-2.20683974	-3.88638701	-3.55003977
H	-1.74704881	-4.55933333	-1.96789745
H	-0.61304780	-3.51836929	-2.85357332
C	0.91957765	-2.26399959	0.13460588
C	1.22594880	-3.65436472	-0.38043645
C	0.67818184	-4.72228514	0.21334688
H	0.84576517	-5.72933263	-0.16534827
H	0.02555748	-4.61167450	1.07537378
H	0.51753167	-2.32862648	1.14764223
O	0.26008885	-1.37820508	-2.03236319
C	-0.69443282	2.40993308	0.13142391
H	-0.72120947	2.25077645	1.21209727
H	-1.43959609	3.16713256	-0.15083887
H	0.29266838	2.80165512	-0.13350332
C	-2.42775133	0.67954513	-0.19617963
H	-2.82227490	-0.10304308	-0.85331962
H	-3.10641108	1.53975030	-0.28197358

H	-2.43825274	0.31298021	0.83504768
C	-0.96130172	1.35340505	-2.10106598
H	-1.20719692	0.46821805	-2.69568924
H	0.01793263	1.71725988	-2.43407563
H	-1.69758057	2.13281859	-2.34510628

Anionic Ester Enolate 88⁻

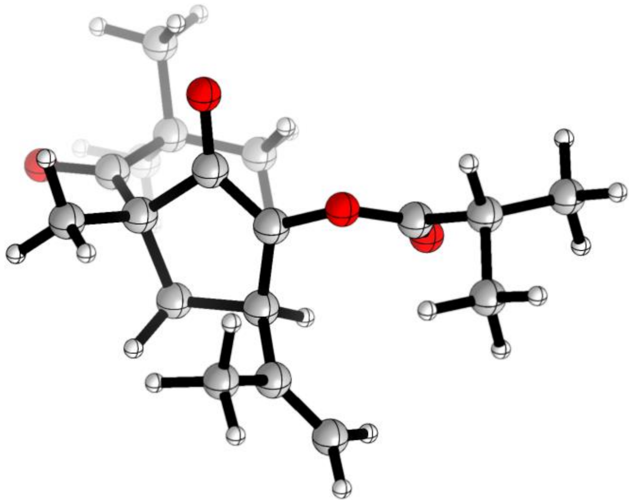

C	0.00000000	0.00000000	0.00000000
O	0.00000000	0.00000000	1.22010025
C	1.32540954	0.00000000	-0.76073753
C	1.32465707	0.97659309	-1.96521054
C	1.72317500	0.25467809	-3.06037236
C	1.39681837	0.42455455	-5.39101461
C	2.03483414	1.01919570	-6.63798455
H	2.27395720	2.06475358	-6.41011650
C	2.44116735	0.42715912	0.19695611
H	2.25195216	1.44134097	0.56022633
H	3.39434289	0.42577895	-0.34322382
H	2.50839261	-0.24662190	1.05771093
C	1.59906568	-1.40251485	-1.37850453
H	2.44594950	-1.87458318	-0.86577411
H	0.74726451	-2.07999196	-1.24275194
C	4.45446659	-1.07762847	-2.76852589
H	5.37603896	-1.48933794	-3.19288949
H	4.48168342	-1.21571483	-1.68070126
H	4.42441520	0.00387495	-2.94604333
O	1.01523691	2.19127674	-1.81130519
O	0.48774177	-0.38002929	-5.41388688
C	-1.38483395	0.01122235	-0.69816345
C	1.08398125	0.93835584	-7.82672580
H	0.82644786	-0.10500259	-8.03382473
H	1.55167884	1.37018766	-8.71911703
H	0.15143468	1.47471906	-7.62670474
C	3.34608718	0.26862110	-6.91310559
H	4.02116587	0.33266628	-6.05478054
H	3.84880185	0.68791147	-7.79280213
H	3.14178210	-0.79158691	-7.10312318
C	1.88032074	-1.21986773	-2.90139709
C	3.23503304	-1.72519790	-3.36865339
C	3.33084328	-2.64920651	-4.33171309
H	4.29442016	-2.99243454	-4.70472194
H	2.43866168	-3.07350926	-4.78791131
H	1.12730671	-1.77869893	-3.48178742
O	2.01520553	0.86328987	-4.29684358
C	-2.25731742	-1.05104361	-0.01079120
H	-3.28688019	-0.97850346	-0.38293871
H	-2.25964187	-0.91136619	1.07318499
H	-1.89025208	-2.06230547	-0.22938264
C	-1.40756964	-0.22791607	-2.21265255

H	-0.96285092	0.59994051	-2.76647549
H	-2.45648747	-0.32464528	-2.52467255
H	-0.89128738	-1.14641852	-2.51171330
C	-1.96256401	1.41230131	-0.41843888
H	-1.30723094	2.17677325	-0.84987076
H	-2.05185676	1.58168022	0.65979221
H	-2.95847178	1.49233929	-0.87424580

TS 76⁻ → 90⁻

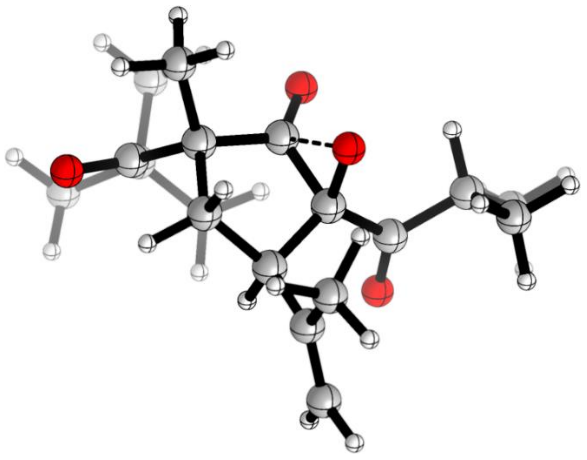

C	0.00000000	0.00000000	0.00000000
O	0.00000000	0.00000000	1.21908632
C	1.39975019	0.00000000	-0.67861414
C	1.40929870	-0.14513168	-2.21730099
C	2.02658607	-1.47535223	-2.53381767
C	1.55821176	-2.13824879	-3.80170581
C	2.21096289	-1.66586265	-5.08548046
H	2.33324608	-0.58079706	-4.98037654
C	2.07697205	1.31910708	-0.28466495
H	1.52566499	2.17056189	-0.69964604
H	3.08416295	1.32251979	-0.71152179
H	2.12501316	1.41590702	0.80463477
C	2.16588754	-1.24578332	-0.17060826
H	3.22548324	-0.98619407	-0.11084443
H	1.82505579	-1.56227214	0.81911003
C	4.42471774	-3.08565738	-1.08122132
H	5.07405985	-3.93470805	-1.32017310
H	4.63027086	-2.77857782	-0.04776218
H	4.66700775	-2.23048590	-1.72263677
O	0.92771839	0.68288315	-3.00133011
O	0.67924118	-2.98699636	-3.78741085
C	-1.35757690	0.05060806	-0.73034920
C	1.36804463	-2.01273945	-6.30569118
H	1.22556250	-3.09631375	-6.38083820
H	1.85880189	-1.66015648	-7.22087334
H	0.37705613	-1.55189570	-6.24273572
C	3.60876301	-2.30142319	-5.14930261
H	4.19199032	-2.01282351	-4.26983516
H	4.13709562	-1.96338375	-6.04911800
H	3.53044670	-3.39547380	-5.18832929
C	1.96624383	-2.32705880	-1.25240463
C	2.97206797	-3.45227330	-1.25312575
C	2.58782129	-4.71722133	-1.46359348
H	3.31253048	-5.52750858	-1.52055783
H	1.54169487	-4.96969297	-1.61695070
H	0.95977719	-2.76039928	-1.18110597
O	3.14671459	-0.64876344	-2.57806013
C	-2.46784863	-0.17210372	0.30409029
H	-3.44145465	-0.10959441	-0.19676545
H	-2.43073442	0.57778646	1.09882607
H	-2.38078957	-1.15800169	0.77340649
C	-1.48002513	-1.03140830	-1.81612426

H	-0.82999710	-0.85088823	-2.67180096
H	-2.51547251	-1.04185562	-2.18003485
H	-1.26120771	-2.02871170	-1.41677715
C	-1.52590983	1.45512074	-1.34188340
H	-0.75085459	1.65786219	-2.08449713
H	-1.49128213	2.21961145	-0.55512473
H	-2.50878917	1.51388365	-1.82792204

TS 90⁻ → 87⁻

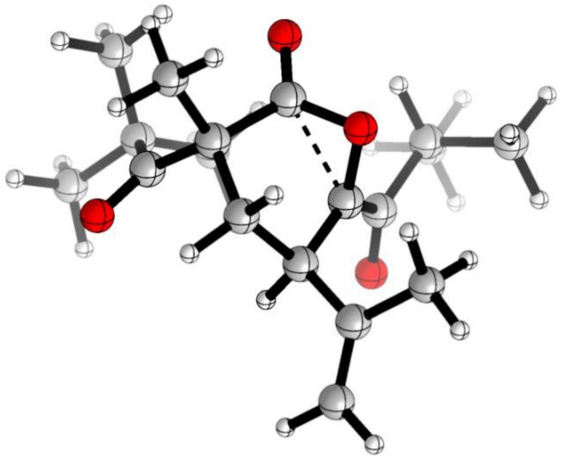

C	0.00000000	0.00000000	0.00000000
O	0.00000000	0.00000000	1.21813115
C	1.40917646	0.00000000	-0.66775612
C	1.47683820	-0.34254645	-2.17131290
C	1.60043611	-2.27957648	-1.86187315
C	0.73999879	-3.08516190	-2.61681043
C	0.64219664	-2.80948788	-4.12525035
H	0.69992134	-1.72600543	-4.28985658
C	1.96474292	1.42922514	-0.49281820
H	1.35397403	2.15854896	-1.03329200
H	2.98340900	1.47675935	-0.89378332
H	1.98833800	1.69074770	0.57002429
C	2.28401401	-1.01598357	0.08305733
H	3.33247380	-0.80795759	-0.16029891
H	2.15720273	-0.91209494	1.16259401
C	4.19150881	-3.43644474	-1.02172184
H	4.97145022	-4.12306718	-0.67689662
H	4.62311322	-2.43500219	-1.13329666
H	3.86119656	-3.74354688	-2.02110240
O	0.92911005	0.33537731	-3.03366830
O	0.06134606	-4.01266659	-2.10956247
C	-1.35713959	0.10674706	-0.72913324
C	-0.67415145	-3.34403682	-4.68171313
H	-0.74849247	-4.42261582	-4.51073153
H	-0.74425354	-3.14468181	-5.75886035
H	-1.53143902	-2.87738826	-4.18431583
C	1.84216263	-3.46097438	-4.82487602
H	2.78076595	-3.02374865	-4.46876502
H	1.78679683	-3.32131283	-5.91268309
H	1.85789016	-4.53899259	-4.61892109
C	1.90747717	-2.43438083	-0.40294986
C	3.01330787	-3.42601698	-0.08309779
C	2.93800158	-4.24403961	0.97075429
H	3.73606813	-4.94473105	1.20942872
H	2.06158016	-4.25086003	1.61482349
H	0.99274430	-2.76762822	0.10624124
O	2.42769083	-1.27787478	-2.49391987
C	-2.46481613	0.00140375	0.32798697
H	-3.43757890	0.10790061	-0.16623899
H	-2.36966382	0.77848615	1.09166580
H	-2.43613898	-0.96918499	0.83322992
C	-1.57749470	-1.00456590	-1.77076811

H	-0.95939329	-0.86198614	-2.65752184
H	-2.63038173	-0.97284561	-2.08163200
H	-1.37343249	-2.00178953	-1.36394588
C	-1.44868508	1.49233279	-1.39739739
H	-0.69834797	1.60139473	-2.18275126
H	-1.32710995	2.29238163	-0.65530151
H	-2.44399766	1.59929920	-1.84770811

TS 87^{-} → 80^{-}

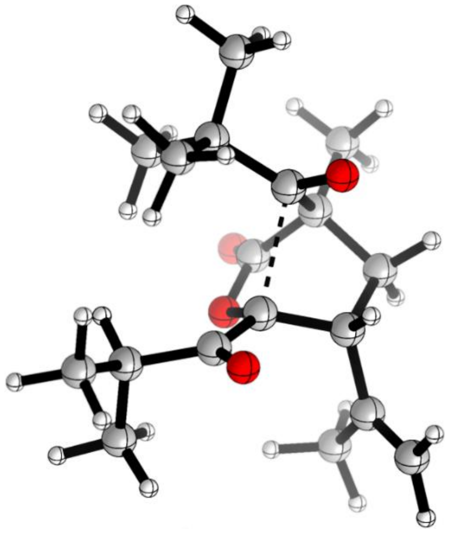

C	0.00000000	0.00000000	0.00000000
O	0.00000000	0.00000000	1.24178126
C	1.40636159	0.00000000	-0.69237366
C	1.33511245	-0.62227524	-2.06840195
C	0.17528697	-2.08975743	-0.72968076
C	-0.96849313	-2.94145862	-0.67681214
C	-1.77178546	-3.19613608	-1.96520739
H	-1.80096800	-2.27643472	-2.56183383
C	2.06806312	1.36990828	-0.72441852
H	3.11701939	1.27396819	-1.02741904
H	2.02342909	1.81904093	0.27376436
H	1.58294352	2.03860904	-1.44232699
C	2.20550457	-1.02862961	0.14028228
H	3.16259161	-1.23919556	-0.35405201
H	2.41608329	-0.60513308	1.12651376
C	2.69148632	-3.87295362	-1.25011908
H	3.33233300	-3.04954352	-1.58755801
H	1.91581928	-3.98880760	-2.01461831
H	3.29139718	-4.78839010	-1.21727224
O	1.87558006	-0.22228771	-3.07866992
O	-1.33008523	-3.49099070	0.37711989
C	-1.22808014	0.68141118	-0.67610774
C	-3.19341849	-3.64640865	-1.63985760
H	-3.72758604	-2.90023245	-1.04291177
H	-3.16845584	-4.56978019	-1.05328769
H	-3.75781839	-3.82498370	-2.56359337
C	-1.04062304	-4.26861880	-2.78639080
H	-1.63149152	-4.55122428	-3.66708175
H	-0.88595269	-5.16656229	-2.17522659
H	-0.06777186	-3.90477063	-3.12841328
C	1.30718191	-2.28797098	0.26019205
C	2.06260223	-3.58964290	0.09163016
C	2.13163355	-4.47687540	1.09005819
H	2.65802755	-5.42319496	0.97582864
H	1.63286312	-4.29386225	2.03803433
H	0.83021216	-2.28639337	1.24424147
O	0.65134933	-1.77725619	-2.04929983
C	-1.25123395	2.12971651	-0.14354372
H	-1.29901142	2.12568307	0.94896277

H	-2.13018899	2.65546887	-0.53976318
H	-0.35981114	2.68983979	-0.44709754
C	-2.50358546	-0.01504604	-0.19260752
H	-2.61040744	-1.00182765	-0.64936267
H	-3.38404727	0.58539992	-0.45941852
H	-2.47279453	-0.14607725	0.89273879
C	-1.21155082	0.73285383	-2.20589791
H	-1.19959757	-0.26397684	-2.65371101
H	-0.35058670	1.28866078	-2.59488667
H	-2.11818707	1.24567341	-2.55421291

TS 76⁻ → 88⁻

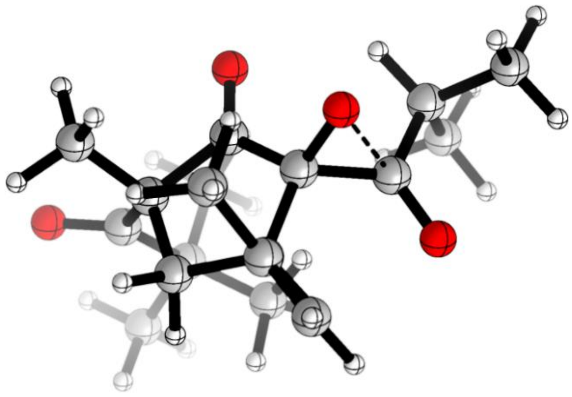

C	0.00000000	0.00000000	0.00000000
O	0.00000000	0.00000000	1.21725753
C	1.33451720	0.00000000	-0.75452078
C	1.45372768	1.03400087	-1.89713471
C	1.85254382	0.37685822	-3.20431985
C	1.05014783	0.69100423	-4.46099717
C	0.60620138	2.13165576	-4.71270827
H	1.02857784	2.77148965	-3.93638613
C	2.45872115	0.36271475	0.22676387
H	2.27676495	1.34912550	0.66094868
H	3.41684069	0.38604253	-0.30232918
H	2.50921778	-0.37522706	1.03236391
C	1.59746969	-1.37542861	-1.42372486
H	2.40313881	-1.88544425	-0.88367141
H	0.72638328	-2.03396314	-1.34866650
C	4.52034318	-1.09684319	-2.58609981
H	5.45514633	-1.52726355	-2.96005916
H	4.48380599	-1.26065318	-1.50118135
H	4.52031254	-0.01623865	-2.77576136
O	1.31239611	2.22464766	-1.69668534
O	0.75028819	-0.20118349	-5.26445645
C	-1.36191433	0.02318354	-0.72944947
C	-0.92428831	2.20427938	-4.67289088
H	-1.35319088	1.50065939	-5.39620839
H	-1.26822436	3.21617421	-4.92243172
H	-1.31741617	1.95427017	-3.68062558
C	1.12952303	2.57625568	-6.07878171
H	2.22219306	2.53323469	-6.08439862
H	0.80638046	3.60124741	-6.30441458
H	0.75159094	1.90824184	-6.86088439
C	1.97159126	-1.14260374	-2.91618105
C	3.32747053	-1.69829375	-3.27891497
C	3.45245468	-2.65988448	-4.19790355
H	4.42796571	-3.05317752	-4.47807924
H	2.58630952	-3.04179347	-4.73194137
H	1.23477909	-1.63408588	-3.56222218
O	2.81479614	0.99180322	-3.98094716
C	-2.38440464	-0.74233518	0.11970287

H	-3.36806727	-0.69354314	-0.36266498
H	-2.45879550	-0.31956835	1.12429474
H	-2.10224161	-1.79755047	0.21533370
C	-1.36157351	-0.55988185	-2.14722281
H	-0.59334977	-0.14056141	-2.79987545
H	-2.32749041	-0.34189847	-2.61903858
H	-1.23863728	-1.64804652	-2.14046749
C	-1.77218742	1.51099776	-0.77963723
H	-1.05892933	2.11161023	-1.35005147
H	-1.83173140	1.92095268	0.23433700
H	-2.75949571	1.59538352	-1.25074709
